# Peptide Thioester and Triazole Derivatives Through On‐Resin Dual‐Modification of Peptide Thiosulfonates

**DOI:** 10.1002/anie.8315692

**Published:** 2026-07-13

**Authors:** Marius Werner, Agnes Bergmann, Chenxi Liu, Mira Behnam, Christian Klein, Franziska Thomas

**Affiliations:** ^1^ Institute of Organic Chemistry Heidelberg University Heidelberg Germany; ^2^ Institute of Pharmacy and Molecular Biotechnology (IPMB) Heidelberg University Heidelberg Germany

**Keywords:** click chemistry, dengue protease inhibitors, late‐stage functionalization, peptide thioesters, peptides

## Abstract

Modified peptides are an important class of drugs due to their high specificity and affinity in particular to pharmacological targets that are not druggable by conventional small molecules. Methods that allow the efficient diversification of peptides with non‐canonical modifications are therefore highly sought after. One possible strategy for accessing broadly diversified peptides is on‐resin late‐stage modification, whereby a functional site—typically an amino acid side chain or the N‐terminus—is usually singly modified after peptide chain assembly, while the other functionalities remain protected. Here, we present an on‐resin late‐stage dual‐modification approach. This approach involves a reaction sequence that includes copper‐catalyzed S‐alkynylation of a resin‐bound peptide thiosulfonate, followed by an iridium‐catalyzed azide‐alkyne click reaction (IrAAC). Thus, peptides with complex non‐canonical modifications are obtained. The dual‐modification approach is highly modular and compatible with a wide range of alkynes for S‐alkynylation and azides for IrAAC. Furthermore, this chemistry can be used to modify complex peptides, such as the 34‐amino‐acid WW domain. In a structure‐activity relationship study of triazole‐containing peptide protease inhibitors of the dengue virus, we demonstrate the great potential of the modular on‐resin dual‐modification of peptide thiosulfonates for modulating peptide‐protein interactions.

## Graphical abstract

An on‐resin dual‐modification strategy for peptides is presented, comprising S‐alkynylation of peptide thiosulfonates and regioselective iridium‐catalyzed azide–thioalkyne cycloaddition. This method reliably provides access to peptides with intricate non‐canonical modifications, being compatible with complex peptides, alkynes and azides. We further present the structure‐activity relationship for triazole‐modified dengue virus protease inhibitors.

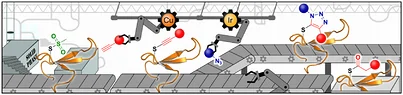

## 1 Introduction

Chemical modification of peptides is a means to tailor their function and properties. This can include their structural stability, solubility, pharmacokinetics and interaction with biological target molecules; all of which are important factors in the development of peptide pharmaceuticals [1, 2, 3, 4, 5]. In the field of drug discovery, there is an increasing demand for synthetic strategies that reliably and easily enable peptide diversification in order to generate large libraries of modified peptides [6]. In this context, late‐stage functionalization of peptides on the solid phase has gained increasing interest as it perfectly allows for parallelization and automation in a time‐efficient manner [7]. Compared to bioconjugation reactions that occur in water under mild conditions, the solid‐phase approach is compatible with a wider range of chemical reactions, including those that proceed under harsher conditions and in non‐aqueous solvents. In recent years, various methods have emerged in this field, including cross‐coupling reactions and photocatalyzed reactions, to name but a few [8, 9, 10, 11, 12, 13]. Of particular interest are multicomponent reactions and methods that introduce functional handles for further modification, as these offer an even greater range of diversification [14, 15, 16, 17].

**FIGURE 1:**
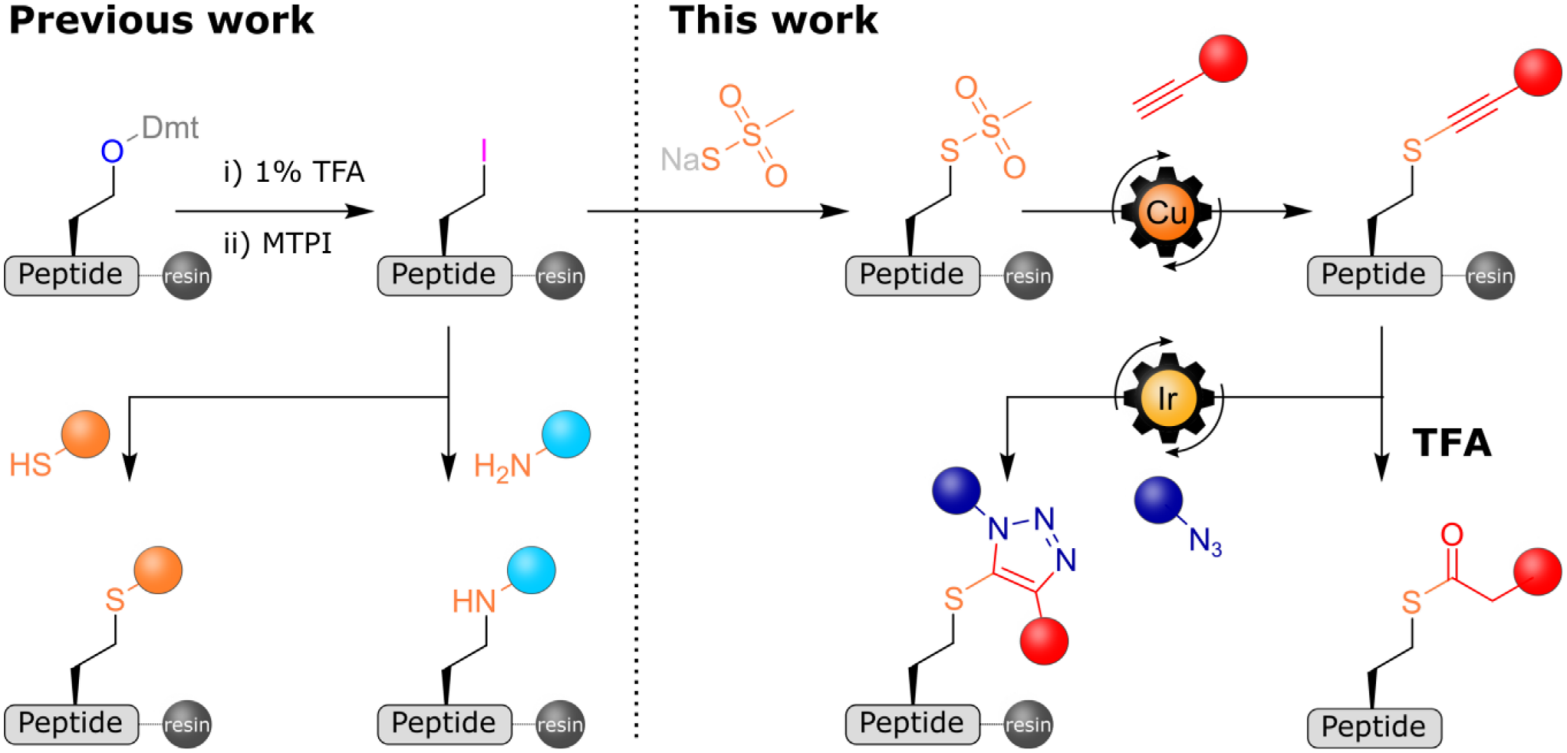
Functionalization of resin‐bound peptides by an iodination‐substitution approach. Iodohomoalanine is prepared via selective on‐resin iodination of homoserine and can be derivatized using various nucleophiles, including thiols and amines (left). Substitution with sodium methanethiosulfonate provides access to peptide thiosulfonates that are susceptible to S‐alkynylation. Thioalkynes then form thioesters upon acidic cleavage from the resin, but can be subjected to further modification on the solid phase in a regioselective IrAAC (right).

In previous studies, we have demonstrated that a simple reaction sequence involving on‐resin iodination, followed by nucleophilic substitution using either sulfur nucleophiles or amines, provides access to a wide variety of structural modifications (Figure 1) [18, 19]. One specific modification that caught our interest was the thiosulfonate moiety, which is accessible by substitution with the corresponding thiosulfonate salt [20]. The polarization of the S‐S bond in thiosulfonates results in an electrophilic sulfur that can react with nucleophiles [21]. For instance, when thiols are used, asymmetric disulfides are obtained, which has previously been utilized to label cysteine‐containing proteins [20, 22, 23]. Despite their potential in chemical transformations, thiosulfonates have been relatively underexplored in bioorganic chemistry due to their perceived difficulty in synthesis and limited commercial availability [24].

Recently, several chemical reactions involving thiosulfonates have been described in the literature. These include the copper‐catalyzed cross‐coupling reaction with boronic acids, the photocatalyzed thioester synthesis with aldehydes, the thiosulfonylation of unsaturated bonds and the copper‐catalyzed alkynylation [25, 26, 27, 28, 29, 30, 31]. Of these reactions, we were particularly interested in the copper‐catalyzed alkynylation reaction, since the resulting thioalkynes are themselves useful intermediates for further modifications, such as the regioselective iridium‐catalyzed azide‐alkyne click reaction (IrAAC), thiol addition, pyrimidine synthesis and hydrofunctionalization [32, 33, 34, 35, 36, 37, 38]. Although the introduction of thioalkynes into peptides has been described in solution, this has only been demonstrated using relatively simple examples. For instance, Guo et al. demonstrated S‐alkynylation of cysteine residues in fully protected dipeptides using a continuous flow method [39]. More recently, Xu et al. described copper‐catalyzed S‐alkynylation in solution using simple alkynes on fully protected dipeptides. They also demonstrated the viability of this reaction as a means of peptide cyclization [40]. The only viable method for accessing thioalkynes in complex peptides, as detailed in the literature, involves the functionalization of cysteine residues using hypervalent iodine reagents [41, 42]. This reaction can be performed on unprotected peptides in solution; however, the range of alkynes that can be used is limited. Copper‐catalyzed S‐alkynylation on the solid phase has not yet been demonstrated.

We hypothesized that copper‐catalyzed alkynylation should have greater potential for peptide diversification when applied to resin‐bound peptides. While the formation of disulfides in solution limits the applicability of the S‐alkynylation, thiosulfonate moieties on resin‐bound peptides are spatially separated, which should minimize possible side reactions. However, some thiosulfonates have been described to be sensitive to air and moisture, which would pose a challenge [24]. Using our iodination‐substitution approach, we successfully introduced the methylthiosulfonate group into resin‐bound peptides in excellent yields (Figure 1). We then used this as a starting point to explore copper‐catalyzed S‐alkynylation on the resin. A broad range of structurally diverse alkynes could be introduced, some of which had unprotected functional groups. During acidic cleavage, the thioalkyne moieties are converted to thioesters, which are important reactive handles in chemical biology—for example, native chemical ligation—and play a role in biological processes such as the non‐ribosomal peptide synthesis [43, 44, 45, 46, 47]. Having a dual‐modification strategy in mind, we explored further modification of the peptide thioalkynes using IrAAC and synthesized 1,5‐triazoles with high regioselectivity and good yields. Despite the four‐step sequence of iodination, substitution, S‐alkynylation, and cycloaddition, we observed complete conversion to the desired cycloaddition product. Finally, we demonstrated the potential of our sequential late‐stage modification approach in a structure‐activity relationship study of peptide inhibitors of the dengue virus protease. One of the peptide inhibitor variants obtained via the on‐resin alkynylation‐IrAAC reaction sequence exhibited improved inhibitory activity by one order of magnitude compared to the lead structure. This impressively illustrates the efficiency of on‐resin late‐stage functionalization approaches in enabling the high‐throughput synthesis of peptides with non‐canonical modifications, which is difficult to achieve with in‐solution approaches.

## 2 Results and Discussion

### 2.1 Synthesis of Peptide Thiosulfonates

In our previous work on late‐stage peptide iodination and substitution, we demonstrated the introduction of p‐toluenethiosulfonate into the GFXFGG peptide (X indicates the position of the modified residue) [18]. As outlined above, the diverse possibilities for downstream chemical transformations offered by this functional group inspired us to explore the use of peptide thiosulfonates in late‐stage diversification reactions. However, GFXFGG is merely a simple test peptide, lacking diversity of amino acid residues. Therefore, we selected the peptide **P1**, a peptide epitope derived from the influenza virus M1 matrix protein, as a reference test peptide to explore thiosulfonate chemistry [48]. **P1** is a 12‐amino acid residue peptide that exhibits a variety of functional groups, thus representing the typical amino acid composition of peptides of interest, and furthermore has a biological relevance in immunology [49]. As the project progressed, we also investigated more complex peptides covering almost all canonical amino acids except cysteine and aspartate (Table 1).

**TABLE 1:** Sequences of peptides used in the study. X: Position of Dmt‐protected ʟ‐homoserine; x: Position of Dmt‐protected d‐homoserine.

Peptide	Sequence
**P1** ^a^	H‐GPXKAEIAQRLE‐NH_2_
**P2** ^a^	H‐KSAFXILPSIISNEK‐OH
**P3** ^a^	H‐KAPRKQXATKAARMSAPSTGGVKKPHR‐OH
**P4**	Ac‐KLPPGWEKR*Nle*SRSSGRVYYFNHITNASQXERPSG‐NH_2_
**P5**	Ac‐KXK‐NH_2_
**P6**	Bz‐RKx‐NH_2_
**P7**	Bz‐RKm‐NH_2_

^a^ **P1**, **P2,** and **P3** were N‐terminally protected with a *tert*‐butoxycarbonyl (Boc) group during SPPS.

**FIGURE 2:**
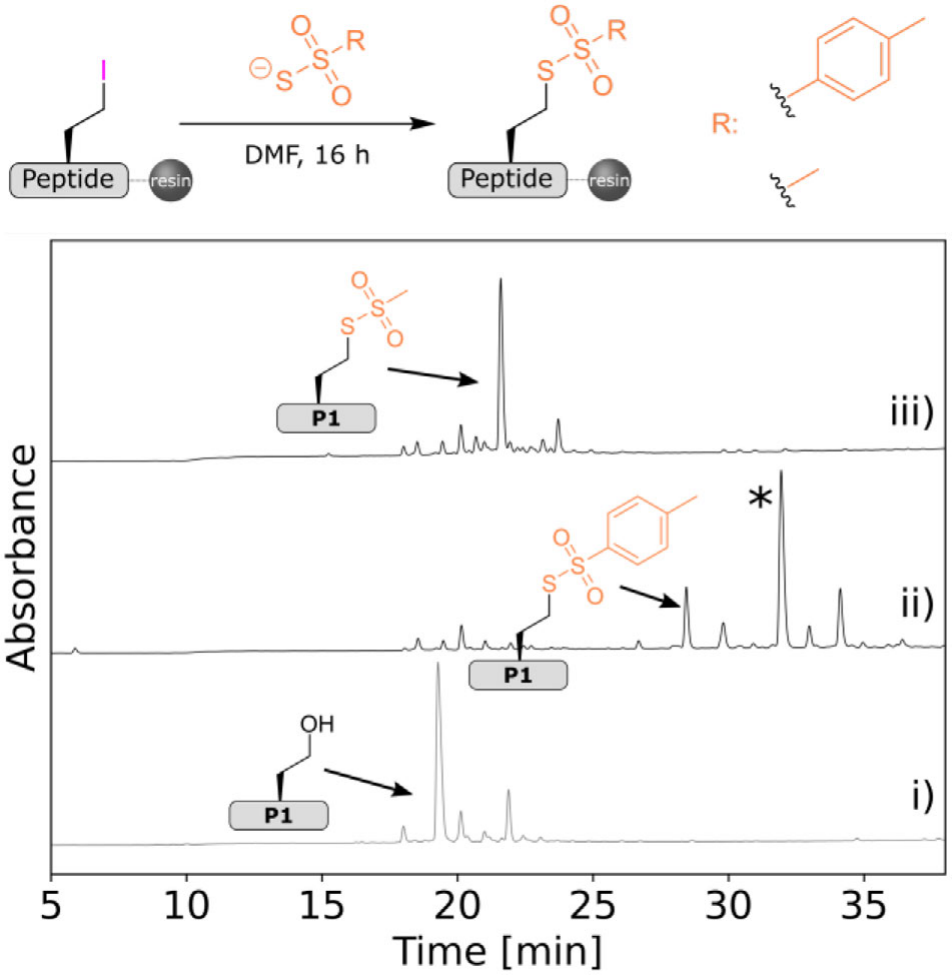
On‐resin synthesis of peptide thiosulfonates. Thiosulfonates are prepared on the solid phase via an iodination‐substitution reaction sequence using the corresponding thiosulfonate salt at 500 mm concentration in DMF in an overnight reaction at room temperature. The HPLC traces show: (i) unmodified **P1–OH** (**OH** refers to the unmodified homoserine‐containing peptide); (ii) **P1** after substitution with potassium p‐toluenethiosulfonate, giving the desired peptide thiosulfonate as a minor product (*major byproduct with a mass difference ‐32), (iii) **P1** after substitution with sodium methanethiosulfonate, yielding the peptide thiosulfonate in high yield.

*p*‐Toluenethiosulfonate was introduced into **P1** using our previously reported late‐stage iodination‐substitution approach [18]. Briefly, the peptide chain was synthesized by Fmoc‐based solid‐phase peptide synthesis (SPPS), with a 4,4′‐dimethoxytrityl (Dmt) protected homoserine introduced at position 3. Homoserine was used instead of serine, since the corresponding iodoalanine is prone to 2‐oxazoline formation and is easily eliminated under basic conditions, leading to the formation of dehydroalanine [50, 51]. After the Dmt protecting group was removed under mildly acidic conditions, on‐resin iodination was achieved via an Appel‐type reaction with methyltriphenoxyphosphonium iodide (MTPI). This was followed by an overnight on‐resin nucleophilic substitution at room temperature using 500 mm potassium p‐toluenethiosulfonate in *N,N*‐dimethylformamide (DMF). However, although the starting material was fully converted, the main product identified by HPLC and mass spectrometry was not the desired peptide thiosulfonate, but a by‐product with a mass 32 lower than that of the expected thiosulfonate, possibly indicating sulfonylation (Figures 2 and S1, S2). Therefore, we opted for the less reactive sodium methanethiosulfonate and obtained the respective peptide thiosulfonate in high yield (Figures 2iii and S3, S4).

### 2.2 Optimization of the S‐Alkynylation of Peptide Thiosulfonates

Having developed an optimized protocol for synthesizing peptide thiosulfonates, we decided to explore on‐resin S‐alkynylation. To this end, we adapted the conditions for copper‐catalyzed S‐alkynylation based on the work of Hosoya et al. and Xu et al. (Table 2, entry 1) [31, 40]. Successful S‐alkynylation with complete conversion was demonstrated for the modification of **P1** with phenylacetylene **A1**, which, upon acidic cleavage from the resin with trifluoroacetic acid (TFA), furnished the respective peptide thioester (Figures S5, S6) [42, 52]. However, low yields were obtained with aliphatic alkynes, such as 1‐hexyne, which is consistent with previous reports of incomplete conversion in copper‐catalyzed S‐alkynylation with aliphatic alkynes, even at increased amounts of reactants and elevated temperatures [31, 40]. This prompted us to optimize the alkynylation conditions (Table 2). We achieved complete conversion with 1‐hexyne by reducing the temperature to room temperature and using Cs_2_CO_3_ instead of K_2_CO_3_ as a base (Table 2, entry 5; Figures S7, S8). Interestingly, the use of Cs_2_CO_3_ in the S‐alkynylation reaction has previously been reported to produce only trace amounts of the product. Therefore, we were surprised to observe product formation with very good yields [31]. This could be attributed to the numerous key changes compared to the original publication by Hosoya et al., such as the use of methanethiosulfonate instead of *p*‐toluenethiosulfonate, and Cu(OTf)_2_ instead of CuI. Although Xu et al. had already demonstrated that the use of Cu(OTf)_2_ improved the yield, they did not investigate this copper salt in combination with Cs_2_CO_3_. Another reason for the improved yields observed in our on‐resin approach may be the spatial separation of the thiosulfonates, which reduces side reactions such as disulfide formation during S‐alkynylation. This has previously been identified as a limitation of this chemistry.

**TABLE 2:** Optimization of on‐resin S‐alkynylation of **P1** with 1‐hexyne **A21**.

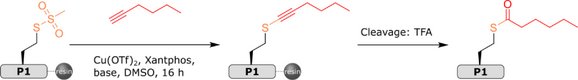				
Entry^a^	[Catalyst]	Base	Temperature	Yield^b^
1	50 mm	K_2_CO_3_	37°C	20%
2	200 mm	K_2_CO_3_	37°C	25%
3	50 mm	K_2_CO_3_	rt	70%
4	50 mm	Cs_2_CO_3_	37°C	80%
5	50 mm	Cs_2_CO_3_	rt	85%

^a^ 5 µmol of resin‐bound **P1T2** (**T2** refers to the methanethiosulfonate moiety) was used in the reaction with 500 mm 1‐hexyne in a solution of 0.5 mL DMSO. A one‐to‐one combination of Cu(OTf)_2_ and xanthphos was used as catalyst.

^b^ Yields are the ratio of the purity of the thioester peptide **P1A21** (**A** refers to the respective alkyne that was used in the S‐alkynylation) to the unmodified peptide **P1–OH**. Peptide purities were determined by RP‐HPLC.

### 2.3 Scope and Limitations of the S‐Alkynylation

**FIGURE 3:**
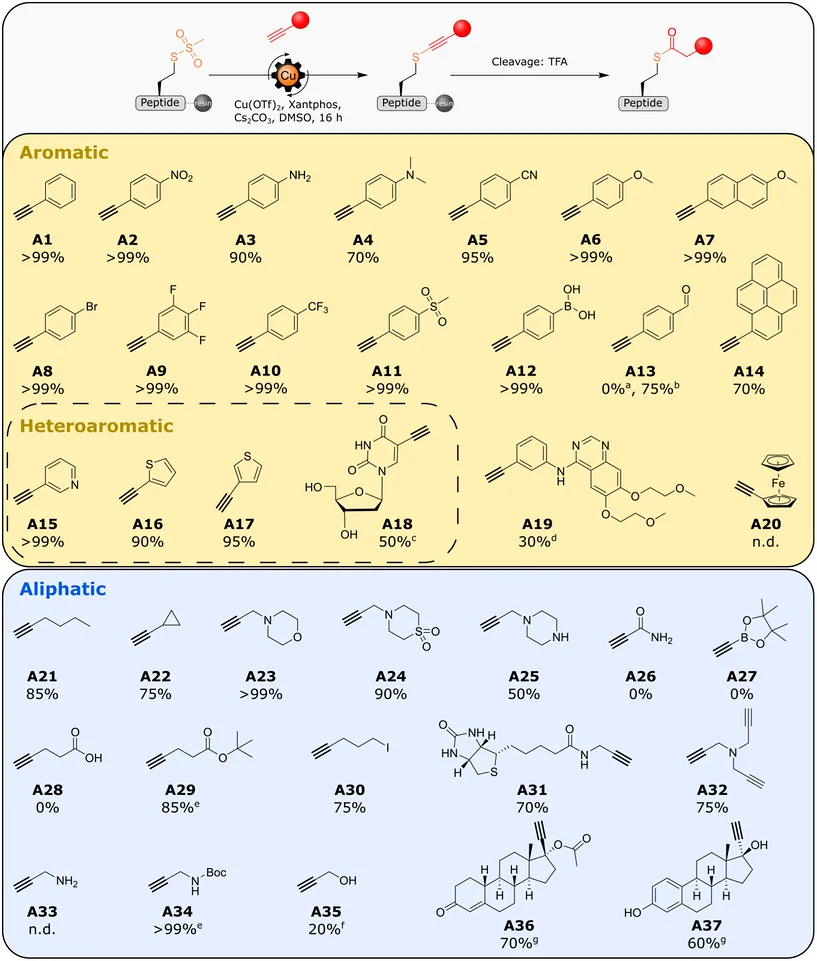
Scope of the S‐alkynylation of **P1**. Yields are defined as the ratio of the purity of the thioester peptide (excluding **A36** and **A37**) to that of the unmodified homoserine‐containing peptide **P1–OH**, as determined by RP‐HPLC. Further details are available in the Supporting Information. The conditions used are those listed in Table 1, entry 5, except for alkynes **A18** and **A19** (see below). ^a^S‐Alkynylation with **A13** produced Cannizzaro reaction products as peptide thioesters. ^b^ **A13** was converted to the corresponding imine via reaction with propylamine prior to S‐alkynylation. ^c^ **A18** was used at a concentration of 40 mm instead of 50 mm. ^d^ **A19** did not fully dissolve during the reaction. ^e^The protecting groups (*tert*‐butyl and Boc) are removed during acidic cleavage from the resin. ^f^ **A35** produced a mixture of the corresponding peptide thioester (20% yield) and thioacrylate (40% yield). ^g^The yield refers to the corresponding product after Meyer–Schuster rearrangement. n.d.: Not determined.

Having established suitable conditions for the S‐alkynylation, we investigated the scope of alkynes that could be used in this reaction (Figures 3 and S5–S88). We tested both aromatic and aliphatic alkynes bearing a variety of functional groups, including amines, amides, carboxylic acids, boronic acids, esters and nitriles. With a few exceptions, aromatic and heteroaromatic alkynes underwent S‐alkynylation with almost quantitative yields, while aliphatic alkynes produced the S‐alkynylated product with good to very good yields. On‐resin S‐alkynylation proved tolerant of a variety of functional groups, including boronic acids, which have been shown to react with thiosulfonates, albeit under different conditions [25]. However, carboxylic acids, such as those in **A28**, were not tolerated, nor were aldehydes, such as **A13**, which underwent a Cannizzaro reaction (Figures S31, S32). However, if protected as an ester (e.g. as a *tert*‐butyl ester, **A29**), carboxylic acids were accessible. The Cannizzaro reaction of **A13** was prevented by protecting the aldehyde through imine formation with propylamine prior to S‐alkynylation. The aldehyde was restored by hydrolysis during cleavage (Figure S33). Moderate yields were observed for more structurally complex alkynes. For example, 5‐ethynyl‐2'‐deoxyuridine **A18** reacted with a yield of 50%, but the HPLC trace showed a large amount of unreacted thiosulfonate, indicating that optimizing the reaction conditions could improve the yield. In addition, the reaction solution turned dark green instead of the usual light green, which indicates that the copper binds to the substrate in other ways, potentially explaining the reduced conversion. Another challenging alkyne that was tested was erlotinib (**A19**), which is known for its low solubility. Consequently, it did not fully dissolve during the S‐alkynylation. Nevertheless, we could still achieve a yield of 30%.

Aliphatic alkynes (**A21–A37**) reacted with very good yields in the case of the morpholine derivative **A22** and the thiomorpholine dioxide derivative **A23**. The piperazine‐containing alkyne **A25** produced the desired thioester **P1A25**, albeit with a lower yield of 50%. However, we demonstrated that the thioalkyne could undergo further conversion in an amide coupling with 5(6)‐carboxy‐TAMRA on the resin, producing the TAMRA‐labeled product with a yield of 95% (Figures S55, S56). This suggests that, unlike other substrates examined in our study, thioester **P1A25** is highly susceptible to hydrolysis, which may be due to the nucleophilic secondary amino group of the piperazine ring. Alkynes **A26**, **A27** and **A28** (*vide supra*) did not react. 5‐Iodopentyne **A30** was coupled with a yield of 75% without any elimination being observed. Biotin alkyne **A31** was introduced with a yield of 70%. Interestingly, on‐resin S‐alkynylation with substrates containing more than one alkynyl group, such as tripropargylamine (**A32**), proceeded without observable cross‐linking. The reaction with propargylamine (**A33**) resulted in complete conversion; however, only traces of the respective thioester were observed and instead a by‐product was formed that was not further investigated. Using Boc‐protected propargylamine (**A34**) prevented this side reaction, enabling us to observe the corresponding amine‐containing thioester in high yields. Propargyl alcohol (**A35**), similar to propargylamine, proved to be a challenging substrate: the thiosulfonate was completely converted, but the crude peptide contained a mixture of thioester, thioacrylate and other products (Figures S68–S70). The formation of the thioacrylate was thoroughly investigated using NMR spectroscopy with the modified tripeptide **P5A35** (Figures S71–S79). It presumably forms during acidic cleavage from the resin via a Meyer–Schuster rearrangement. This hypothesis is supported by the fact that further conversion of propargyl alcohol‐modified peptides in an iridium‐catalyzed thioalkyne‐azide click reaction (IrAAC) to corresponding triazoles proceeds with excellent yield, as demonstrated for **P6A35Az14** (*vide infra*). The hormones norethisterone acetate (**A36**) and ethinyl estradiol (**A37**) also contain a propargyl alcohol motif, which may influence the reaction. As in **A35**, Meyer‐Schuster rearrangement occurs (Figures S80, S81), which was verified by NMR analysis of the tripeptide **P5A37** (Figures S82–S88).

**FIGURE 4:**
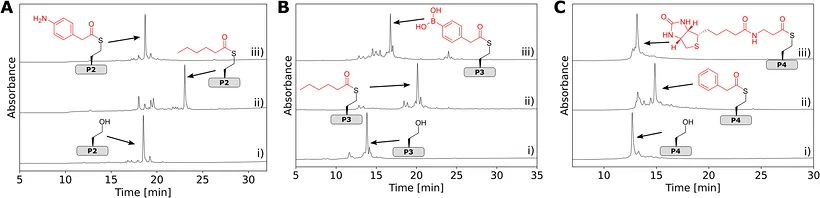
HPLC traces of crude, unmodified peptides and peptide thioesters obtained via on‐resin S‐alkynylation of methanethiosulfonate‐containing peptides, followed by cleavage with TFA. (A‐i) **P2–OH**, (A‐ii) **P2A21**, (A‐iii) **P2A3**; (B‐i) **P3–OH**, (B‐ii) **P3A21**, (B‐iii) **P3A12**; (C‐i) **P4–OH**, (C‐ii) **P4A1**, (C‐iii) **P4A31**.

To demonstrate that S‐alkynylation also works with more complex peptides and on different resins, we performed this reaction on peptides **P2**, a SARS‐CoV‐2 ORF1a protein peptide epitope produced on Wang resin, **P3**, a histone mutant H3.3_K27M peptide epitope containing methionine and **P4**, the 34‐amino‐acid‐long human Pin1 WW domain (Figure 4) [53, 54, 55]. As with **P1**, clean conversion to the S‐alkynylation products and, after acidic cleavage, the respective thioesters was observed when using aliphatic and aromatic alkynes of varying structural complexity. It should be noted that, in the case of **P3** and **P4**, the respective peptide thiosulfonates could not be isolated; however, the S‐alkynylation proceeded well even when the long peptide **P4** was modified with biotin **A31**, as can be seen from the HPLC traces (Figures S89–S97).

### 2.4 Triazole Synthesis from Peptide Thioalkynes

On‐resin S‐alkynylation proved to be a valuable tool for preparing thioalkynylated peptides with a level of complexity yet unattained for this type of reaction. Since thioalkynes are particularly well‐known for their versatile chemistry [32], we were interested in further modifying this group to open up new avenues for sequential modification strategies in the diversity‐oriented synthesis of complex, modified peptides. The ruthenium‐ or iridium‐catalyzed thioalkyne‐azide click reaction (IrAAC) in particular had piqued our interest, as it enables the regioselective formation of 1,5‐triazoles with internal alkynes due to the π‐donating alkylthio substituent. Furthermore, it is compatible with multiple solvents and exhibits high selectivity for aliphatic azides [33, 56, 57]. First, we tested the ruthenium‐catalyzed thioalkyne‐azide click reaction by equilibrating peptide resins of phenylacetylene modified **P1A1** and 1‐hexyne modified **P1A21** and **P2A21** with 250 mm 6‐azidohexanoic acid (**Az1**) and the Cp*RuCl(PPh_3_)_2_ catalyst at room temperature for 16 h. The triazole was obtained in very good yields in both cases (Figures S98–S101). However, triazole formation in the 1‐hexyne‐modified peptides **P1A21** and **P2A21** (Figures S100–S102) also led to the formation of another isomer, albeit in low yields. To circumvent this problem, we opted for IrAAC, using the [Ir(cod)Cl]_2_ catalyst, since it is reported to catalyze the reaction with improved regioselectivity compared to ruthenium catalysts [33]. Indeed, the iridium‐catalyst led to the complete regioselective conversion of the thioalkyne to the 1,5‐triazole (Table 3, entries 2–4, Figures S100, S101). Furthermore, changing the solvent to DMF ensured the solubilization of more polar azides. We were also able to reduce the reaction time to 2 h and decrease the amounts of catalyst and azide required for complete conversion to 10 and 100 mm, respectively. The regioselectivity of the IrAAC was confirmed by NMR spectroscopic analysis of modified tripeptide **P5** (Table 1, Figures S103–S110). S‐alkynylation using **A1**, followed by IrAAC using **Az1**, resulted in the formation of the 1,5‐triazole isomer, as revealed by ^1^H,^13^C‐HMBC‐NMR spectrum (Figure S109).

**TABLE 3:** Optimization of on‐resin metal‐catalyzed thioalkyne‐azide click reaction in **P1A21**.

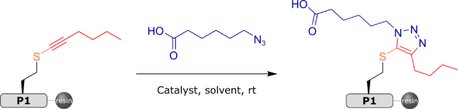					
Entry^a^	Catalyst	Solvent	Azide	Time	Yield^b^
1	40 mm Cp*RuCl(PPh_3_)_2_	Toluene	250 mm **Az1**	16 h	50%
2	40 mm [Ir(cod)Cl]_2_	Toluene	250 mm **Az1**	2 h	90%
3	5 mm [Ir(cod)Cl]_2_	DMF	50 mm **Az1**	2 h	90%
4	10 mm [Ir(cod)Cl]_2_	DMF	100 mm **Az1**	2 h	95%

^a^ 5 µmol of resin‐bound **P1A21** was used in the reaction in 0.5 mL solution of the reaction mixture at room temperature.

^b^ Yields are defined as the ratio of the purity of the 1,5‐triazole peptide to the unmodified peptide **P1–OH**. Peptide purities were determined by RP‐HPLC.

### 2.5 Scope and Limitations of On‐Resin IrAAC

**FIGURE 5:**
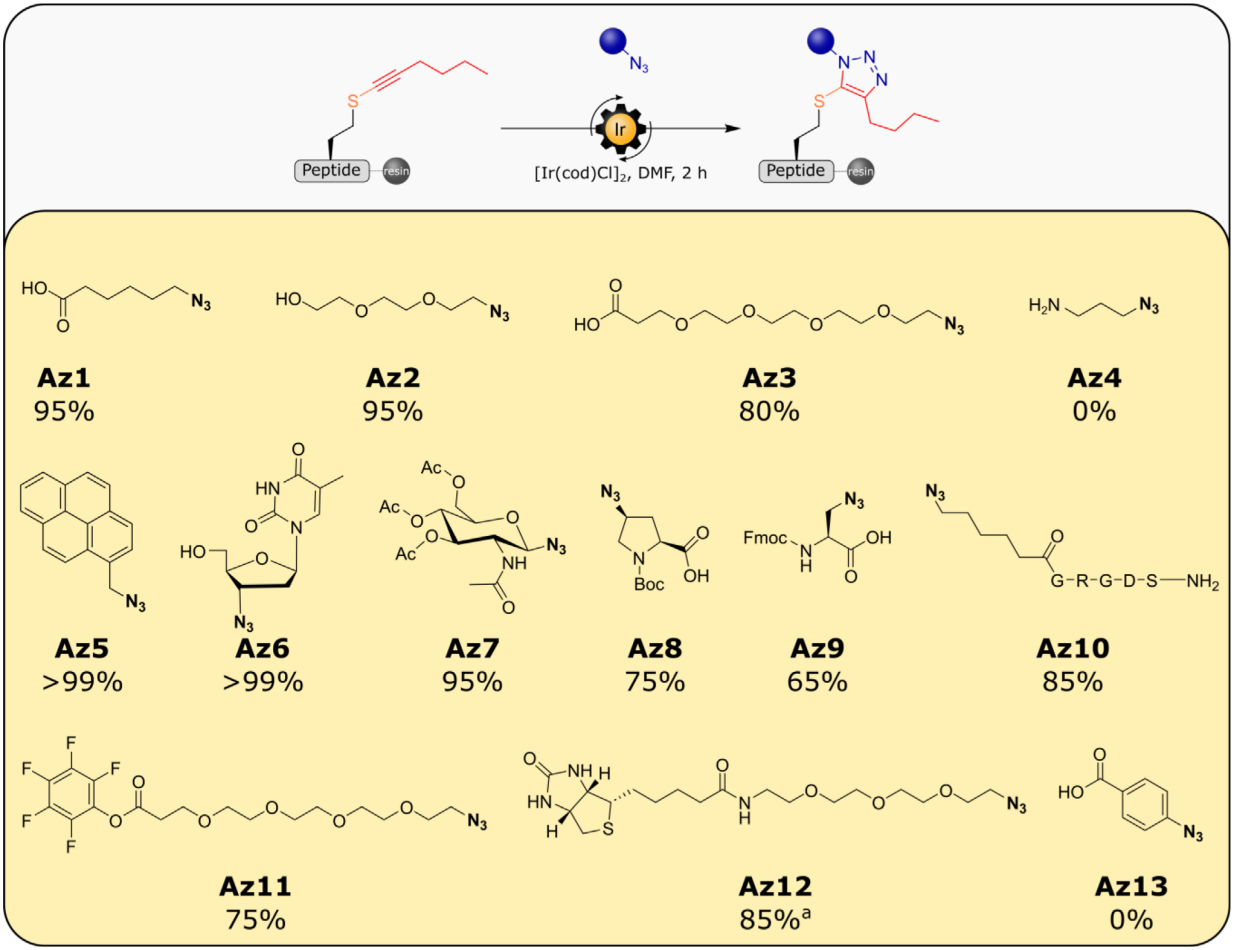
Scope of azides used in the IrAAC with 1‐hexyne modified peptide **P1A21** and conditions of Table 2, entry 5. The yields refer to the ratio of the purity of the 1,5‐triazole‐containing peptide compared to the purity of the unmodified homoserine‐containing peptide **P1–OH** determined by RP‐HPLC. Further details are available in the Supporting Information. ^a^ After reduction with TMSBr and ethane‐1,2‐dithiol.

In order to explore the scope and limitations of the IrAAC for on‐resin peptide diversification, we tested the reaction using hexyne‐modified peptide **P1A21** and 13 different azides (Figures 5; Figures S111–S131). PEG‐containing azides **Az2** and **Az3** produced the respective cycloaddition products in good yields. However, azidopropylamine **Az4** did not react, potentially due to the amino group coordinating to iridium. The polarity of the azide is not decisive for the IrAAC, as reactions with the hydrophobic azide 1‐(azido)‐methylpyrene (**Az5**), the polar, highly functionalized azides zidovudine (**Az6**) and β‐d‐glucopyranosyl azide (**Az7**), all proceeded with very good yields. We further verified compatibility with biomolecules by using the amino acids N‐Boc‐azido‐proline (**Az8**) and Fmoc‐β‐azido‐Ala‐OH (**Az9**), as well as the azide‐containing cell‐adhesive RGD peptide **Az10**, the latter being completely unprotected [58]. Even azide **Az11** containing a reactive pentafluorophenyl ester can be incorporated into peptides without being compromised by the applied reaction conditions [59, 60]. For the modification with the biotin‐containing azide **Az12**, we observed oxidation after cleavage of the peptide from the resin. Therefore, we applied a sulfoxide reduction protocol consisting of a mixture of TFA, trimethylsilyl bromide (TMSBr) and ethane‐1,2‐dithiol, which resulted in an improved yield of 85% (Figure S130; further details are available in the Chapter 3.3.4, Supporting Information) [61]. Lastly, we tested the reactivity of the aromatic azide **Az13**, which does not react, as this reaction is described in the literature as being selective for aliphatic azides [62].

**FIGURE 6:**
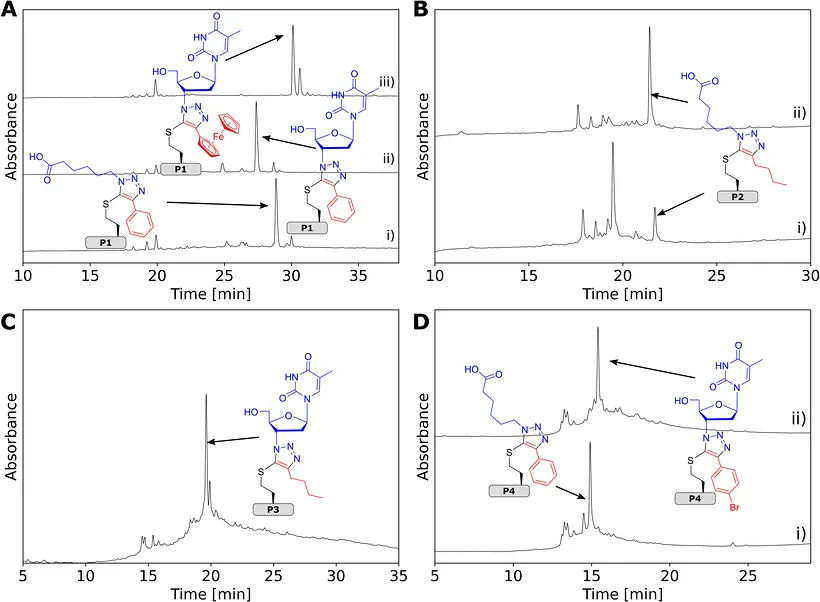
HPLC traces of crude triazole peptides obtained by on‐resin S‐alkynylation and IrAAC. (A‐i) **P1A1Az1**, (A‐ii) **P1A1Az6**, (A‐iii) **P1A20Az6**. (B‐i) **P2A21Az1** prepared with standard conditions. (B‐ii) **P2A21Az1** prepared with adjusted conditions. (C) **P3A21Az6**. (D‐i) **P4A1Az1**. (D‐ii) **P4A8Az6**.

We also tested other S‐alkyne peptides in IrAAC, such as the phenylacetylene‐containing peptide **P1A1** with zidovudine **Az6**, which provides the cycloaddition product with complete conversion in five steps after initial homoserine deprotection (Figures 6; Figure S132). We were interested in preparing triazoles with ferrocene‐containing thioalkyne peptides since modification with ethynylferrocene **A20** proceeded on the resin; however, the corresponding thioester could not be isolated. Ferrocene can be used as an electrochemical sensor in peptides, prompting us to investigate the incorporation of ethynylferrocene further, using our sequential dual‐modification strategy. Interestingly, the reaction sequence proceeded with complete conversion of **P1–OH** to the ferrocene‐containing triazole peptide. In this case, however, HPLC revealed the formation of the two possible isomers of the cycloaddition reaction (Figures 6A‐iii and S133). NMR analysis of the ferrocene‐modified peptide **P5A20Az1** (**Az** refers to the respective azide that was used to produce the triazole‐containing peptide) revealed that the main product is the expected 1,5‐triazole, with 1,4‐triazole as byproduct, which may be formed due to a steric or electronic effect of the ferrocene group (Figures S134–S148).

Finally, we investigated the broad applicability of the IrAAC reaction using peptides **P2** to **P4** (Figures 6, S102, S149–S151). For **P2**, the reaction did not proceed to full conversion under standard conditions, but could be completed by increasing the amounts of catalyst and azide, as well as extending the reaction time. **P3**, which contained a methionine residue, could be converted to the triazole **P3A21Az6**, albeit with lower purity than the unmodified peptide under standard conditions. However, applying the adjusted conditions used for **P2**, as well as the sulfoxide reduction protocol mentioned above, significantly improved the purity of the final crude product (Figures 6C and S149). Conversely, the WW domain **P4** yielded the triazole as the main product under standard conditions, demonstrating that the complexity and size of the peptide are not decisive factors in the outcome of the IrAAC reaction.

### 2.6 Structure–Activity Relationships of Dengue Protease Inhibitors

**FIGURE 7:**
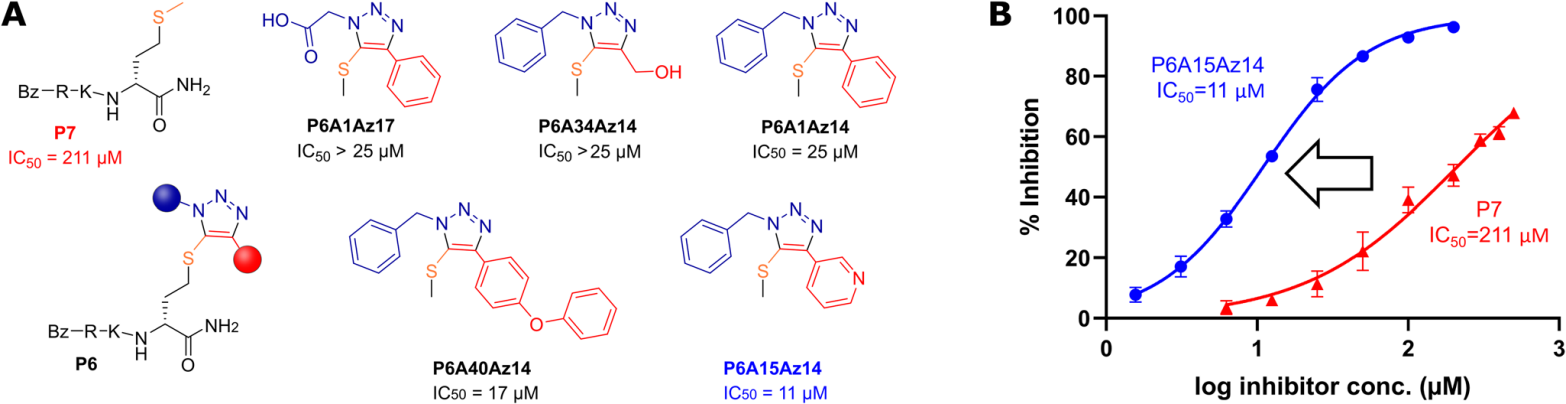
SAR exploration at the C‐terminal residue of DENVpro inhibitors. (A) Structures of the lead and reference compound (**P7**), general structure of the obtained inhibitors, and selected variants of **P6**. (B) Dose–response curves of **P7** versus **P6A15Az14** in a biochemical DENVpro activity assay, indicating a 20‐fold increase in activity.

We applied our on‐resin dual‐modification approach of peptide thiosulfonates to explore structure‐activity relationships of peptide inhibitors of the dengue virus protease (DENVpro), a well‐recognized target for the development of novel antivirals [63]. We used the phenylglycine (d‐Phg) derivatives first described by Behnam et al. [64] as lead structures, with the intention to increase structural variability at the position of the d‐Phg, where previous modifications had delivered potent inhibitors and indicated steric tolerance towards larger residues. The incorporation of d‐Phg derivatives resulted in potent inhibitors, but their pronounced hydrophobicity is associated with low aqueous solubility, and care must be taken to prevent epimerization during SPPS [65]. These limitations prompted us to search for alternative residues. First, replacement of the d‐Phg in the previous lead compound MB‐8 with a d‐Methionine (d‐Met) residue resulted in an expected decrease in potency (Figure 7). Based on the d‐Met analog **P7** as lead and reference compound for the present series, a library of 17 triazole analogs (Figures S152–S169) was synthesized and characterized in terms of their inhibitory potential against DENVpro (see Supporting Information). We observed interesting structure–activity relationships, with compound **P6A15Az14** demonstrating an IC_50_ of about 10 µM, corresponding to a 20‐fold increase in comparison to the lead compound (Figure 7). This was accompanied by a prominent and desirable increase of aqueous solubility in comparison to d‐Phg analogs like MB‐53, which can likely be attributed to the triazole moiety. Not unexpected for DENVpro as a target, hydrophilic functions in the C‐terminal side chain (**P6A1Az17**, **P6A34Az14**) led to a loss in activity, whereas hydrophobic elements were generally favorable. From a drug discovery perspective, the tolerance of the synthetic methodology towards functional groups and heterocycles such as the pyridine ring in **P6A15Az14** is an attractive feature that should encourage its further application.

## 3 Conclusion

The dual‐modification approach to resin‐bound peptides presented herein, comprising S‐alkynylation and IrAAC, provides reliable access to complex non‐canonical peptide modifications. We demonstrated that the first step of our dual modification, the copper‐catalyzed S‐alkynylation of peptide thiosulfonates, is compatible with a variety of aromatic and aliphatic alkynes, exhibiting excellent functional group tolerance. Subsequent on‐resin IrAAC then selectively delivered 1,5‐triazoles with various benzylic and aliphatic azides. We have demonstrated that this method can be applied to peptide‐alkyne‐azide combinations exhibiting a high level of complexity. Moreover, biologically useful tags, such as pyrene fluorescent tags, PEG, biotin, and an RGD peptide, have been introduced into peptides in very good yields using this method. Furthermore, we demonstrated that the method can be used to rapidly evaluate structure–activity relationships and improve the potency of peptide inhibitors of DENV protease. Please note that the peptide thioalkynes produced on the resin are converted into the corresponding peptide thioesters during acidic cleavage. Thioesters are known to be reactive handles in both nature and chemical biology. The on‐resin S‐alkynylation can therefore also be regarded as a method to produce SPPS‐compatible masked thioesters.

While some reports describe the copper‐catalyzed S‐alkynylation of fully protected di‐ and tripeptides in solution, no resin‐based approach has yet been presented. However, transferring this chemistry to the solid phase has unleashed its full potential: long peptide sequences can now be modified, and undesirable side reactions, such as the formation of disulfides, can be suppressed. In most cases, the individual reaction steps proceed with the complete conversion of the starting material and very good yields, since an excess of reagents can be used. Azide‐alkyne click reactions are frequently employed in peptide diversification, for example, the synthesis of compound libraries in drug discovery or for the introduction of fluorescent labels and other analytical tags. Until now, however, one of the components—either azide or alkyne—had to be present in the peptide [13, 66, 67, 68]. Our dual‐modification approach allows alkyne and azide to be introduced sequentially and efficiently, starting from a conventional peptide sequence synthesized by SPPS. This makes it a promising new addition to the chemical toolkit for diversifying peptides and discovering drugs.

Finally, we would like to point out that the dual‐modification approach involves a sequence of reactions comprising not two, but four late‐stage modifications: on‐resin homoserine iodination, nucleophilic substitution with sodium methanethiosulfonate, copper‐catalyzed S‐alkynylation and IrAAC. This demonstrates the power and potential of on‐resin late‐stage peptide modifications in general. On‐resin iodination‐substitution alone enables a variety of possible non‐canonical modifications and serves as a hub for further diversification of peptides in this case. The synthetic potential of thiosulfonates and thioalkynes could be exploited further beyond the scope of this study, a prospect that will be investigated in future research.

## Author Contributions

**Marius Werner**: conceptualization, investigation, methodology, writing – original draft. **Agnes Bergmann**: investigation, methodology, writing – review and editing. **Chenxi Liu**: methodology, investigation, writing – review and editing. **Mira Behnam**: investigation, methodology, writing – review and editing. **Christian Klein**: conceptualization, writing – original draft, supervision, resources. **Franziska Thomas**: conceptualization, writing – original draft, supervision, resources.

## Conflicts of Interest

The authors declare no conflicts of interest.

## Supporting information



The authors have cited additional references within the Supporting Information [69, 70, 71, 72].
**Supporting File**: anie73438‐sup‐0001‐SuppMat.pdf.

## Data Availability

All data are available in the Supporting Information. Additionally, the data are published in the public data respoitory HeiData of Heidelberg University, doi.org/10.11588/DATA/KLPKJD.
