# Supplementary material for: Peptide Thioester and Triazole Derivatives Through On‐Resin Dual‐Modification of Peptide Thiosulfonates
Source: Angew Chem Int Ed Engl. 2026 Jul 13;65(38):e8315692. doi: 10.1002/anie.8315692 (PMC13573147; doi:10.1002/anie.8315692)
Supplement: Supplementary file 1 [file ANIE-65-e8315692-s001.pdf]

Table of Contents

1 Supplementary figures S1

2 Inhibition of dengue virus protease S161

3 Experimental procedures S163

3.1 Reagents and solvents S163

3.2 Software for data analysis and visualization S163

3.3 Peptide synthesis, functionalization and purification S163

3.4 NMR spectroscopy S169

3.5 Thin layer and column chromatography S169

4 Organic synthesis S170

4.1 Fmoc-Hse(Dmt)-OH S170

4.2 Fmoc-D-Hse-OH S170

4.3 Fmoc-D-Hse(Dmt)-OH S171

5 References S173

1 Supplementary figures

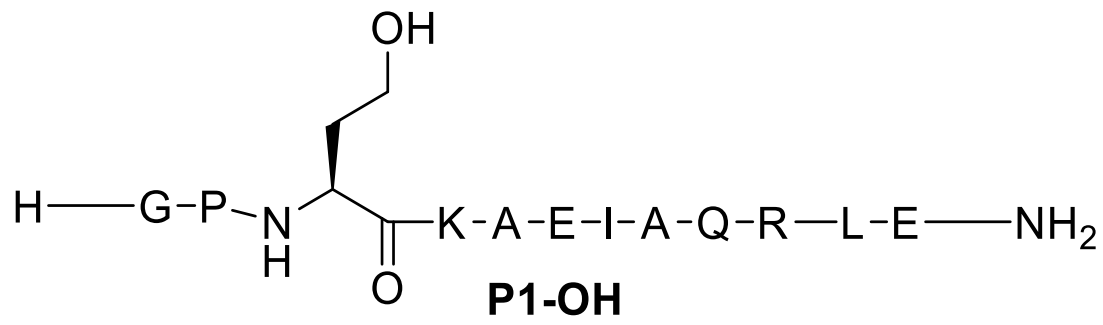

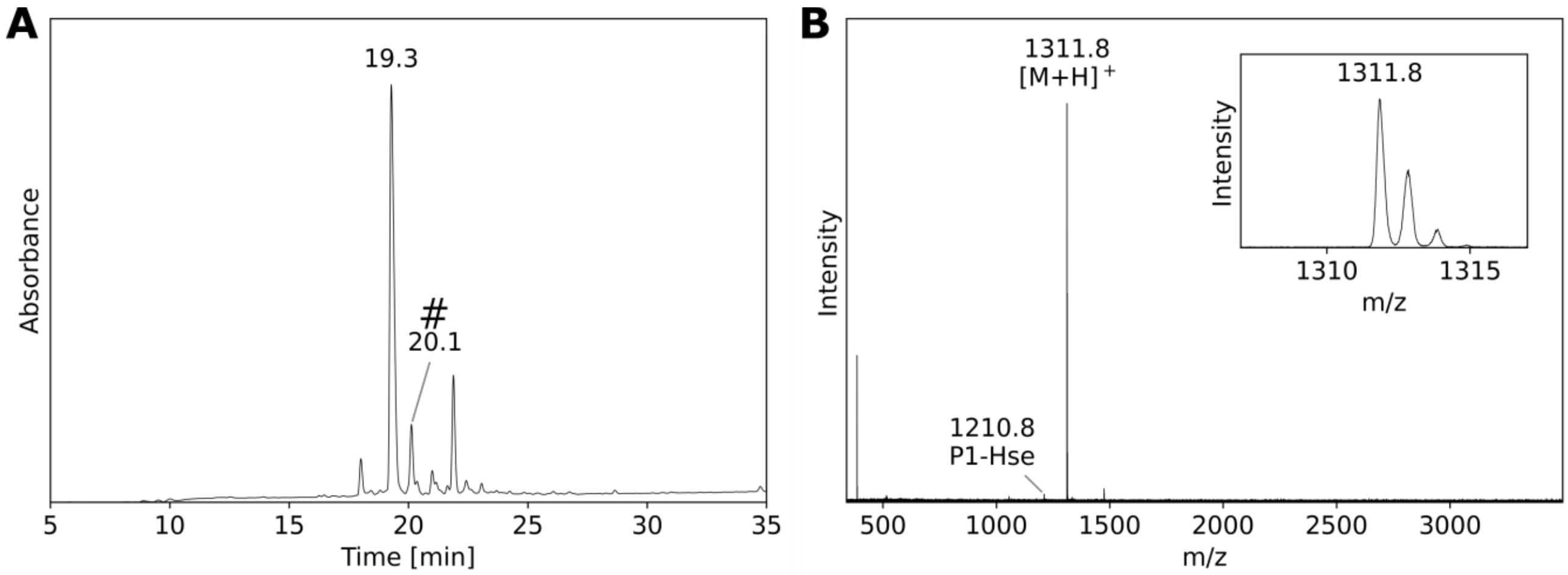

Figure S1: **P1-OH** A) Analytical HPLC (220 nm) of crude peptide. HPLC Gradients are listed in Section 2.3.5 Table S3. # Peptide without homoserine (P1-Hse) was observed as byproduct in varying amounts in all batches. B) MALDI-TOF MS of crude peptide. Calculated mass: $[M+H]^+$ 1311.7, $[P1\text{-}Hse+H]^+$ 1210.7.

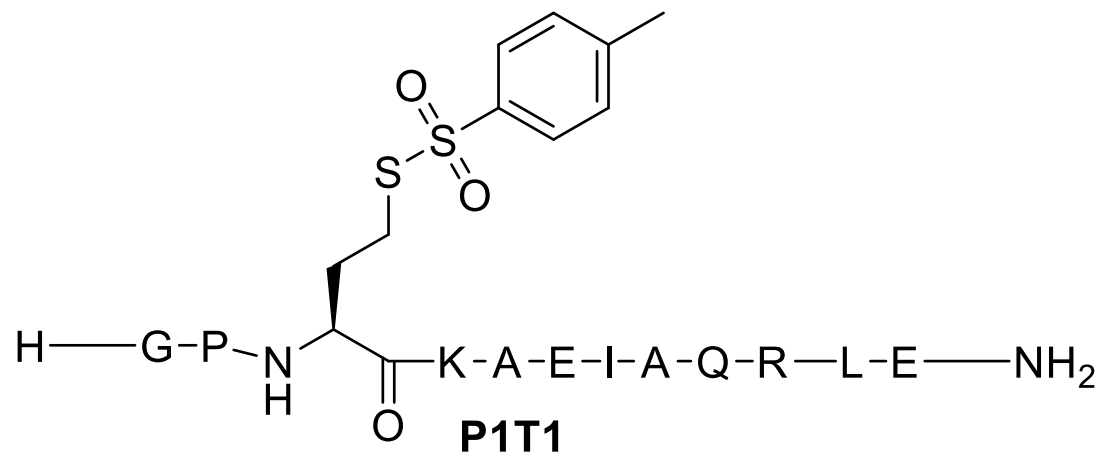

P1T1

Chemical Formula: $C_{63}H_{104}N_{18}O_{19}S_2$
Exact Mass: 1480.72
Molecular Weight: 1481.75

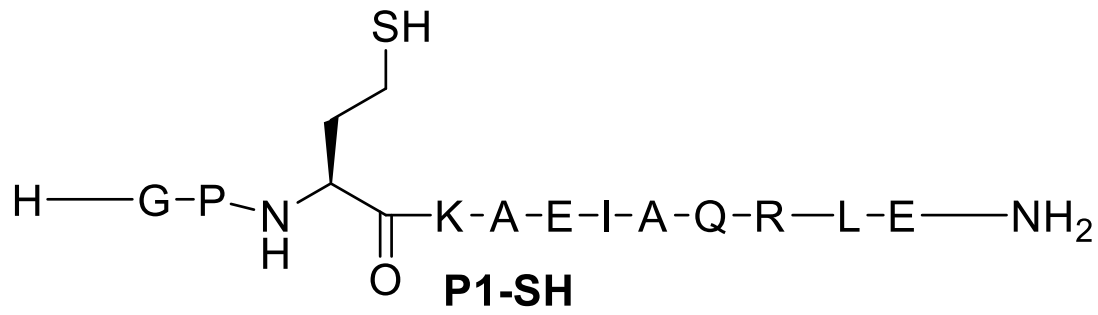

P1-SH

Chemical Formula: $C_{56}H_{98}N_{18}O_{17}S$
Exact Mass: 1326.71
Molecular Weight: 1327.57

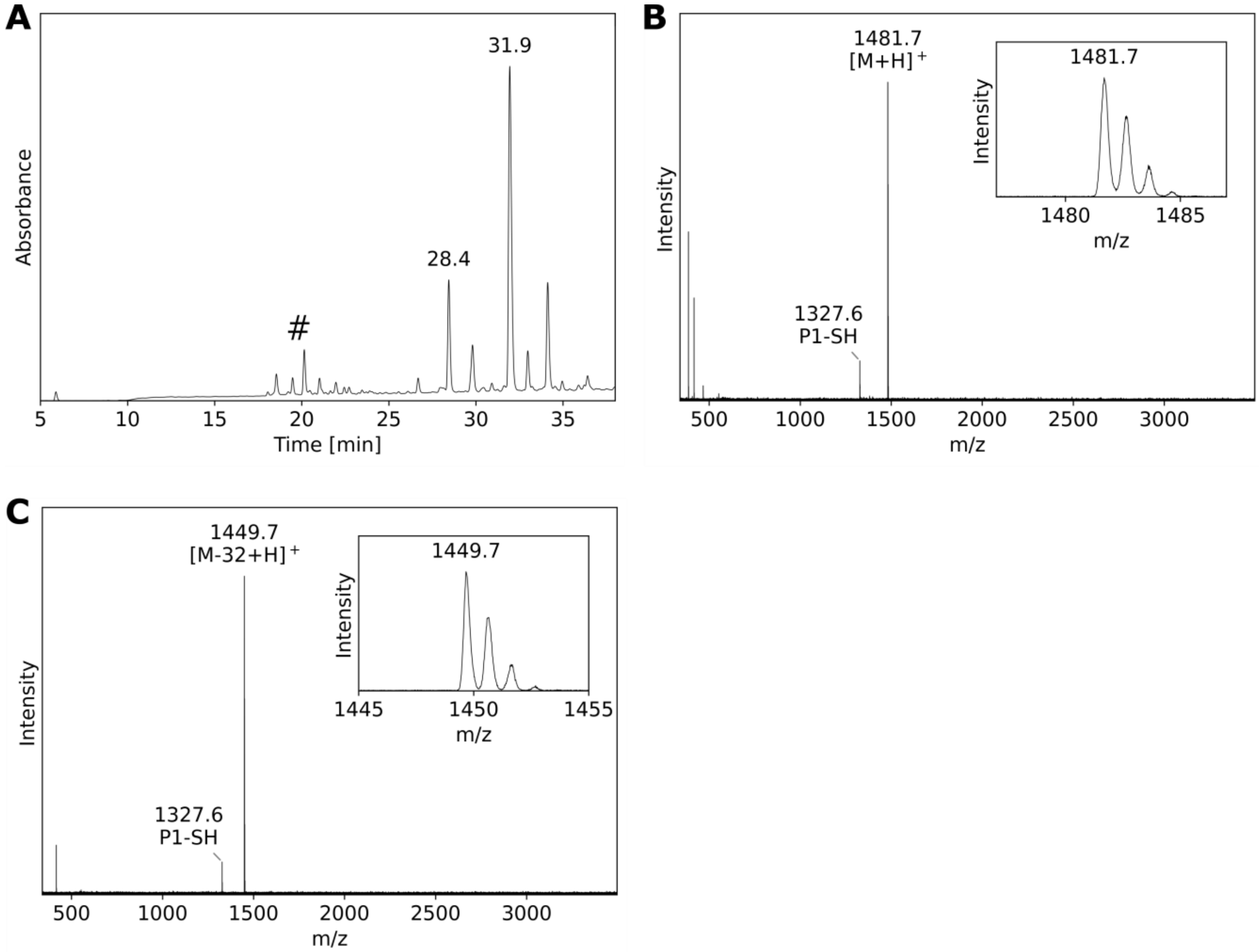

Figure S2: **P1T1** A) Analytical HPLC (220 nm) of crude peptide. HPLC Gradients are listed in Section 2.3.5 Table S3. # Peptide without homoserine. B) MALDI-TOF MS of purified peptide **P1T1** with a retention time of 28.4 min. C) MALDI-TOF MS of purified main peptide species with a retention time of 31.9 min. Calculated mass: $[M+H]^+$ 1481.7, $[M-32+H]^+$ 1449.7, $[P1\text{-}SH+H]^+$ 1327.7.

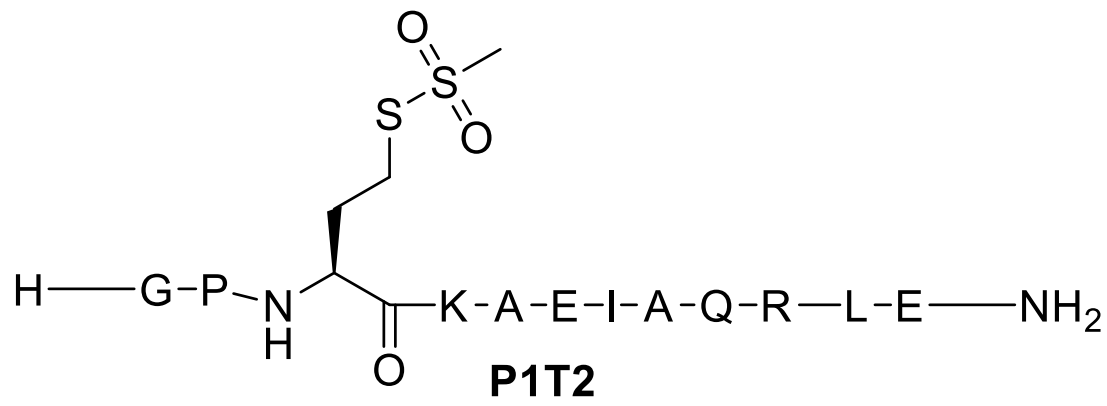

P1T2

Chemical Formula: $C_{57}H_{100}N_{18}O_{19}S_2$

Exact Mass: 1404.69

Molecular Weight: 1405.65

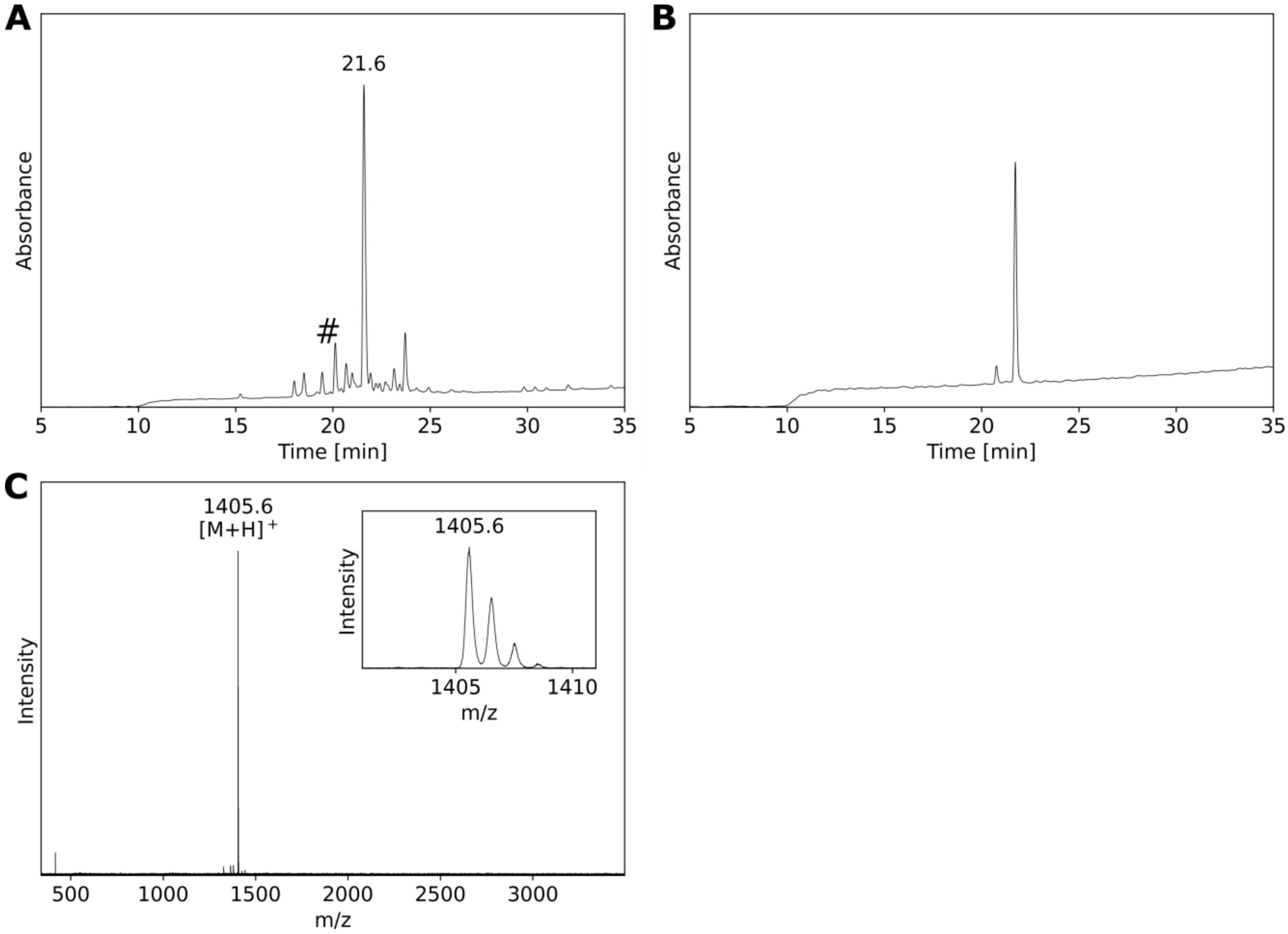

Figure S3: **P1T2** A) Analytical HPLC (220 nm) of crude peptide. HPLC Gradients are listed in Section 2.3.5 Table S3. # Peptide without homoserine. B) MALDI-TOF MS of pure peptide. Calculated mass: $[M+H]^+$ 1405.7.

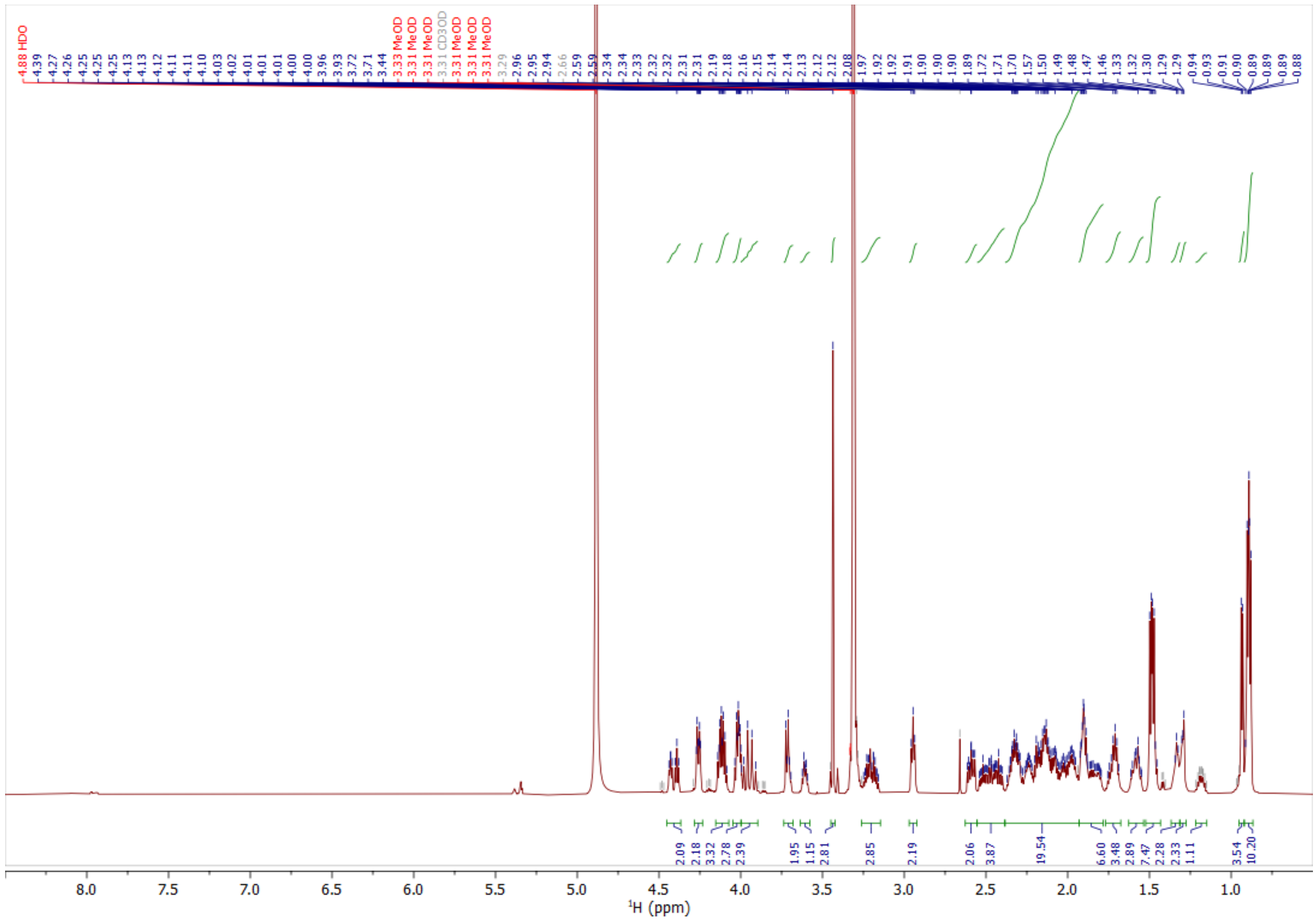

Figure S4: ^{1}H-NMR (700 MHz, MeOH-d_4, 295 K) of **P1T2**. Hydrogen-deuterium exchange was performed prior to the measurement.

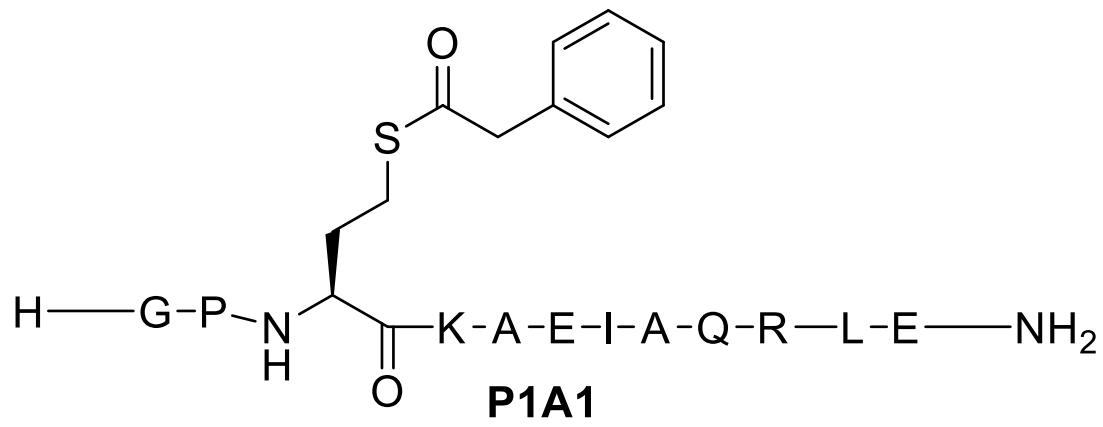

P1A1

Chemical Formula: $C_{64}H_{104}N_{18}O_{18}S$

Exact Mass: 1444.75

Molecular Weight: 1445.70

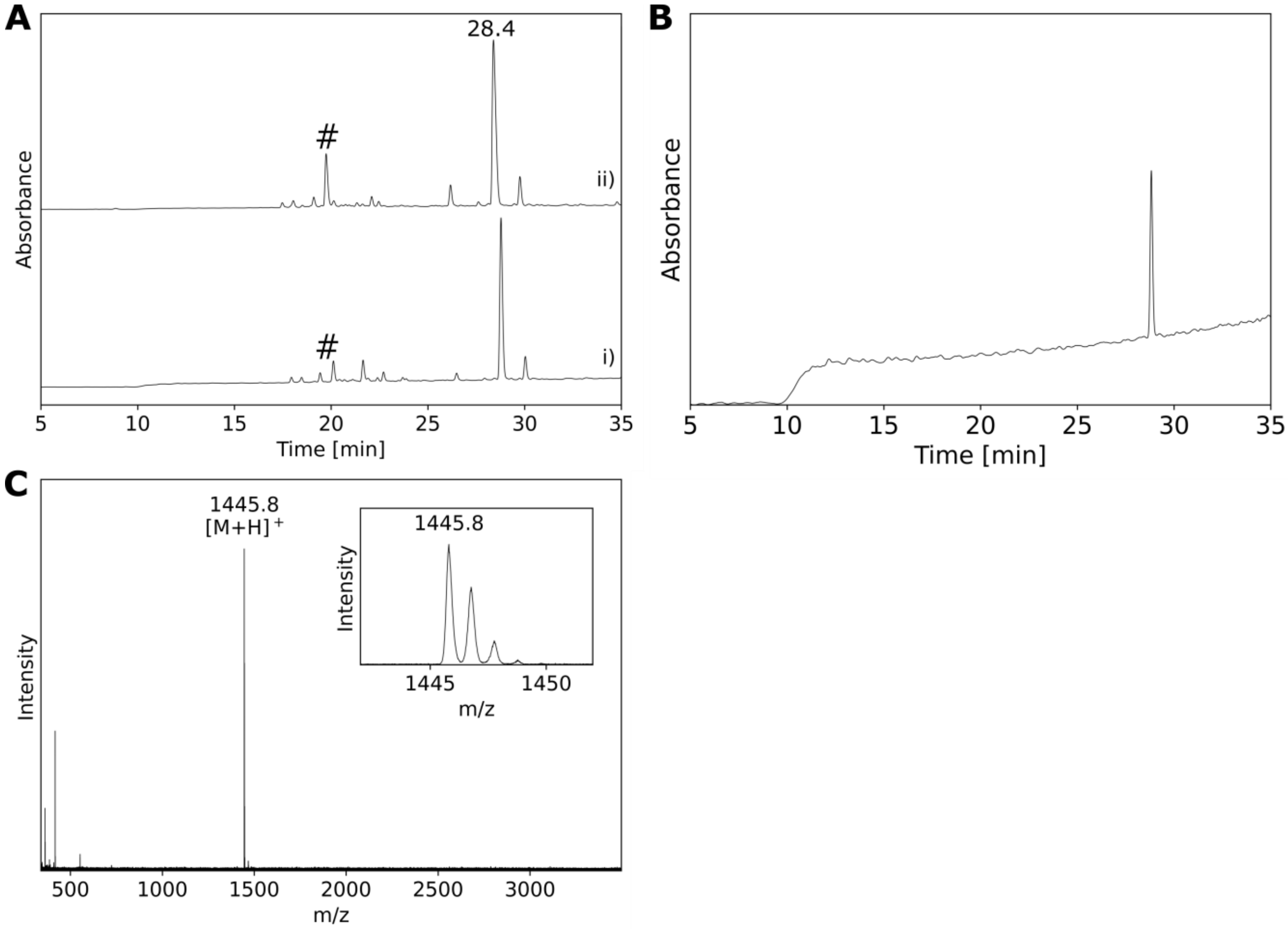

Figure S5: **P1A1** A) Analytical HPLC (220 nm) of crude peptide (i) prepared with conditions from Entry 1 in Table 2, (ii) prepared with conditions from Entry 5 in Table 2 . HPLC Gradients are listed in Section 2.3.5 Table S3. # Peptide without homoserine. B) Analytical HPLC (220 nm) of pure peptide. C) MALDI-TOF MS of pure peptide. Calculated mass: $[M+H]^+$ 1445.8.

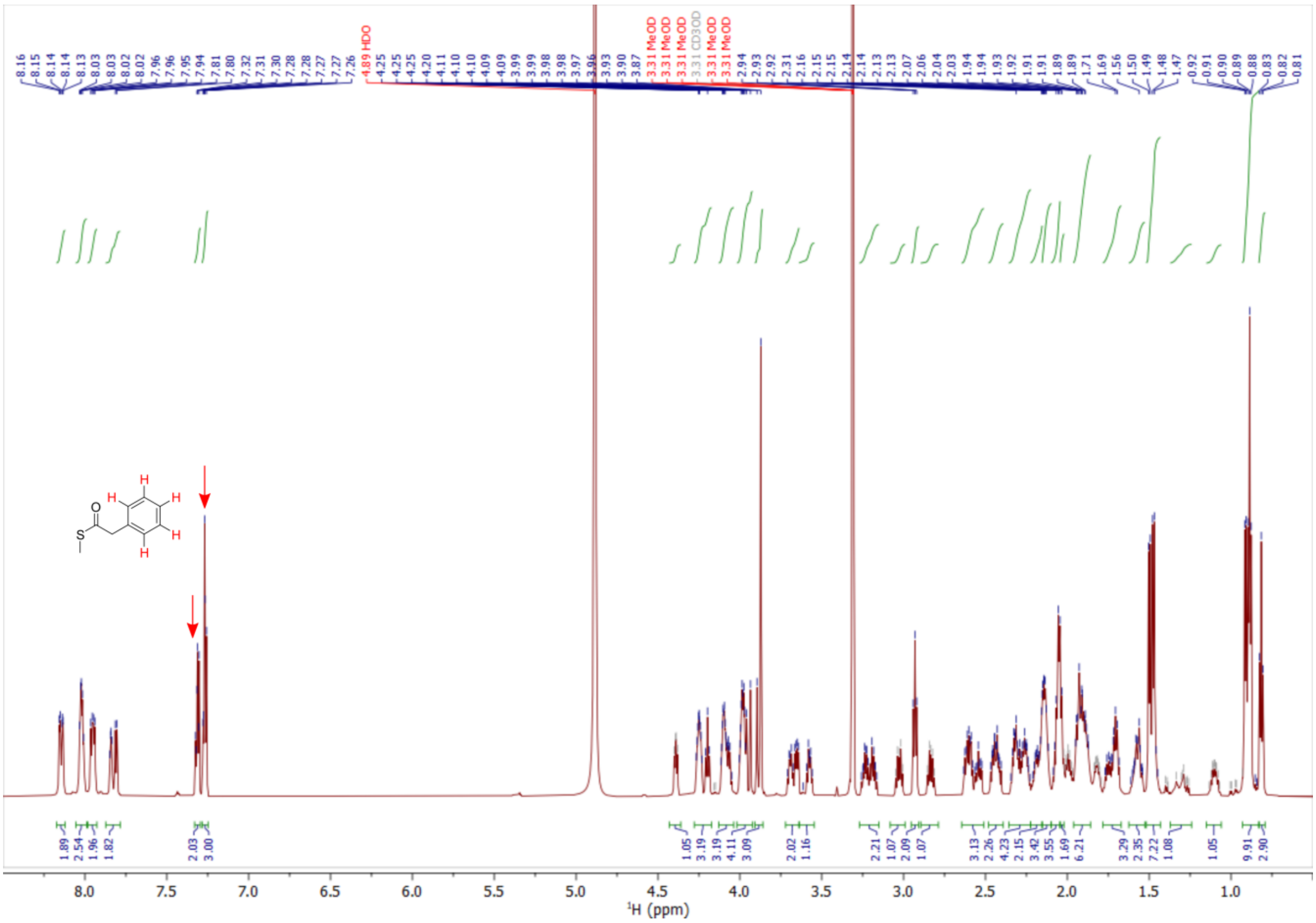

Figure S6: ^{1}H-NMR (700 MHz, MeOH-d_4, 295 K) of **P1A1**.

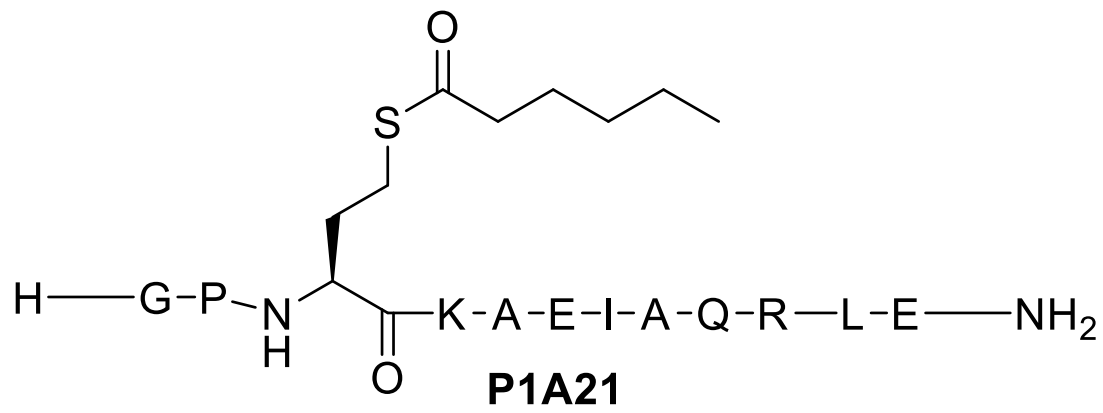

P1A21

Chemical Formula: $C_{62}H_{108}N_{18}O_{18}S$

Exact Mass: 1424.78

Molecular Weight: 1425.71

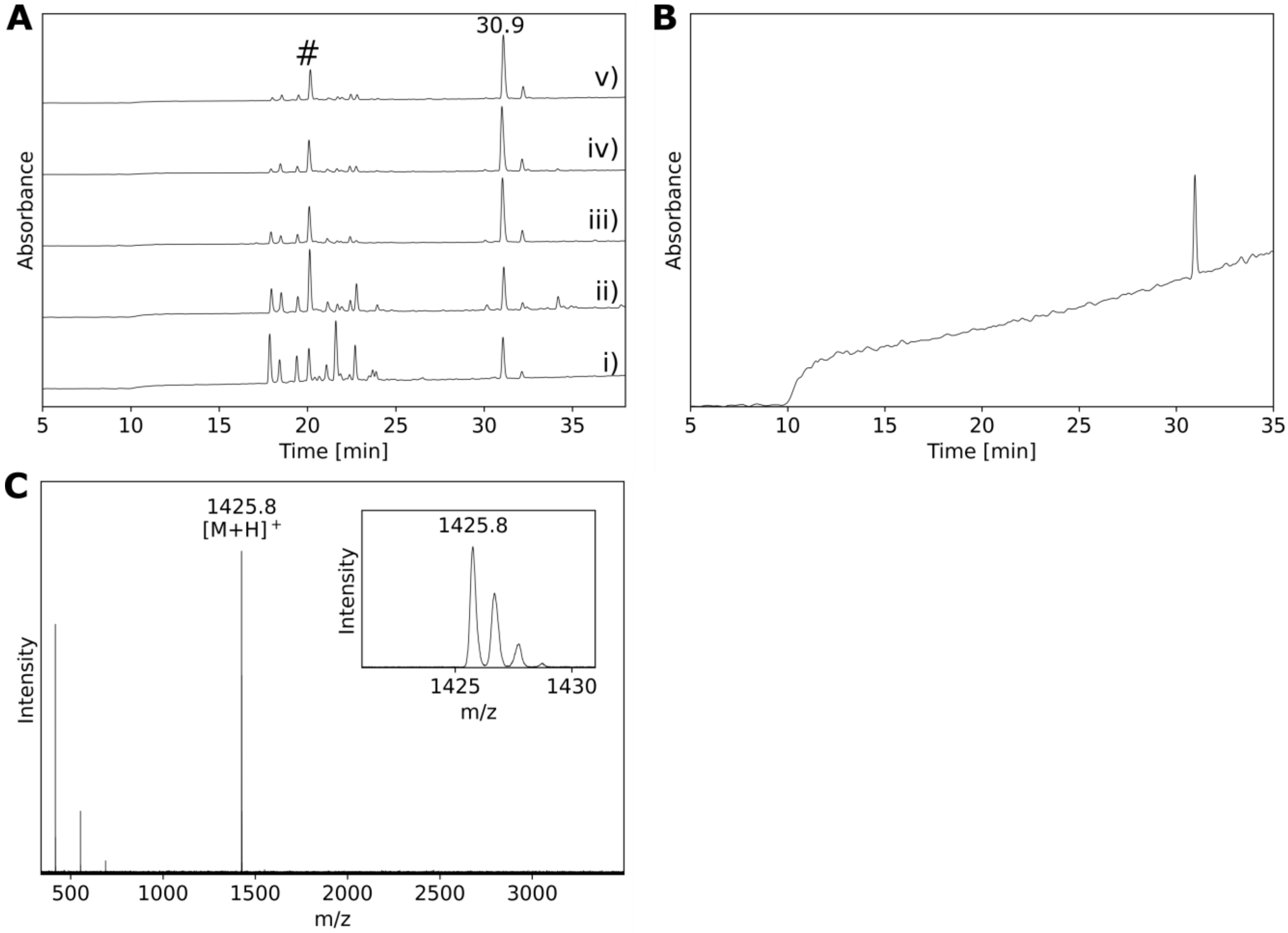

Figure S7: **P1A21** A) Analytical HPLC (220 nm) of crude peptides from Table 2 (i) Entry 1, (ii) Entry 2, (iii) Entry 3, (iv) Entry 4 and (v) Entry 5. HPLC Gradients are listed in Section 2.3.5 Table S3. # Peptide without homoserine. B) Analytical HPLC (220 nm) of pure peptide. C) MALDI-TOF MS of pure peptide. Calculated mass: $[M+H]^+$ 1425.8.

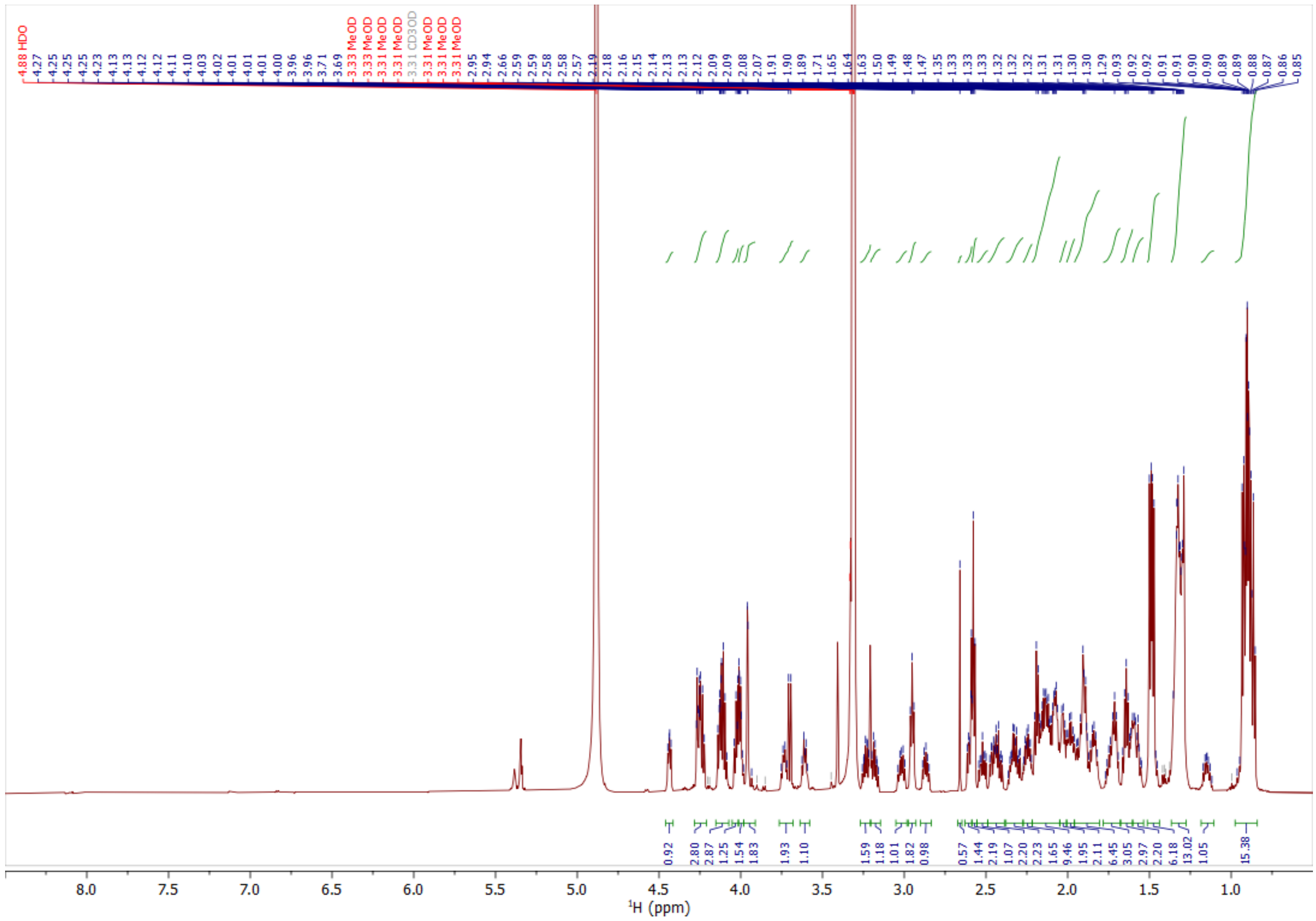

Figure S8: ^{1}H-NMR (700 MHz, MeOH-d_4, 295 K) of **P1A21**. Hydrogen-deuterium exchange was performed prior to the measurement.

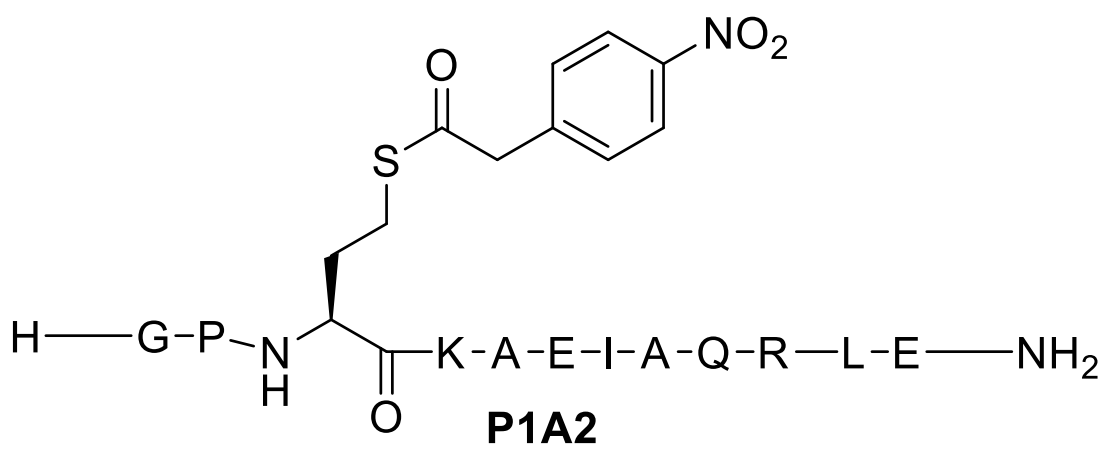

P1A2

Chemical Formula: $C_{64}H_{103}N_{19}O_{20}S$
Exact Mass: 1489.73
Molecular Weight: 1490.70

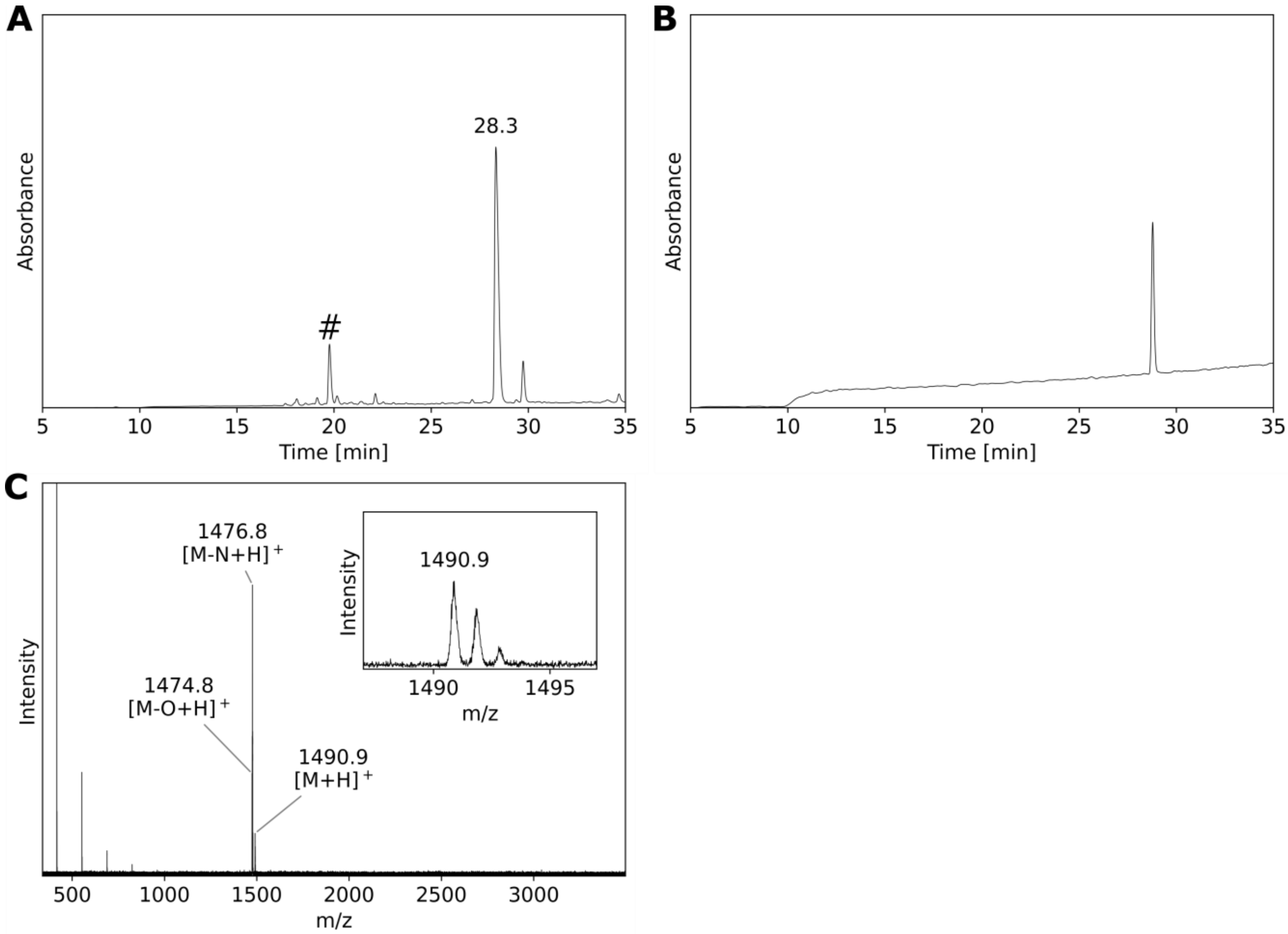

Figure S9: **P1A2** A) Analytical HPLC (220 nm) of crude peptide. HPLC Gradients are listed in Section 2.3.5 Table S3. # Peptide without homoserine. B) Analytical HPLC (220 nm) of pure peptide. C) MALDI-TOF MS of pure peptide. Calculated mass: $[M+H]^+$ 1490.7, $[M-O+H]^+$ 1474.7, $[M-N+H]^+$ 1476.7.

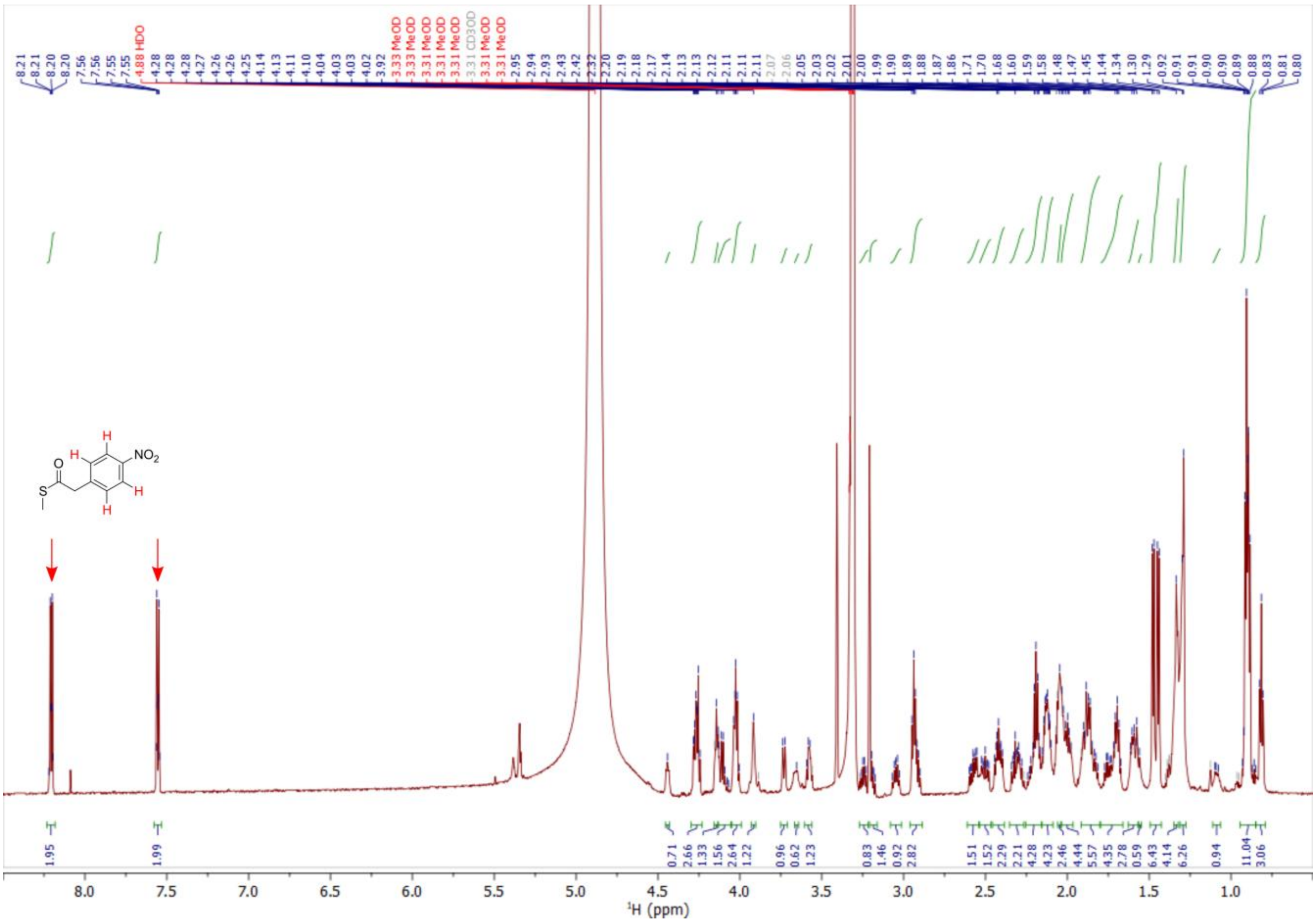

Figure S10: ^{1}H-NMR (700 MHz, MeOH-d_4, 295 K) of **P1A2**. Hydrogen-deuterium exchange was performed prior to the measurement.

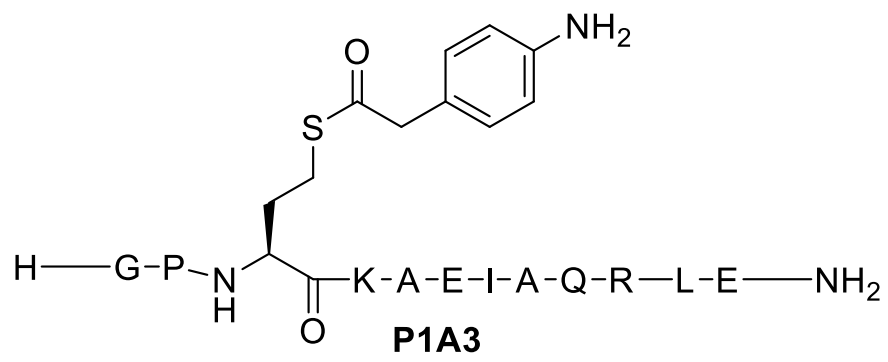

P1A3

Chemical Formula: $C_{64}H_{105}N_{19}O_{18}S$

Exact Mass: 1459.76

Molecular Weight: 1460.72

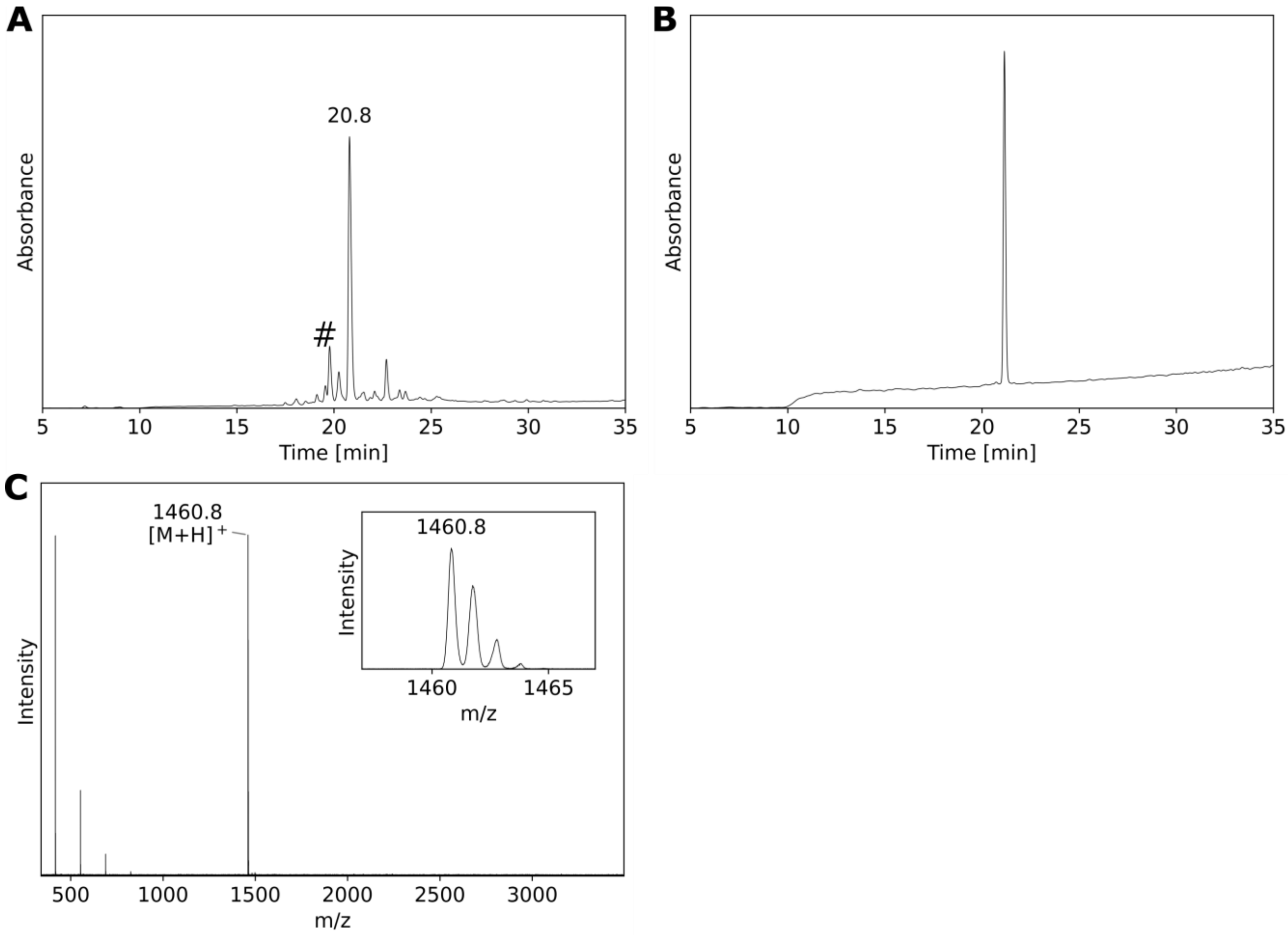

Figure S11: **P1A3** A) Analytical HPLC (220 nm) of crude peptide. HPLC Gradients are listed in Section 2.3.5 Table S3. # Peptide without homoserine. B) Analytical HPLC (220 nm) of pure peptide. C) MALDI-TOF MS of pure peptide. Calculated mass: $[M+H]^+$ 1460.8.

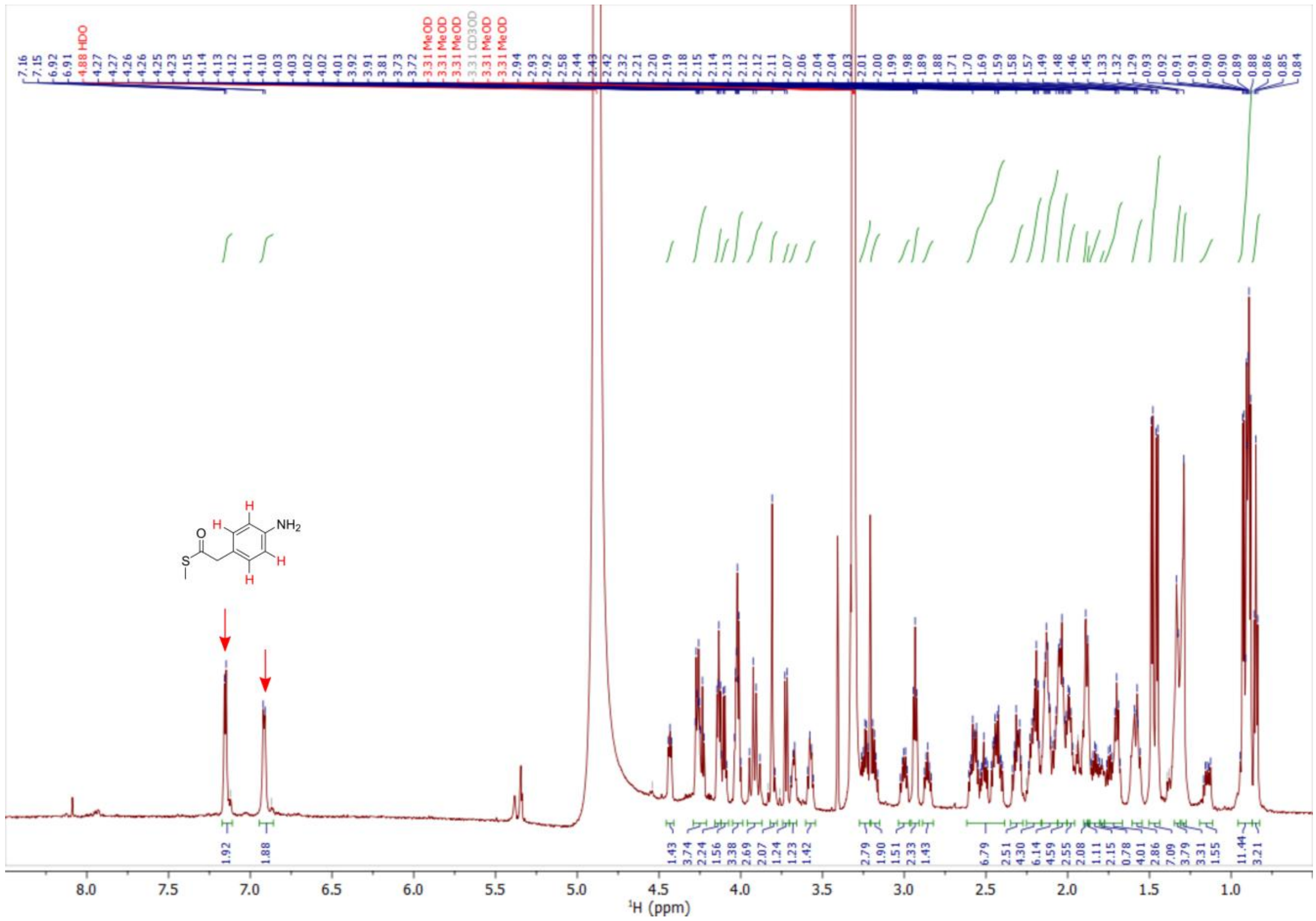

Figure S12: ^{1}H-NMR (700 MHz, MeOH-d_4, 295 K) of **P1A3**. Hydrogen-deuterium exchange was performed prior to the measurement.

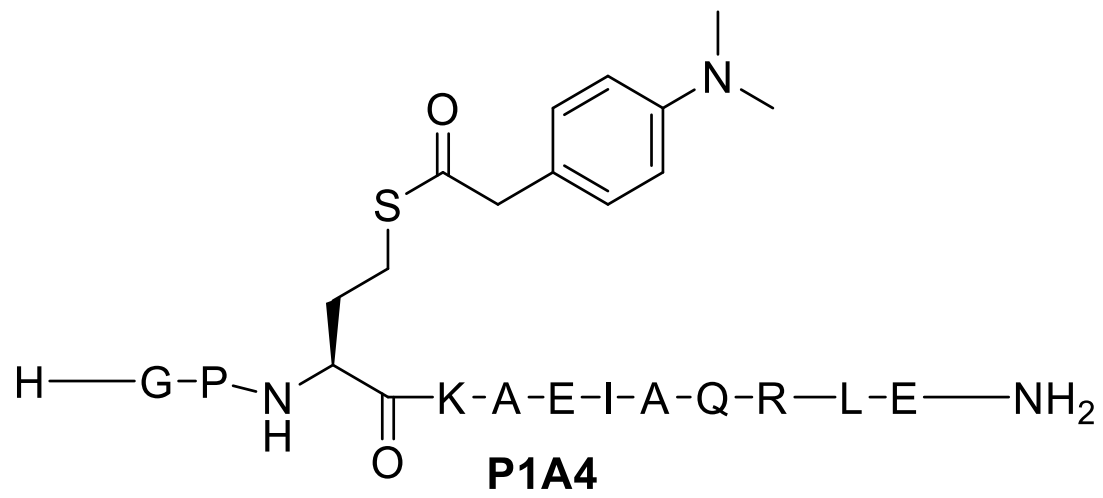

P1A4

Chemical Formula: $C_{66}H_{109}N_{19}O_{18}S$
Exact Mass: 1487.79
Molecular Weight: 1488.77

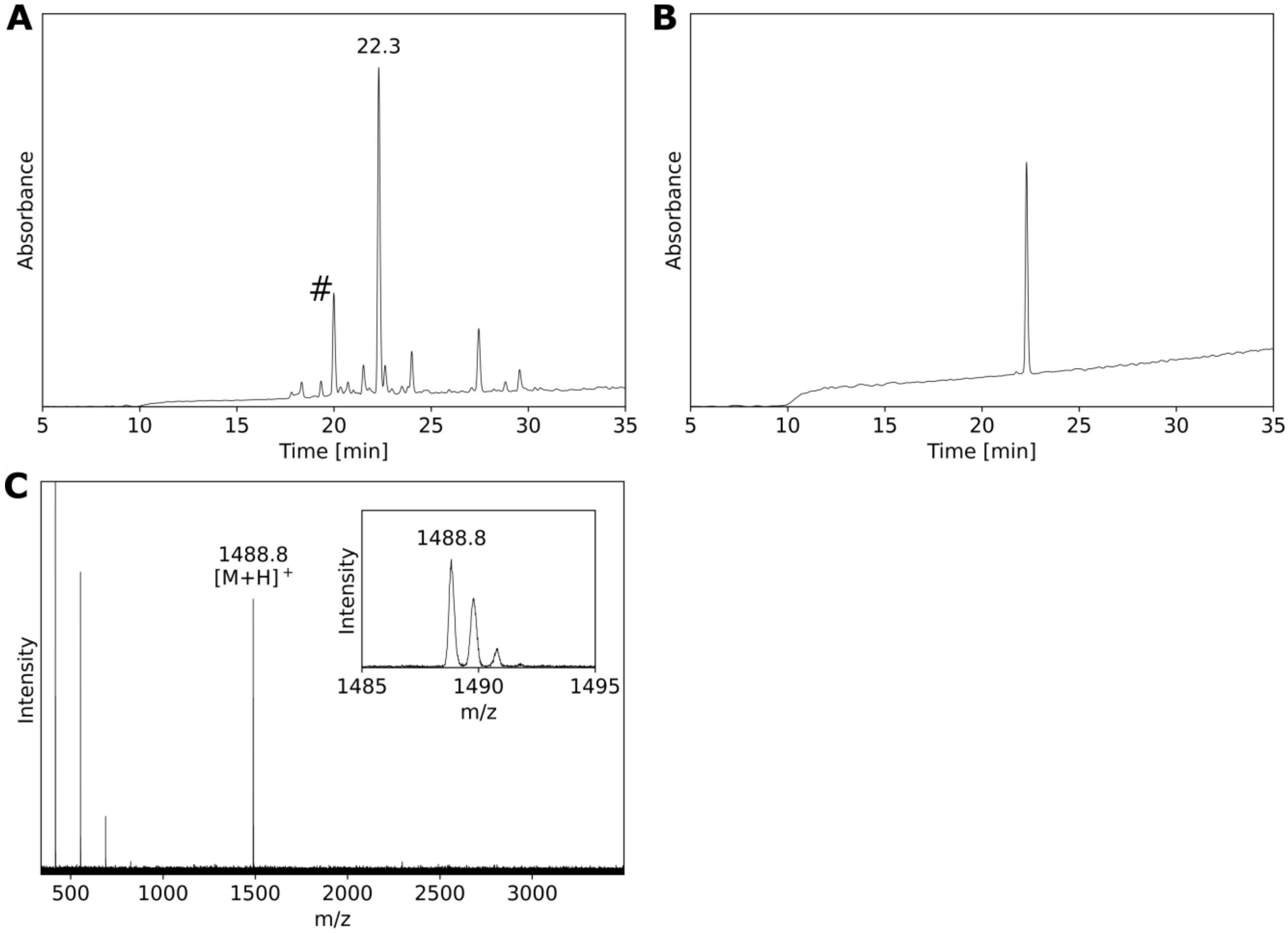

Figure S13: **P1A4** A) Analytical HPLC (220 nm) of crude peptide. HPLC Gradients are listed in Section 2.3.5 Table S3. # Peptide without homoserine. B) Analytical HPLC (220 nm) of pure peptide. C) MALDI-TOF MS of pure peptide. Calculated mass: $[M+H]^+$ 1488.8.

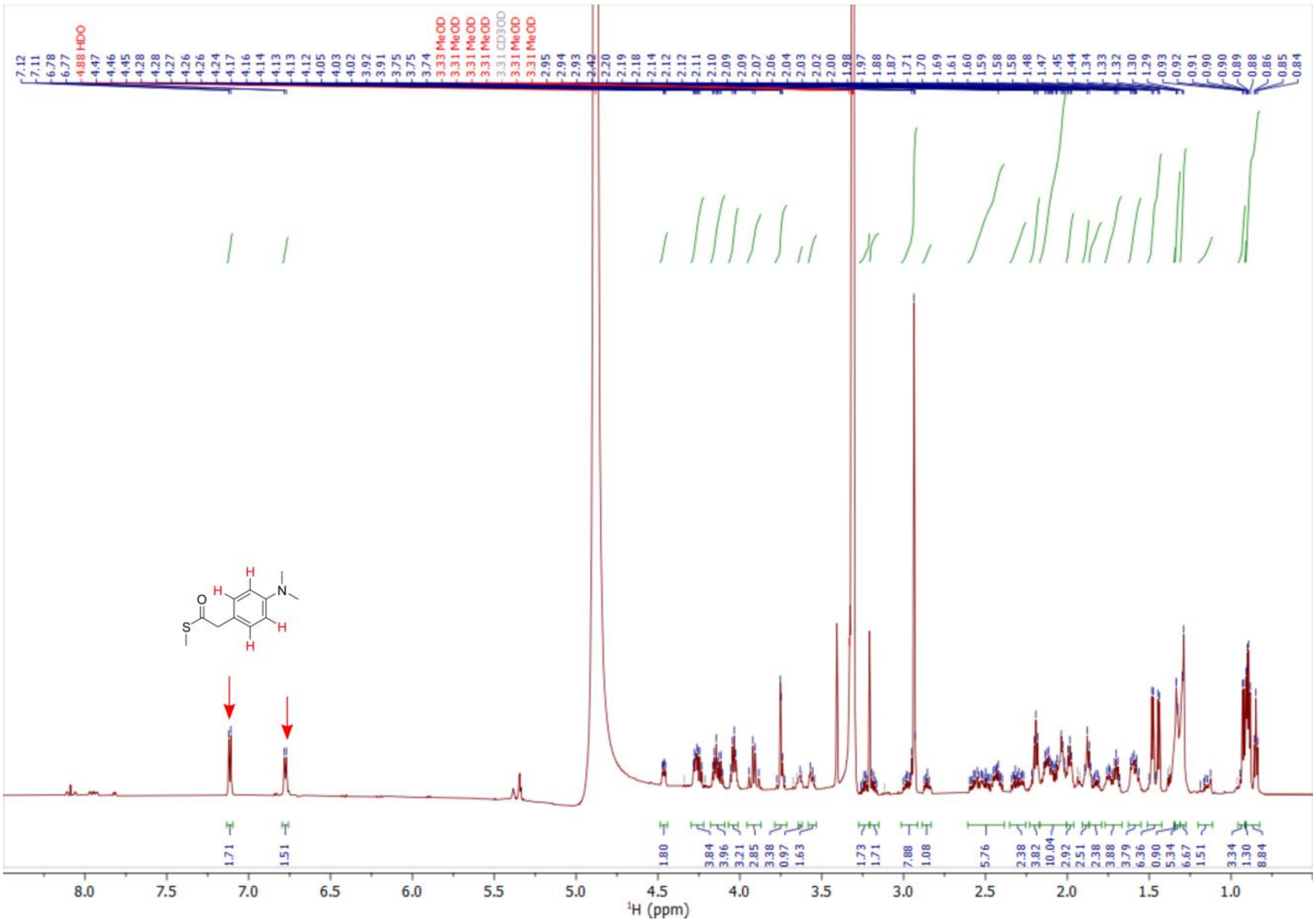

Figure S14: ^{1}H-NMR (700 MHz, MeOH-d_4, 295 K) of **P1A4**. Hydrogen-deuterium exchange was performed prior to the measurement.

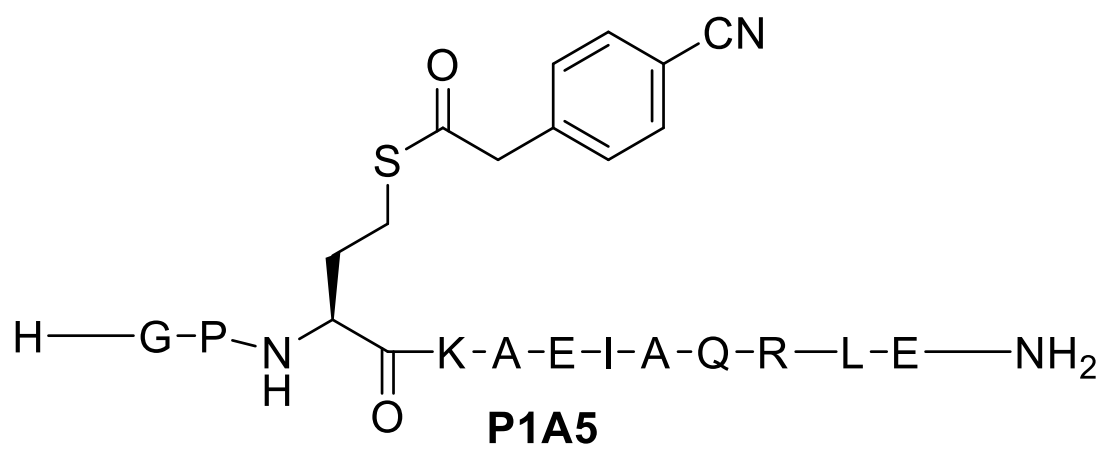

P1A5

Chemical Formula: $C_{65}H_{103}N_{19}O_{18}S$
Exact Mass: 1469.74
Molecular Weight: 1470.71

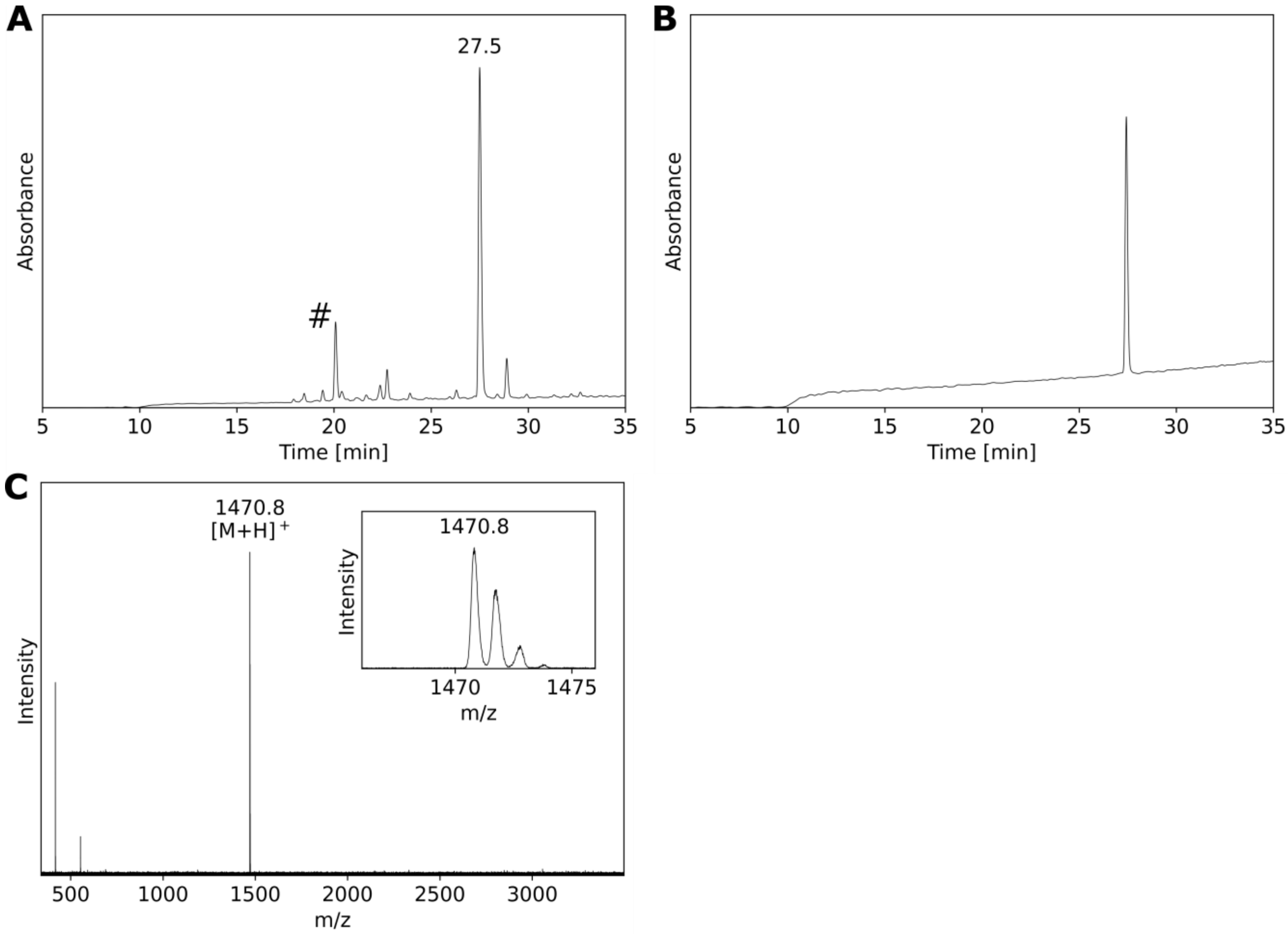

Figure S15: **P1A5** A) Analytical HPLC (220 nm) of crude peptide. HPLC Gradients are listed in Section 2.3.5 Table S3. # Peptide without homoserine. B) Analytical HPLC (220 nm) of pure peptide. C) MALDI-TOF MS of pure peptide. Calculated mass: $[M+H]^+$ 1470.7.

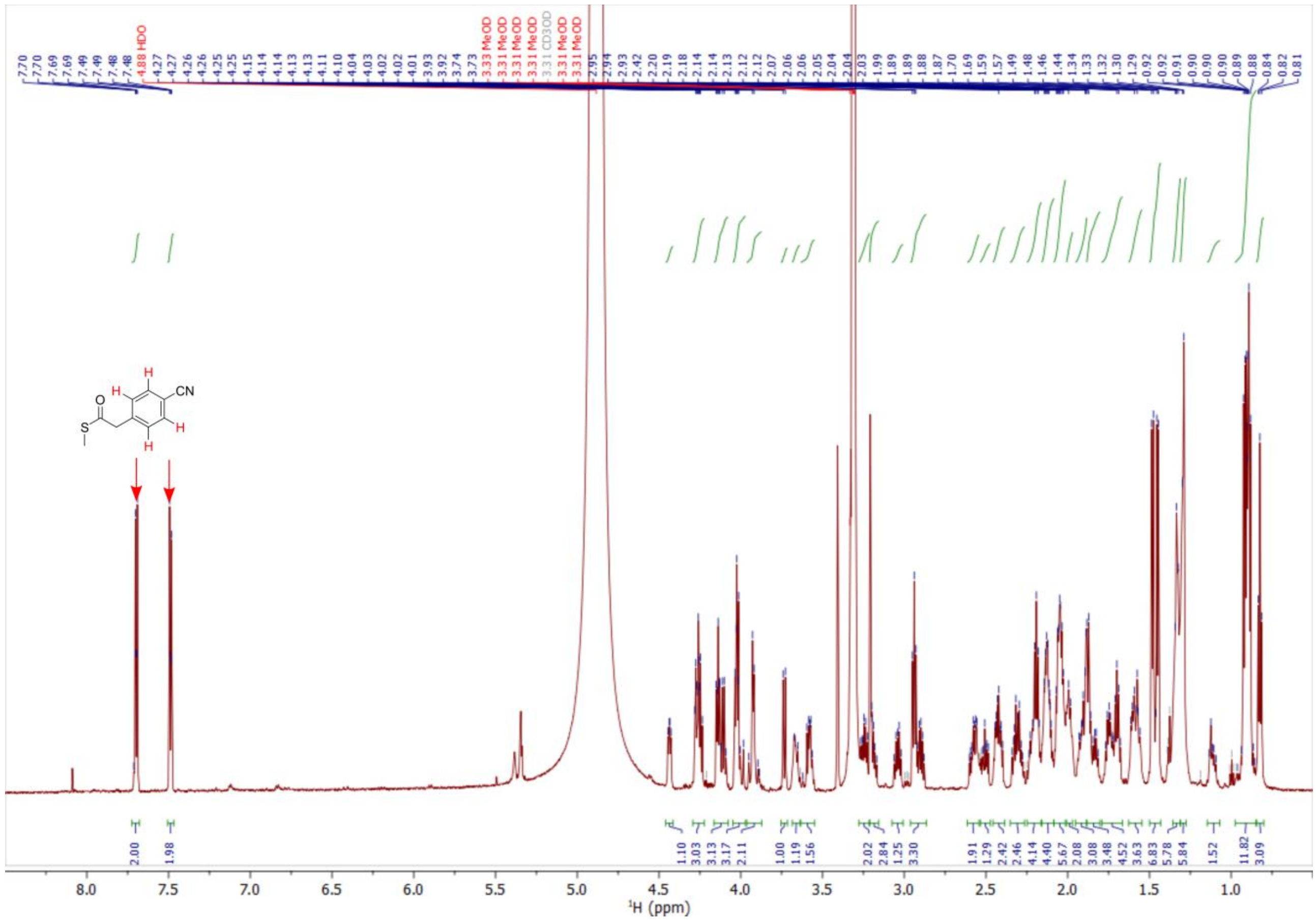

Figure S16: ^{1}H-NMR (700 MHz, MeOH-d_4, 295 K) of **P1A5**. Hydrogen-deuterium exchange was performed prior to the measurement.

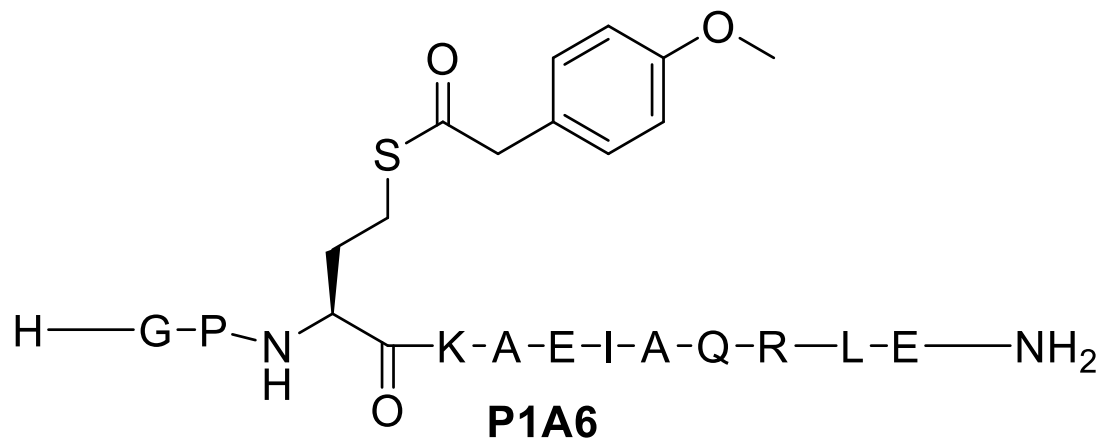

P1A6

Chemical Formula: $C_{65}H_{106}N_{18}O_{19}S$

Exact Mass: 1474.76

Molecular Weight: 1475.73

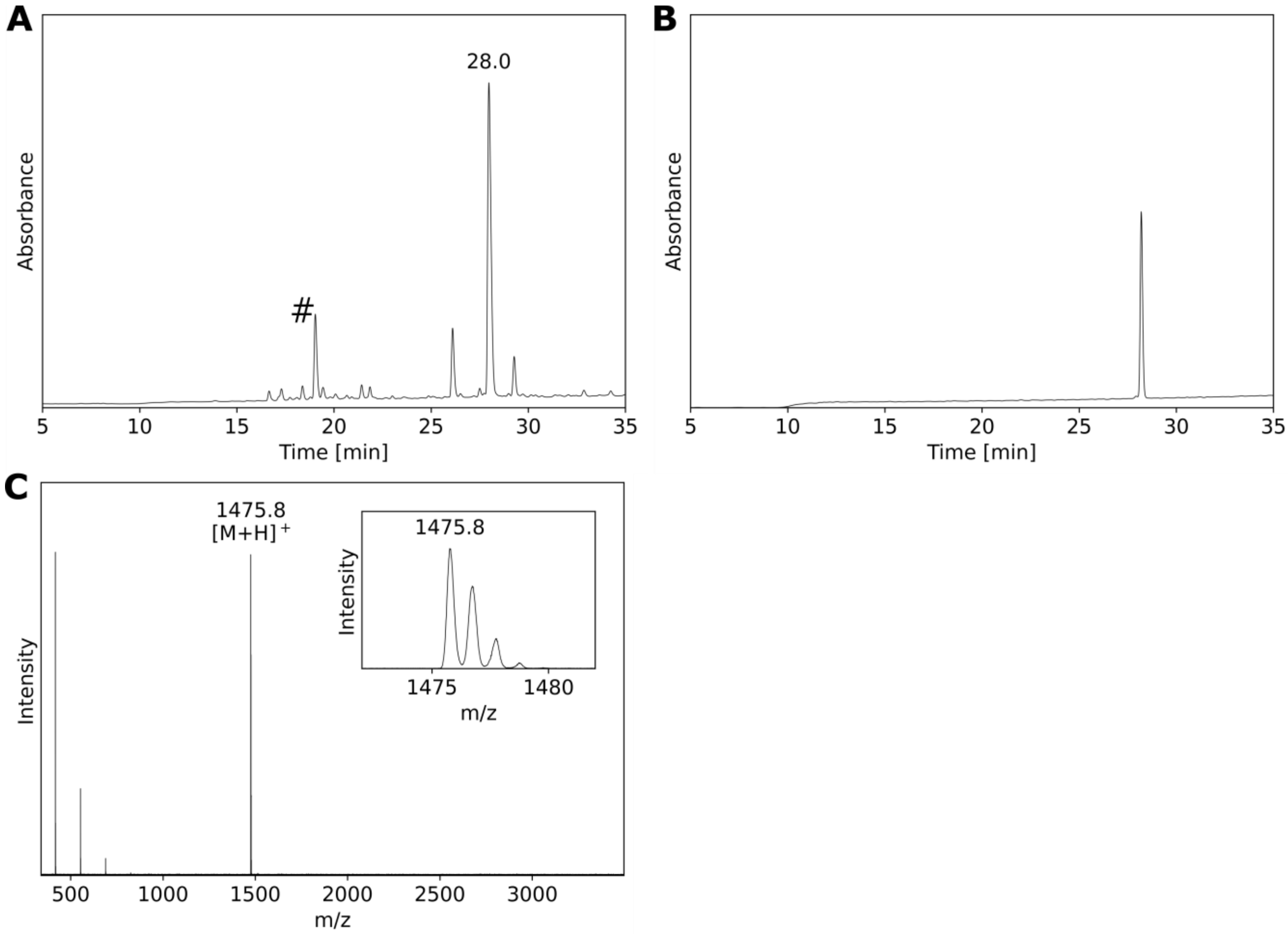

Figure S17: **P1A6** A) Analytical HPLC (220 nm) of crude peptide. HPLC Gradients are listed in Section 2.3.5 Table S3. # Peptide without homoserine. B) Analytical HPLC (220 nm) of pure peptide. C) MALDI-TOF MS of pure peptide. Calculated mass: $[M+H]^+$ 1475.8.

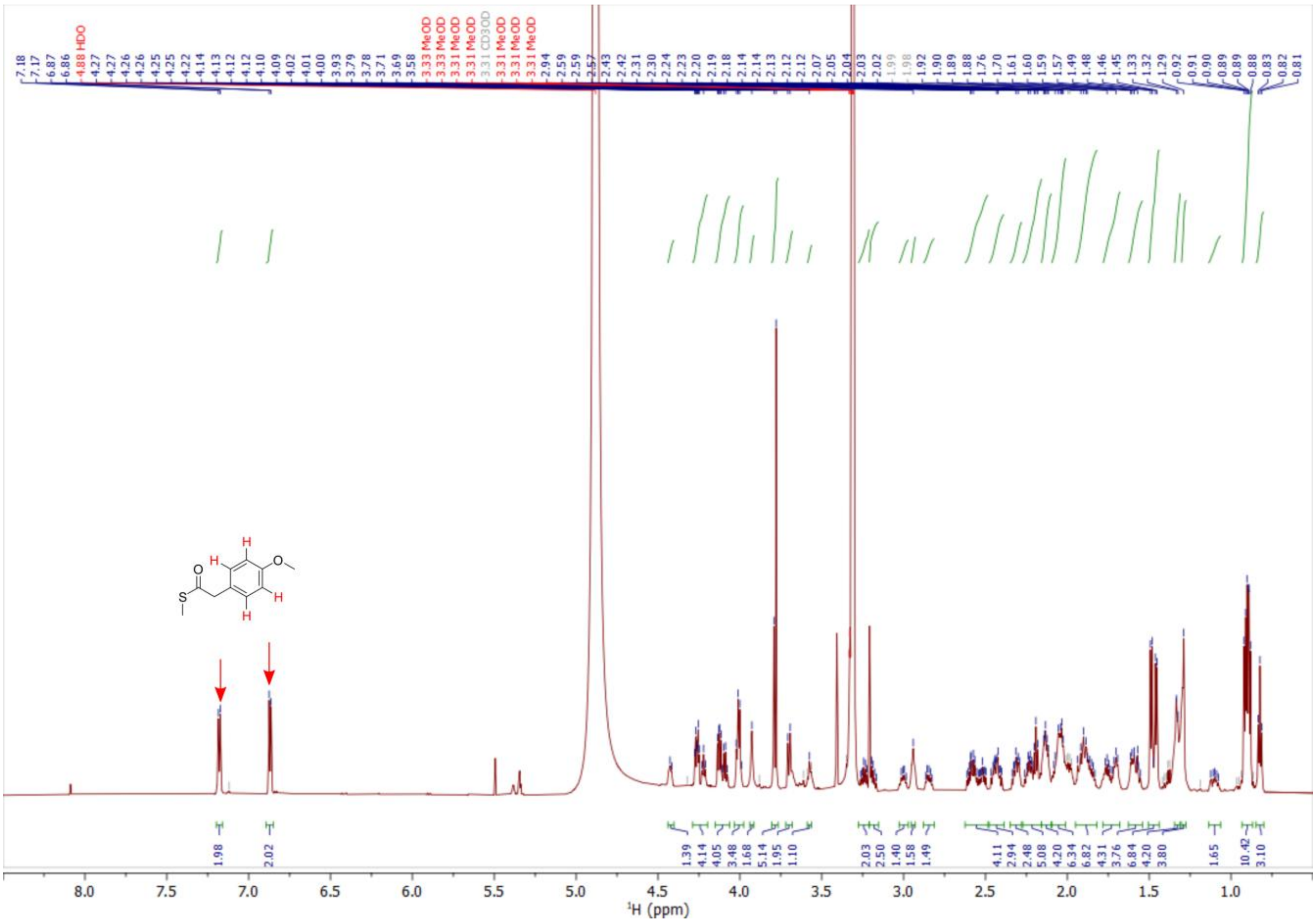

Figure S18: ^{1}H-NMR (700 MHz, MeOH-d_4, 295 K) of **P1A6**. Hydrogen-deuterium exchange was performed prior to the measurement.

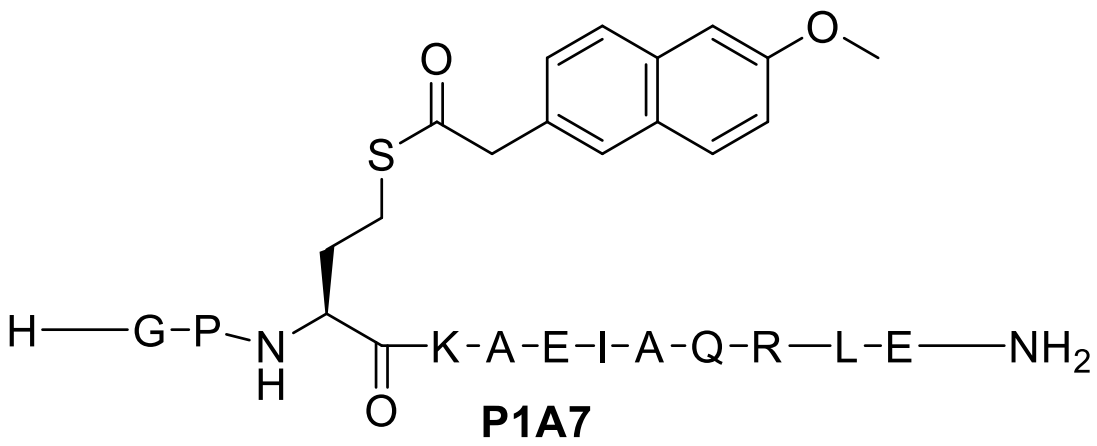

P1A7

Chemical Formula: $C_{69}H_{108}N_{18}O_{19}S$

Exact Mass: 1524.78

Molecular Weight: 1525.79

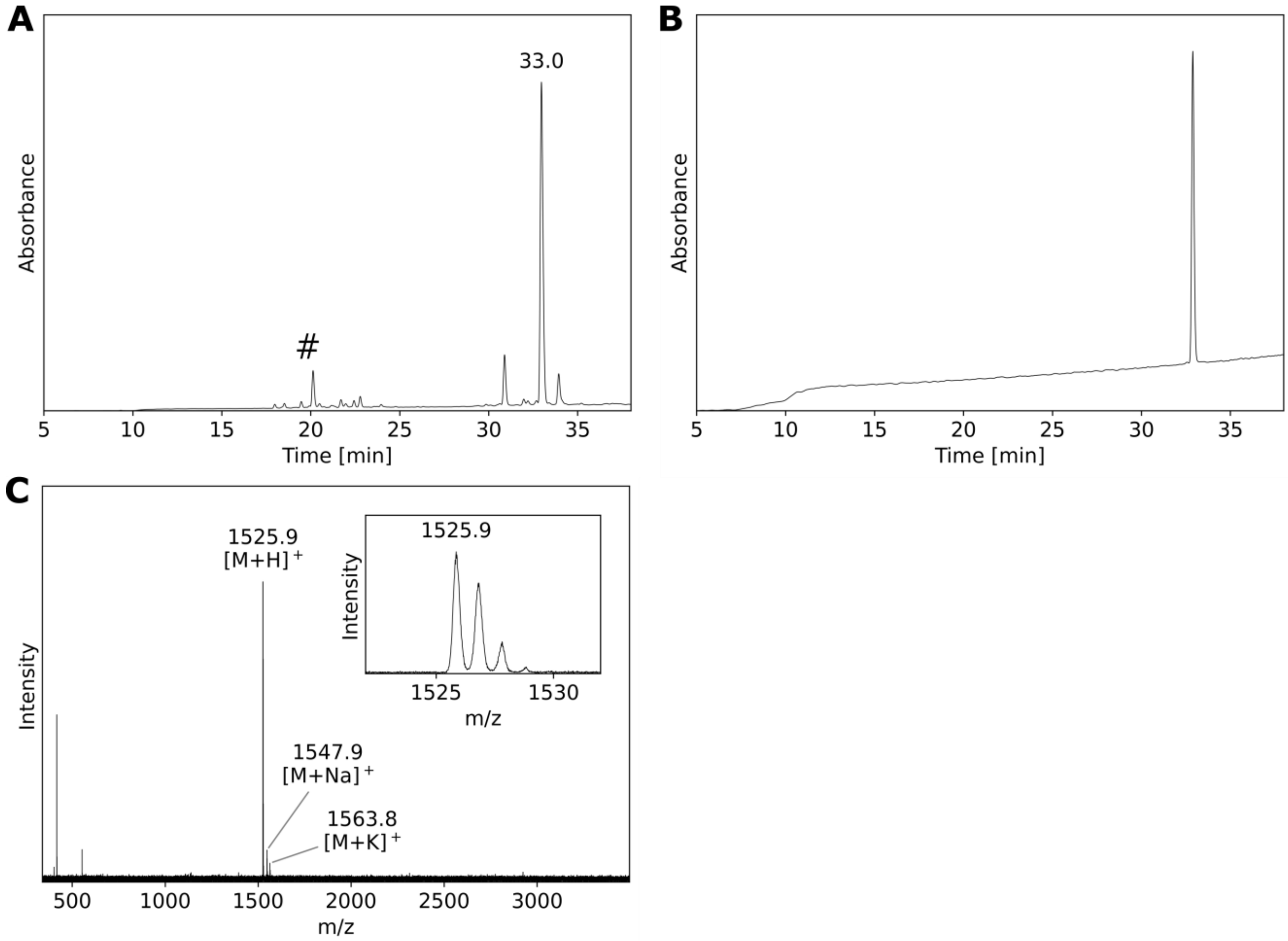

Figure S19: **P1A7** A) Analytical HPLC (220 nm) of crude peptide. HPLC Gradients are listed in Section 2.3.5 Table S3. # Peptide without homoserine. B) Analytical HPLC (220 nm) of pure peptide. C) MALDI-TOF MS of pure peptide. Calculated mass: $[M+H]^+$ 1525.8, $[M+Na]^+$ 1547.8, $[M+H]^+$ 1563.7.

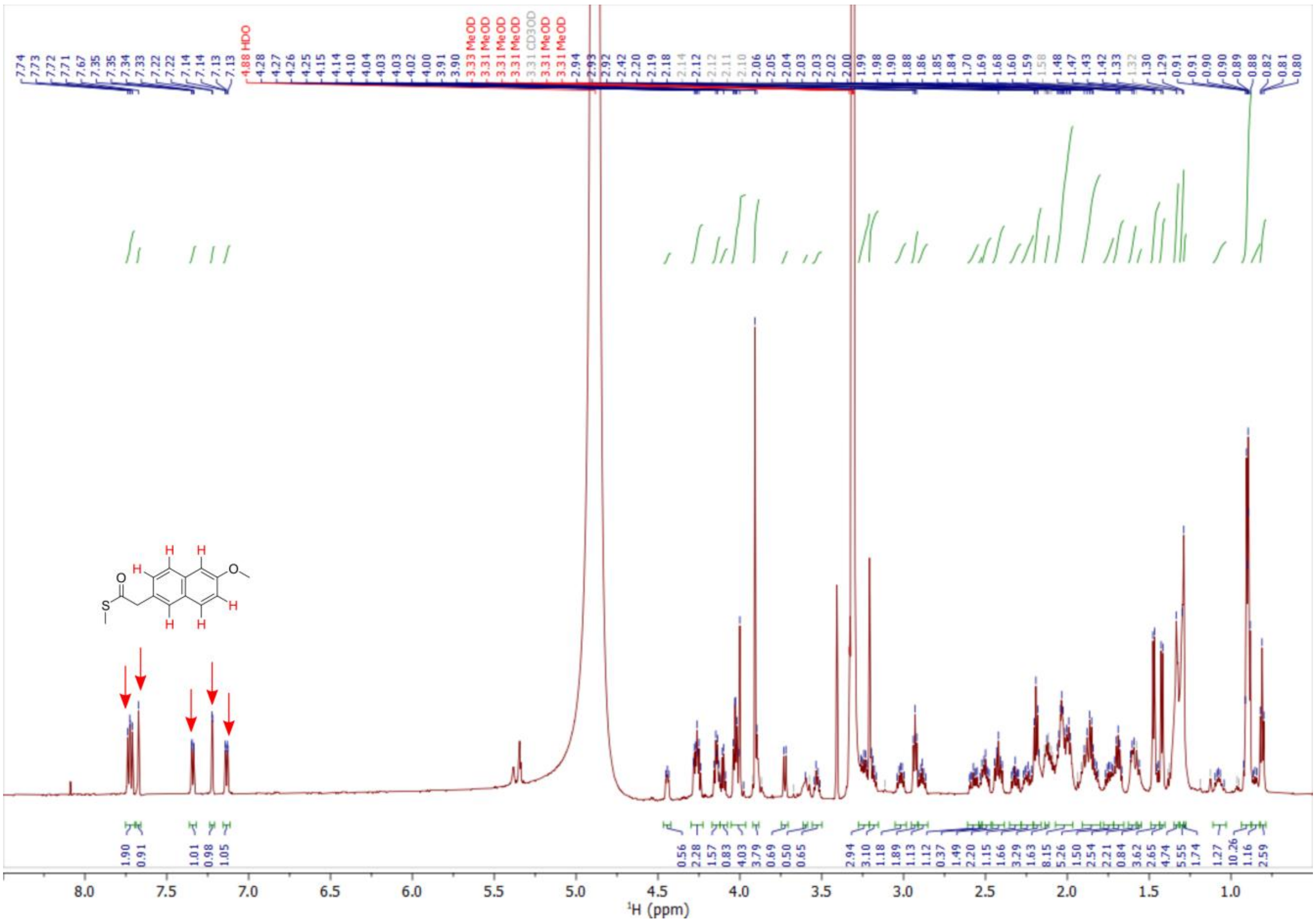

Figure S20: ^{1}H-NMR (700 MHz, MeOH-d_4, 295 K) of **P1A7**. Hydrogen-deuterium exchange was performed prior to the measurement.

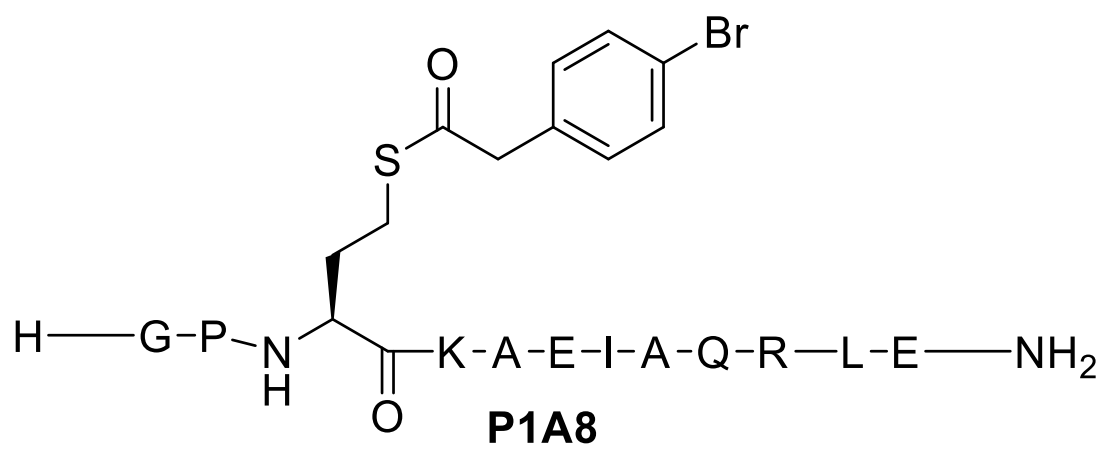

P1A8

Chemical Formula: $C_{64}H_{103}BrN_{18}O_{18}S$

Exact Mass: 1522.66

Molecular Weight: 1524.60

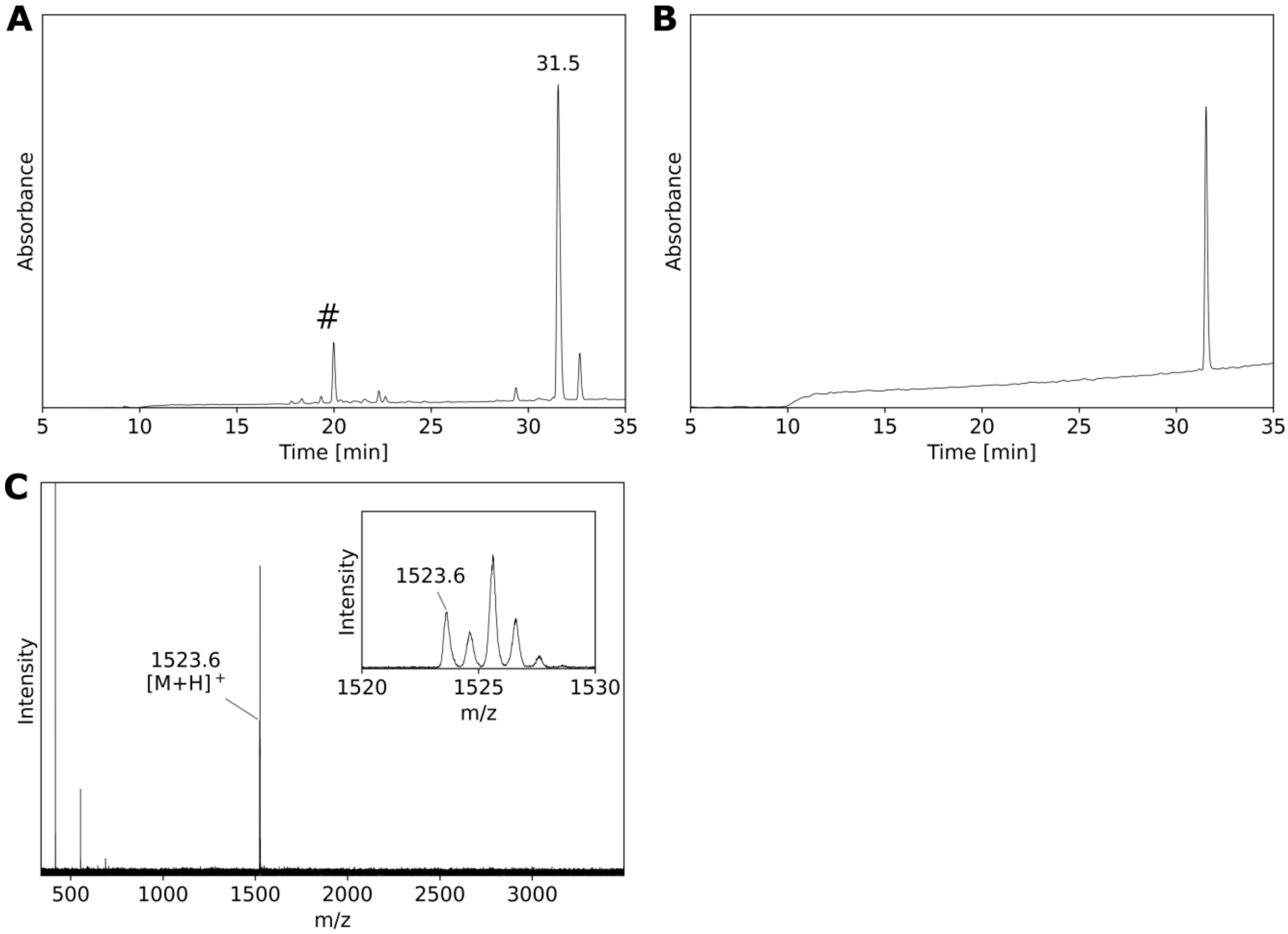

Figure S21: **P1A8** A) Analytical HPLC (220 nm) of crude peptide. HPLC Gradients are listed in Section 2.3.5 Table S3. # Peptide without homoserine. B) Analytical HPLC (220 nm) of pure peptide. C) MALDI-TOF MS of pure peptide. Calculated mass: $[M+H]^+$ 1523.7.

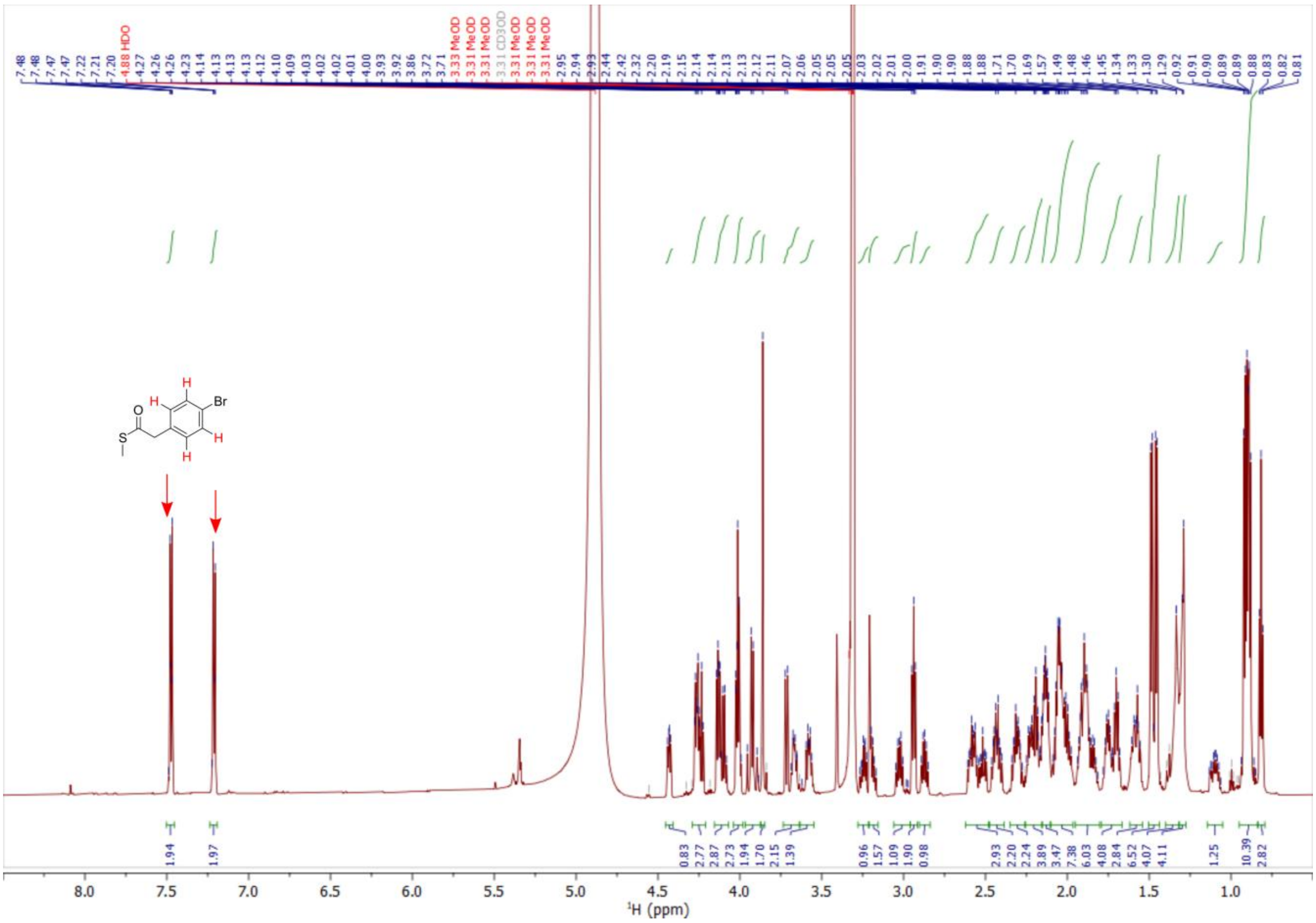

Figure S22: ^{1}H-NMR (700 MHz, MeOH-d_4, 295 K) of **P1A8**. Hydrogen-deuterium exchange was performed prior to the measurement.

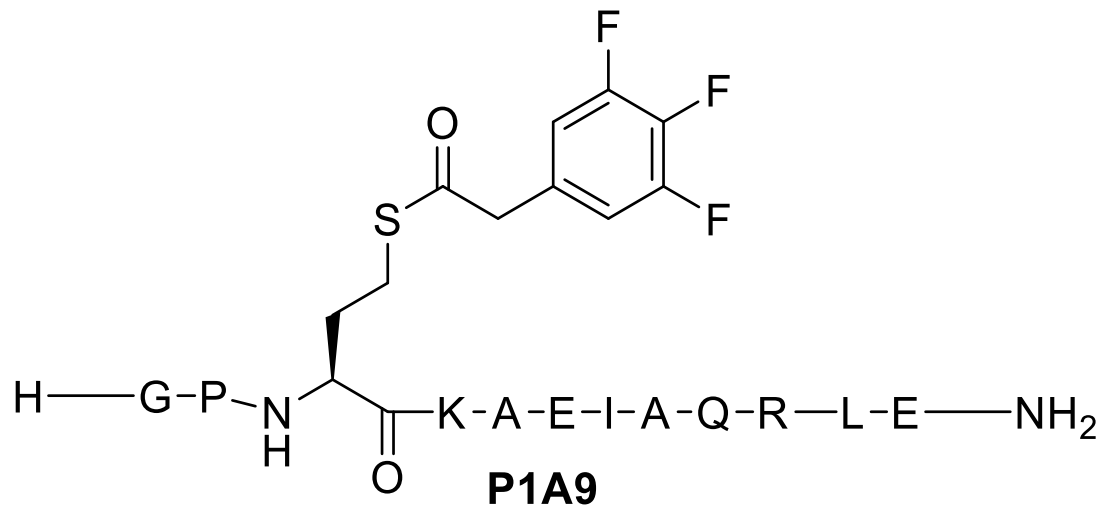

P1A9

Chemical Formula: $C_{64}H_{101}F_3N_{18}O_{18}S$
Exact Mass: 1498.72
Molecular Weight: 1499.68

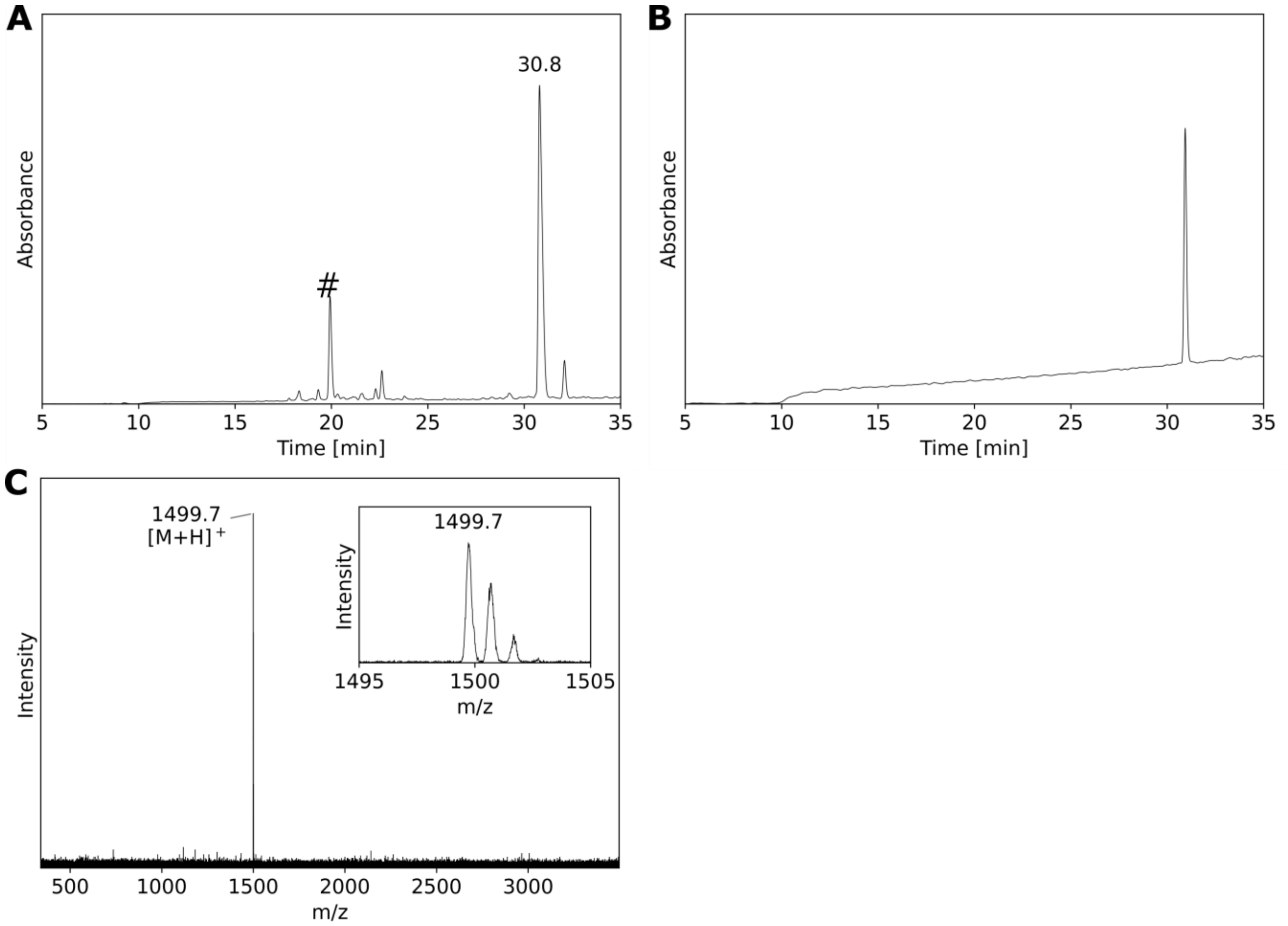

Figure S23: **P1A9** A) Analytical HPLC (220 nm) of crude peptide. HPLC Gradients are listed in Section 2.3.5 Table S3. # Peptide without homoserine. B) Analytical HPLC (220 nm) of pure peptide. C) MALDI-TOF MS of pure peptide. Calculated mass: $[M+H]^+$ 1499.7.

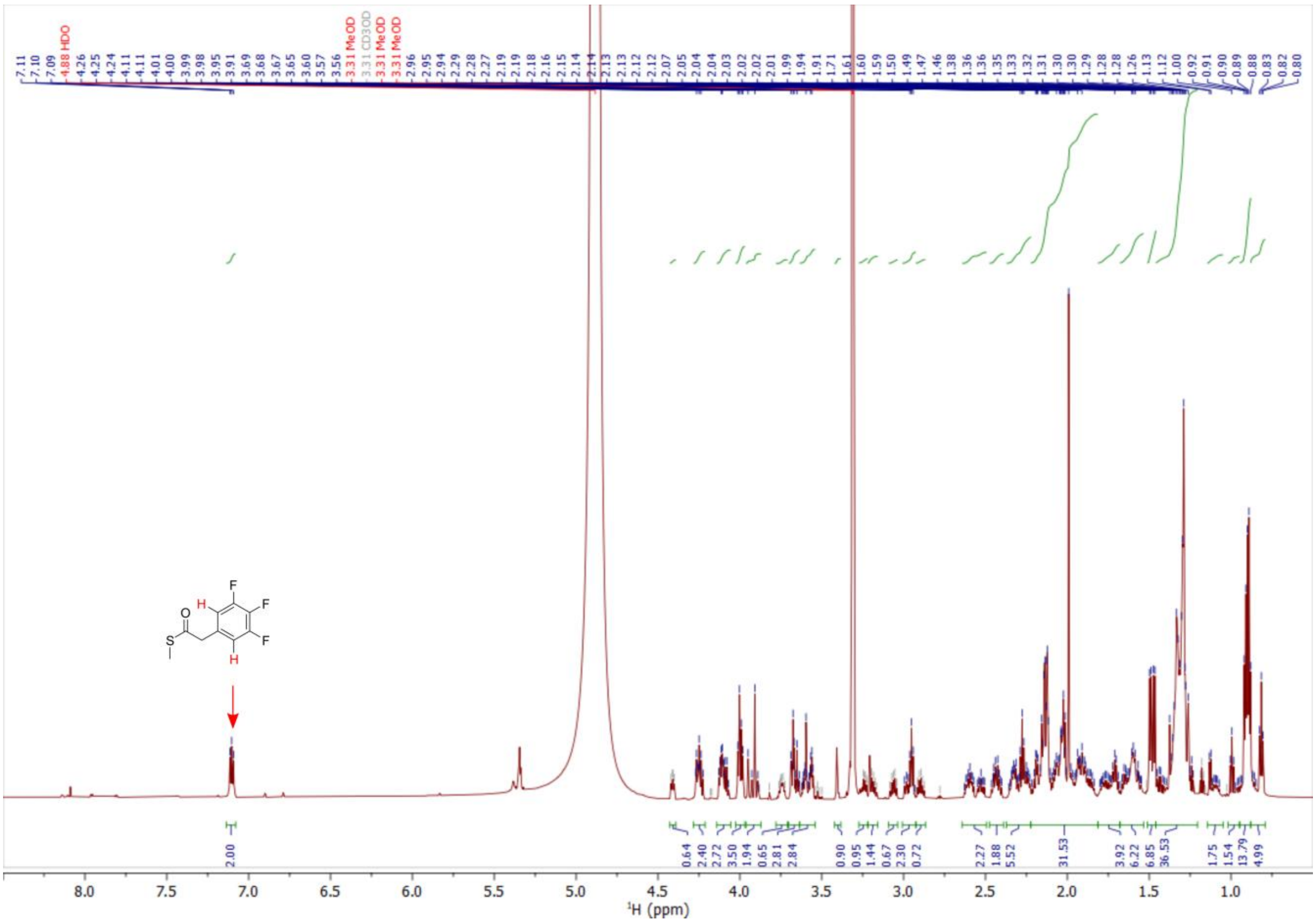

Figure S24: ^{1}H-NMR (700 MHz, MeOH-d_4, 295 K) of **P1A9**. Hydrogen-deuterium exchange was performed prior to the measurement.

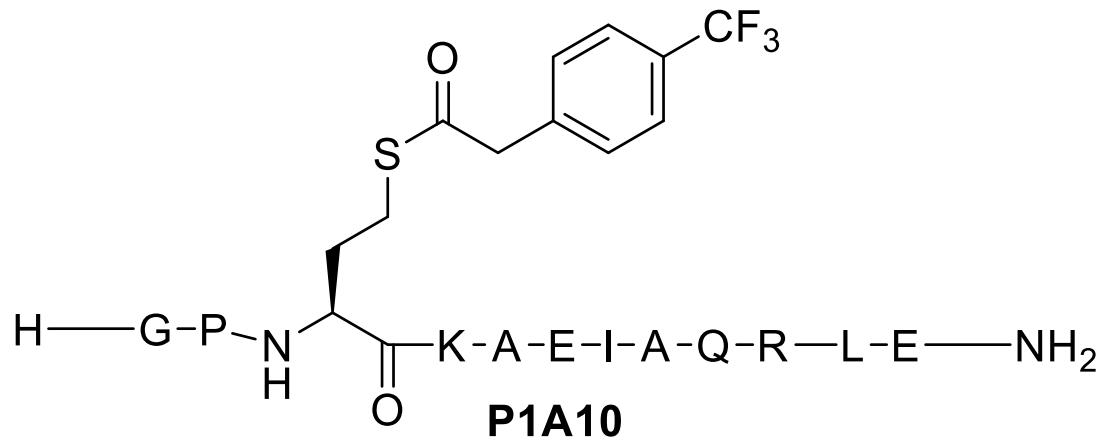

P1A10

Chemical Formula: $C_{65}H_{103}F_3N_{18}O_{18}S$

Exact Mass: 1512.74

Molecular Weight: 1513.70

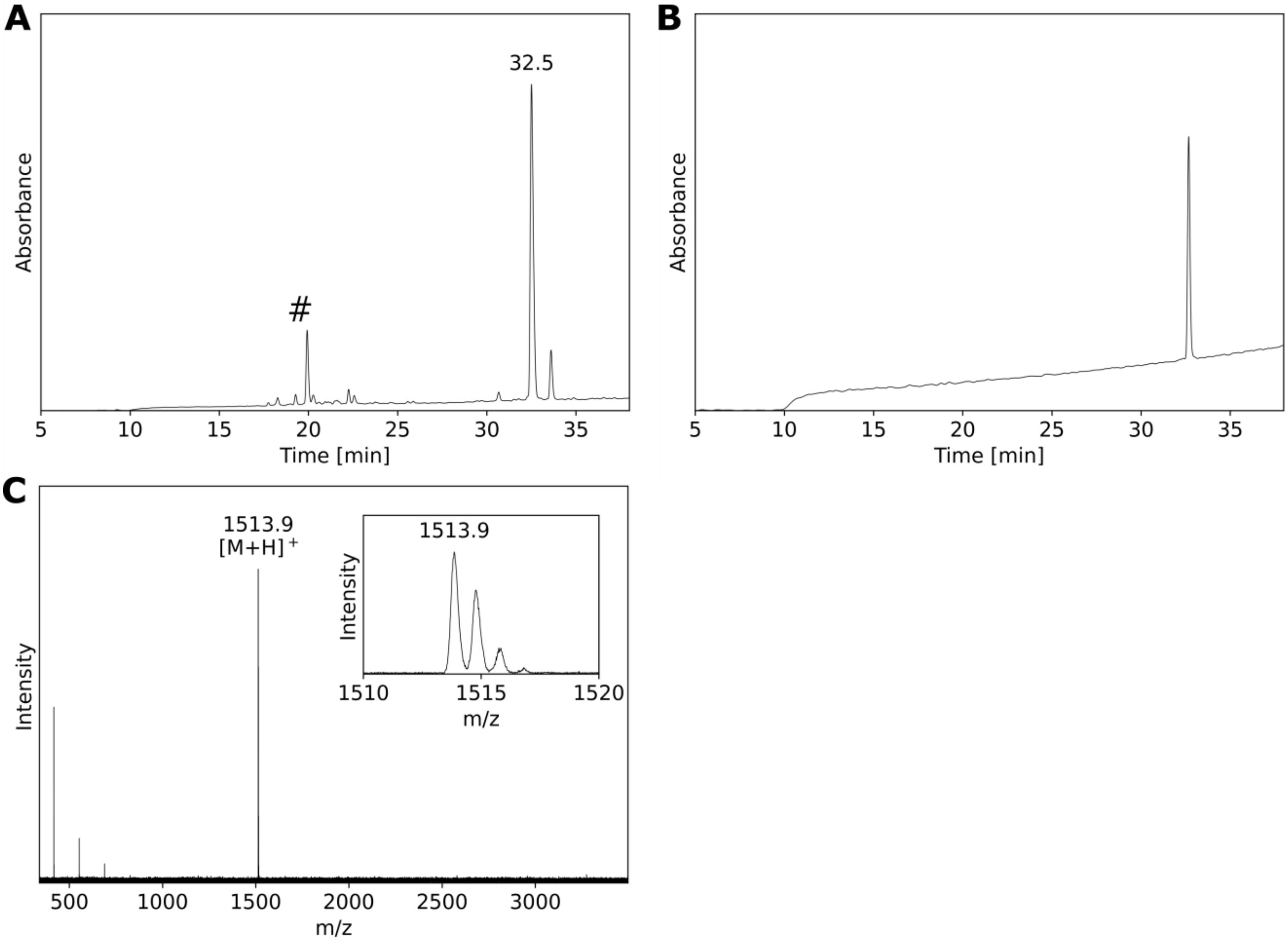

Figure S25: **P1A10** A) Analytical HPLC (220 nm) of crude peptide. HPLC Gradients are listed in Section 2.3.5 Table S3. # Peptide without homoserine. B) Analytical HPLC (220 nm) of pure peptide. C) MALDI-TOF MS of pure peptide. Calculated mass: $[M+H]^+$ 1513.7.

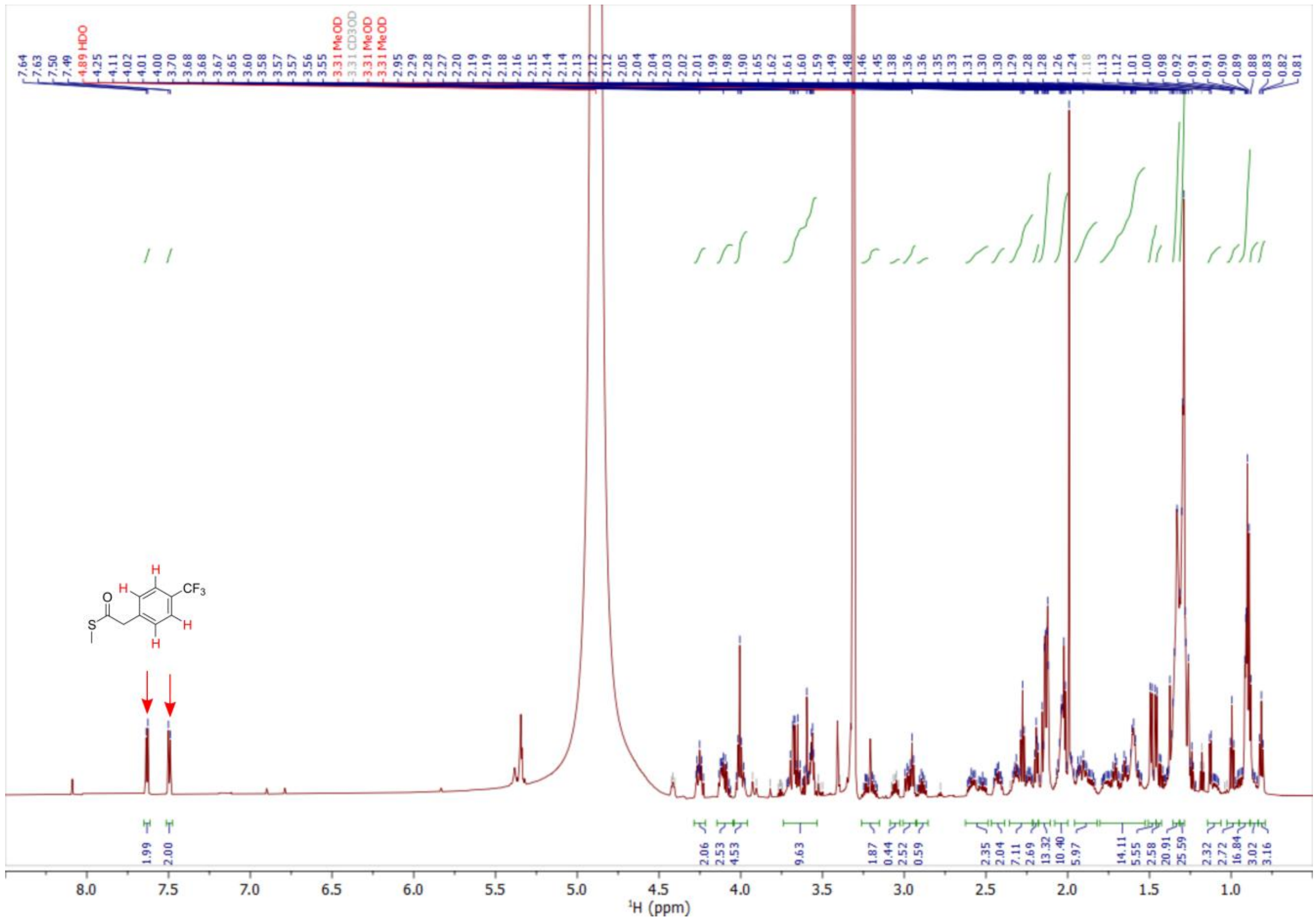

Figure S26: ¹H-NMR (700 MHz, MeOH-d_4, 295 K) of **P1A10**. Hydrogen-deuterium exchange was performed prior to the measurement.

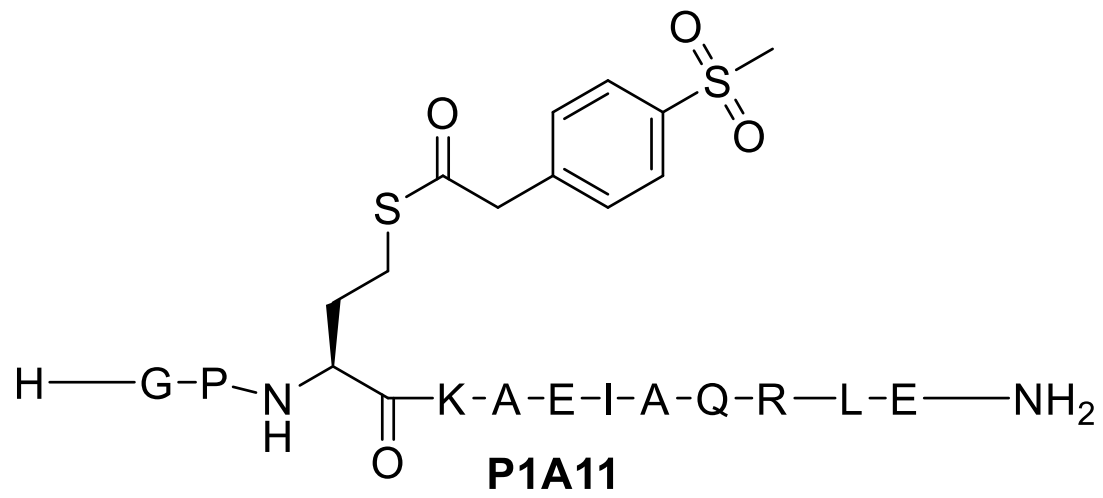

P1A11

Chemical Formula: $C_{65}H_{106}N_{18}O_{20}S_2$
Exact Mass: 1522.73
Molecular Weight: 1523.79

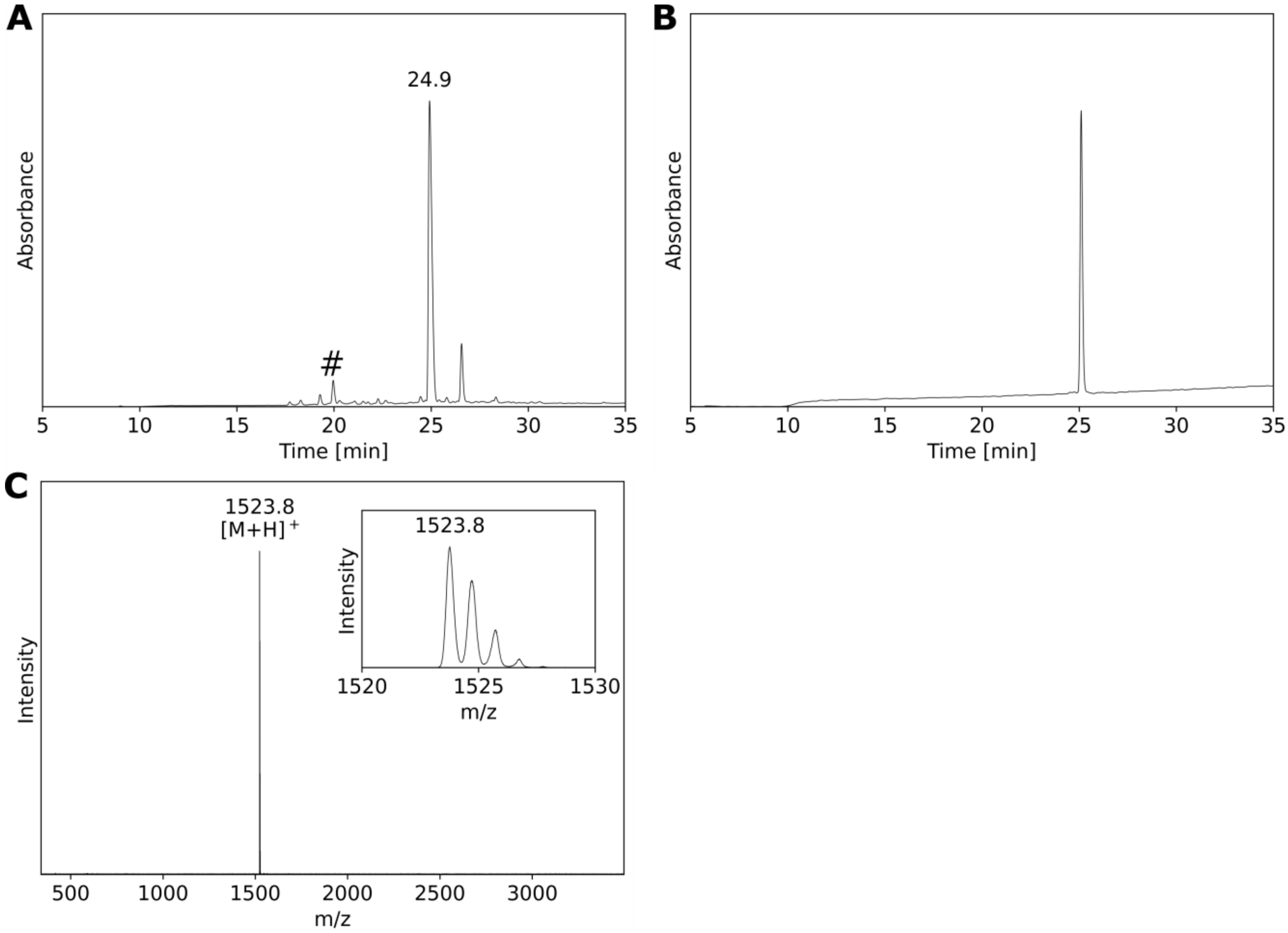

Figure S27: **P1A11** A) Analytical HPLC (220 nm) of crude peptide. HPLC Gradients are listed in Section 2.3.5 Table S3. # Peptide without homoserine. B) Analytical HPLC (220 nm) of pure peptide. C) MALDI-TOF MS of pure peptide. Calculated mass: $[M+H]^+$ 1523.7.

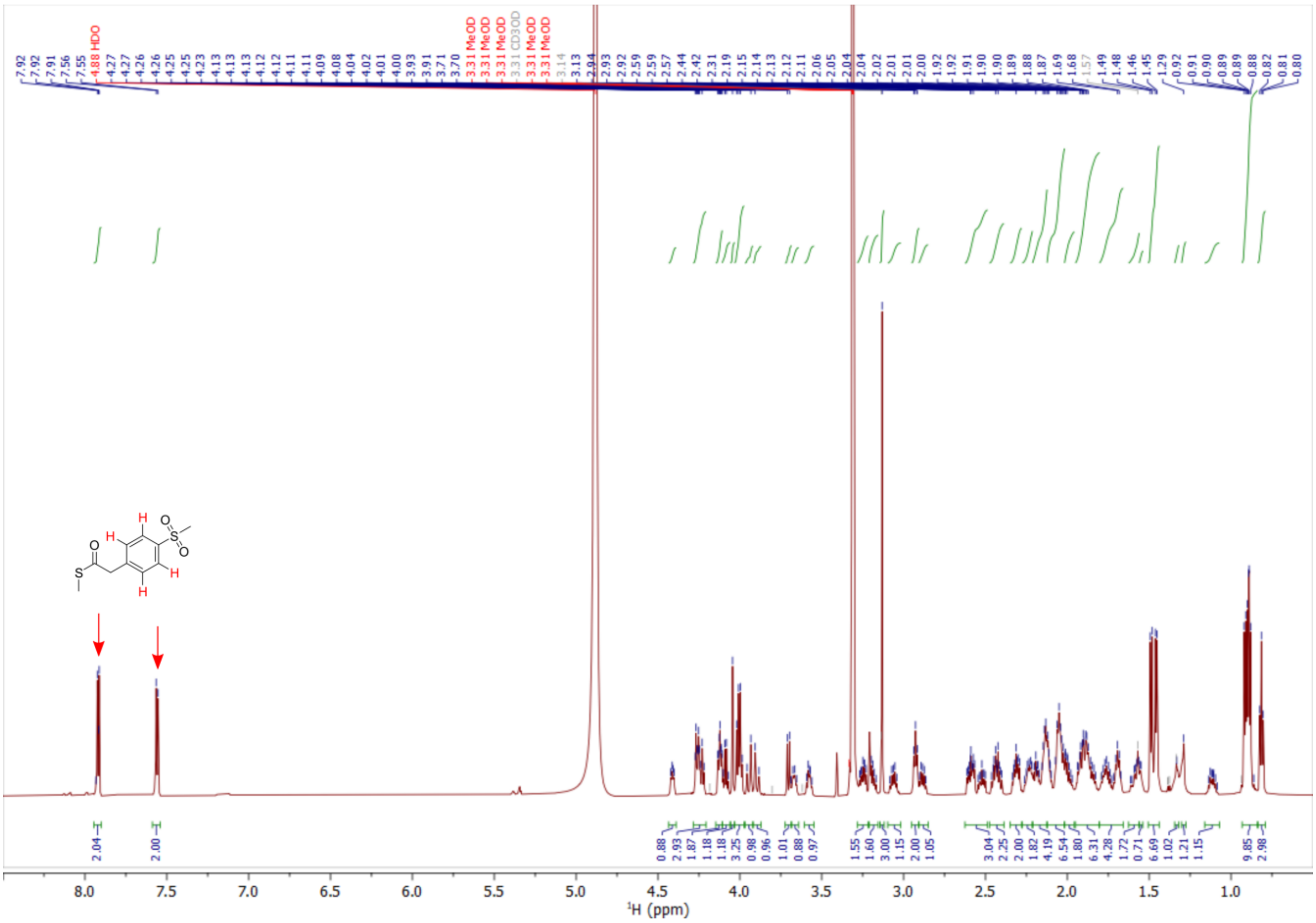

Figure S28: ^{1}H-NMR (700 MHz, MeOH-d_4, 295 K) of **P1A11**. Hydrogen-deuterium exchange was performed prior to the measurement.

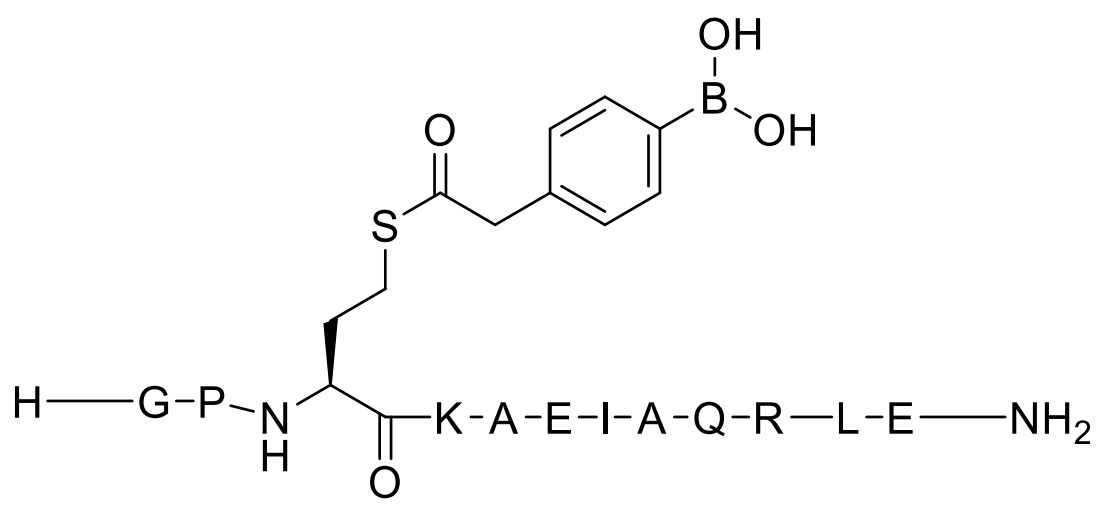

P1A12

Chemical Formula: $C_{64}H_{105}BN_{18}O_{20}S$
Exact Mass: 1488.76
Molecular Weight: 1489.52

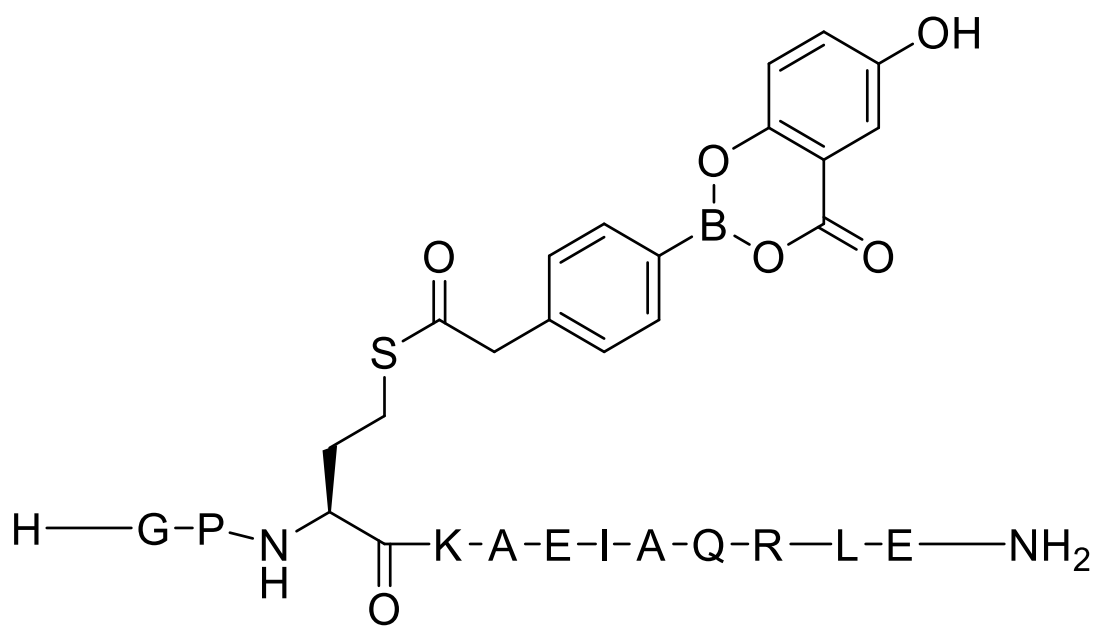

P1A12 x DHB

Chemical Formula: $C_{71}H_{107}BN_{18}O_{22}S$
Exact Mass: 1606.76
Molecular Weight: 1607.61

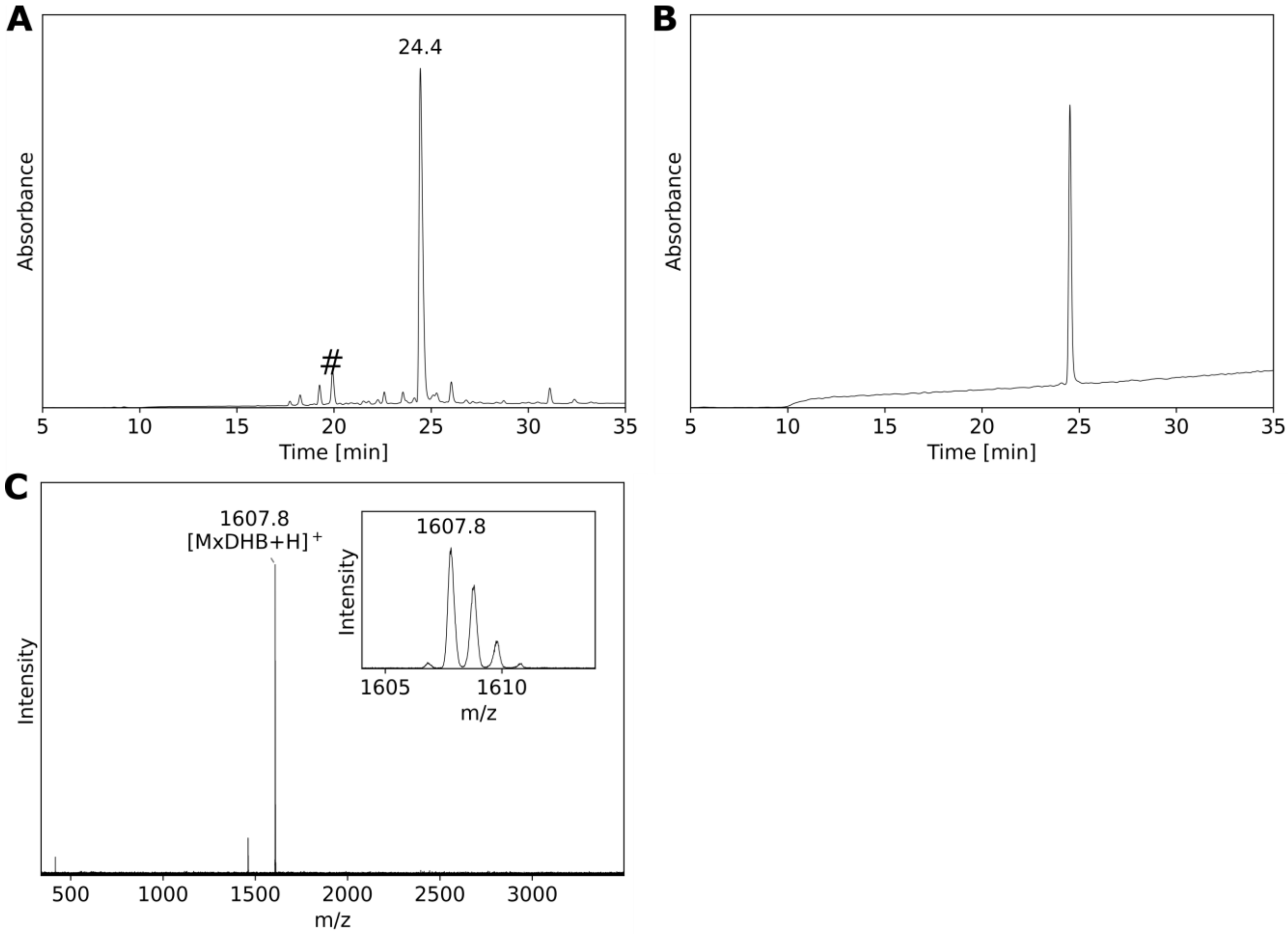

Figure S29: **P1A12** A) Analytical HPLC (220 nm) of crude peptide. HPLC Gradients are listed in Section 2.3.5 Table S3. # Peptide without homoserine. B) Analytical HPLC (220 nm) of pure peptide. C) MALDI-TOF MS of pure peptide. The formation of adducts between boronic acid and DHB was observed, which is described in the literature.[1] Calculated mass: $[MxDHB+H]^+$ 1607.8.

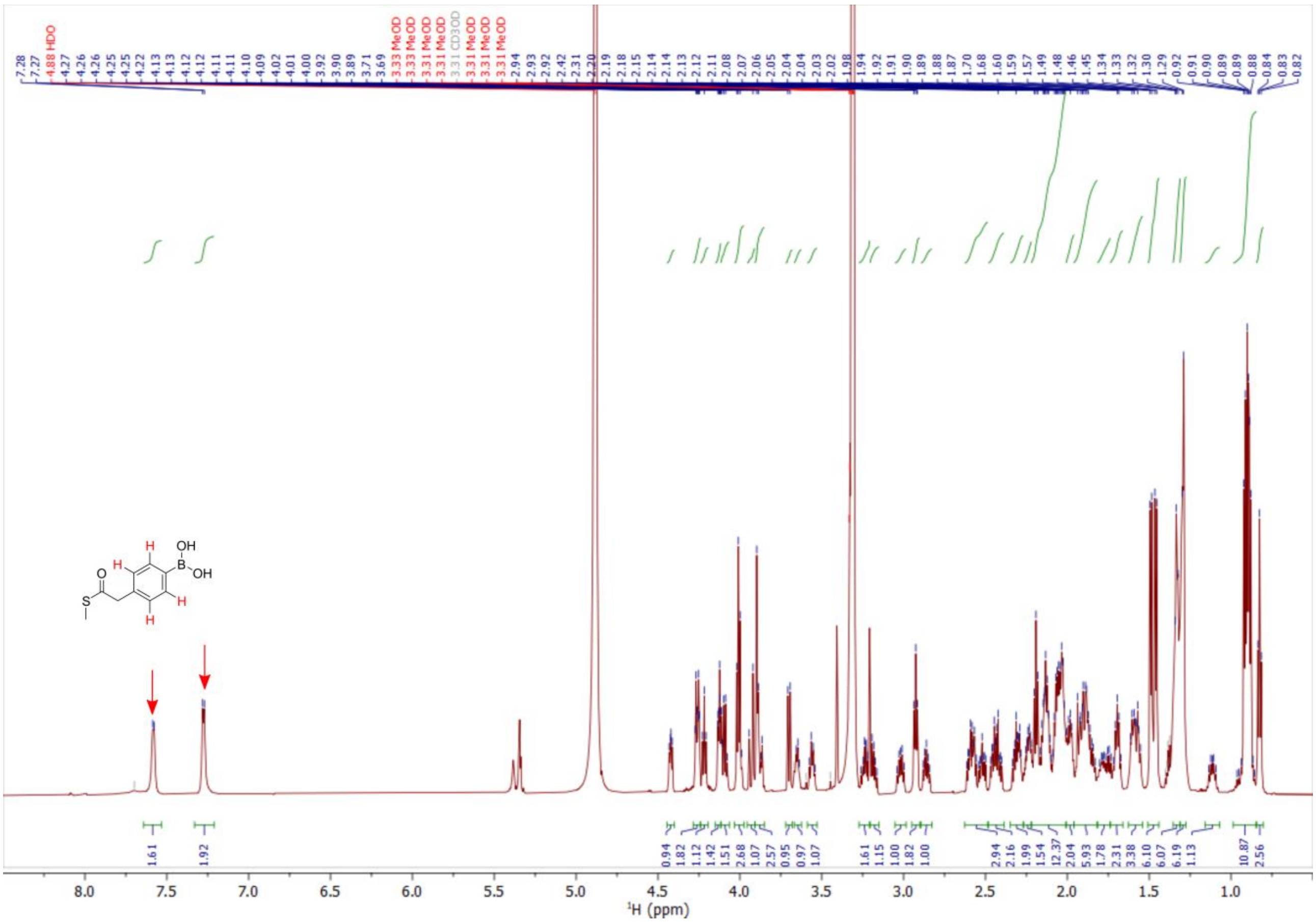

Figure S30: ^{1}H-NMR (700 MHz, MeOH-d_4, 295 K) of **P1A12**. Hydrogen-deuterium exchange was performed prior to the measurement.

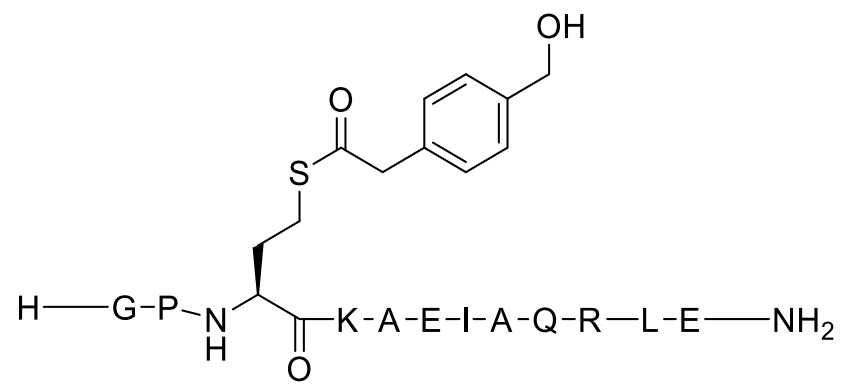

P1A13-Alcohol

Chemical Formula: $C_{65}H_{106}N_{18}O_{19}S$
Exact Mass: 1474.7602
Molecular Weight: 1475.7300

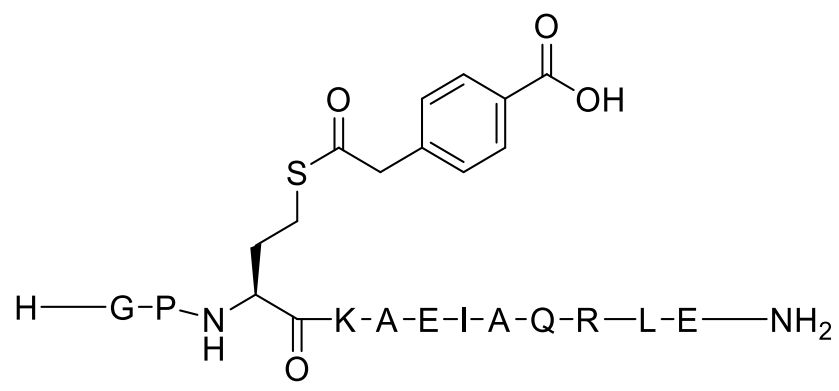

P1A13-Carboxylic acid

Chemical Formula: $C_{65}H_{104}N_{18}O_{20}S$
Exact Mass: 1488.74
Molecular Weight: 1489.71

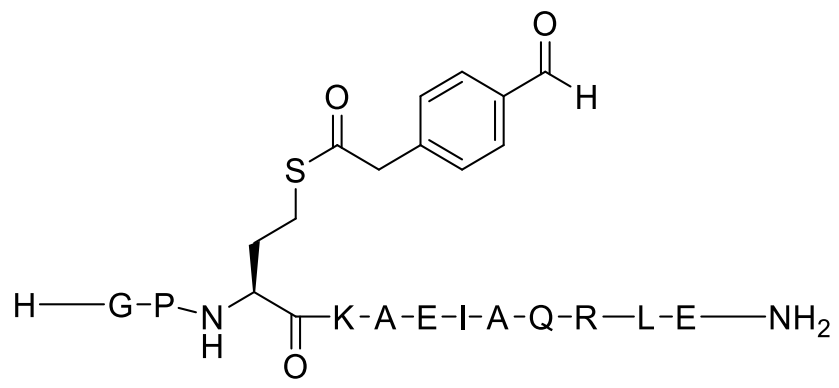

P1A13

Chemical Formula: $C_{65}H_{104}N_{18}O_{19}S$
Exact Mass: 1472.74
Molecular Weight: 1473.71

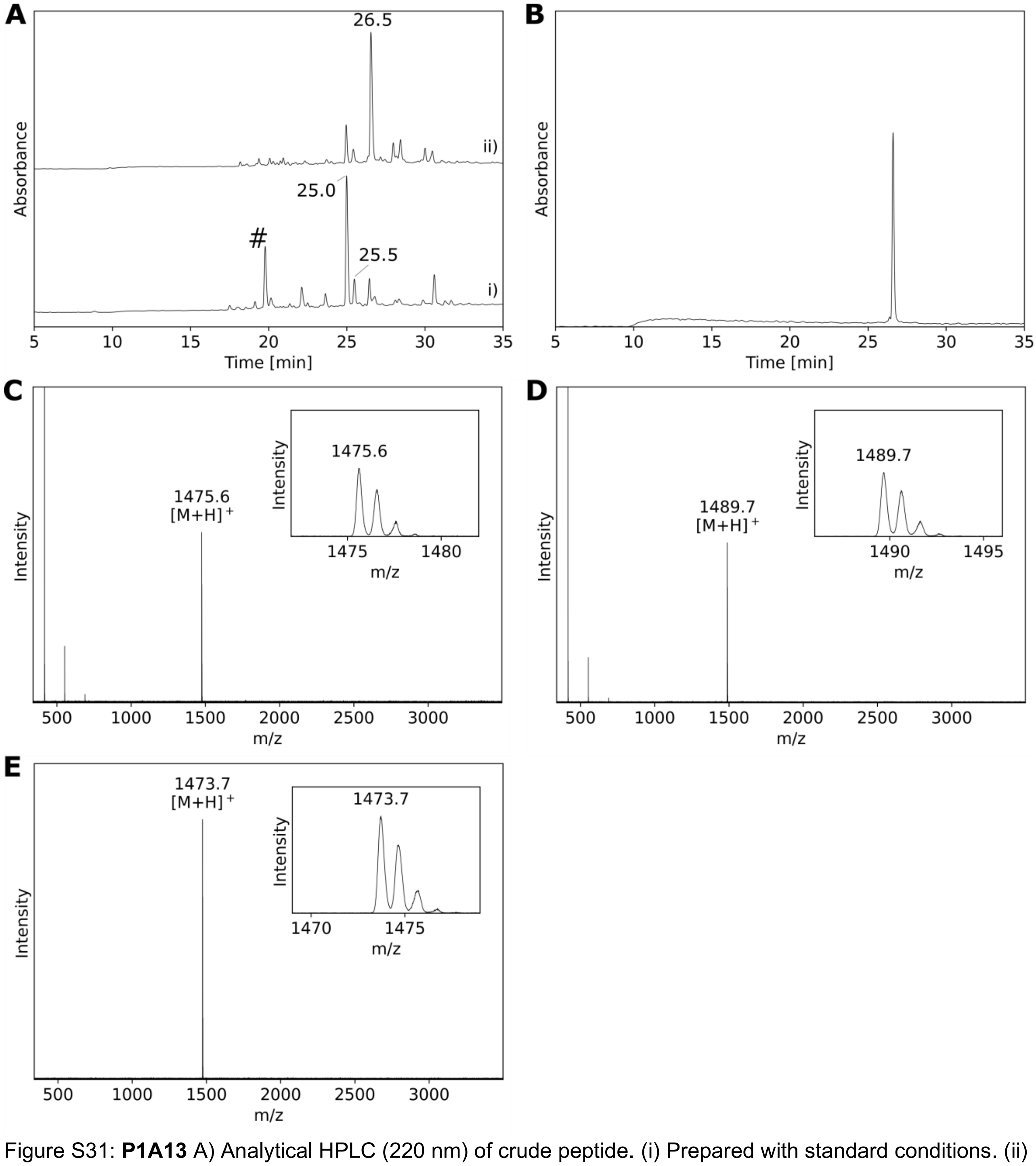

Figure S31: **P1A13** A) Analytical HPLC (220 nm) of crude peptide. (i) Prepared with standard conditions. (ii) Benzaldehyde was converted into an imine with propylamine prior to S-alkynylation. HPLC Gradients are listed in Section 2.3.5 Table S3. # Peptide without homoserine. B) Analytical HPLC (220 nm) of pure peptide. C) MALDI-TOF MS of purified peptide with a retention time of 25.0 min. D) MALDI-TOF MS of purified peptide with a retention time of 25.5 min. E) MALDI-TOF MS of purified peptide with a retention time of 26.5 min. Calculated mass for **P1A13-Alcohol**: $[M+H]^+$ 1475.8. Calculated mass for **P1A13-Carboxylic acid**: $[M+H]^+$ 1489.7.

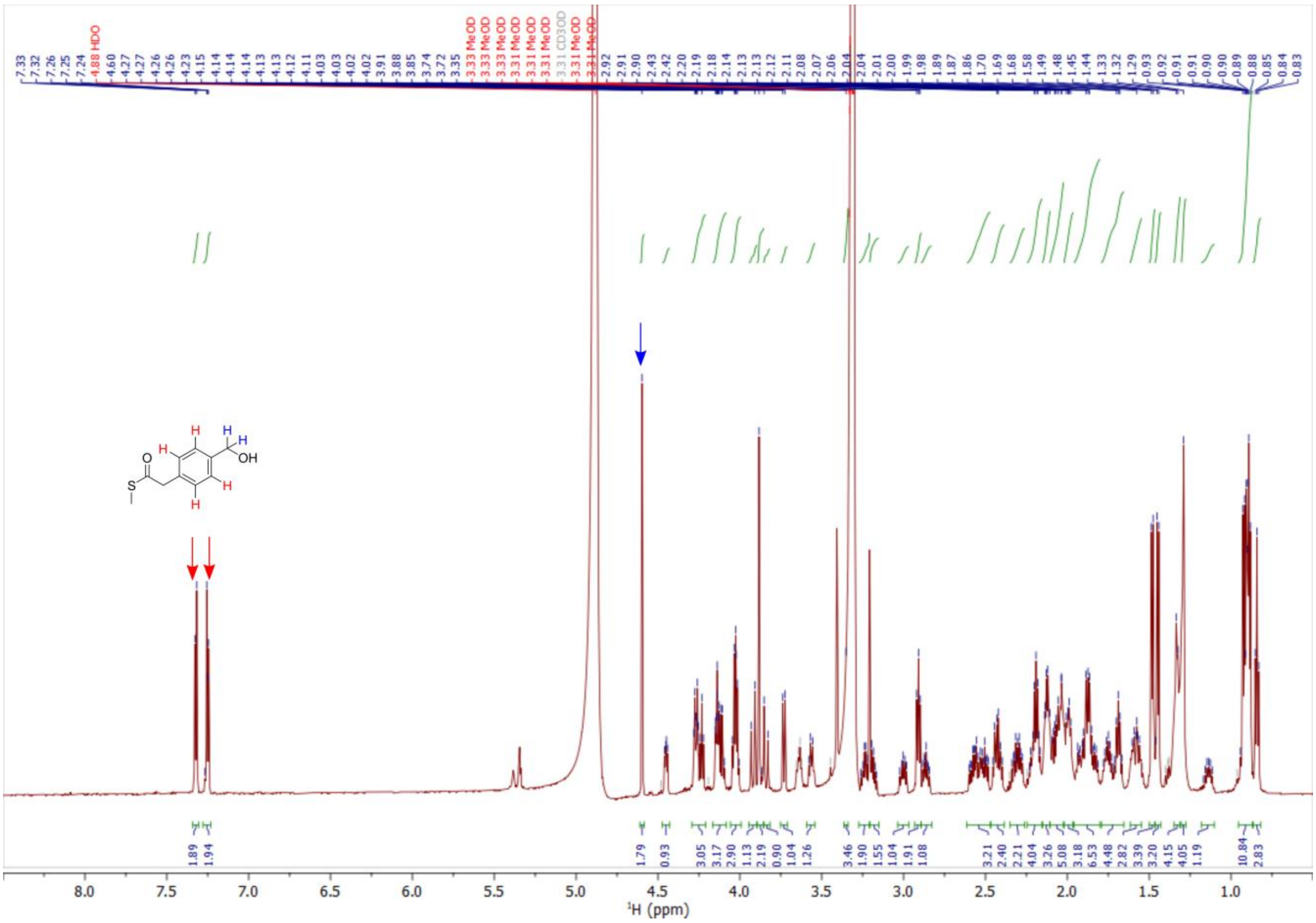

Figure S32: ^{1}H-NMR (700 MHz, MeOH-d_4, 295 K) of **P1A13-Alcohol**. Hydrogen-deuterium exchange was performed prior to the measurement.

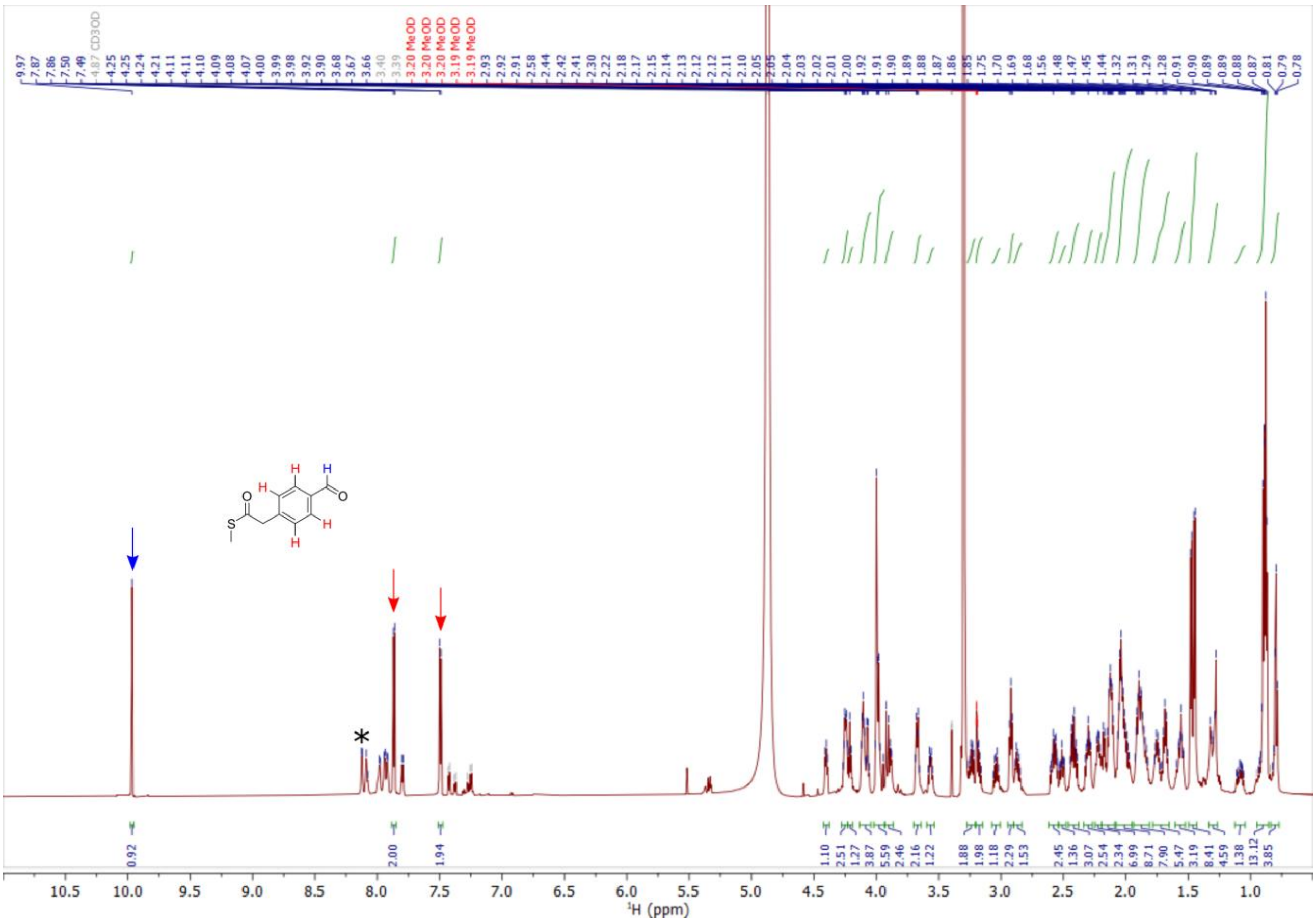

Figure S33: ^{1}H-NMR (700 MHz, MeOH-d_4, 295 K) of **P1A13**. * Hydrogen-deuterium exchange was not complete, wherefore signals of the amide backbone protons are still visible.

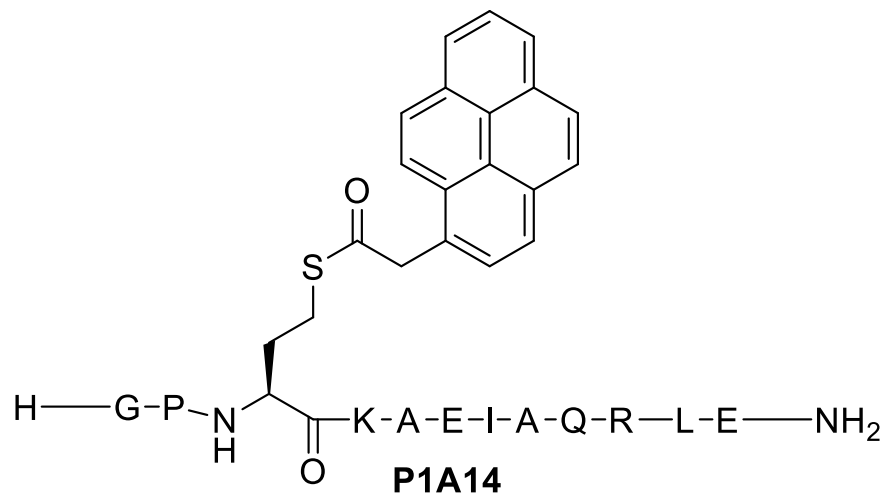

P1A14

Chemical Formula: $C_{74}H_{108}N_{18}O_{18}S$

Exact Mass: 1568.78

Molecular Weight: 1569.85

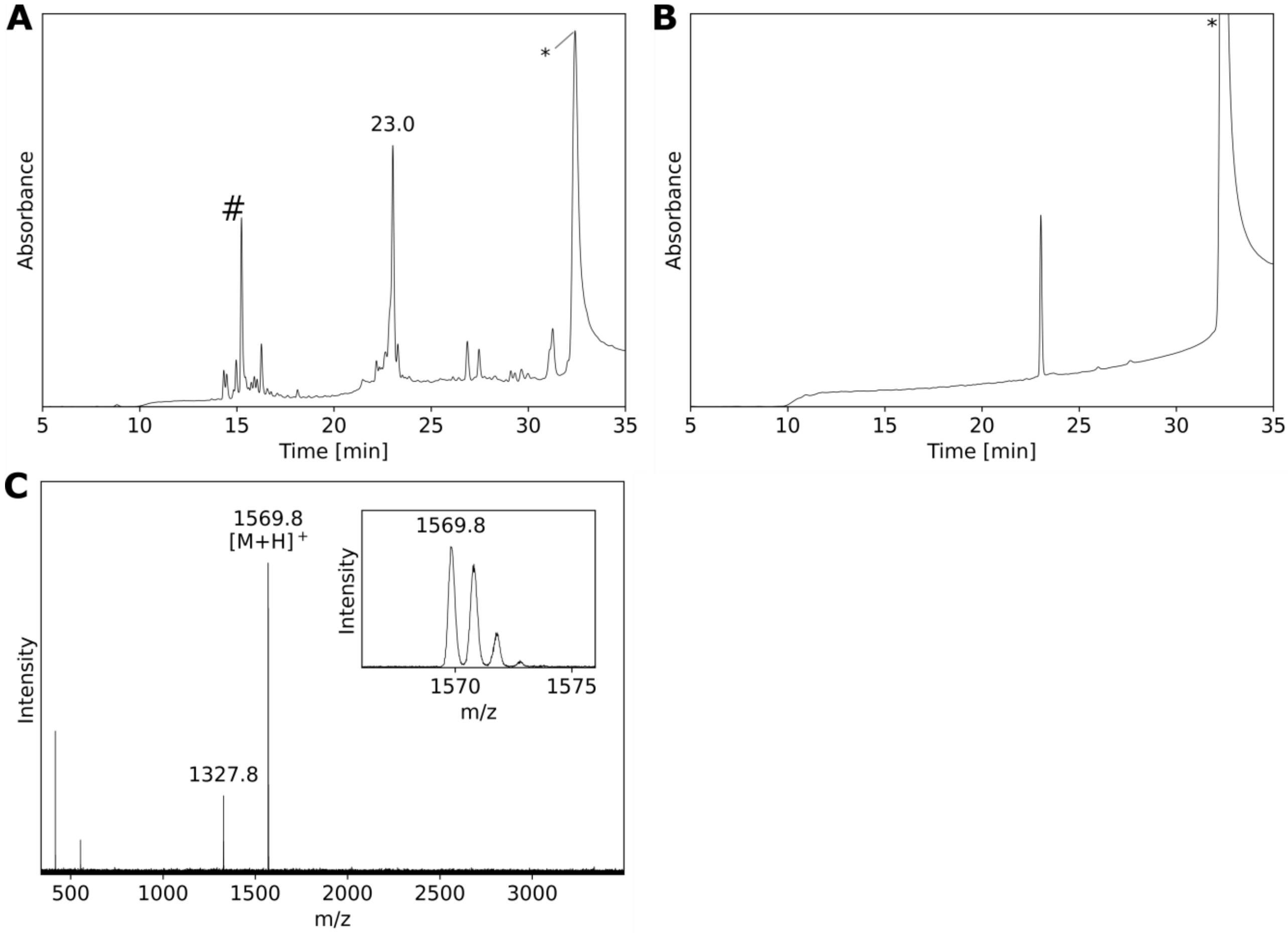

Figure S34: **P1A14** A) Analytical HPLC (220 nm) of crude peptide. HPLC Gradients are listed in Section 2.3.5 Table S3. # Peptide without homoserine. *Carryover peak. B) Analytical HPLC (220 nm) of pure peptide. C) MALDI-TOF MS of pure peptide. Calculated mass: $[M+H]^+$ 1569.8.

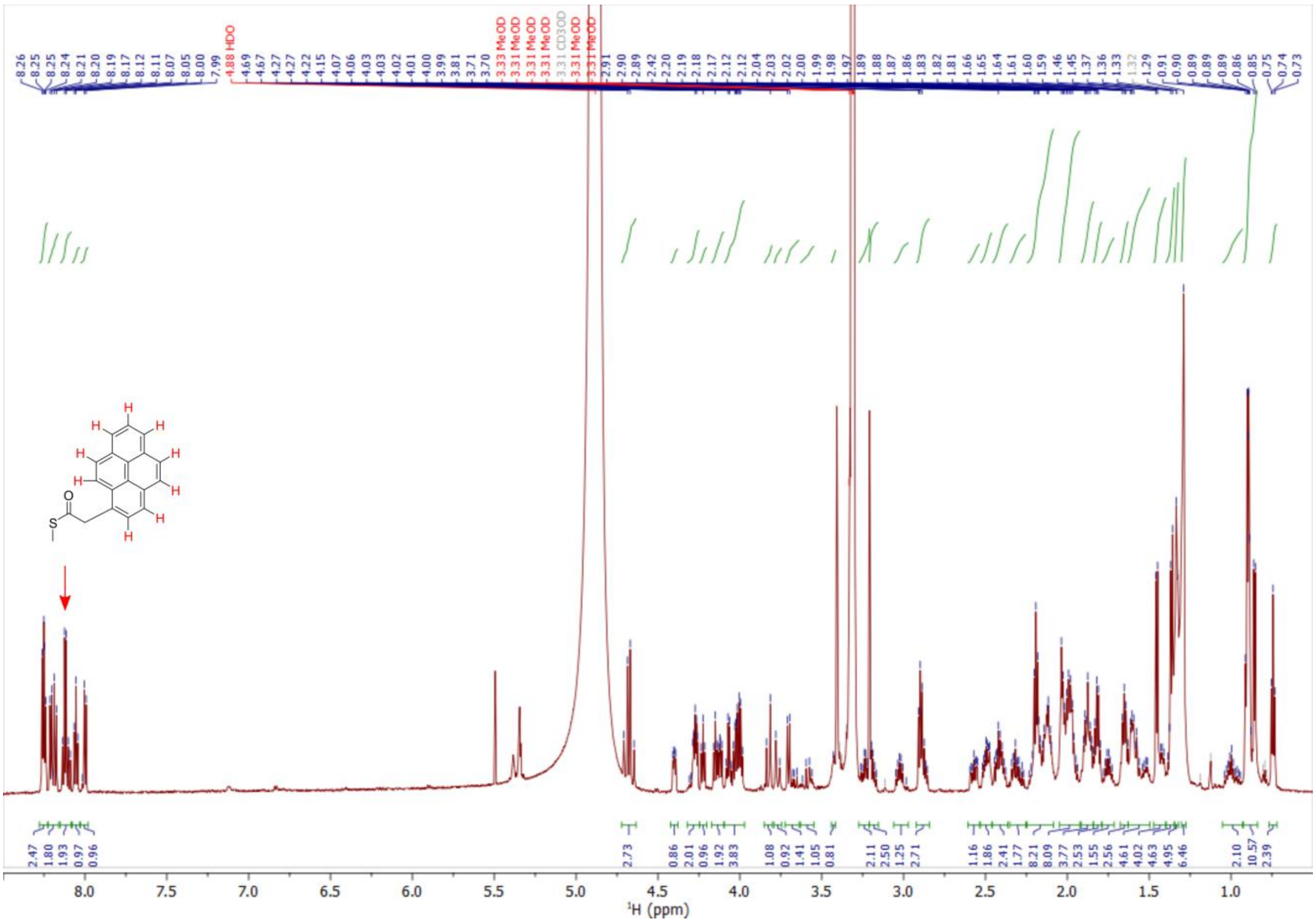

Figure S35: ^{1}H-NMR (700 MHz, MeOH-d_4, 295 K) of **P1A14**. Hydrogen-deuterium exchange was performed prior to the measurement.

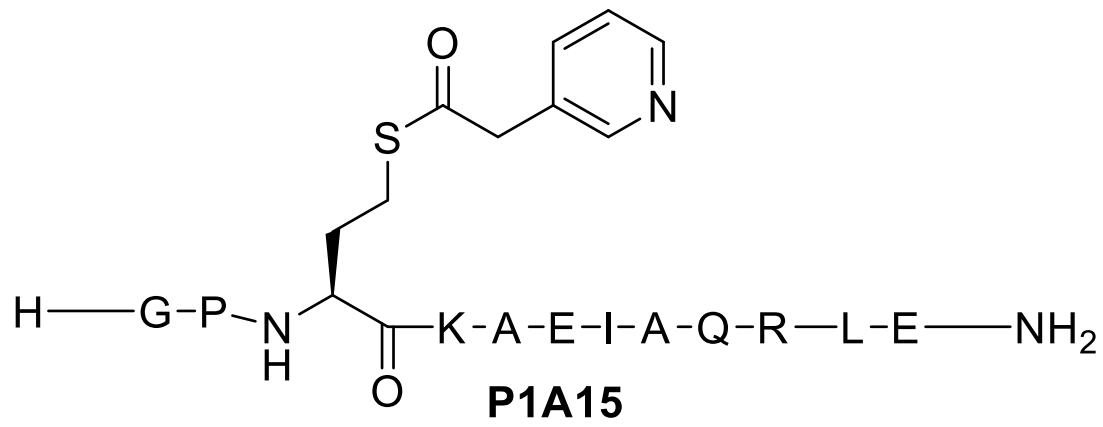

P1A15

Chemical Formula: $C_{63}H_{103}N_{19}O_{18}S$
Exact Mass: 1445.74
Molecular Weight: 1446.69

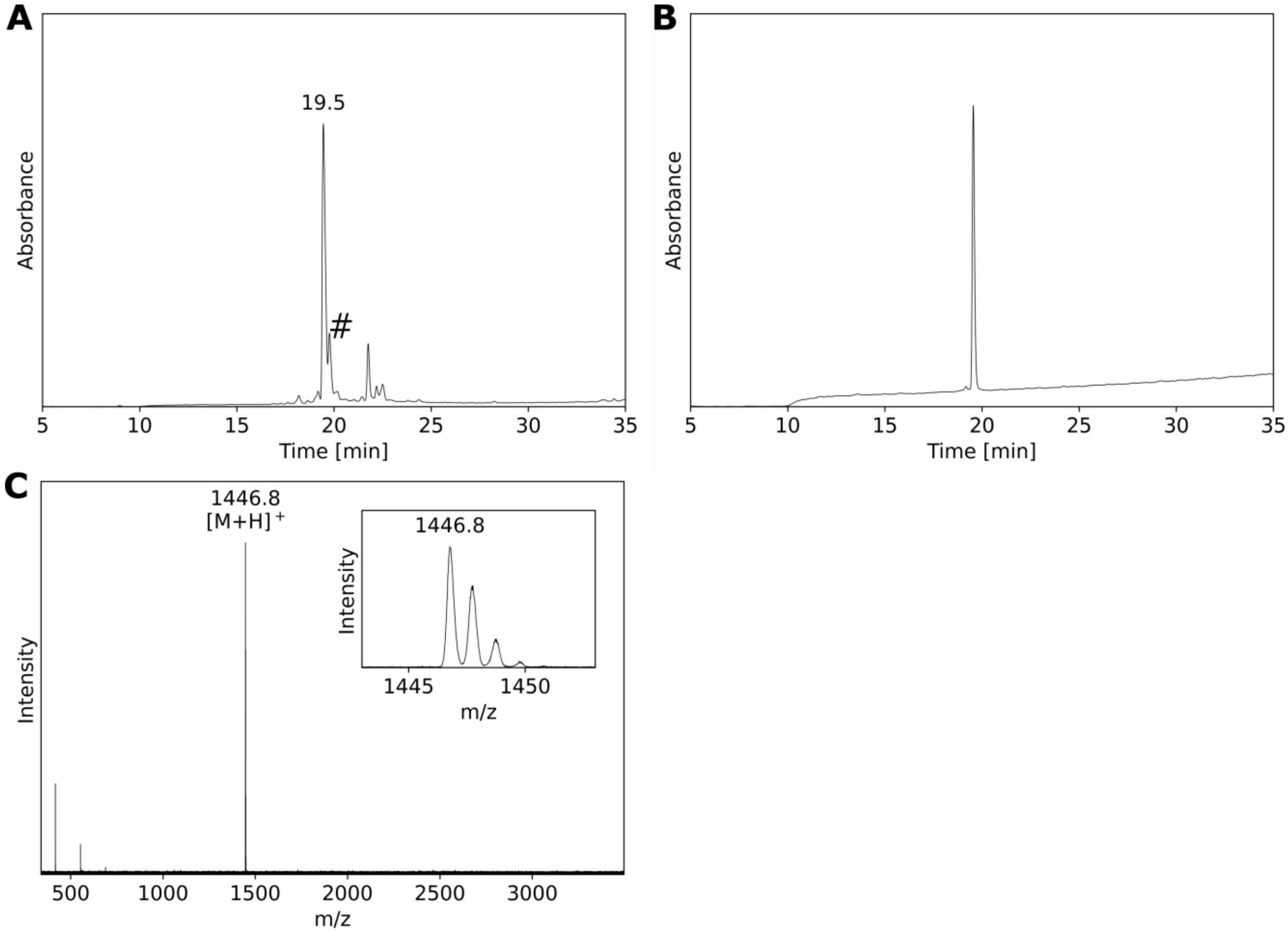

Figure S36: **P1A15** A) Analytical HPLC (220 nm) of crude peptide. HPLC Gradients are listed in Section 2.3.5 Table S3. # Peptide without homoserine. B) Analytical HPLC (220 nm) of pure peptide. C) MALDI-TOF MS of pure peptide. Calculated mass: $[M+H]^+$ 1446.8.

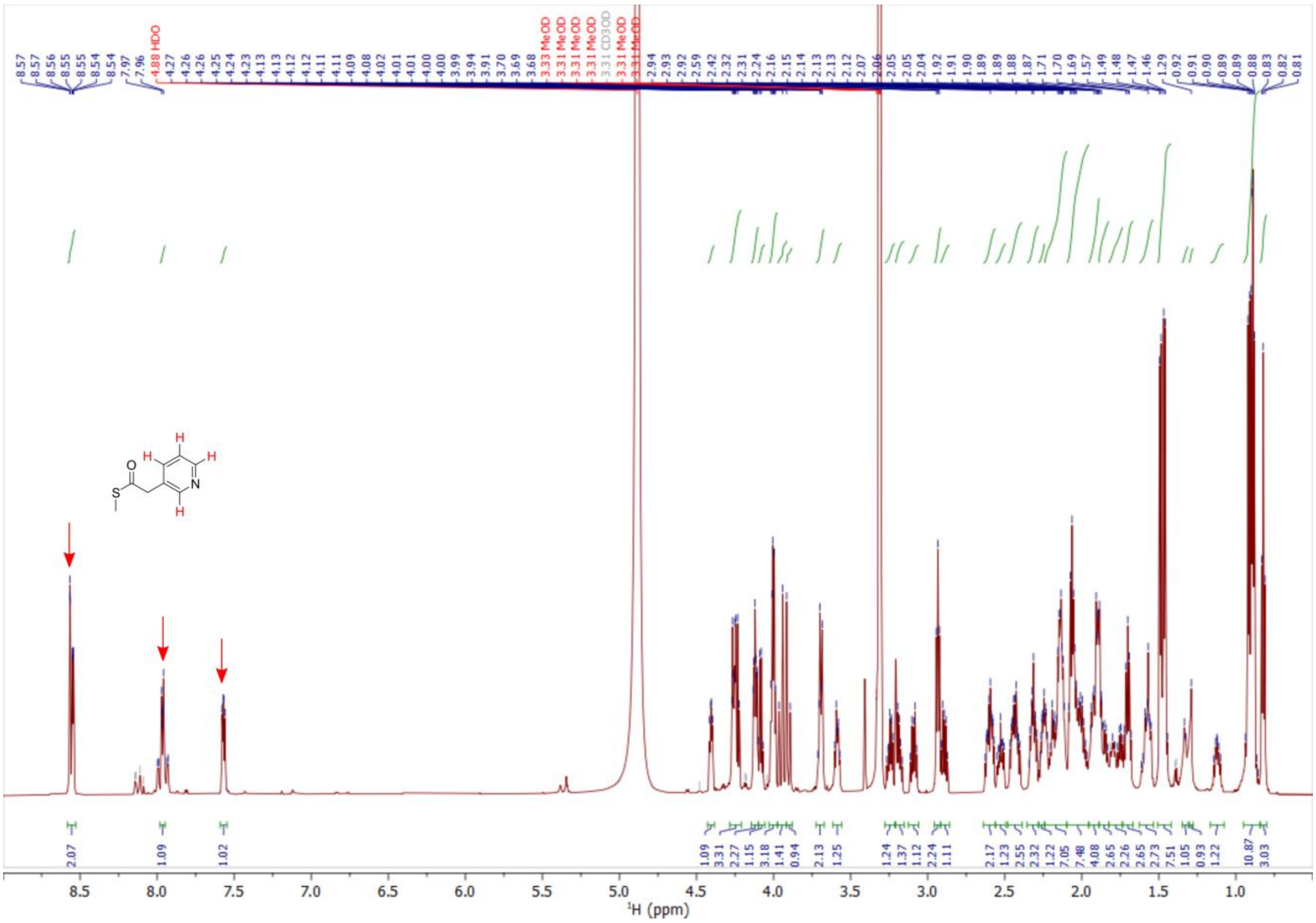

Figure S37: ^{1}H-NMR (700 MHz, MeOH-d_4, 295 K) of **P1A15**. Hydrogen-deuterium exchange was performed prior to the measurement.

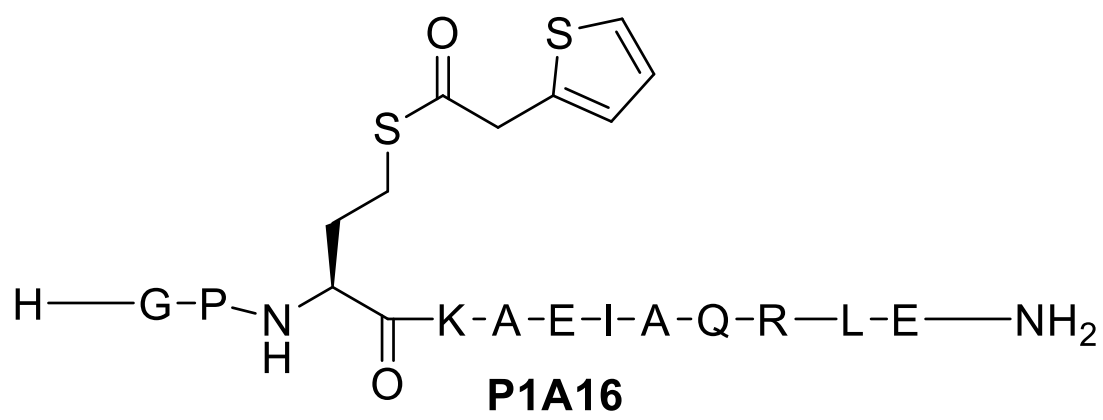

P1A16

Chemical Formula: $C_{62}H_{102}N_{18}O_{18}S_2$

Exact Mass: 1450.71

Molecular Weight: 1451.73

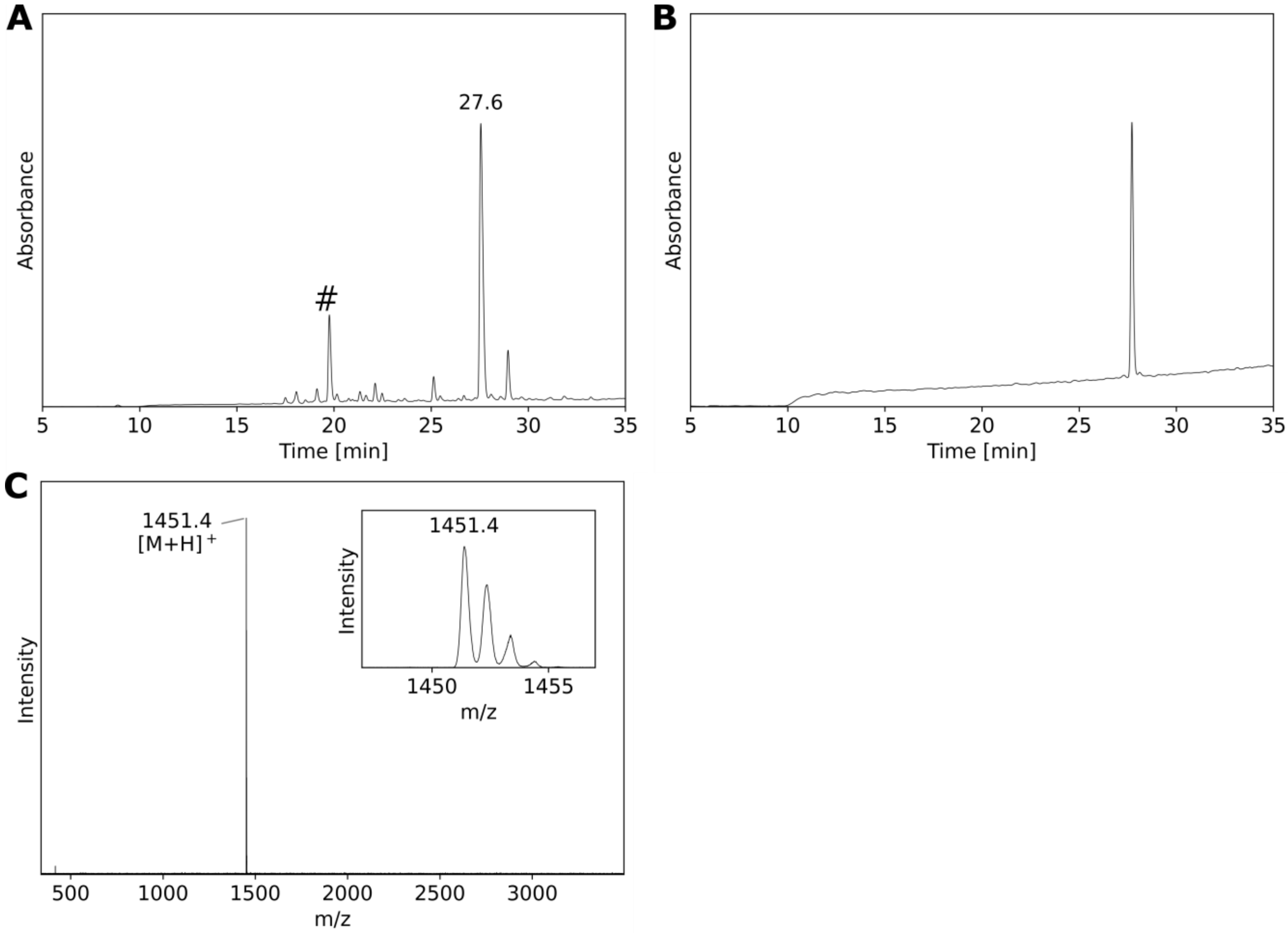

Figure S38: **P1A16** A) Analytical HPLC (220 nm) of crude peptide. HPLC Gradients are listed in Section 2.3.5 Table S3. # Peptide without homoserine. B) Analytical HPLC (220 nm) of pure peptide. C) MALDI-TOF MS of pure peptide. Calculated mass: $[M+H]^+$ 1451.7.

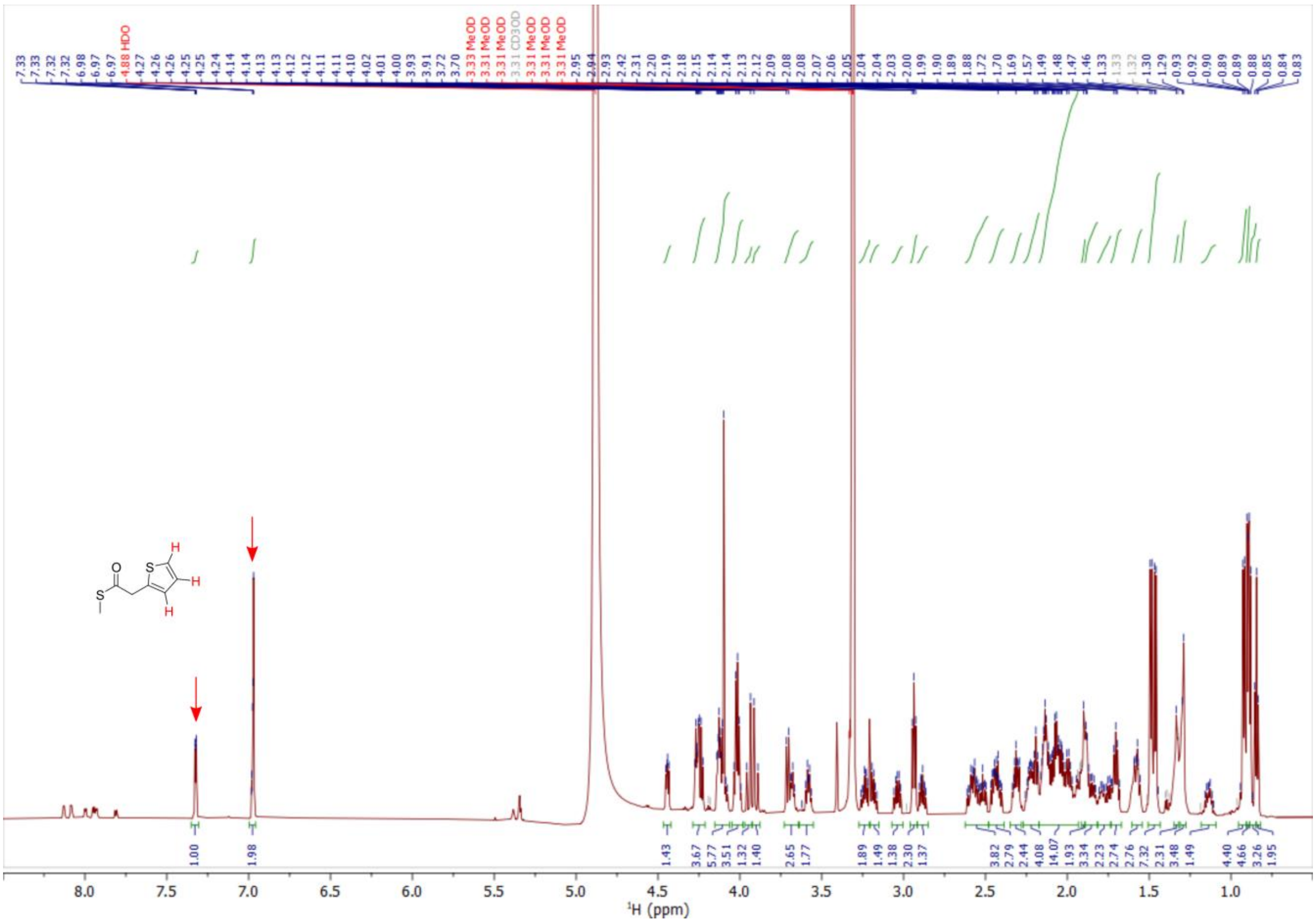

Figure S39: ^{1}H-NMR (700 MHz, MeOH-d_4, 295 K) of **P1A16**. Hydrogen-deuterium exchange was performed prior to the measurement.

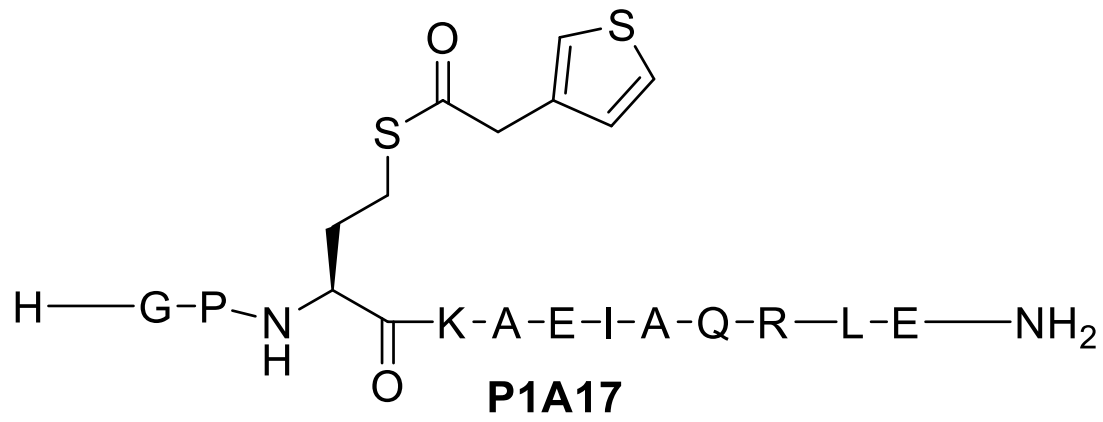

P1A17

Chemical Formula: $C_{62}H_{102}N_{18}O_{18}S_2$
Exact Mass: 1450.71
Molecular Weight: 1451.73

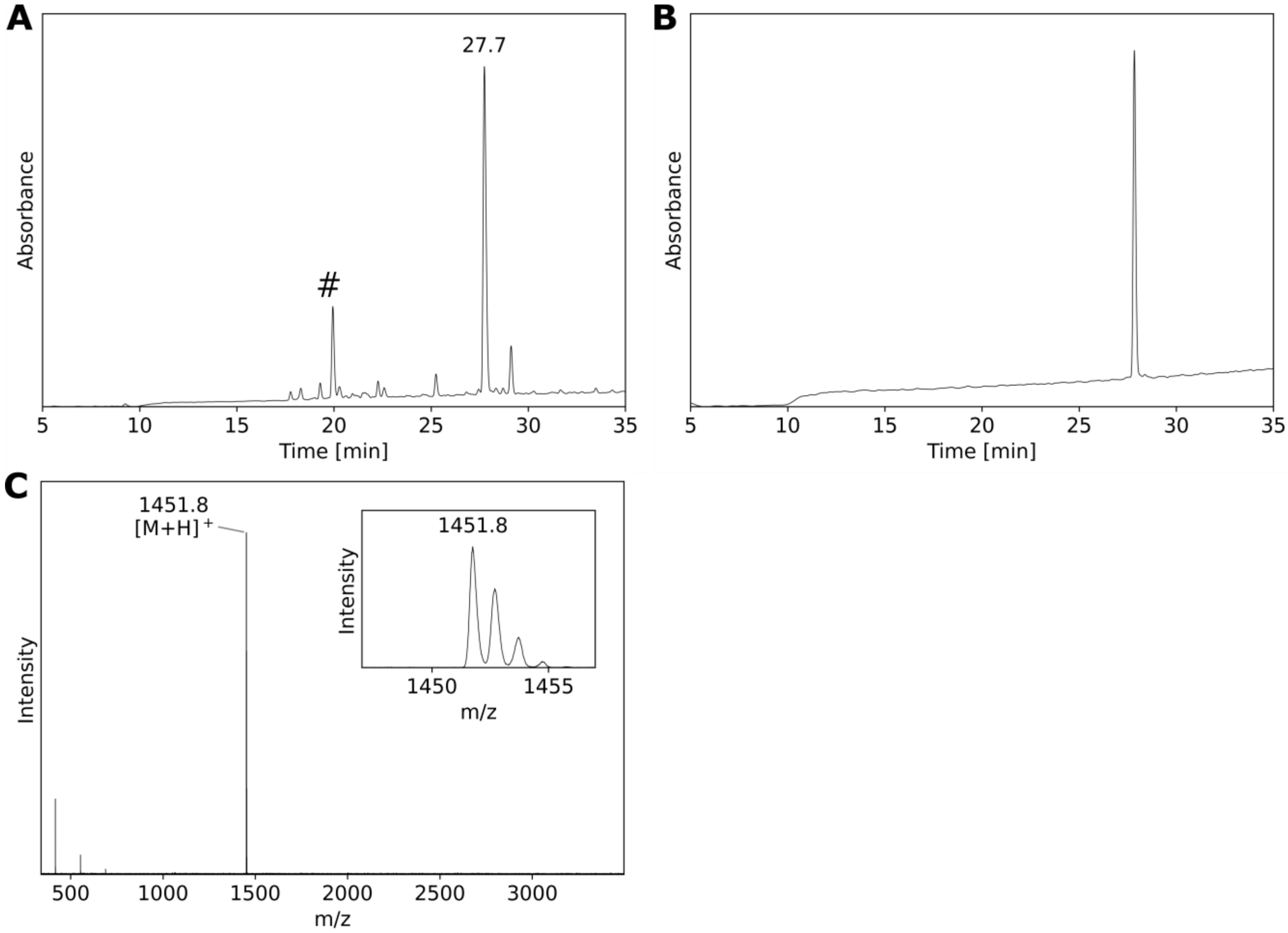

Figure S40: **P1A17** A) Analytical HPLC (220 nm) of crude peptide. HPLC Gradients are listed in Section 2.3.5 Table S3. # Peptide without homoserine. B) Analytical HPLC (220 nm) of pure peptide. C) MALDI-TOF MS of pure peptide. Calculated mass: $[M+H]^+$ 1451.7.

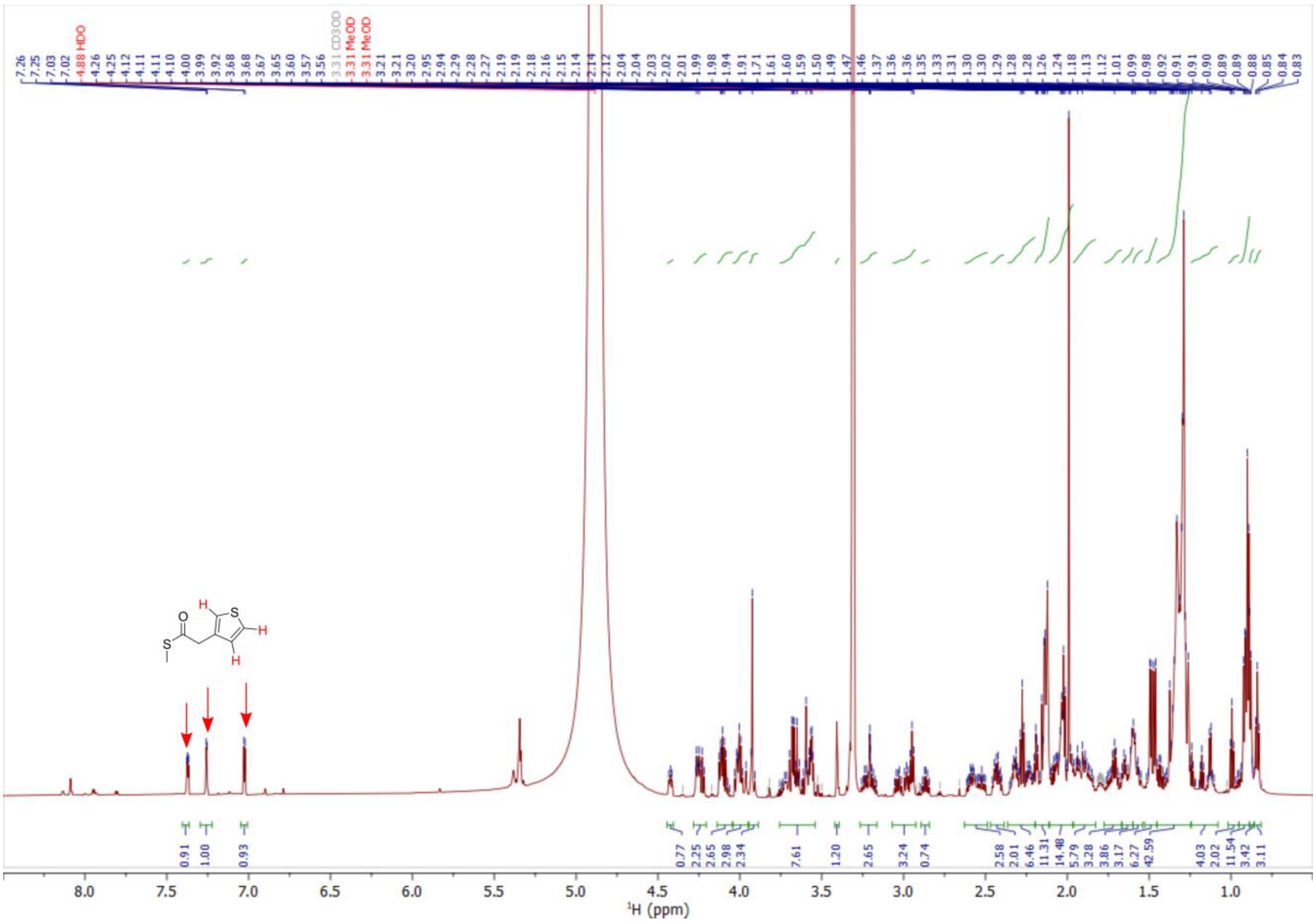

Figure S41: ^{1}H-NMR (700 MHz, MeOH-d_4, 295 K) of **P1A17**. Hydrogen-deuterium exchange was performed prior to the measurement.

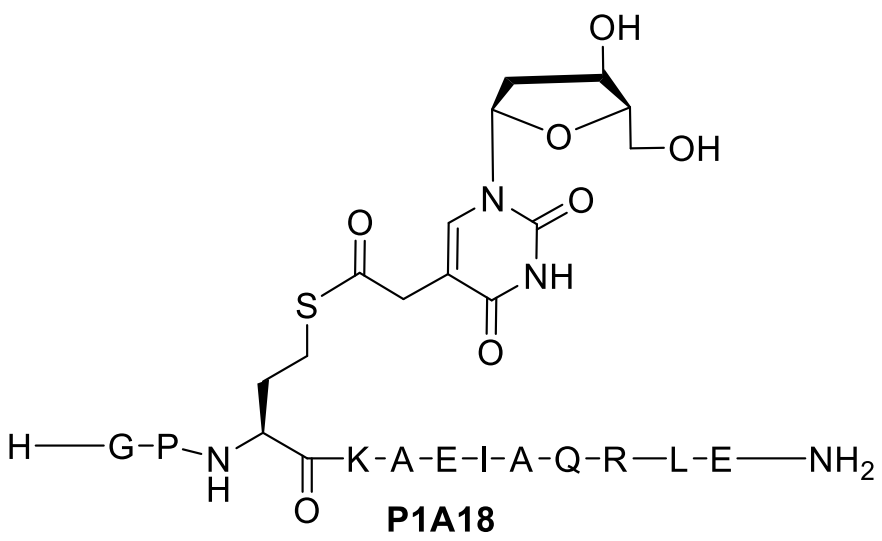

P1A18
Chemical Formula: $C_{67}H_{110}N_{20}O_{23}S$
Exact Mass: 1594.78
Molecular Weight: 1595.79

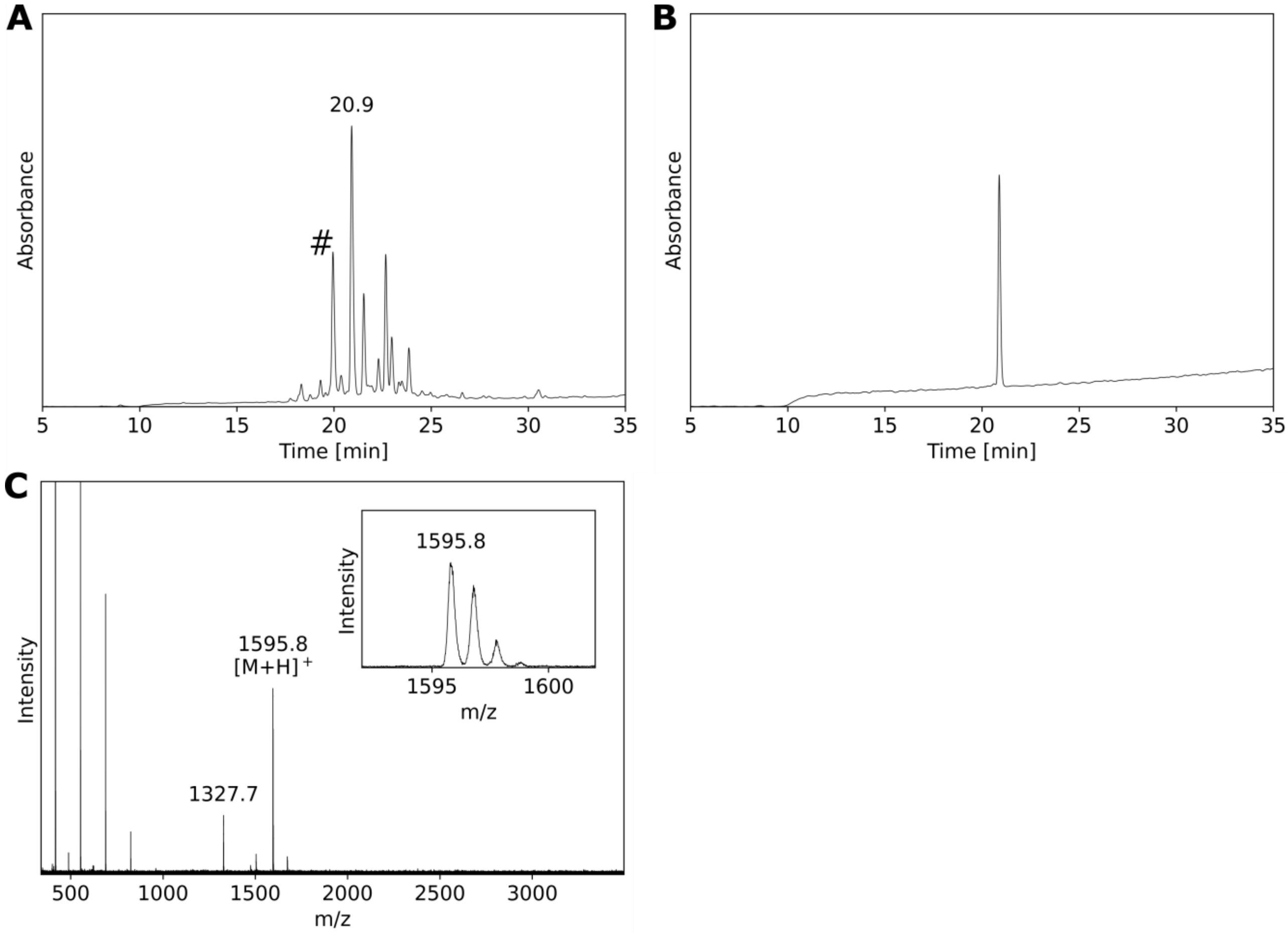

Figure S42: **P1A18** A) Analytical HPLC (220 nm) of crude peptide. HPLC Gradients are listed in Section 2.3.5 Table S3. # Peptide without homoserine. B) Analytical HPLC (220 nm) of pure peptide. C) MALDI-TOF MS of pure peptide. Calculated mass: $[M+H]^+$ 1595.8.

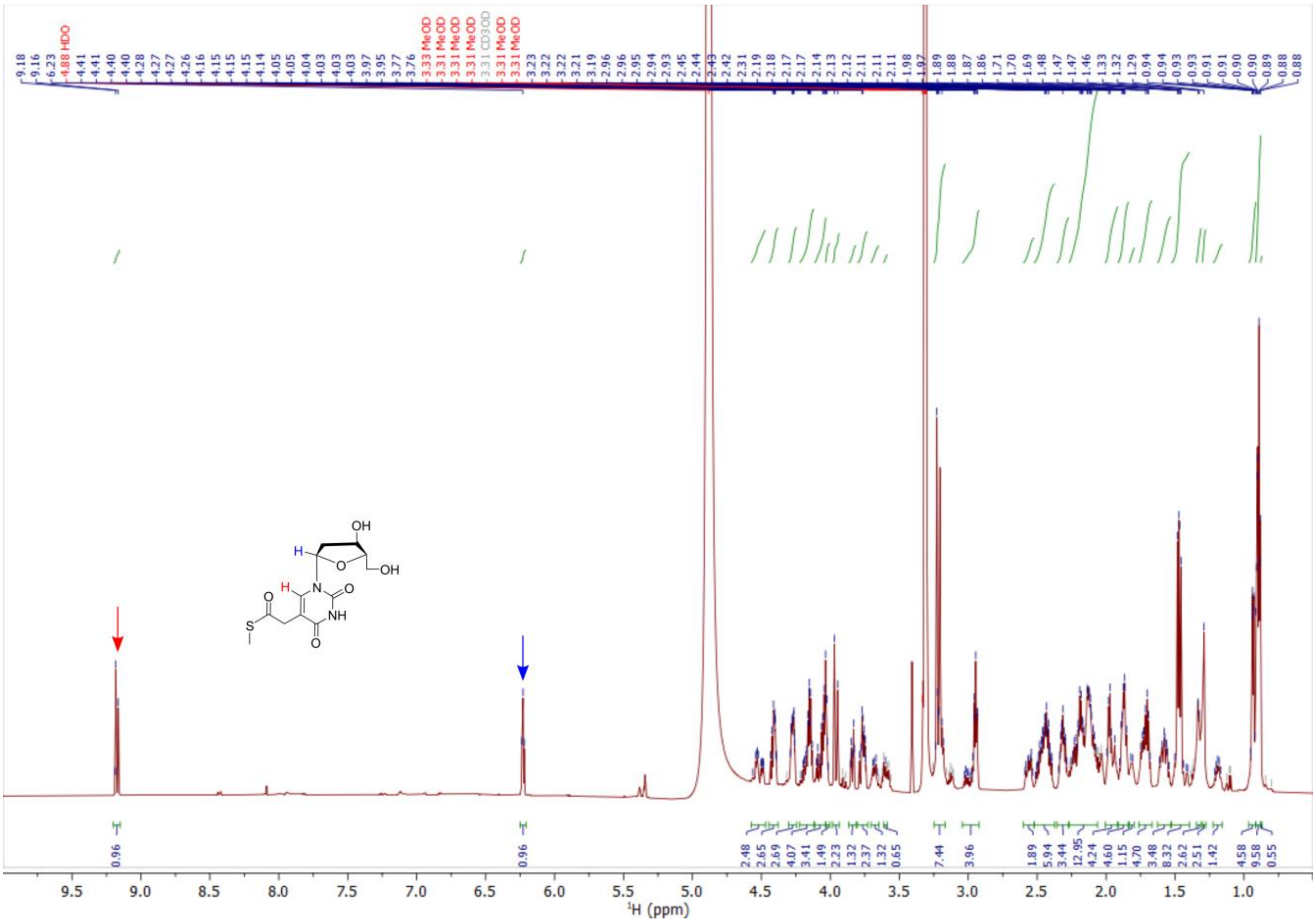

Figure S43: ¹H-NMR (700 MHz, MeOH-d_4, 295 K) of **P1A18**. Hydrogen-deuterium exchange was performed prior to the measurement.

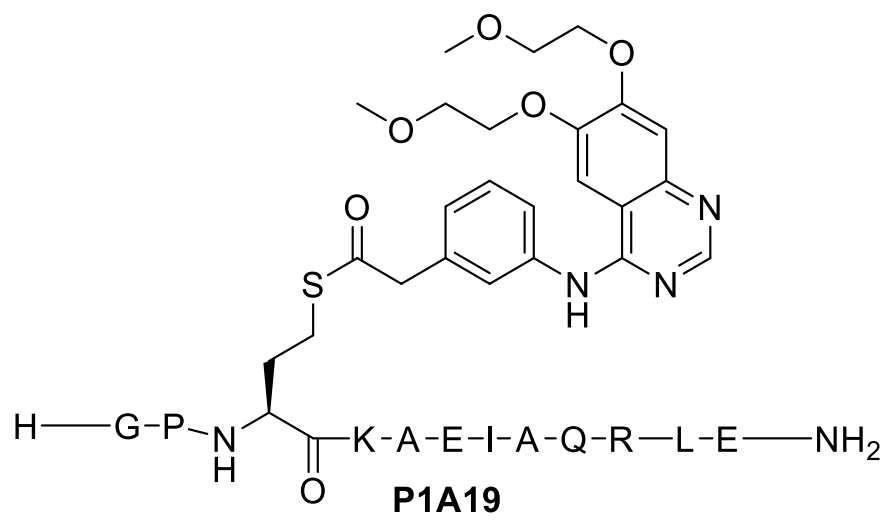

P1A19
Chemical Formula: $C_{78}H_{121}N_{21}O_{22}S$
Exact Mass: 1735.87
Molecular Weight: 1737.01

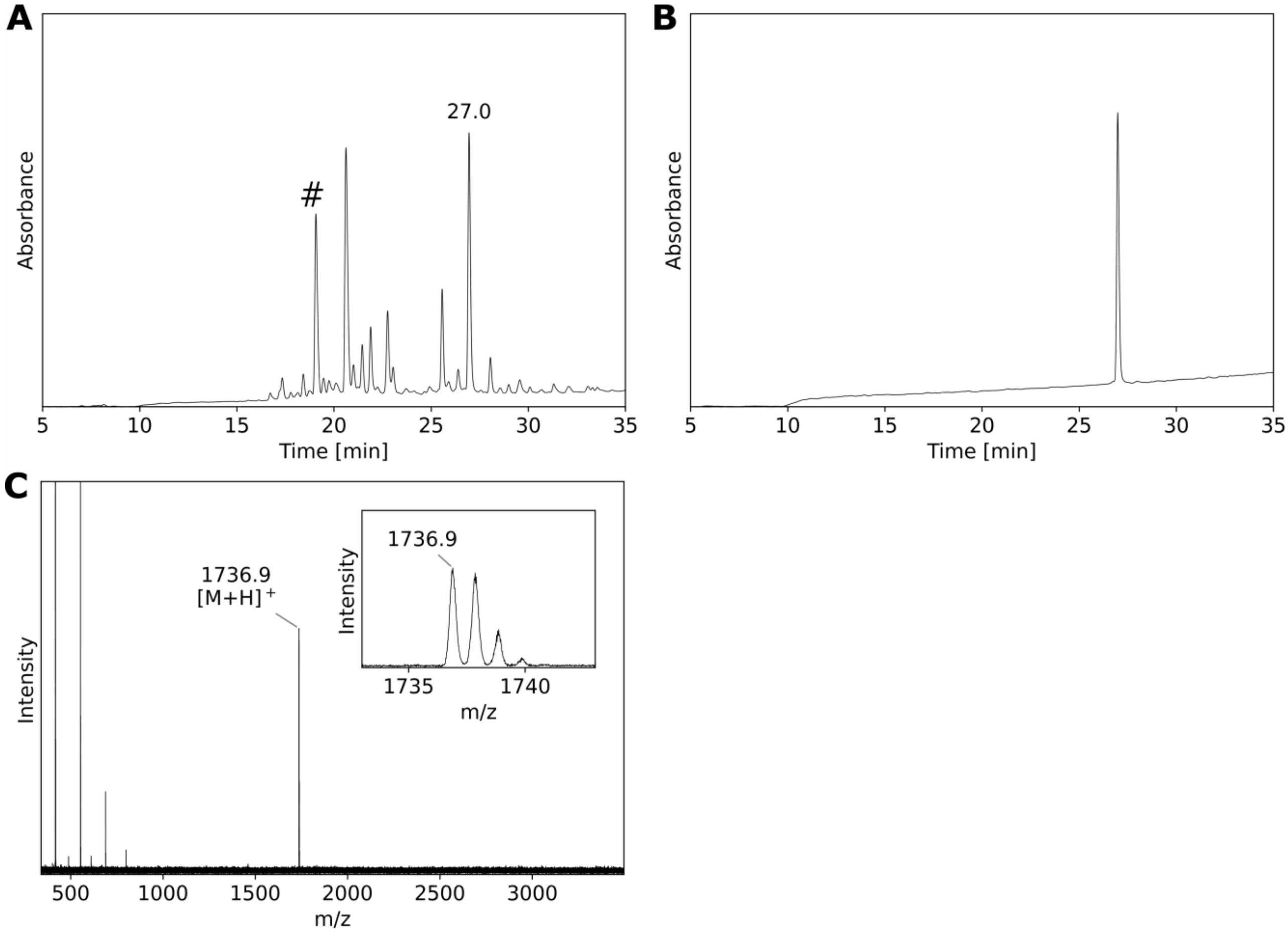

Figure S44: **P1A19** A) Analytical HPLC (220 nm) of crude peptide. HPLC Gradients are listed in Section 2.3.5 Table S3. # Peptide without homoserine. B) Analytical HPLC (220 nm) of pure peptide. C) MALDI-TOF MS of pure peptide. Calculated mass: $[M+H]^+$ 1736.9.

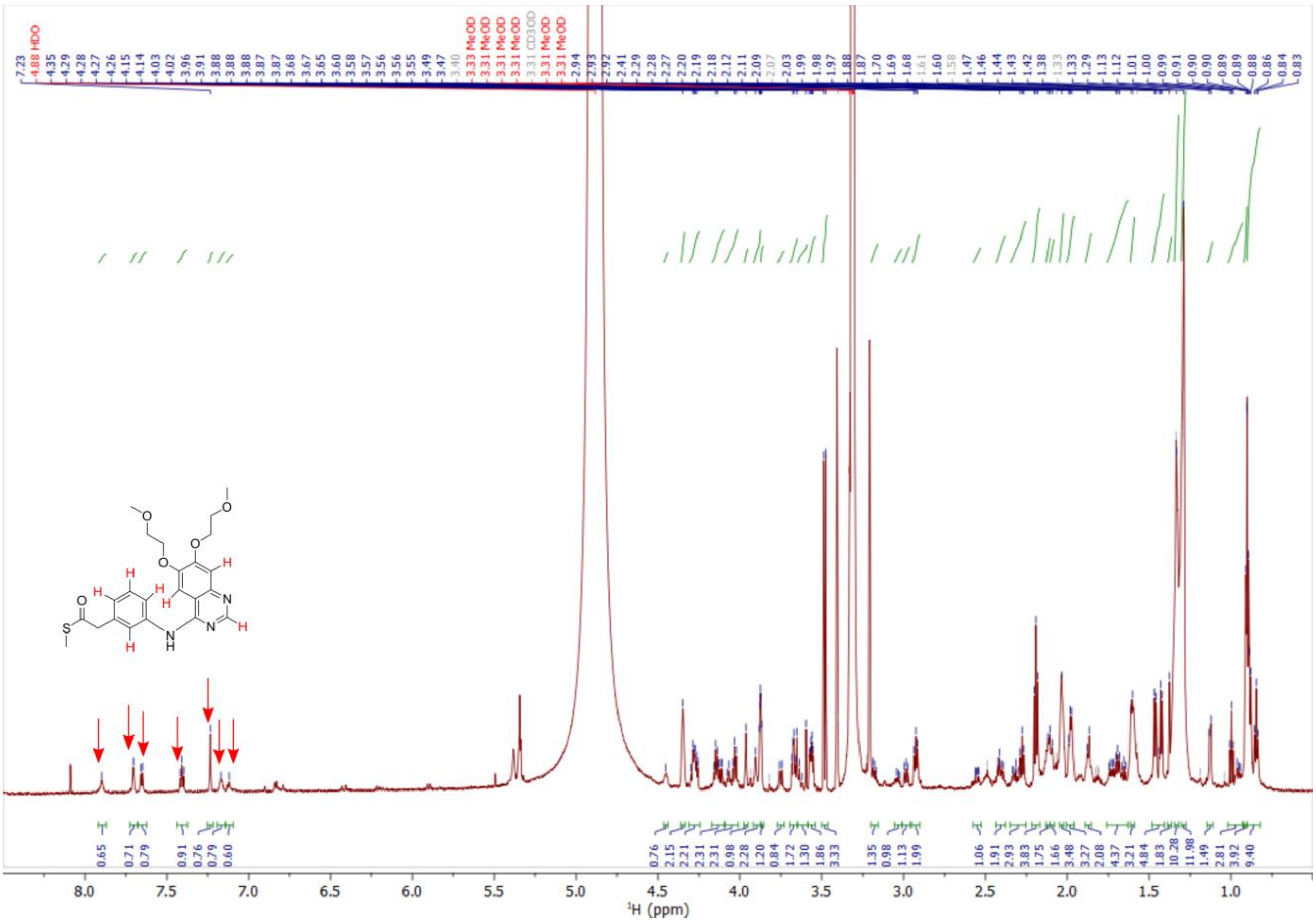

Figure S45: ^{1}H-NMR (700 MHz, MeOH-d_4, 295 K) of **P1A19**. Hydrogen-deuterium exchange was performed prior to the measurement.

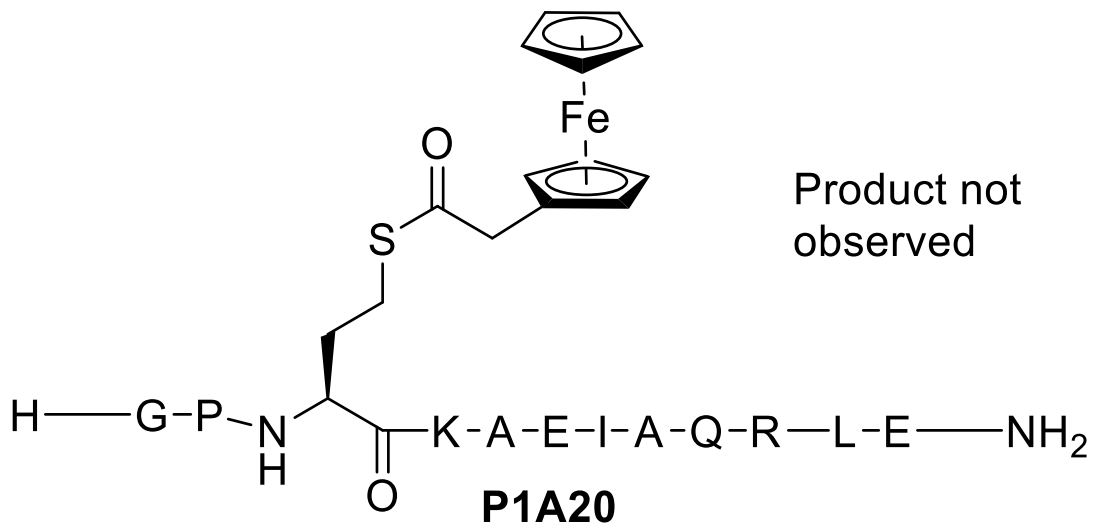

Product not observed

P1A20

Chemical Formula: $C_{68}H_{108}FeN_{18}O_{18}S$
Exact Mass: 1552.72
Molecular Weight: 1553.63

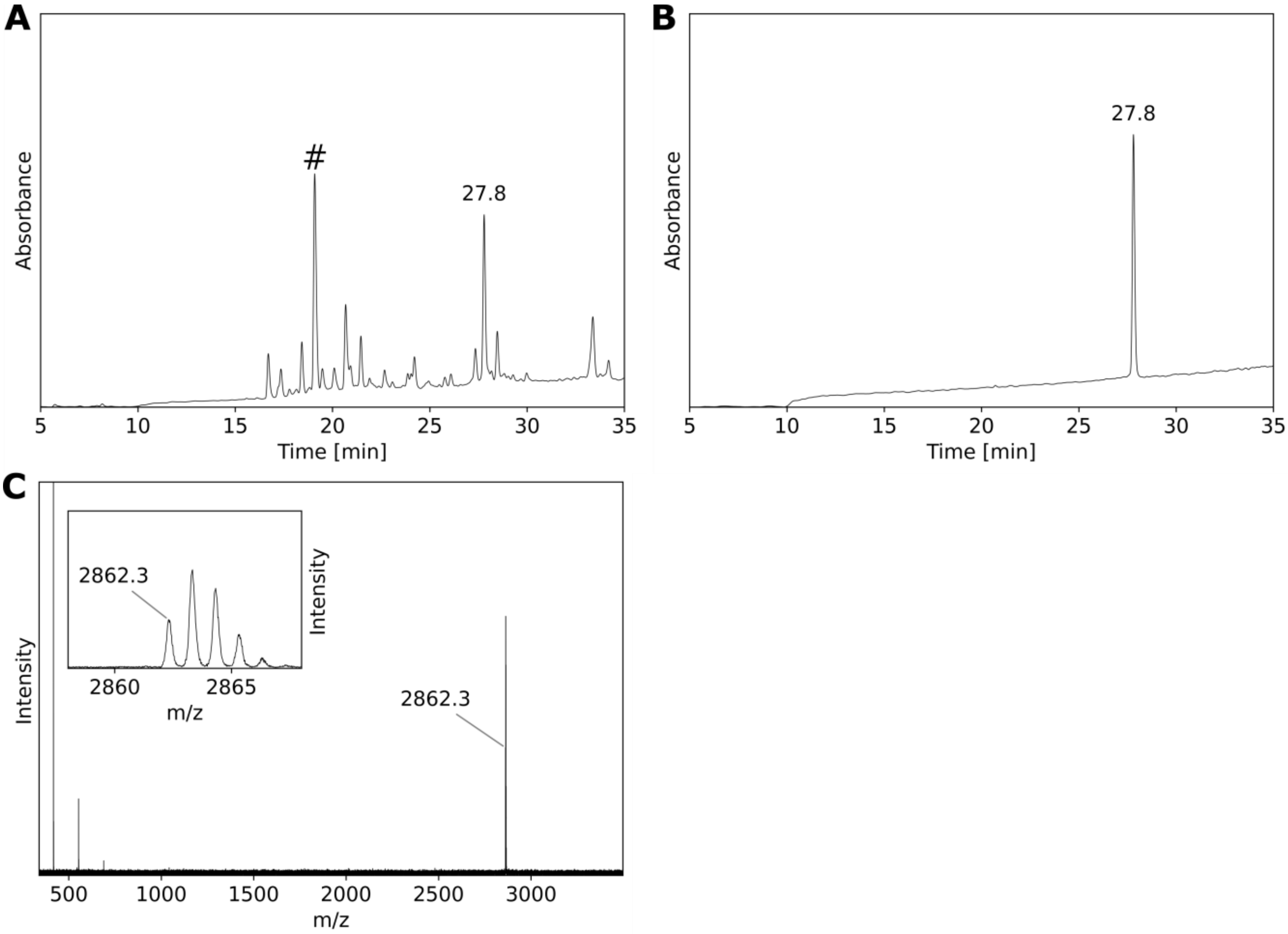

Figure S46: **P1A20** A) Analytical HPLC (220 nm) of crude peptide. The thioester **P1A20** was not observed. HPLC Gradients are listed in Section 2.3.5 Table S3. # Peptide without homoserine. B) Analytical HPLC (220 nm) of purified peptide with a retention time of 27.8 min. C) MALDI-TOF MS of purified peptide with a retention time of 27.8 min.

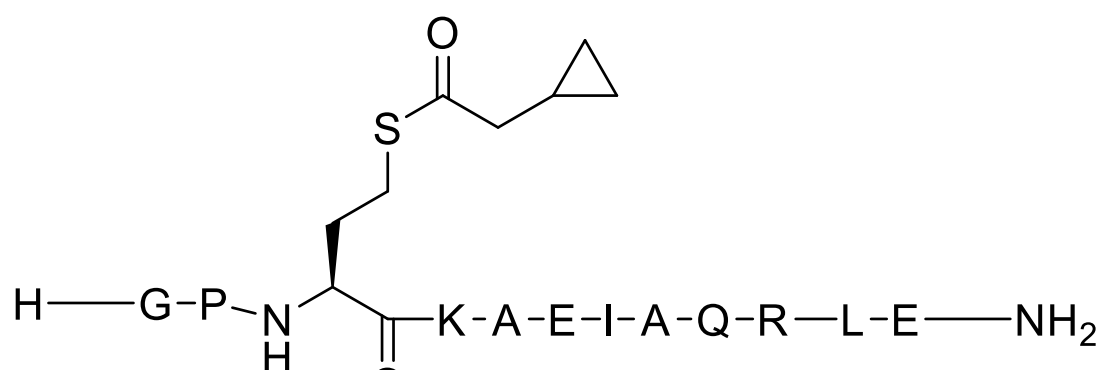

P1A22

Chemical Formula: $C_{61}H_{104}N_{18}O_{18}S$

Exact Mass: 1408.75

Molecular Weight: 1409.67

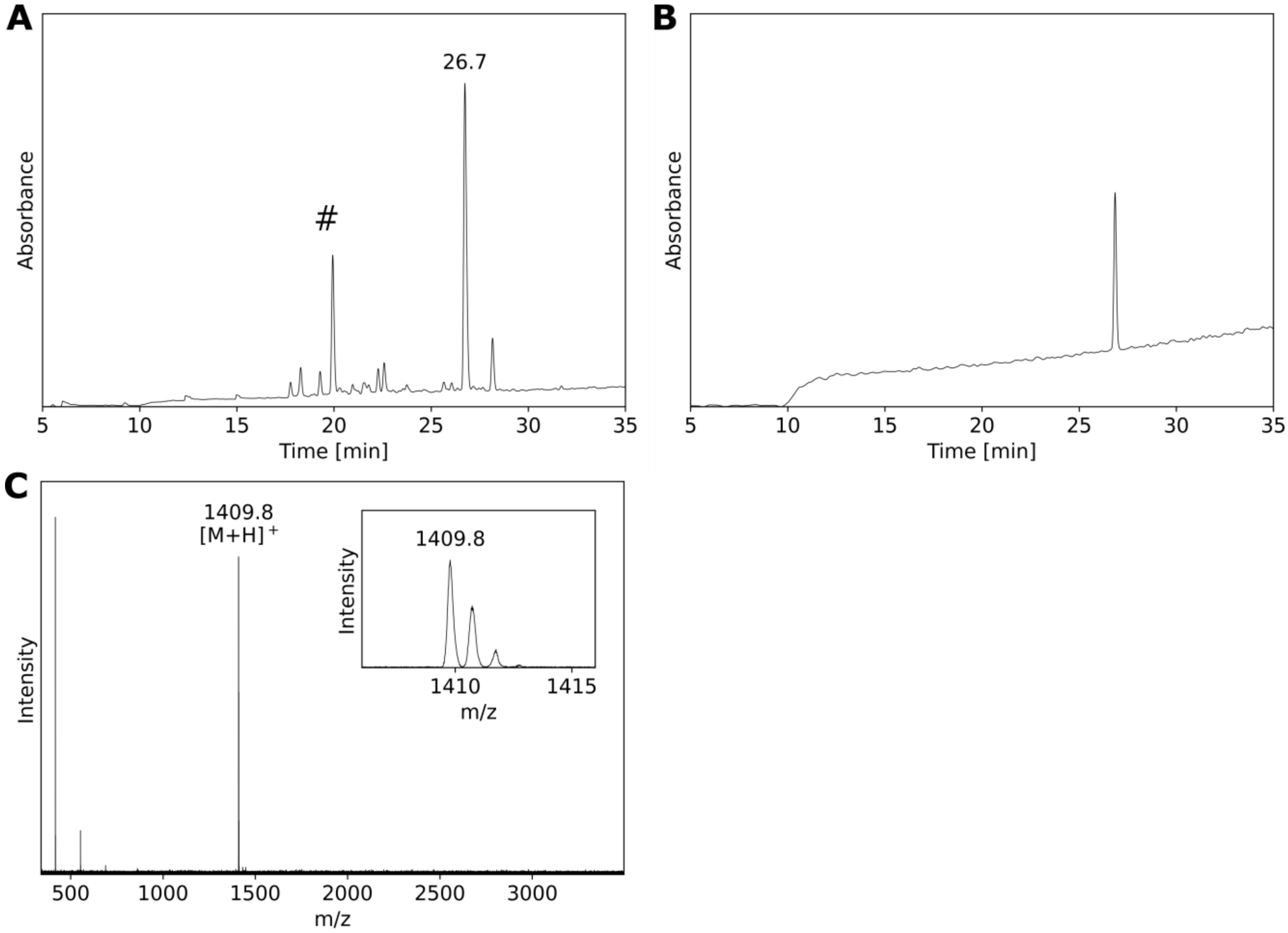

Figure S47: **P1A22** A) Analytical HPLC (220 nm) of crude peptide. HPLC Gradients are listed in Section 2.3.5 Table S3. # Peptide without homoserine. B) Analytical HPLC (220 nm) of pure peptide. C) MALDI-TOF MS of pure peptide. Calculated mass: $[M+H]^+$ 1409.8.

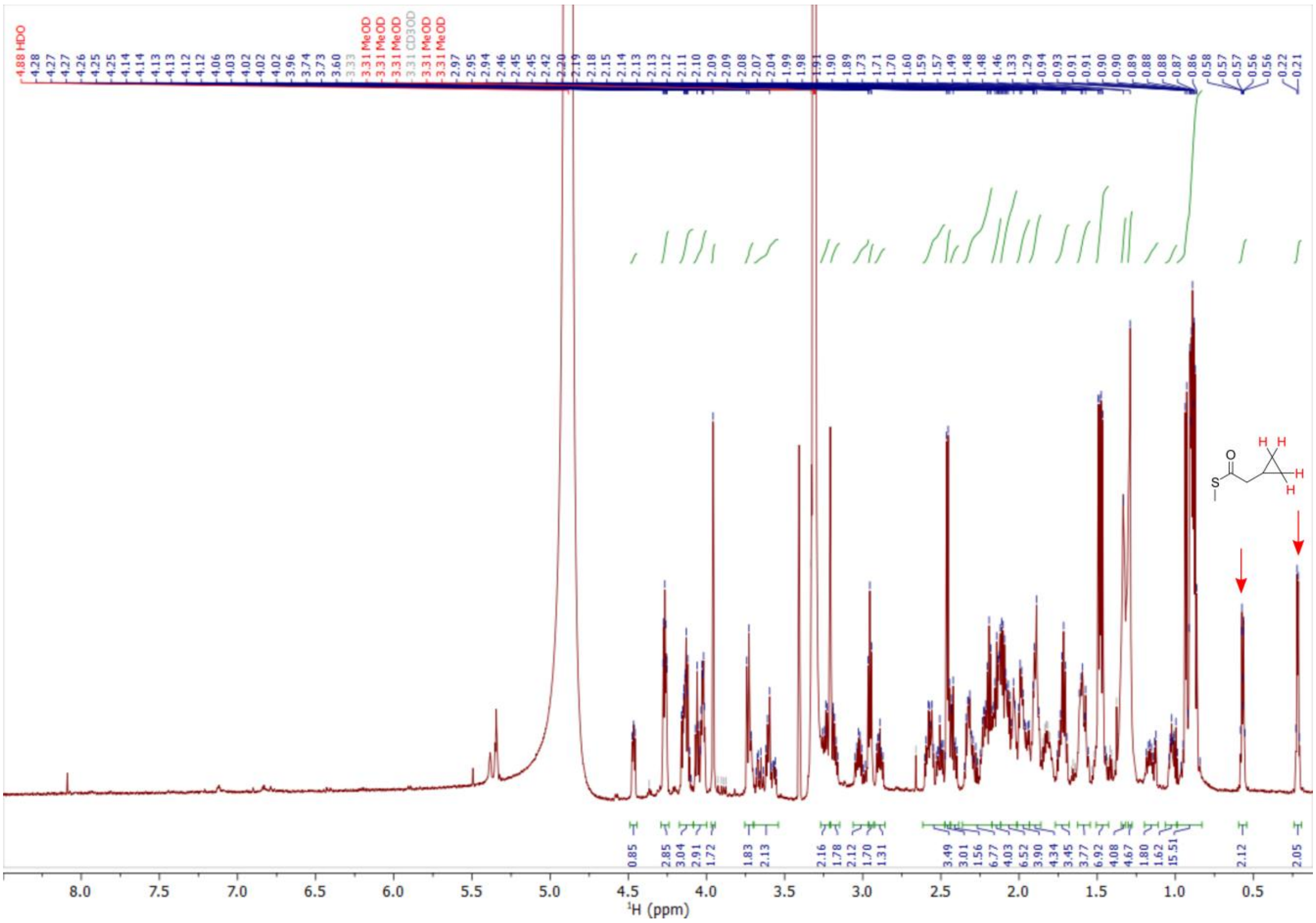

Figure S48: ^{1}H-NMR (700 MHz, MeOH-d_4, 295 K) of **P1A22**. Hydrogen-deuterium exchange was performed prior to the measurement.

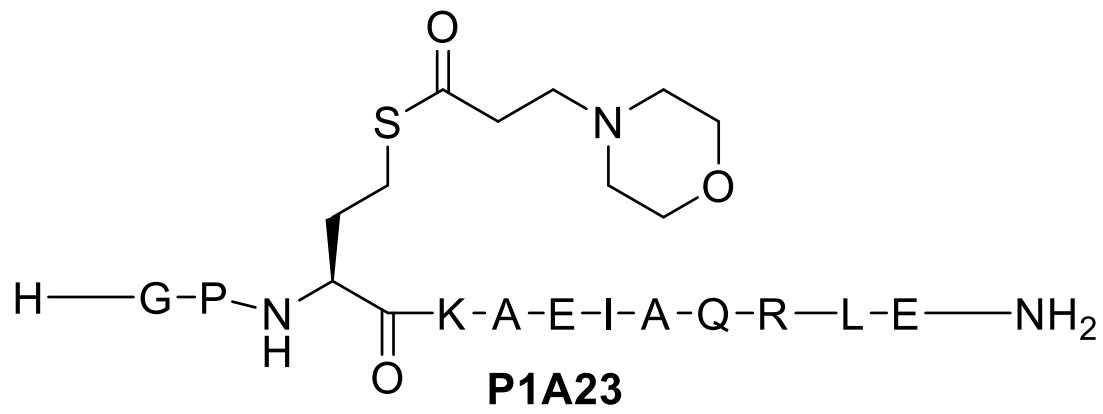

P1A23
Chemical Formula: $C_{63}H_{109}N_{19}O_{19}S$
Exact Mass: 1467.79
Molecular Weight: 1468.74

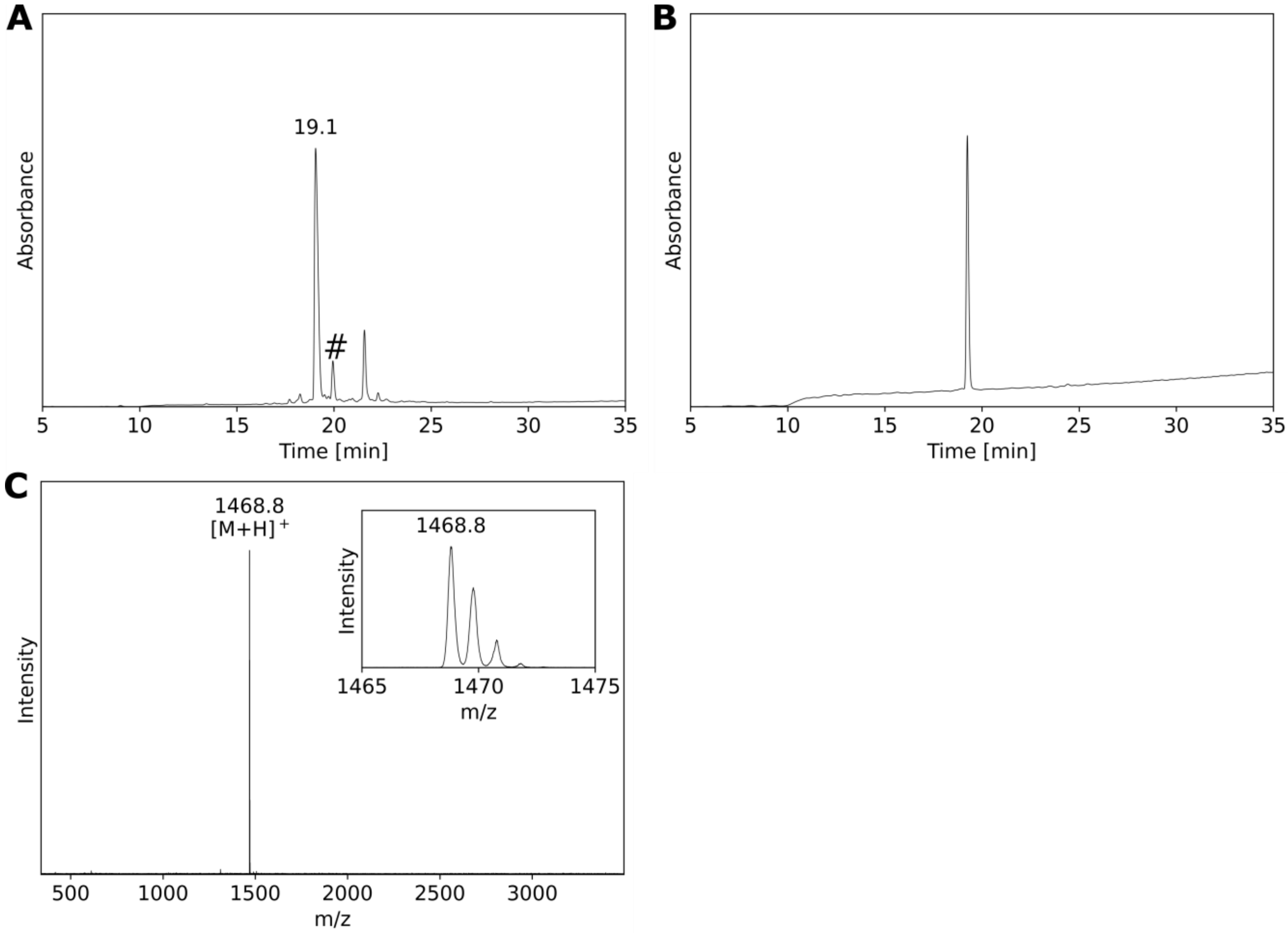

Figure S49: **P1A23** A) Analytical HPLC (220 nm) of crude peptide. HPLC Gradients are listed in Section 2.3.5 Table S3. # Peptide without homoserine. B) Analytical HPLC (220 nm) of pure peptide. C) MALDI-TOF MS of pure peptide. Calculated mass: $[M+H]^+$ 1468.8.

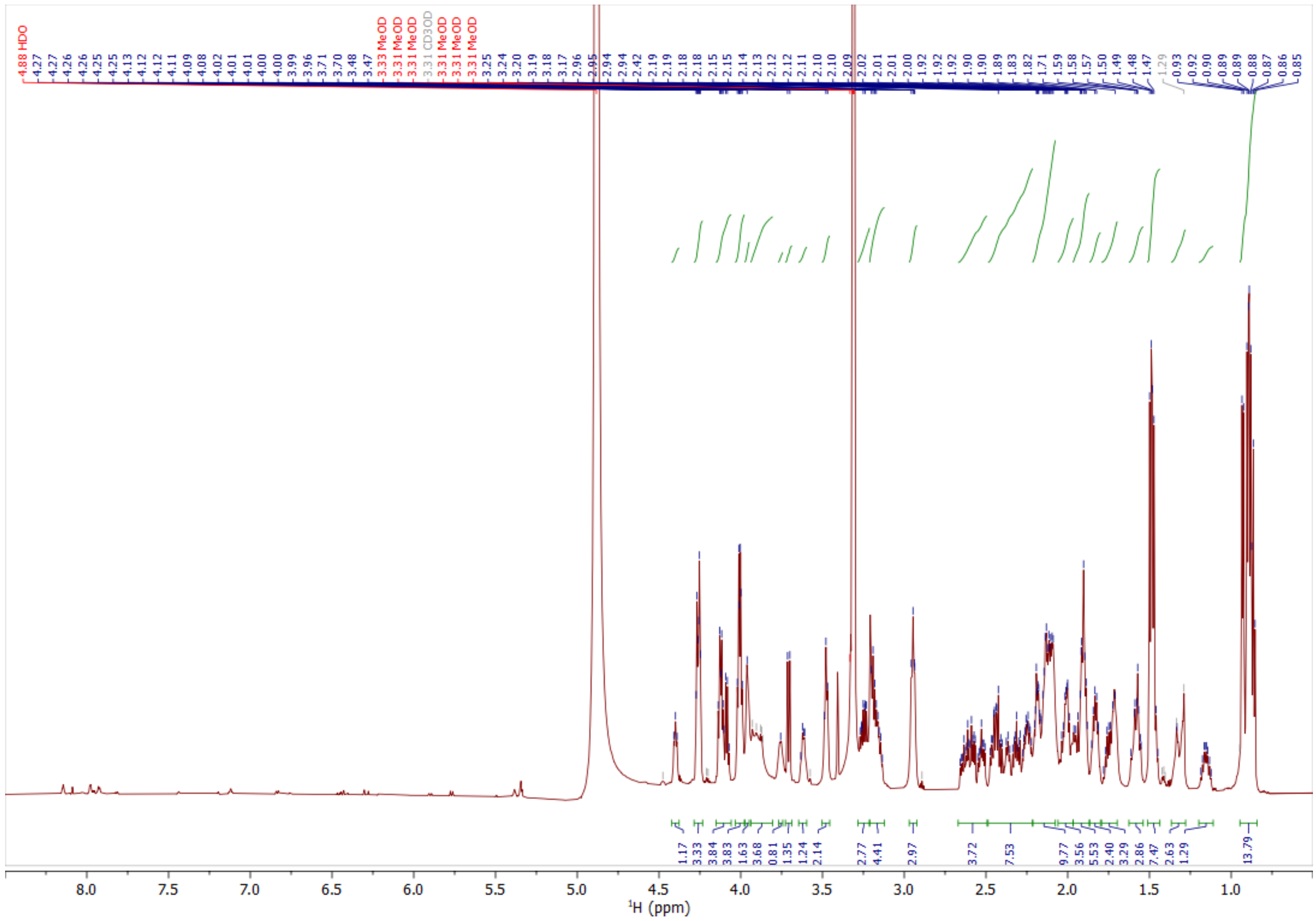

Figure S50: ^{1}H-NMR (700 MHz, MeOH-d_4, 295 K) of **P1A23**. Hydrogen-deuterium exchange was performed prior to the measurement.

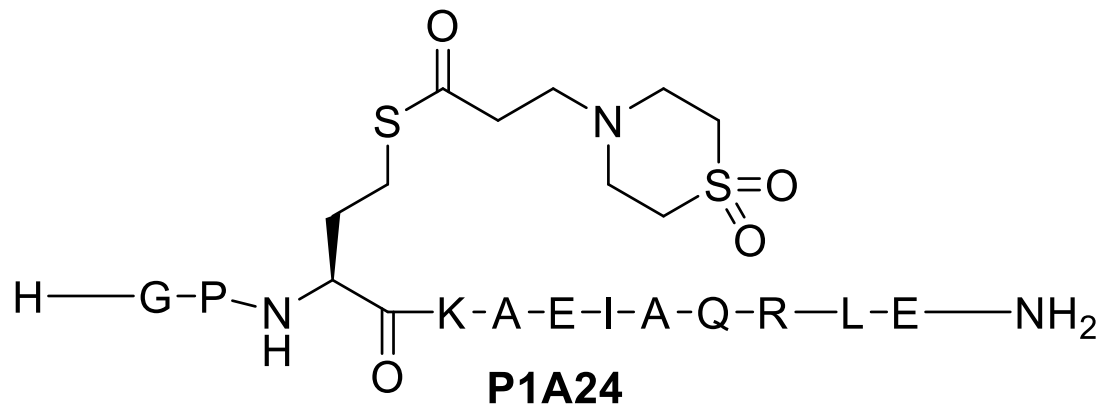

P1A24

Chemical Formula: $C_{63}H_{109}N_{19}O_{20}S_2$
Exact Mass: 1515.75
Molecular Weight: 1516.80

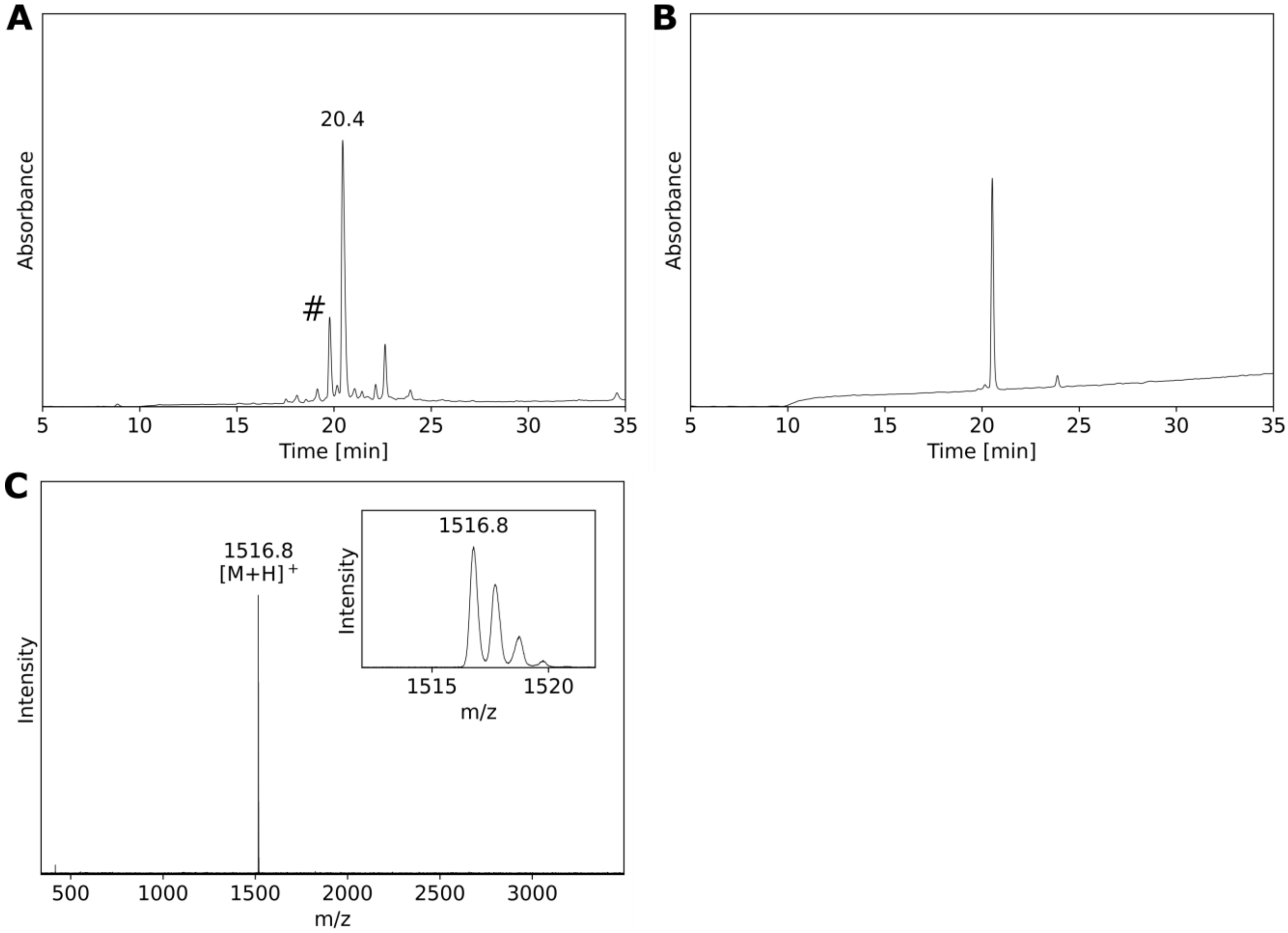

Figure S51: **P1A24** A) Analytical HPLC (220 nm) of crude peptide. HPLC Gradients are listed in Section 2.3.5 Table S3. # Peptide without homoserine. B) Analytical HPLC (220 nm) of pure peptide. C) MALDI-TOF MS of pure peptide. Calculated mass: $[M+H]^+$ 1516.8.

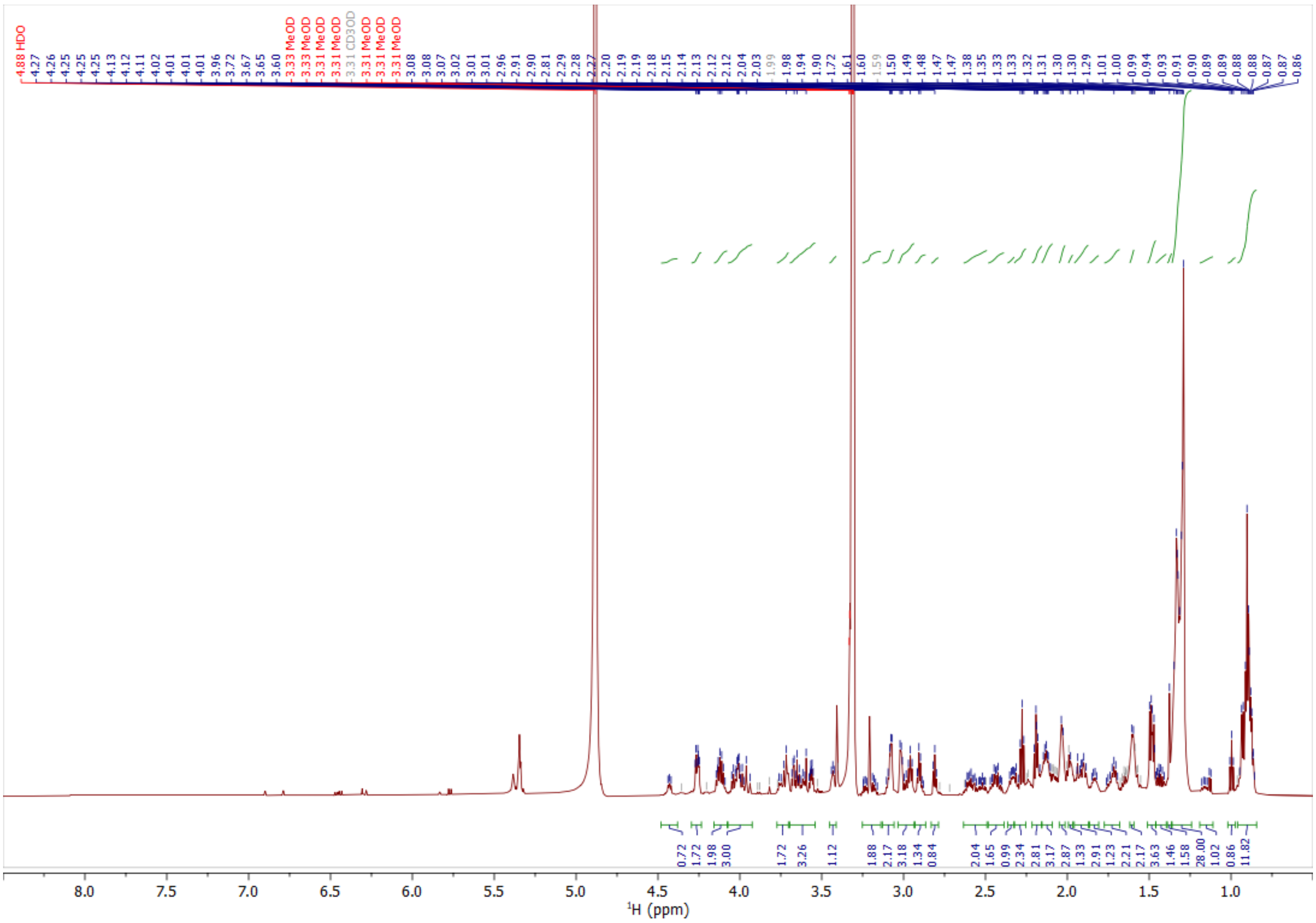

Figure S52: ^{1}H-NMR (700 MHz, MeOH-d_4, 295 K) of **P1A24**. Hydrogen-deuterium exchange was performed prior to the measurement.

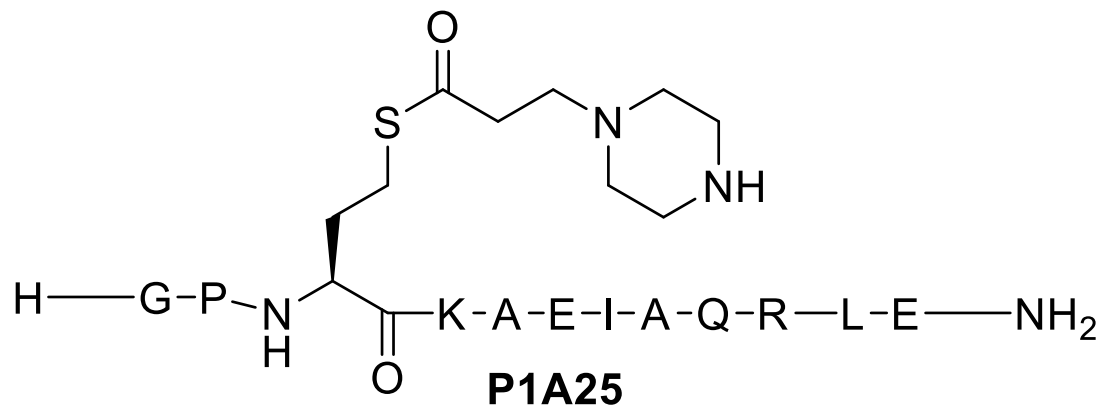

P1A25

Chemical Formula: $C_{63}H_{110}N_{20}O_{18}S$

Exact Mass: 1466.80

Molecular Weight: 1467.76

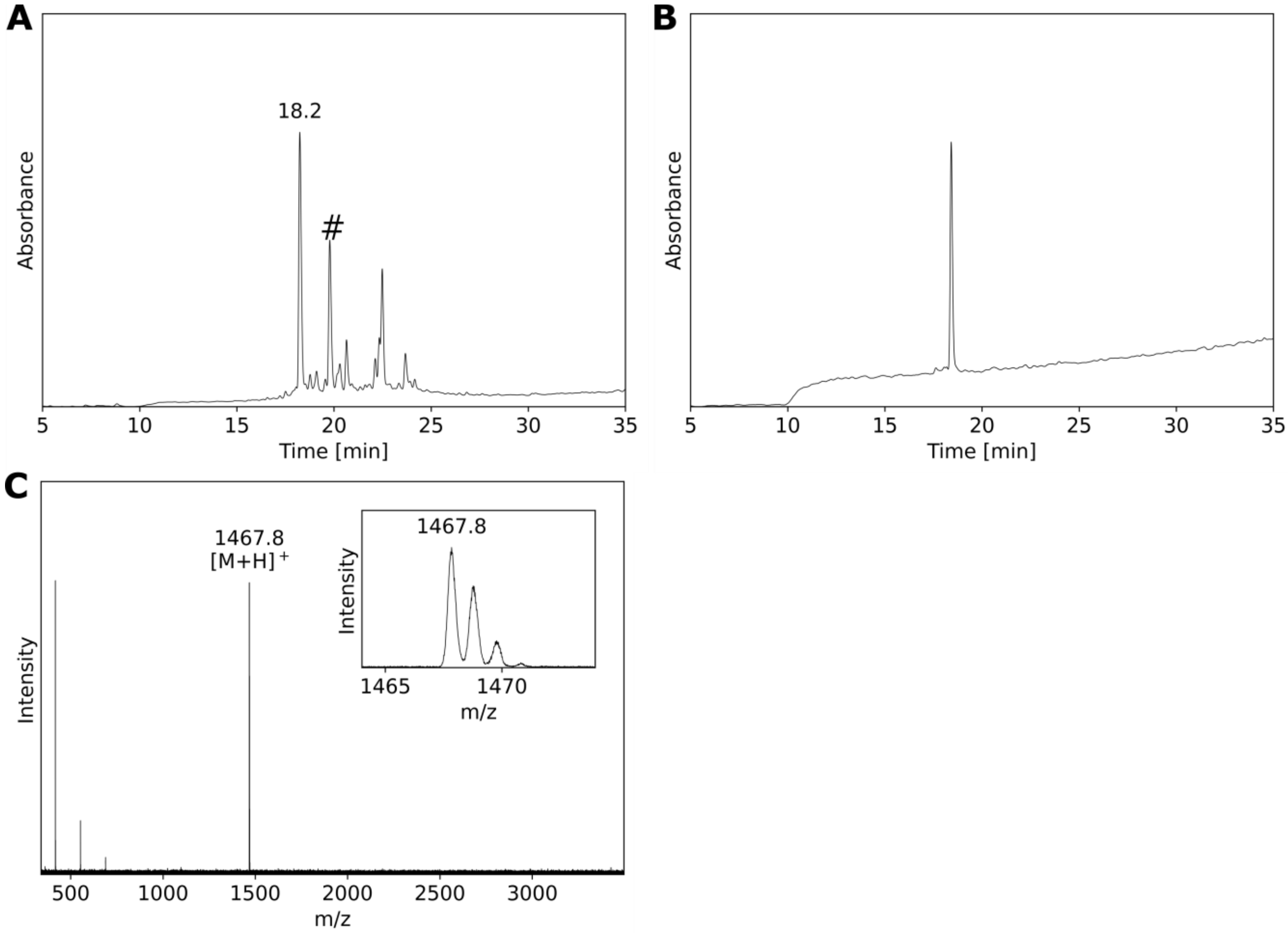

Figure S53: **P1A25** A) Analytical HPLC (220 nm) of crude peptide. HPLC Gradients are listed in Section 2.3.5 Table S3. # Peptide without homoserine. B) Analytical HPLC (220 nm) of pure peptide. C) MALDI-TOF MS of pure peptide. Calculated mass: $[M+H]^+$ 1467.8.

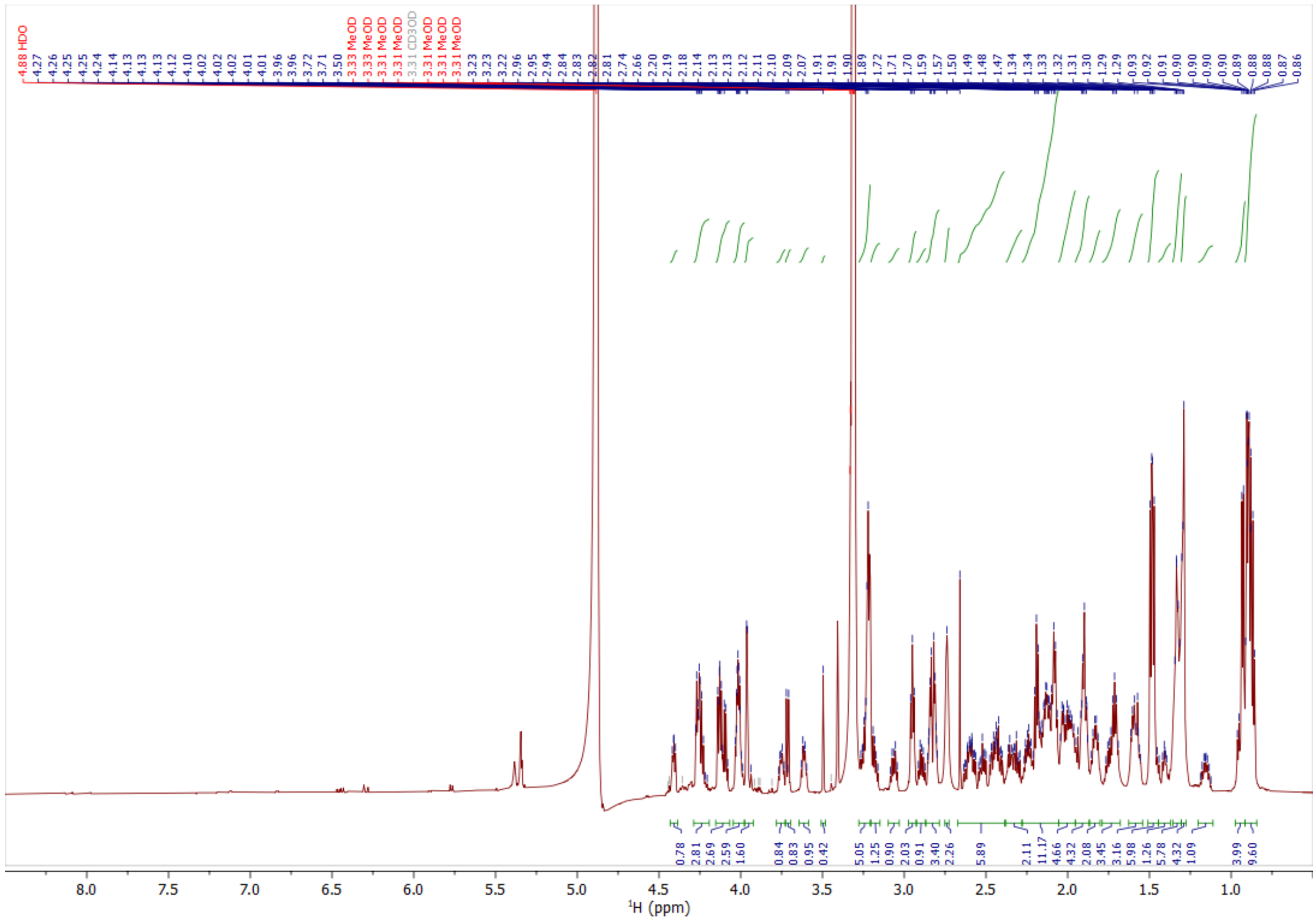

Figure S54: ^{1}H-NMR (700 MHz, MeOH-d_4, 295 K) of **P1A25**. Hydrogen-deuterium exchange was performed prior to the measurement.

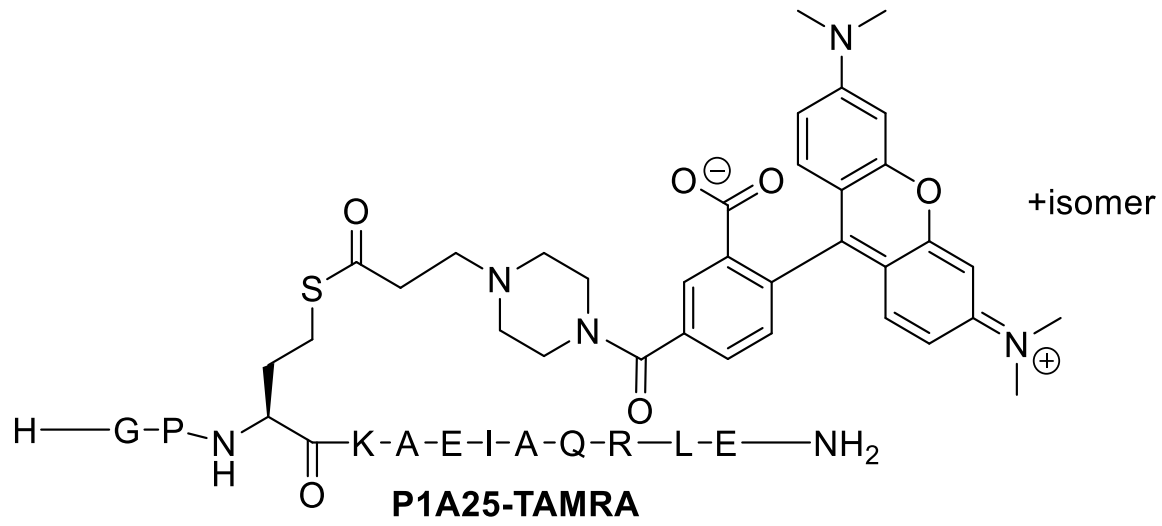

P1A25-TAMRA

Chemical Formula: $C_{88}H_{130}N_{22}O_{22}S$

Exact Mass: 1878.95

Molecular Weight: 1880.20

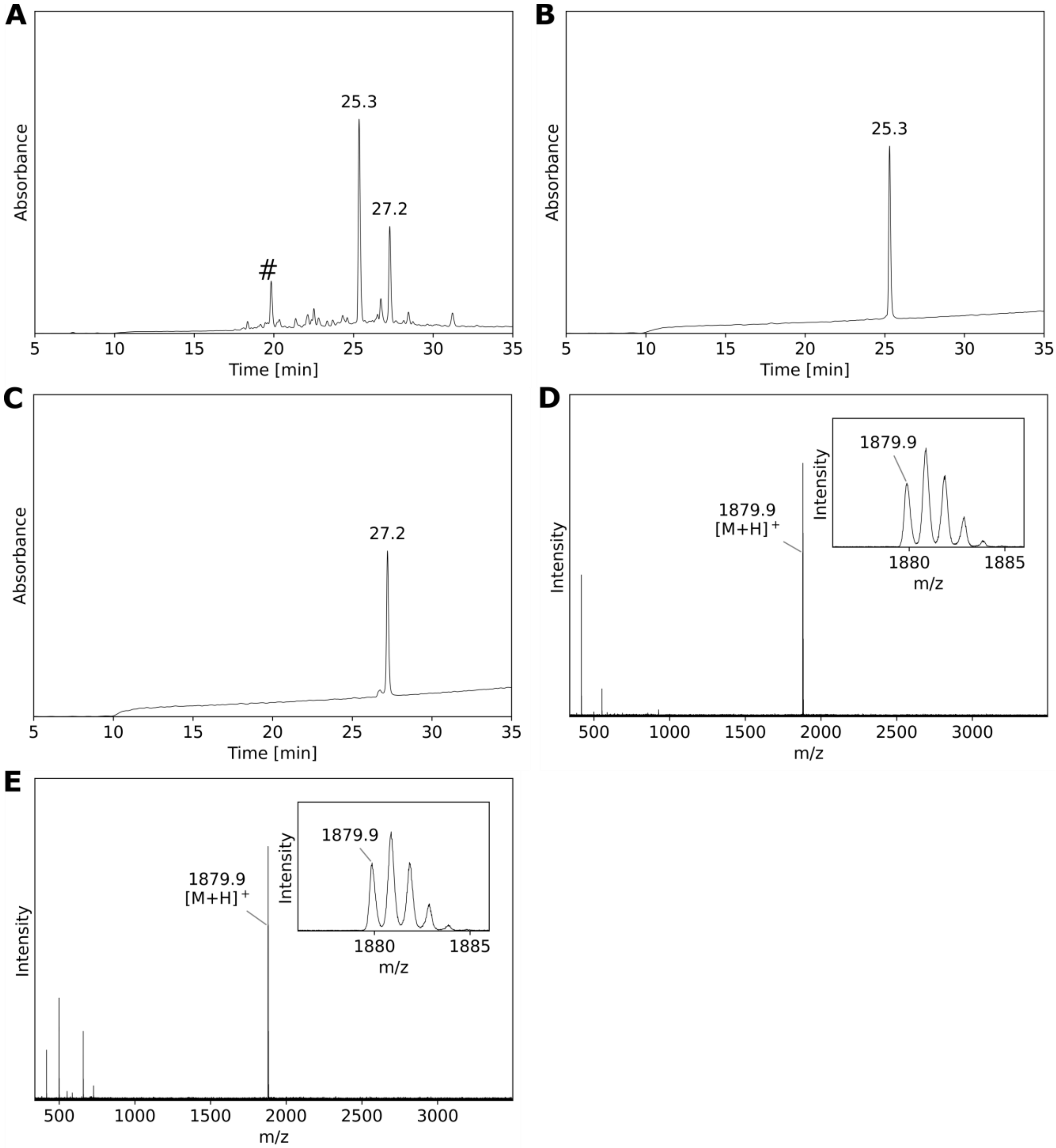

Figure S55: **P1A25-TAMRA** A) Analytical HPLC (220 nm) of crude peptide. HPLC Gradients are listed in Section 2.3.5 Table S3. # Peptide without homoserine. B-C) Analytical HPLC (220 nm) of pure peptide. D) MALDI-TOF MS of purified peptide with a retention time of 25.3 min. E) MALDI-TOF MS of purified peptide with a retention time of 27.2 min. Calculated mass: $[M+H]^+$ 1880.0. **P1A25-TAMRA** was prepared with the isomeric mixture 5(6)-TAMRA.

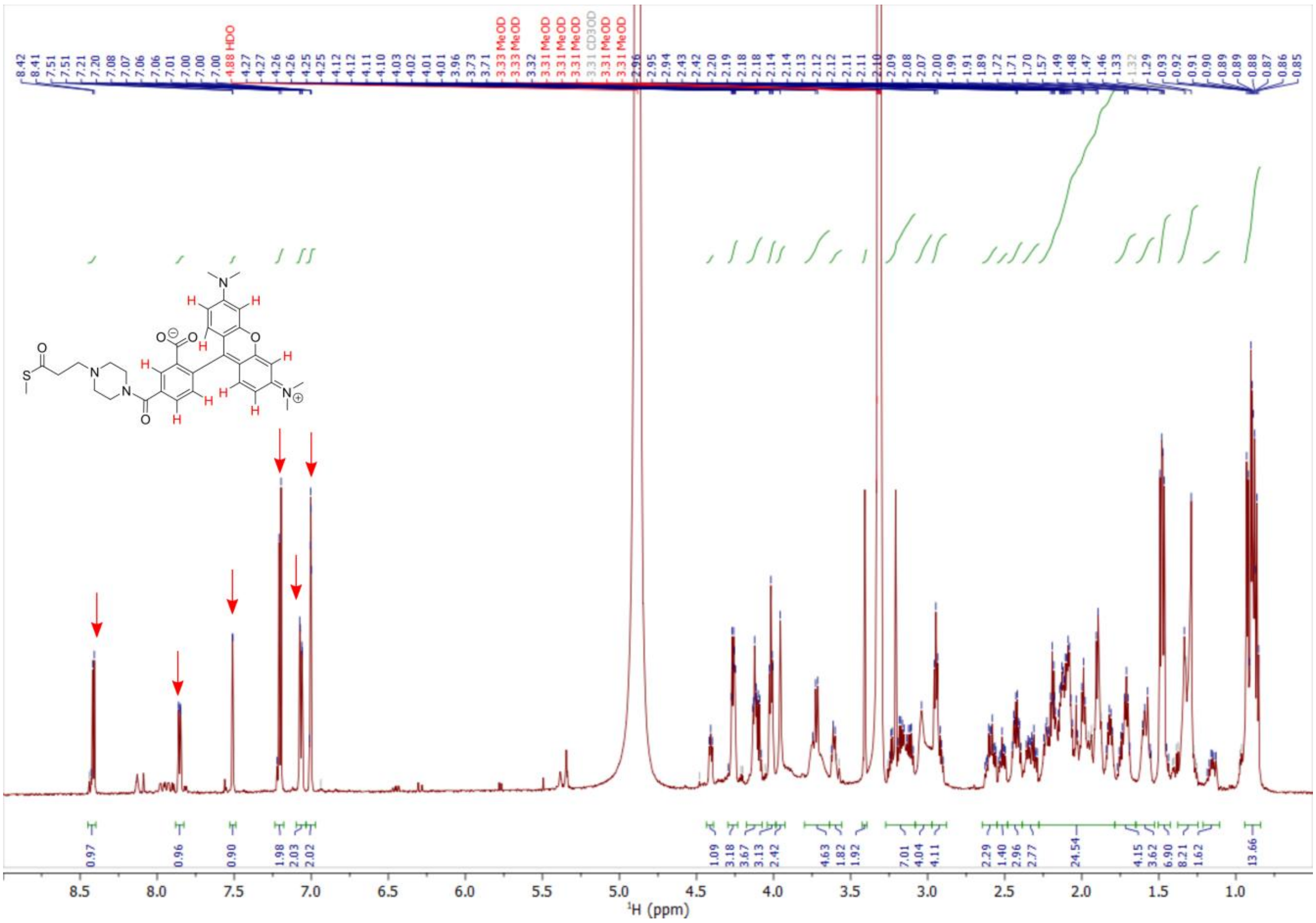

Figure S56: ^{1}H-NMR (700 MHz, MeOH-d_4, 295 K) of **P1A25-TAMRA** isomer with a retention time of 25.3 min. Hydrogen-deuterium exchange was performed prior to the measurement.

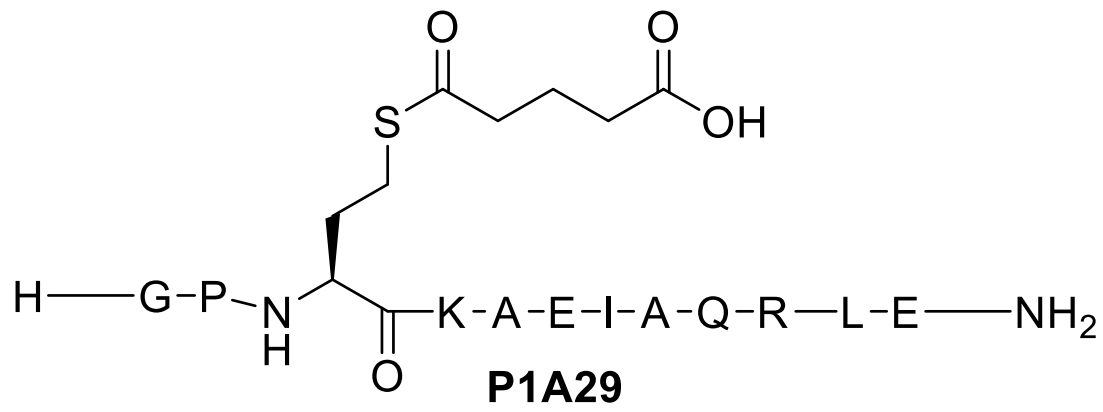

P1A29

Chemical Formula: $C_{61}H_{104}N_{18}O_{20}S$

Exact Mass: 1440.74

Molecular Weight: 1441.67

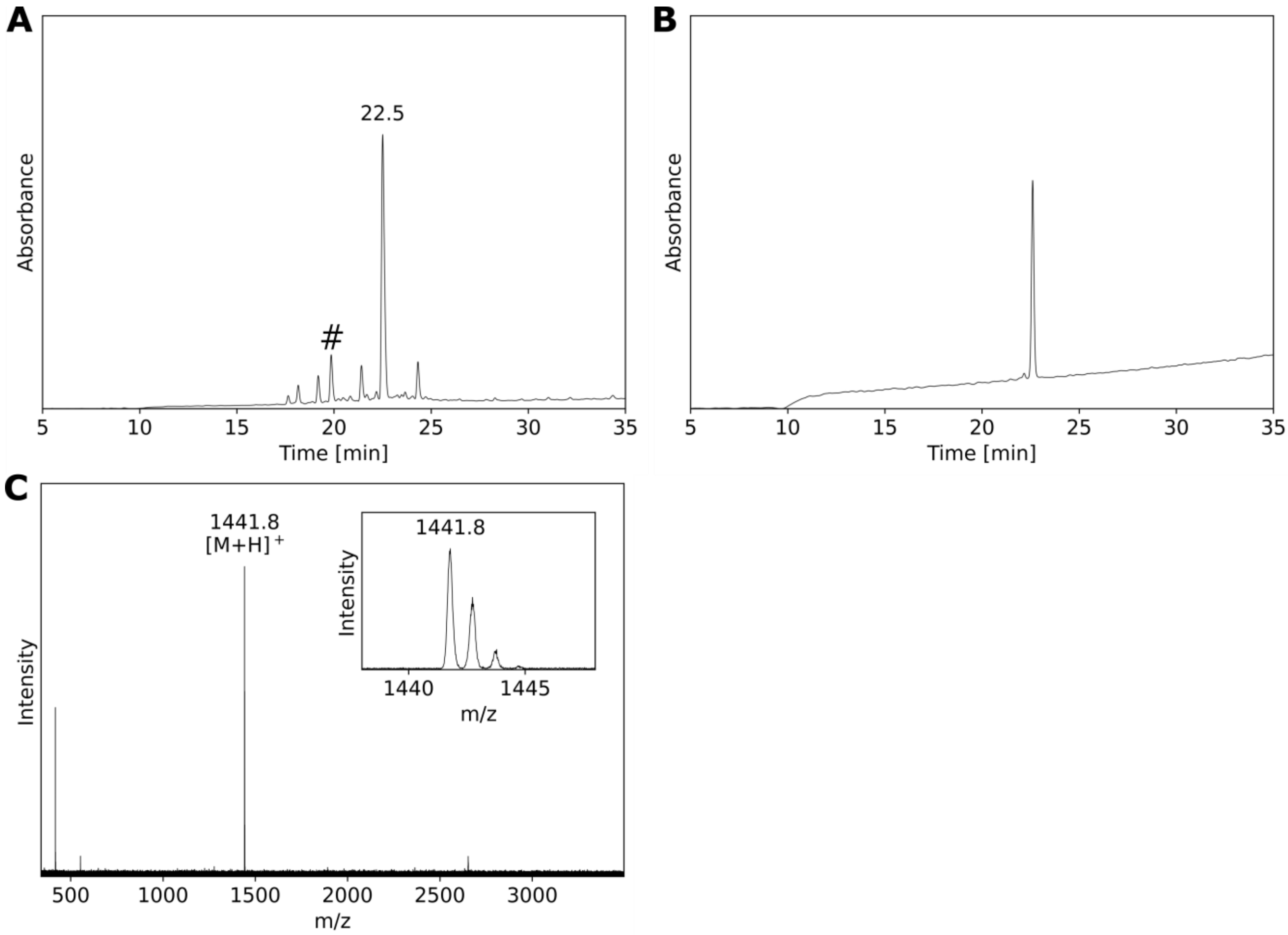

Figure S57: **P1A29** prepared with *tert*-butyl-pentynoate **A29**. A) Analytical HPLC (220 nm) of crude peptide. HPLC Gradients are listed in Section 2.3.5 Table S3. # Peptide without homoserine. B) Analytical HPLC (220 nm) of pure peptide. C) MALDI-TOF MS of pure peptide. Calculated mass: $[M+H]^+$ 1441.7.

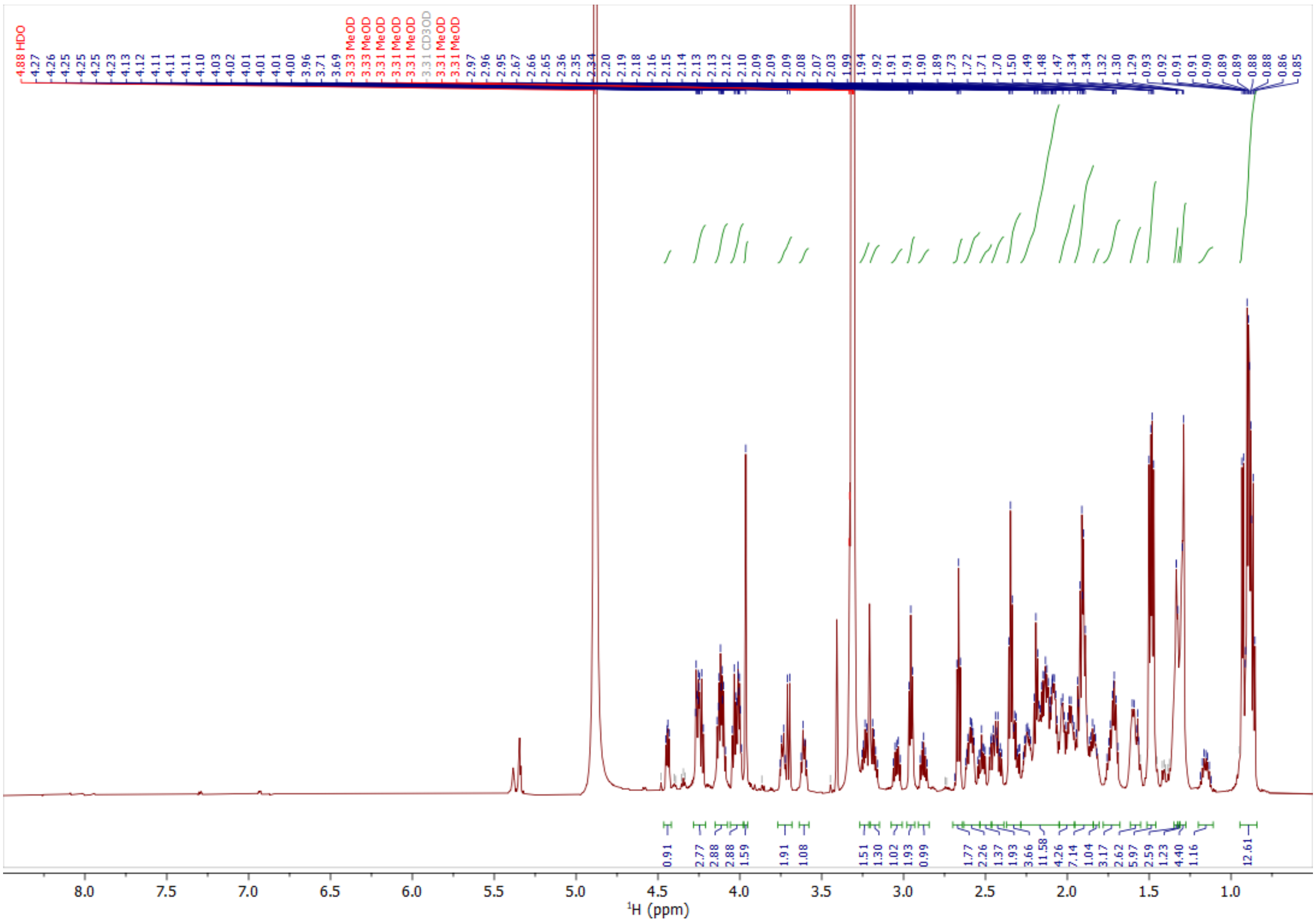

Figure S58: ^{1}H-NMR (700 MHz, MeOH-d_4, 295 K) of **P1A29**. Hydrogen-deuterium exchange was performed prior to the measurement.

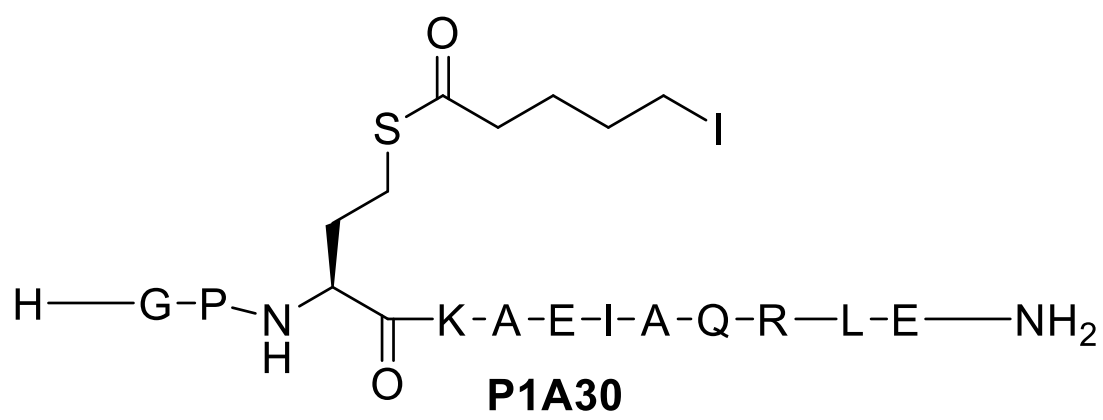

P1A30

Chemical Formula: $C_{61}H_{105}IN_{18}O_{18}S$

Exact Mass: 1536.66

Molecular Weight: 1537.58

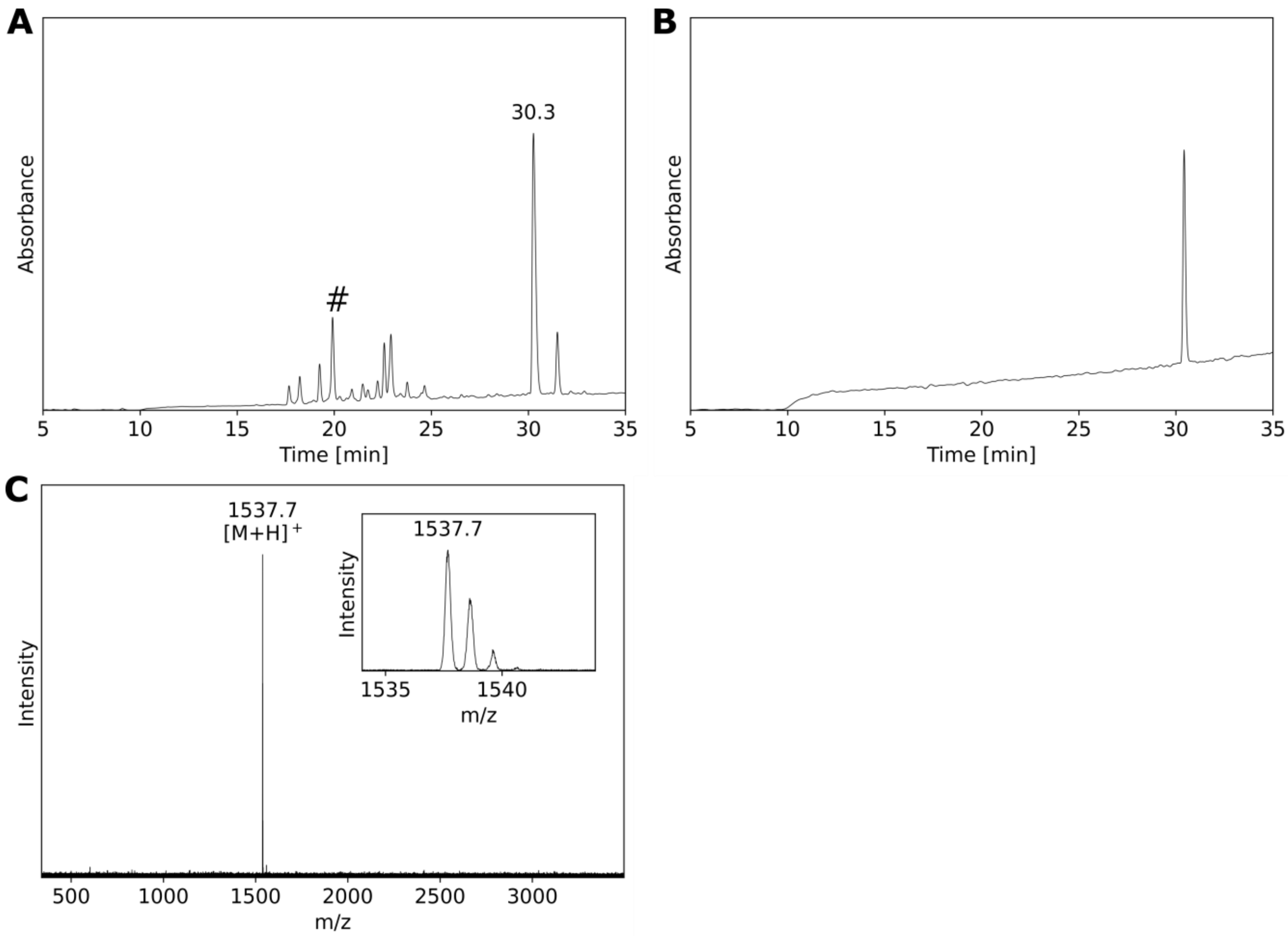

Figure S59: **P1A30** A) Analytical HPLC (220 nm) of crude peptide. HPLC Gradients are listed in Section 2.3.5 Table S3. # Peptide without homoserine. B) Analytical HPLC (220 nm) of pure peptide. C) MALDI-TOF MS of pure peptide. Calculated mass: $[M+H]^+$ 1537.7.

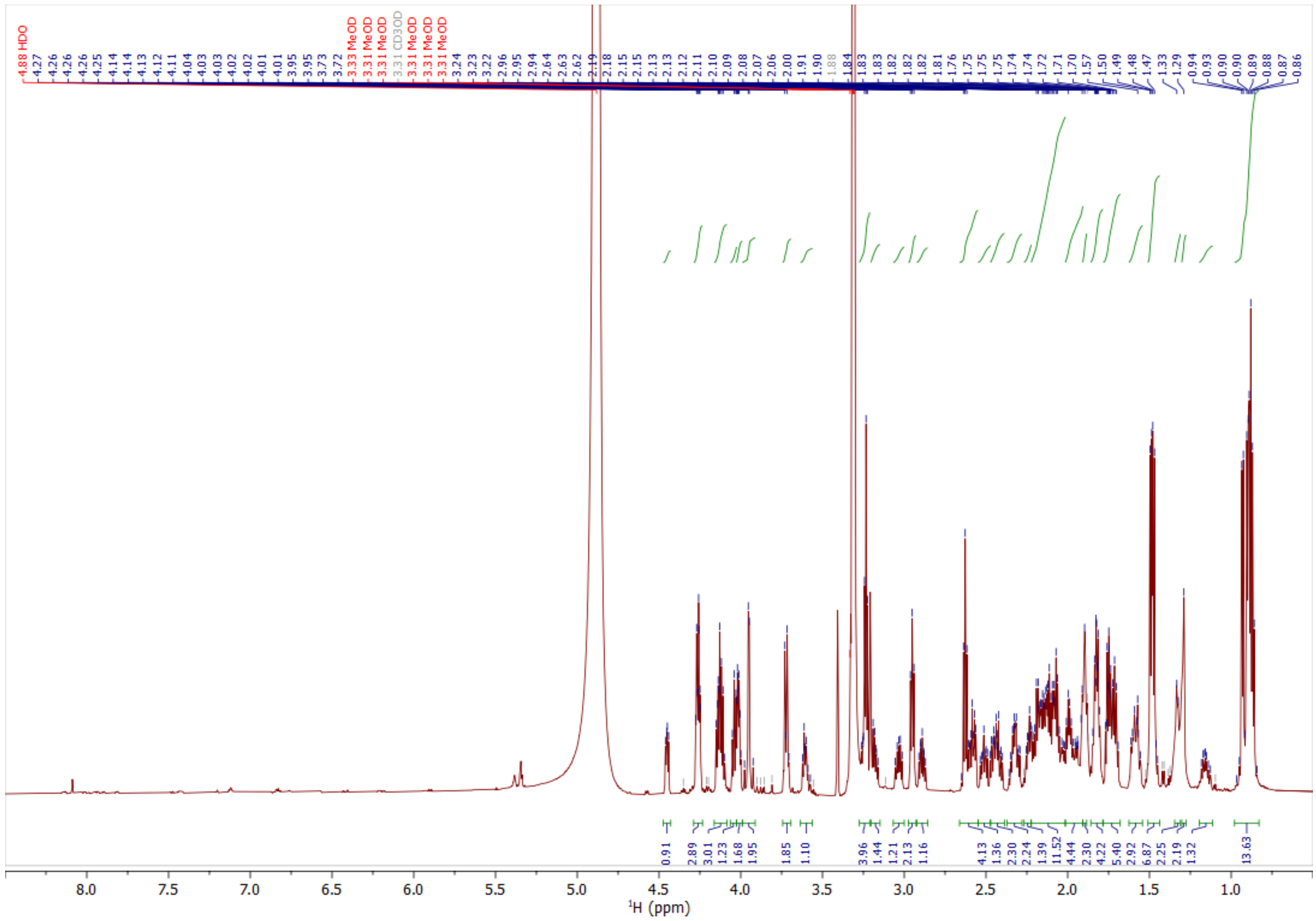

Figure S60: ^{1}H-NMR (700 MHz, MeOH-d_4, 295 K) of **P1A30**. Hydrogen-deuterium exchange was performed prior to the measurement.

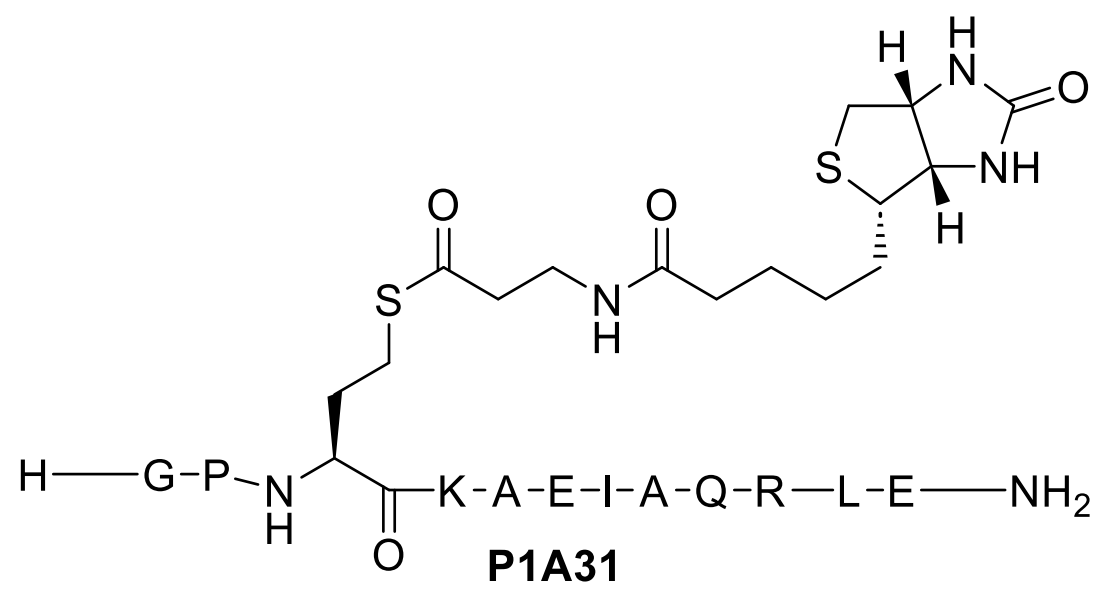

P1A31

Chemical Formula: $C_{69}H_{117}N_{21}O_{20}S_2$

Exact Mass: 1623.82

Molecular Weight: 1624.94

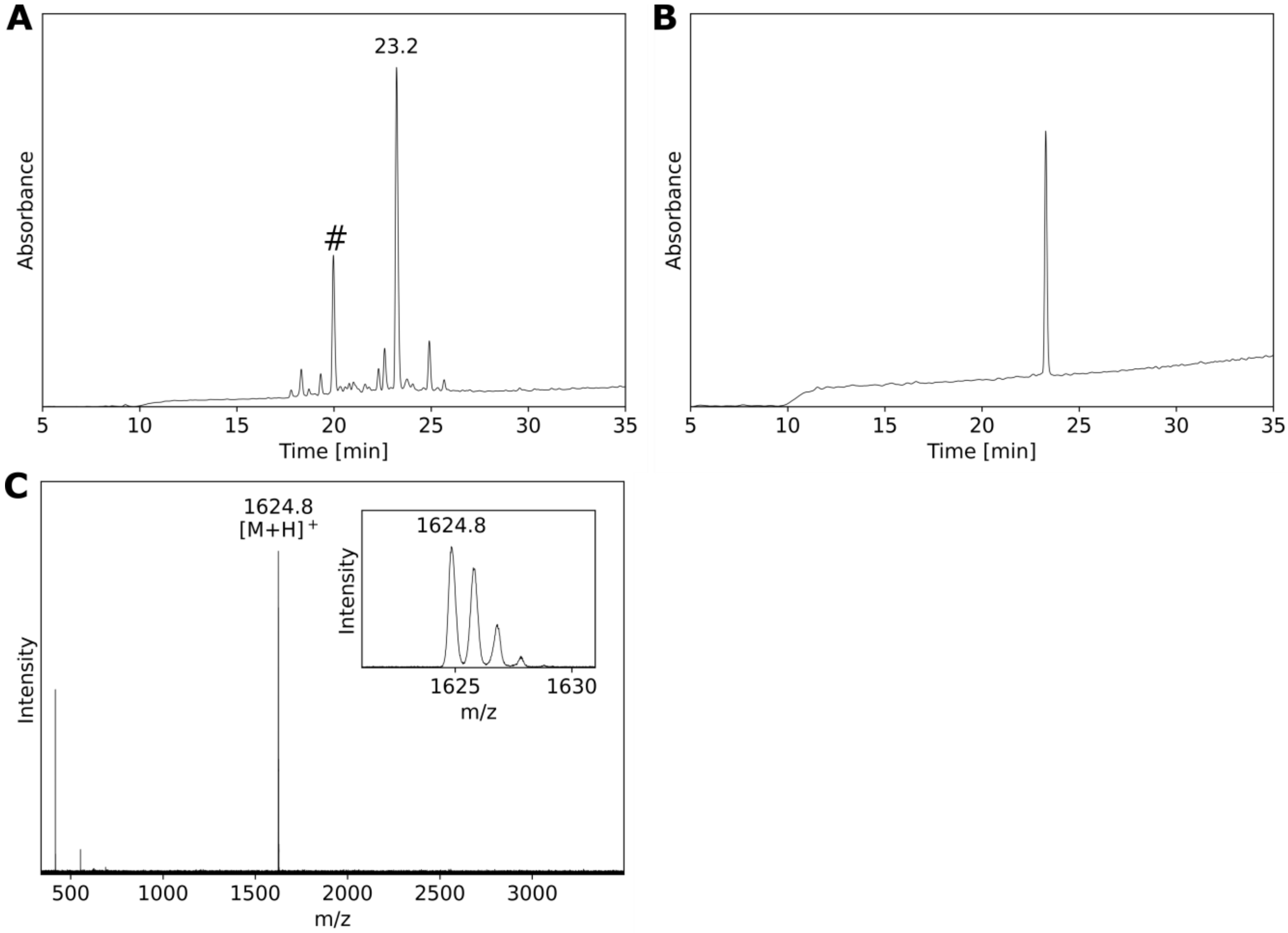

Figure S61: **P1A31** A) Analytical HPLC (220 nm) of crude peptide. HPLC Gradients are listed in Section 2.3.5 Table S3. # Peptide without homoserine. B) Analytical HPLC (220 nm) of pure peptide. C) MALDI-TOF MS of pure peptide. Calculated mass: $[M+H]^+$ 1624.8.

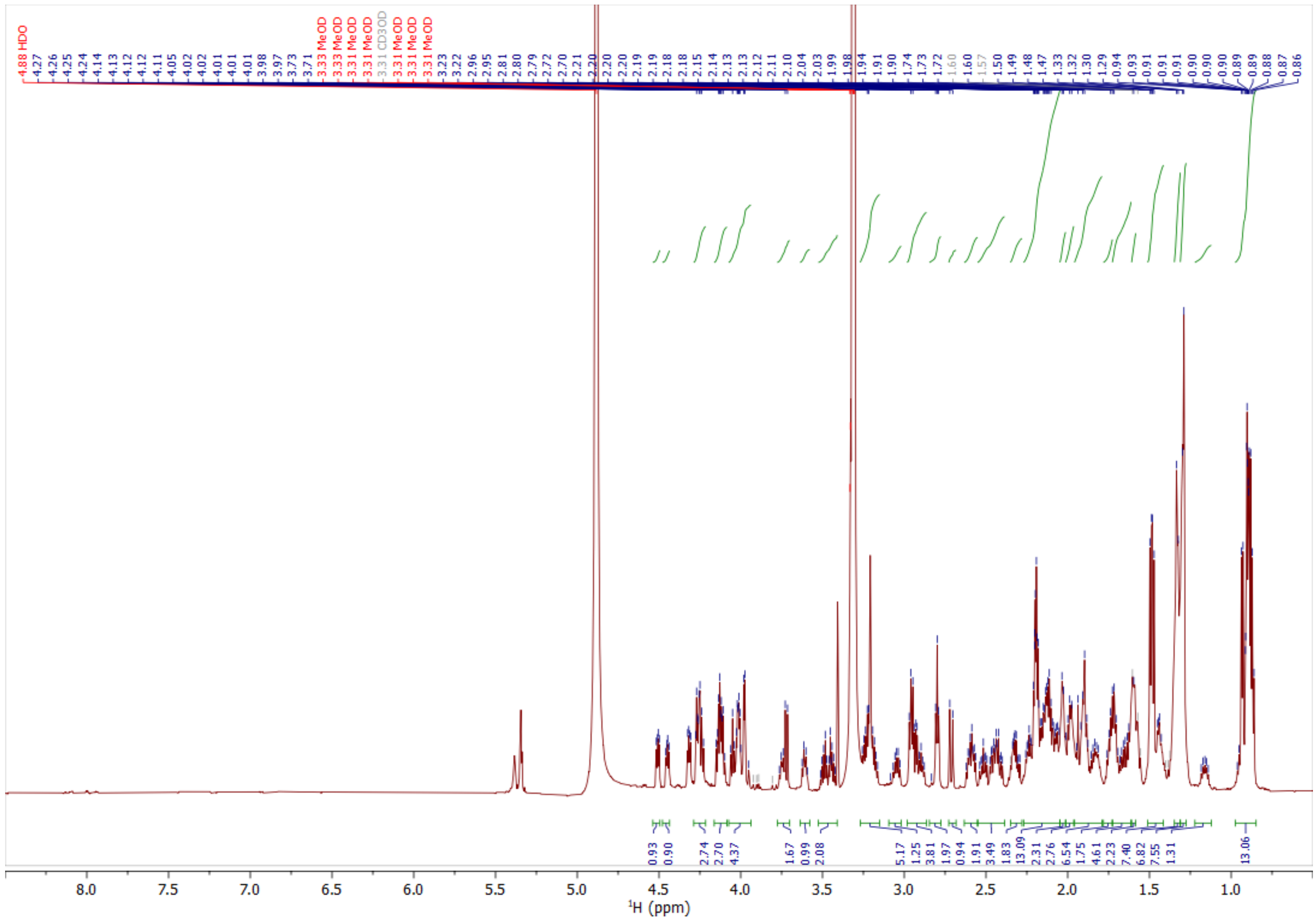

Figure S62: ^{1}H-NMR (700 MHz, MeOH-d_4, 295 K) of **P1A31**. Hydrogen-deuterium exchange was performed prior to the measurement.

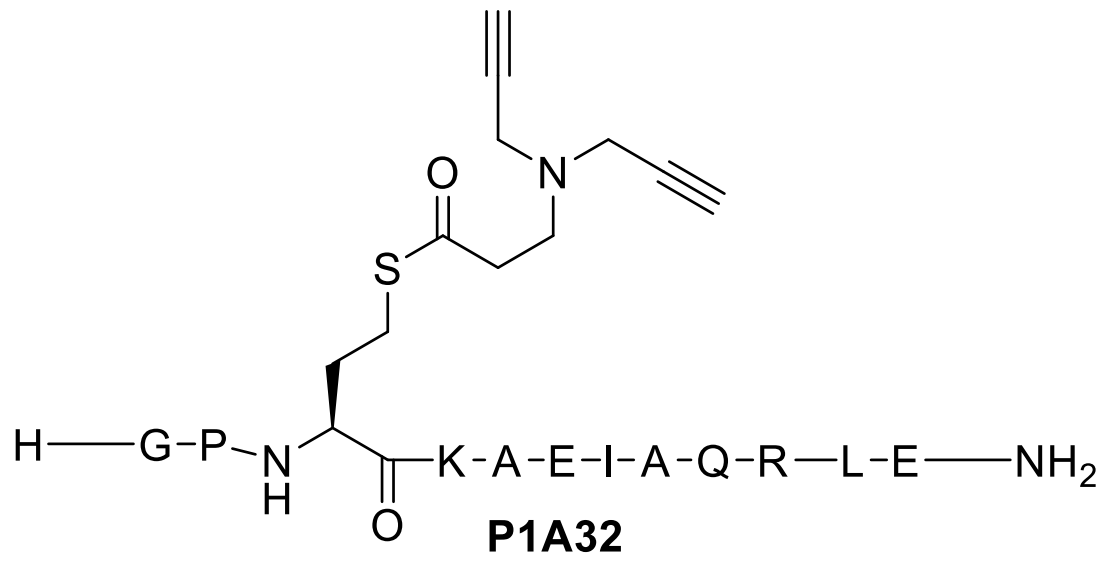

P1A32

Chemical Formula: $C_{65}H_{107}N_{19}O_{18}S$
Exact Mass: 1473.78
Molecular Weight: 1474.75

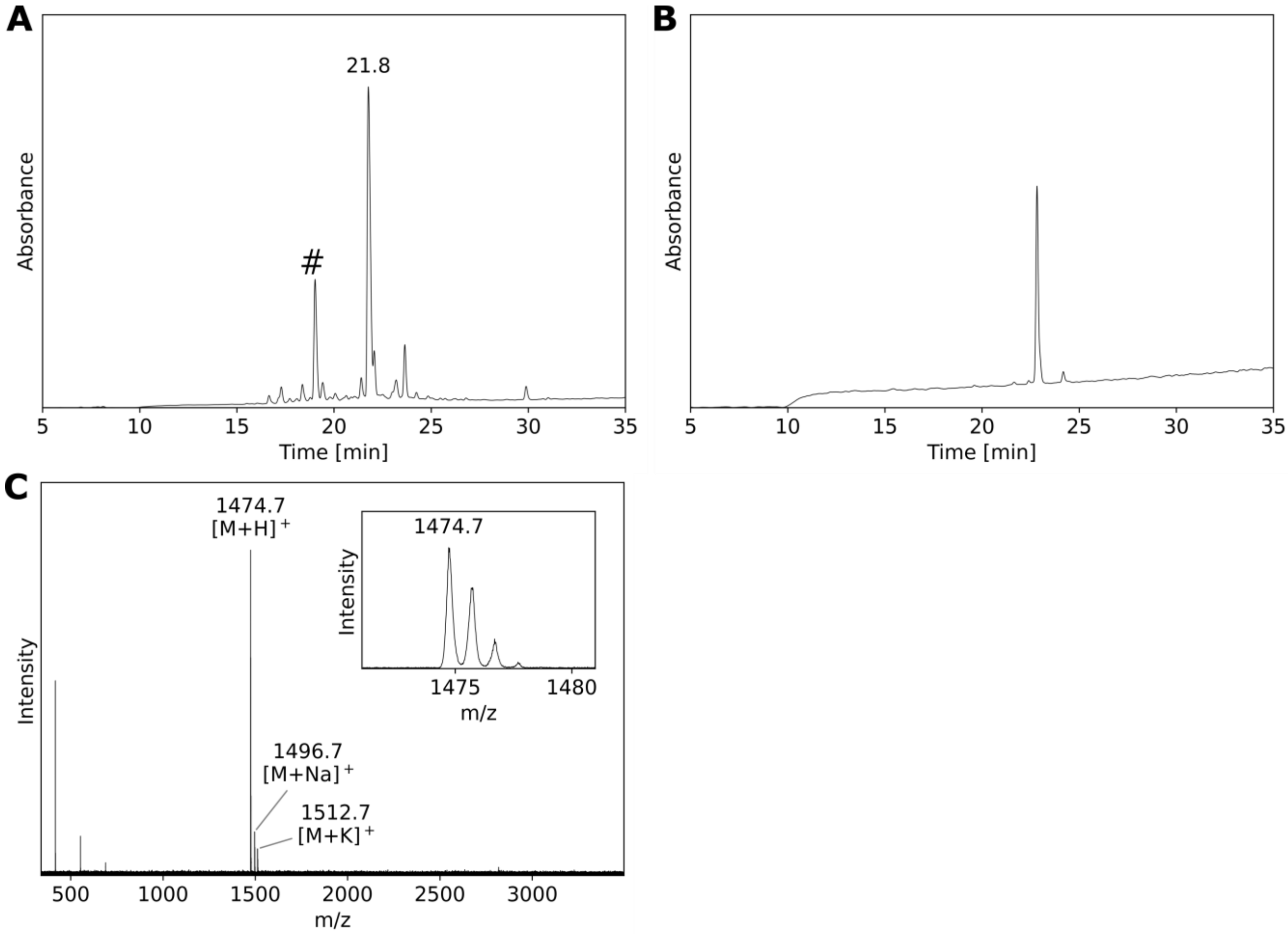

Figure S63: **P1A32** A) Analytical HPLC (220 nm) of crude peptide. HPLC Gradients are listed in Section 2.3.5 Table S3. # Peptide without homoserine. B) Analytical HPLC (220 nm) of pure peptide. C) MALDI-TOF MS of pure peptide. Calculated mass: [M+H]+ 1474.8.

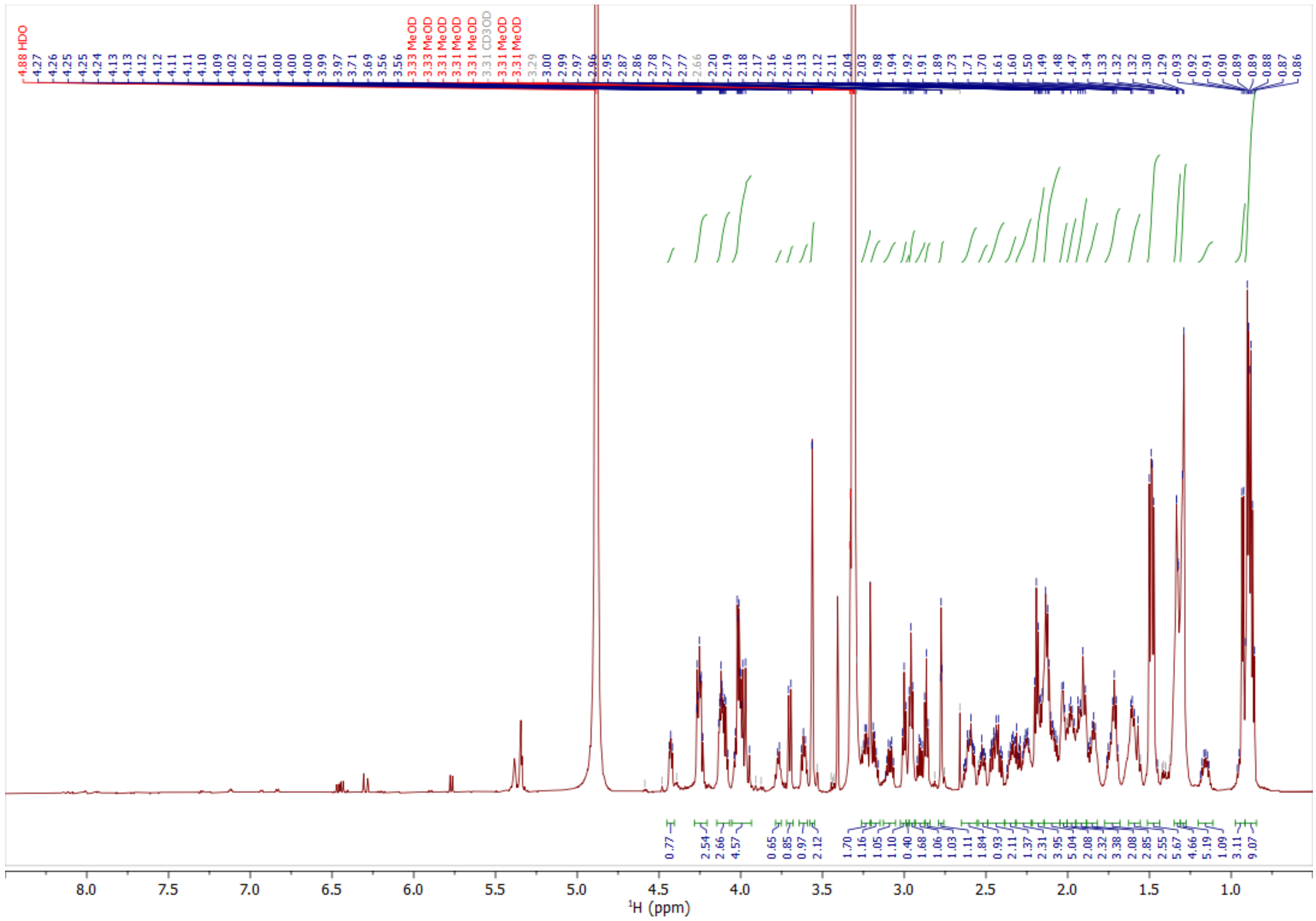

Figure S64: ^{1}H-NMR (700 MHz, MeOH-d_4, 295 K) of **P1A32**. Hydrogen-deuterium exchange was performed prior to the measurement.

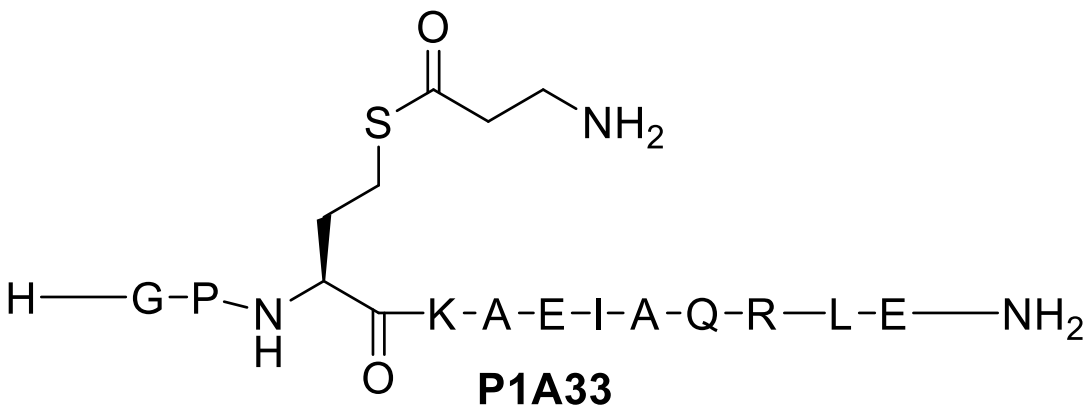

P1A33

Chemical Formula: $C_{59}H_{103}N_{19}O_{18}S$

Exact Mass: 1397.74

Molecular Weight: 1398.65

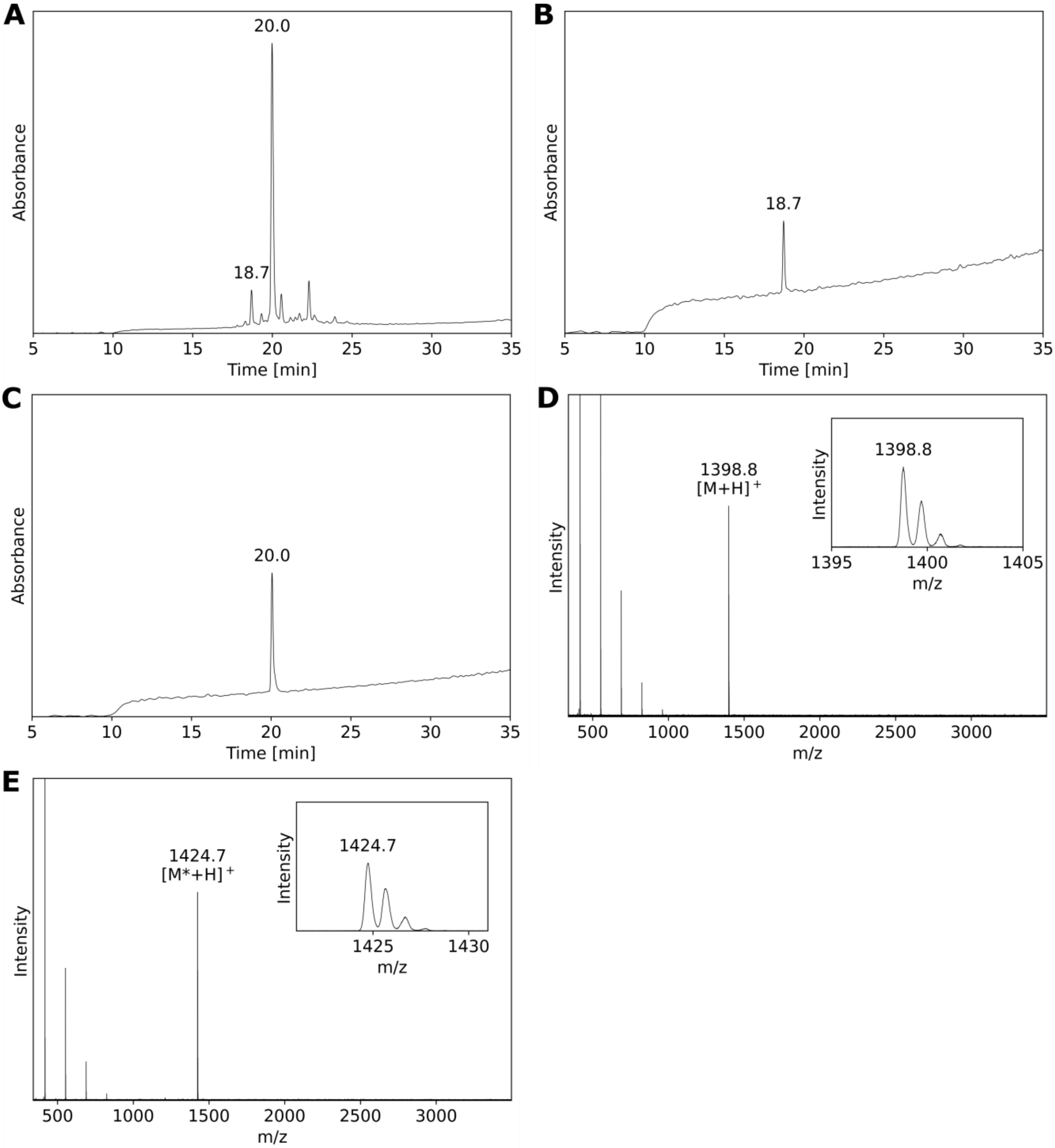

Figure S65: **P1A33** A) Analytical HPLC (220 nm) of crude peptide. HPLC Gradients are listed in Section 2.3.5 Table S3. B-C) Analytical HPLC (220 nm) of pure peptide. D) MALDI-TOF MS of purified peptide with a retention time of 18.7 min. E) MALDI-TOF MS of purified peptide with a retention time of 20.0 min. Calculated mass for **P1A33**: $[M+H]^+$ 1398.8.

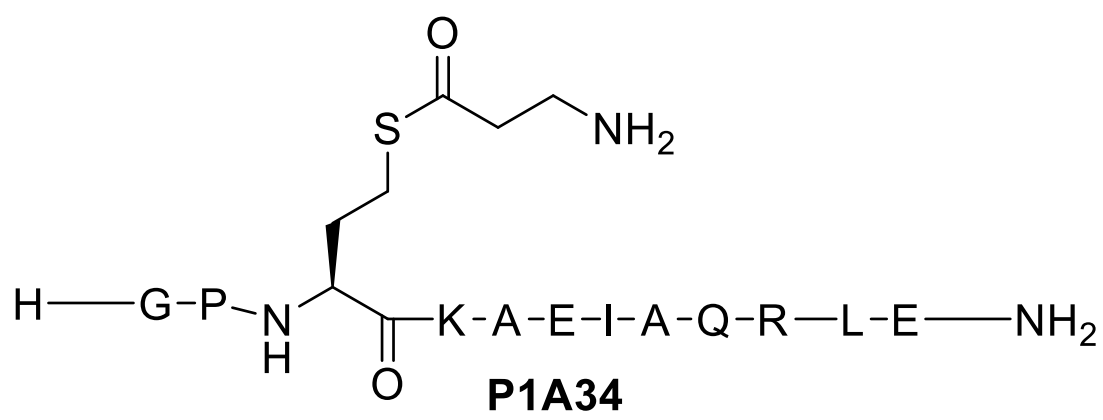

P1A34

Chemical Formula: $C_{59}H_{103}N_{19}O_{18}S$

Exact Mass: 1397.74

Molecular Weight: 1398.65

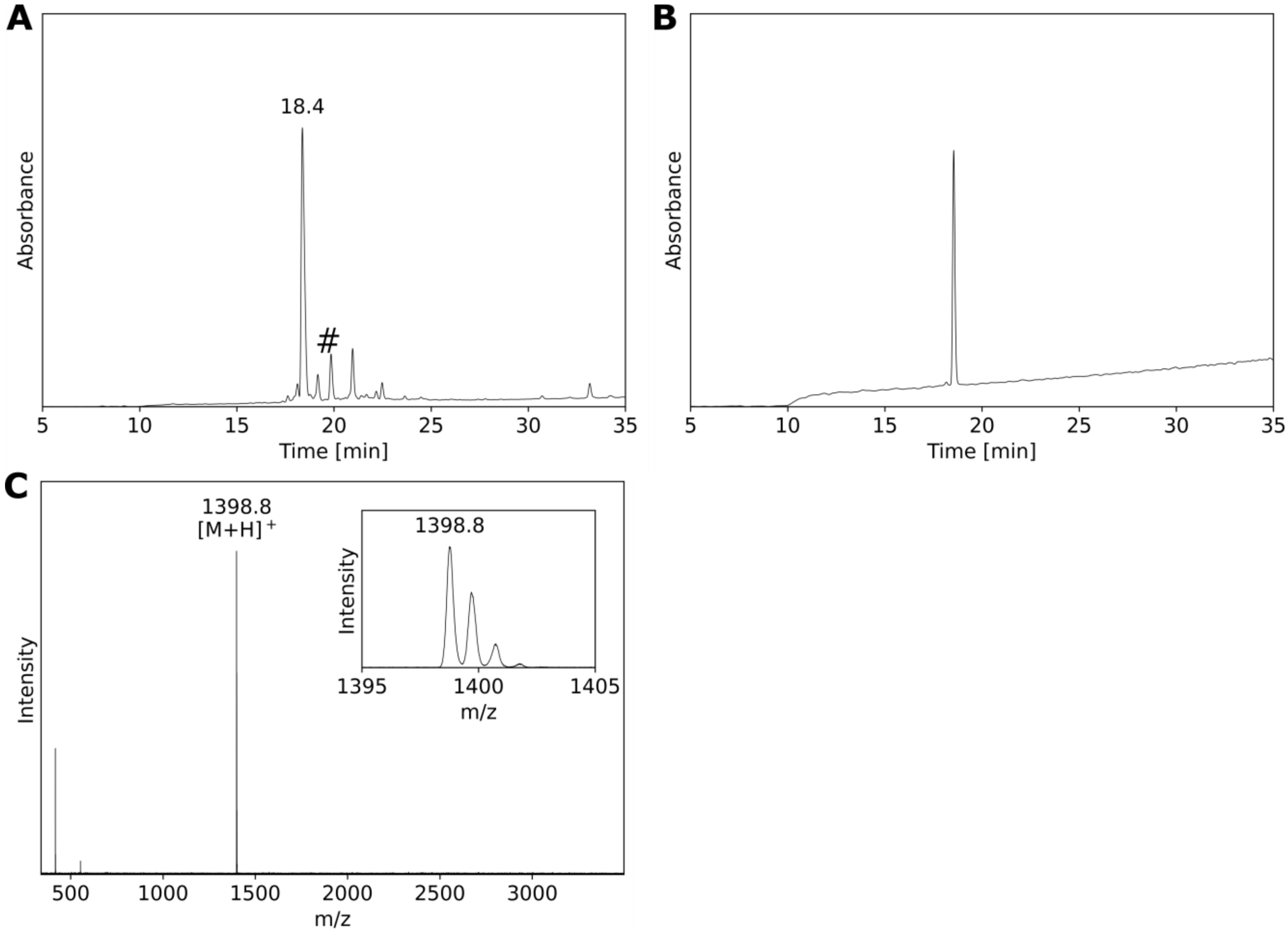

Figure S66: **P1A34** prepared with Boc-propargylamine **A34** A) Analytical HPLC (220 nm) of crude peptide. HPLC Gradients are listed in Section 2.3.5 Table S3. # Peptide without homoserine. B) Analytical HPLC (220 nm) of pure peptide. C) MALDI-TOF MS of pure peptide. Calculated mass: $[M+H]^+$ 1398.8.

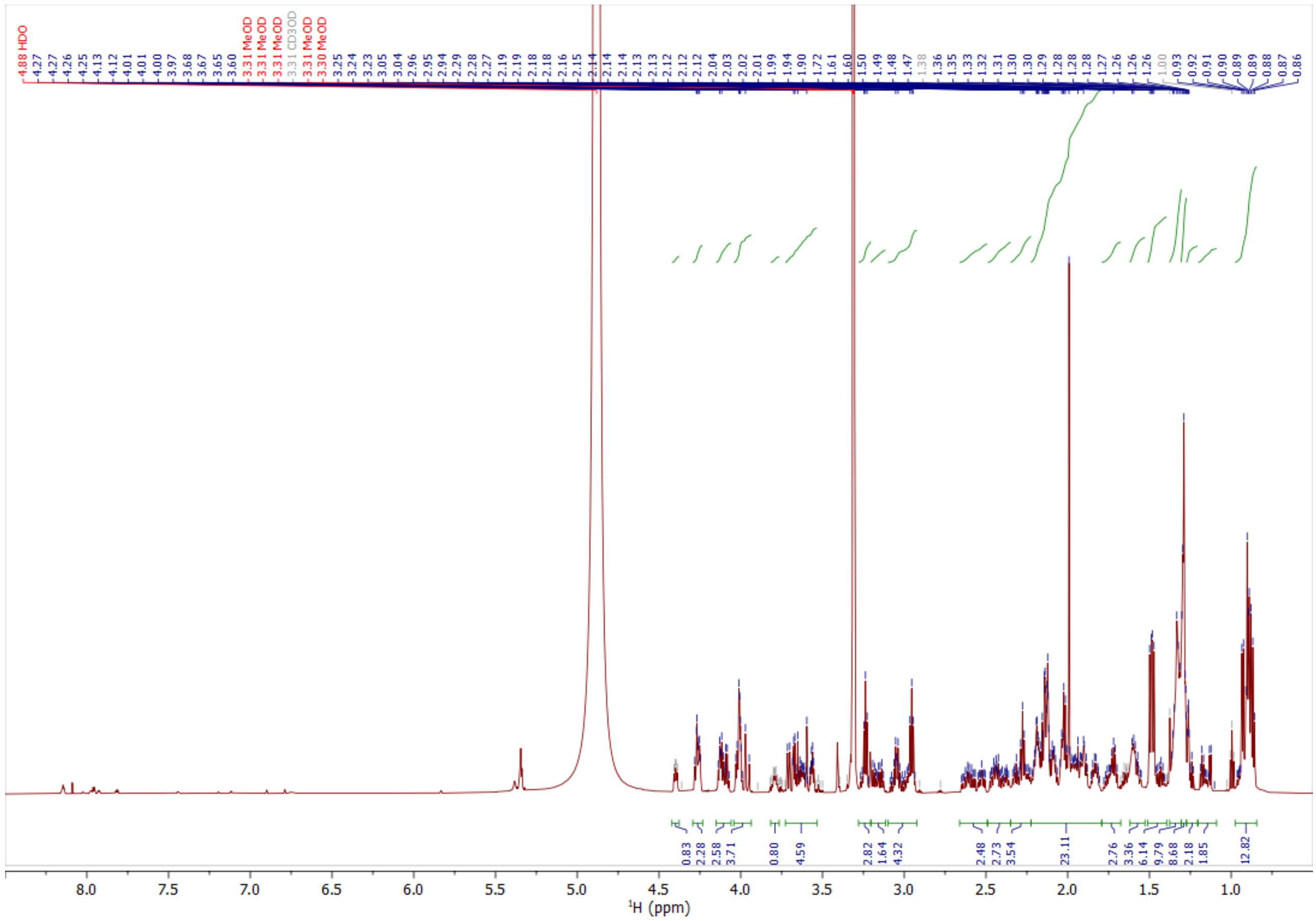

Figure S67: ^{1}H-NMR (700 MHz, MeOH-d_4, 295 K) of **P1A34**. Hydrogen-deuterium exchange was performed prior to the measurement.

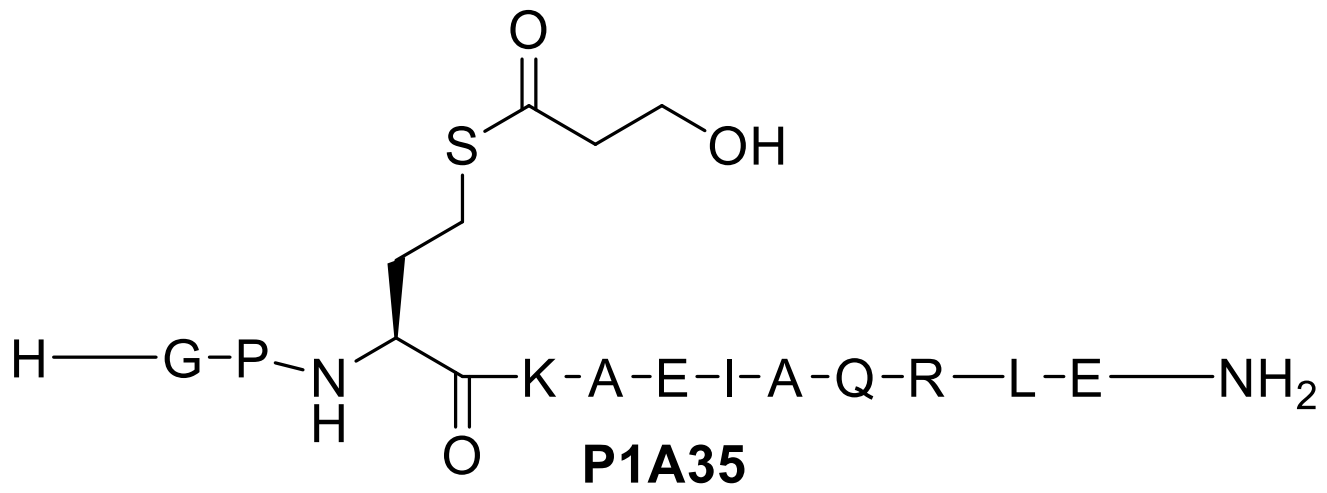

P1A35

Chemical Formula: $C_{59}H_{102}N_{18}O_{19}S$

Exact Mass: 1398.73

Molecular Weight: 1399.63

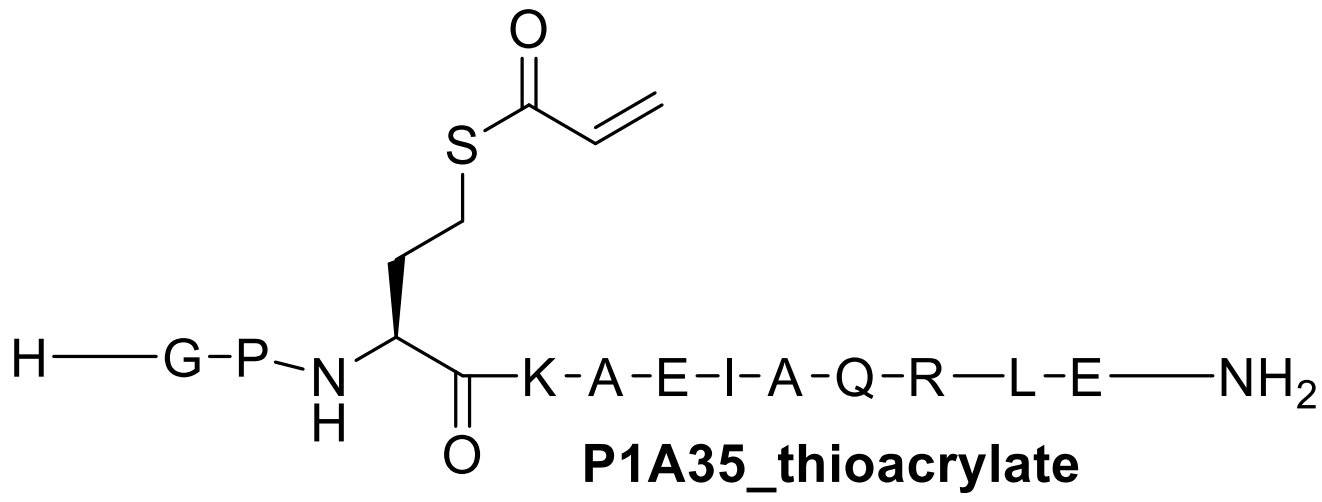

P1A35_thioacrylate

Chemical Formula: $C_{59}H_{100}N_{18}O_{18}S$

Exact Mass: 1380.72

Molecular Weight: 1381.62

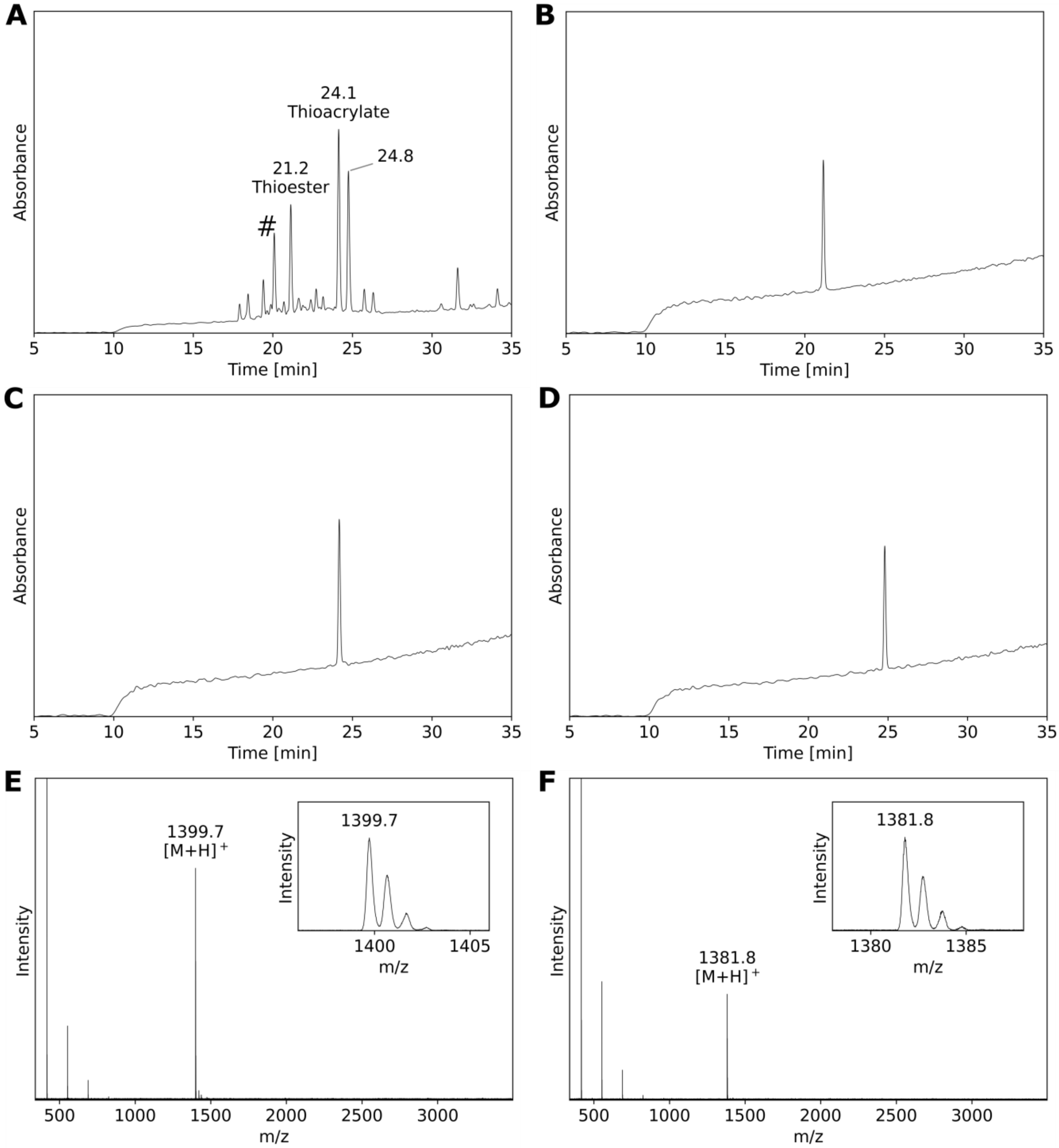

G

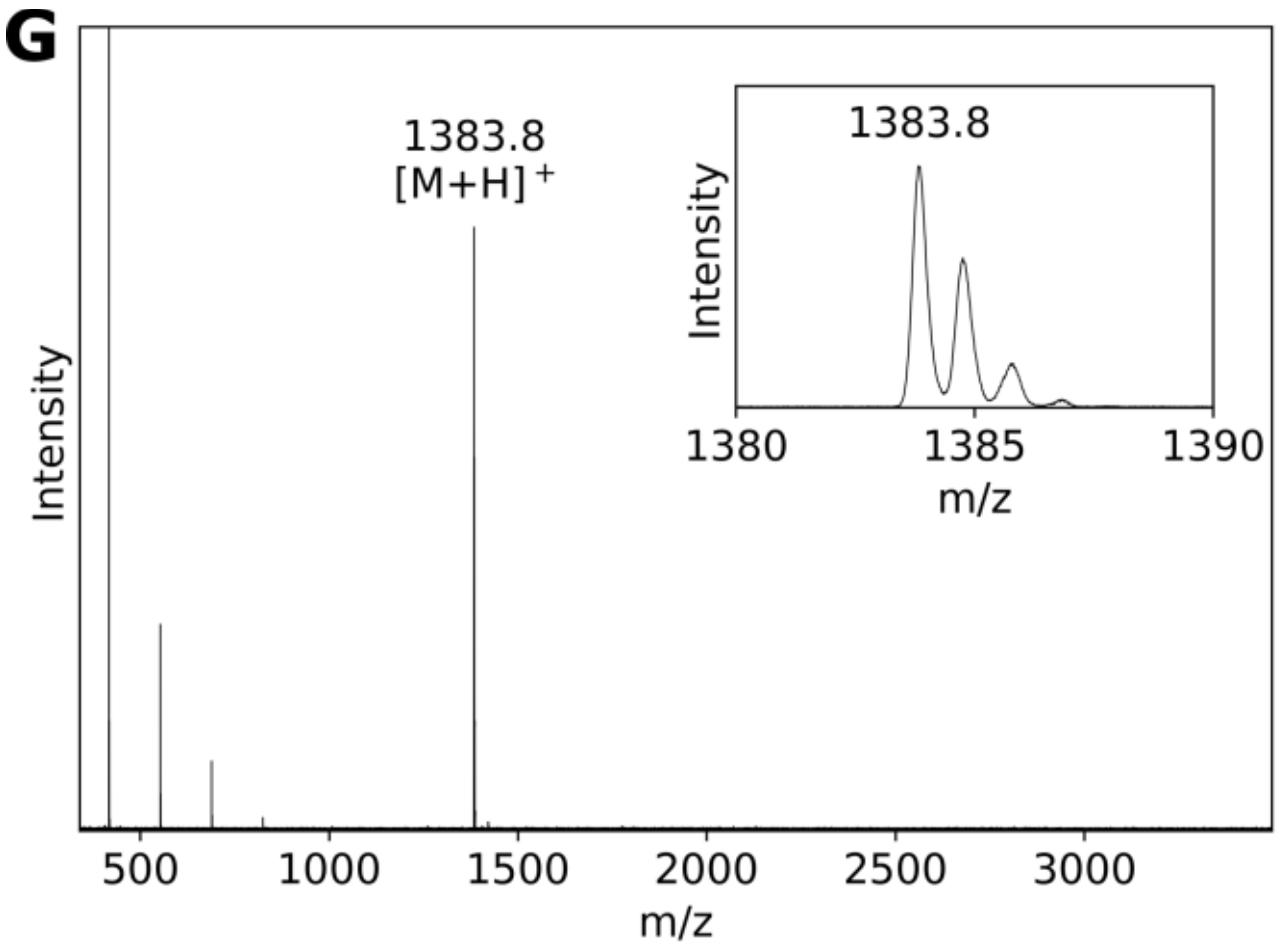

Figure S68: **P1A35** A) Analytical HPLC (220 nm) of crude peptide. HPLC Gradients are listed in Section 2.3.5 Table S3. # Peptide without homoserine. B) Analytical HPLC (220 nm) of purified peptide with a retention time of 21.2 min. C) Analytical HPLC (220 nm) of purified peptide with a retention time of 24.1 min. D) Analytical HPLC (220 nm) of purified peptide with a retention time of 24.8 min. E) MALDI-TOF MS of purified peptide with a retention time of 21.2 min. F) MALDI-TOF MS of purified peptide with a retention time of 24.1 min. G) MALDI-TOF MS of purified peptide with a retention time of 24.8 min. Calculated mass for **P1A35**: $[M+H]^+$ 1399.7. Calculated mass for **P1A35_thioacrylate**: $[M+H]^+$ 1381.7.

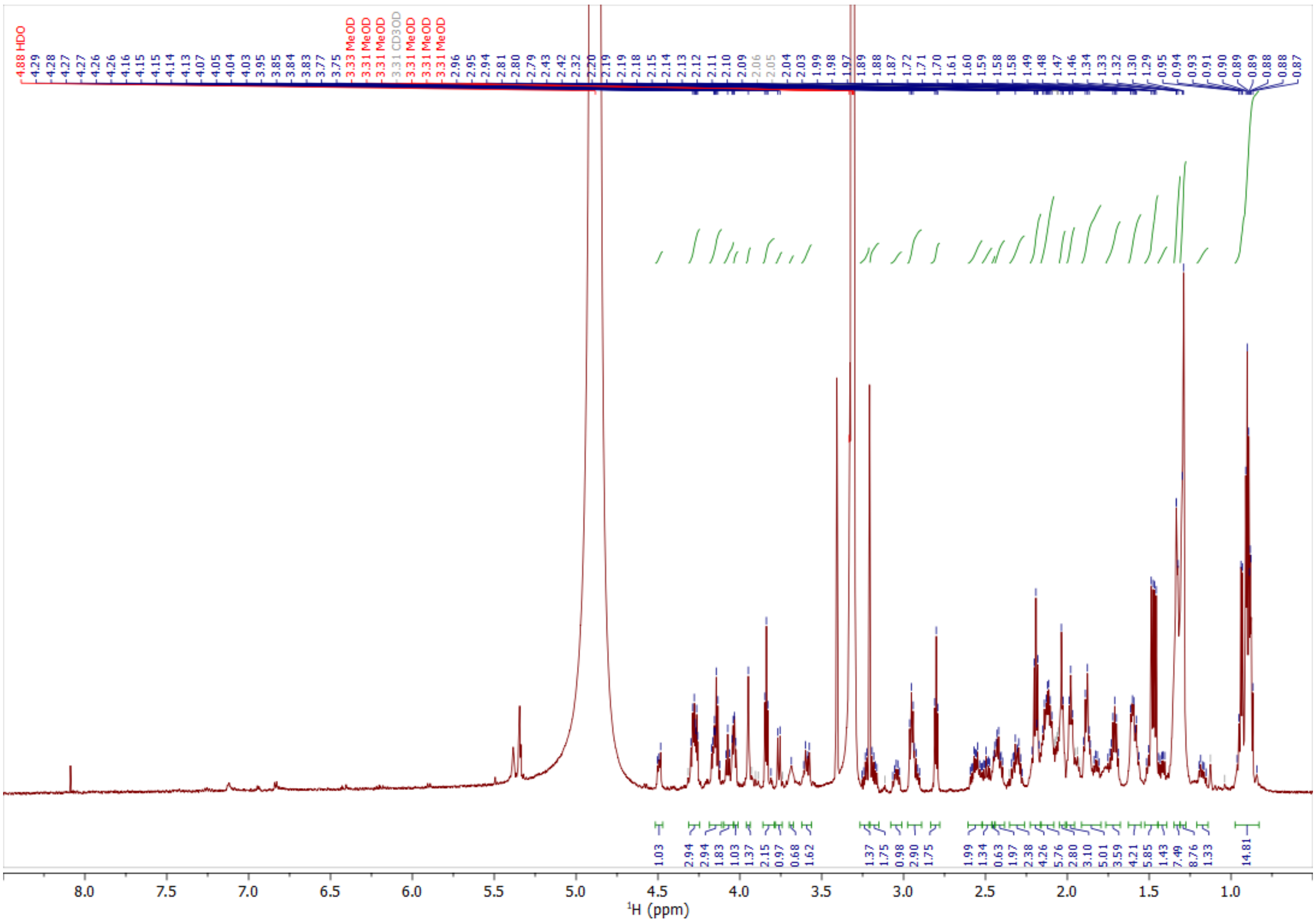

Figure S69: ^{1}H-NMR (700 MHz, MeOH-d_4, 295 K) of **P1A35**. Hydrogen-deuterium exchange was performed prior to the measurement.

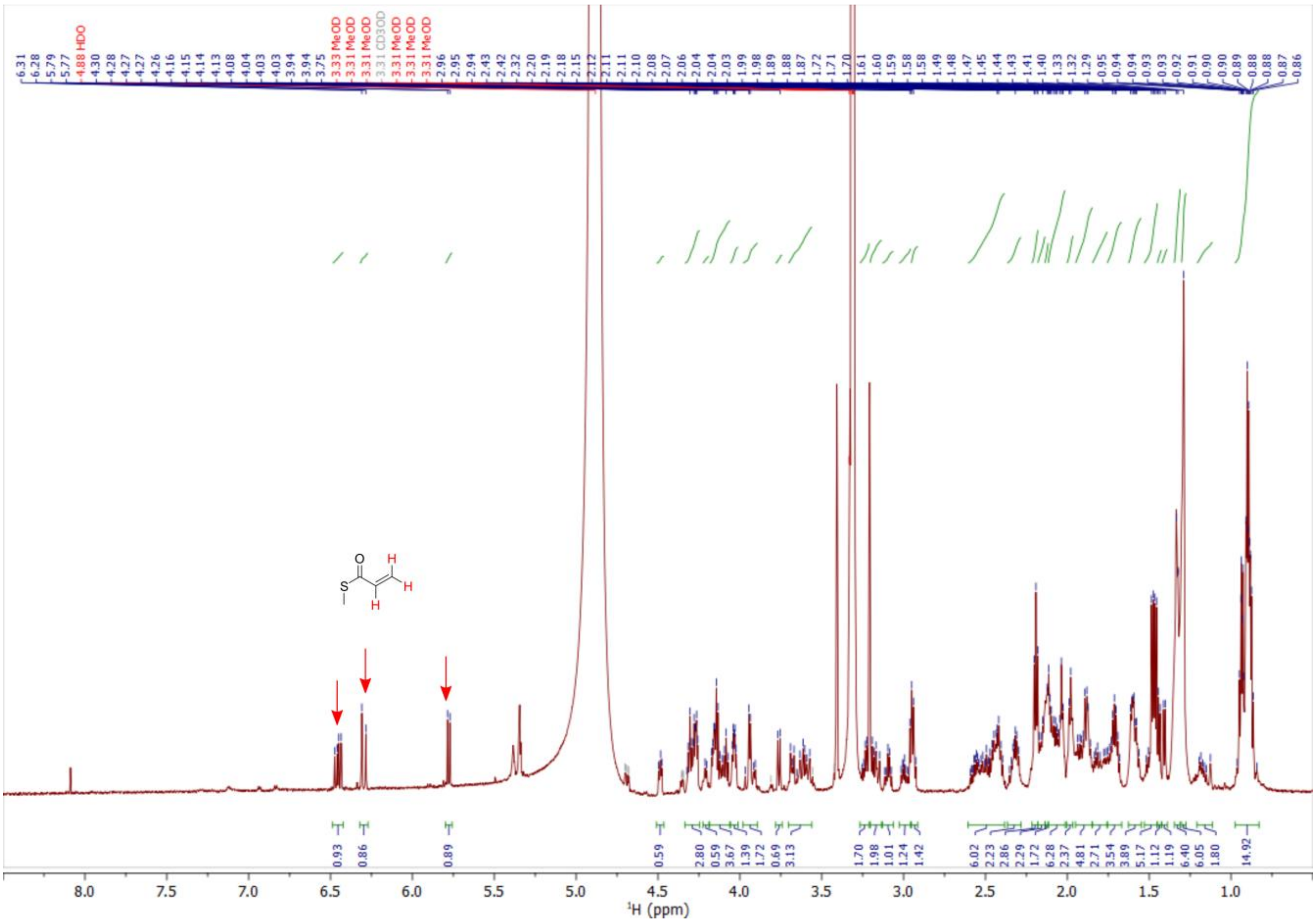

Figure S70: ^{1}H-NMR (700 MHz, MeOH-d_4, 295 K) of **P1A35_thioacrylate** with a retention time of 24.1 min. Hydrogen-deuterium exchange was performed prior to the measurement.

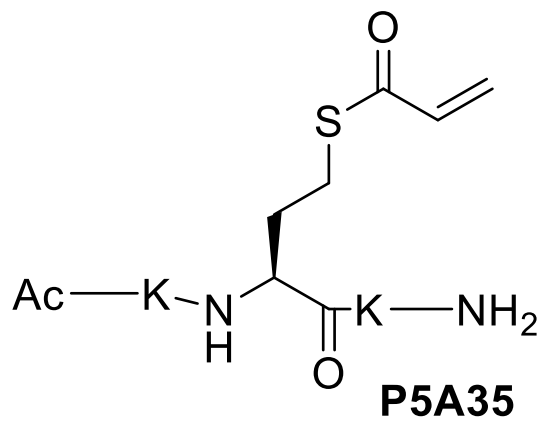

P5A35

Chemical Formula: $C_{21}H_{38}N_6O_5S$

Exact Mass: 486.26

Molecular Weight: 486.63

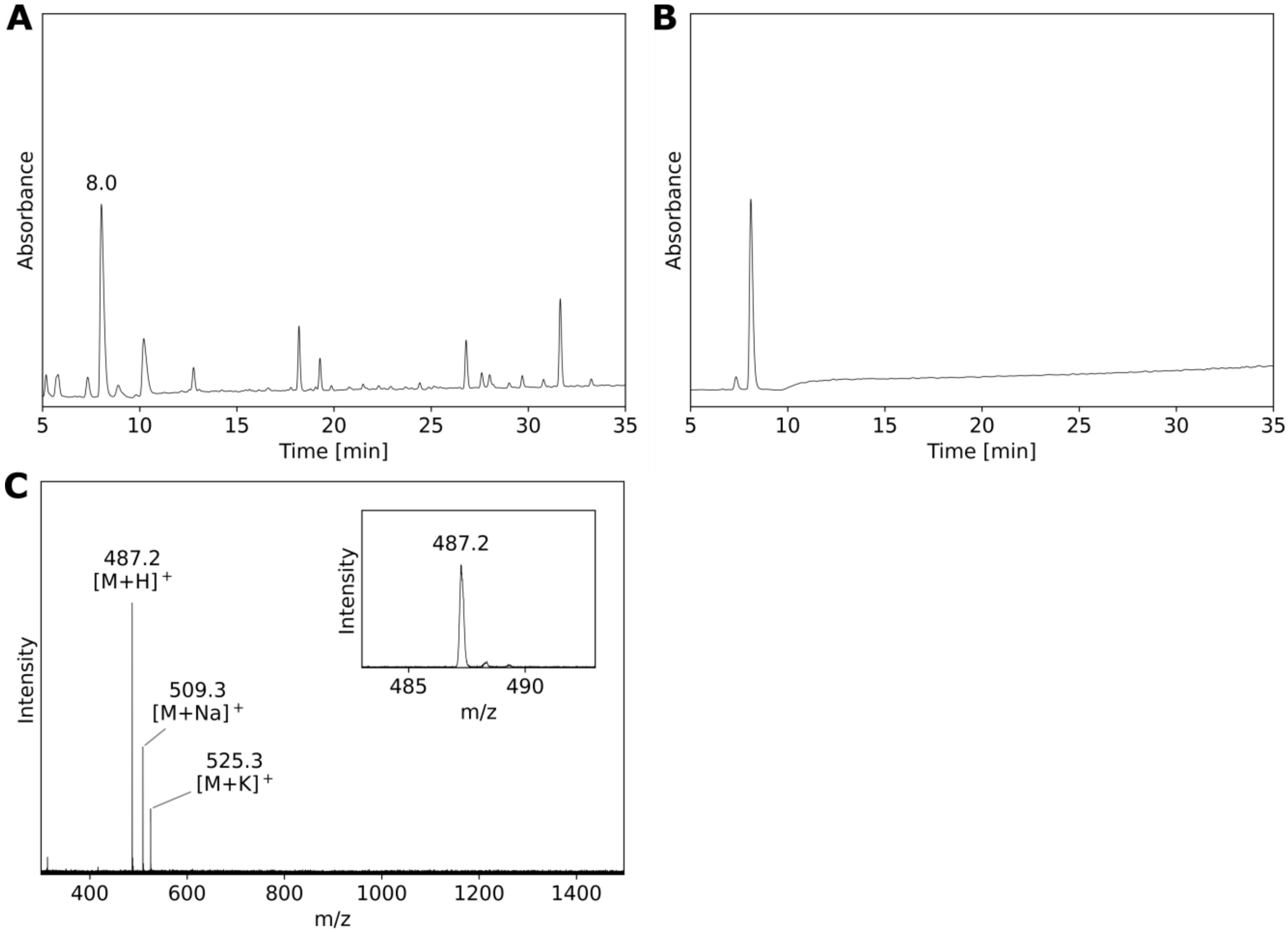

Figure S71: **P5A35** A) Analytical HPLC (220 nm) of crude peptide. HPLC Gradients are listed in Section 2.3.5 Table S3. B) Analytical HPLC (220 nm) of pure peptide. C) MALDI-TOF MS of pure peptide. Calculated mass: $[M+H]^+$ 487.3, $[M+Na]^+$ 509.3, $[M+K]^+$ 525.2.

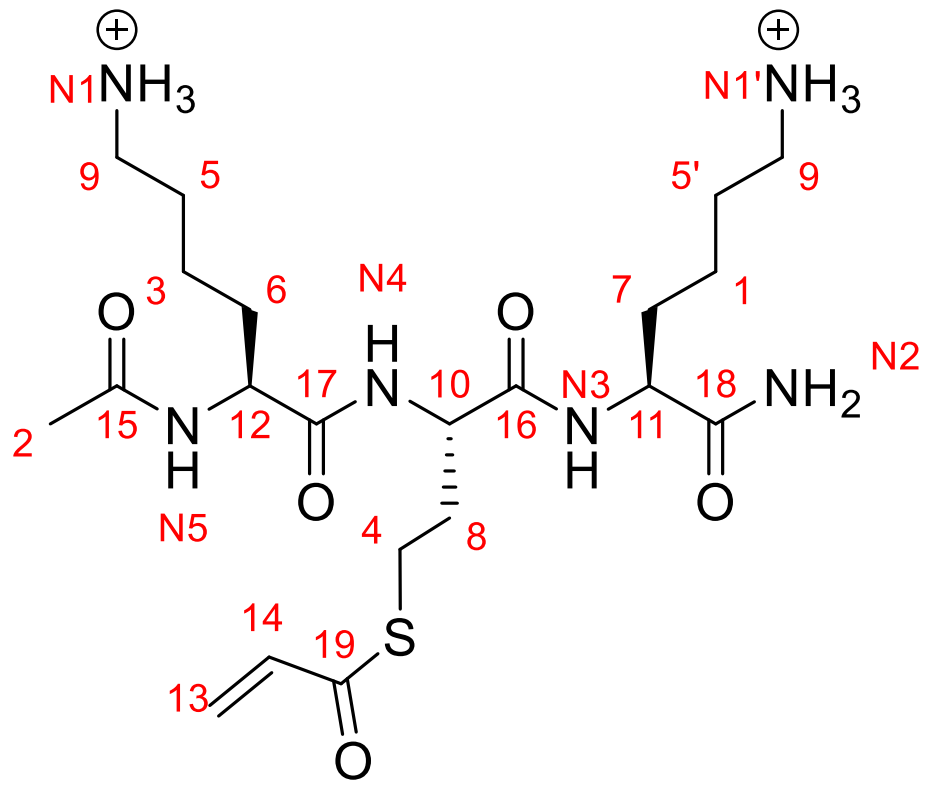

^{1}H NMR (700 MHz, DMSO-d_6, 295 K):

δ [ppm] = 8.12 (d, *J* = 7.4 Hz, 1H, H-N5), 8.08-8.05 (m, 1H, H-N4), 7.86 (d, *J* = 7.9 Hz, 1H, H-N3), 7.74-7.71 (m, 6H, H-N1), 7.32 (s, 1H, H-N2), 7.06 (s, 1H, H-N2), 6.49 (dd, J= 17.1 Hz, 10.4 Hz, 1H, H-14), 6.24 (d, J= 17.2, 1H, H-13), 5.83 (d, J= 10.5 Hz, 1H, H-13) 4.31-4.27 (m, 1H, H-13), 4.17-4.14 (m, 1H, H-12), 4.13-4.09 (m, 1H, H-11), 2.97-2.88 (m, 2H, H-4), 2.79-2.72 (m, 4H, H-9), 1.98-1.93 (m, 1H, H-8), 1.86 (s, 3H, H-2), 1.85-1.80 (m, 1H, H-8), 1.68-1.61 (m, 2H, H-7/6), 1.54-1.48 (m, 6H, H-7/6/5), 1.35-1.23 (m, 4H, H-3/1).

^{13}C NMR (176 MHz, DMSO-d_6, 295 K):

δ [ppm] = 189.6 (C-19), 173.3 (C-18), 172.1 (C-17), 170.6 (C-16), 169.8 (C-15), 134.9 (C-14), 127.6 (C-13), 52.9 (C-12), 52.3 (C-11), 52.0 (C-10), 38.71 (C-9), 31.7 (C-8), 31.3 (C-7), 31.1 (C-6), 26.71 (C-5), 26.67 (C-5), 24.7 (C-4), 22.6 (C-3), 22.4 (C-2), 22.3 (C-1).

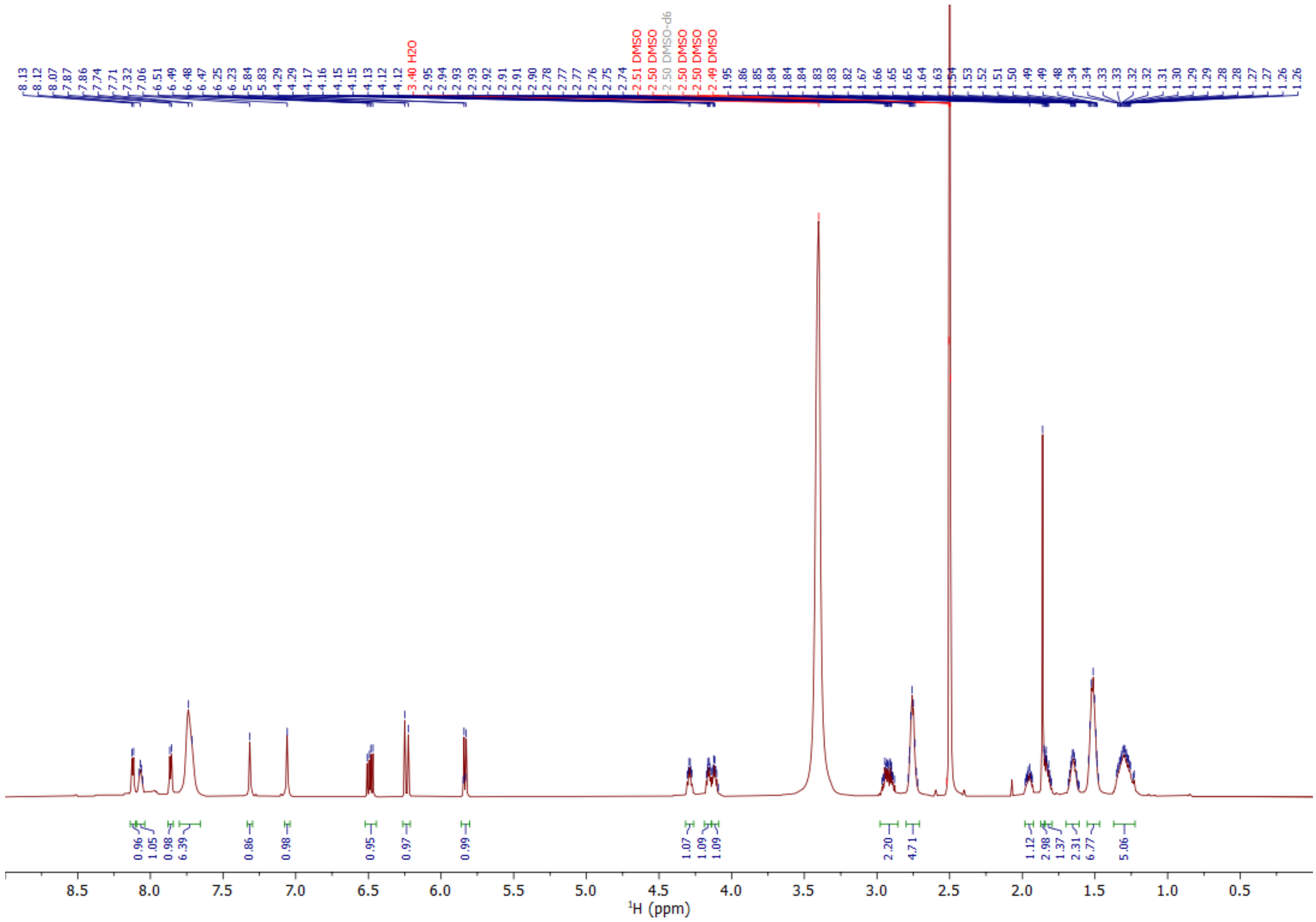

Figure S72: ^{1}H NMR spectrum (700 MHz, DMSO-d_6, 295 K) of **P5A35**.

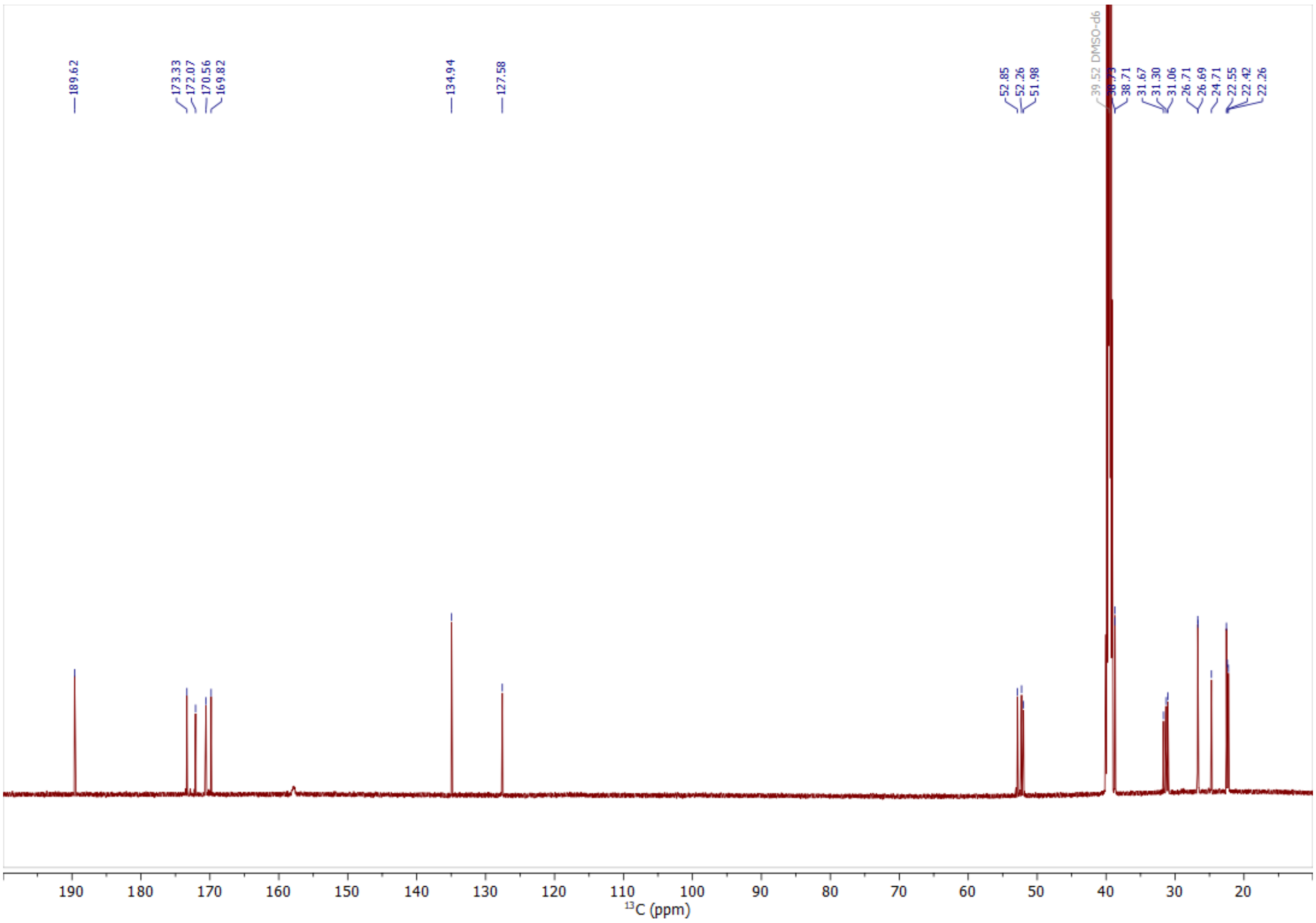

Figure S73: 295 K) of **P5A35**.

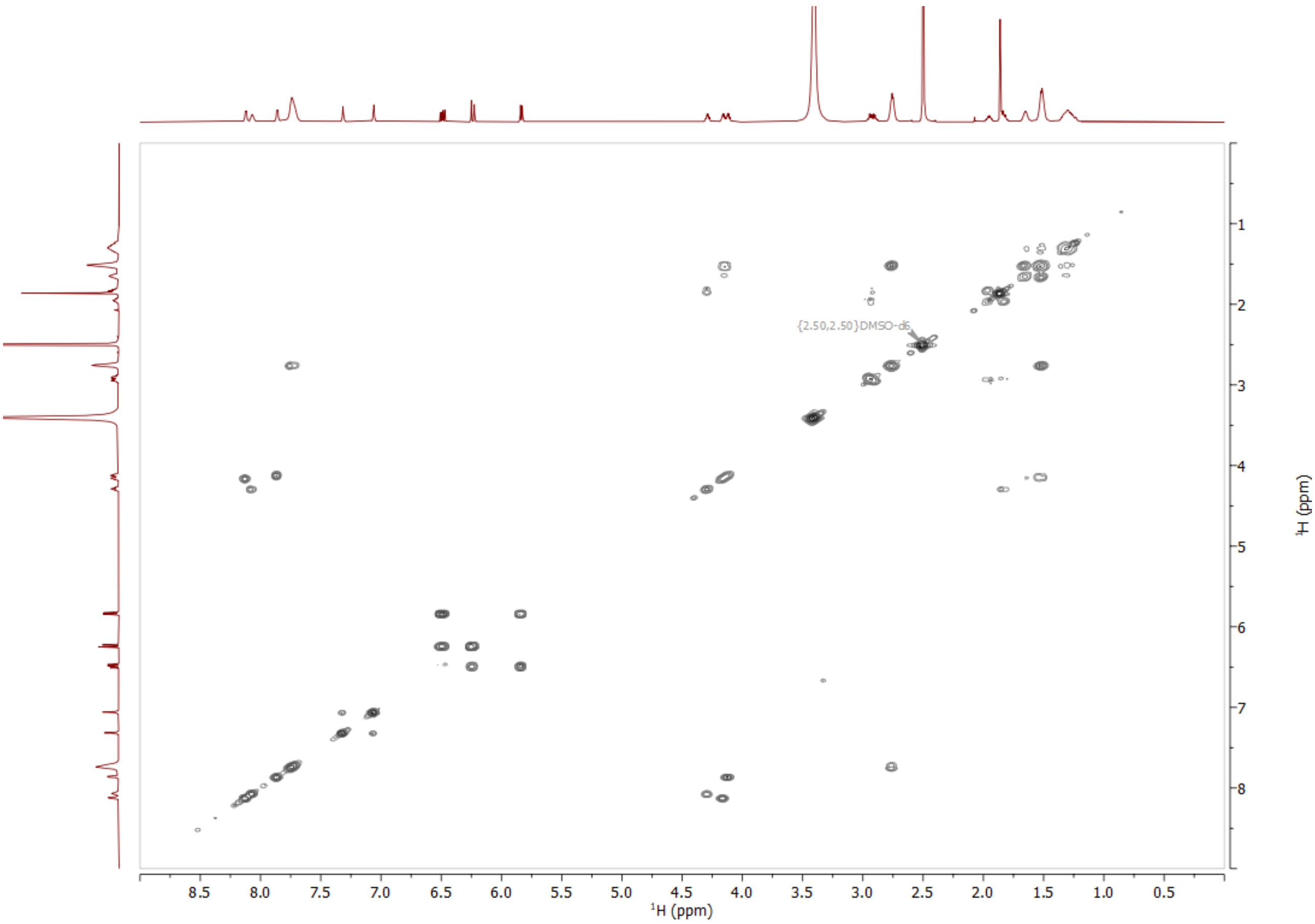

Figure S74: ^{1}H-^{1}H COSY spectrum (700 MHz, 700 MHz, DMSO-d_6, 295 K) of **P5A35**.

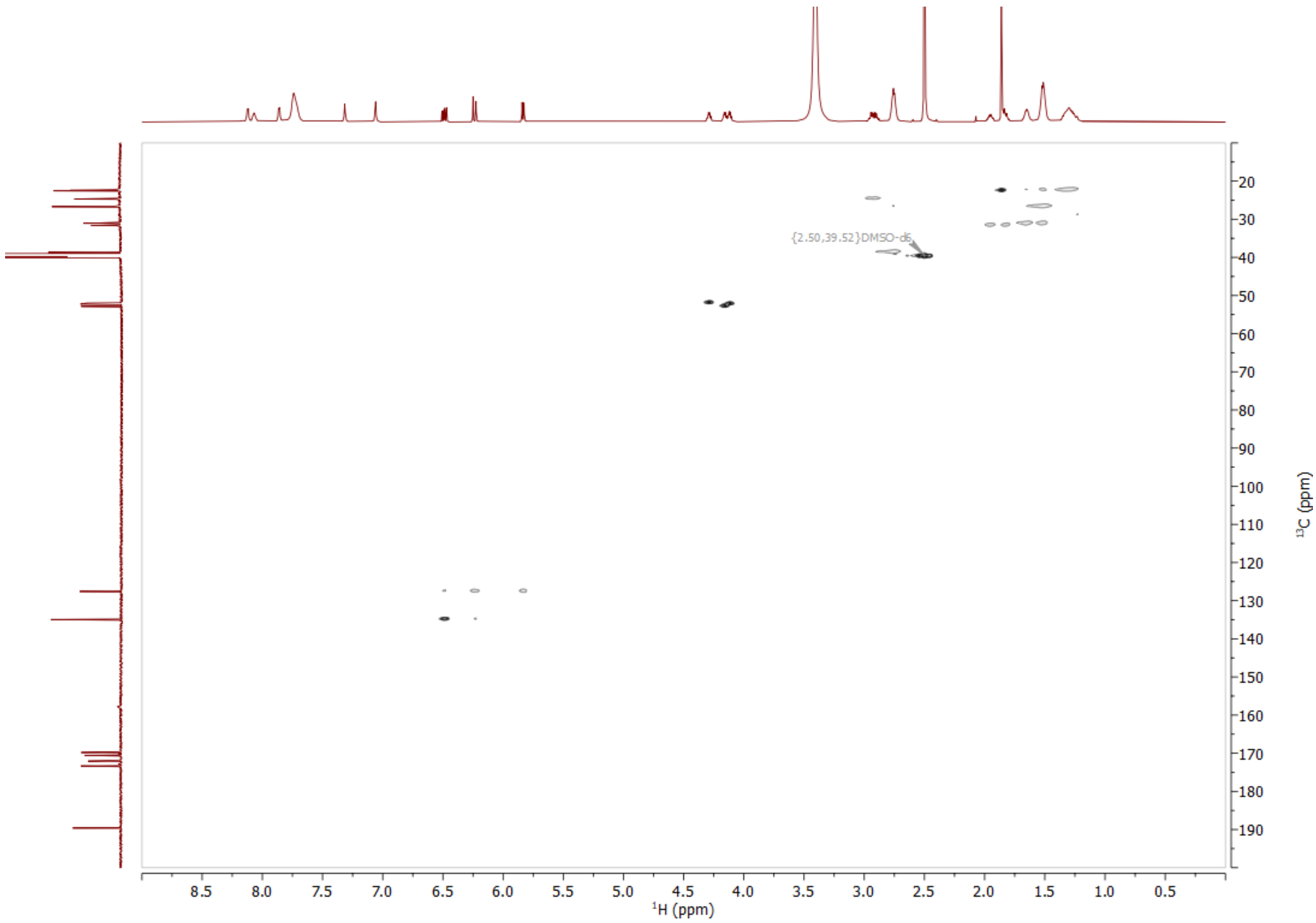

Figure S75: ^{1}H-^{13}C HSQC spectrum (700 MHz, 176 MHz, DMSO-d_6, 295 K) of **P5A35**.

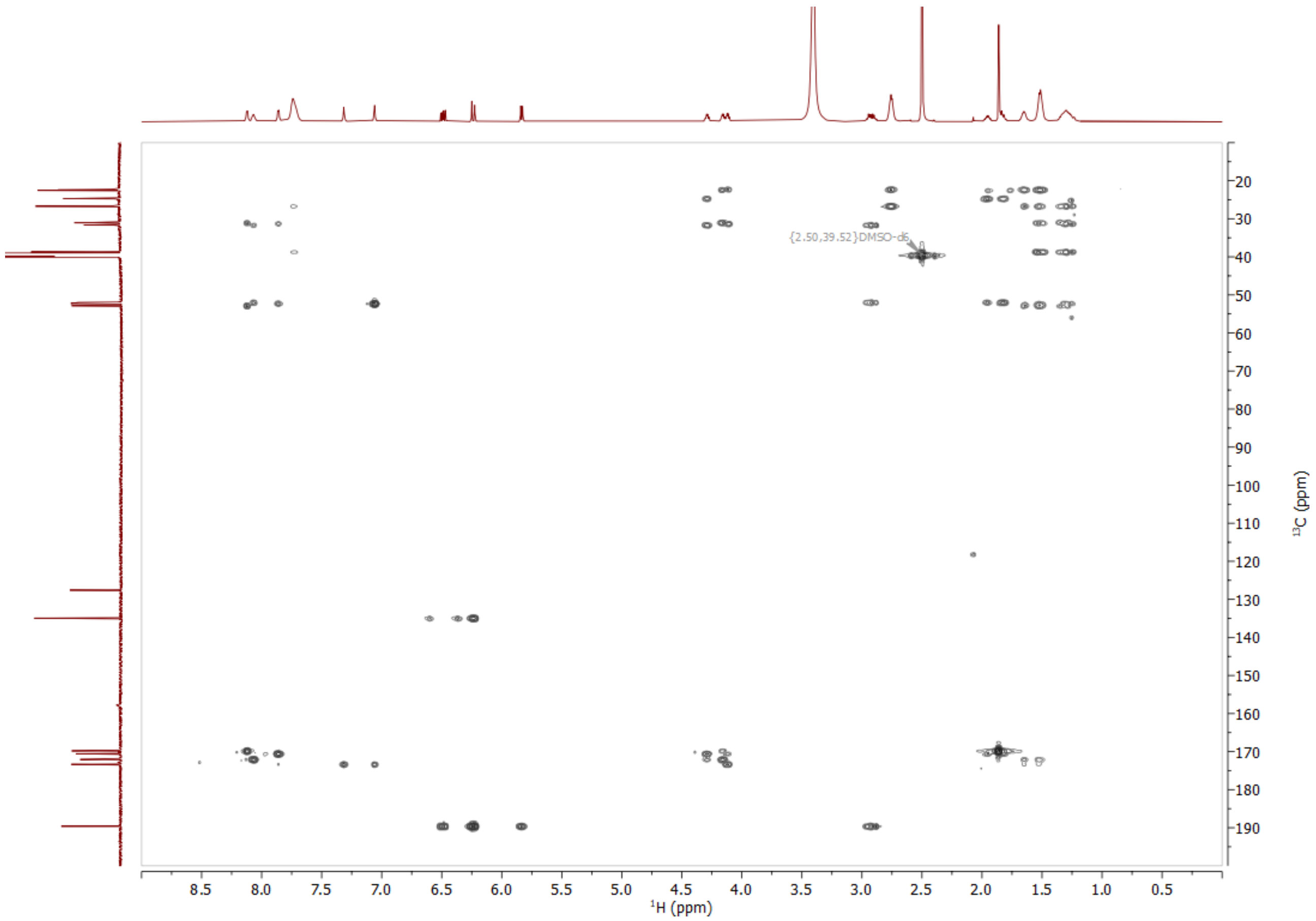

Figure S76: ^{1}H-^{13}C HMBC spectrum (700 MHz, 176 MHz, DMSO-d_6, 295 K) of **P5A35**.

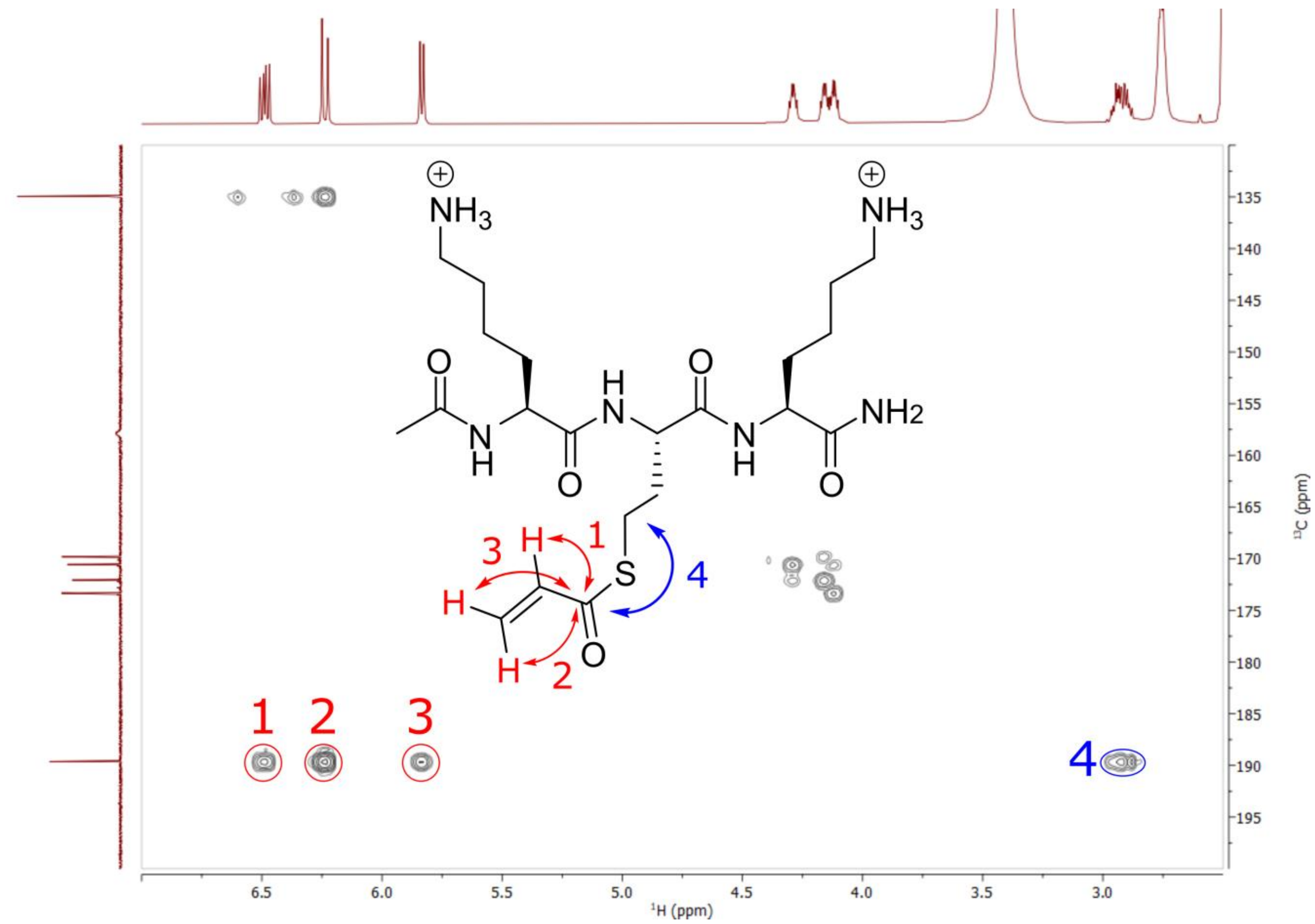

Figure S77: Extract from the ^{1}H-^{13}C HMBC spectrum (700 MHz, 176 MHz, DMSO-d_6, 295 K) of **P5A35** showing the cross-peaks relevant for isomer determination.

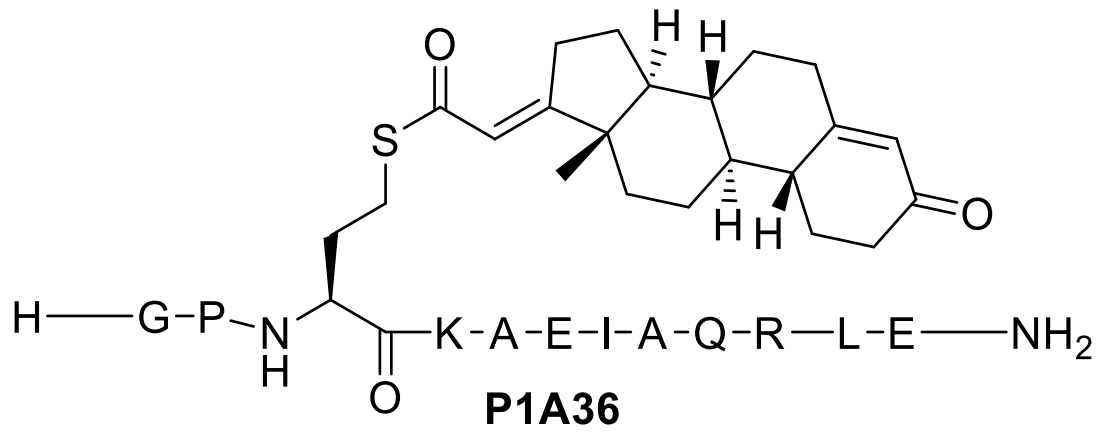

P1A36

Chemical Formula: $C_{76}H_{122}N_{18}O_{19}S$
Exact Mass: 1622.9
Molecular Weight: 1624.0

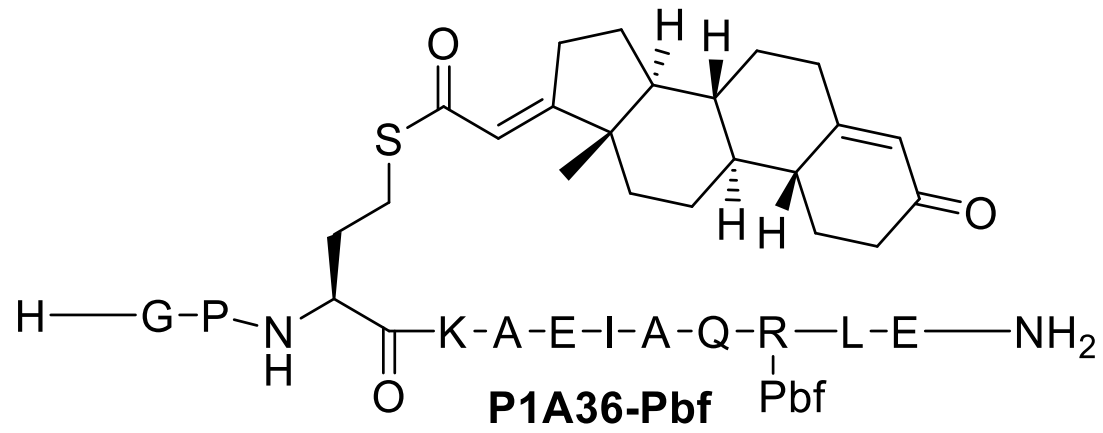

P1A36-Pbf

Chemical Formula: $C_{89}H_{138}N_{18}O_{22}S_2$
Exact Mass: 1875.0
Molecular Weight: 1876.3

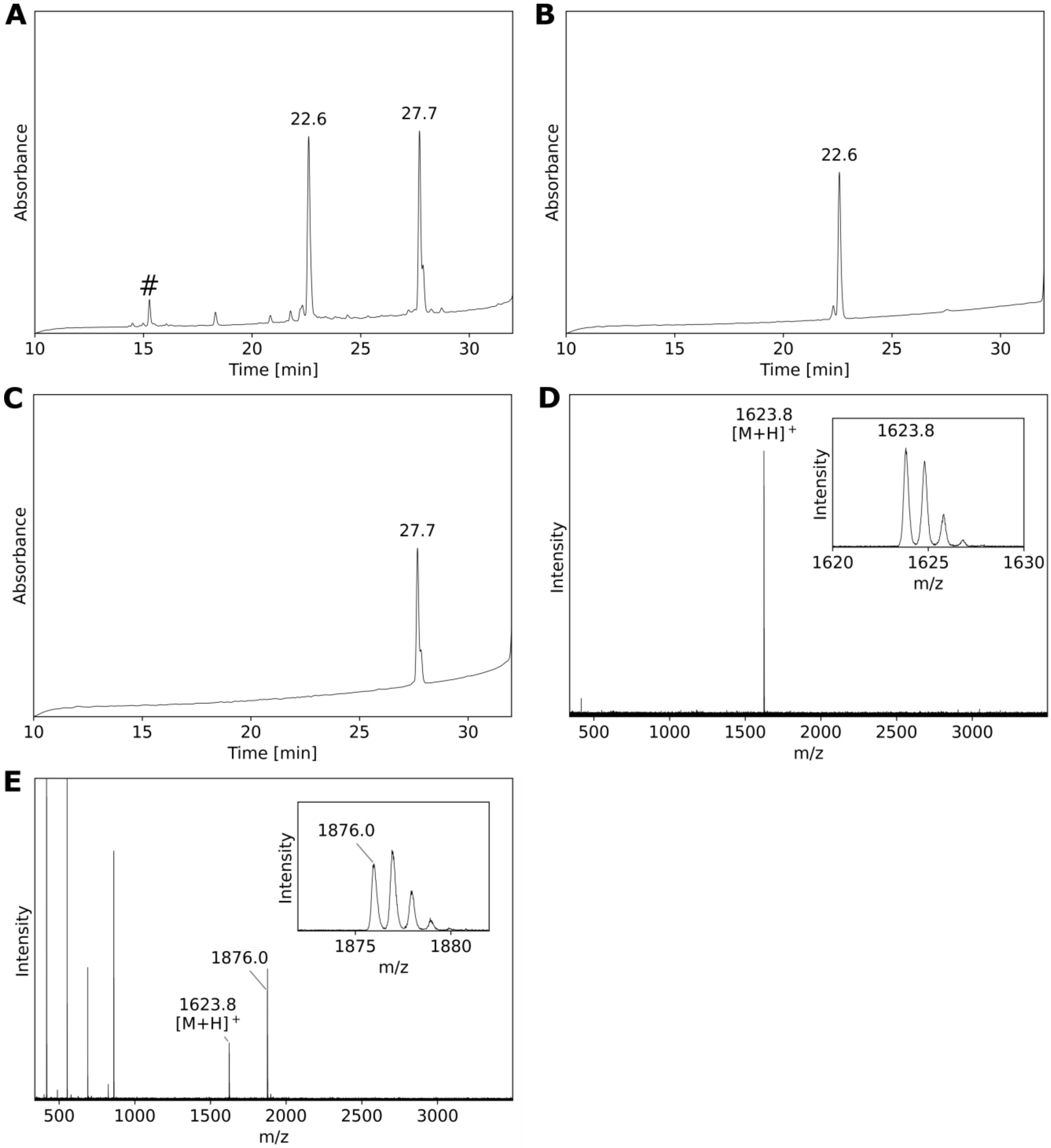

Figure S78: **P1A36** A) Analytical HPLC (220 nm) of crude peptide. HPLC Gradients are listed in Section 2.3.5 Table S3. # Peptide without homoserine. B-C) Analytical HPLC (220 nm) of pure peptide. D) MALDI-TOF MS of purified peptide with a retention time of 22.6 min. E) MALDI-TOF MS of purified peptide with a retention time of 27.7 min. Calculated mass for **P1A36**: $[M+H]^+$ 1623.9. Calculated mass for **P1A36-Pbf**: $[M+H]^+$ 1876.0.

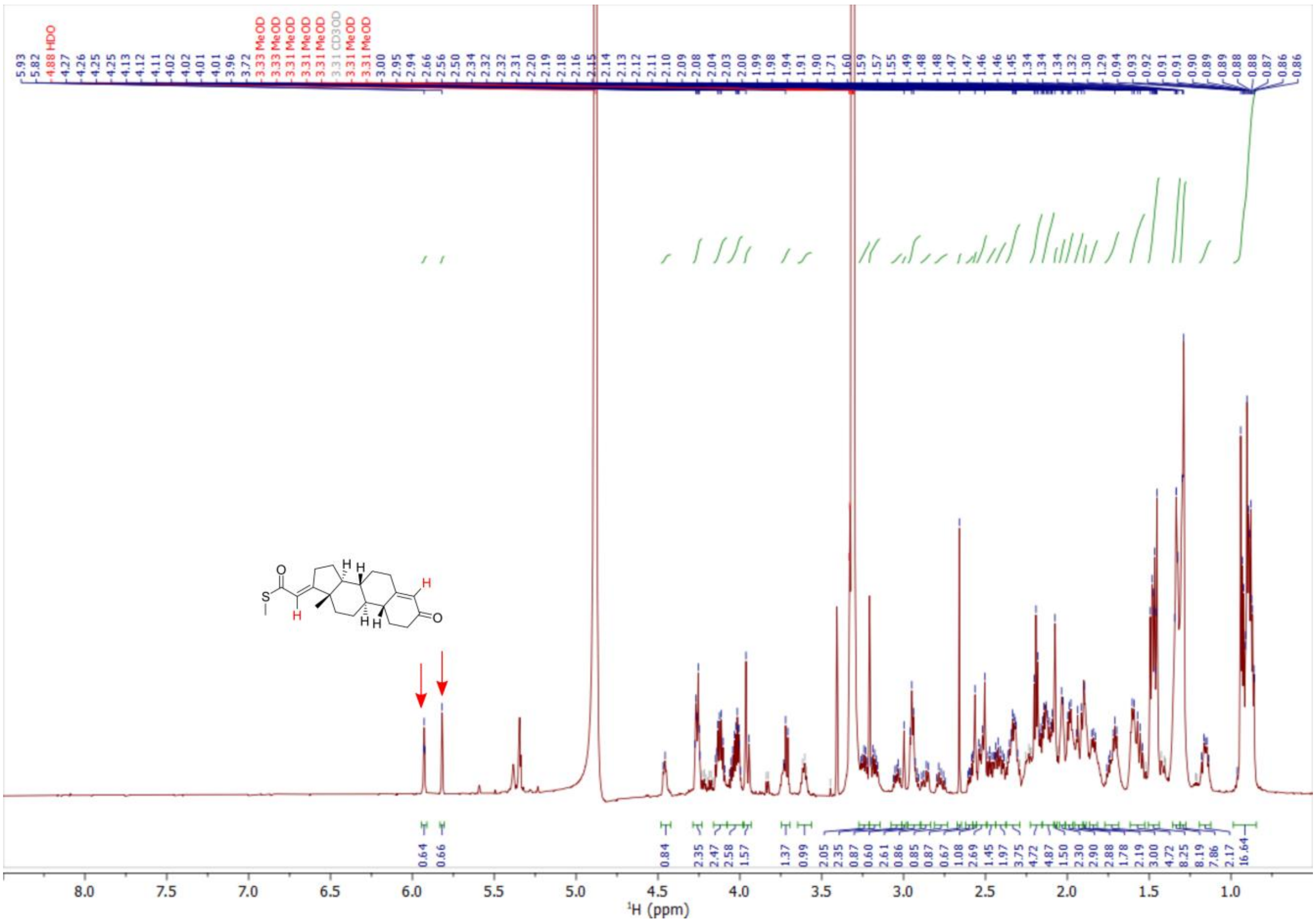

Figure S79: ^{1}H-NMR (700 MHz, MeOH-d_4, 295 K) of **P1A36**. Hydrogen-deuterium exchange was performed prior to the measurement.

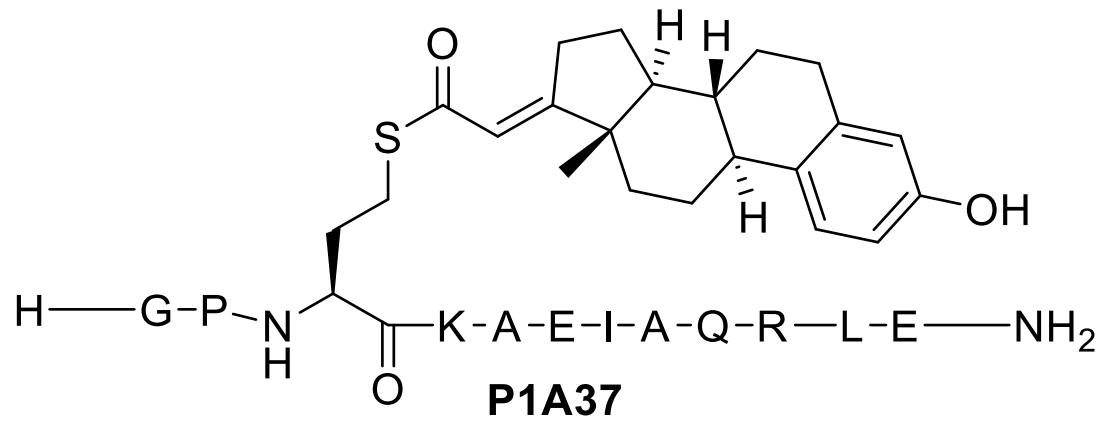

P1A37

Chemical Formula: $C_{76}H_{120}N_{18}O_{19}S$

Exact Mass: 1620.9

Molecular Weight: 1622.0

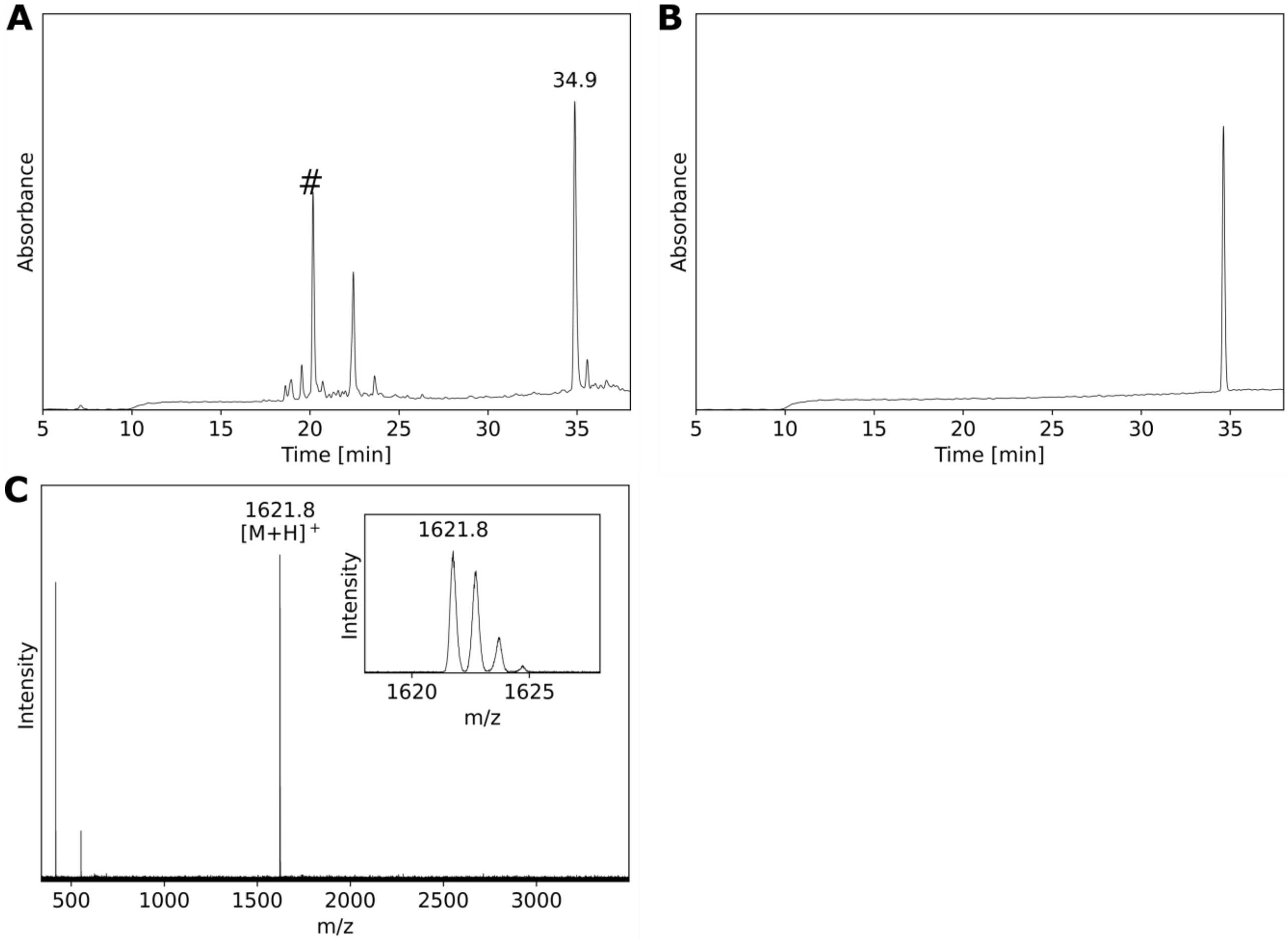

Figure S80: **P1A37** A) Analytical HPLC (220 nm) of crude peptide. HPLC Gradients are listed in Section 2.3.5 Table S3. # Peptide without homoserine. B) Analytical HPLC (220 nm) of pure peptide. C) MALDI-TOF MS of pure peptide. Calculated mass: $[M+H]^+$ 1621.9.

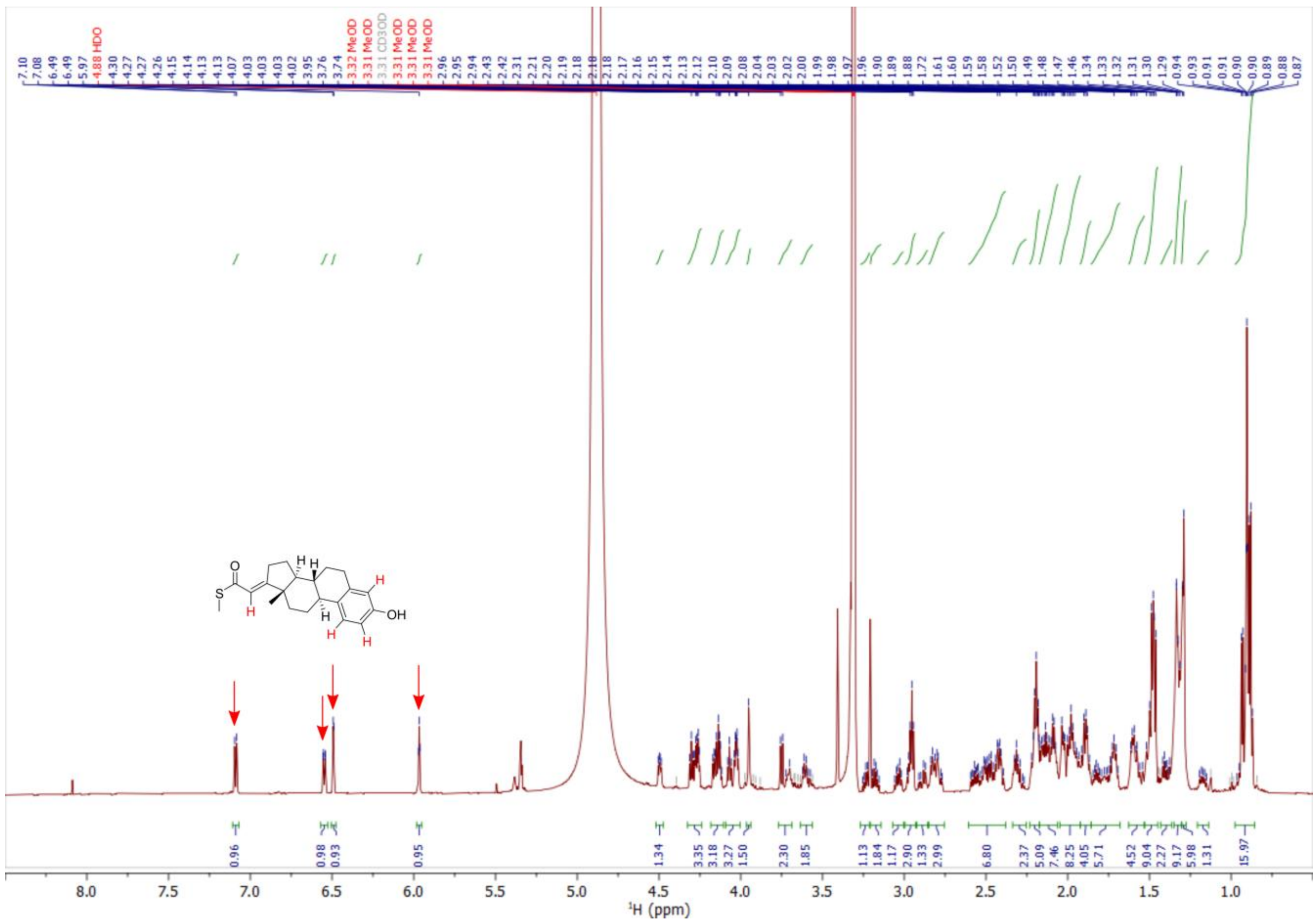

Figure S81: ^{1}H-NMR (700 MHz, MeOH-d_4, 295 K) of **P1A37**. Hydrogen-deuterium exchange was performed prior to the measurement.

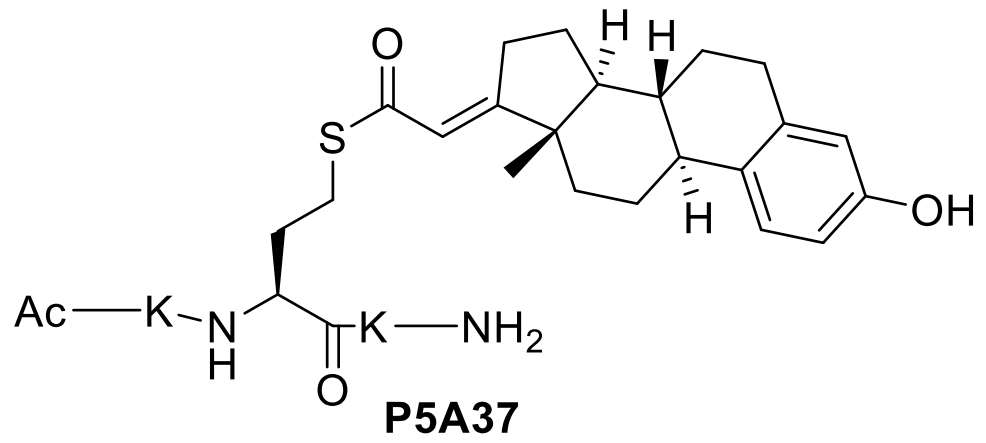

P5A37
Chemical Formula: $C_{38}H_{58}N_6O_6S$
Exact Mass: 726.4
Molecular Weight: 727.0

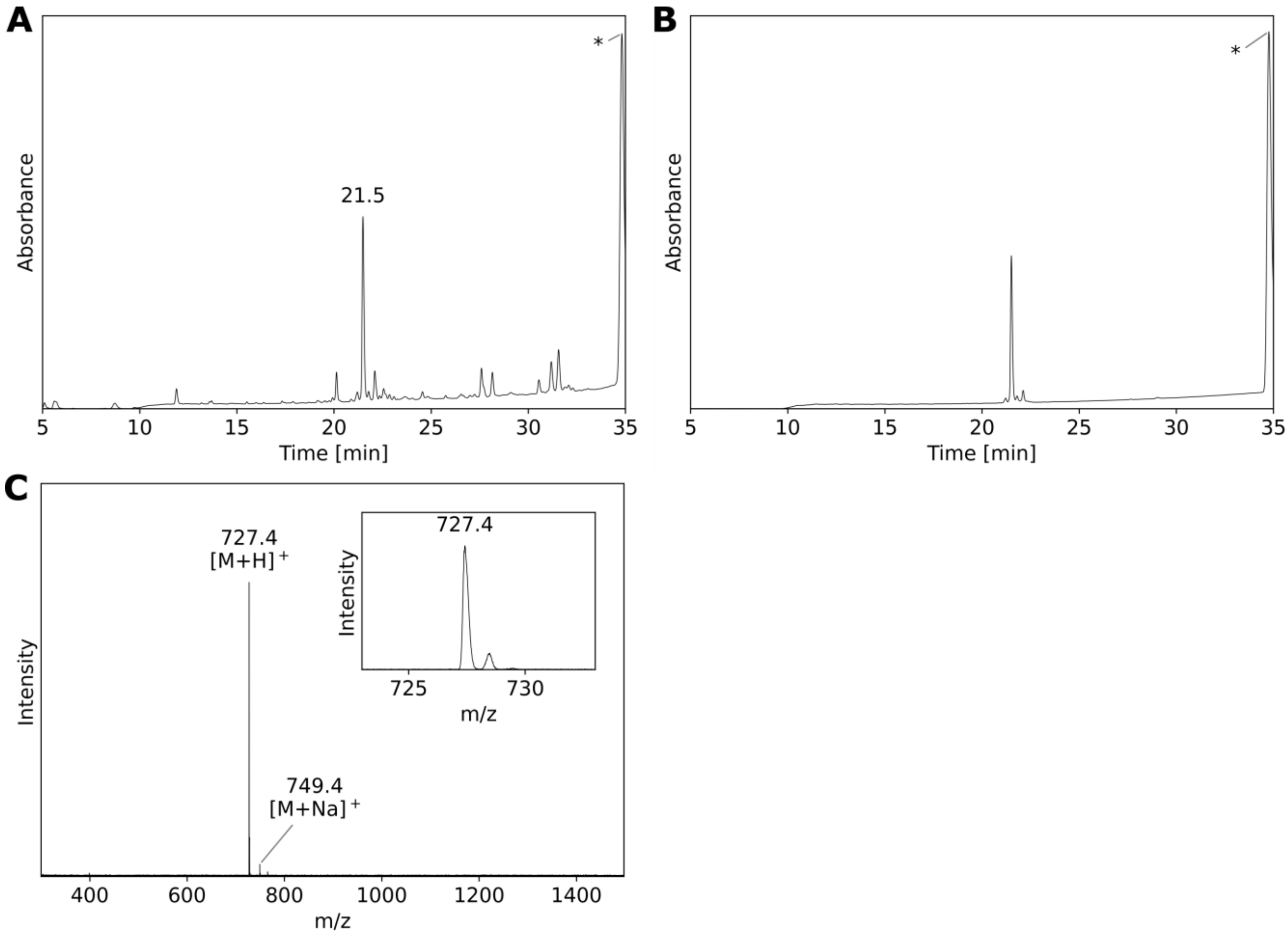

Figure S82: **P5A37** A) Analytical HPLC (220 nm) of crude peptide. HPLC Gradients are listed in Section 2.3.5 Table S3. * Carryover peak. B) Analytical HPLC (220 nm) of pure peptide. C) MALDI-TOF MS of pure peptide. Calculated mass: $[M+H]^+$ 727.4, $[M+Na]^+$ 749.4.

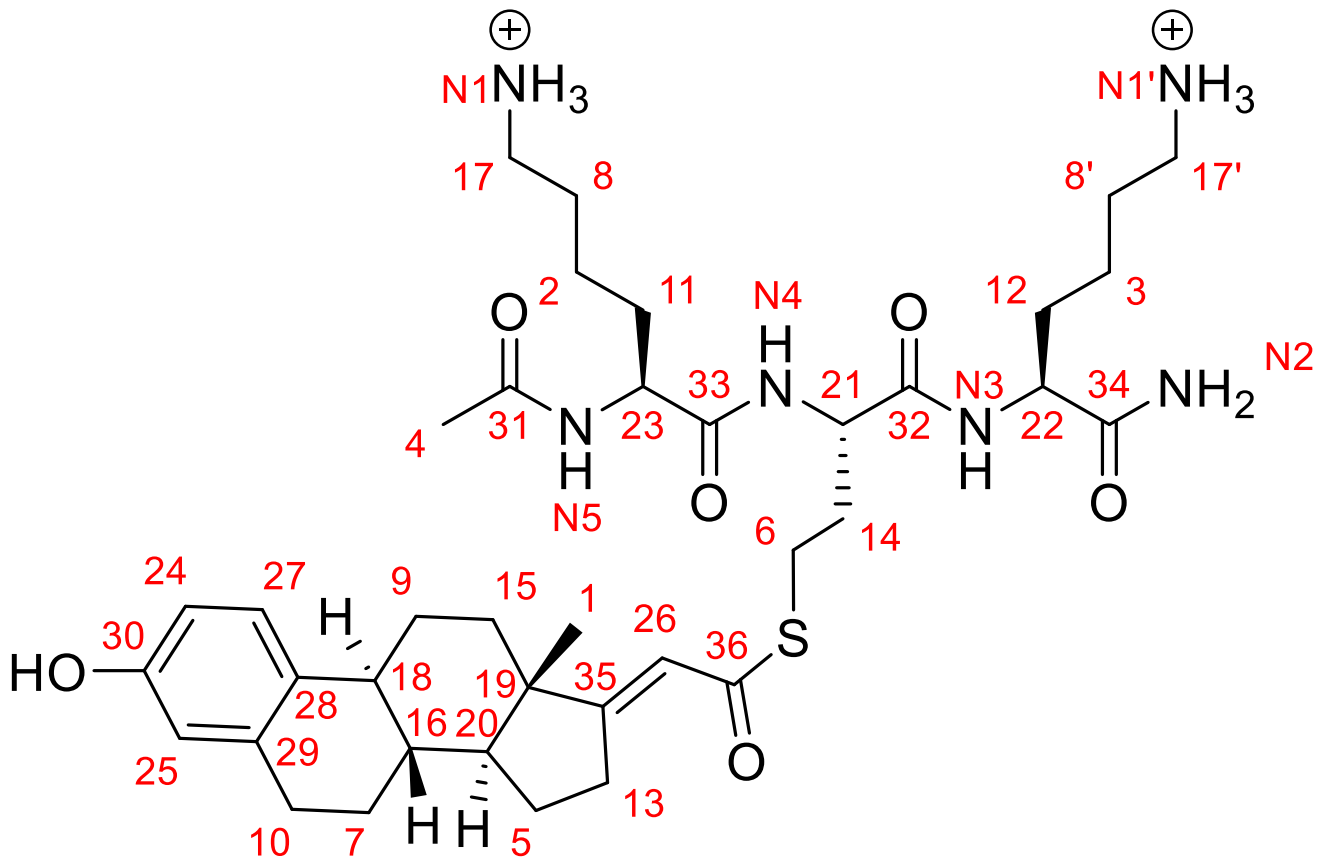

^{1}H NMR (700 MHz, DMSO-d_6, 295 K):

δ [ppm] = 9.05 (s, 1H, OH30), 8.12 (d, *J* = 7.4 Hz, 1H, H-N5), 8.08-8.05 (m, 1H, H-N4), 7.85 (d, *J* = 8.0 Hz, 1H, H-N3), 7.75-7.73 (m, 6H, H-N1), 7.32 (s, 1H, H-N2), 7.06-7.04 (m, 2H, H-27/H-N2), 6.51 (dd, *J* = 8.4, 2.6 Hz, 1H, H-24) 6.44 (d, *J* = 2.6 Hz, 1H, H-25) 5.93 (t, *J* = 2.6 Hz, 1H, H-26) 4.29-4.26 (m, 1H, H-21), 4.18-4.15 (m, 1H, H-23), 4.14-4.11 (m, 1H, H-22), 2.91-2.87 (m, 1H, H-6), 2.85-2.80 (m, 2H, H-13/6), 2.78-2.69 (m, 7H, H-13/10), 2.36-2.33 (m, 1H, H-7), 2.16-2.12 (m, 1H, H-7), 1.98-1.95 (m, 1H, H-15), 1.94-1.90 (m, 1H, H-14), 1.86 (s, 3H, H-4), 1.85-1.77 (m, 3H, H-14/9/5), 1.69-1.63 (m, 2H, H-12/11), 1.55-1.48 (m, 6H, H-17/12/11), 1.43-1.38 (m, 4H, H-16/15/7/5) 1.35-1.22 (m, 6H, H-20/9/3/2).

^{13}C NMR (176 MHz, DMSO-d_6, 295 K):

δ [ppm] = 187.9 (C-36), 173.8 (C-35),173.4 (C-34),172.1 (C-33), 170.7 (C-32),169.8 (C-31), 155.1 (C-30), 137.1 (C-29), 130.0 (C-28), 126.1 (C-27), 115.2 (C-26), 115.0 (C-25), 112.8 (C-24), 52.8 (C-23), 52.3 (C-22), 52.1 (C-21), 52.0 (C-20), 46.3 (C-19), 43.4 (C-18), 38.74 (C-17), 38.71 (C-17), 38.2 (C-16), 34.7 (C-15), 32.0 (C-14), 31.4 (C-13), 31.3 (C-12), 31.1 (C-11), 29.2 (C-10), 27.3 (C-9), 26.72 (C-8), 26.69 (C-8), 26.2 (C-7), 24.7 (C-6), 23.8 (C-5), 22.6 (C-4), 22.4 (C-3), 22.3 (C-2), 18.2 (C-1).

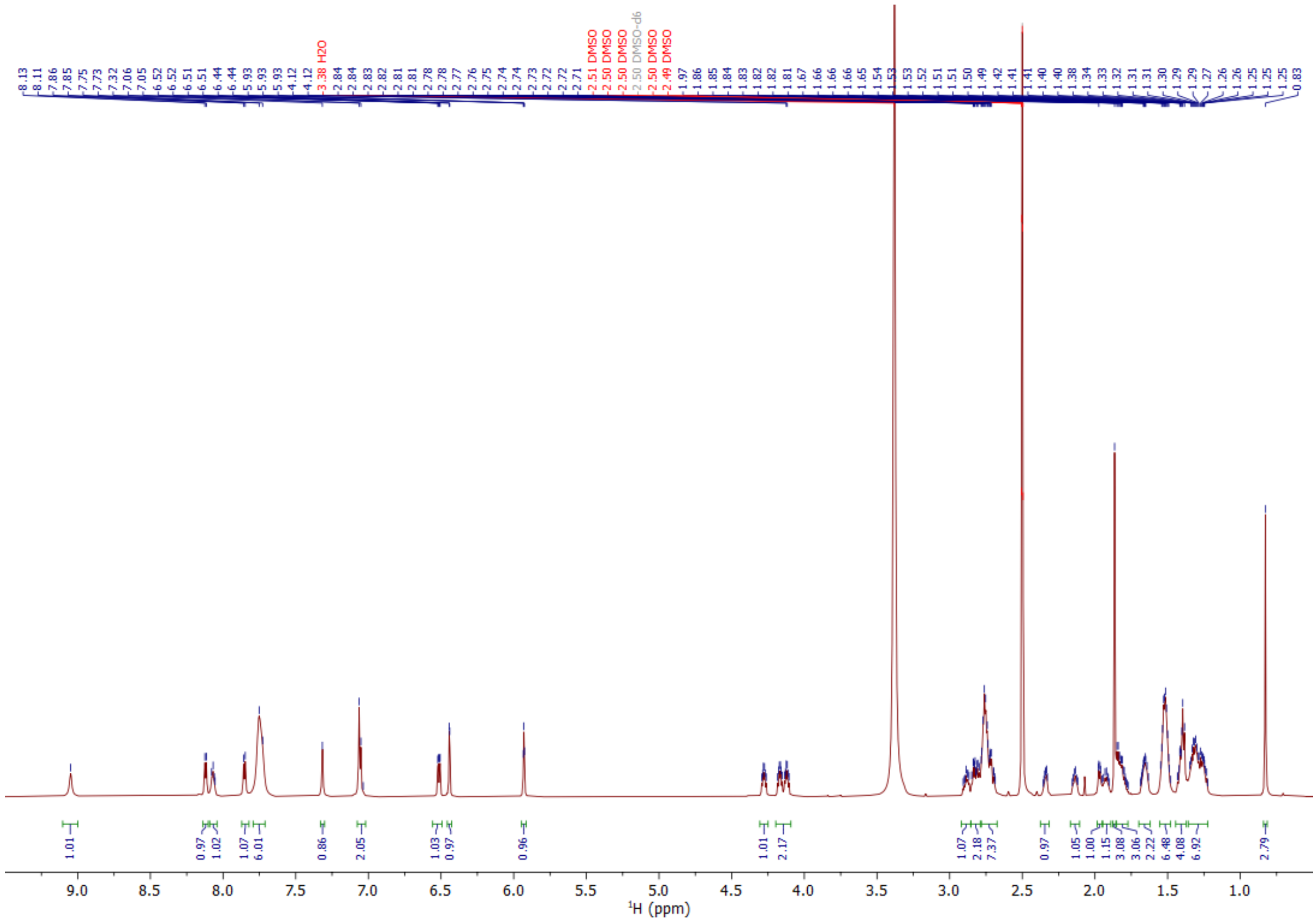

Figure S83: ^{1}H NMR spectrum (700 MHz, DMSO-d_6, 295 K) of **P5A37**.

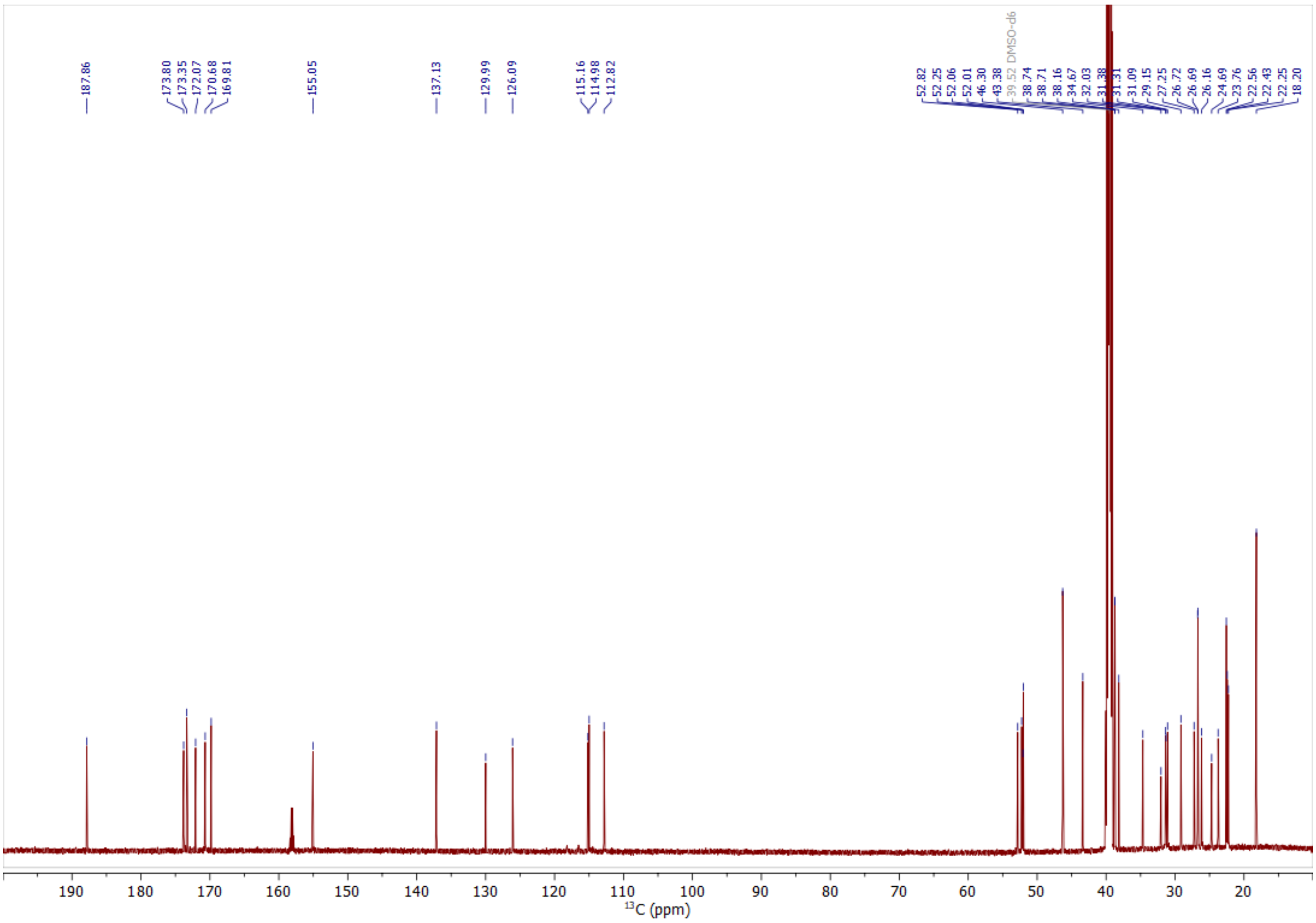

Figure S84: ^{13}C NMR spectrum (176 MHz, DMSO-d_6, 295 K) of **P5A37**.

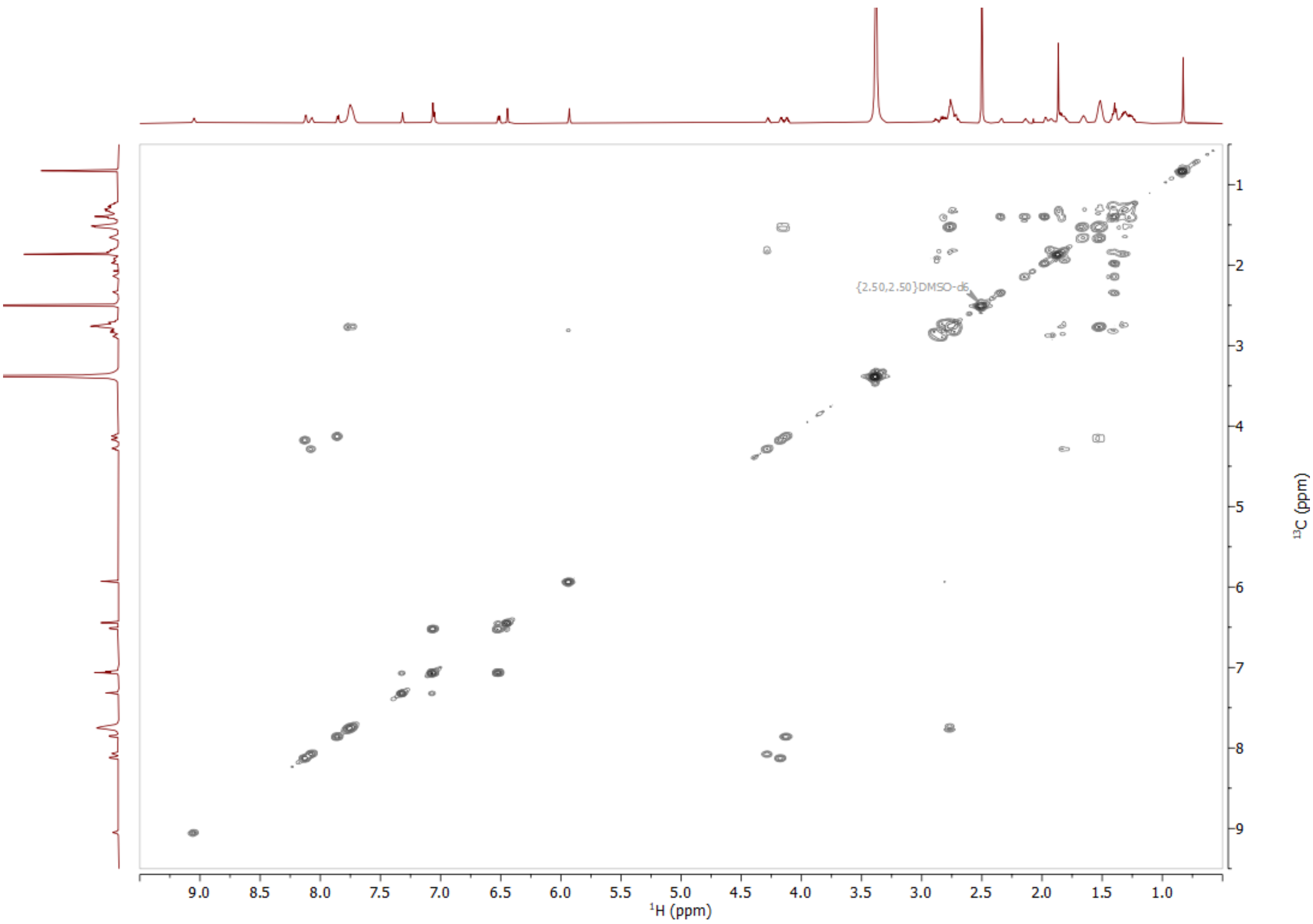

Figure S85: ^{1}H-^{1}H COSY spectrum (700 MHz, 700 MHz, DMSO-d_6, 295 K) of **P5A37**.

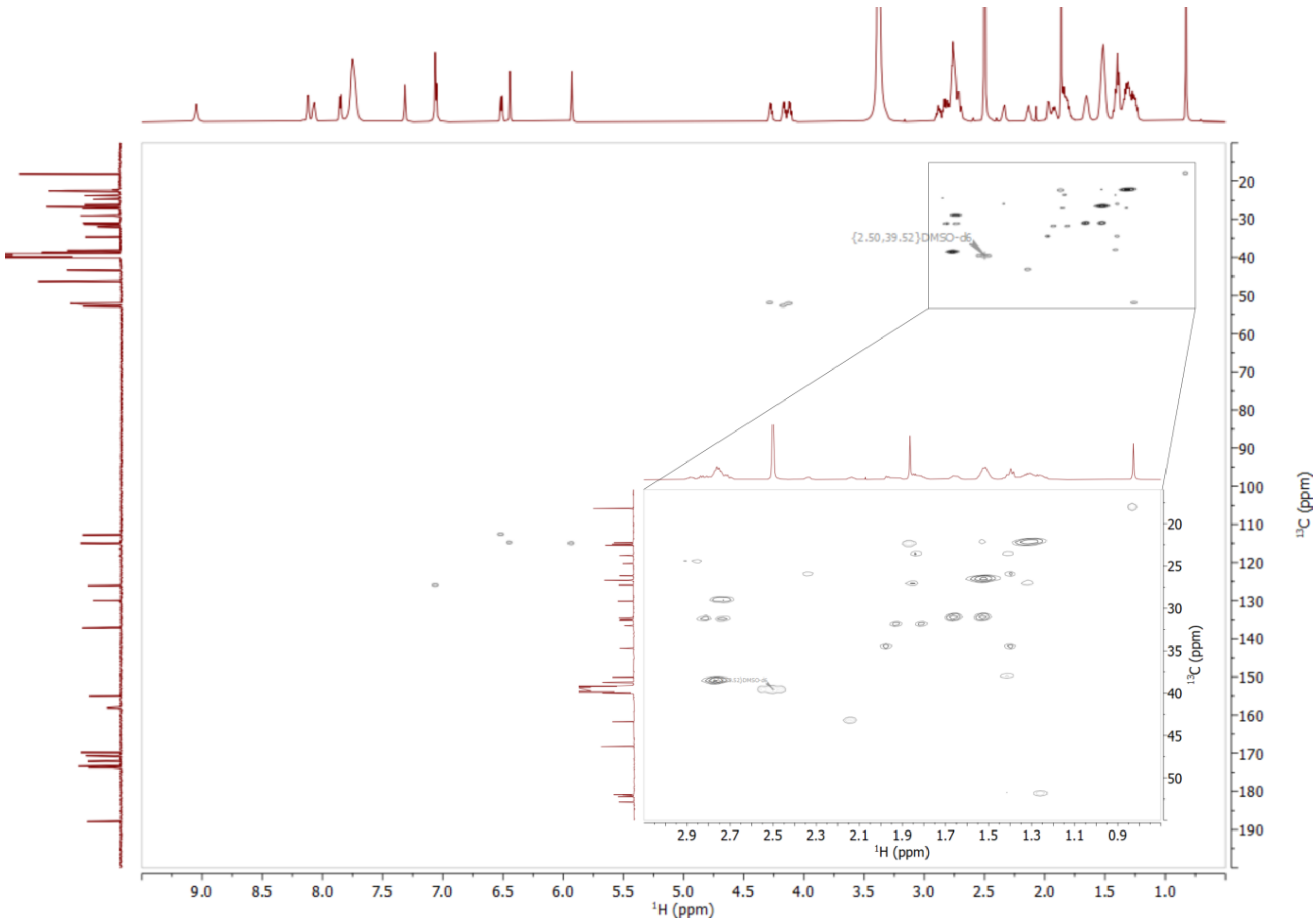

Figure S86: ^{1}H-^{13}C HSQC spectrum (700 MHz, 176 MHz, DMSO-d_6, 295 K) of **P5A37**.

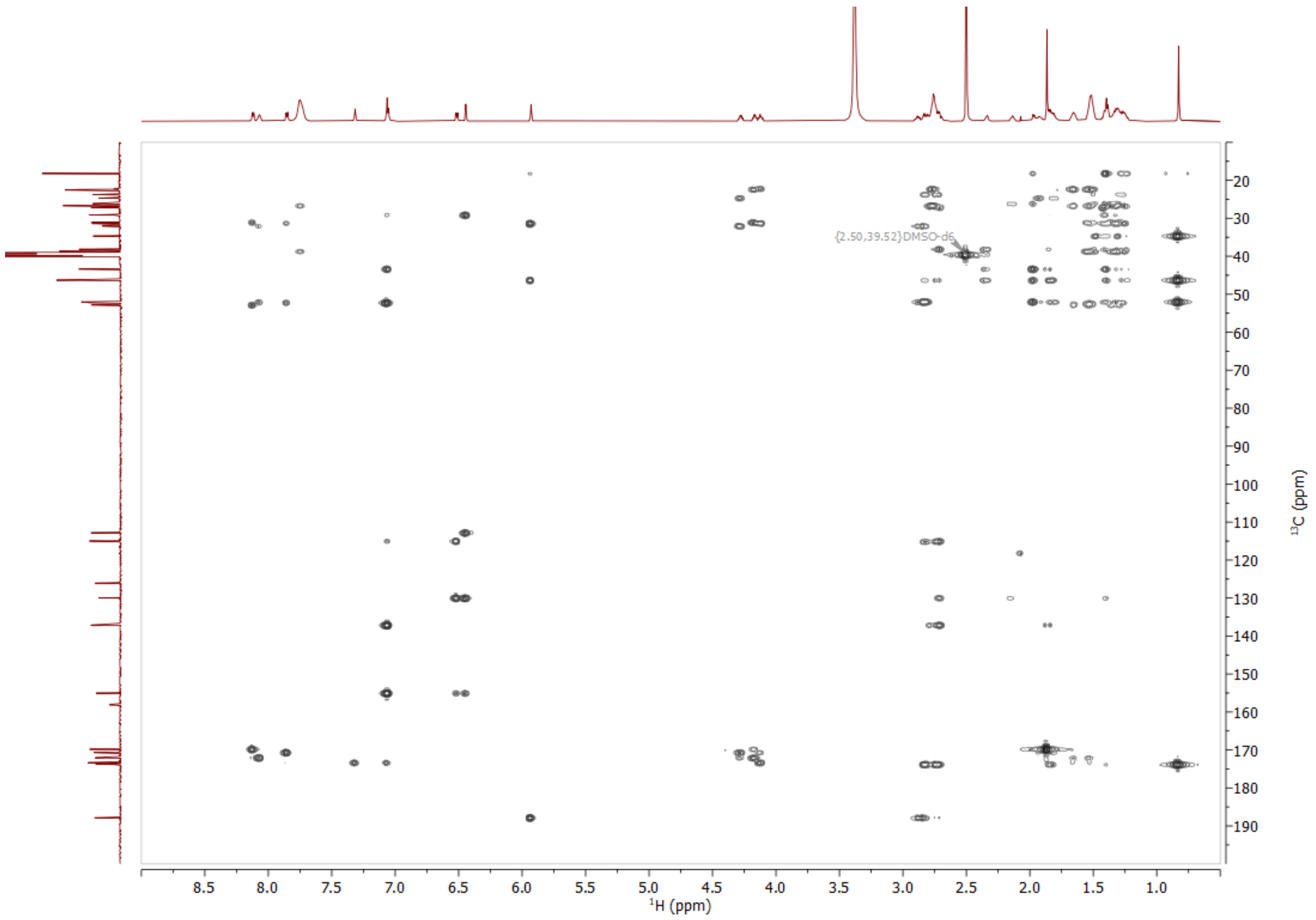

Figure S87: ^1H-^{13}C HMBC spectrum (700 MHz, 176 MHz, DMSO-d_6, 295 K) of **P5A37**.

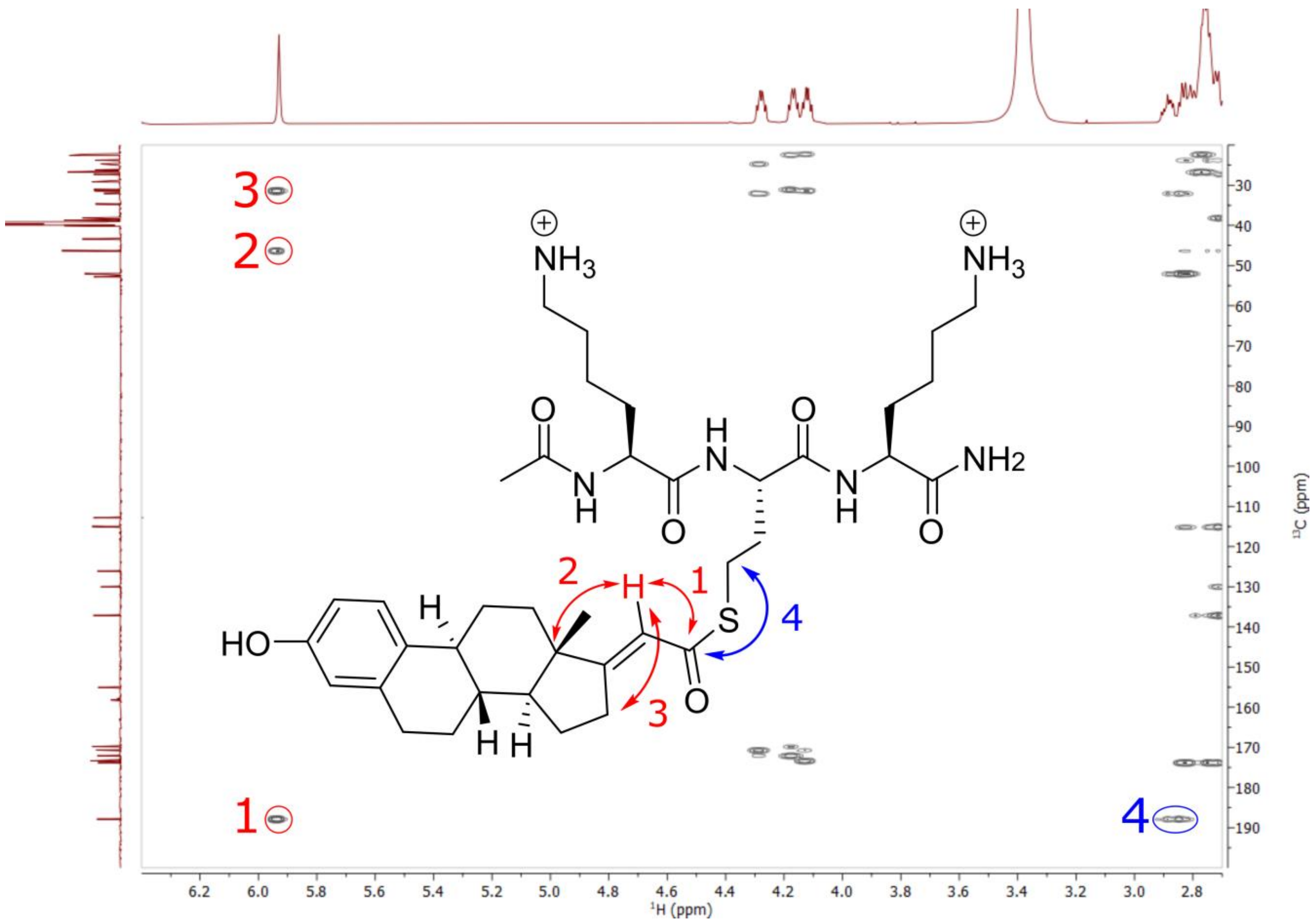

Figure S88: Extract from the ^1H-^{13}C HMBC spectrum (700 MHz, 176 MHz, DMSO-d_6, 295 K) of **P5A37** showing the cross-peaks relevant for isomer determination.

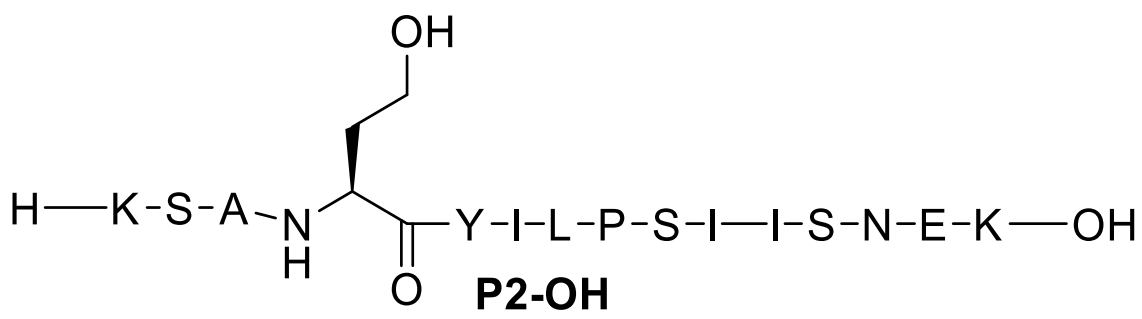

P2-OH

Chemical Formula: $C_{75}H_{126}N_{18}O_{24}$
Exact Mass: 1662.92
Molecular Weight: 1663.94

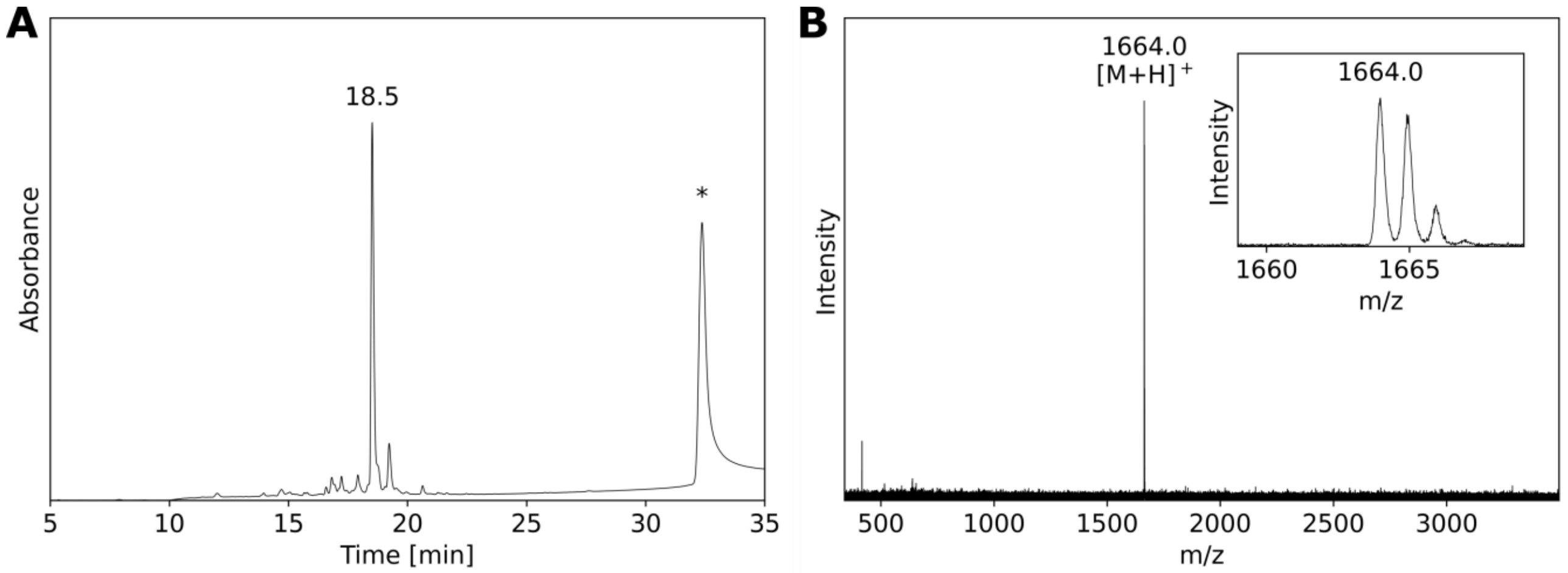

Figure S89: **P2-OH** A) Analytical HPLC (220 nm) of crude peptide. HPLC Gradients are listed in Section 2.3.5 Table S3. * Carryover peak. B) MALDI-TOF MS of crude peptide. Calculated mass: $[M+H]^+$ 1663.9.

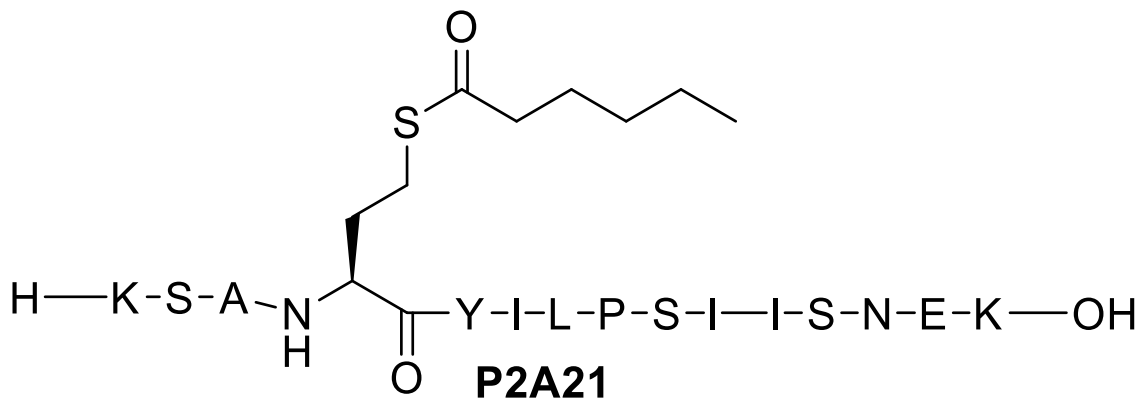

P2A21

Chemical Formula: $C_{81}H_{136}N_{18}O_{24}S$
Exact Mass: 1776.97
Molecular Weight: 1778.14

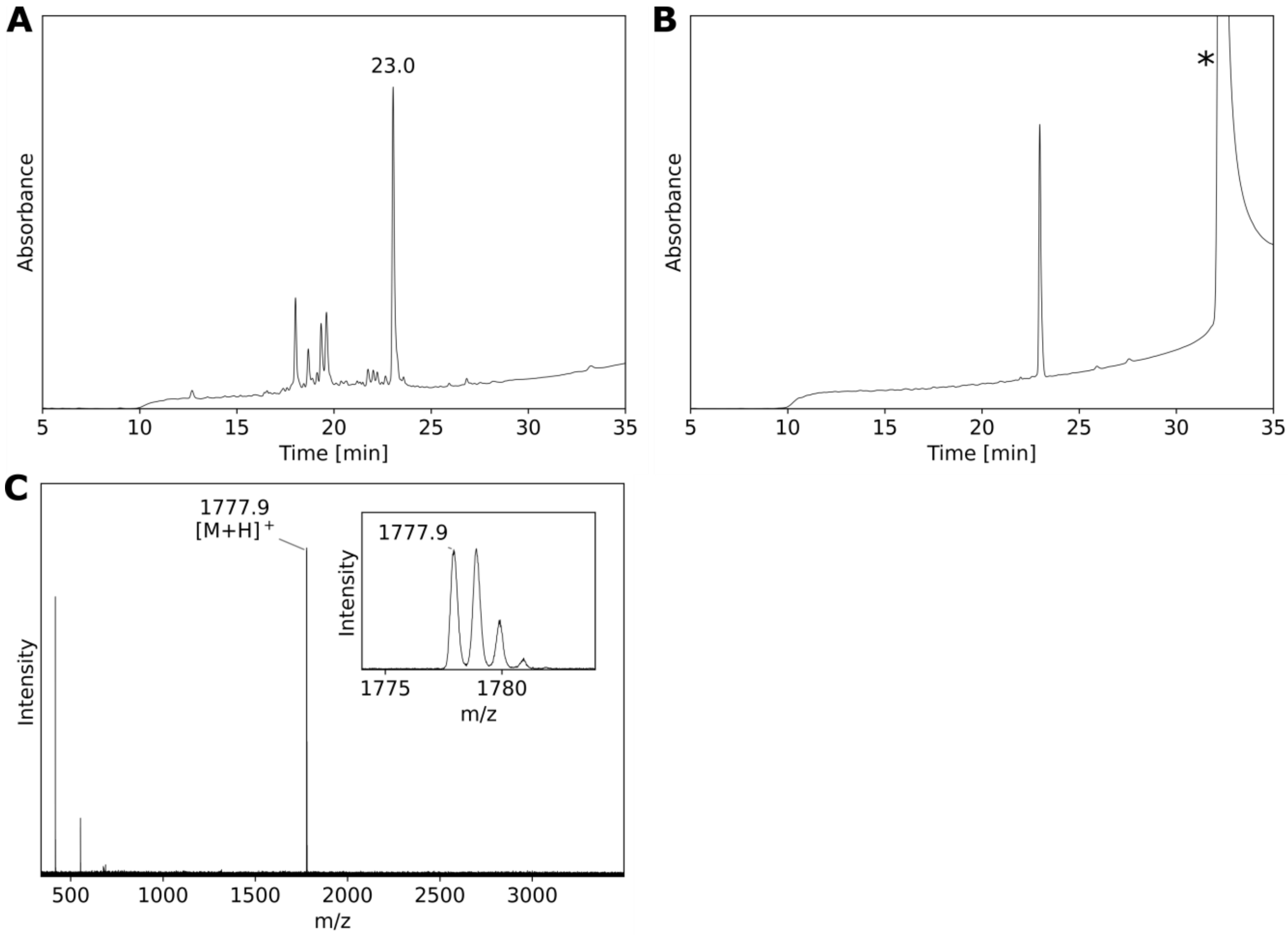

Figure S90: **P2A21** A) Analytical HPLC (220 nm) of crude peptide. HPLC Gradients are listed in Section 2.3.5 Table S3. B) Analytical HPLC (220 nm) of pure peptide. * Carryover peak. C) MALDI-TOF MS of pure peptide. Calculated mass: $[M+H]^+$ 1778.0.

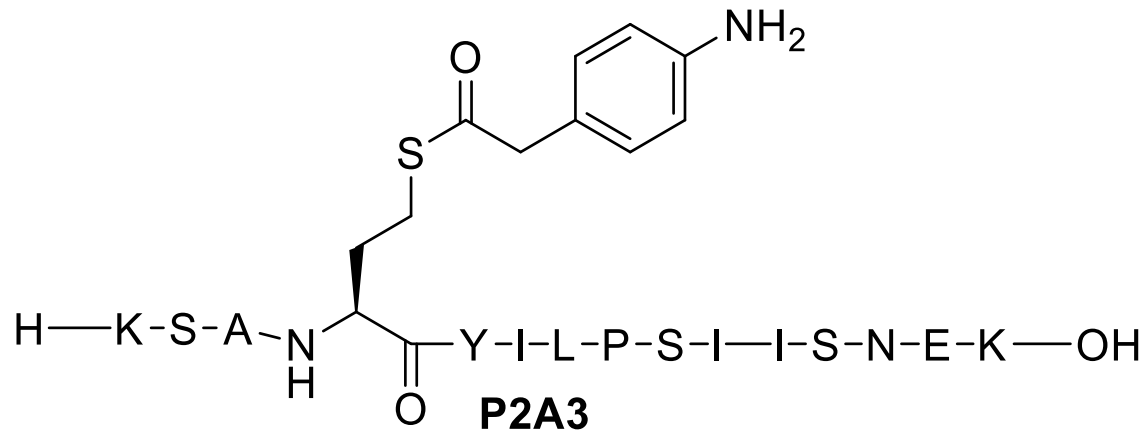

P2A3

Chemical Formula: $C_{83}H_{133}N_{19}O_{24}S$
Exact Mass: 1811.95
Molecular Weight: 1813.15

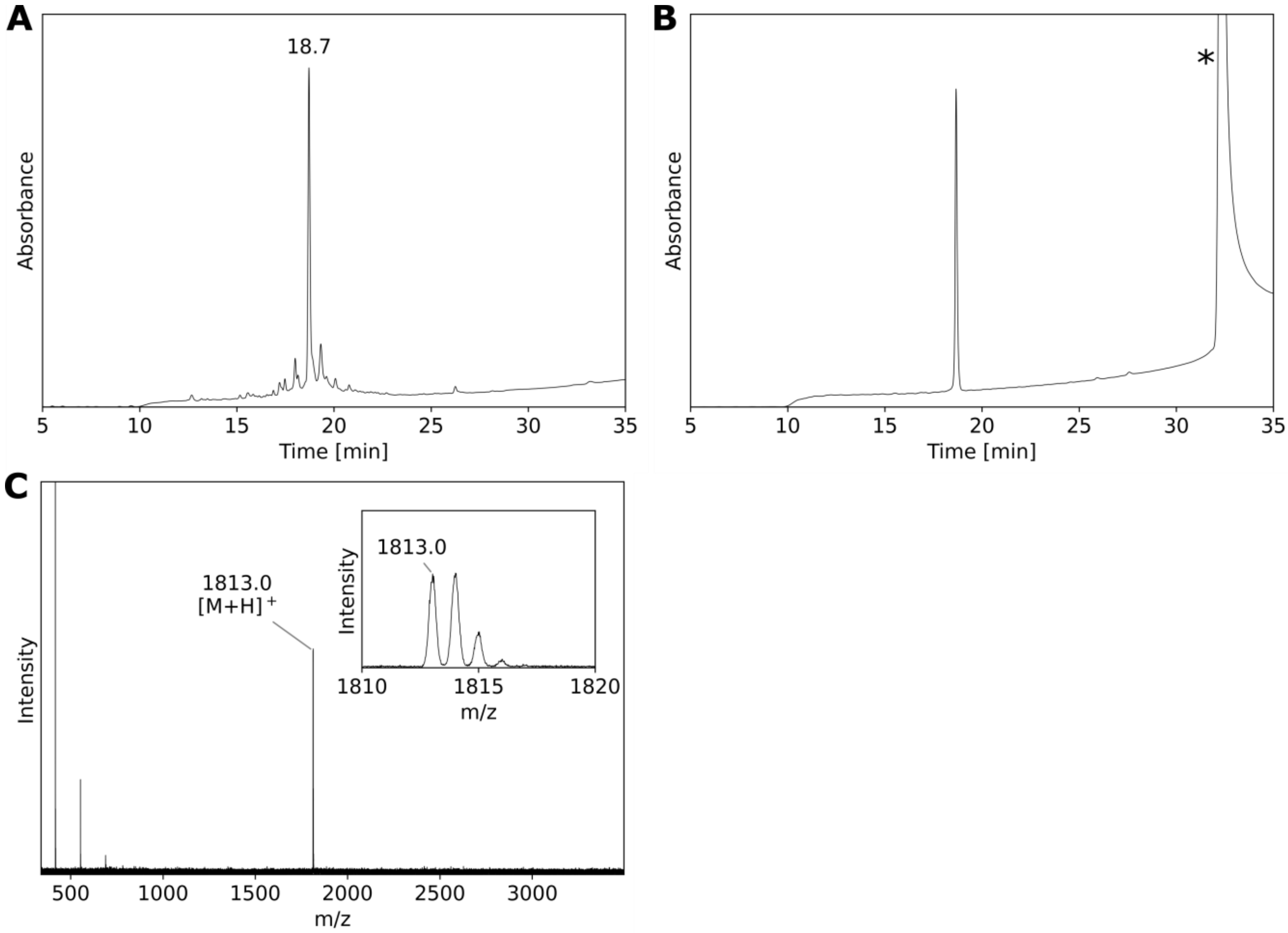

Figure S91: **P2A3** A) Analytical HPLC (220 nm) of crude peptide. HPLC Gradients are listed in Section 2.3.5 Table S3. B) Analytical HPLC (220 nm) of pure peptide. * Carryover peak. C) MALDI-TOF MS of pure peptide. Calculated mass: $[M+H]^+$ 1813.0.

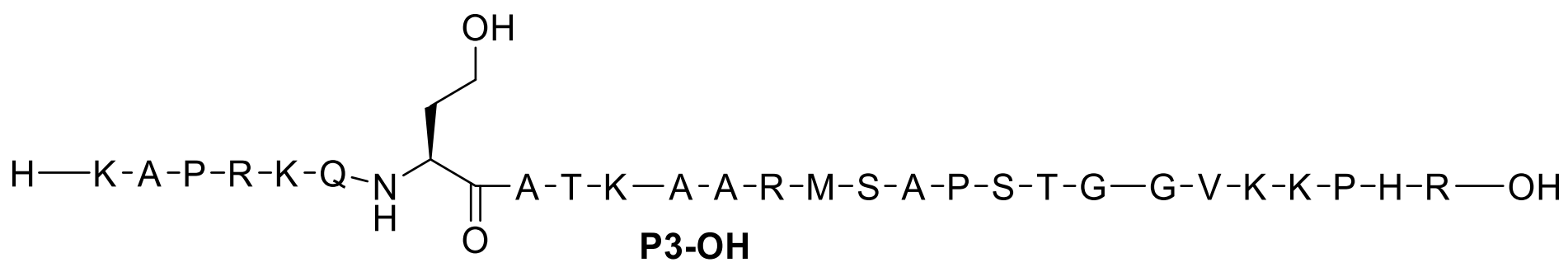

P3-OH

Chemical Formula: $C_{121}H_{214}N_{44}O_{34}S$

Exact Mass: 2859.61

Molecular Weight: 2861.38

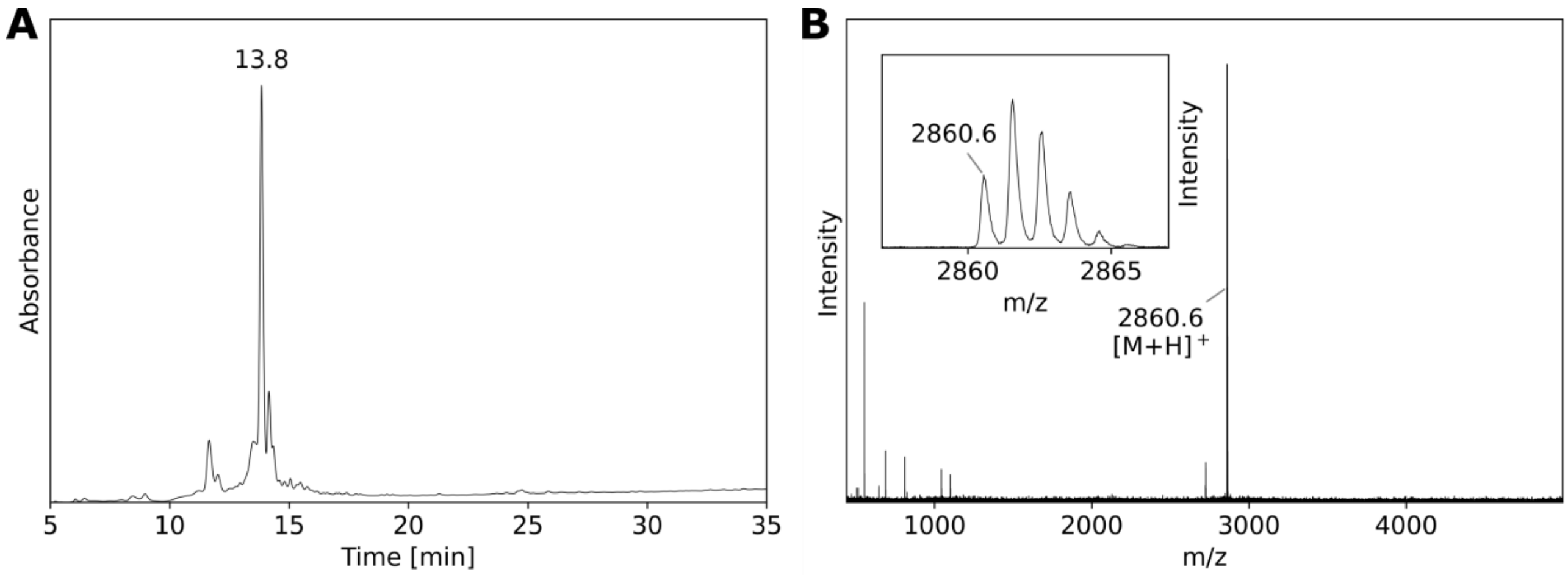

Figure S92: **P3-OH** A) Analytical HPLC (220 nm) of crude peptide. HPLC Gradients are listed in Section 2.3.5 Table S3. B) MALDI-TOF MS of crude peptide. Calculated mass: $[M+H]^+$ 2860.6.

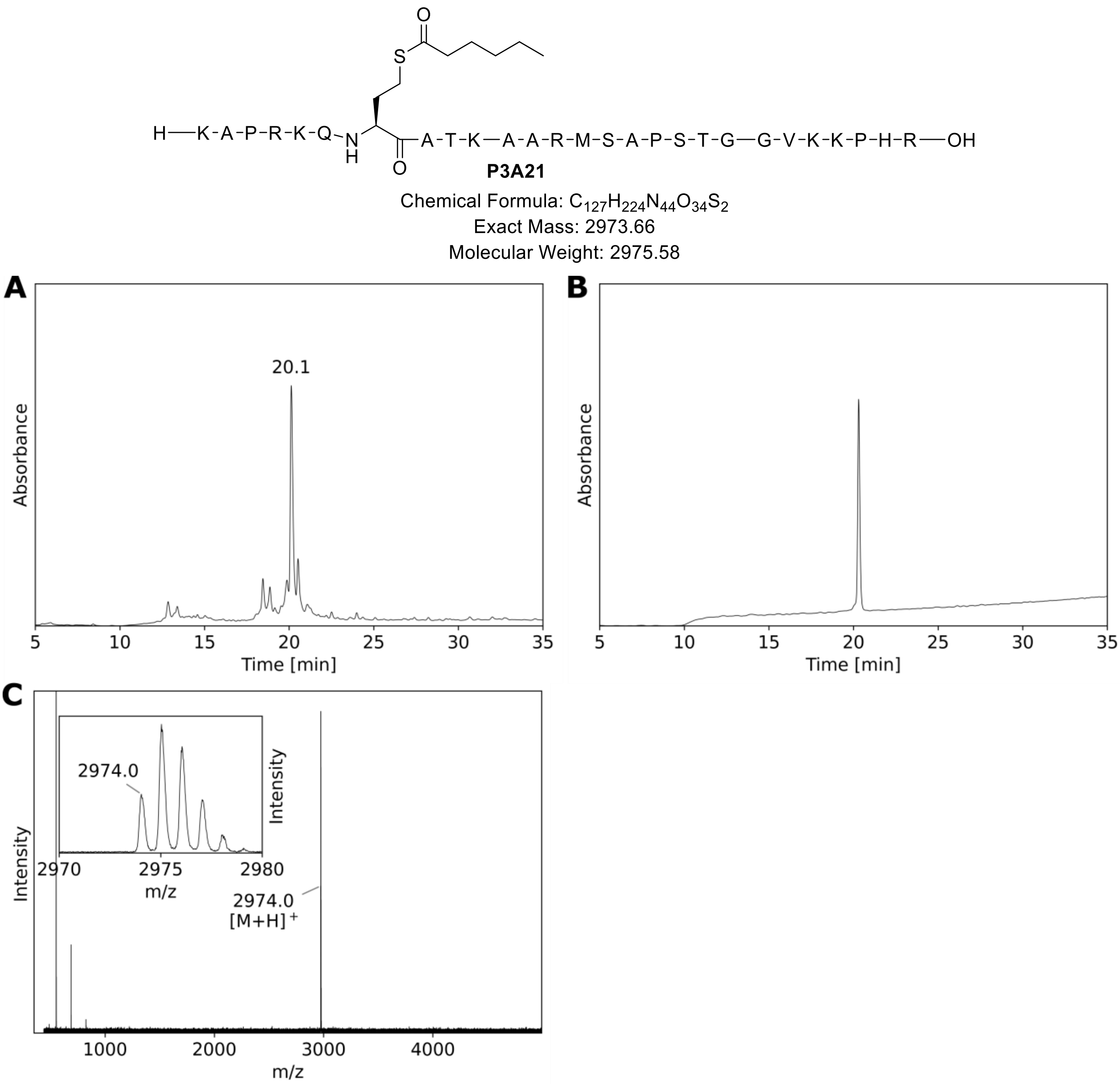

Figure S93: **P3A21** A) Analytical HPLC (220 nm) of crude peptide. HPLC Gradients are listed in Section 2.3.5 Table S3. B) Analytical HPLC (220 nm) of pure peptide. C) MALDI-TOF MS of pure peptide. Calculated mass: $[M+H]^+$ 2974.6.

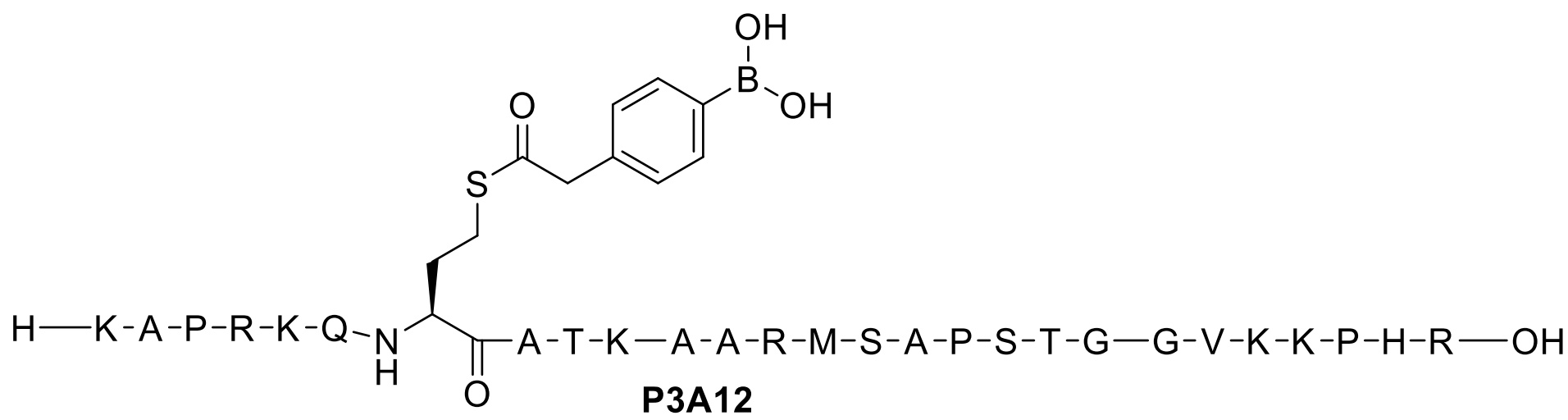

P3A12

Chemical Formula: $C_{129}H_{221}BN_{44}O_{36}S_2$
Exact Mass: 3037.63
Molecular Weight: 3039.39

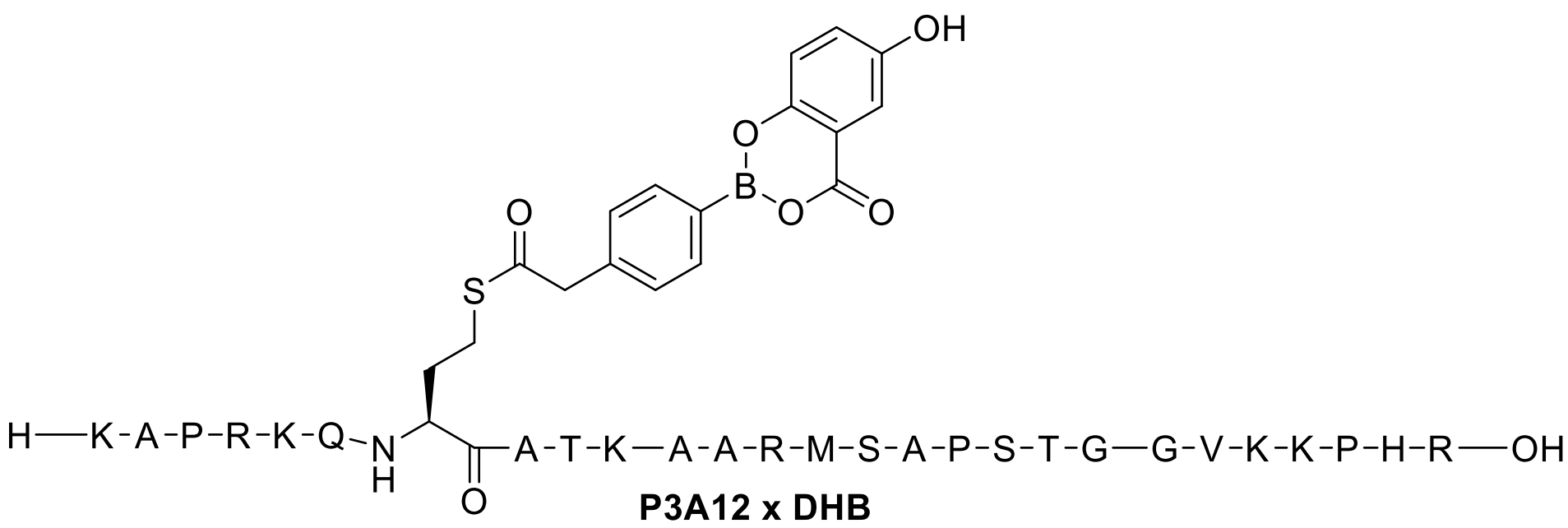

P3A12 x DHB

Chemical Formula: $C_{136}H_{223}BN_{44}O_{38}S_2$
Exact Mass: 3155.64
Molecular Weight: 3157.48

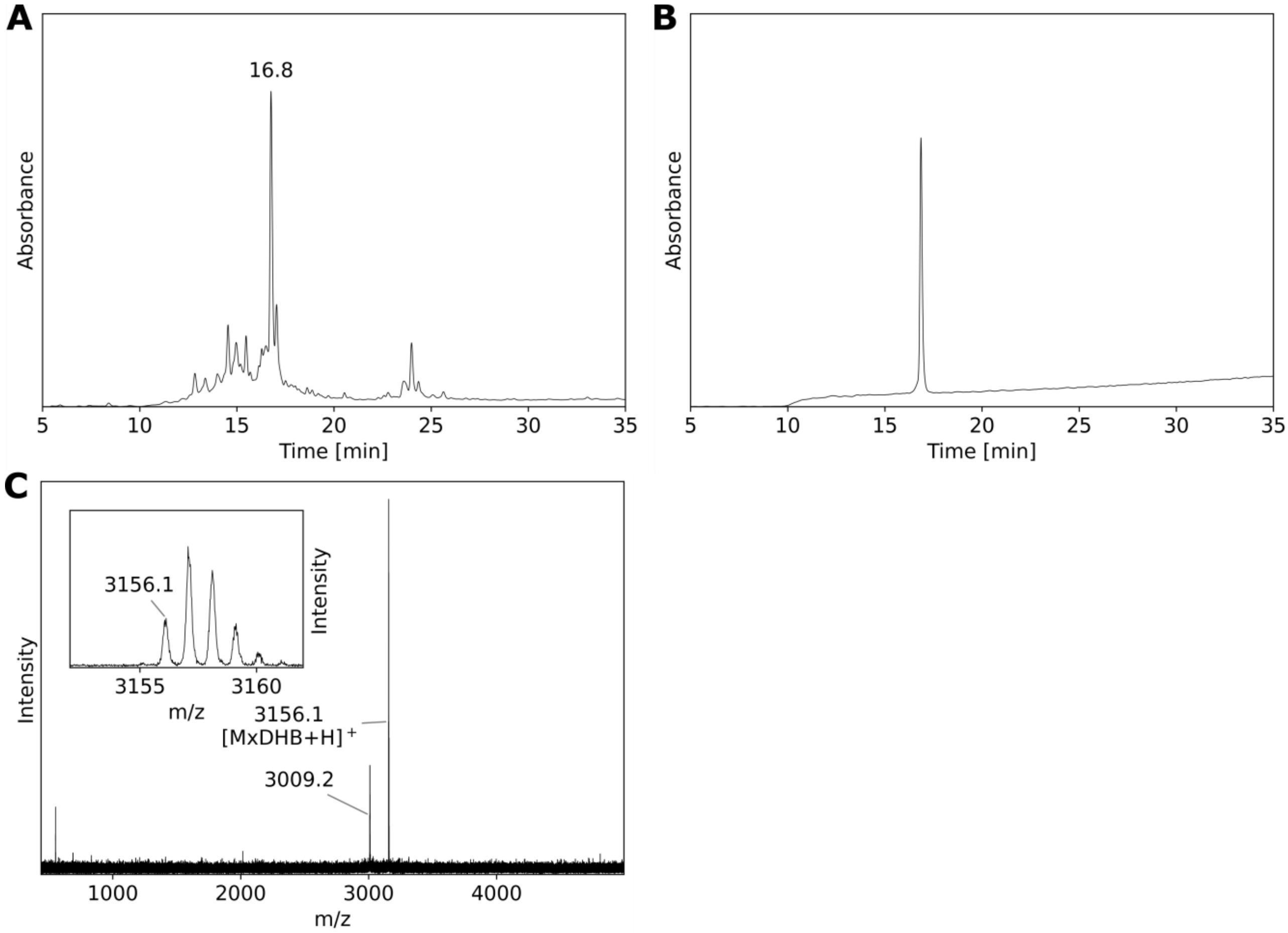

Figure S94: **P3A12** A) Analytical HPLC (220 nm) of crude peptide. HPLC Gradients are listed in Section 2.3.5 Table S3. B) Analytical HPLC (220 nm) of pure peptide. C) MALDI-TOF MS of pure peptide. Calculated mass: $[MxDHB+H]^+$ 3156.6.

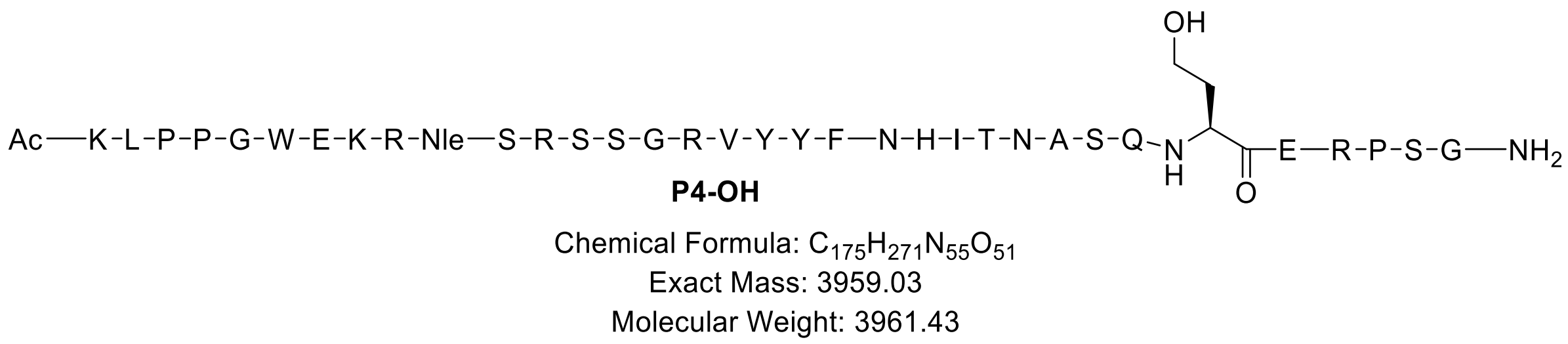

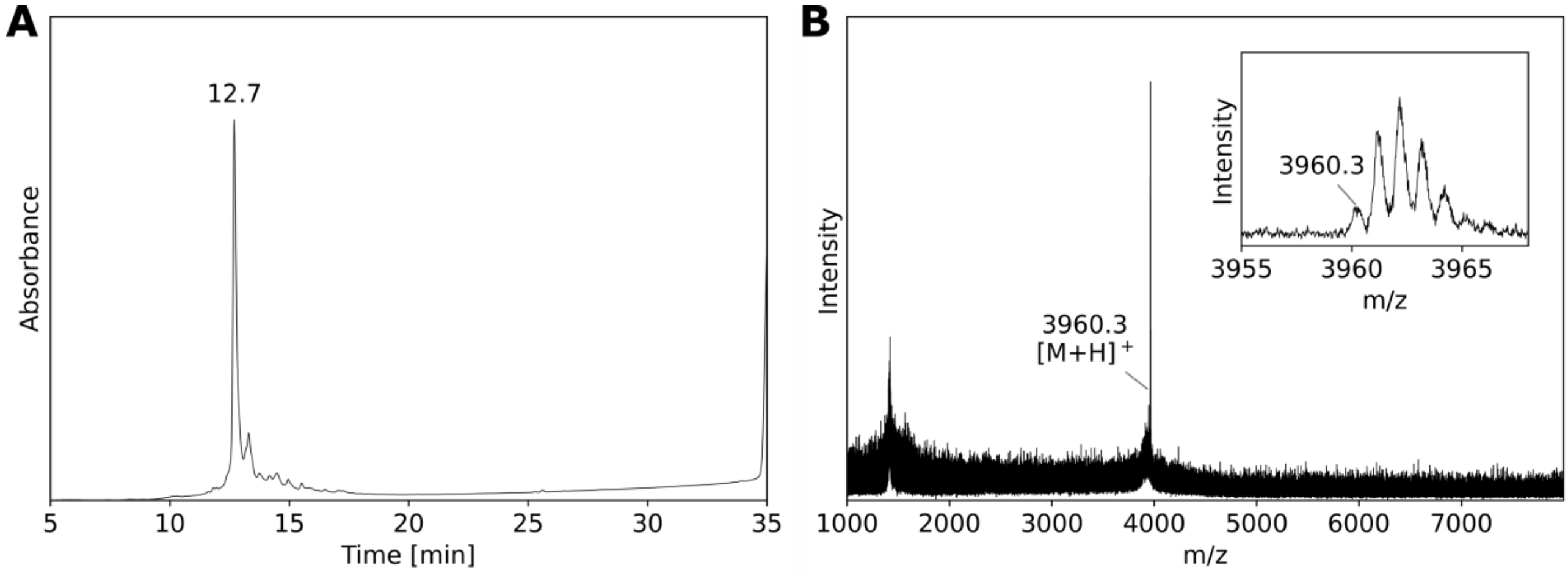

Figure S95: **P4-OH** A) Analytical HPLC (220 nm) of crude peptide. HPLC Gradients are listed in Section 2.3.5 Table S3. B) MALDI-TOF MS of crude peptide. Calculated mass: $[M+H]^+$ 3960.0.

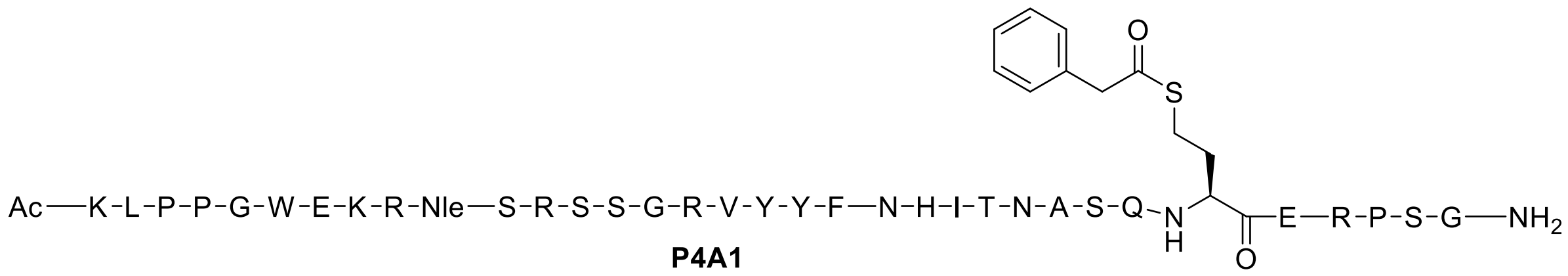

P4A1

Chemical Formula: $C_{183}H_{277}N_{55}O_{51}S$
Exact Mass: 4093.05
Molecular Weight: 4095.62

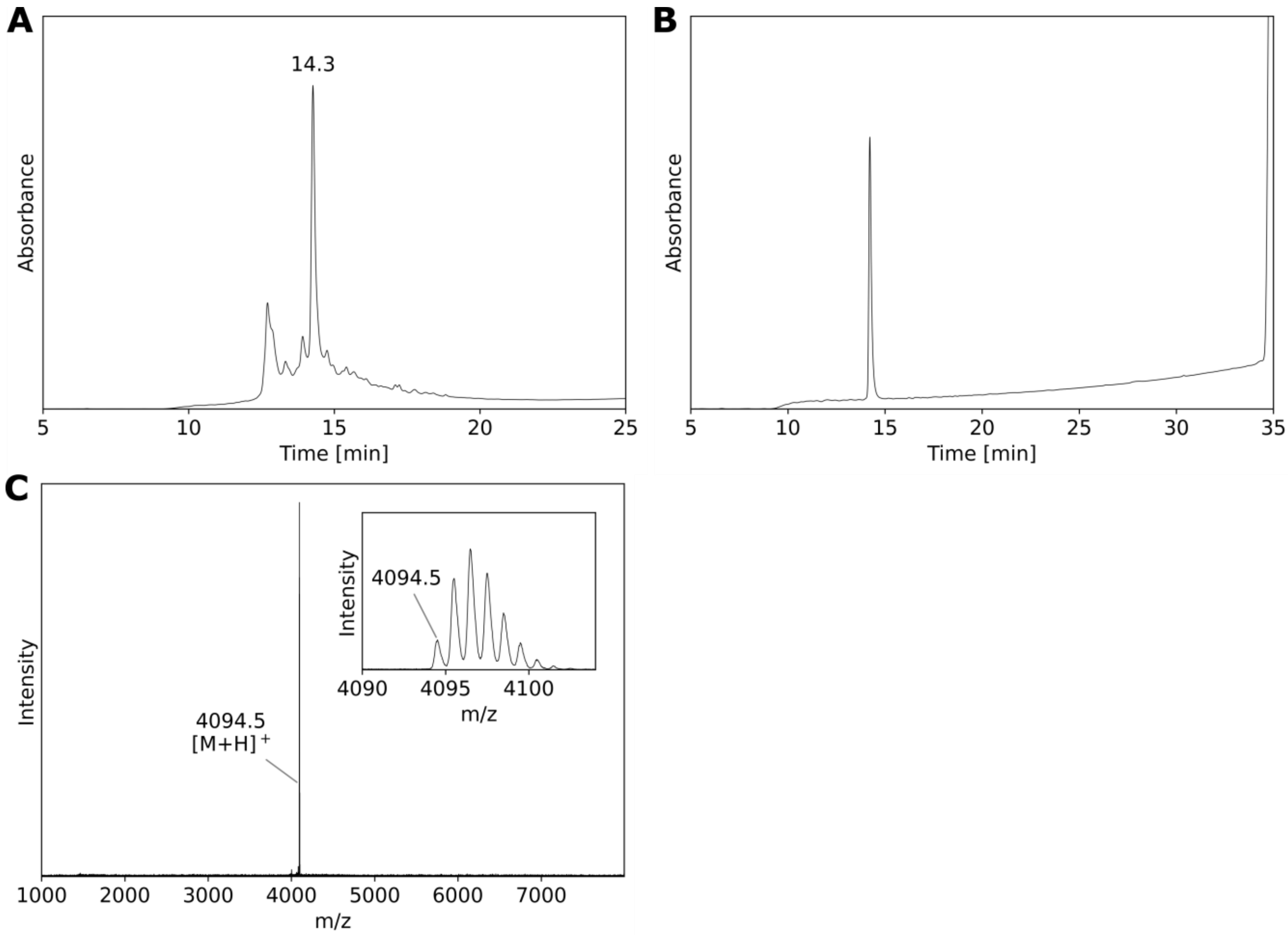

Figure S96: **P4A1** A) Analytical HPLC (220 nm) of crude peptide. HPLC Gradients are listed in Section 2.3.5 Table S3. B) Analytical HPLC (220 nm) of pure peptide. C) MALDI-TOF MS of pure peptide. Calculated mass: $[M+H]^+$ 4094.1.

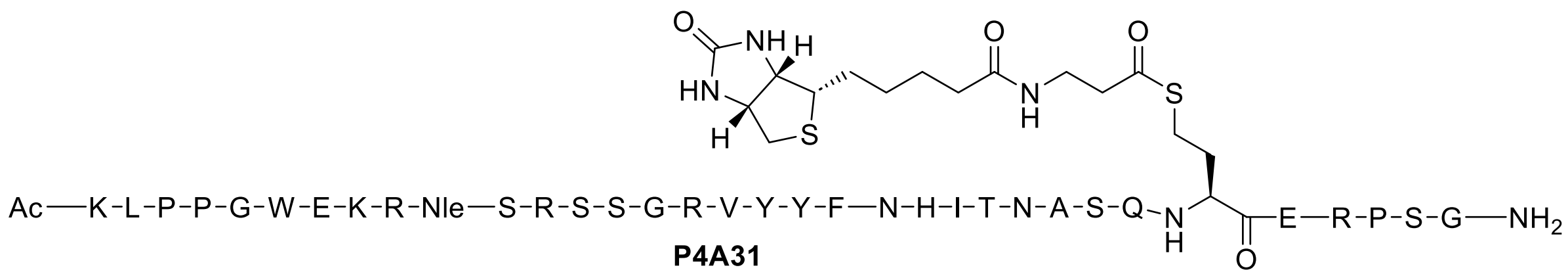

P4A31

Chemical Formula: $C_{188}H_{290}N_{58}O_{53}S_2$
Exact Mass: 4272.12
Molecular Weight: 4274.86

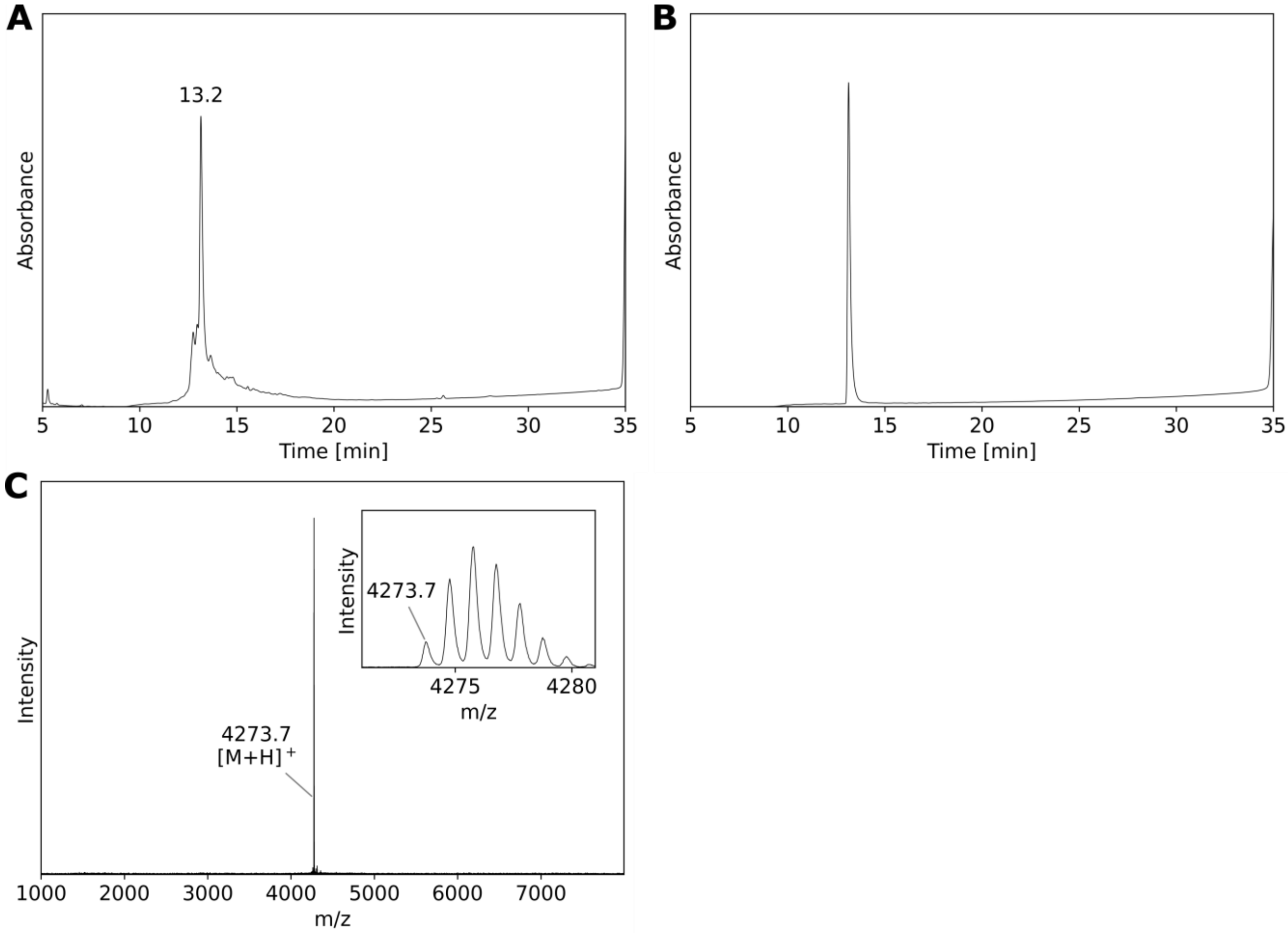

Figure S97: **P4A31** A) Analytical HPLC (220 nm) of crude peptide. HPLC Gradients are listed in Section 2.3.5 Table S3. B) Analytical HPLC (220 nm) of pure peptide. C) MALDI-TOF MS of pure peptide. Calculated mass: $[M+H]^+$ 4273.1.

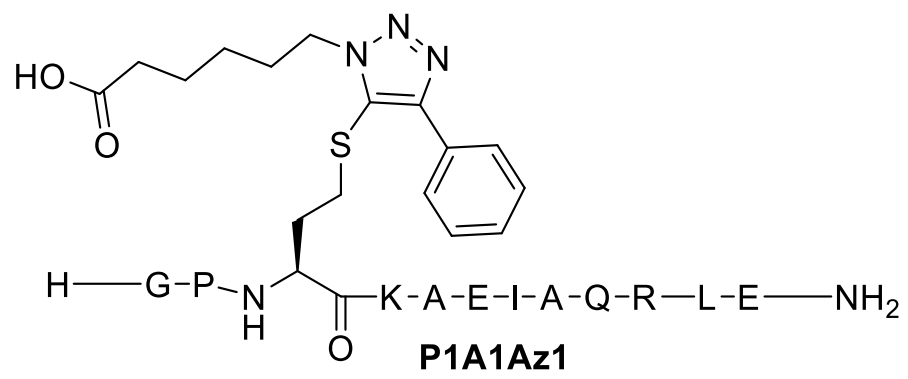

P1A1Az1
Chemical Formula: $C_{70}H_{113}N_{21}O_{19}S$
Exact Mass: 1583.82
Molecular Weight: 1584.86

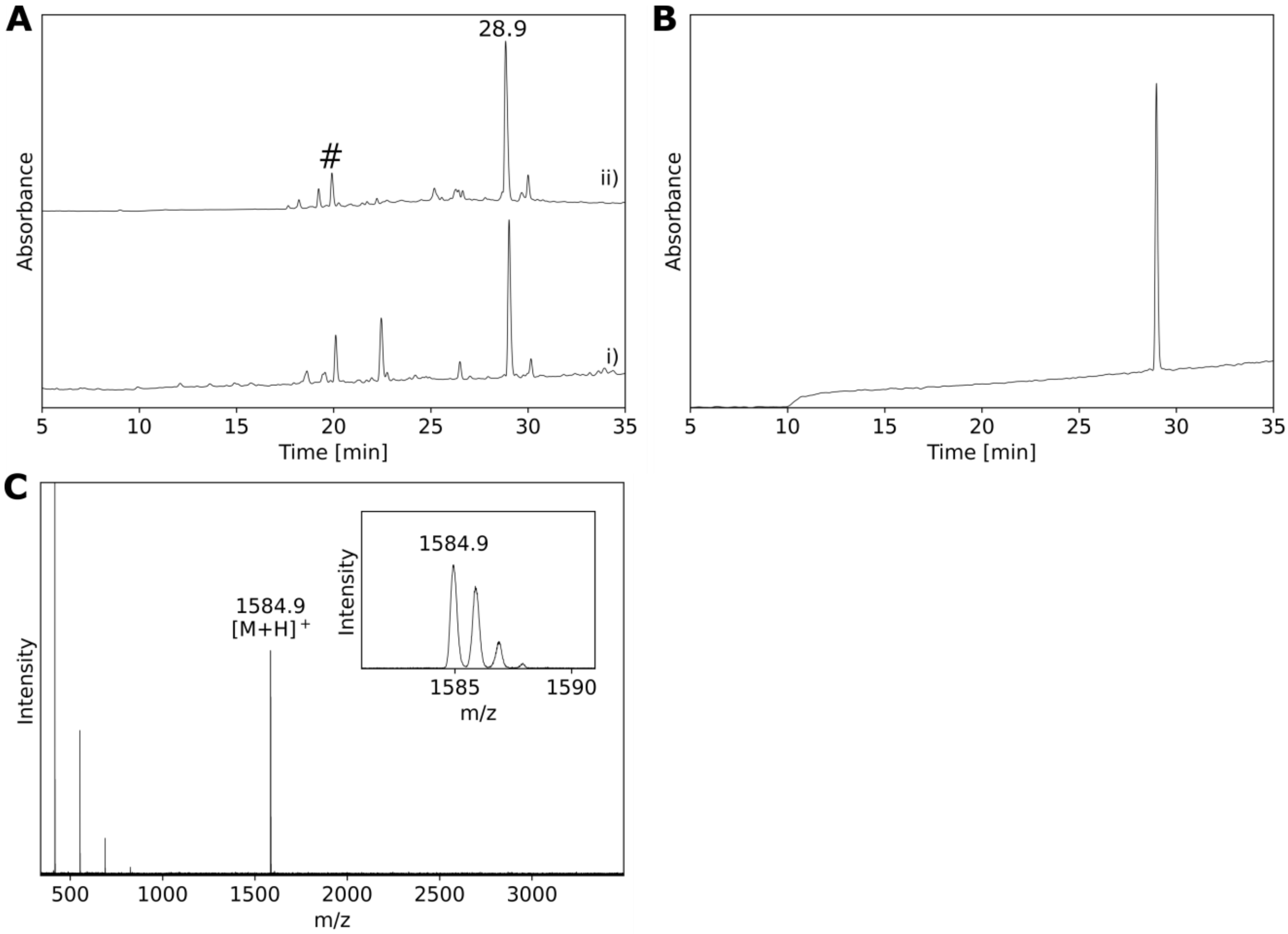

Figure S98: **P1A1Az1** A) Analytical HPLC (220 nm) of crude peptide prepared with (i) conditions for Ru-catalyzed click reaction from Table 3 Entry 1 and (ii) standard conditions for Ir-catalyzed click reaction. HPLC Gradients are listed in Section 2.3.5 Table S3. # Peptide without homoserine. B) Analytical HPLC (220 nm) of pure peptide. C) MALDI-TOF MS of pure peptide. Calculated mass: $[M+H]^+$ 1584.8.

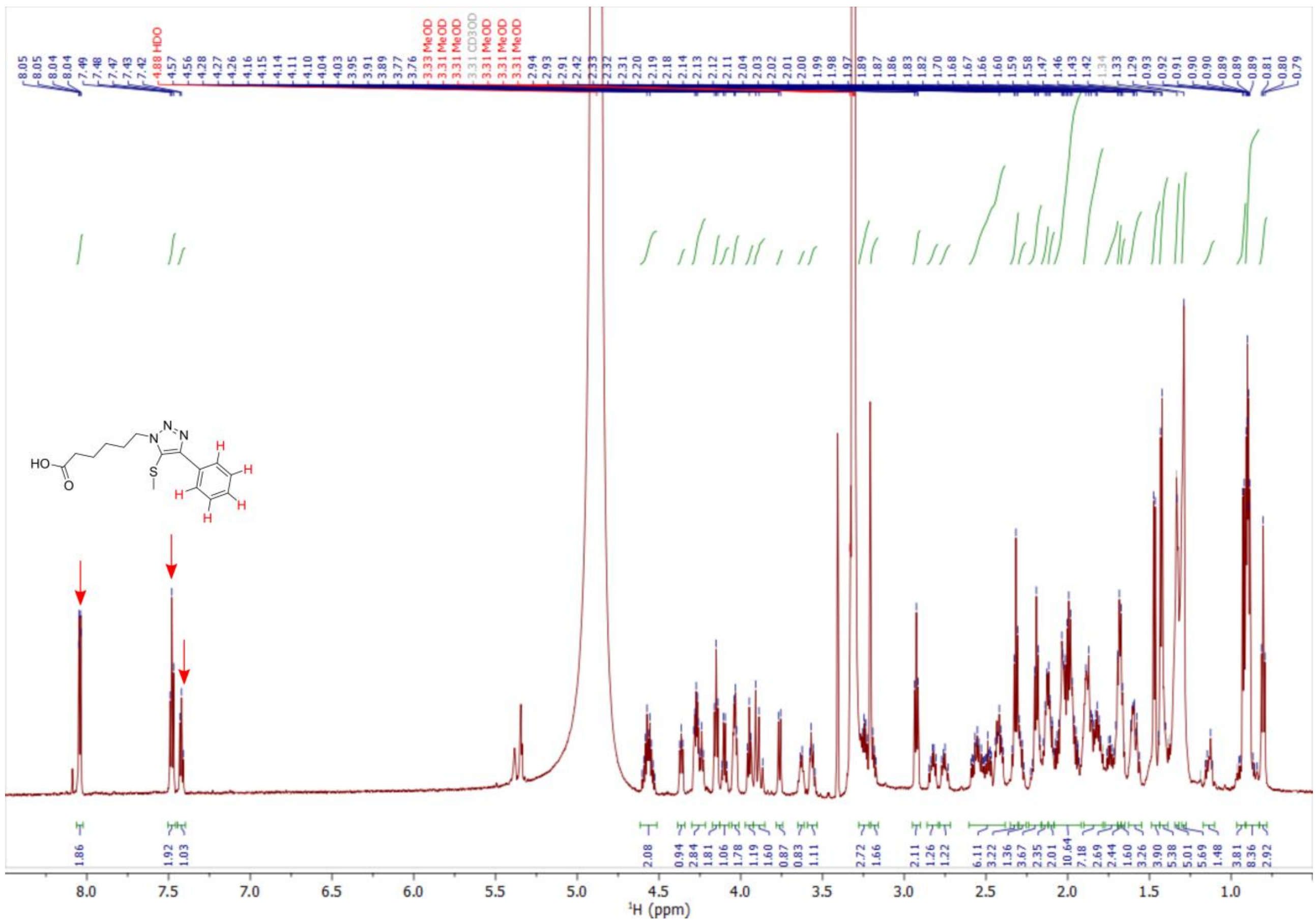

Figure S99: ^{1}H-NMR (700 MHz, MeOH-d_4, 295 K) of **P1A1Az1**. Hydrogen-deuterium exchange was performed prior to the measurement.

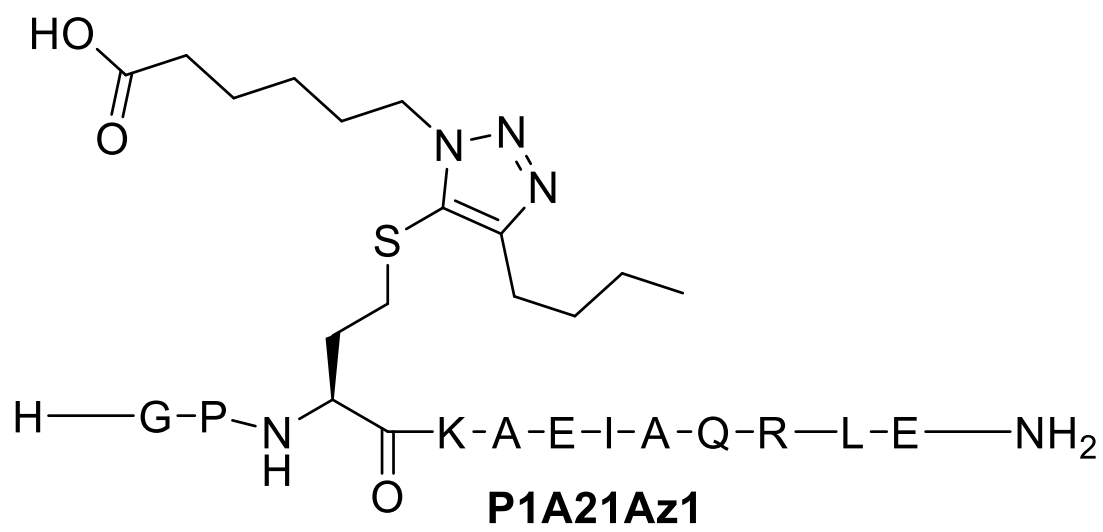

P1A21Az1
Chemical Formula: $C_{68}H_{117}N_{21}O_{19}S$
Exact Mass: 1563.86
Molecular Weight: 1564.87

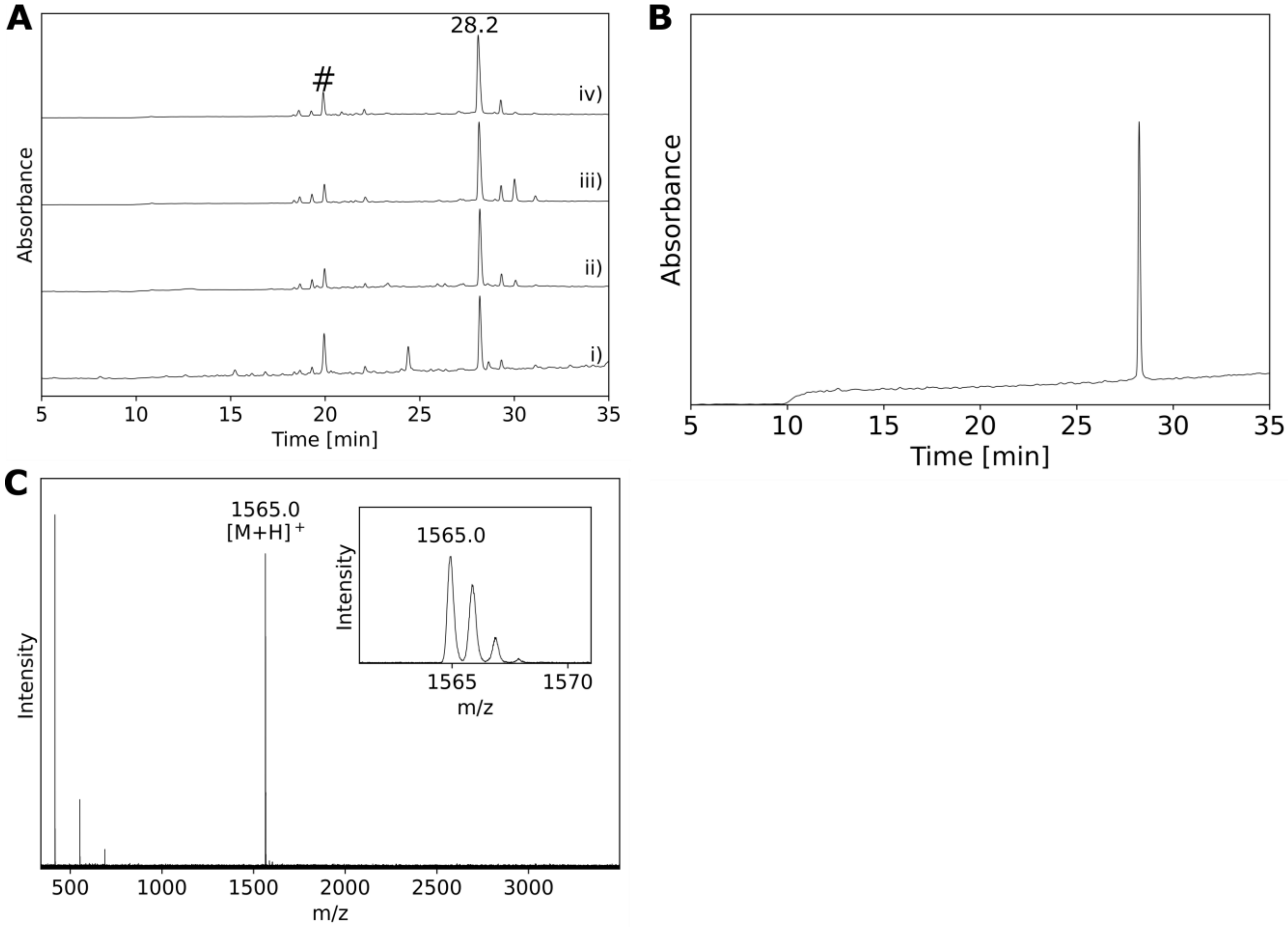

Figure S100: **P1A21Az1** A) Analytical HPLC (220 nm) of crude peptides prepared by a reaction sequence of *S*-alkynylation and azide-alkyne cycloaddition under the conditions displayed in Table 3 (i) Entry 1, (ii) Entry 2, (iii) Entry 3 and (iv) Entry 4. HPLC Gradients are listed in Section 2.3.5 Table S3. # Peptide without homoserine. B) Analytical HPLC (220 nm) of pure peptide. C) MALDI-TOF MS of pure peptide. Calculated mass: $[M+H]^+$ 1564.9.

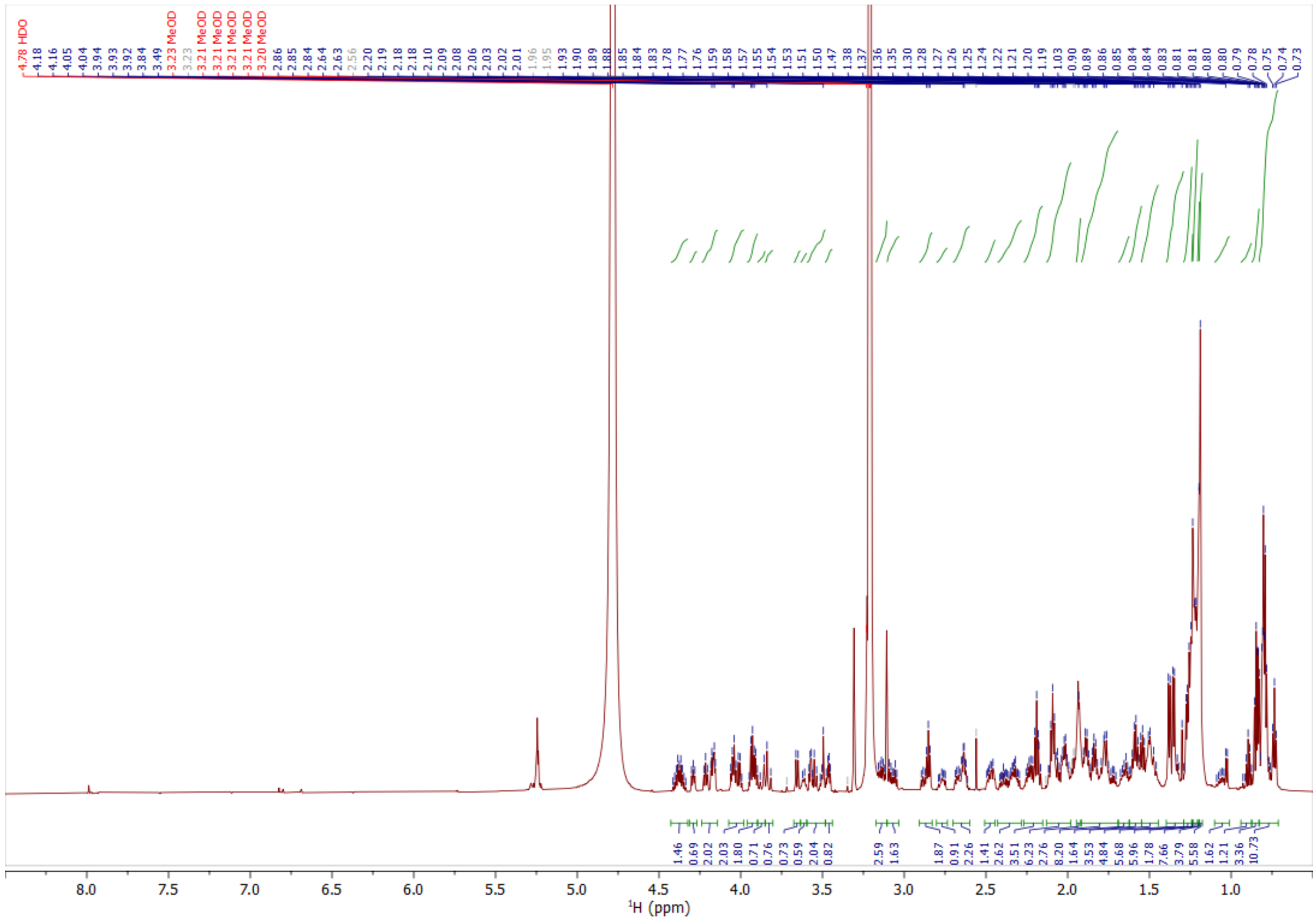

Figure S101: ^{1}H-NMR (700 MHz, MeOH-d_4, 295 K) of **P1A21Az1**. Hydrogen-deuterium exchange was performed prior to the measurement.

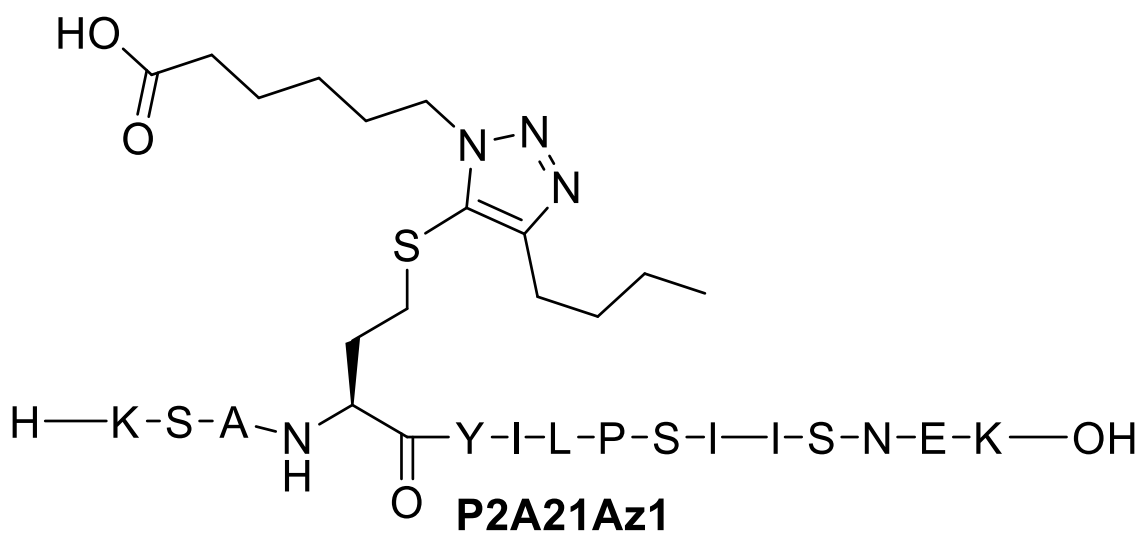

P2A21Az1

Chemical Formula: $C_{87}H_{145}N_{21}O_{25}S$

Exact Mass: 1916.04

Molecular Weight: 1917.30

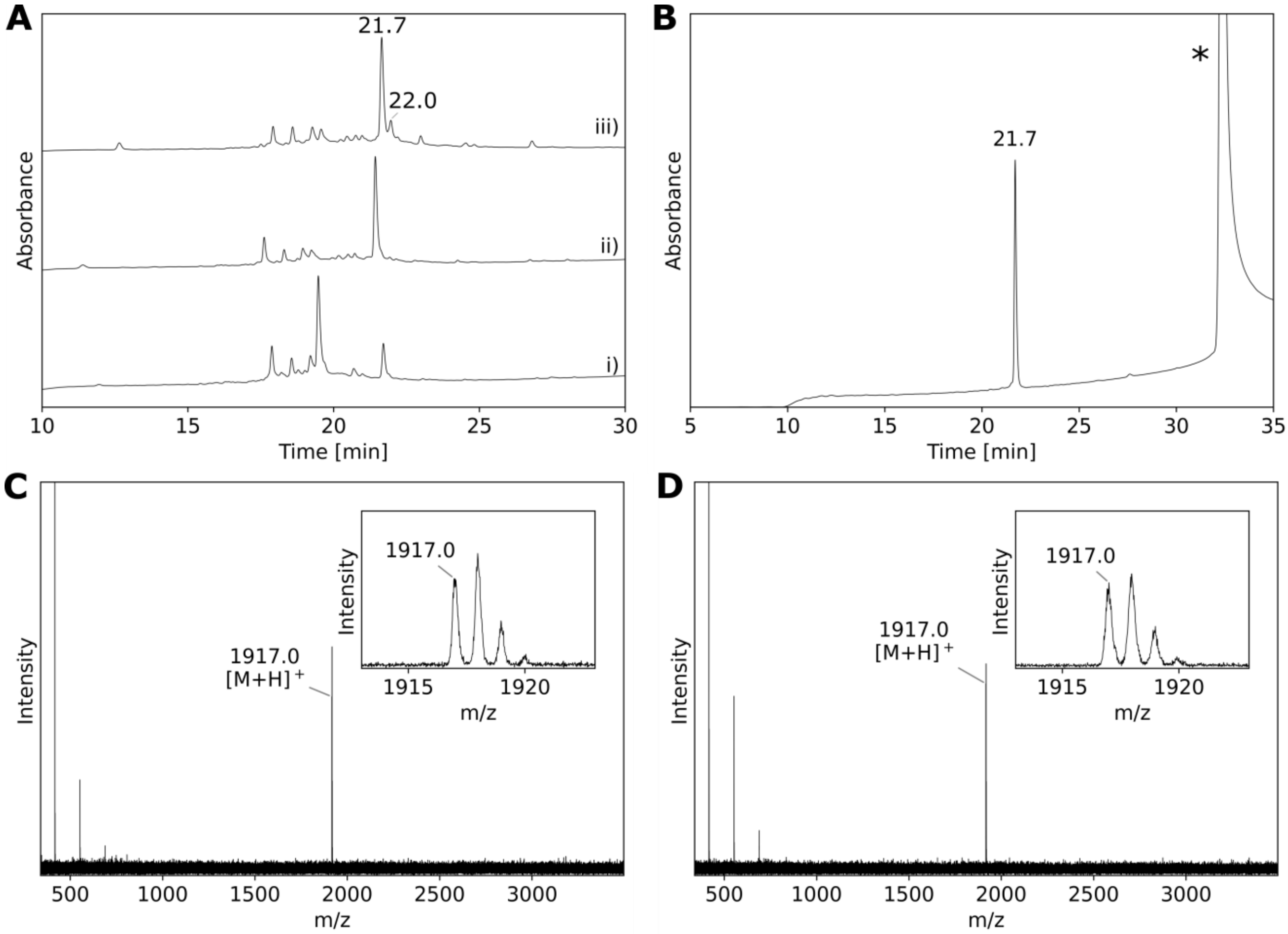

Figure S102: **P2A21Az1** A) Analytical HPLC (220 nm) of crude peptide prepared with (i) standard conditions for Ir-catalyzed click reaction, (ii) adapted conditions for Ir-catalyzed click reaction (iii) conditions for Ru-catalyzed click reaction from Table 3 Entry 1. HPLC Gradients are listed in Section 2.3.5 Table S3. B) Analytical HPLC (220 nm) of purified peptide with a retention time of 21.7 min. * Carryover peak. C) MALDI-TOF MS of purified peptide with a retention time of 21.7 min. D) MALDI-TOF MS of purified peptide with a retention time of 22.0 min. Calculated mass: $[M+H]^+$ 1917.1.

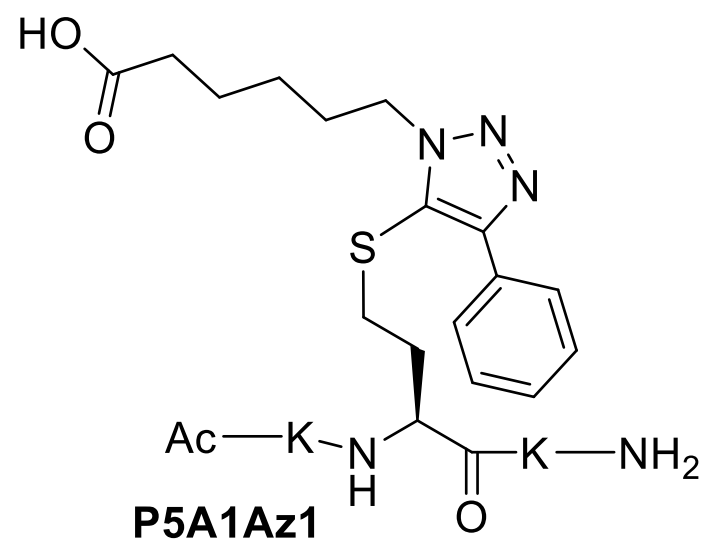

P5A1Az1

Chemical Formula: $C_{32}H_{51}N_9O_6S$
Exact Mass: 689.37
Molecular Weight: 689.88

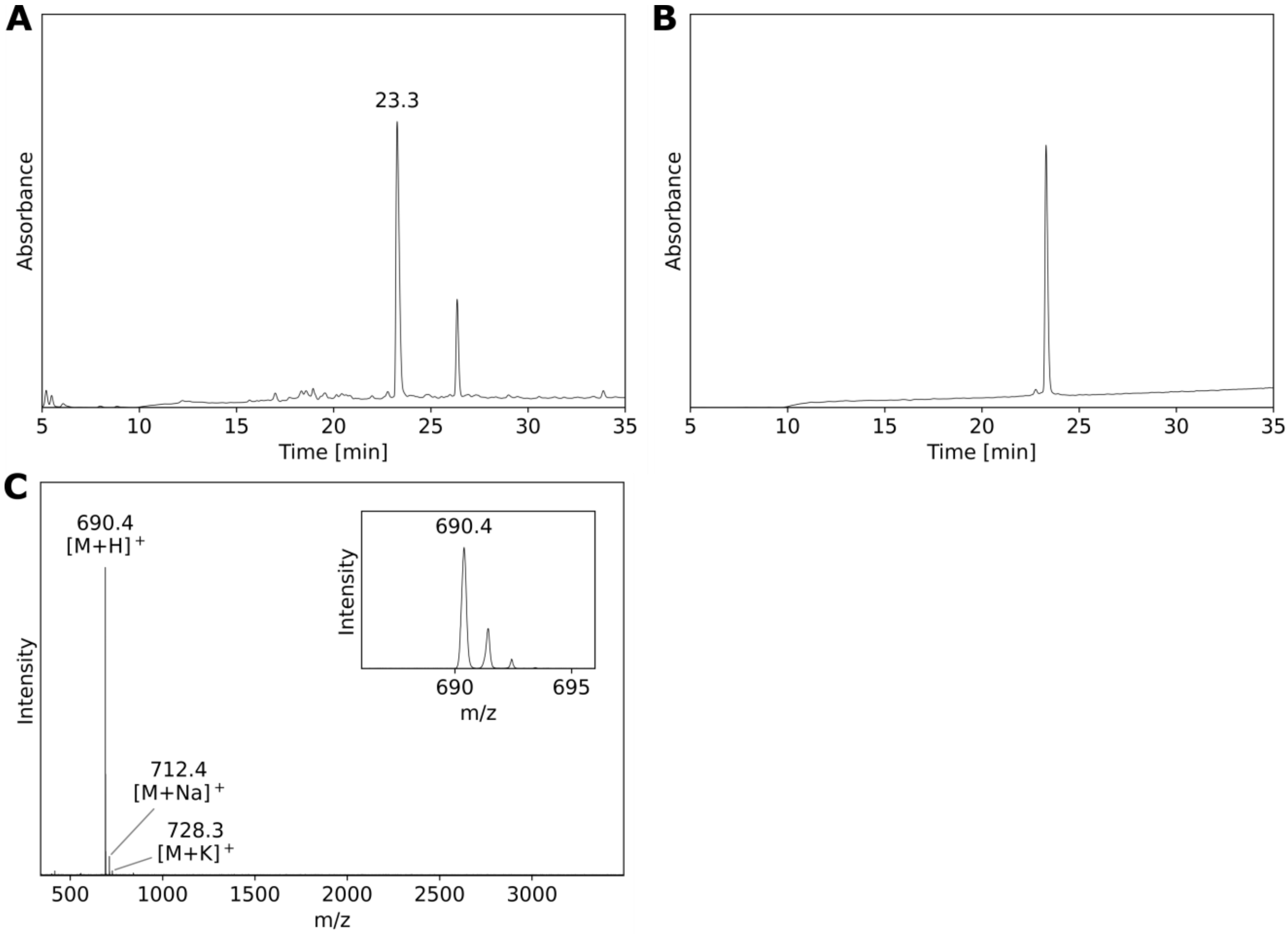

Figure S103: **P5A1Az1** A) Analytical HPLC (220 nm) of crude peptide. HPLC Gradients are listed in section 2.3.5 Table S3. B) Analytical HPLC (220 nm) of pure peptide. C) MALDI-TOF MS of pure peptide. Calculated mass: $[M+H]^+$ 690.4, $[M+Na]^+$ 712.4, $[M+K]^+$ 728.3.

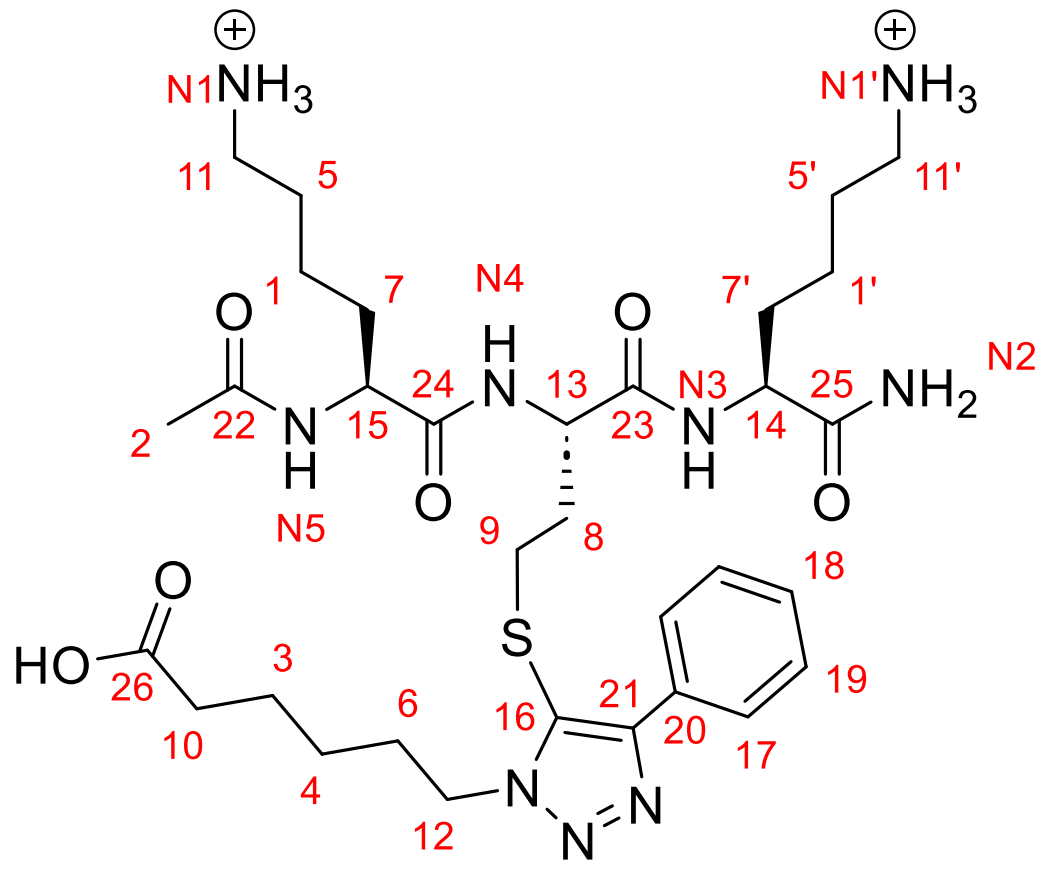

^{1}H NMR (400 MHz, DMSO-*d*$_6$, 295 K):

δ [ppm] = 8.07-8.05 (m, 2H, H-17), 8.01-7.97 (m, 2H, H-N5/H-N4), 7.74-7.72 (m, 7H, H-N1/H-N3), 7.51-7.47 (m, 1H, H-19), 7.42-7.38 (m, 1H, H-18), 7.30 (s, 1H, H-N2), 7.00 (s, 1H, H-N2), 4.46 (t, *J* = 7.1 Hz, 2H, H-12), 4.28-4.22 (m, 1H, H-13), 4.12-4.06 (m, 2H, H-15/H-14), 2.78-2.71 (m, 6H, H-11/9), 2.21 (t, *J* = 7.3 Hz 2H, H-10), 1.93-1.86 (m, 2H, H-6), 1.82 (s, 3H, H-2), 1.61-1.38 (m, 10H, H-7/5/3), 1.35-1.17 (m, 6H, H-4/1).

^{13}C NMR (101 MHz, DMSO-*d*$_6$, 295 K):

δ [ppm] = 174.3 (C-26), 173.2 (C-25), 171.9 (C-24), 170.3 (C-23), 169.6 (C-22), 147.9 (C-21), 130.5 (C-20), 128.7 (C-19), 128.3 (C-18), 126.5 (C-17), 124.6 (C-16), 52.7 (C-15), 52.1 (C-14), 51.5 (C-13), 47.7 (C-12), 38.7 (C-11), 38.6 (C-11), 33.4 (C-10), 32.1 (C-9), 31.8 (C-8), 31.2 (C-7), 31.0 (C-7), 29.3 (C-6), 26.6 (C-5), 25.5 (C-4), 23.9 (C-3), 22.3 (C-2), 22.1 (C-1).

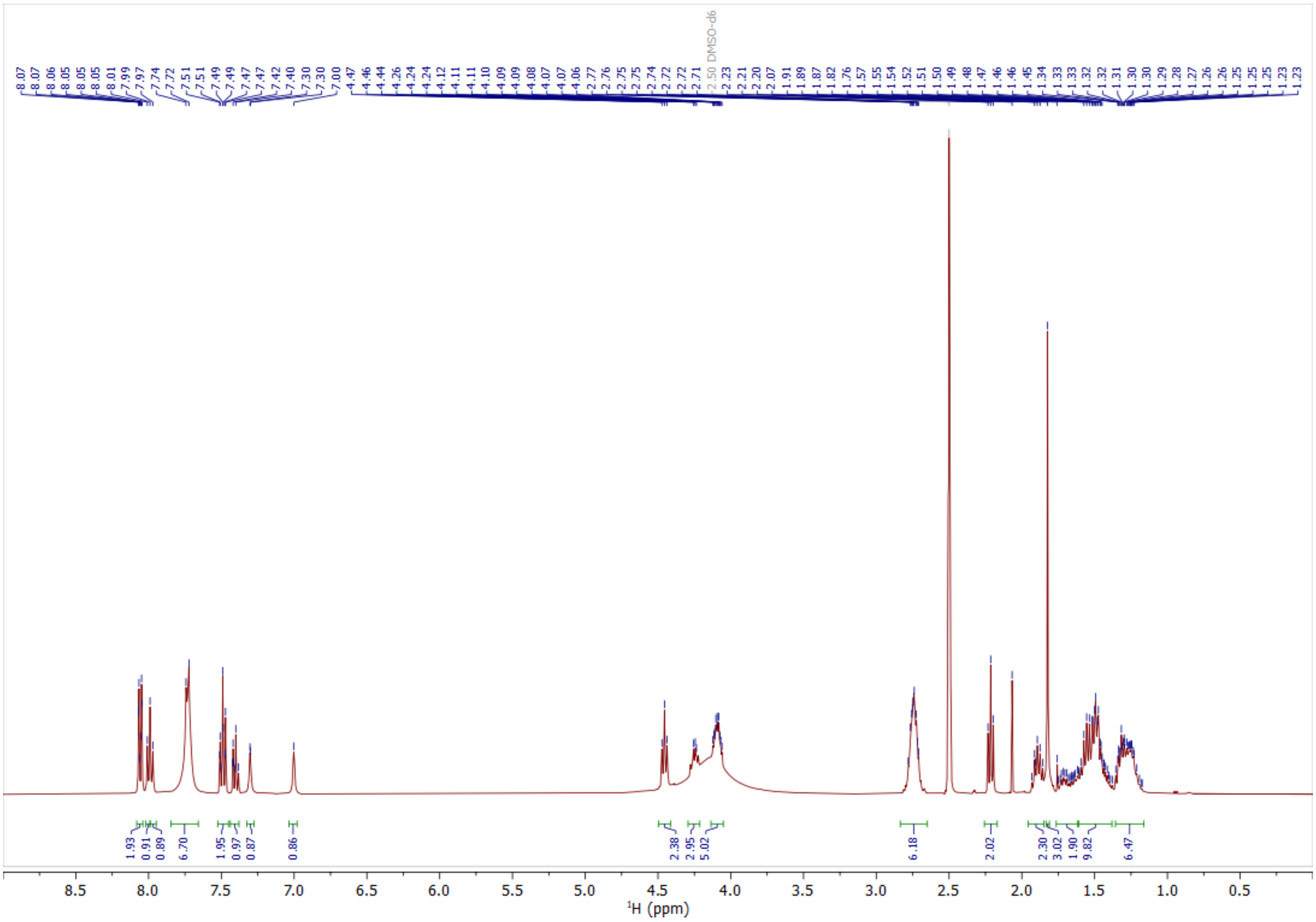

Figure S104: ^{1}H NMR spectrum (400 MHz, DMSO-*d*$_6$, 295 K) of **P5A1Az1**.

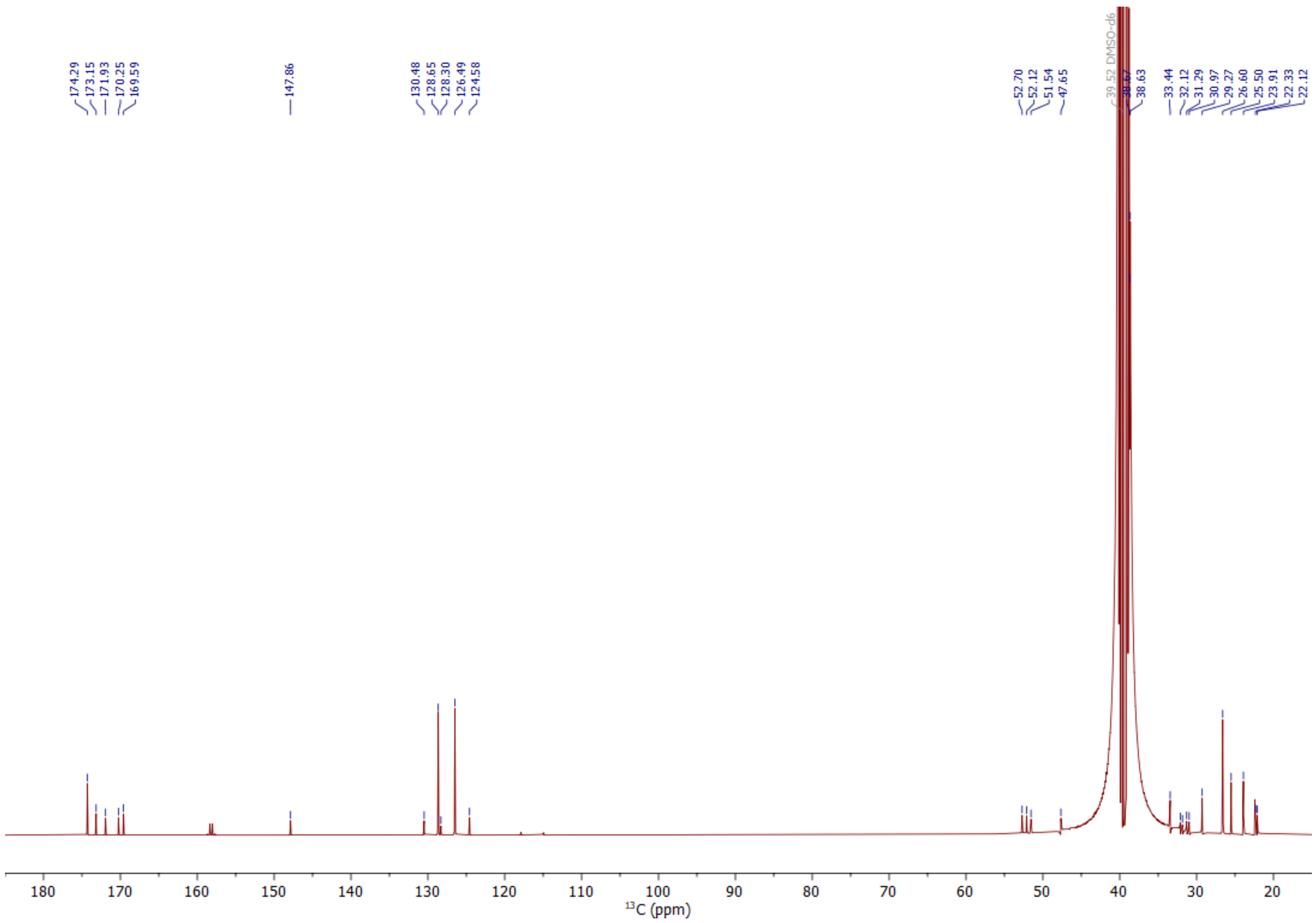

Figure S105: ^{13}C NMR spectrum (101 MHz, DMSO-d_6, 295 K) of **P5A1Az1**.

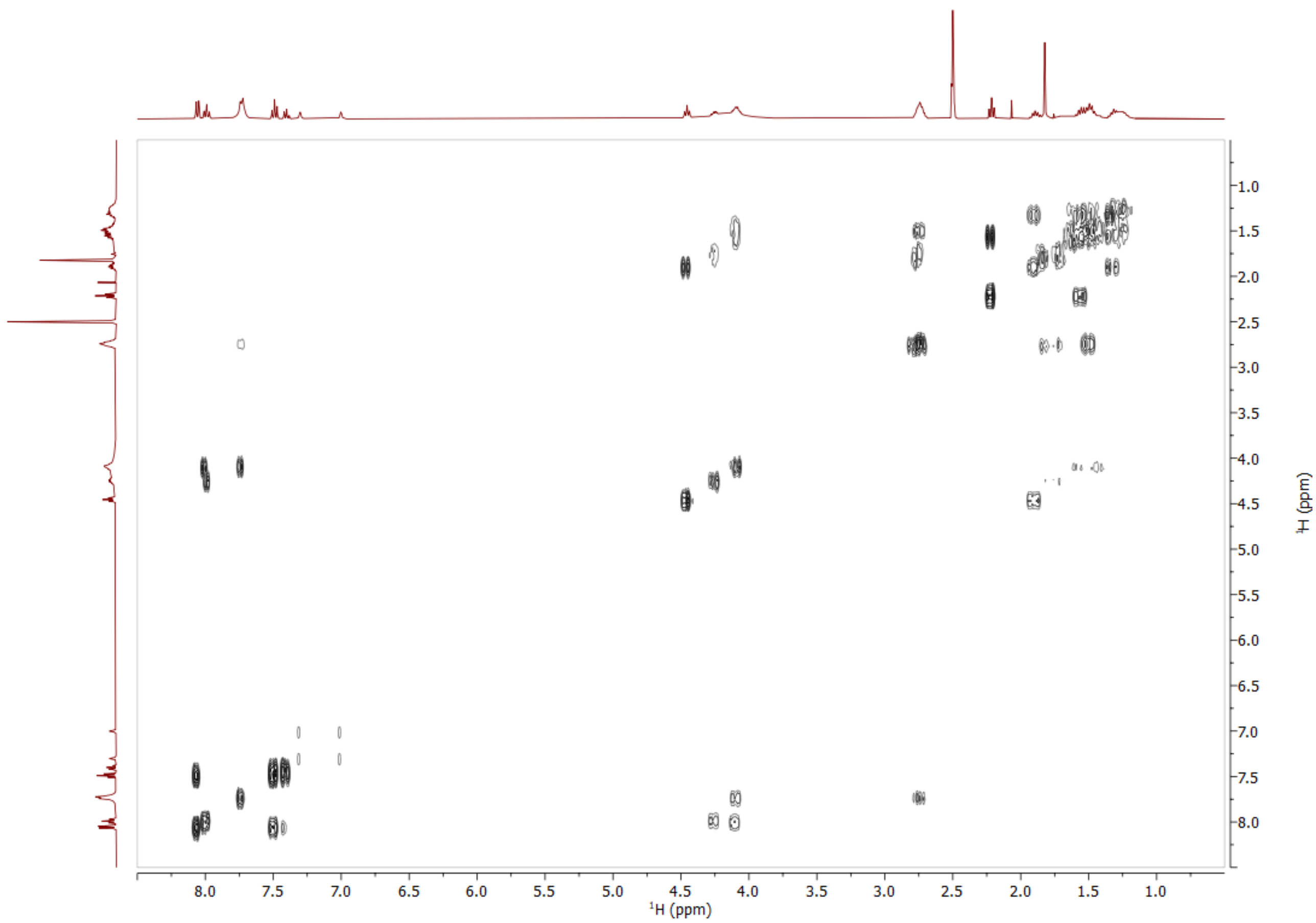

Figure S106: ^{1}H-^{1}H COSY spectrum (400 MHz, 400 MHz, DMSO-d_6, 295 K) of **P5A1Az1.**

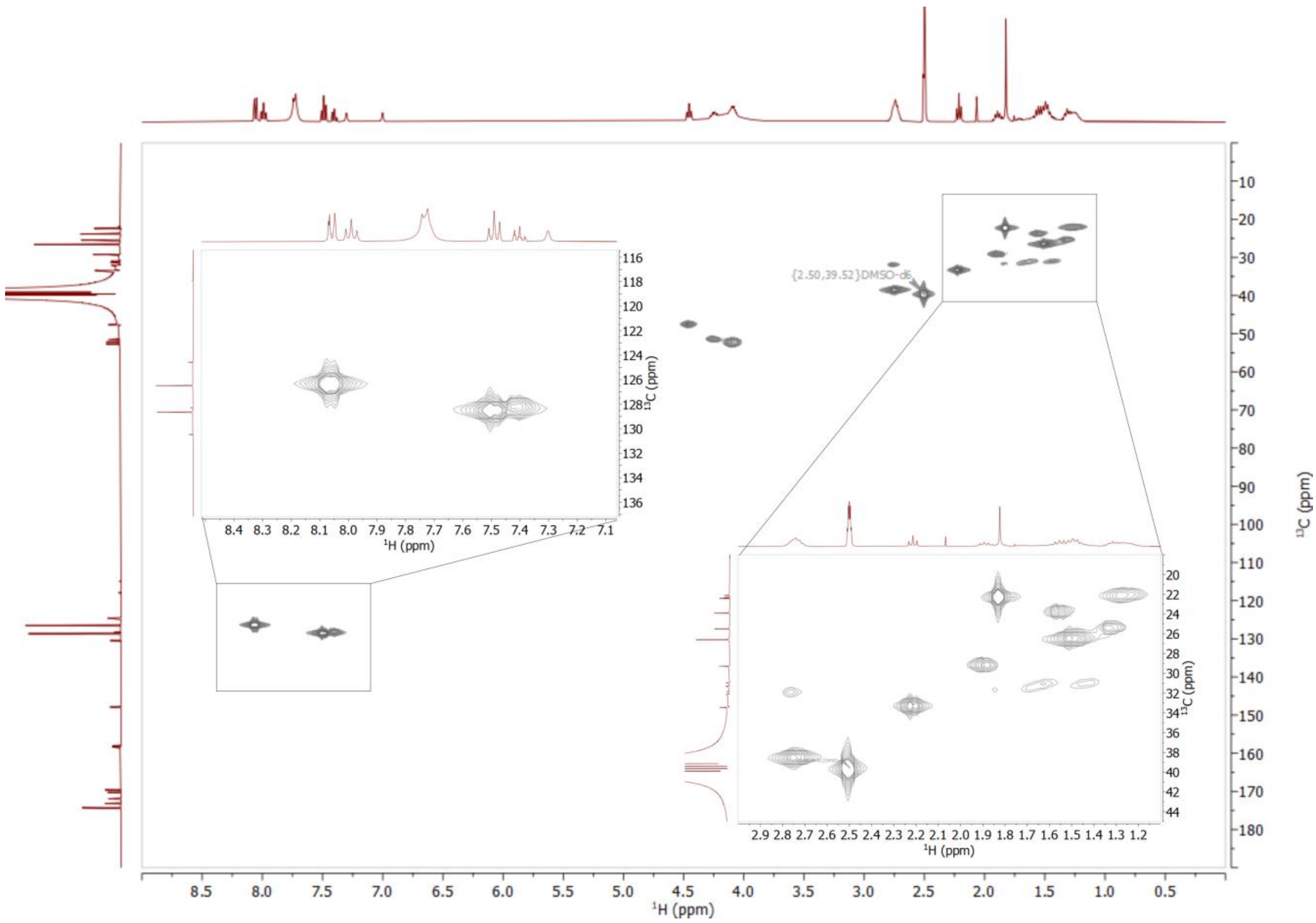

Figure S107: ^{1}H-^{13}C HSQC spectrum (400 MHz, 101 MHz, DMSO-d_6, 295 K) of **P5A1Az1**.

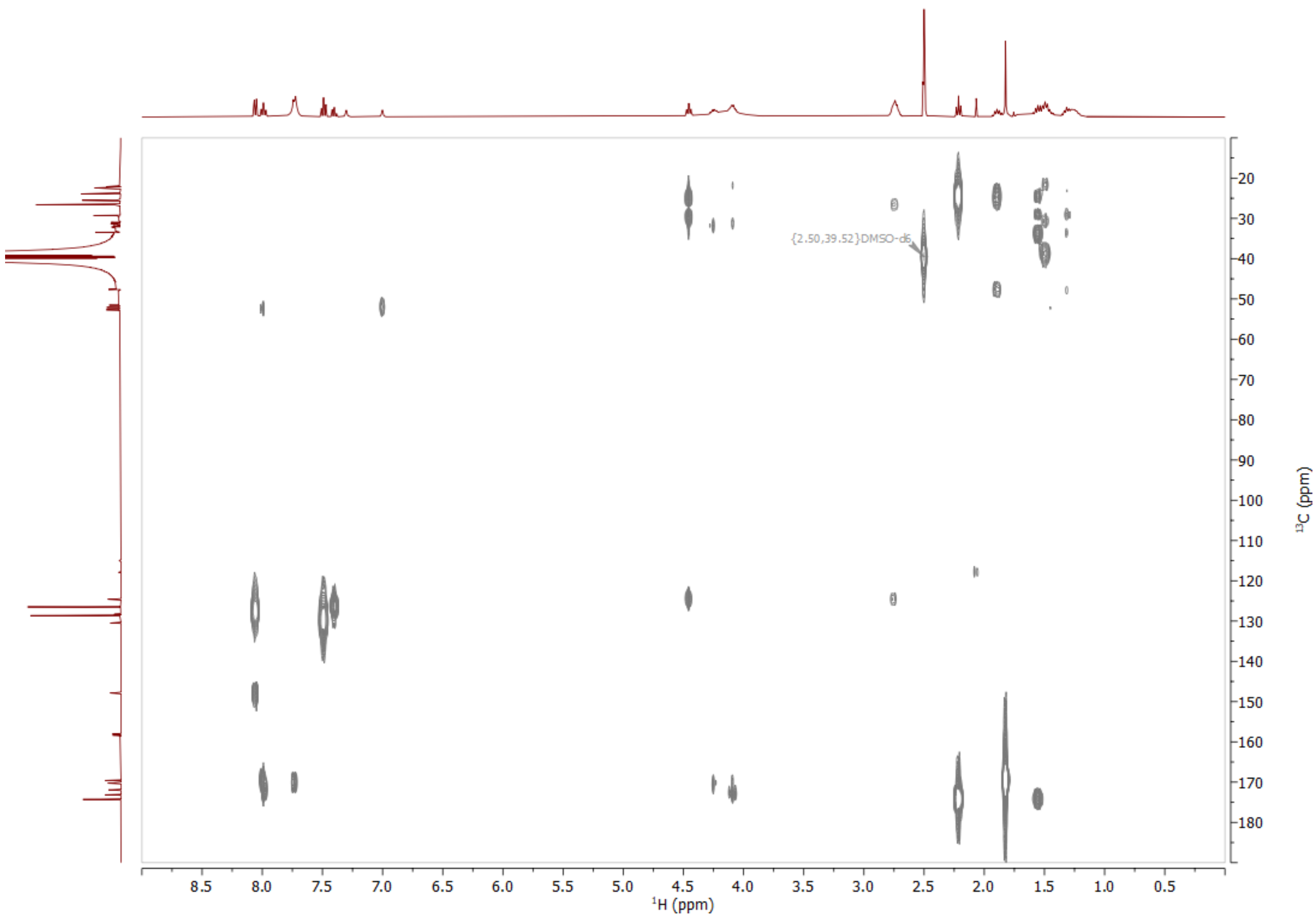

Figure S108: ^{1}H-^{13}C HMBC spectrum (400 MHz, 101 MHz, DMSO-d_6, 295 K) of **P5A1Az1**.

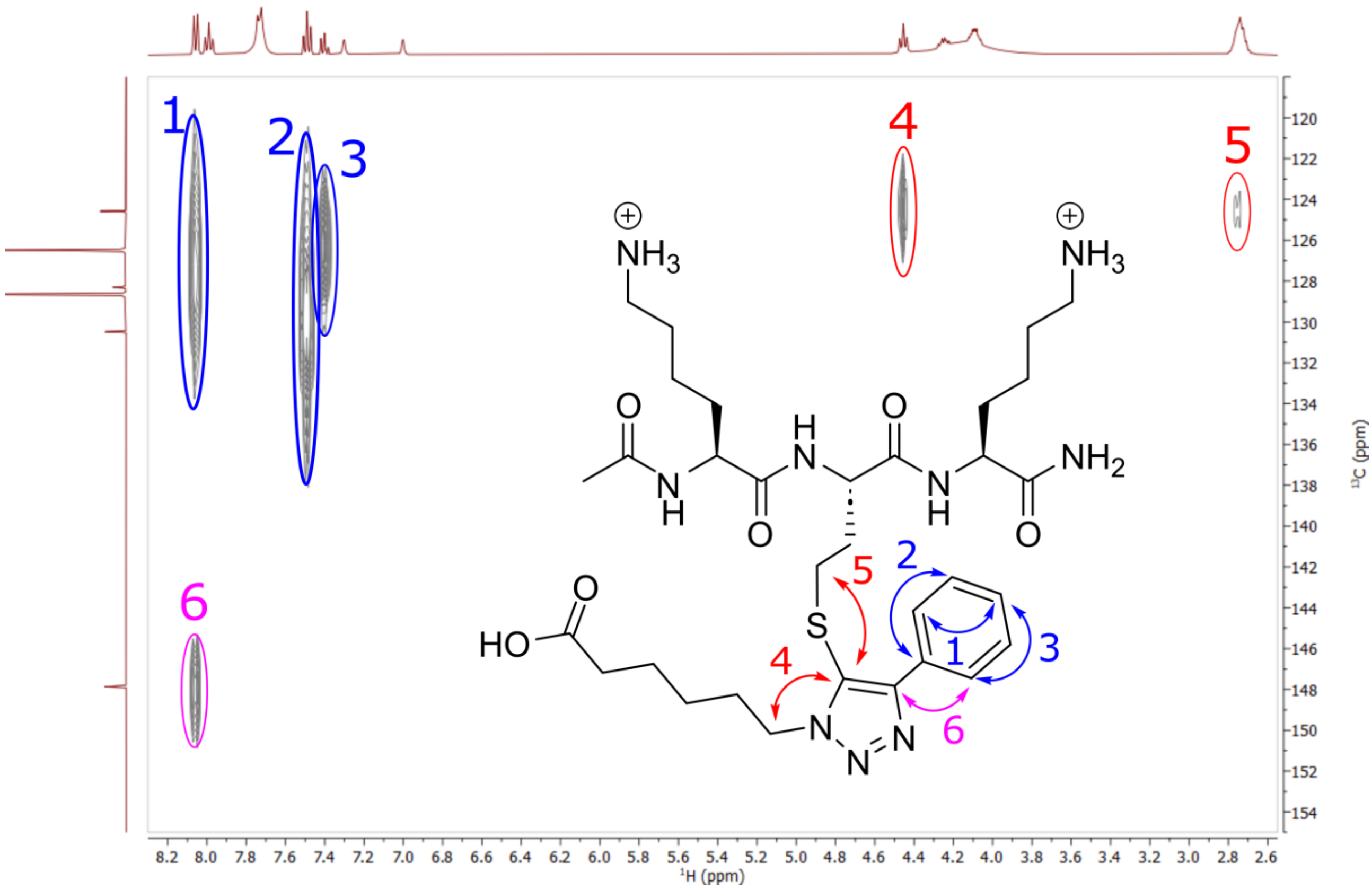

Figure S109: Extract from the ^{1}H-^{13}C HMBC spectrum (400 MHz, 101 MHz, DMSO-d_6, 295 K) of **P5A1Az1** showing the cross-peaks relevant for isomer determination.

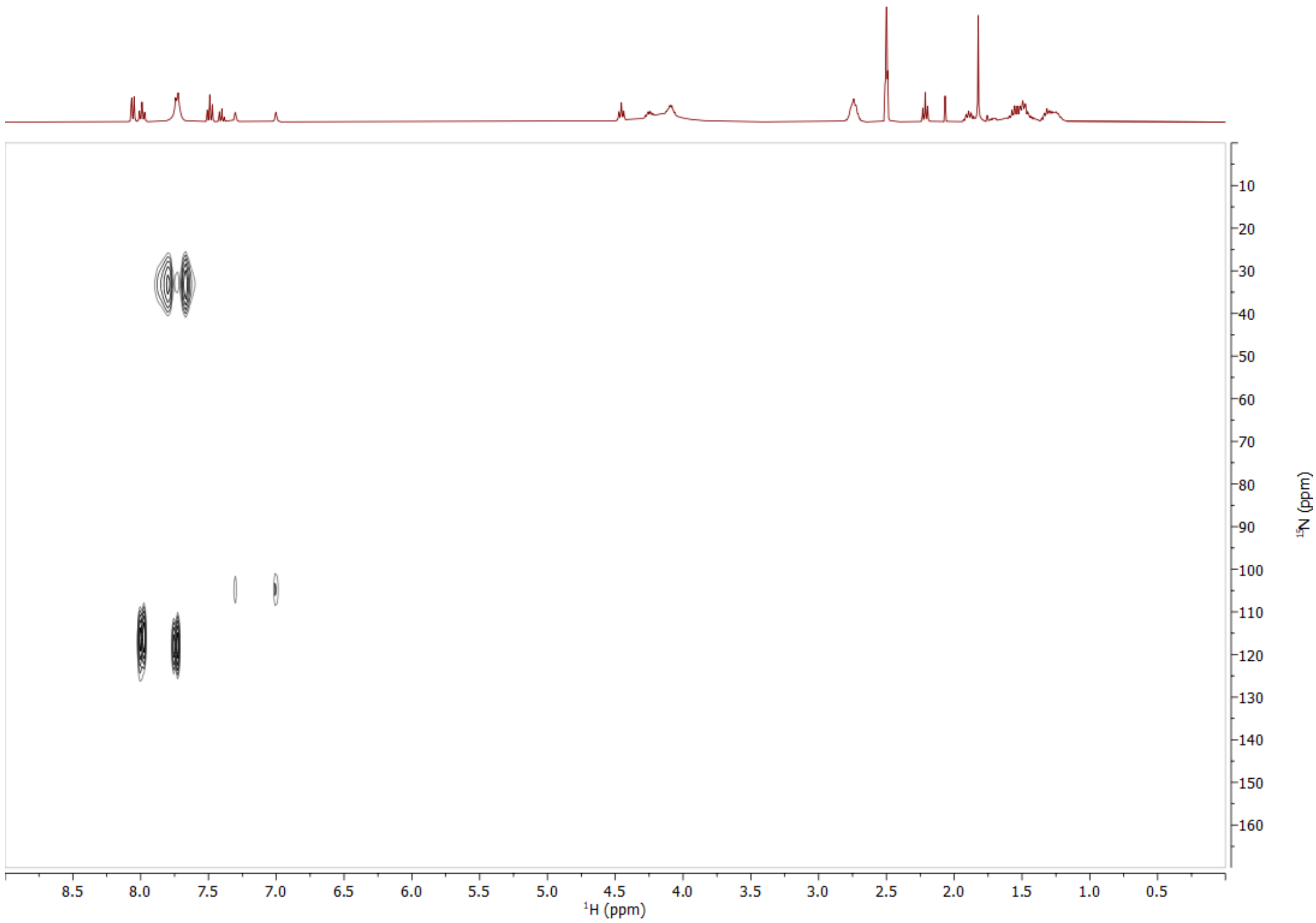

Figure S110: ^{1}H-^{15}N HSQC (400 MHz, 40.6 MHz, DMSO-d_6, 295 K) of **P5A1Az1**.

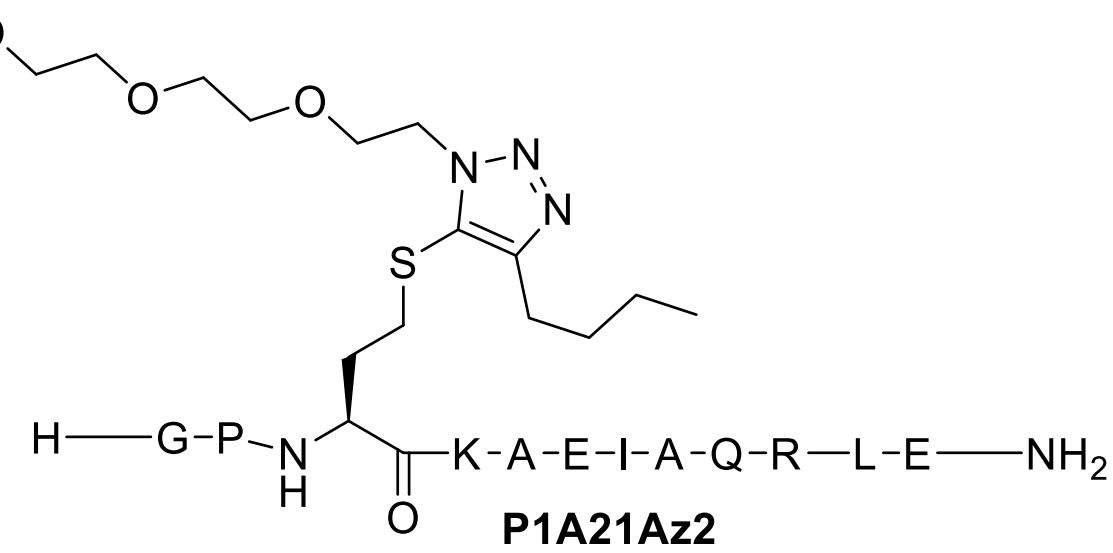

P1A21Az2

Chemical Formula: $C_{68}H_{119}N_{21}O_{20}S$
Exact Mass: 1581.87
Molecular Weight: 1582.89

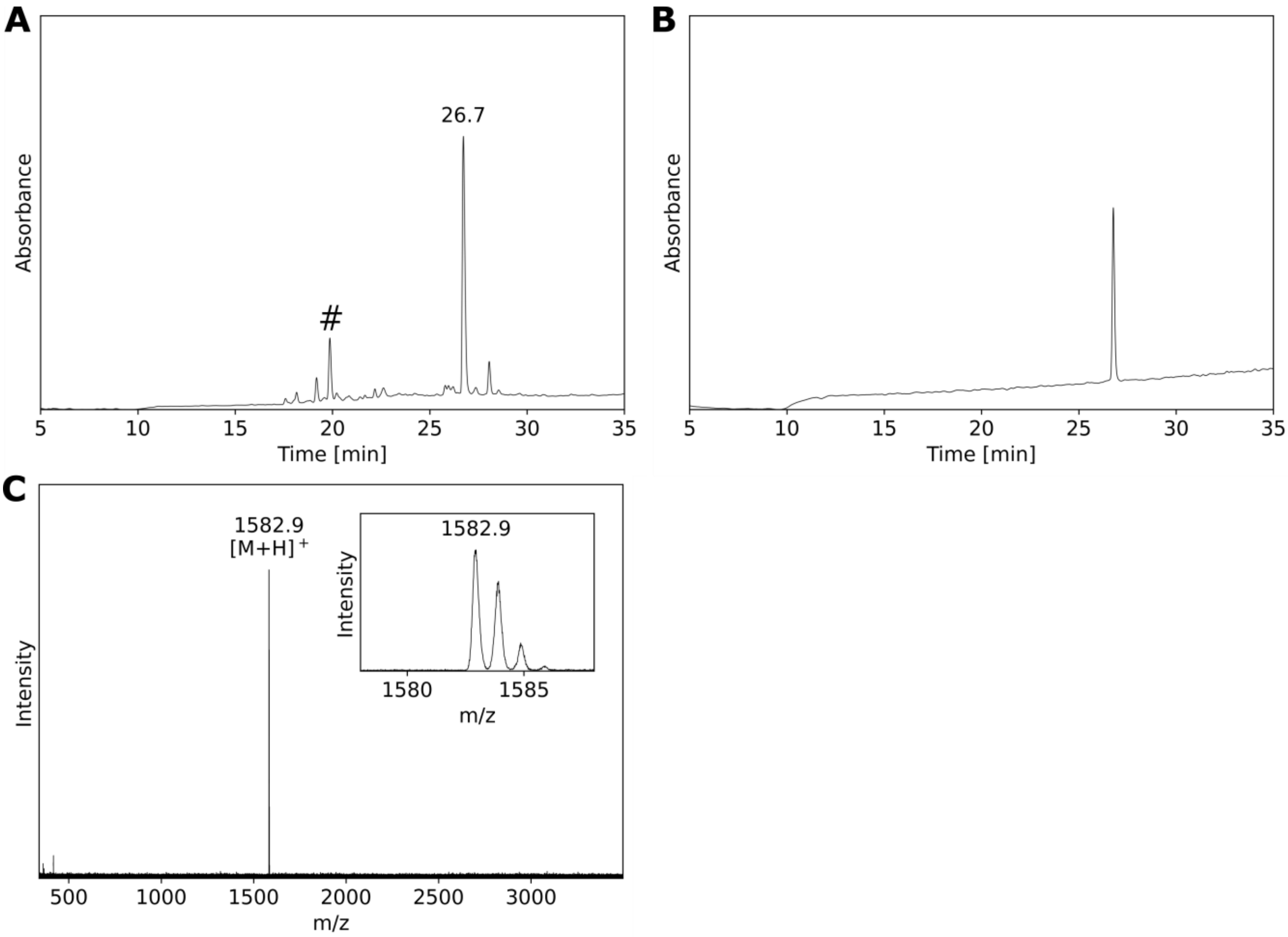

Figure S111: **P1A21Az2** A) Analytical HPLC (220 nm) of crude peptide. HPLC Gradients are listed in Section 2.3.5 Table S3. # Peptide without homoserine. B) Analytical HPLC (220 nm) of pure peptide. C) MALDI-TOF MS of pure peptide. Calculated mass: $[M+H]^+$ 1582.9.

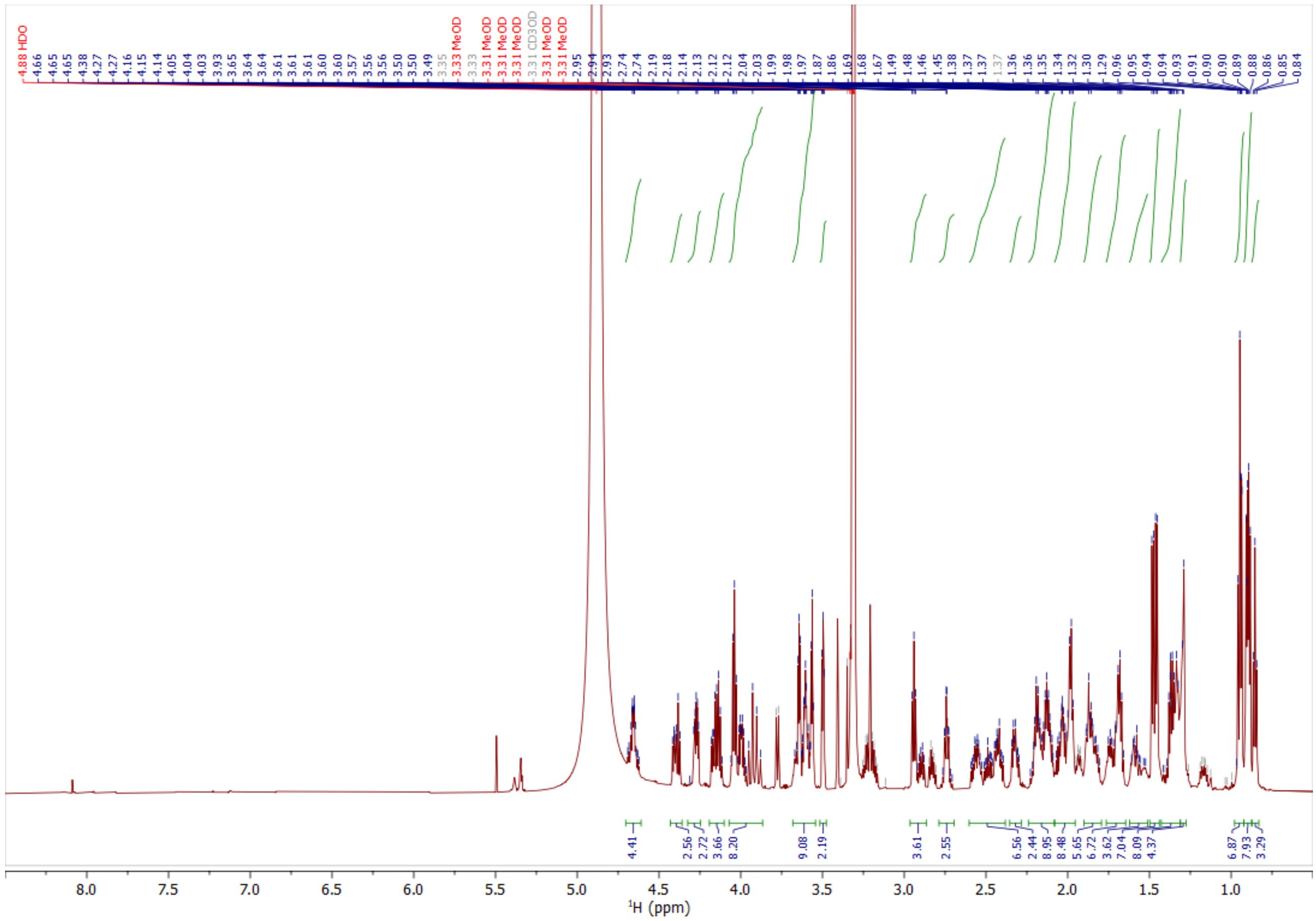

Figure S112: ^{1}H-NMR (700 MHz, MeOH-d_4, 295 K) of **P1A21Az2**. Hydrogen-deuterium exchange was performed prior to the measurement.

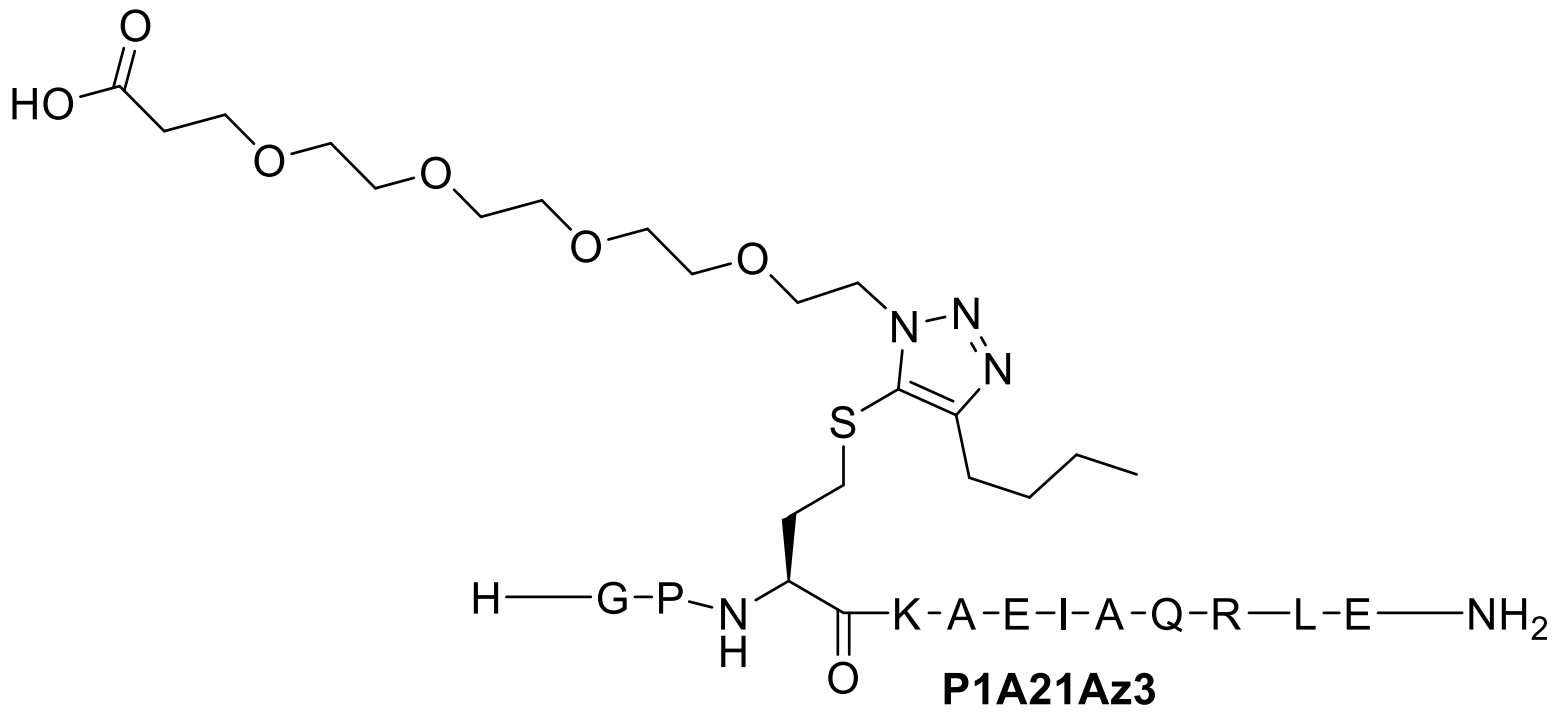

P1A21Az3

Chemical Formula: $C_{73}H_{127}N_{21}O_{23}S$
Exact Mass: 1697.91
Molecular Weight: 1699.00

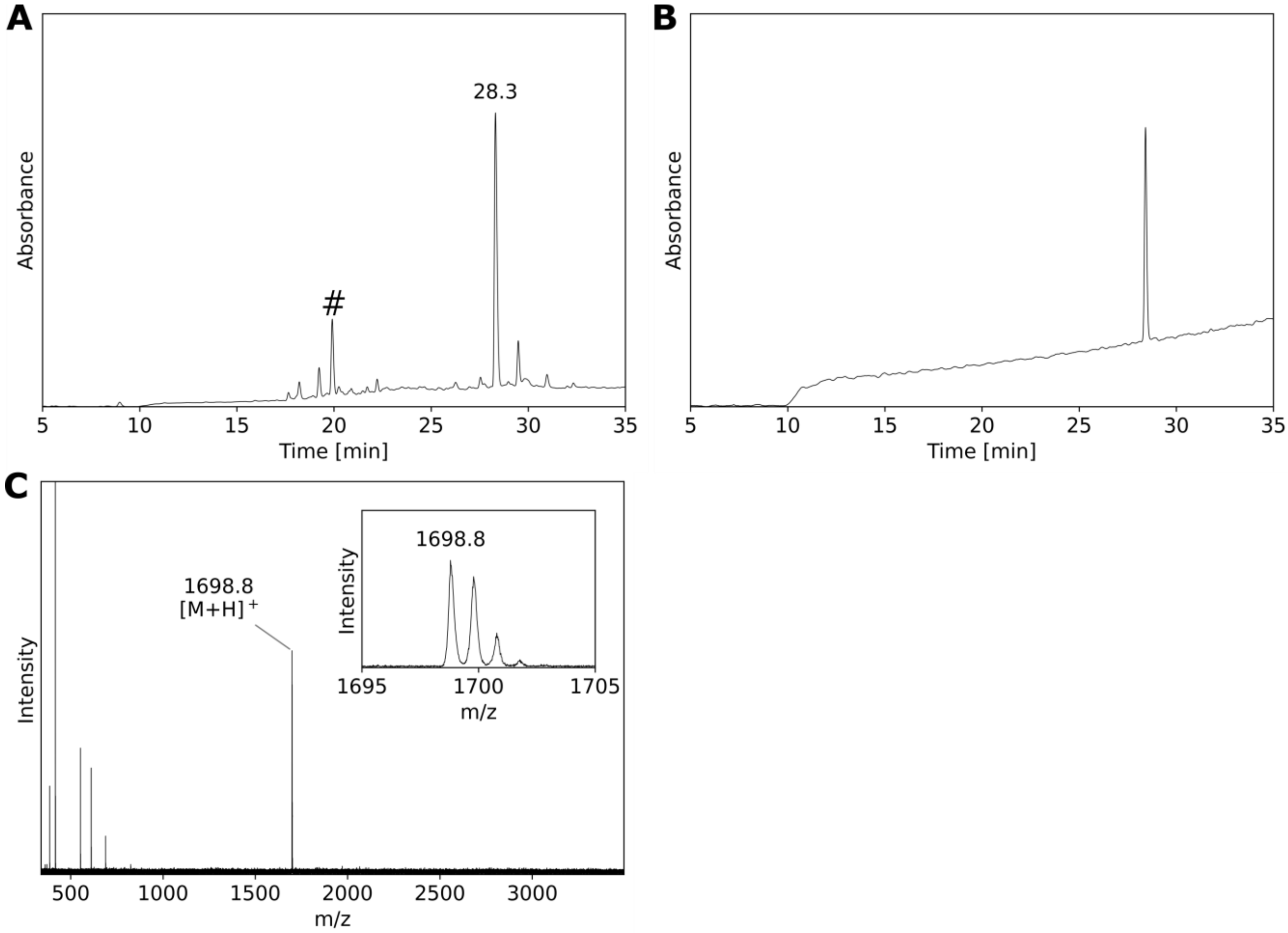

Figure S113: **P1A21Az3** A) Analytical HPLC (220 nm) of crude peptide. HPLC Gradients are listed in Section 2.3.5 Table S3. # Peptide without homoserine. B) Analytical HPLC (220 nm) of pure peptide. C) MALDI-TOF MS of pure peptide. Calculated mass: $[M+H]^+$ 1698.9.

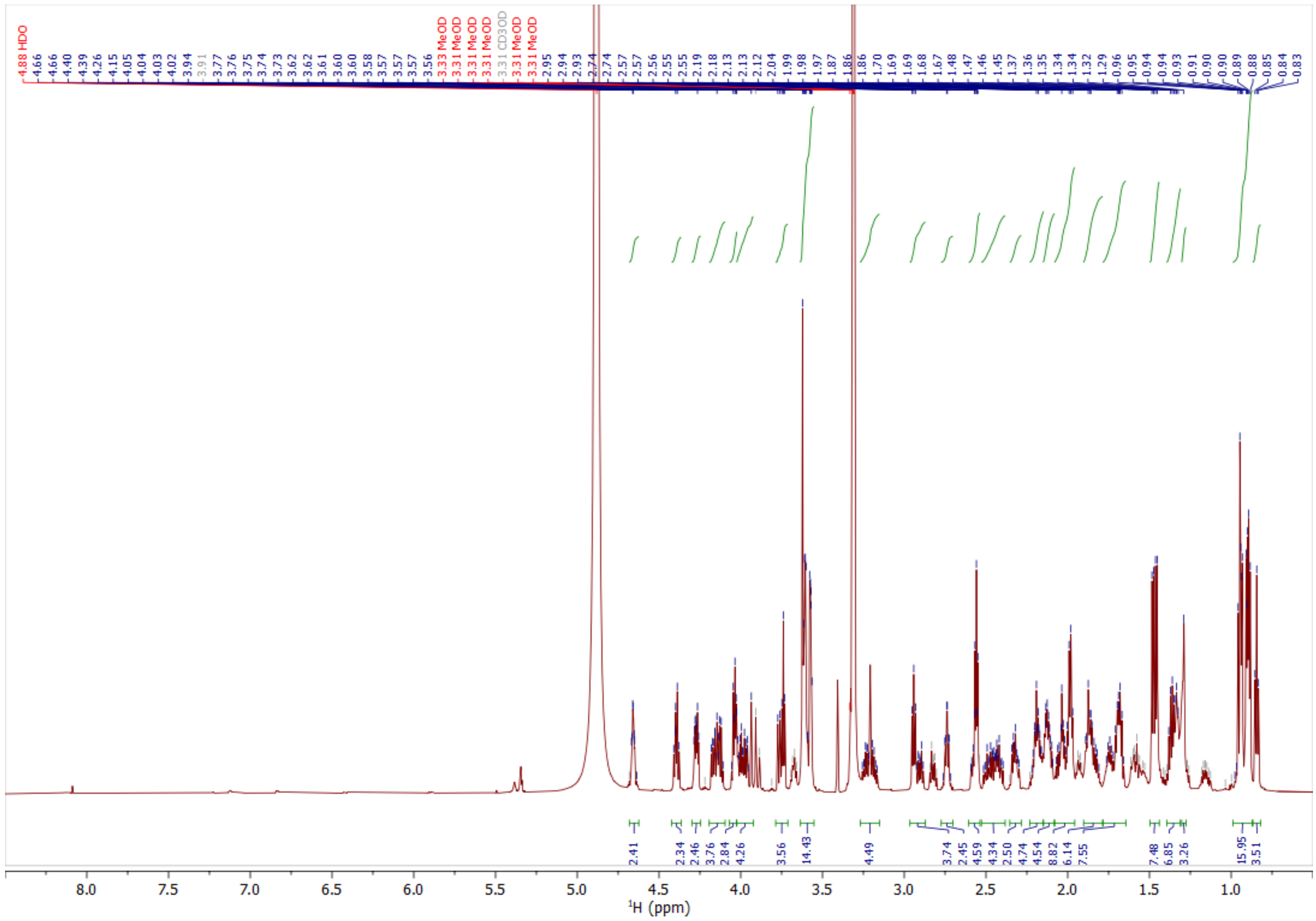

Figure S114: ^{1}H-NMR (700 MHz, MeOH-d_4, 295 K) of **P1A21Az3**. Hydrogen-deuterium exchange was performed prior to the measurement.

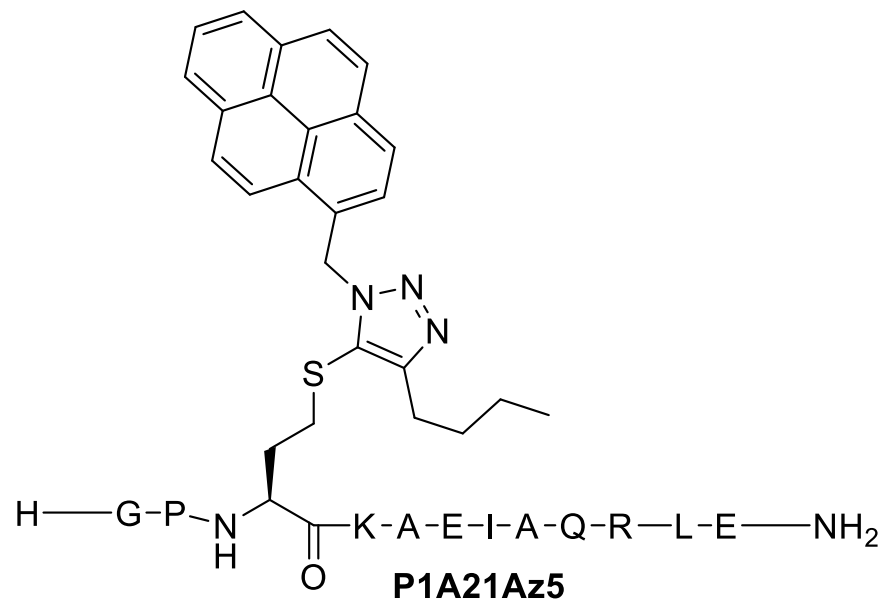

P1A21Az5

Chemical Formula: $C_{79}H_{117}N_{21}O_{17}S$

Exact Mass: 1663.87

Molecular Weight: 1664.99

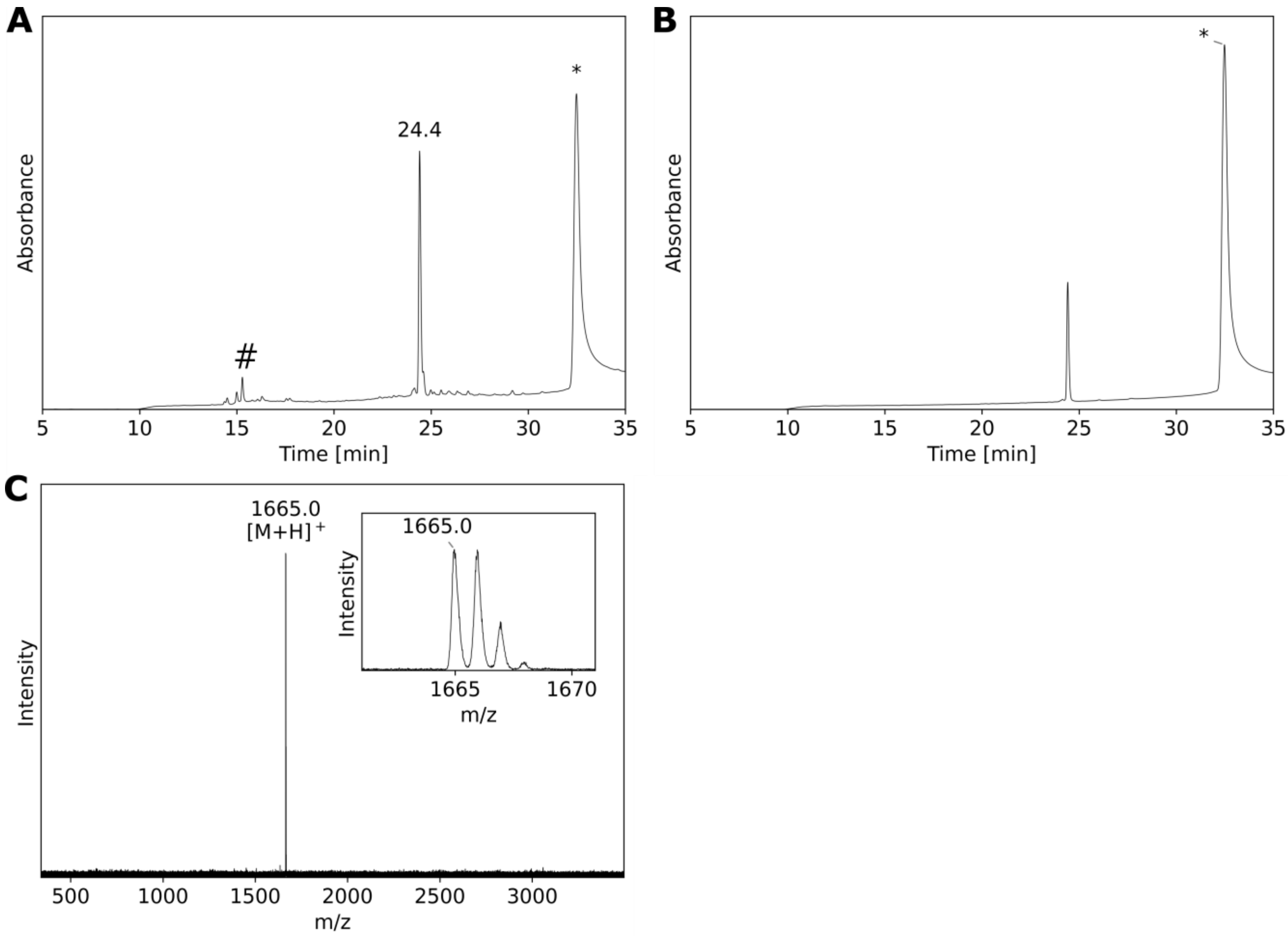

Figure S115: **P1A21Az5** A) Analytical HPLC (220 nm) of crude peptide. HPLC Gradients are listed in Section 2.3.5 Table S3. # Peptide without homoserine. B) Analytical HPLC (220 nm) of pure peptide. * Carryover peak. C) MALDI-TOF MS of pure peptide. Calculated mass: $[M+H]^+$ 1664.9.

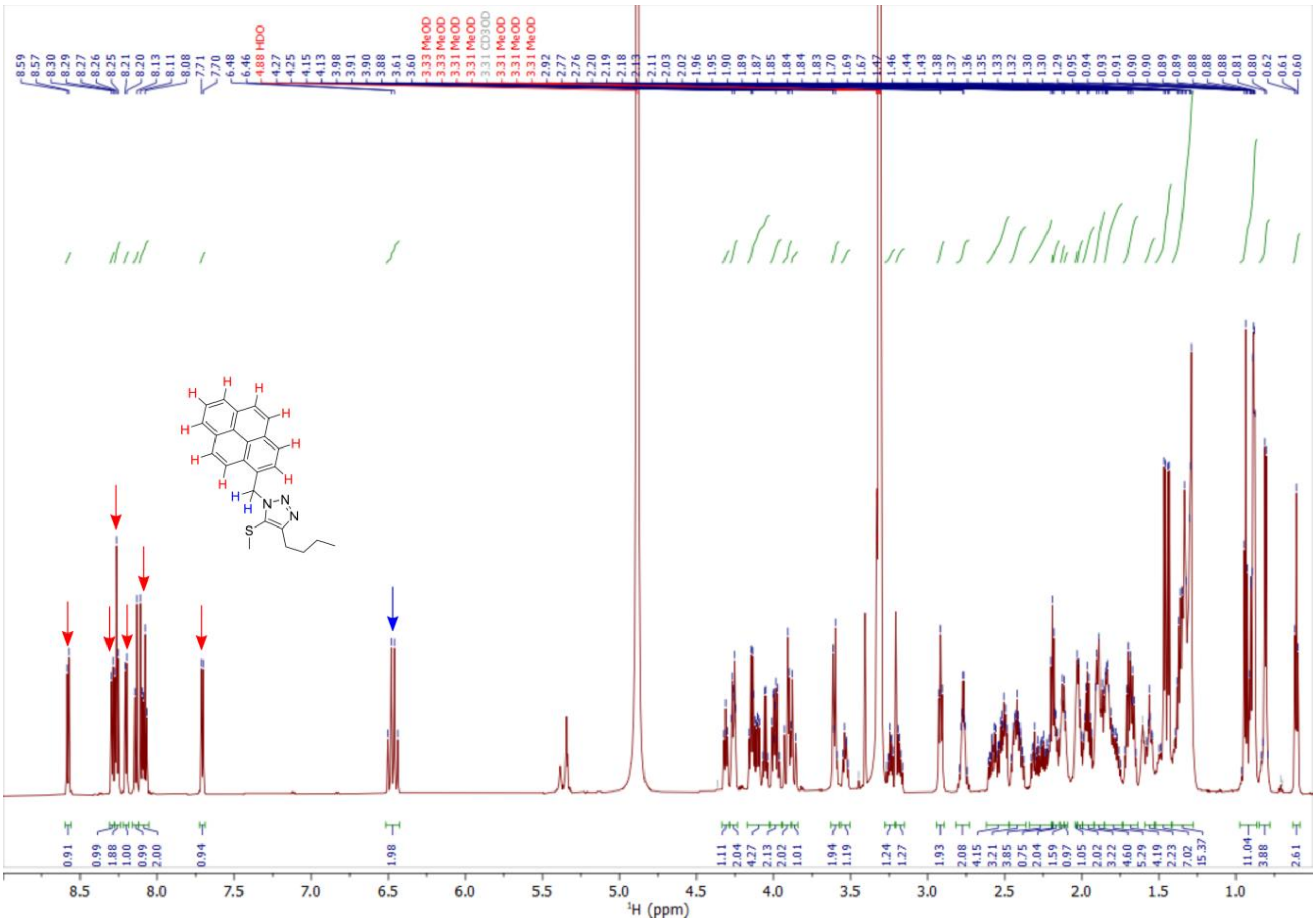

Figure S116: ^{1}H-NMR (700 MHz, MeOH-d_4, 295 K) of **P1A21Az5**. Hydrogen-deuterium exchange was performed prior to the measurement.

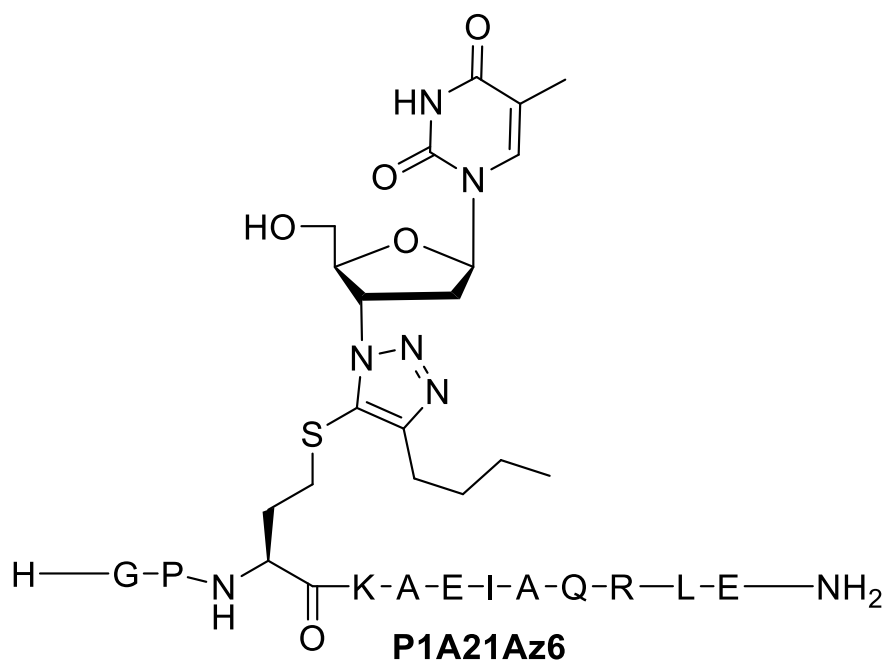

P1A21Az6

Chemical Formula: $C_{72}H_{119}N_{23}O_{21}S$

Exact Mass: 1673.87

Molecular Weight: 1674.94

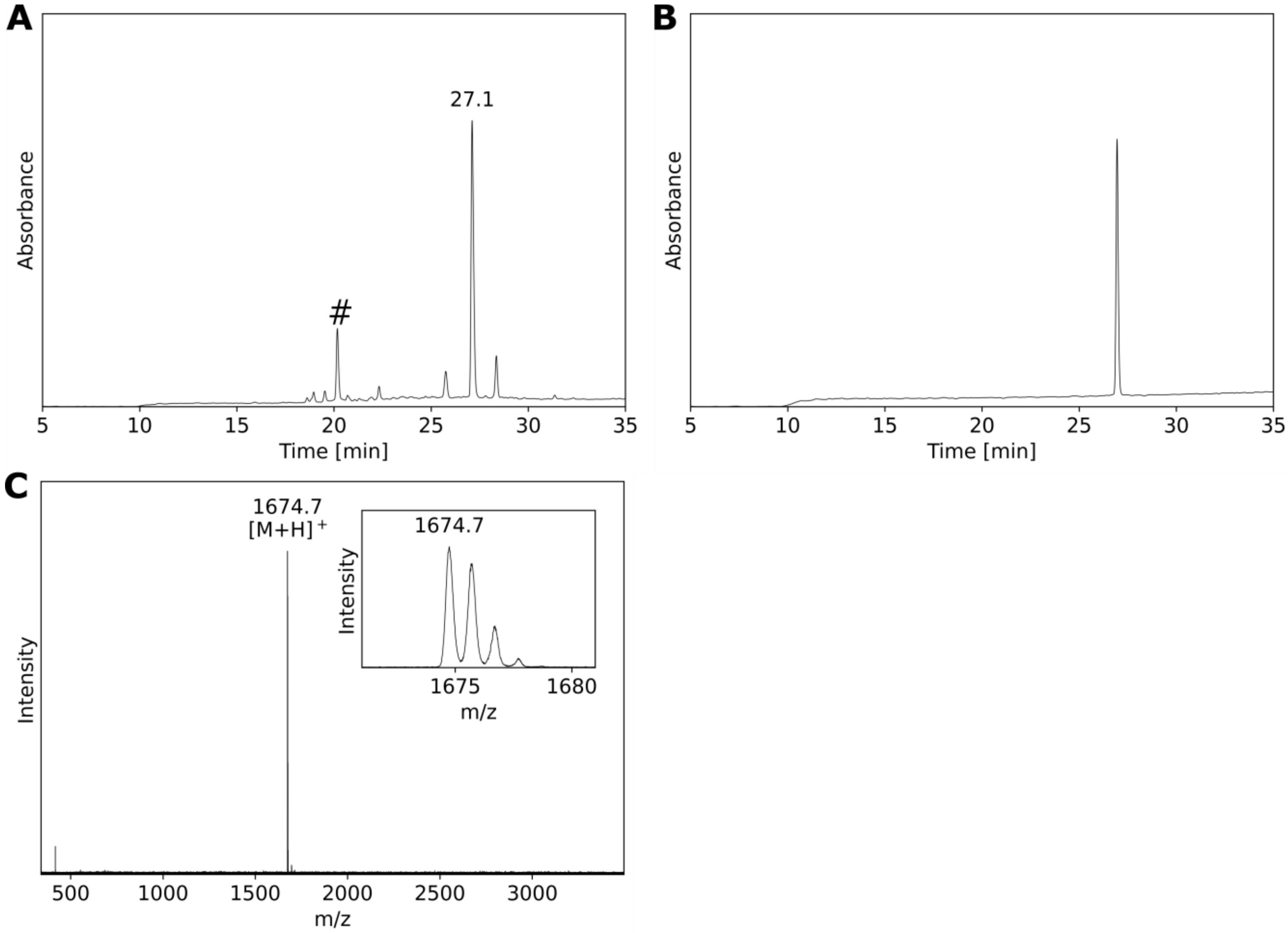

Figure S117: **P1A21Az6** A) Analytical HPLC (220 nm) of crude peptide. HPLC Gradients are listed in Section 2.3.5 Table S3. # Peptide without homoserine. B) Analytical HPLC (220 nm) of pure peptide. C) MALDI-TOF MS of pure peptide. Calculated mass: $[M+H]^+$ 1674.9.

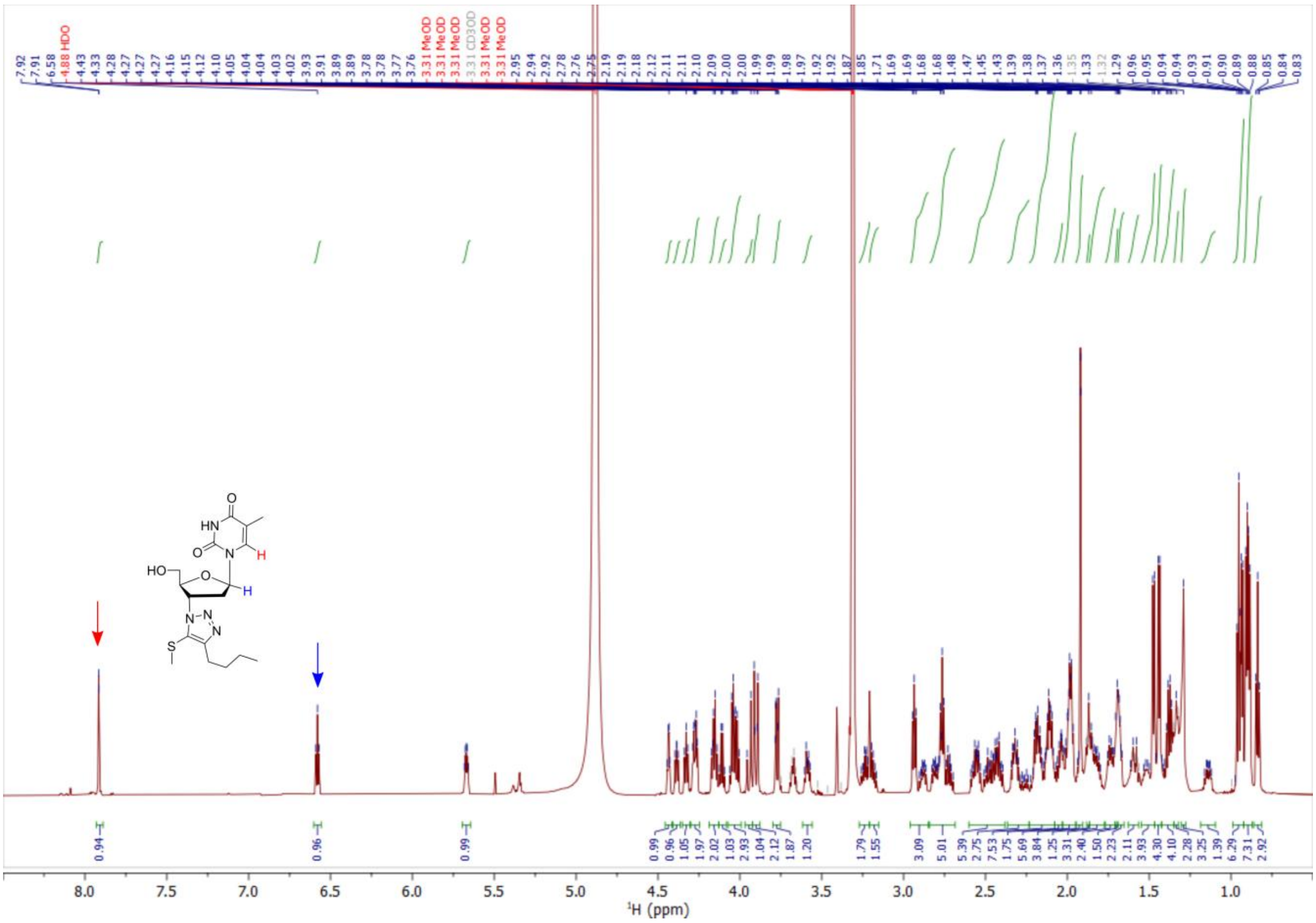

Figure S118: ^{1}H-NMR (700 MHz, MeOH-d_4, 295 K) of **P1A21Az6**. Hydrogen-deuterium exchange was performed prior to the measurement.

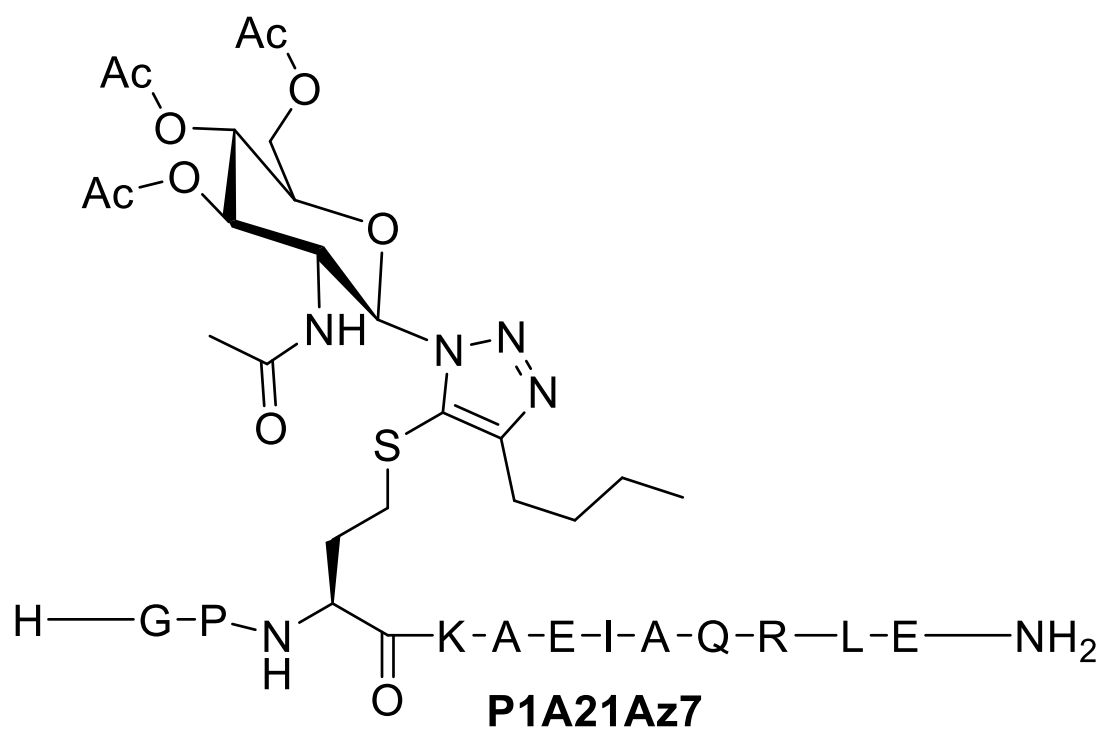

P1A21Az7

Chemical Formula: $C_{76}H_{126}N_{22}O_{25}S$

Exact Mass: 1778.90

Molecular Weight: 1780.03

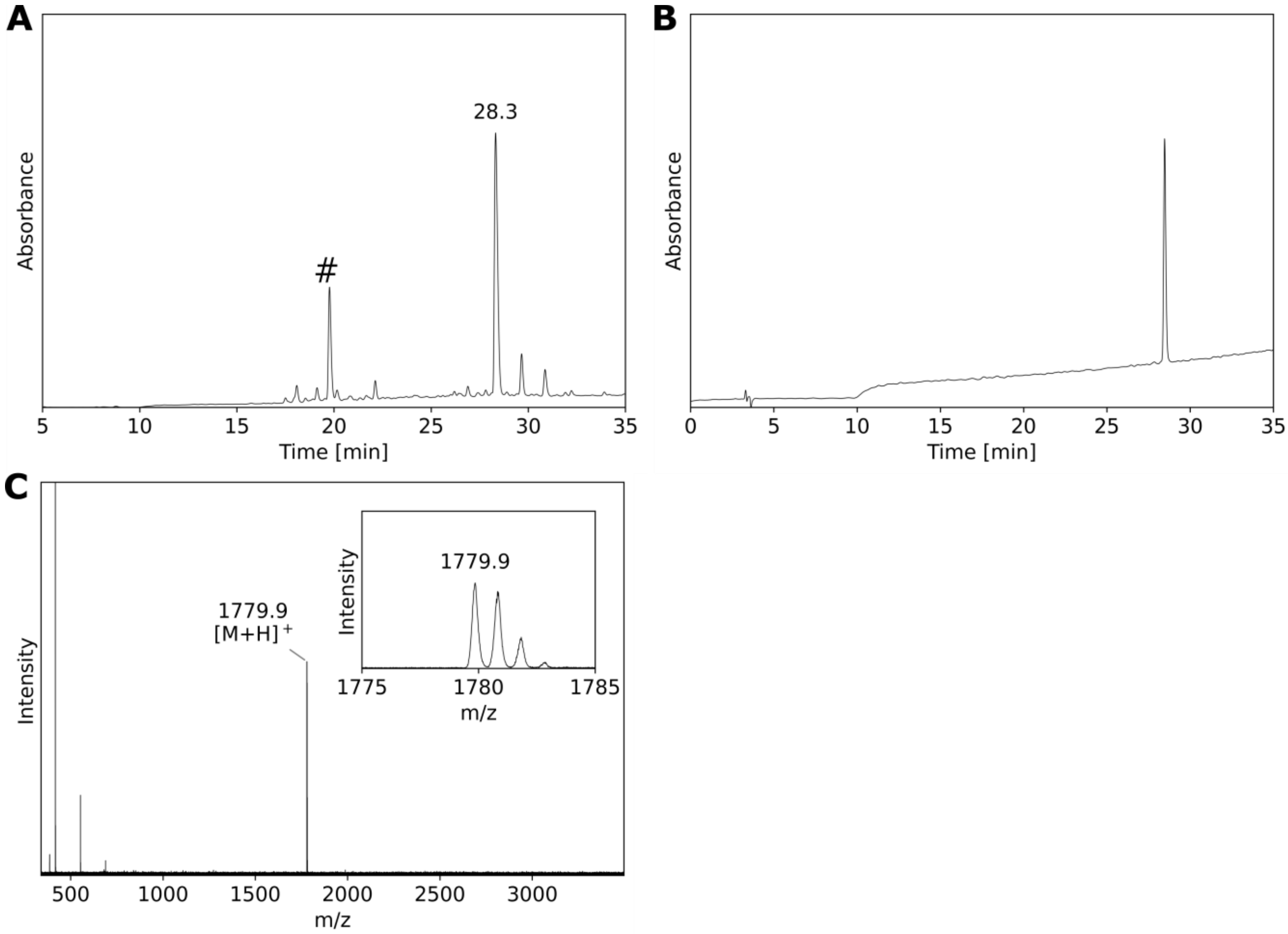

Figure S119: **P1A21Az7** A) Analytical HPLC (220 nm) of crude peptide. HPLC Gradients are listed in Section 2.3.5 Table S3. # Peptide without homoserine. B) Analytical HPLC (220 nm) of pure peptide. C) MALDI-TOF MS of pure peptide. Calculated mass: $[M+H]^+$ 1779.9.

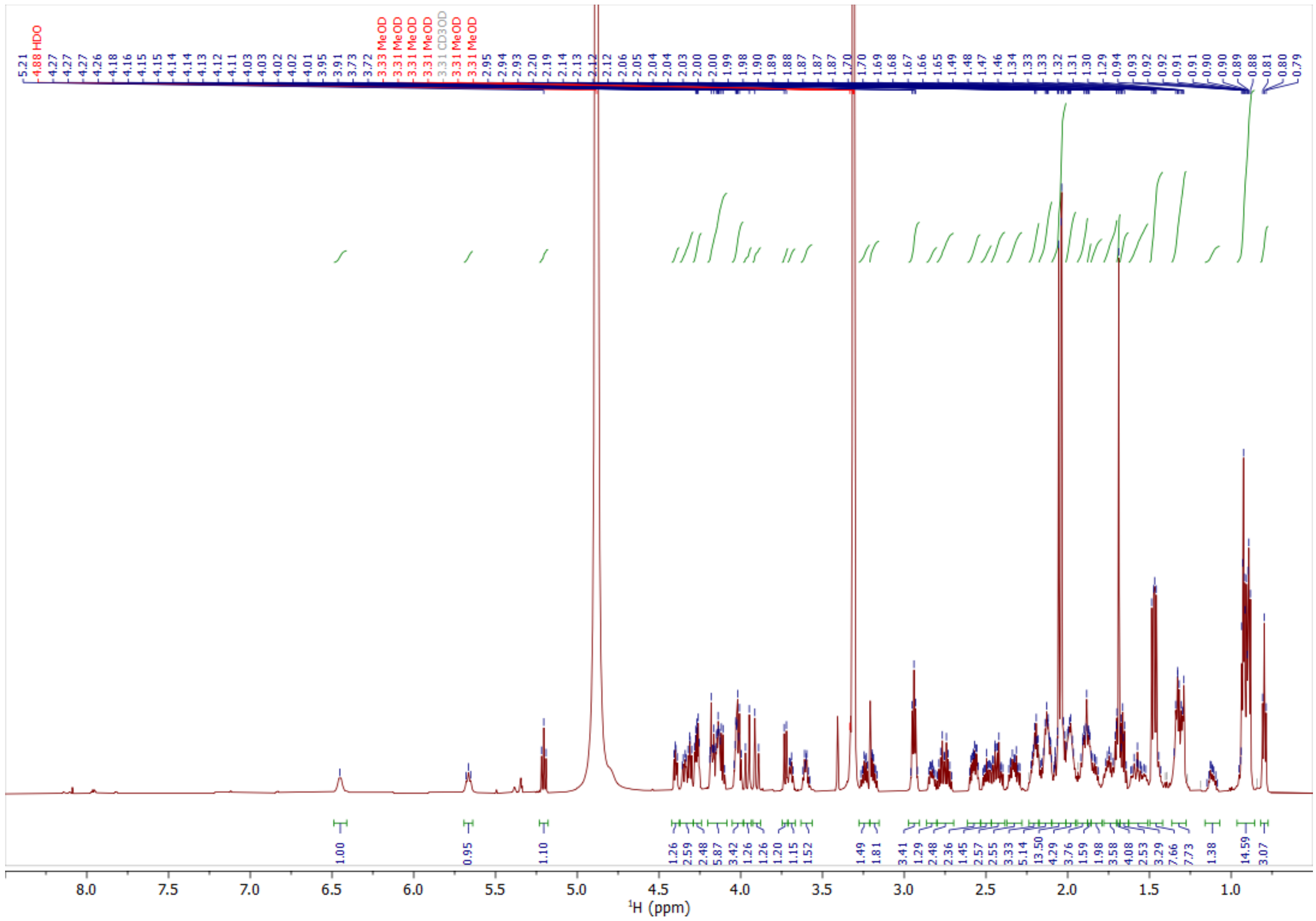

Figure S120: ^{1}H-NMR (700 MHz, MeOH-d_4, 295 K) of **P1A21Az7**. Hydrogen-deuterium exchange was performed prior to the measurement.

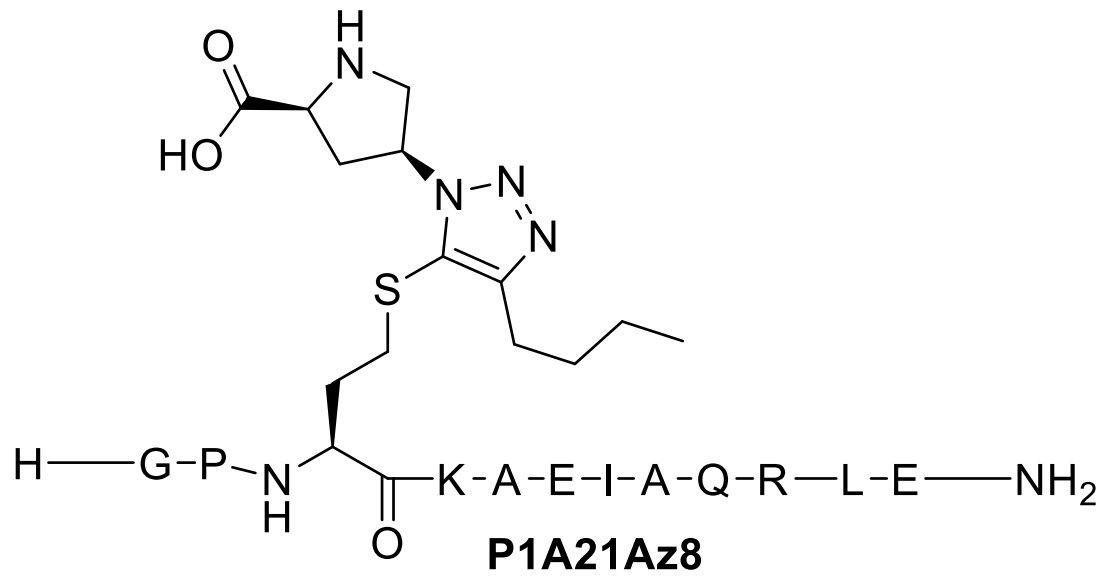

P1A21Az8

Chemical Formula: $C_{67}H_{114}N_{22}O_{19}S$

Exact Mass: 1562.84

Molecular Weight: 1563.84

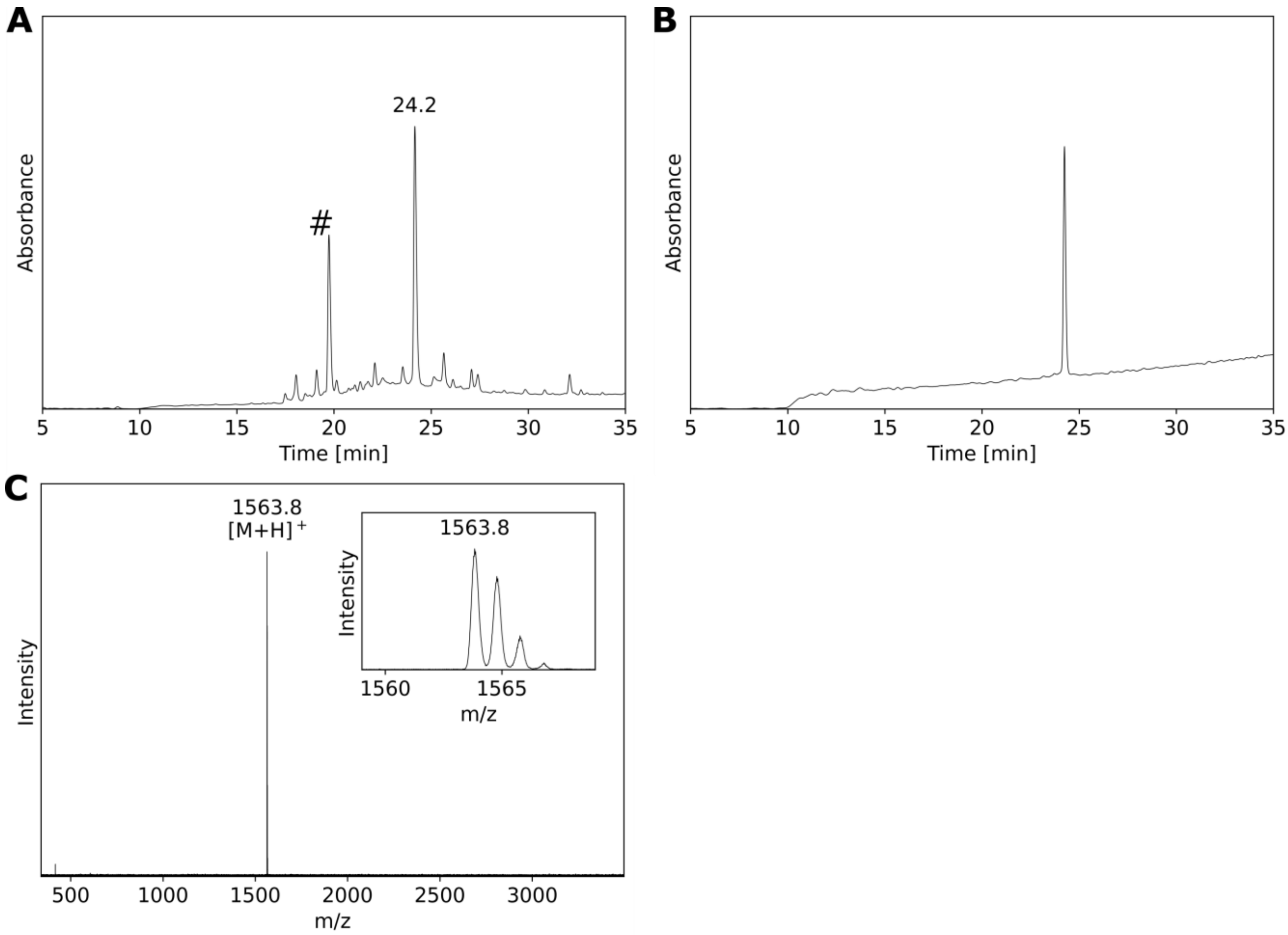

Figure S121: **P1A21Az8** prepared with Boc-azido-proline **Az8**. HPLC Gradients are listed in Section 2.3.5 Table S3. # Peptide without homoserine. A) Analytical HPLC (220 nm) of crude peptide. B) Analytical HPLC (220 nm) of pure peptide. C) MALDI-TOF MS of pure peptide. Calculated mass: $[M+H]^+$ 1563.8.

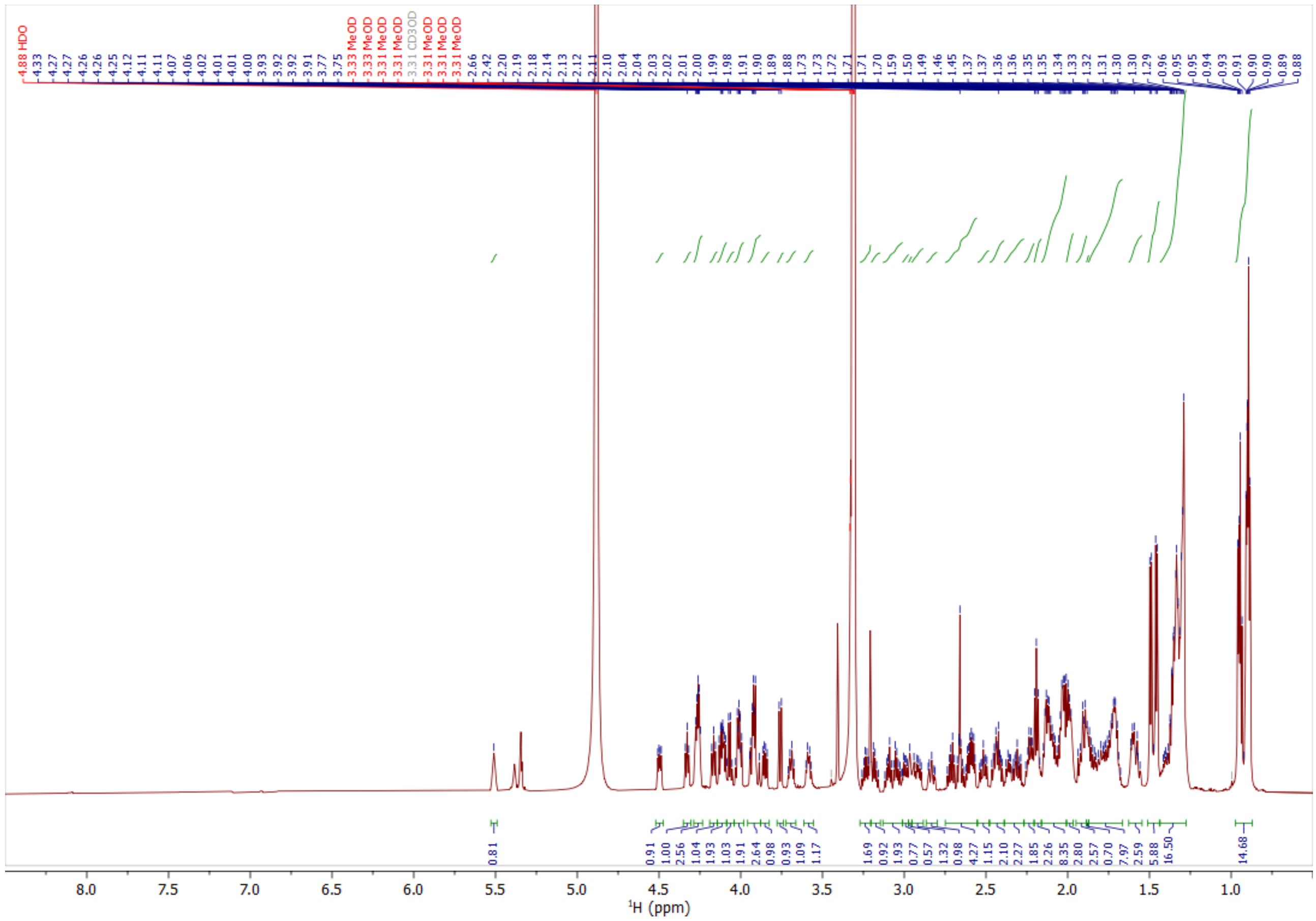

Figure S122: : ^{1}H-NMR (700 MHz, MeOH-d_4, 295 K) of **P1A21Az8**. Hydrogen-deuterium exchange was performed prior to the measurement.

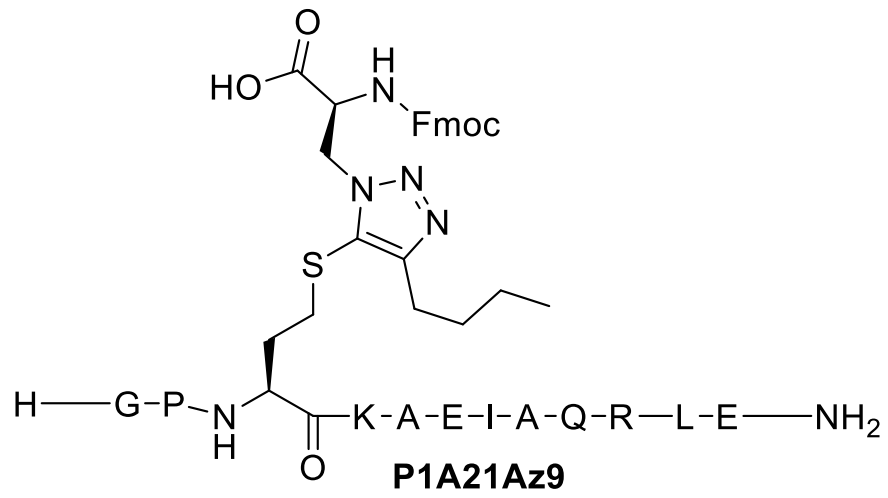

P1A21Az9

Chemical Formula: $C_{80}H_{122}N_{22}O_{21}S$

Exact Mass: 1758.89

Molecular Weight: 1760.05

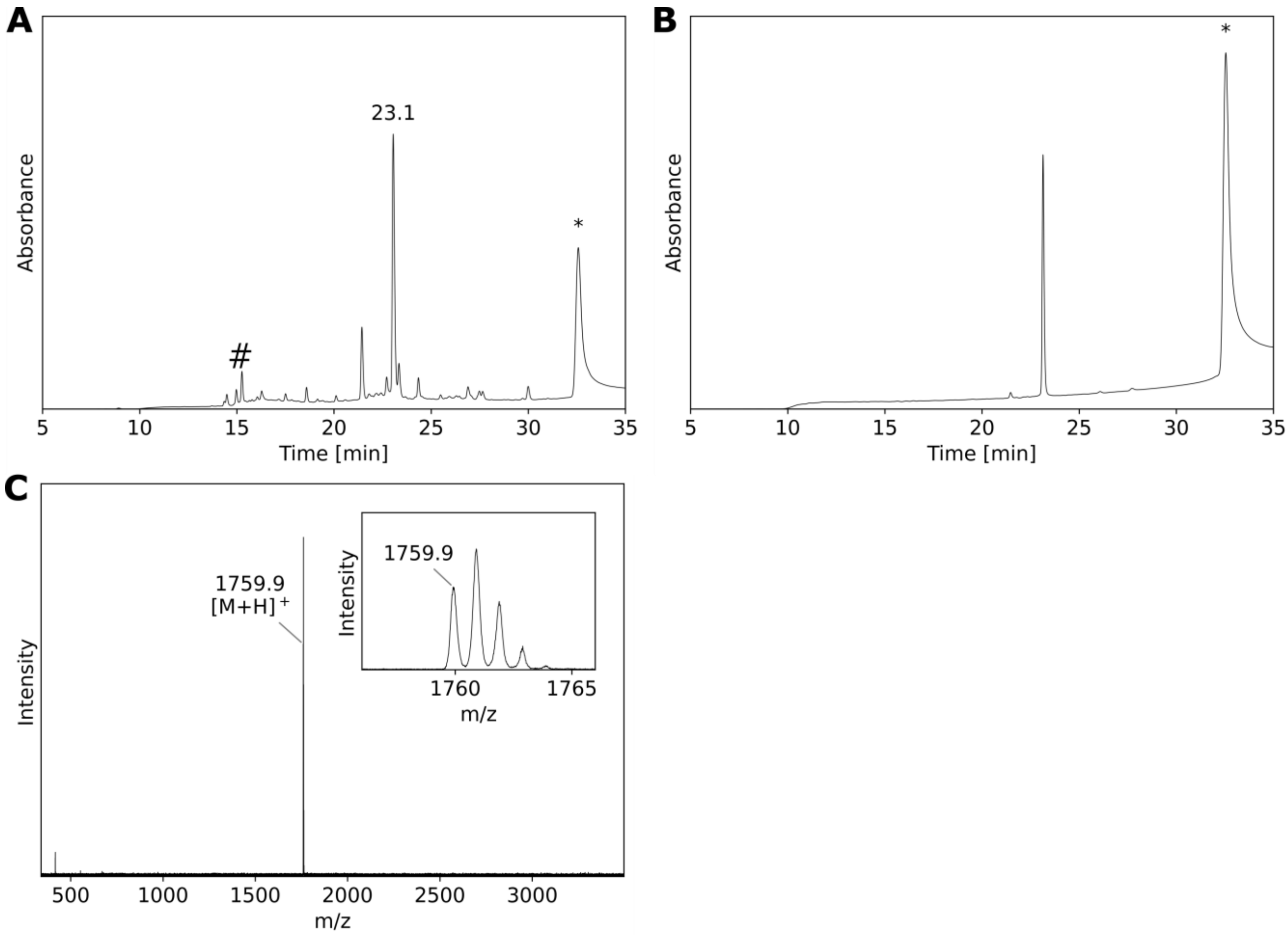

Figure S123: **P1A21Az9** A) Analytical HPLC (220 nm) of crude peptide. HPLC Gradients are listed in Section 2.3.5 Table S3. # Peptide without homoserine. B) Analytical HPLC (220 nm) of pure peptide. * Carryover peak. C) MALDI-TOF MS of pure peptide. Calculated mass: $[M+H]^+$ 1759.9.

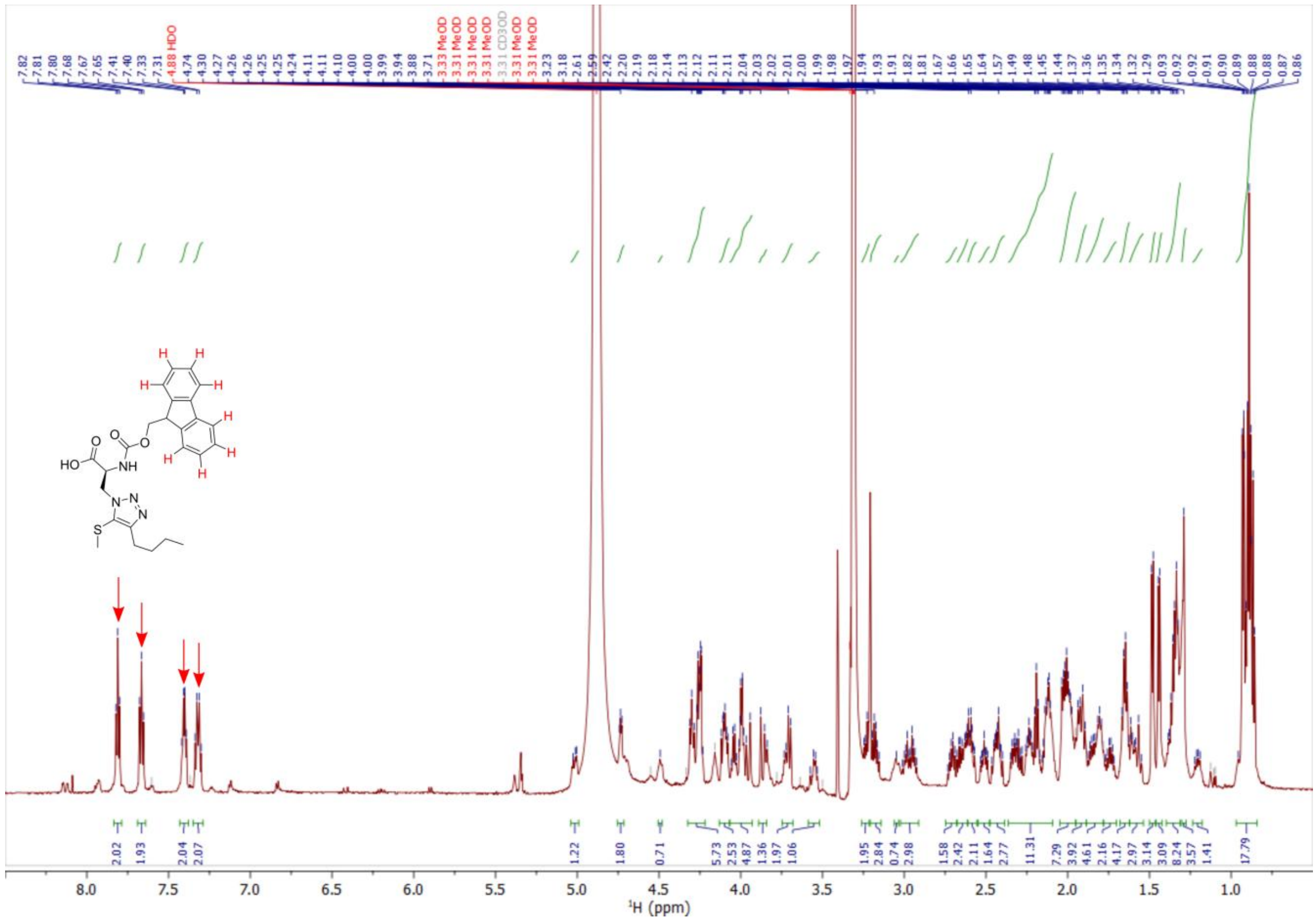

Figure S124: ^{1}H-NMR (700 MHz, MeOH-d_4, 295 K) of **P1A21Az9**. Hydrogen-deuterium exchange was performed prior to the measurement.

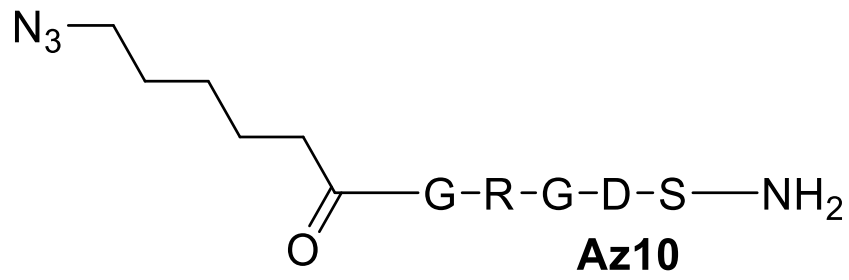

Az10

Chemical Formula: $C_{23}H_{40}N_{12}O_9$
Exact Mass: 628.30
Molecular Weight: 628.65

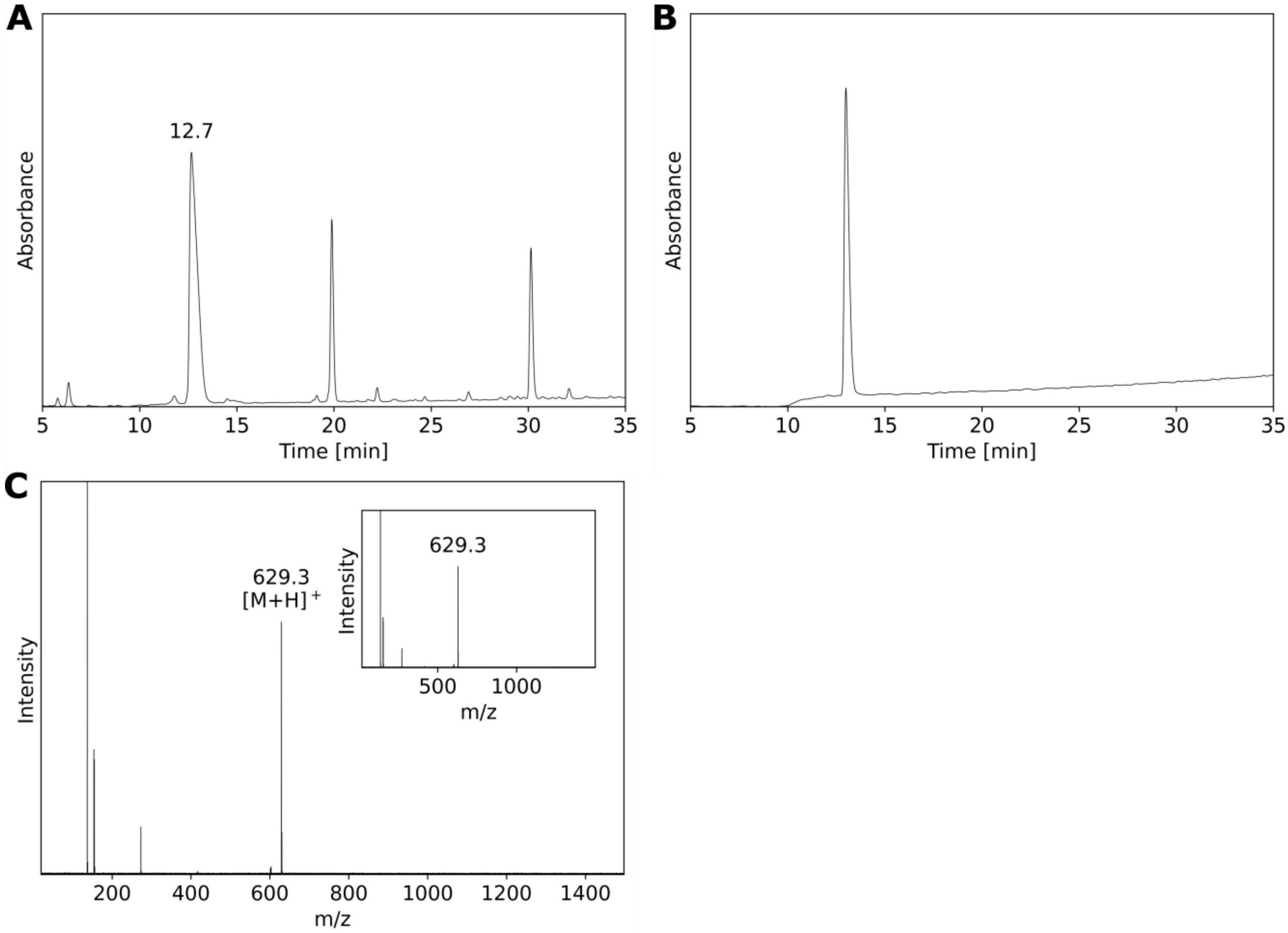

Figure S125: **Az10** A) Analytical HPLC (220 nm) of crude peptide. HPLC Gradients are listed in Section 2.3.5 Table S3. B) Analytical HPLC (220 nm) of pure peptide. C) MALDI-TOF MS of pure peptide. Calculated mass: $[M+H]^+$ 629.3.

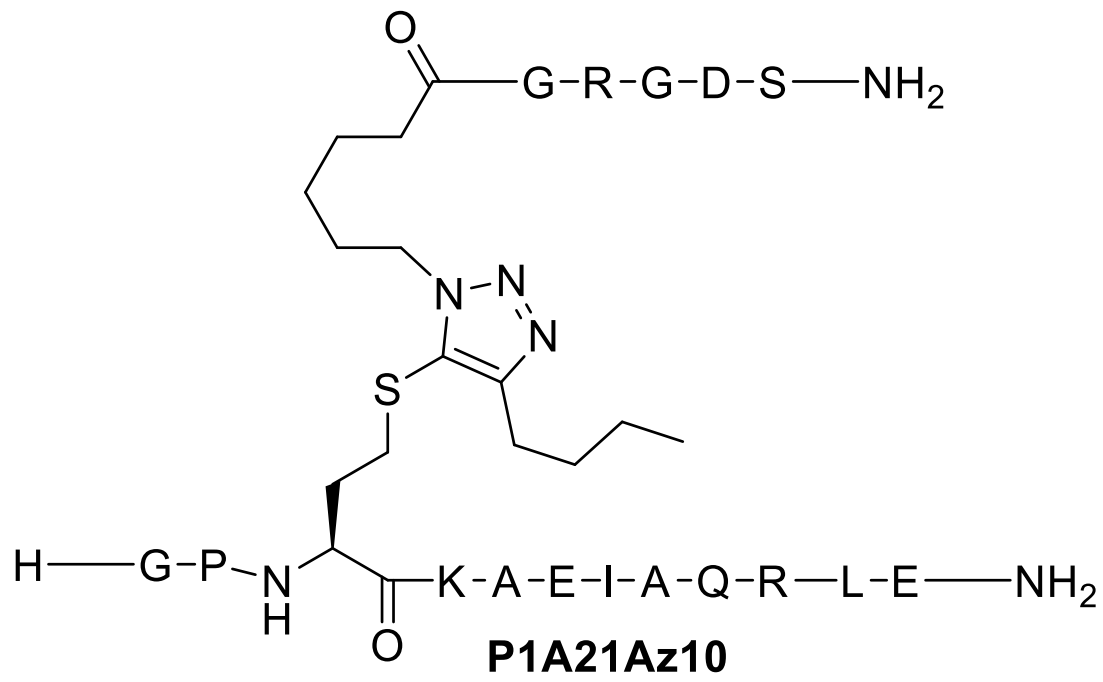

P1A21Az10

Chemical Formula: $C_{85}H_{146}N_{30}O_{26}S$

Exact Mass: 2035.07

Molecular Weight: 2036.35

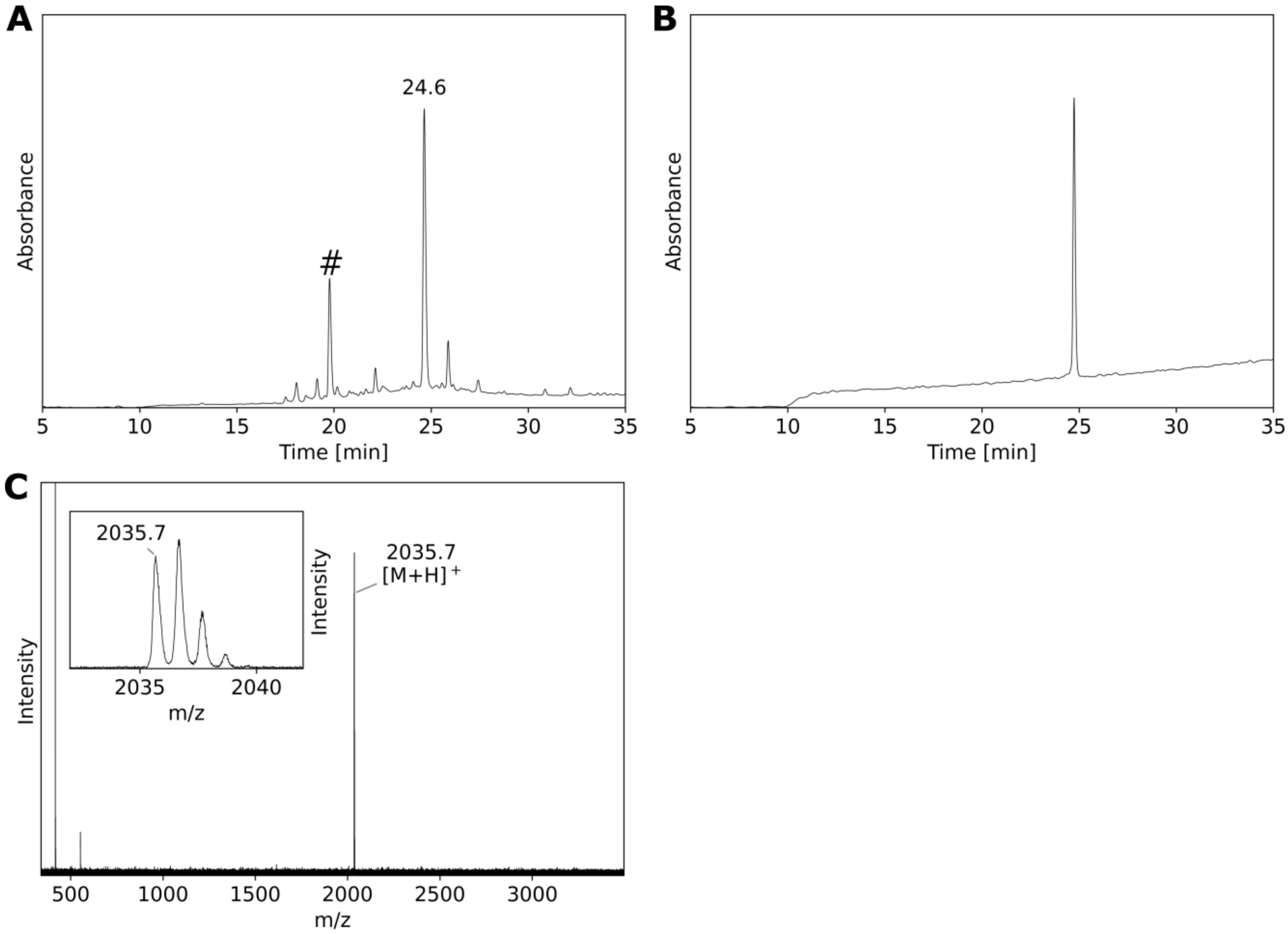

Figure S126: **P1A21Az10** A) Analytical HPLC (220 nm) of crude peptide. HPLC Gradients are listed in Section 2.3.5 Table S3. # Peptide without homoserine. B) Analytical HPLC (220 nm) of pure peptide. C) MALDI-TOF MS of pure peptide. Calculated mass: $[M+H]^+$ 2036.1.

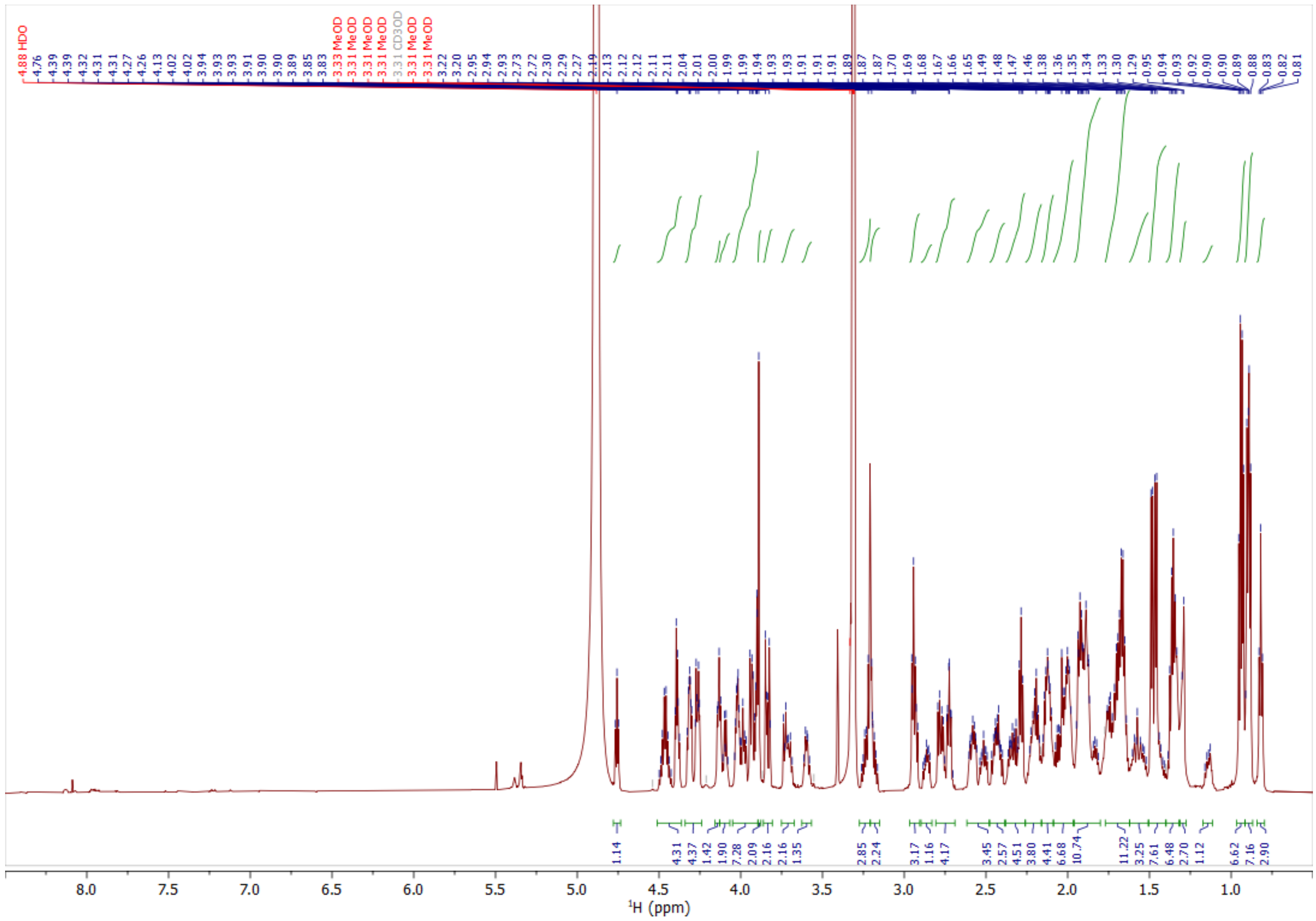

Figure S127: ^{1}H-NMR (700 MHz, MeOH-d_4, 295 K) of **P1A21Az10**. Hydrogen-deuterium exchange was performed prior to the measurement.

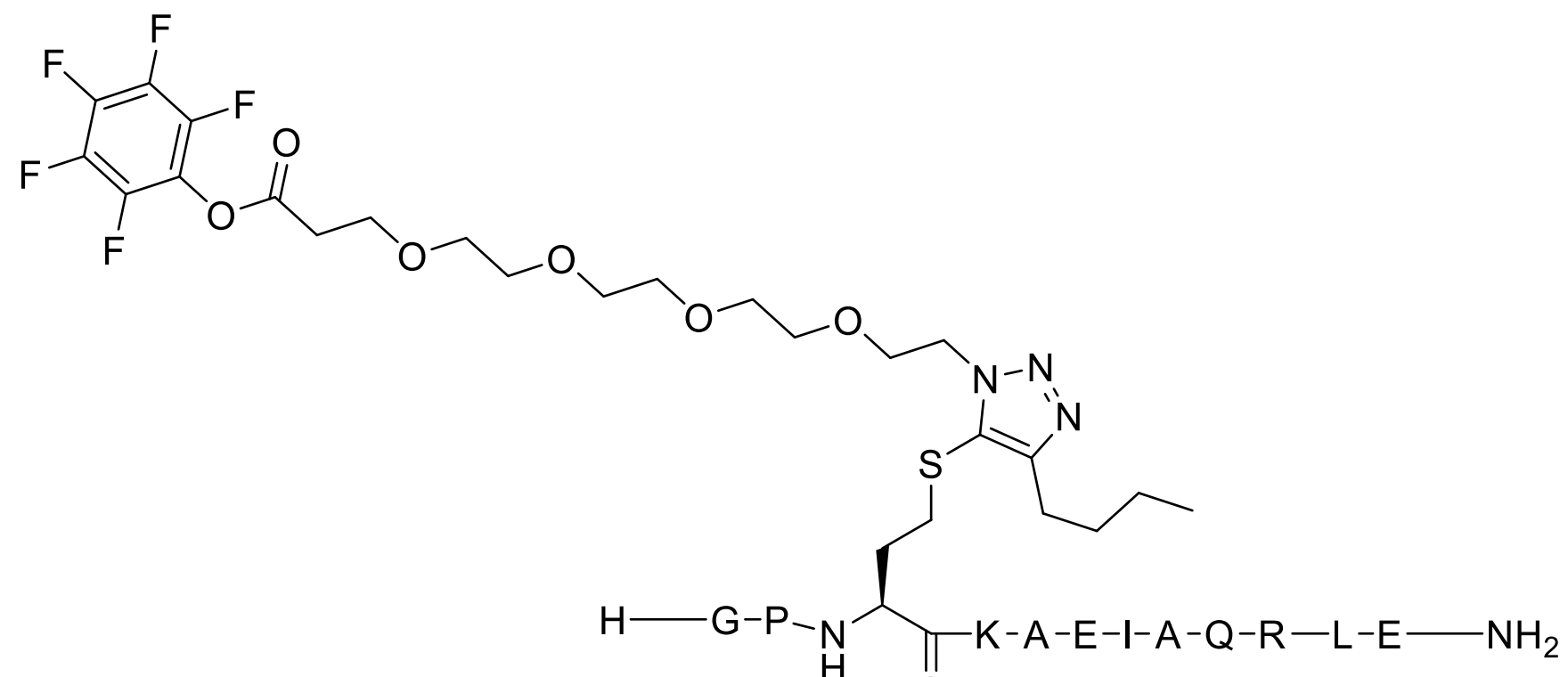

P1A21Az11

Chemical Formula: $C_{79}H_{126}F_5N_{21}O_{23}S$

Exact Mass: 1863.90

Molecular Weight: 1865.05

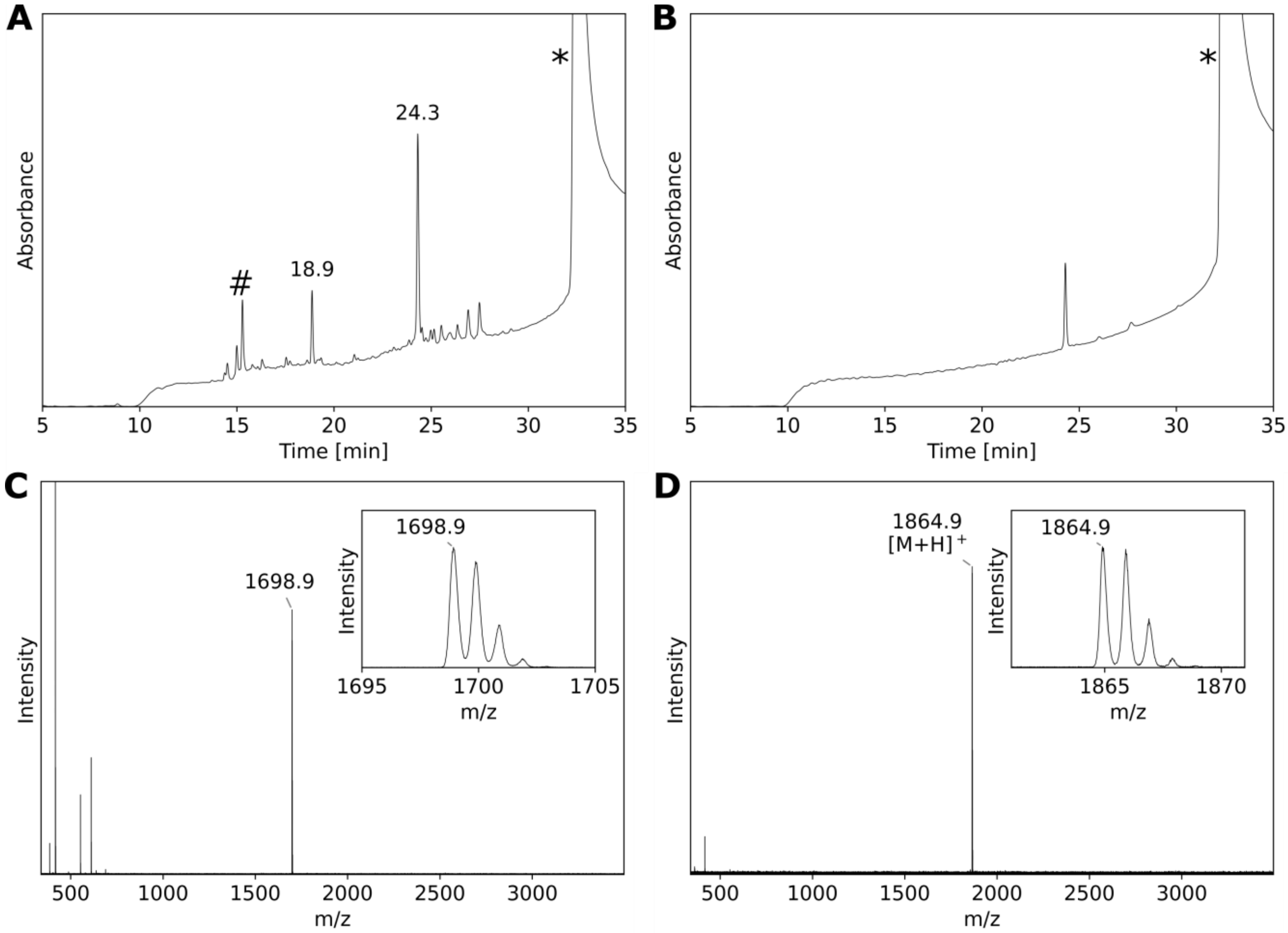

Figure S128: **P1A21Az11** A) Analytical HPLC (220 nm) of crude peptide. HPLC Gradients are listed in Section 2.3.5 Table S3. # Peptide without homoserine. B) Analytical HPLC (220 nm) of pure peptide. * Carryover peak. C) MALDI-TOF MS of purified hydrolyzed peptide with a retention time of 18.9 min. D) MALDI-TOF MS of purified peptide with a retention time of 24.3 min. Calculated mass: $[M+H]^+$ 1864.9, $[M-C_6F_5OH+H_2O+H]^+$ 1698.9.

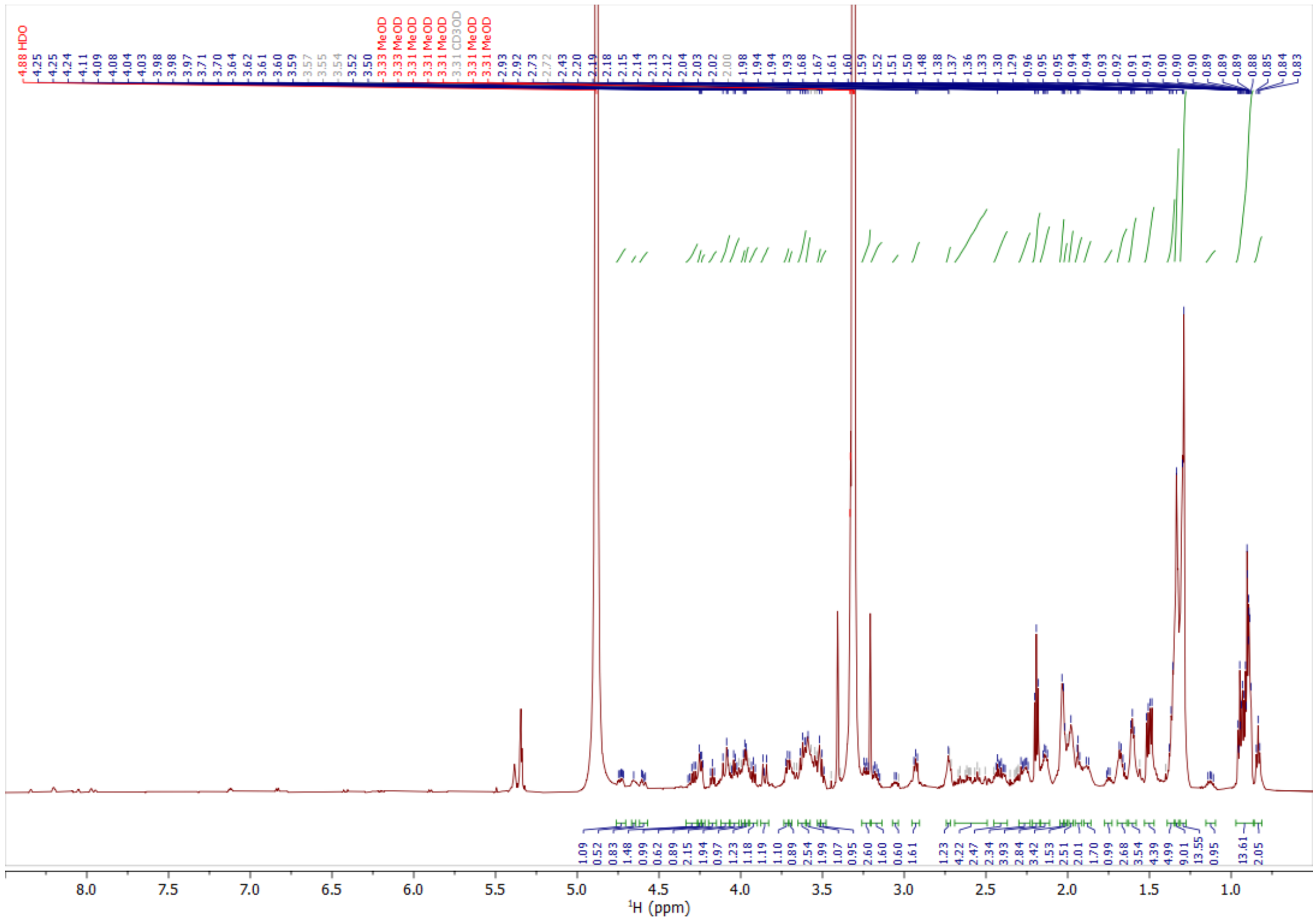

Figure S129: ^{1}H-NMR (700 MHz, MeOH-d_4, 295 K) of **P1A21Az11**. Hydrogen-deuterium exchange was performed prior to the measurement.

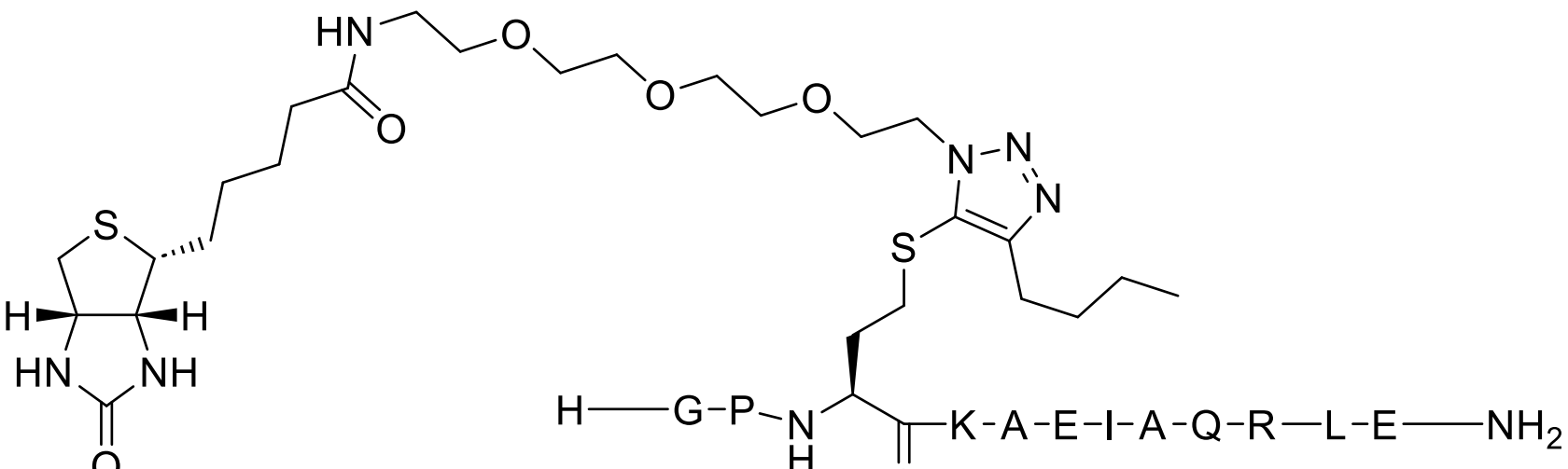

P1A21Az12

Chemical Formula: $C_{80}H_{138}N_{24}O_{22}S_2$
Exact Mass: 1850.99
Molecular Weight: 1852.25

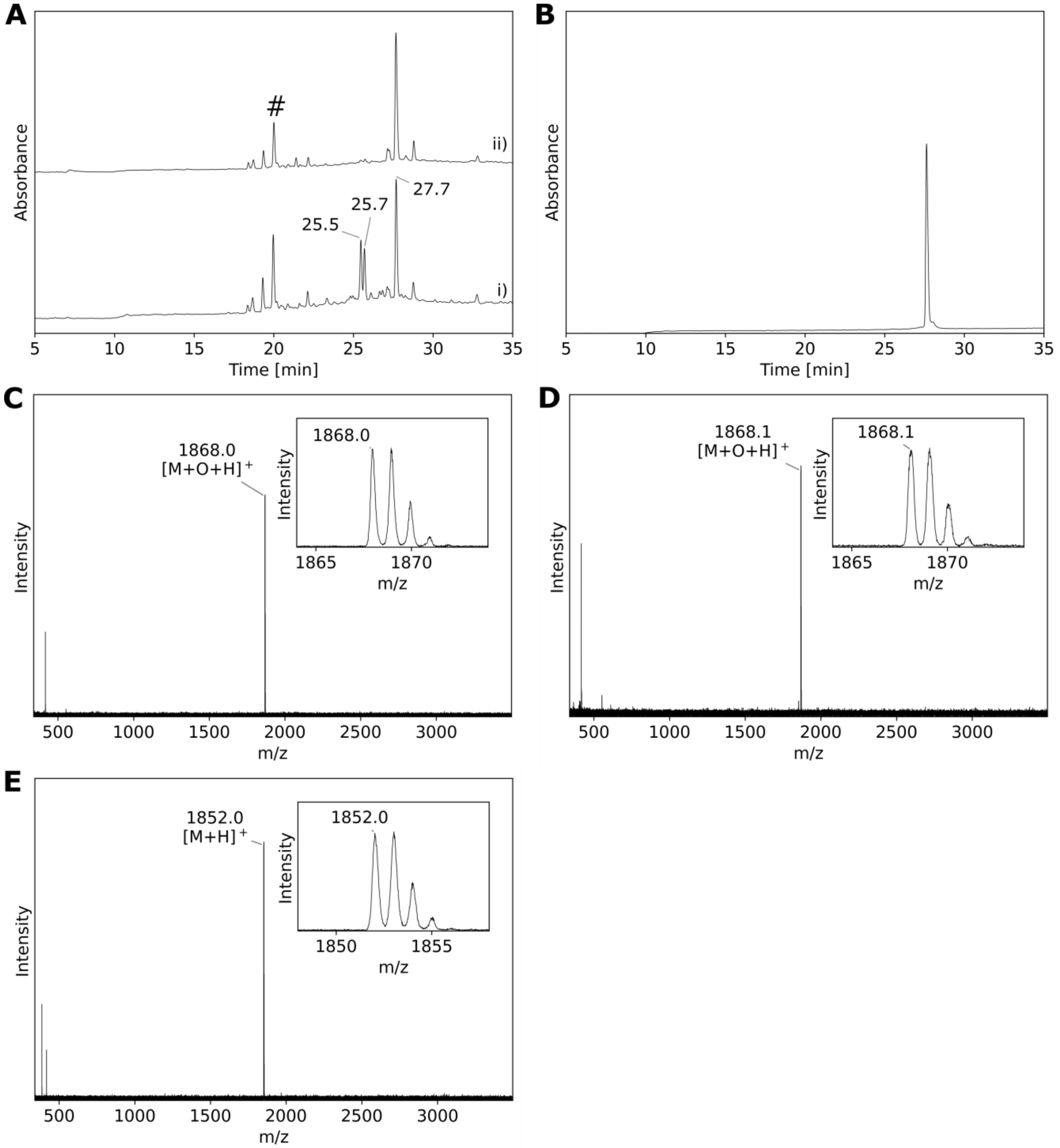

Figure S130: **P1A21Az12** A) Analytical HPLC (220 nm) of crude peptide (i) bevor reduction and (ii) after reduction with TMSBr and ethane-1,2-dithiol. HPLC Gradients are listed in Section 2.3.5 Table S3. # Peptide without homoserine. B) Analytical HPLC (220 nm) of pure peptide. C) MALDI-TOF MS of purified peptide with a retention time of 25.5 min. D) MALDI-TOF MS of purified peptide with a retention time of 25.7 min. E) MALDI-TOF MS of pure peptide. Calculated mass: $[M+H]^+$ 1852.0, : $[M+O+H]^+$ 1868.0.

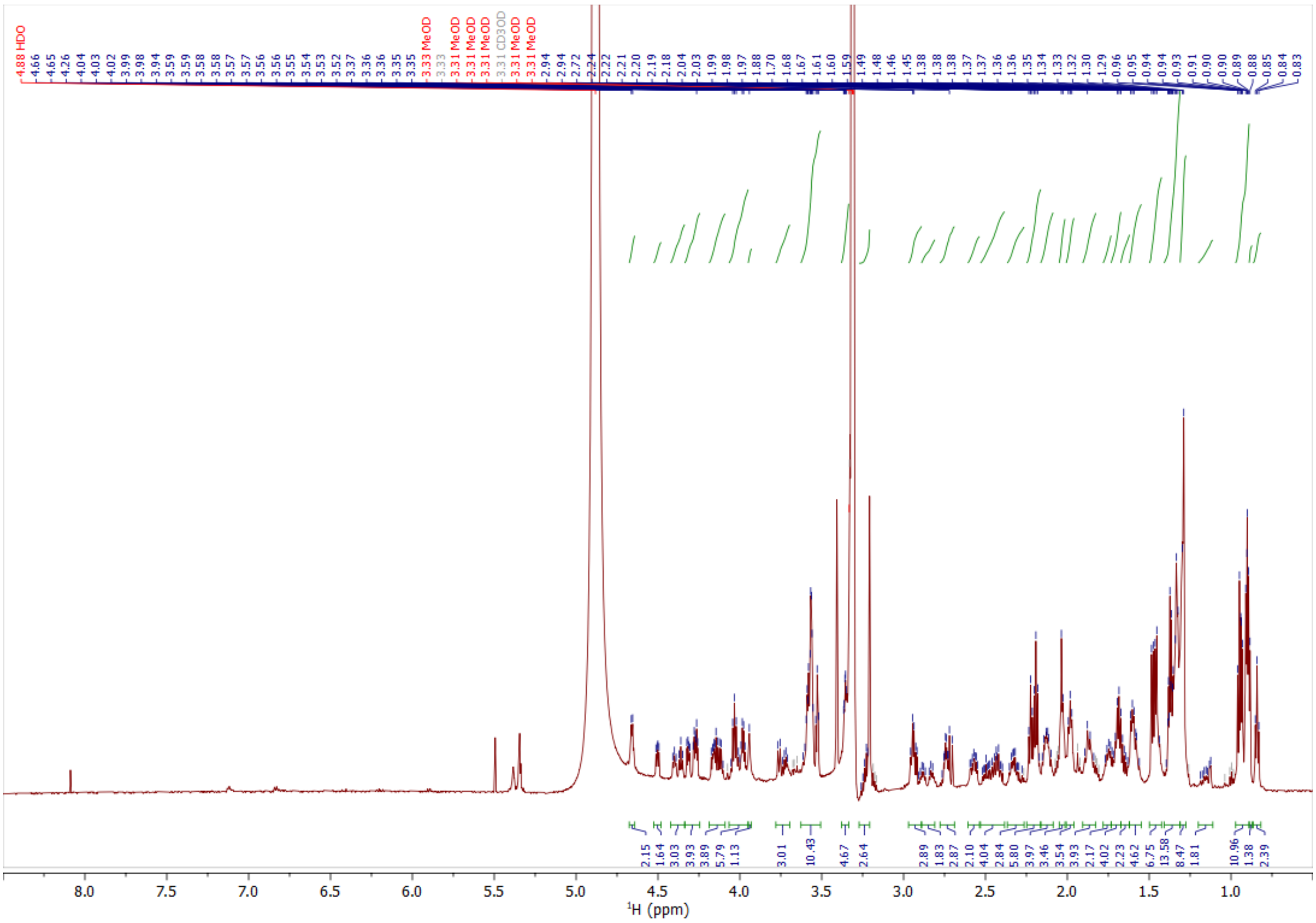

Figure S131: ^{1}H-NMR (700 MHz, MeOH-d_4, 295 K) of **P1A21Az12**. Hydrogen-deuterium exchange was performed prior to the measurement.

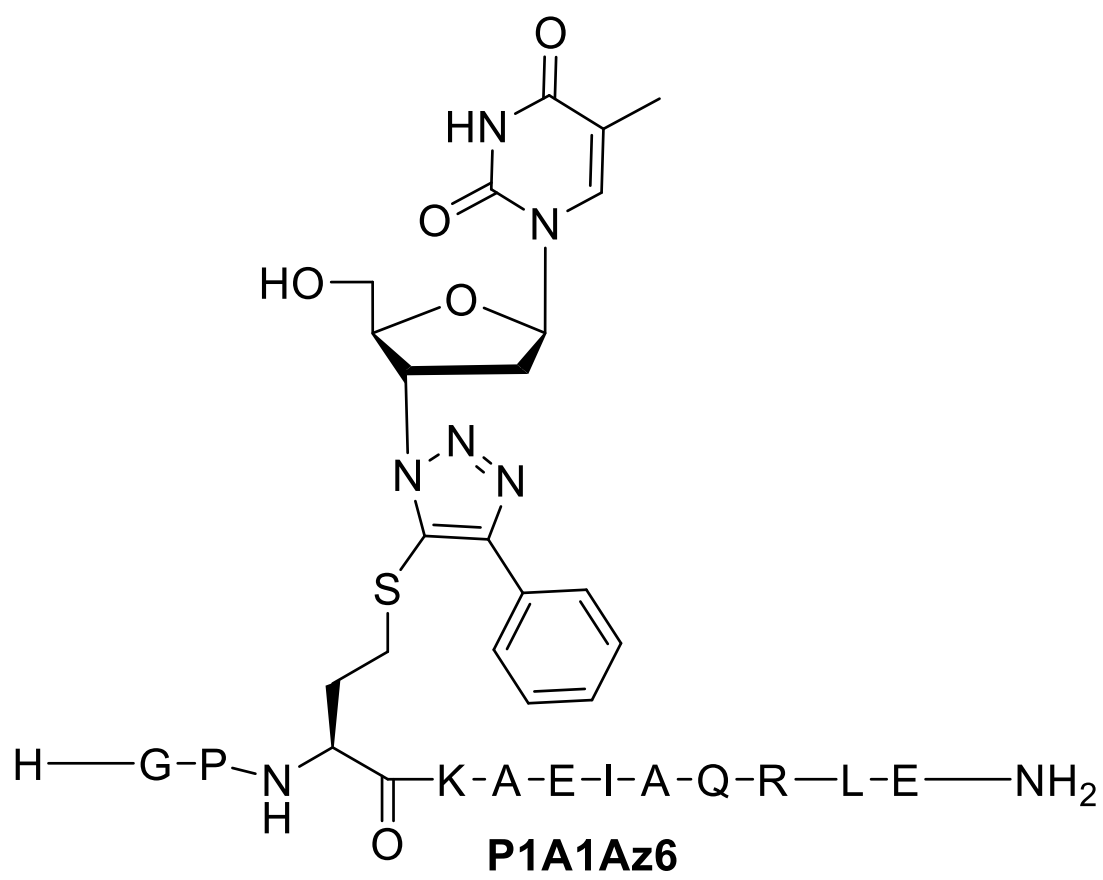

P1A1Az6

Chemical Formula: $C_{74}H_{115}N_{23}O_{21}S$

Exact Mass: 1693.84

Molecular Weight: 1694.93

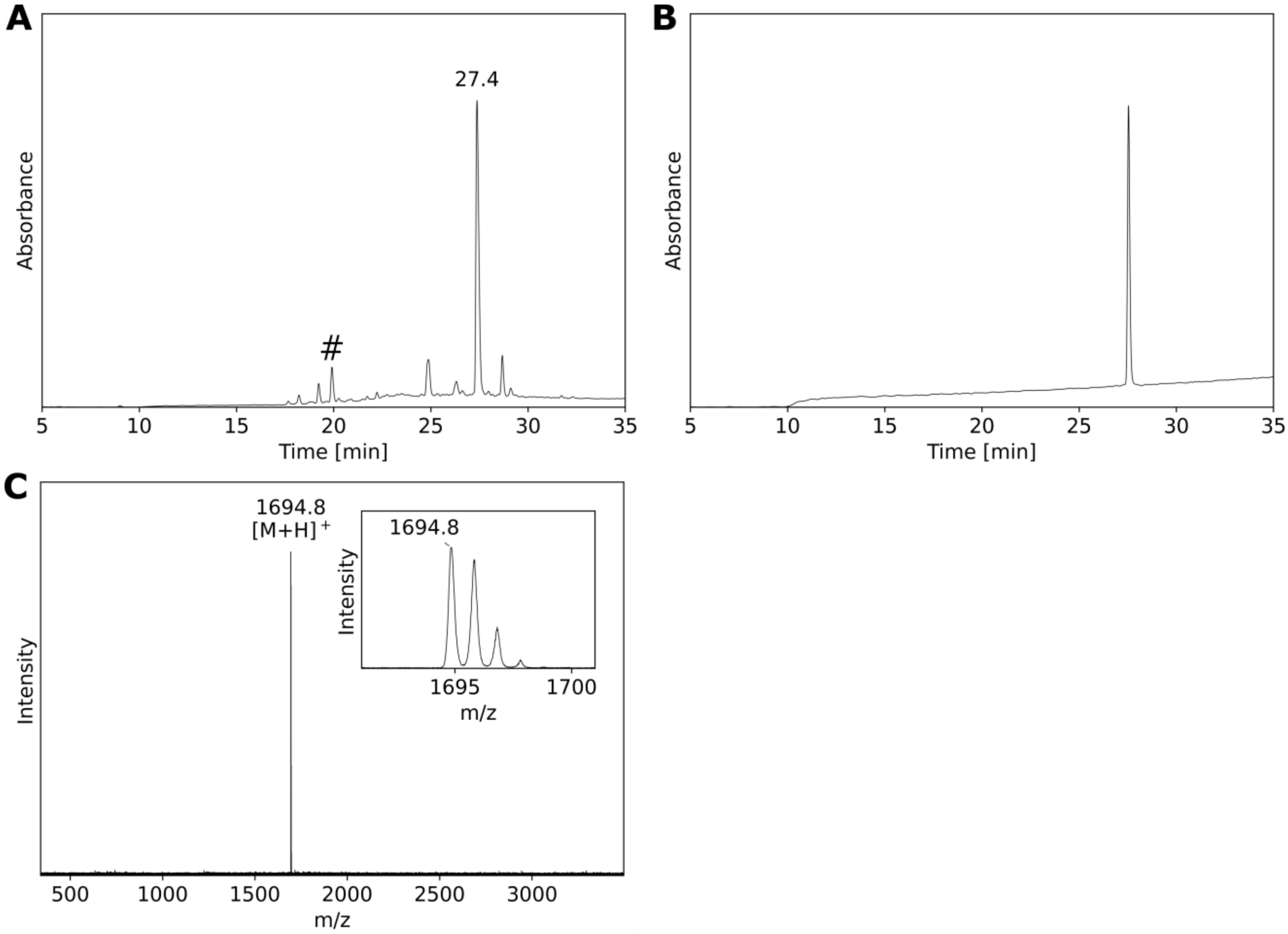

Figure S132: **P1A1Az6** A) Analytical HPLC (220 nm) of crude peptide. HPLC Gradients are listed in Section 2.3.5 Table S3. # Peptide without homoserine. B) Analytical HPLC (220 nm) of pure peptide. C) MALDI-TOF MS of pure peptide. Calculated mass: $[M+H]^+$ 1694.8.

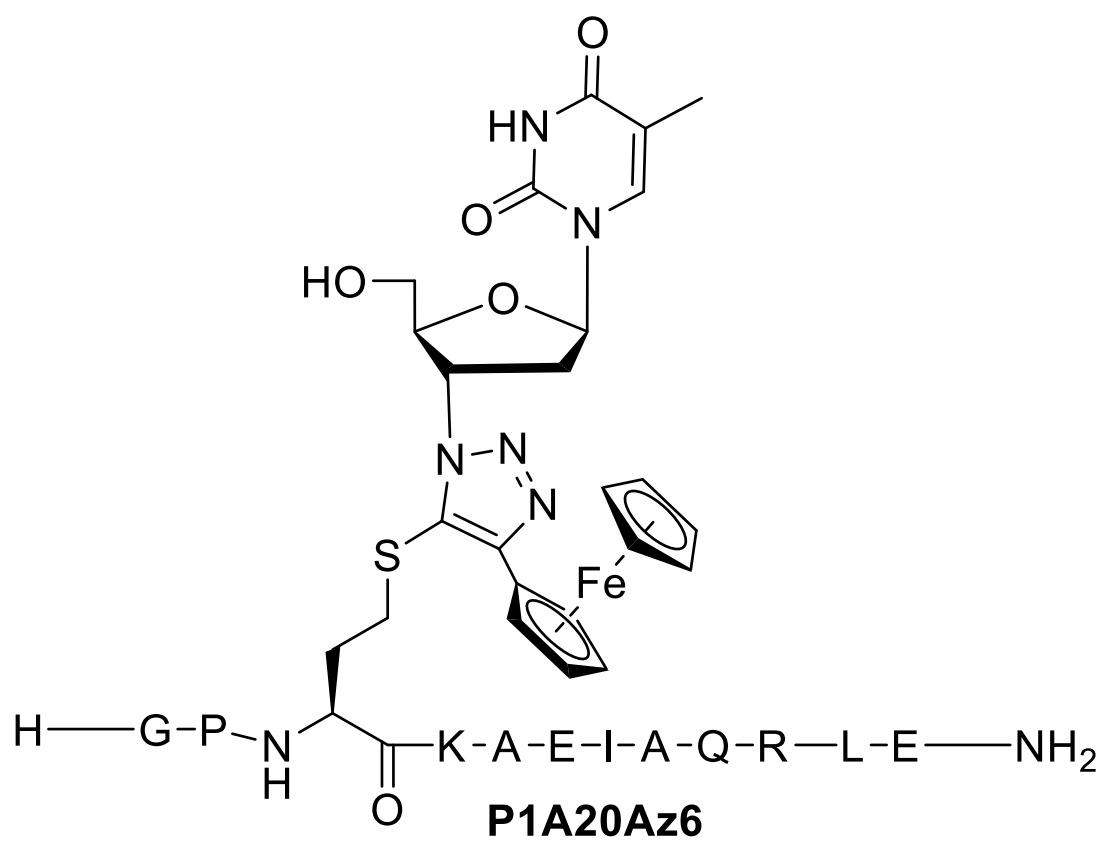

P1A20Az6

Chemical Formula: $C_{78}H_{119}FeN_{23}O_{21}S$

Exact Mass: 1801.80

Molecular Weight: 1802.86

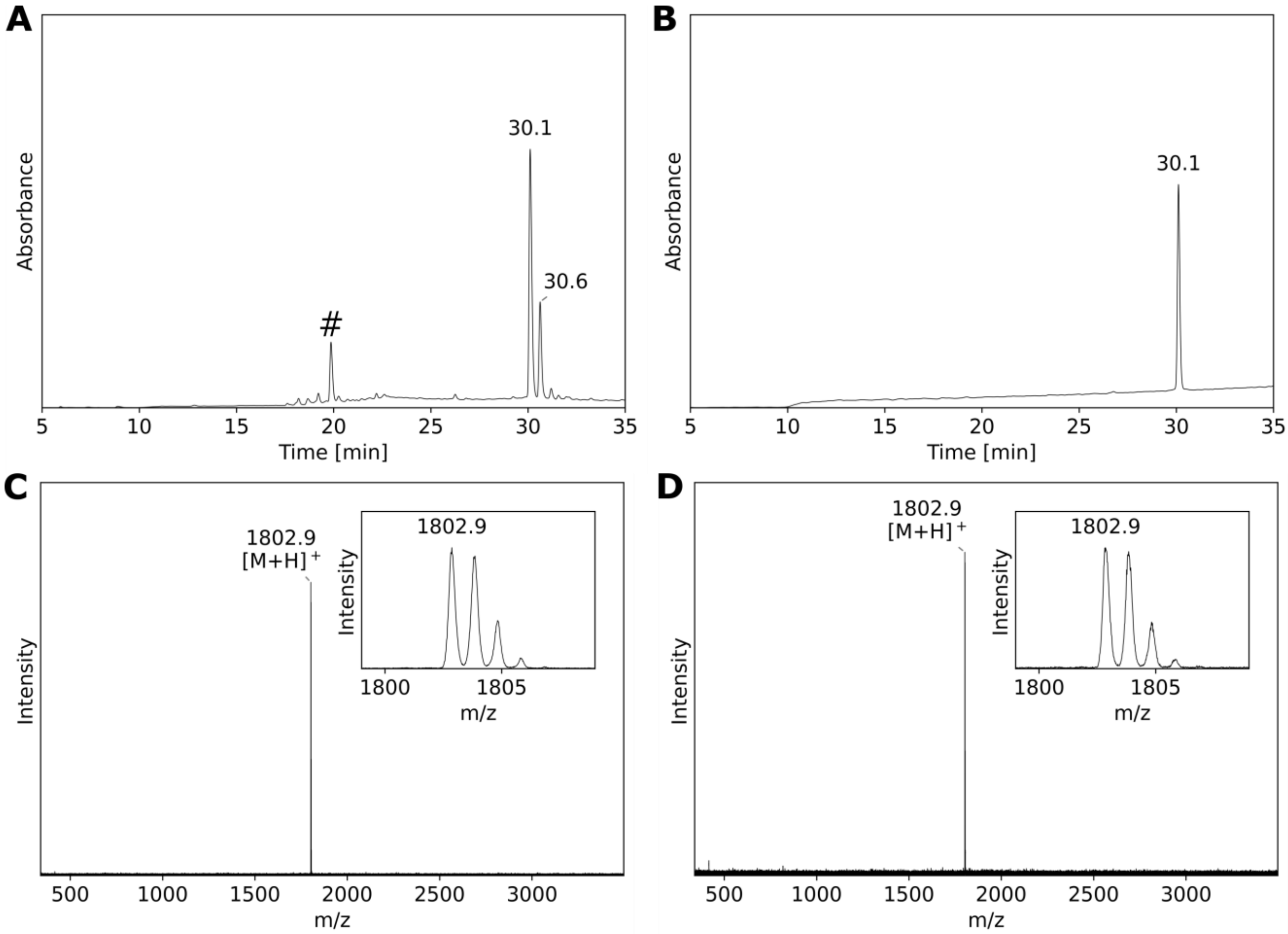

Figure S133: **P1A20Az6** A) Analytical HPLC (220 nm) of crude peptide. HPLC Gradients are listed in Section 2.3.5 Table S3. # Peptide without homoserine. B) Analytical HPLC (220 nm) of purified peptide with a retention time of 30.1 min. C) MALDI-TOF MS of purified peptide with a retention time of 30.1 min. D) MALDI-TOF MS of purified peptide with a retention time of 30.6 min. Calculated mass: $[M+H]^+$ 1802.8.

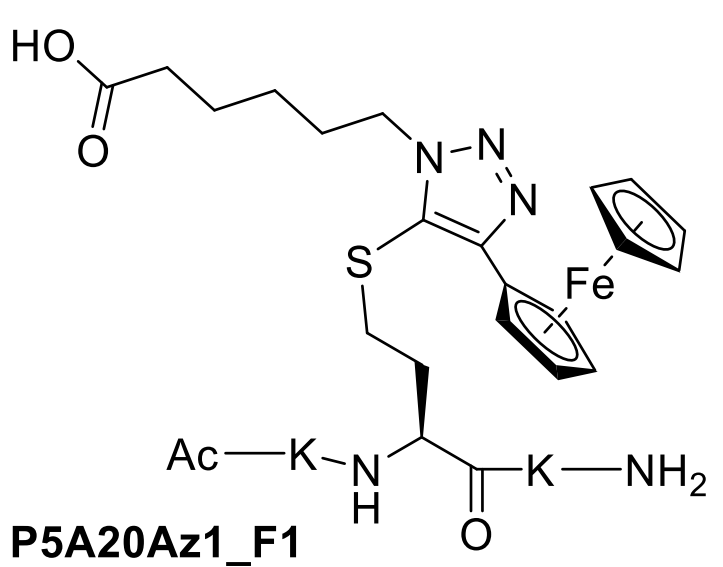

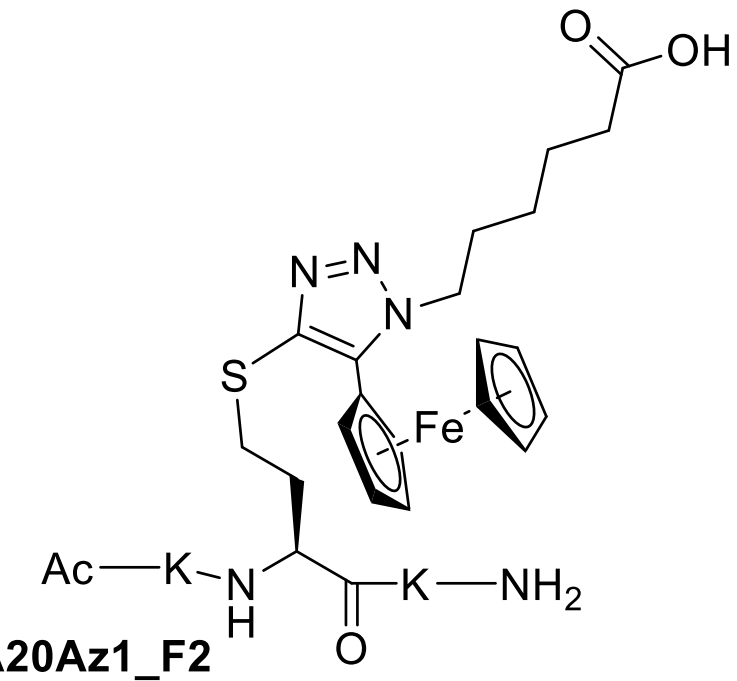

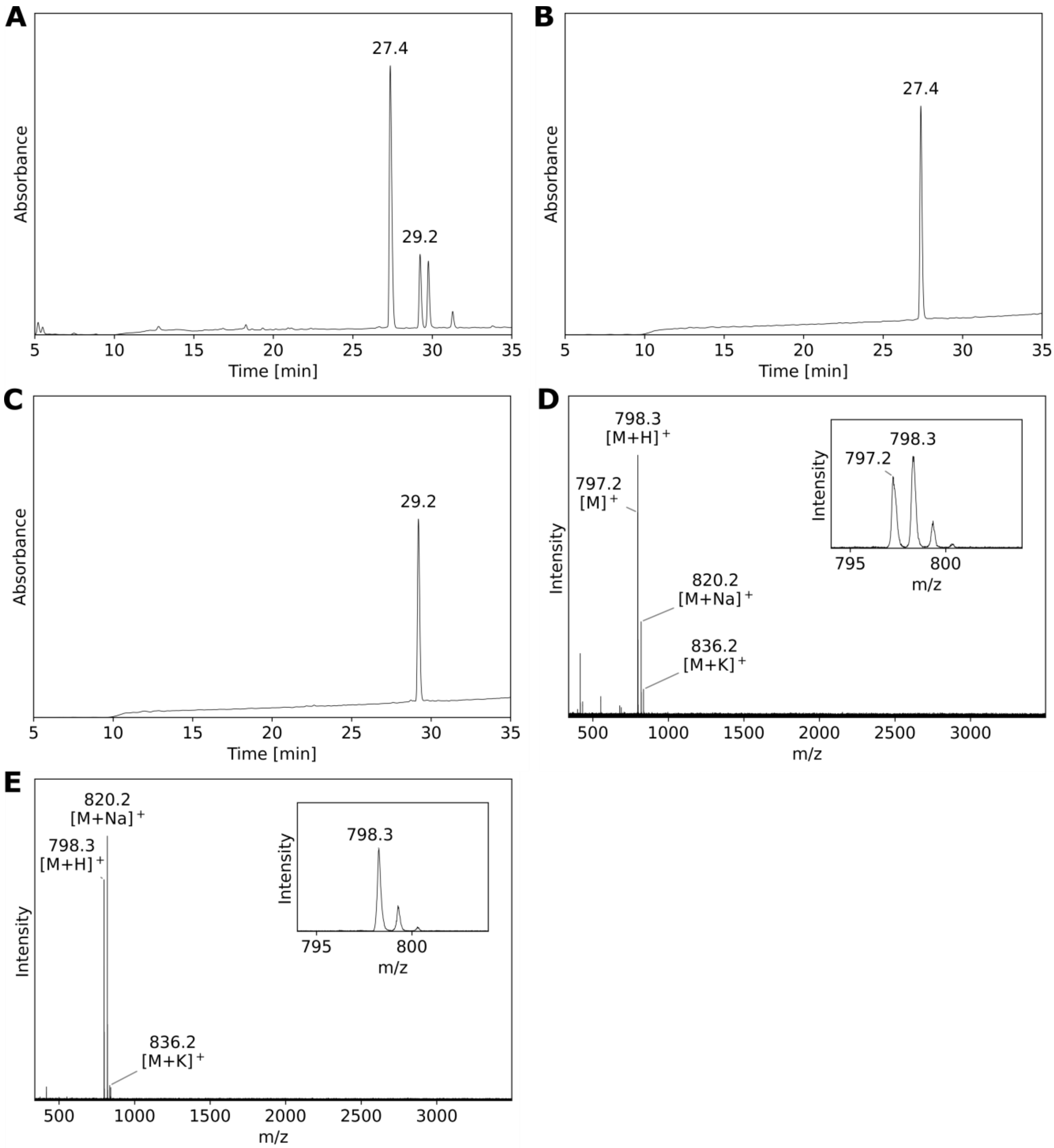

Figure S134: **P5A20Az1** A) Analytical HPLC (220 nm) of crude peptide. HPLC Gradients are listed in Section 2.3.5 Table S3. B) Analytical HPLC (220 nm) of purified peptide with a retention time of 27.4 min (**P5A20Az1_F1**). C) Analytical HPLC (220 nm) of purified peptide with a retention time of 29.2 min (**P5A20Az1_F2**). D) MALDI-TOF MS of purified peptide with a retention time of 27.4 min (**P5A20Az1_F1**). E)

MALDI-TOF MS of purified peptide with a retention time of 29.2 min (**P5A20Az1_F2**). Calculated mass: $[M]^+$ 797.3, $[M+H]^+$ 798.3, $[M+Na]^+$ 820.3, $[M+H]^+$ 836.3.

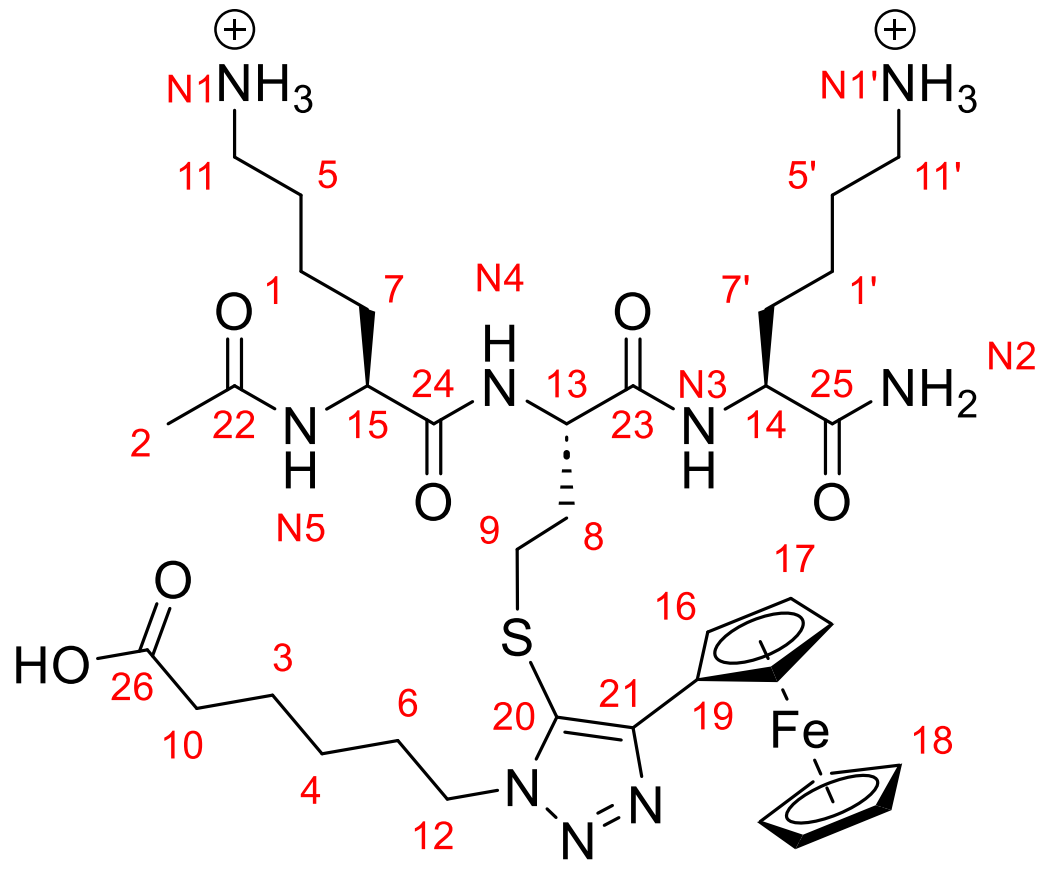

^{1}H NMR (700 MHz, DMSO-d_6, 295 K):

δ [ppm] = 8.08 (d, *J* = 7.8 Hz, 1H, H-N4), 8.06 (d, *J* = 7.8 Hz, 1H, H-N5), 7.81 (d, *J* = 8.0 Hz, 2H, H-N3), 7.73-7.72 (m, 6H, H-N1), 7.35 (s, 1H, H-N2), 7.05 (s, 1H, H-N2), 5.00-5.01 (m, 2H, H-16), 4.41-4.37 (m, 4H, H-12, H-17), 4.33-4.30 (m, 1H, H-13), 4.15-4.10 (m, 2H, H-14/H15), 4.04 (s, 5H, H-18), 2.87-2.79 (m, 2H, H-9), 2.78-2.70 (m, 4H, H-11), 2.76-2.70 (m, 4H, H-11), 2.19 (t, *J* = 7.2 Hz 2H, H-10), 1.96-1.91 (m, 1H, H-8), 1.89-1.85 (m, 2H, H-6), 1.83 (s, 3H, H-2), 1.82-1.78 (m, 1H, H-8), 1.66-1.43 (m, 10H, H-7/5/3), 1.34-1.20 (m, 6H, H-4/1).

^{13}C NMR (176 MHz, DMSO-d_6, 295 K):

δ [ppm] = 174.4 (C-26), 173.3 (C-25), 172.1 (C-24), 170.4 (C-23), 169.7 (C-22), 148.5 (C-21), 122.6 (C-20), 75.0 (C-19), 69.3 (C-18), 68.7 (C-17), 66.6 (C-16), 52.8 (C-15), 52.2 (C-14), 51.7 (C-13), 47.5 (C-12), 38.72 (C-11), 38.68 (C-11), 33.5 (C-10), 32.1 (C-9), 31.4 (C-8), 31.4 (C-7), 31.1 (C-7), 29.3 (C-6), 26.7 (C-5), 25.5 (C-4), 24.0 (C-3), 22.5 (C-2), 22.1 (C-1).

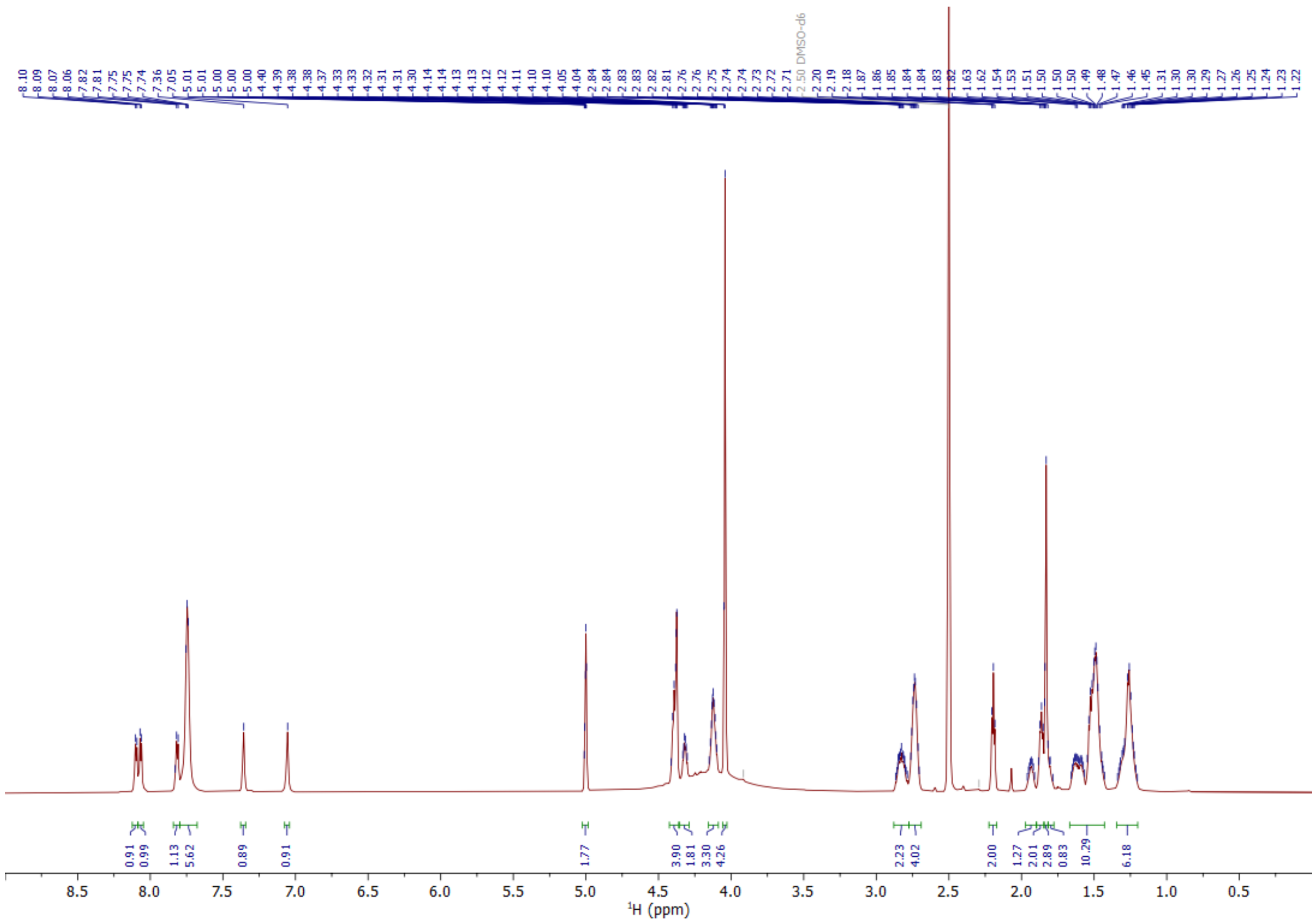

Figure S135: ^{1}H NMR spectrum (700 MHz, DMSO-d_6, 295 K) of **P5A20Az1_F1**.

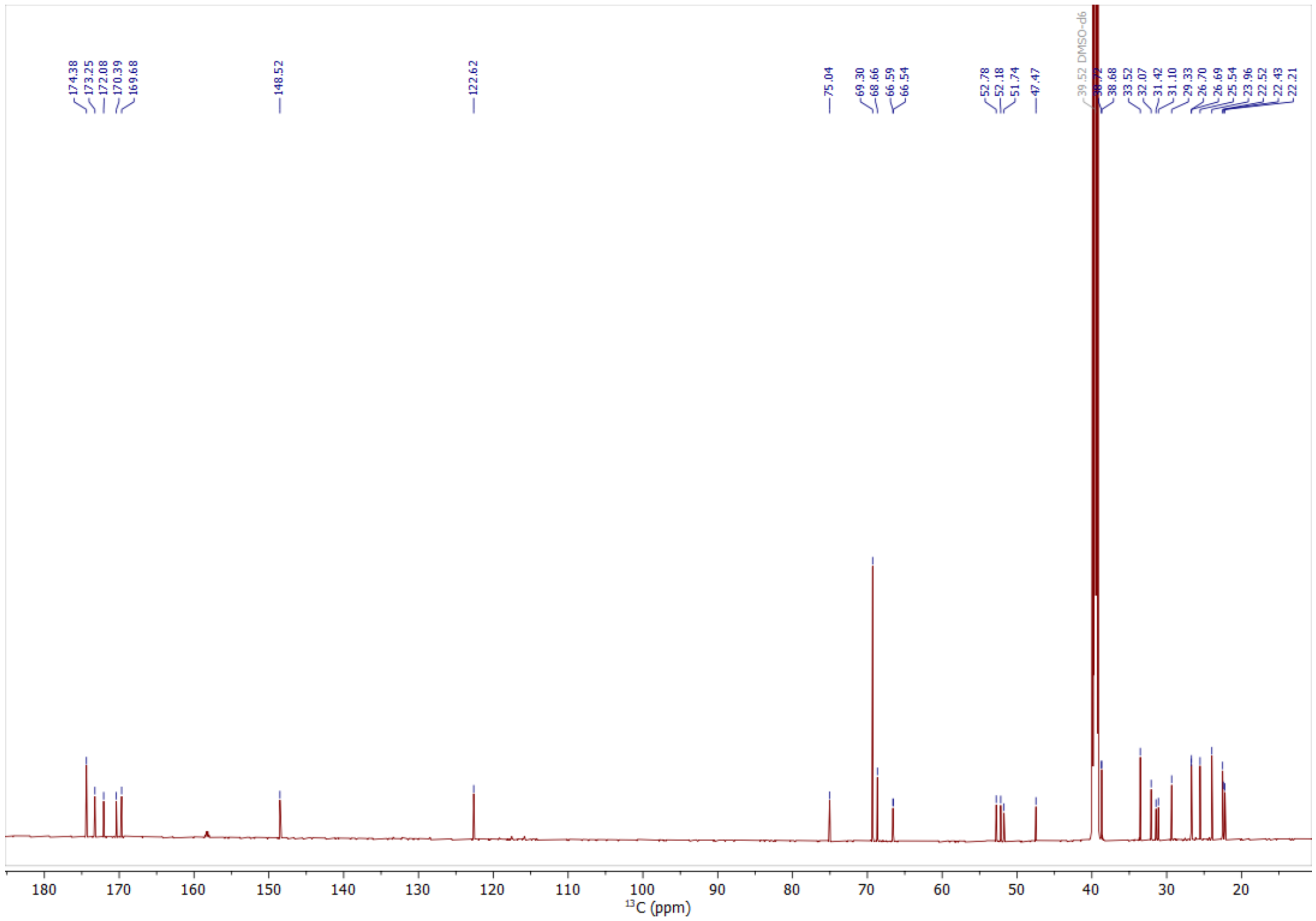

Figure S136: ^{13}C NMR spectrum (176 MHz, DMSO-d_6, 295 K) of **P5A20Az1_F1**.

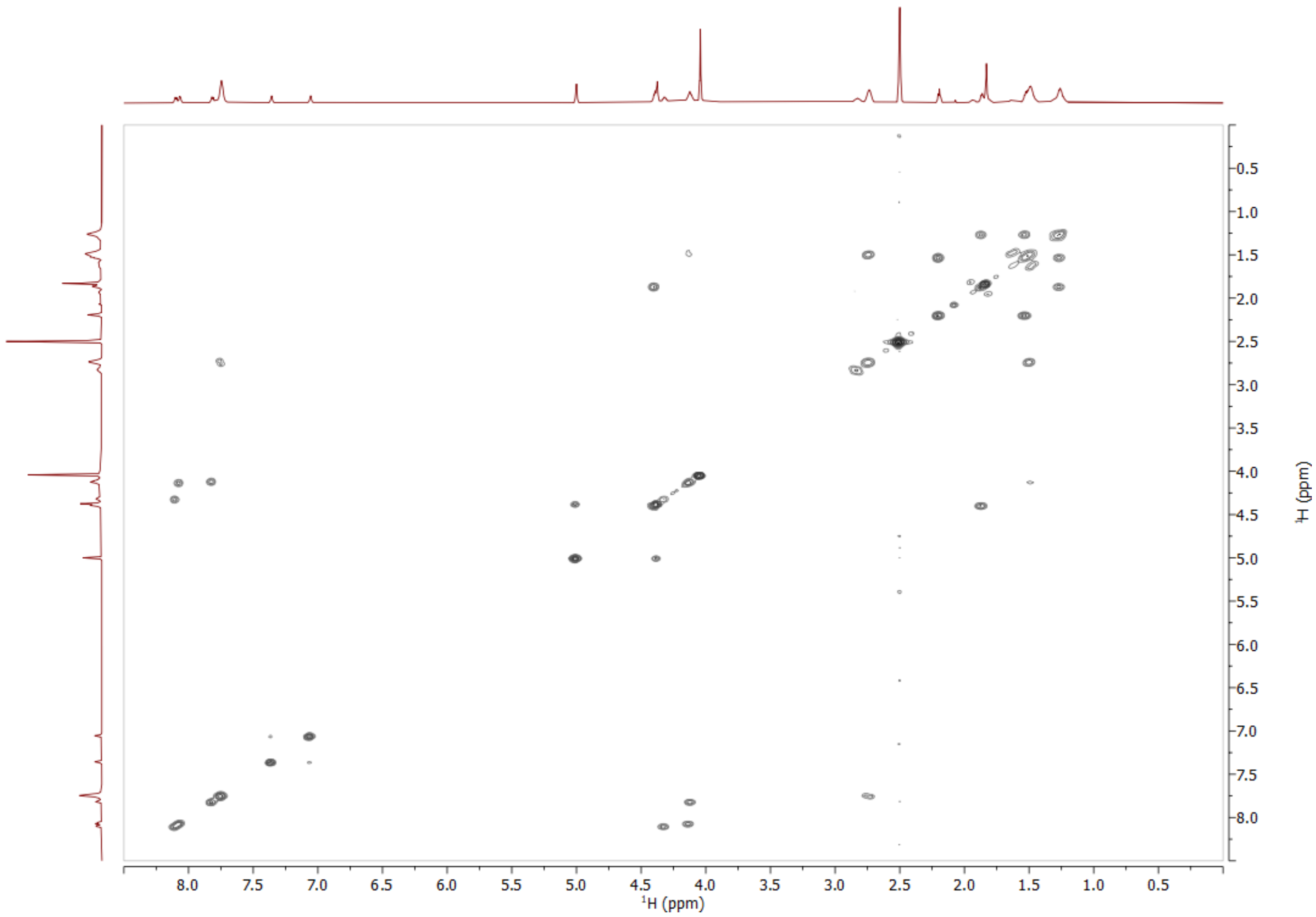

Figure S137: ^{1}H-^{1}H COSY spectrum (700 MHz, 700 MHz, DMSO-d_6, 295 K) of **P5A20Az1_F1**.

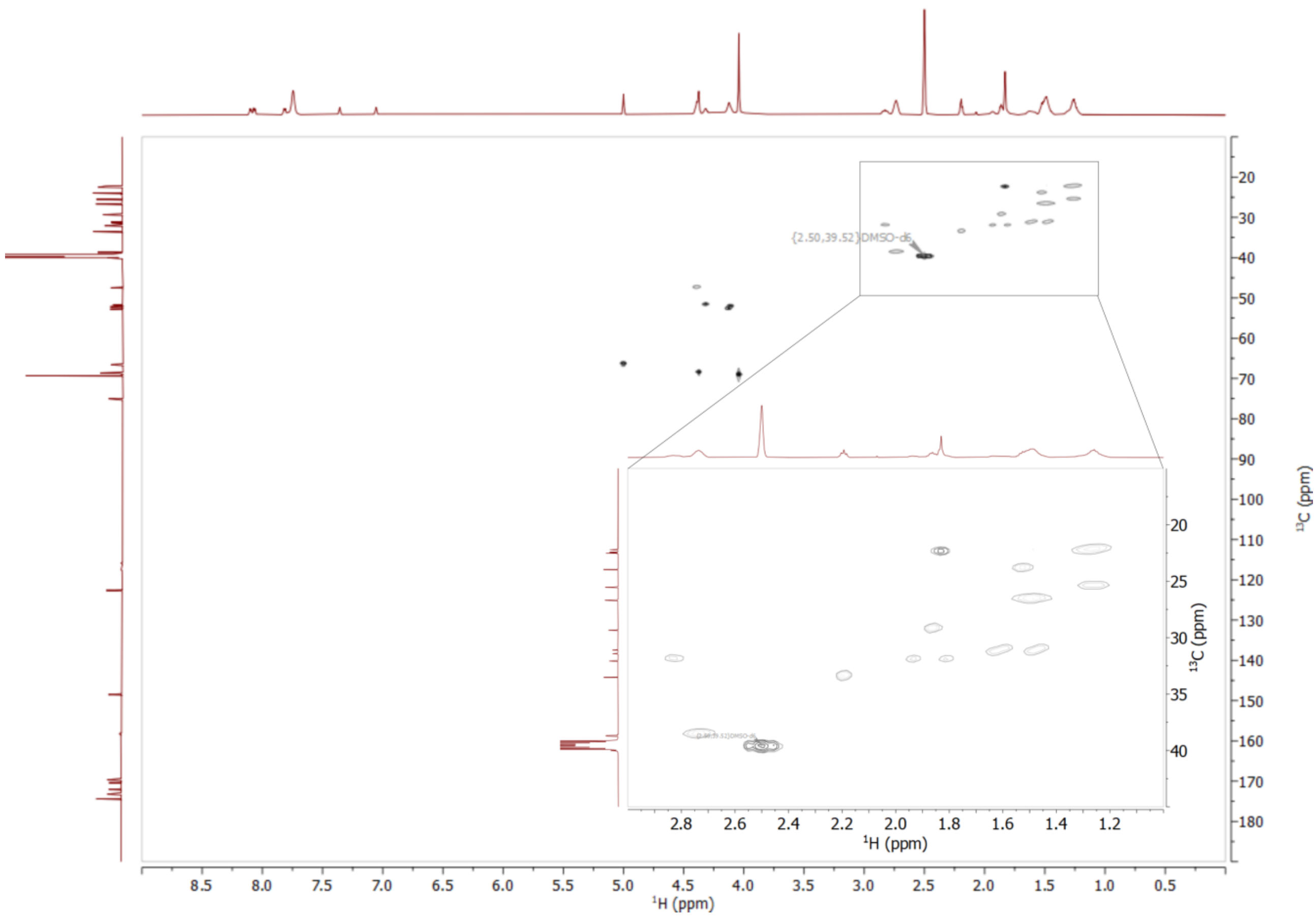

Figure S138: ^{1}H-^{13}C HSQC spectrum (700 MHz, 176 MHz, DMSO-d_6, 295 K) of **P5A20Az1_F1**.

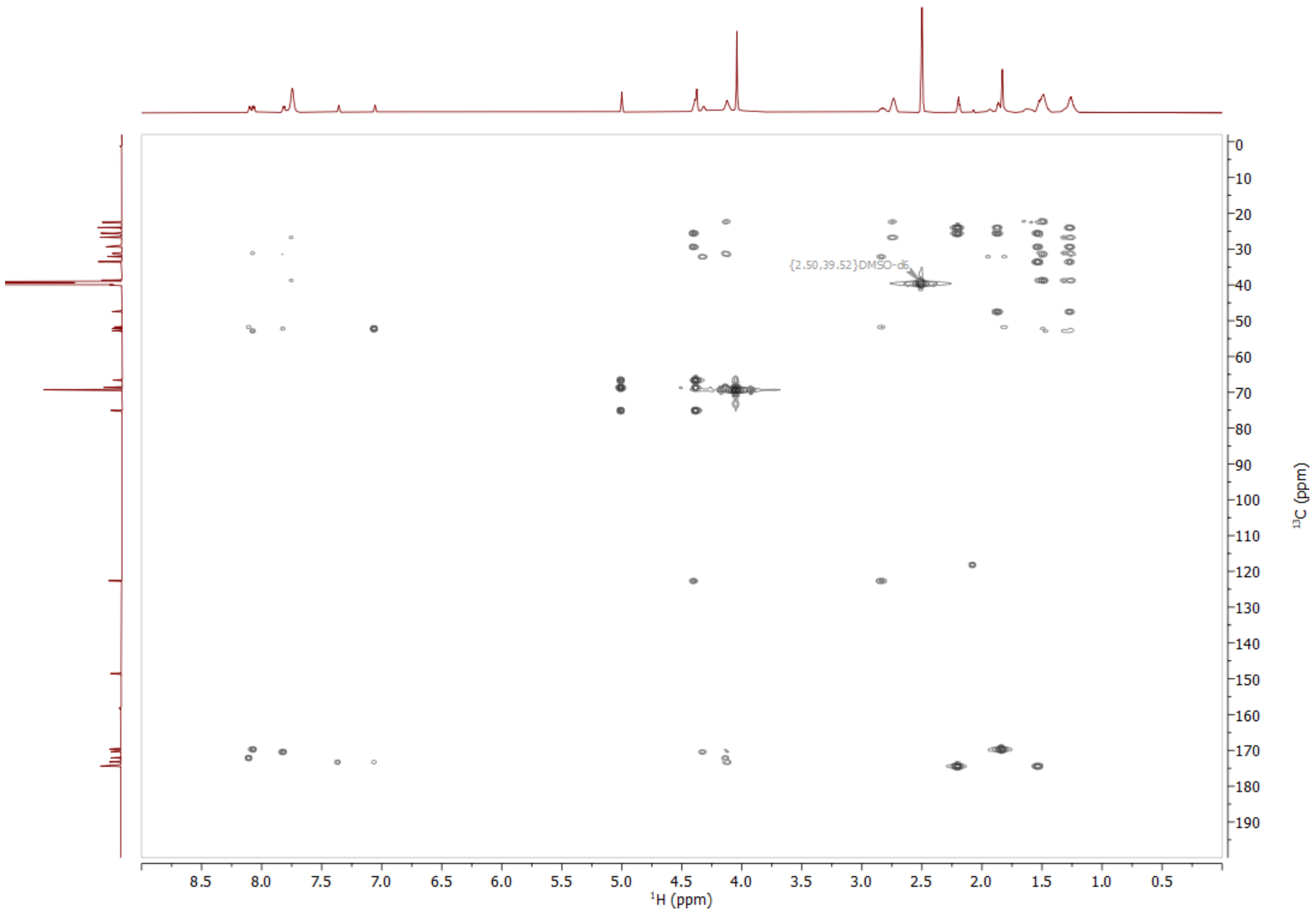

Figure S139: ^1H-^{13}C HMBC spectrum (700 MHz, 176 MHz, DMSO-d_6, 295 K) of **P5A20Az1_F1**.

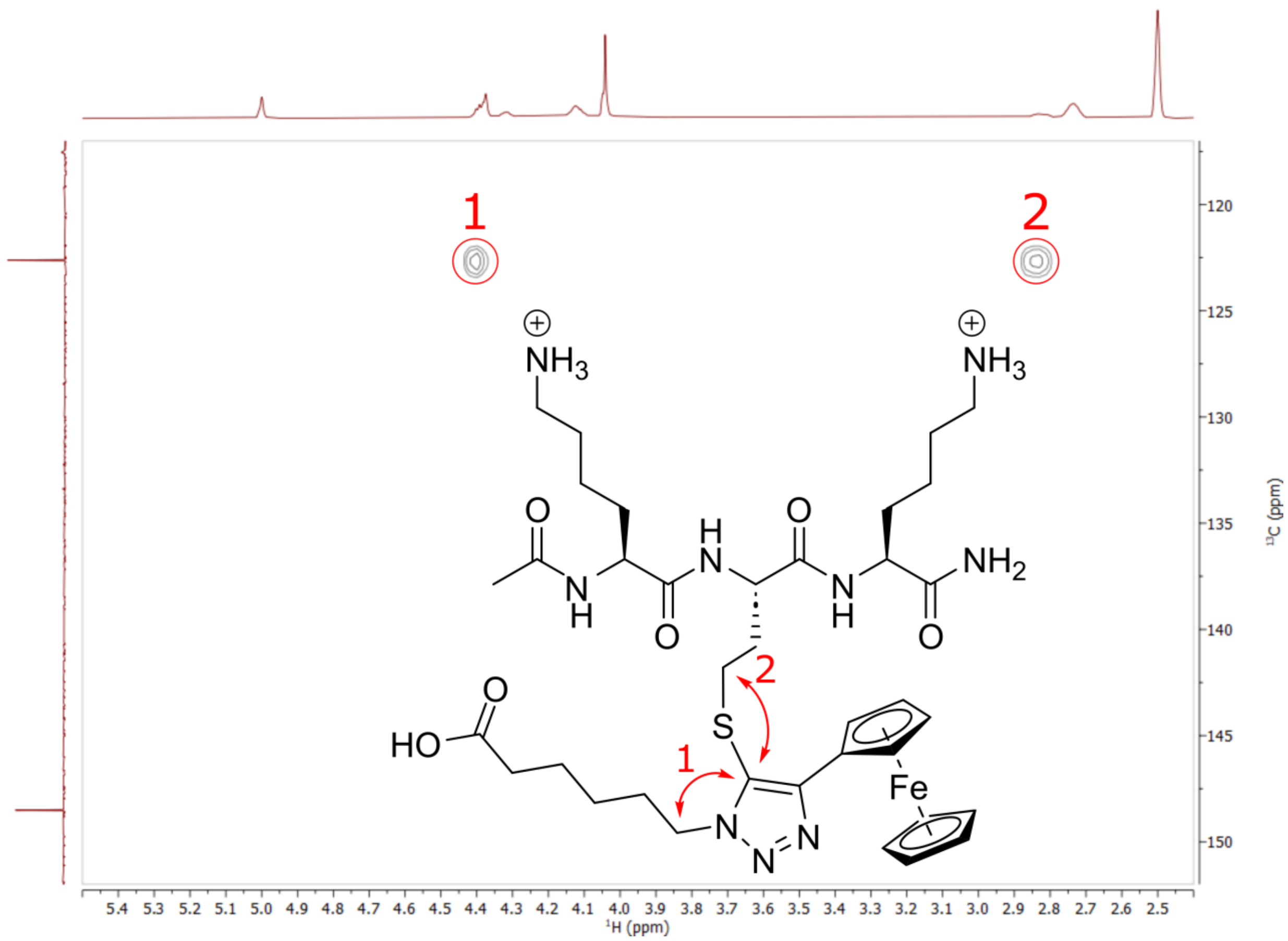

Figure S140: Extract from the ^1H-^{13}C HMBC spectrum (700 MHz, 176 MHz, DMSO-d_6, 295 K) of **P5A20Az1_F1** showing the cross-peaks relevant for isomer determination.

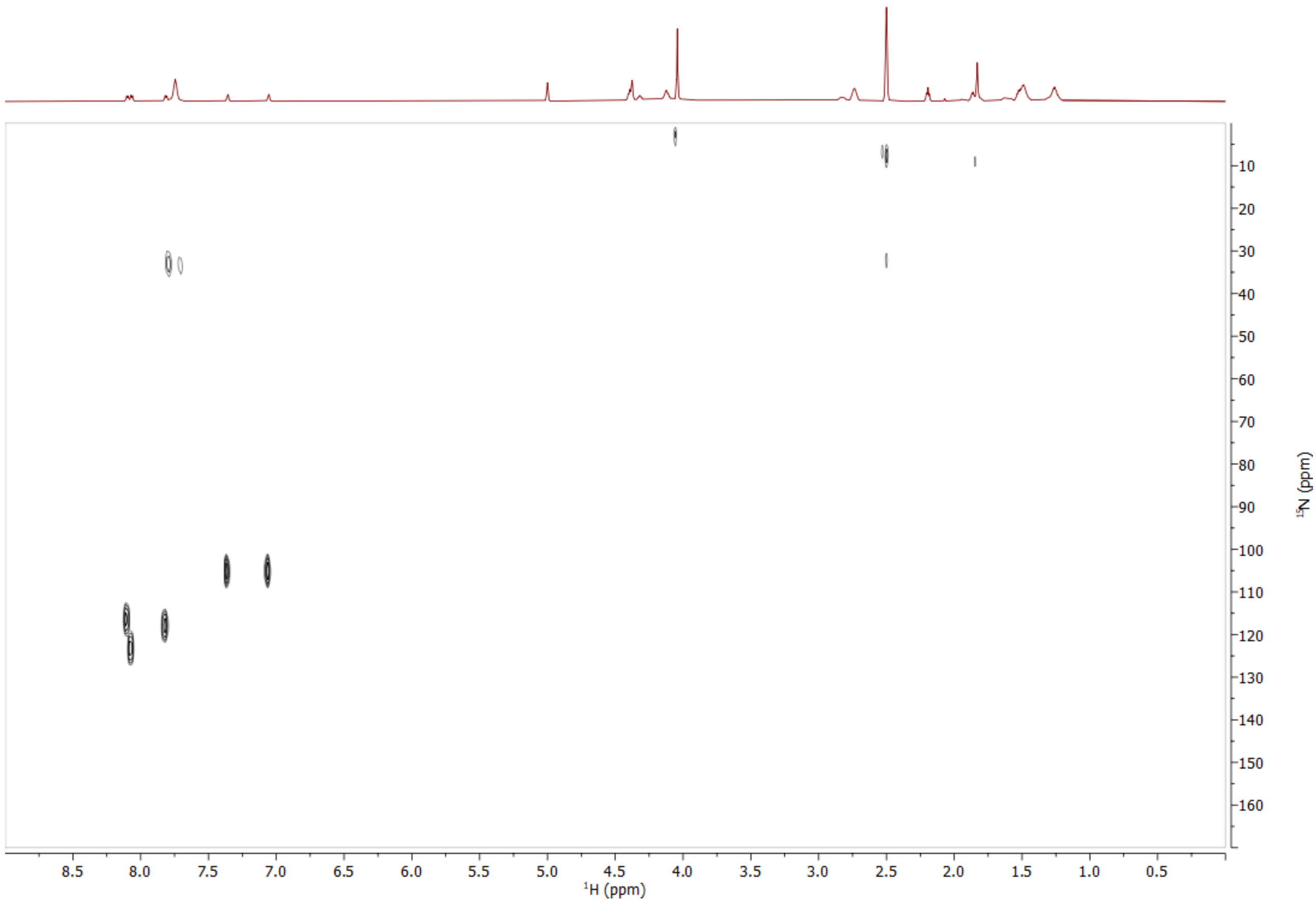

Figure S141: ^{1}H-^{15}N HSQC (700 MHz, 70.9 MHz, DMSO-d_6, 295 K) of **P5A20Az1_F1**.

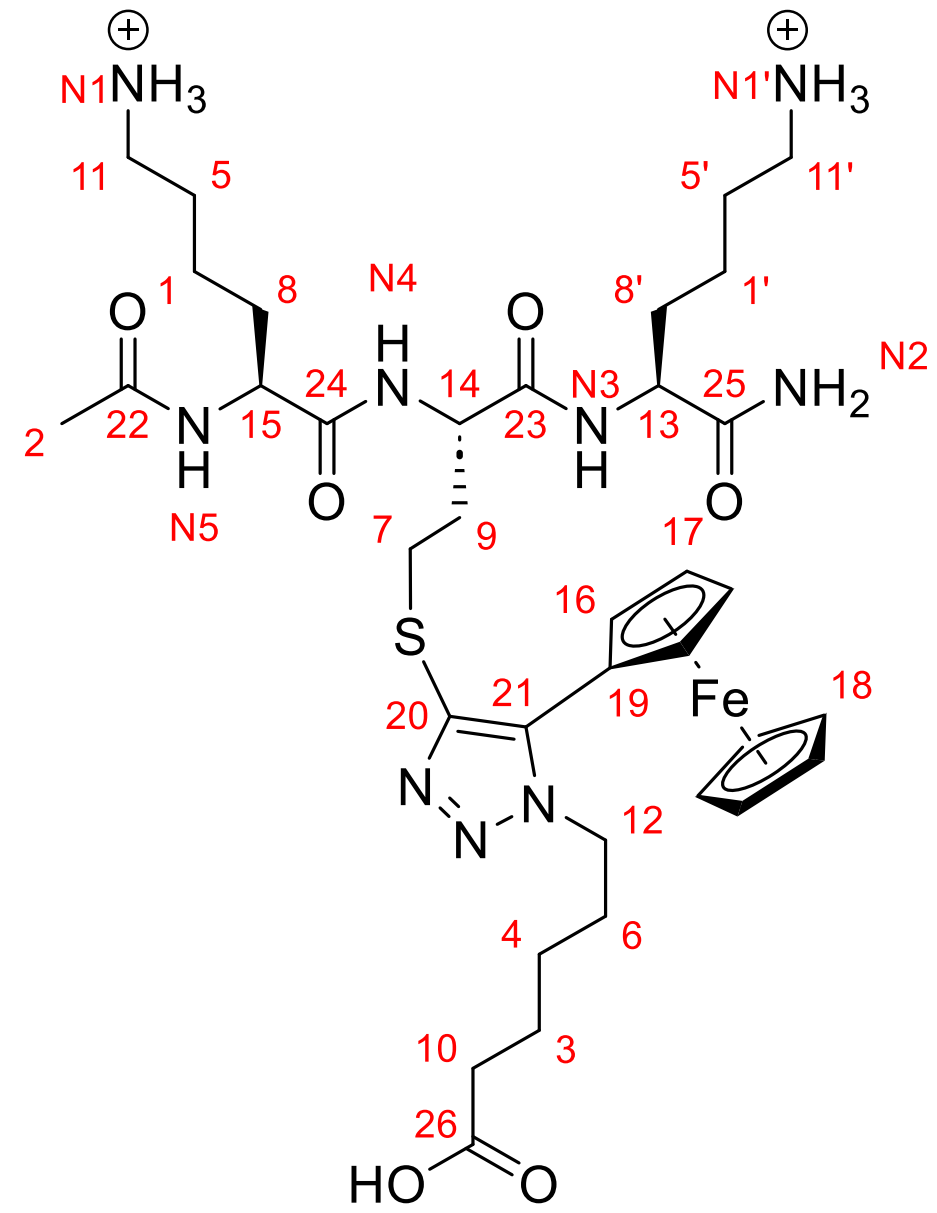

^{1}H NMR (700 MHz, DMSO-d_6, 295 K):

δ [ppm] = 8.09 (d, *J* = 7.6 Hz, 1H, H-N5), 8.08 (d, *J* = 7.6 Hz, 1H, H-N4), 7.82 (d, *J* = 7.9 Hz, 2H, H-N3), 7.70-7.67 (m, 6H, H-N1), 7.32 (s, 1H, H-N2), 7.06 (s, 1H, H-N2), 4.79-4.77 (m, 2H, H-16), 4.53-4.51 (m, 4H, H-17/12), 4.35-4.32 (m, 1H, H-14), 4.22 (s, 5H, H-18), 4.18-4.11 (m, 2H, H-13/H15), 3.05-2.95 (m, 2H, H-7), 2.78-2.73 (m, 4H, H-11), 2.22 (t, *J* = 7.3 Hz 2H, H-10), 2.08-1.90 (m, 2H, H-9), 1.85 (s, 3H, H-2), 1.84-1.82 (m, 2H, H-6), 1.68-1.62 (m, 2H, H-8), 1.57-1.47 (m, 10H, H-8/5/3), 1.38-1.23 (m, 6H, H-4/1).

^{13}C NMR (176 MHz, DMSO-d_6, 295 K):

δ [ppm] = 174.4 (C-26), 173.3 (C-25), 172.0 (C-24), 170.7 (C-23), 169.8 (C-22), 136.0 (C-21), 133.9 (C-20), 70.2 (C-19), 69.5 (C-18), 69.4 (C-17), 67.98 (C-16), 67.97 (C-16), 52.8 (C-15), 52.2 (C-13/C-14), 52.1 (C-13/C-14), 48.7 (C-12), 38.8 (C-11), 38.7 (C-11), 33.5 (C-10), 32.2 (C-9), 31.4 (C-8), 31.1 (C-8), 29.9 (C-7), 29.2 (C-6), 26.7 (C-5), 25.7 (C-4), 24.0 (C-3), 22.4 (C-2), 22.2 (C-1).

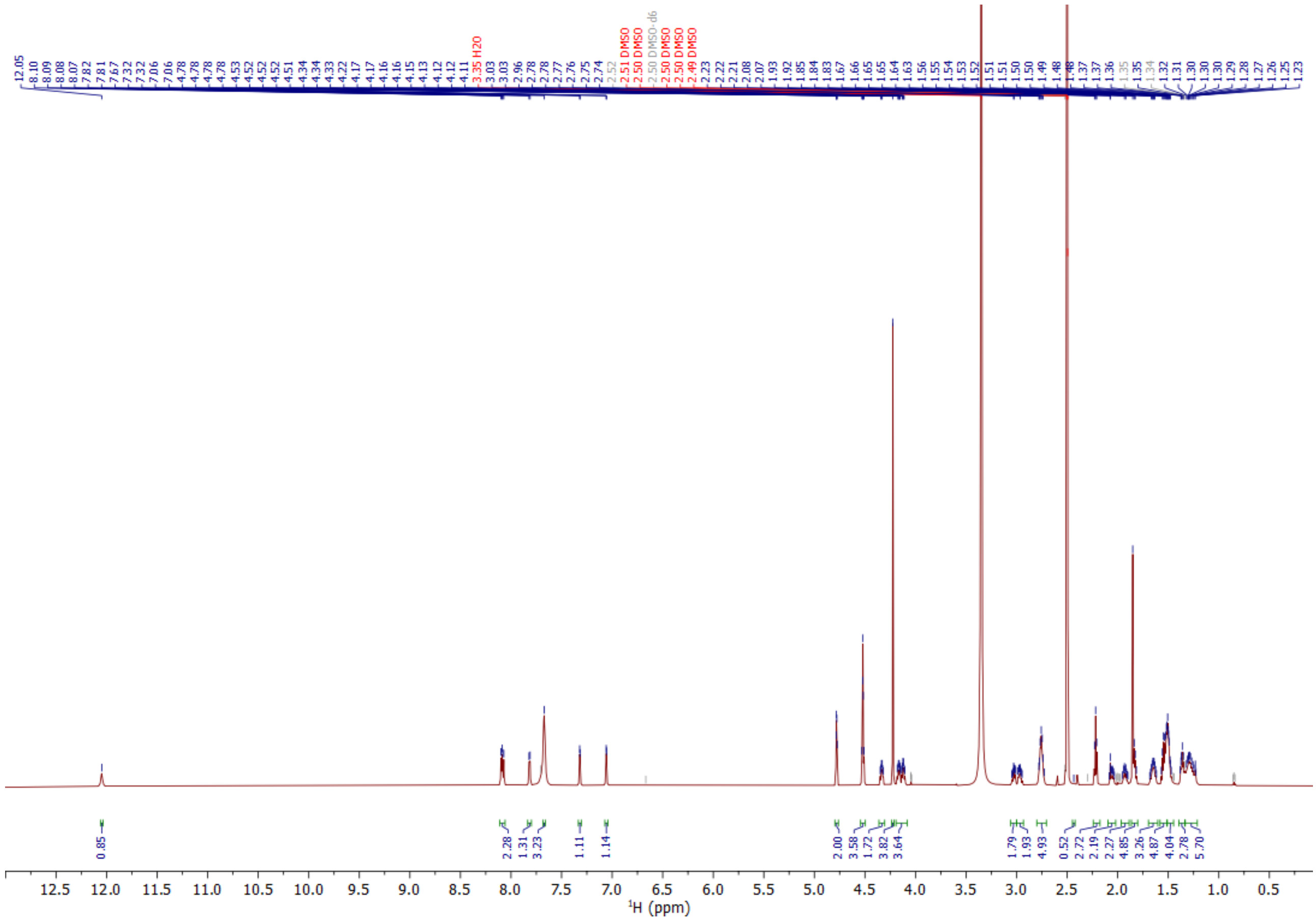

Figure S142: ^{1}H NMR spectrum (700 MHz, DMSO-d_6, 295 K) of **P5A20Az1_F2**.

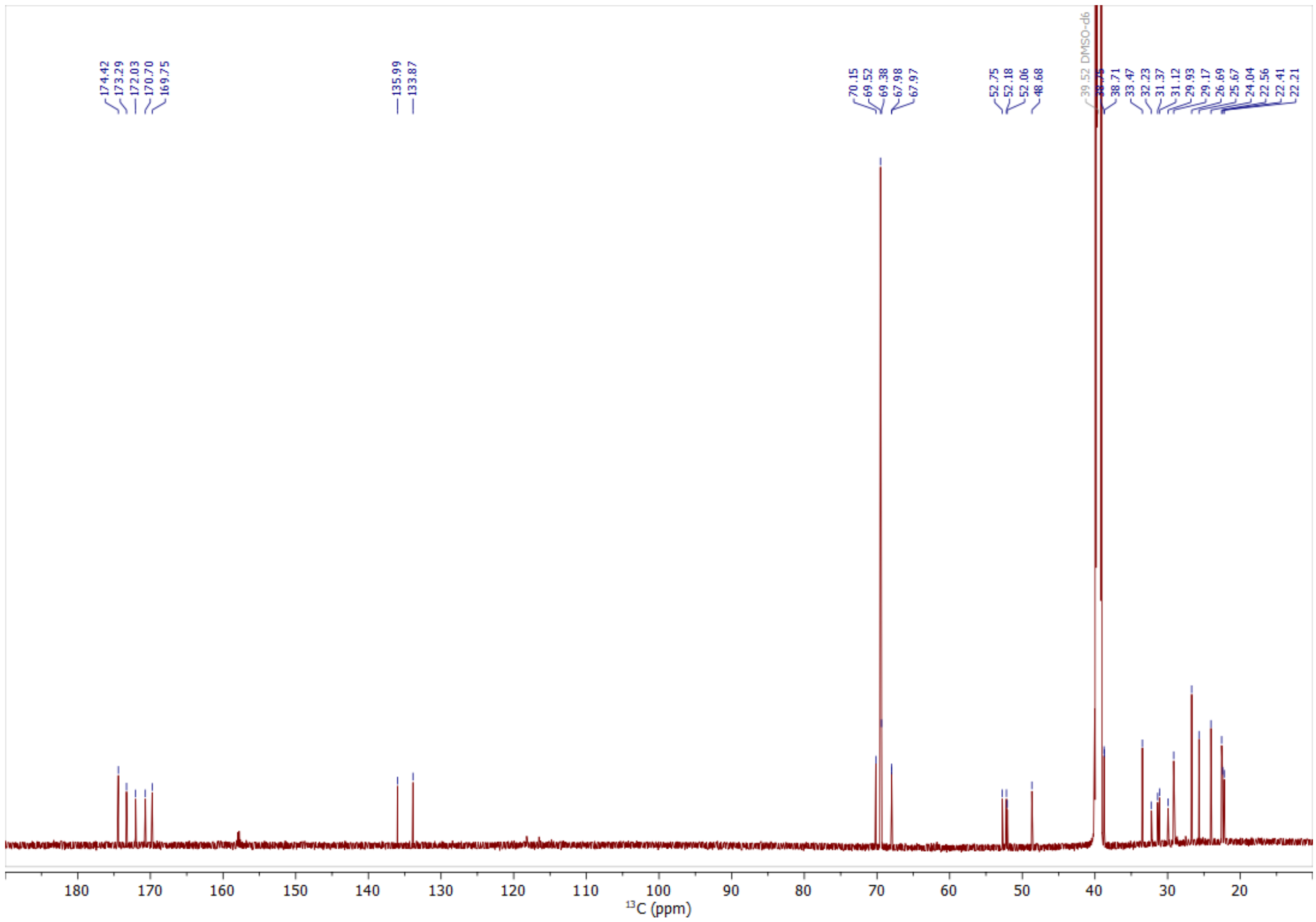

Figure S143: ^{13}C NMR spectrum (176 MHz, DMSO-d_6, 295 K) of **P5A20Az1_F2**.

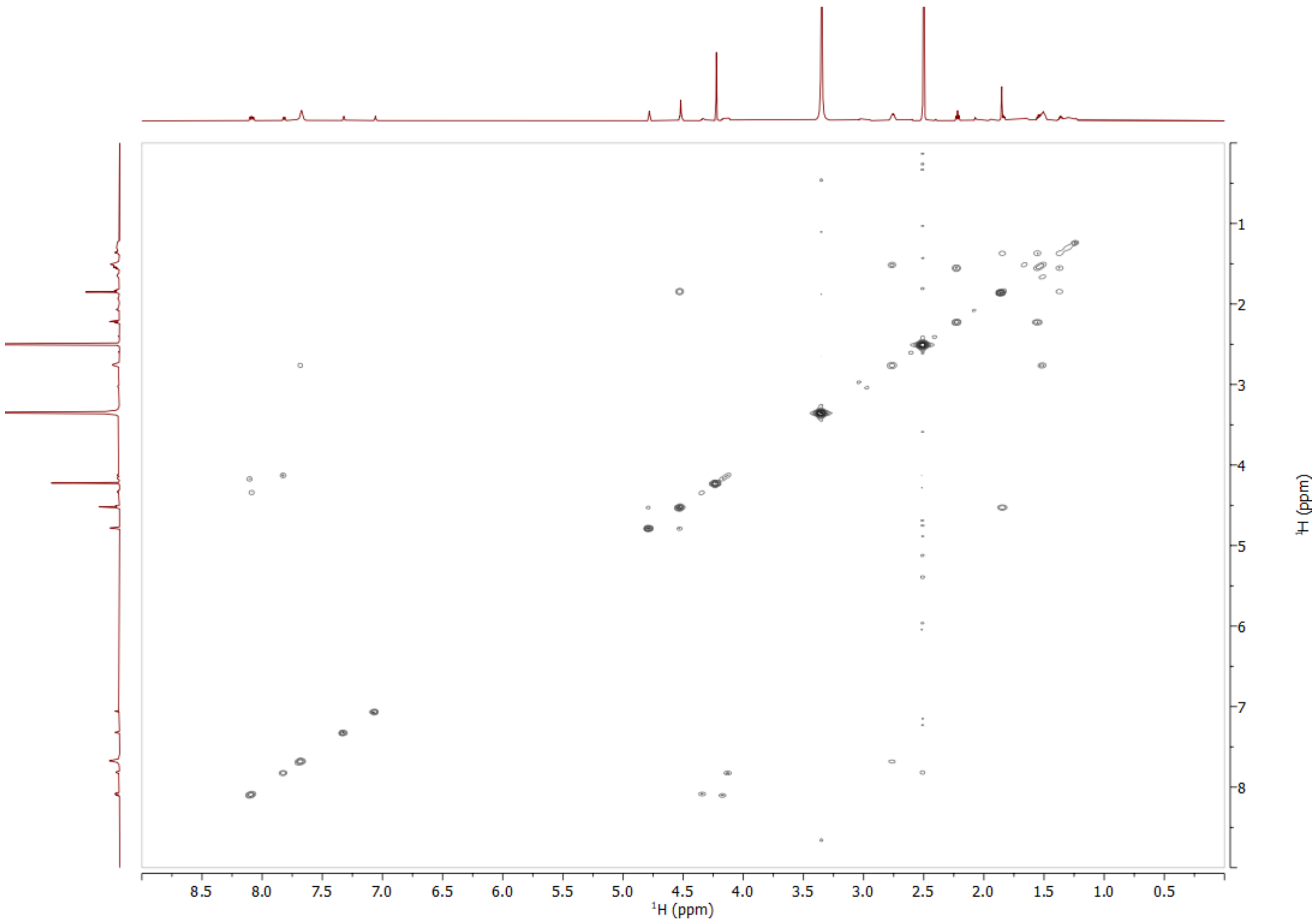

Figure S144: ^{1}H-^{1}H COSY spectrum (700 MHz, 700 MHz, DMSO-d_6, 295 K) of **P5A20Az1_F2**.

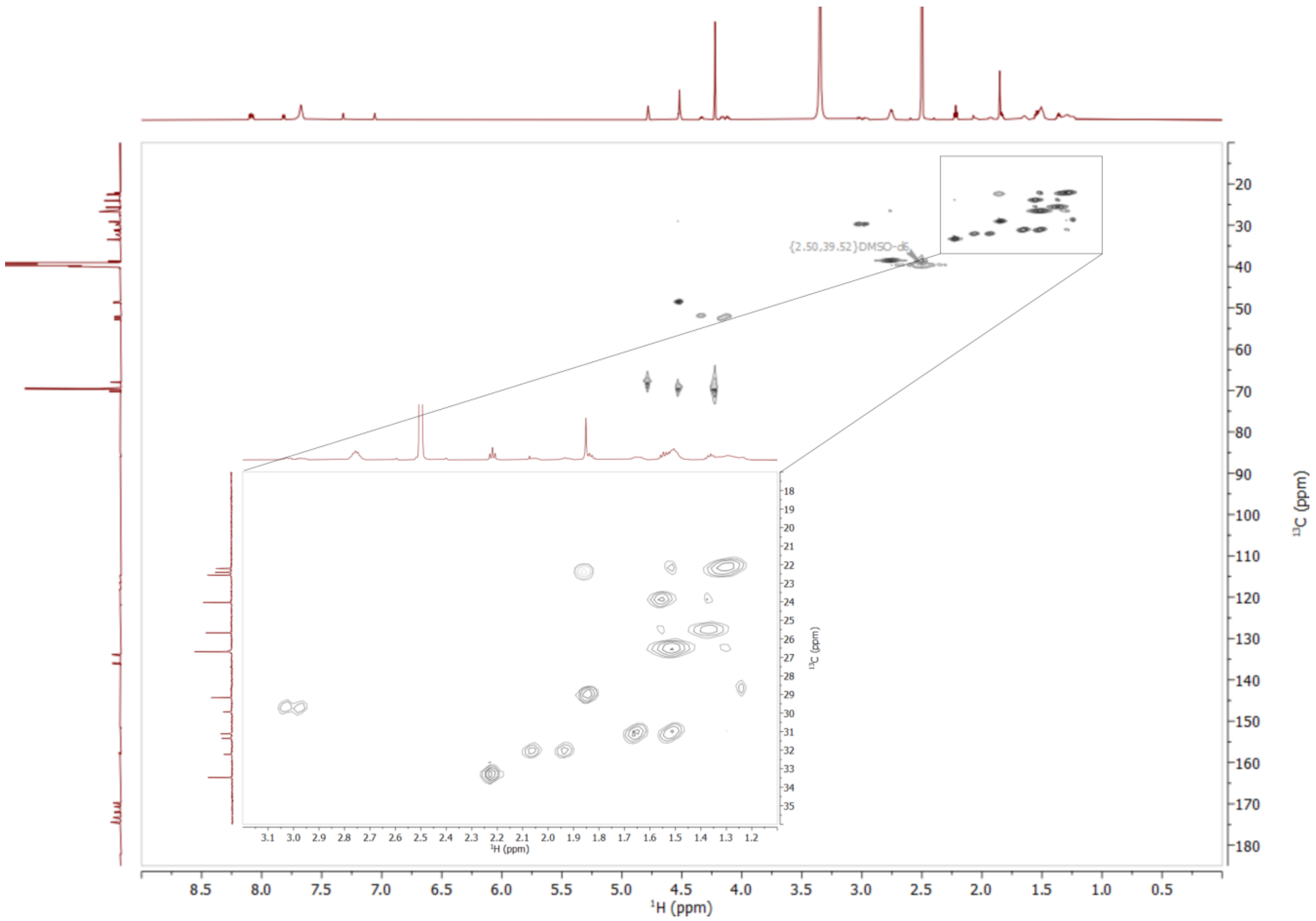

Figure S145: ^{1}H-^{13}C HSQC spectrum (700 MHz, 176 MHz, DMSO-d_6, 295 K) of **P5A20Az1_F1**.

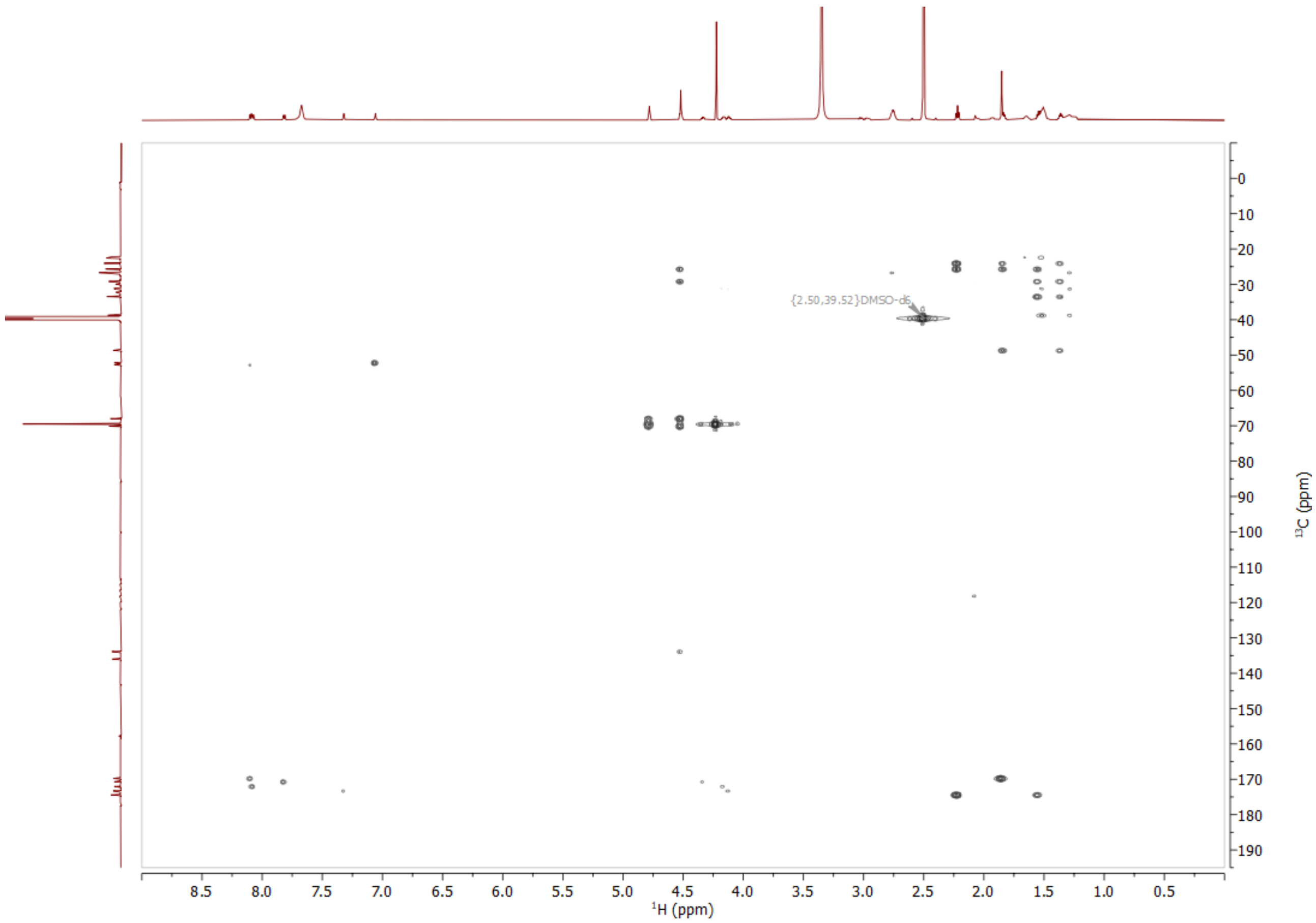

Figure S146: ^{1}H-^{13}C HMBC spectrum (700 MHz, 176 MHz, DMSO-d_6, 295 K) of **P5A20Az1_F2**.

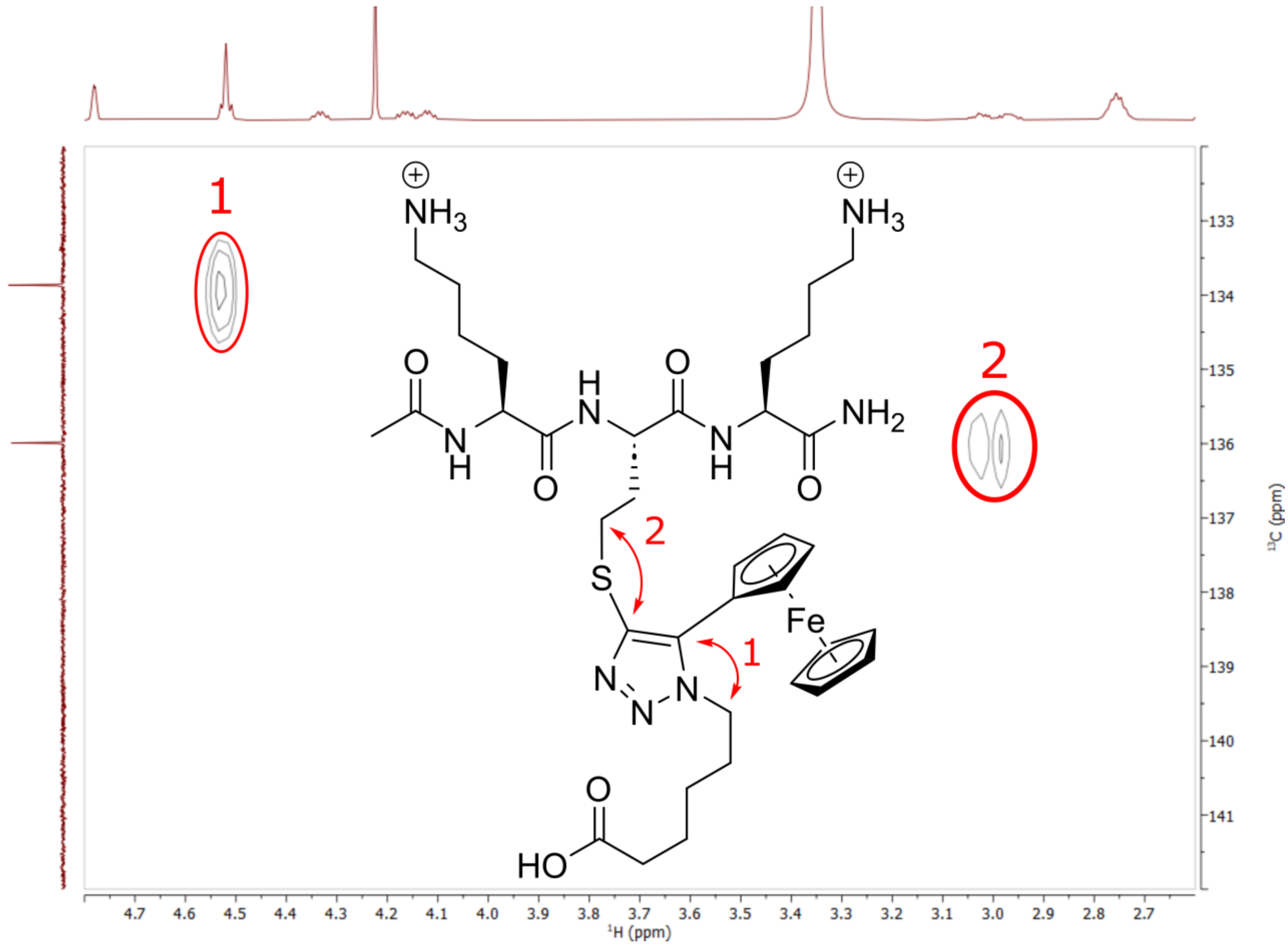

Figure S147: Extract from the ^{1}H-^{13}C HMBC spectrum (700 MHz, 176 MHz, DMSO-d_6, 295 K) of **P5A20Az1_F2** showing the cross-peaks relevant for isomer determination.

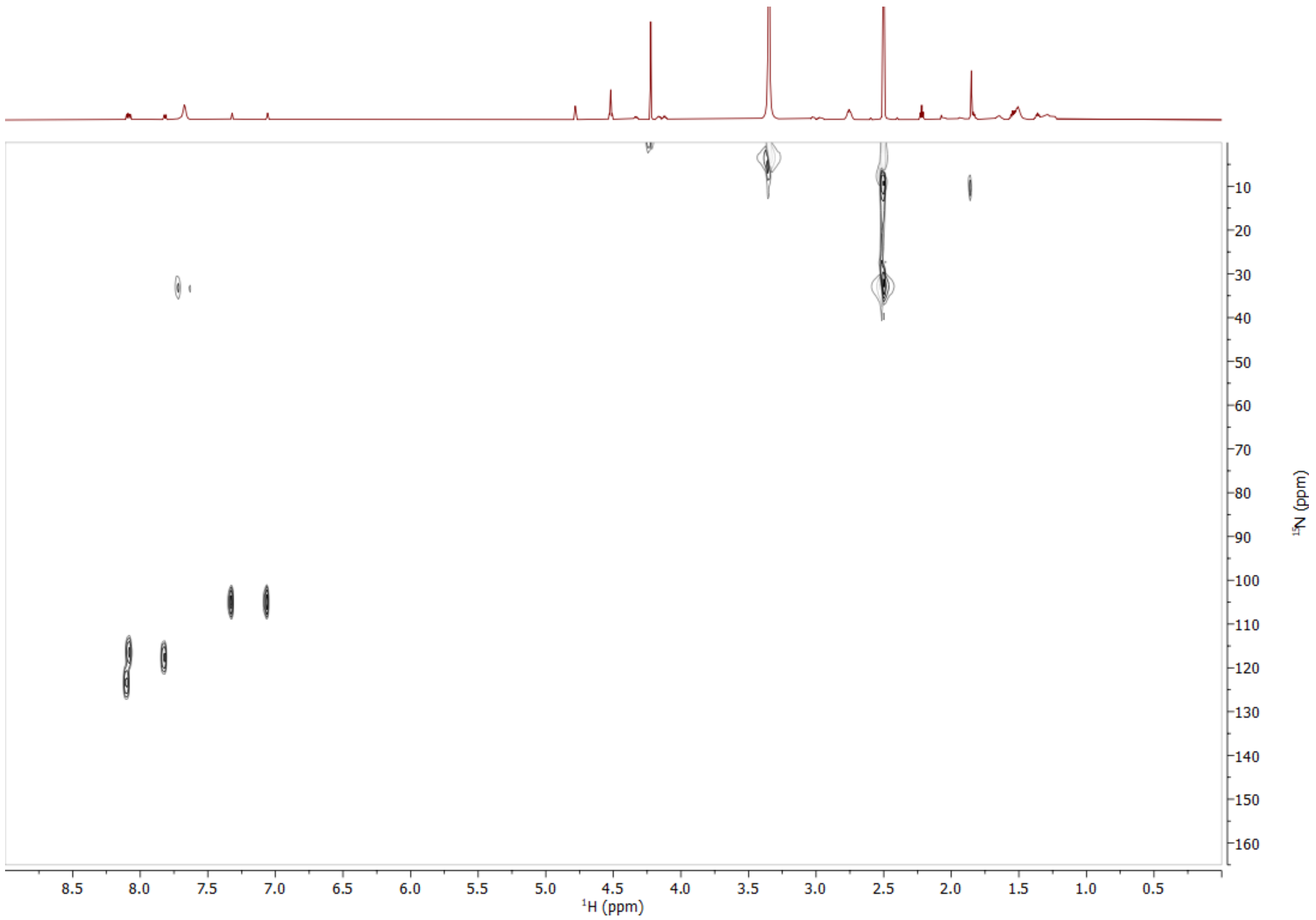

Figure S148: ^{1}H-^{15}N HSQC (700 MHz, 70.9 MHz, DMSO-d_6, 295 K) of **P5A20Az1_F2**.

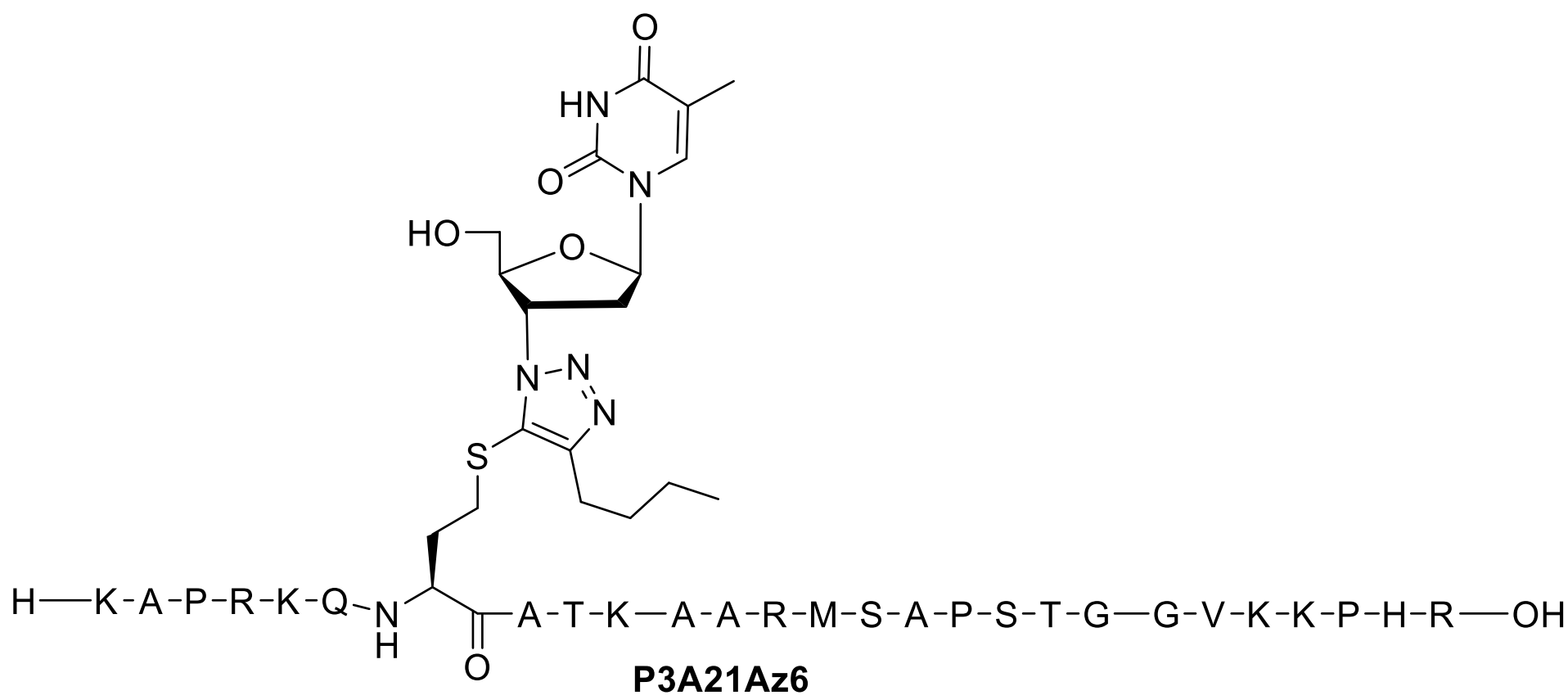

P3A21Az6
Chemical Formula: $C_{137}H_{235}N_{49}O_{37}S_2$
Exact Mass: 3222.75
Molecular Weight: 3224.81

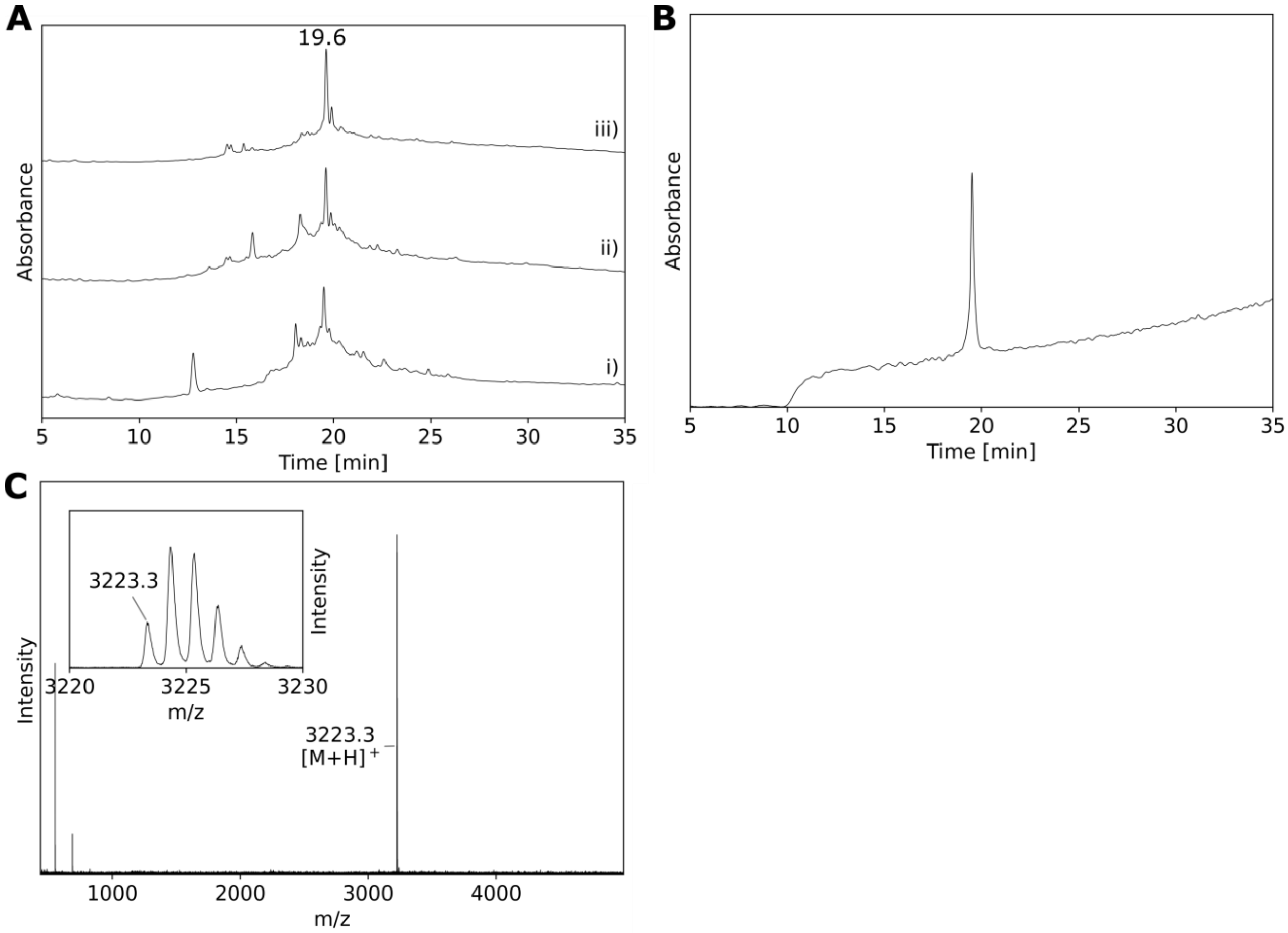

Figure S149: **P3A21Az6** A) Analytical HPLC (220 nm) of crude peptide after (i) standard IrAAC conditions, (ii) adjusted IrAAC conditions, (iii) adjusted IrAAC conditions and methionine sulfoxide reduction with trimethylsilyl bromide and ethane-1,2-dithiol. HPLC Gradients are listed in Section 2.3.5 Table S3. B) Analytical HPLC (220 nm) of purified peptide with a retention time of 30.1 min. C) MALDI-TOF MS of purified peptide with a retention time of 30.1 min. D) MALDI-TOF MS of purified peptide with a retention time of 30.6 min. Calculated mass: $[M+H]^+$ 3223.8.

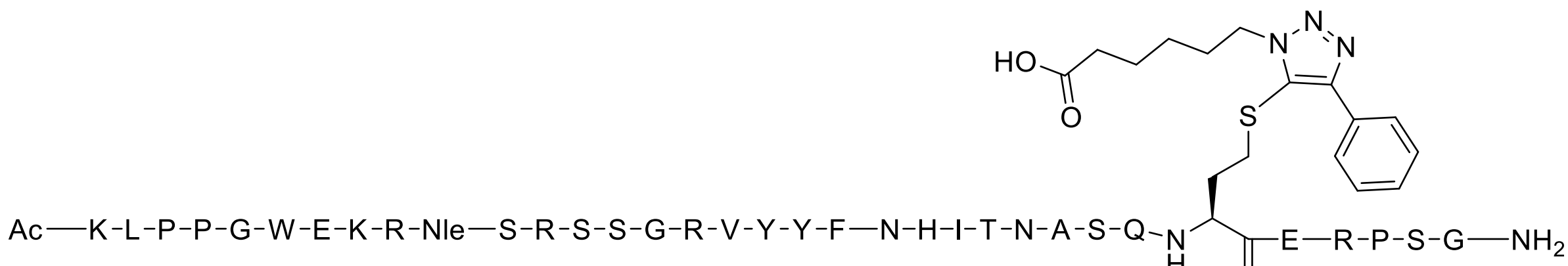

P4A1Az1

Chemical Formula: $C_{189}H_{286}N_{58}O_{52}S$
Exact Mass: 4232.12
Molecular Weight: 4234.78

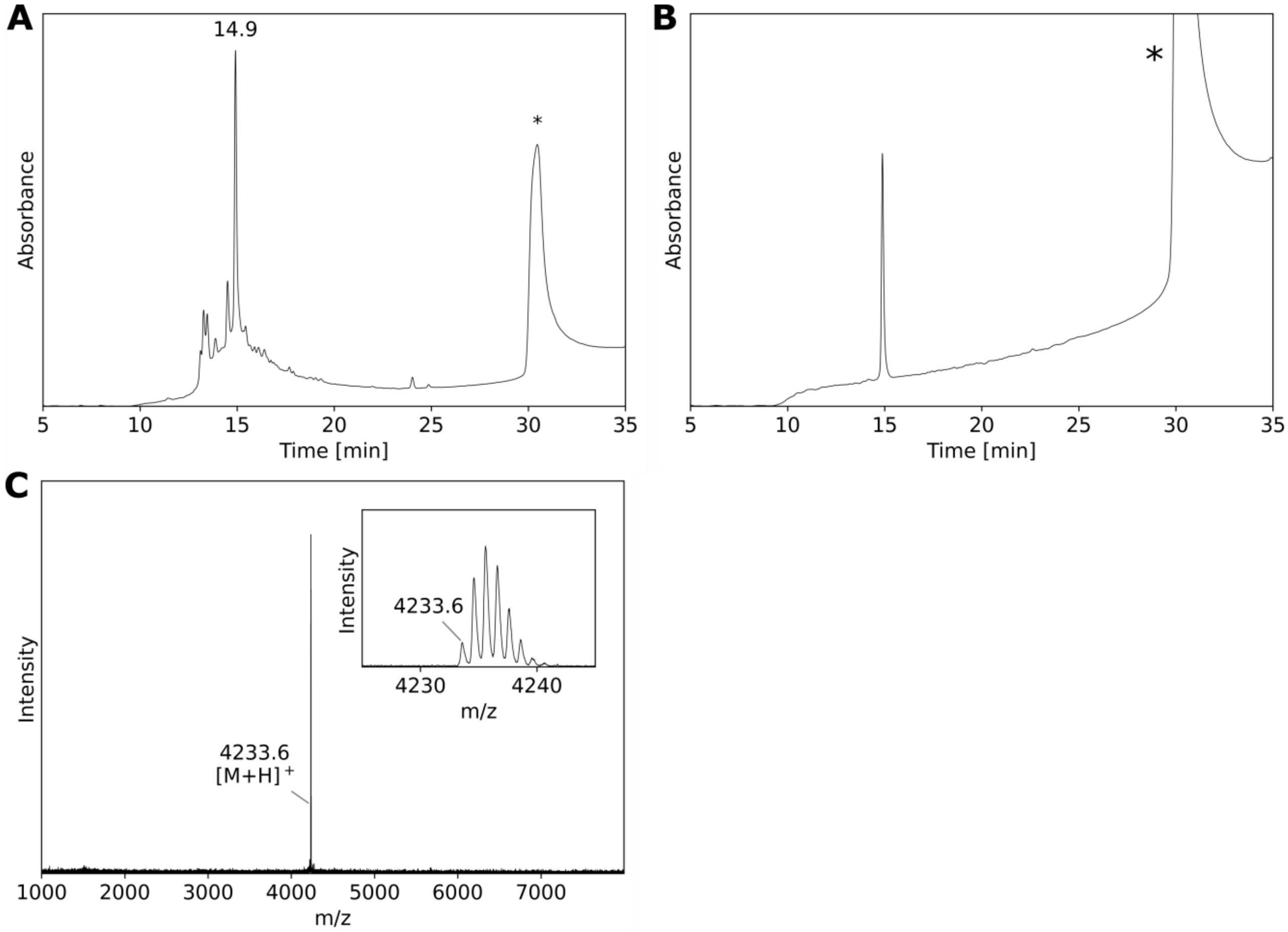

Figure S150: **P4A1Az1** A) Analytical HPLC (220 nm) of crude peptide. HPLC Gradients are listed in Section 2.3.5 Table S3. B) Analytical HPLC (220 nm) of pure peptide. * Carryover peak. C) MALDI-TOF MS of pure peptide. Calculated mass: $[M+H]^+$ 4233.1.

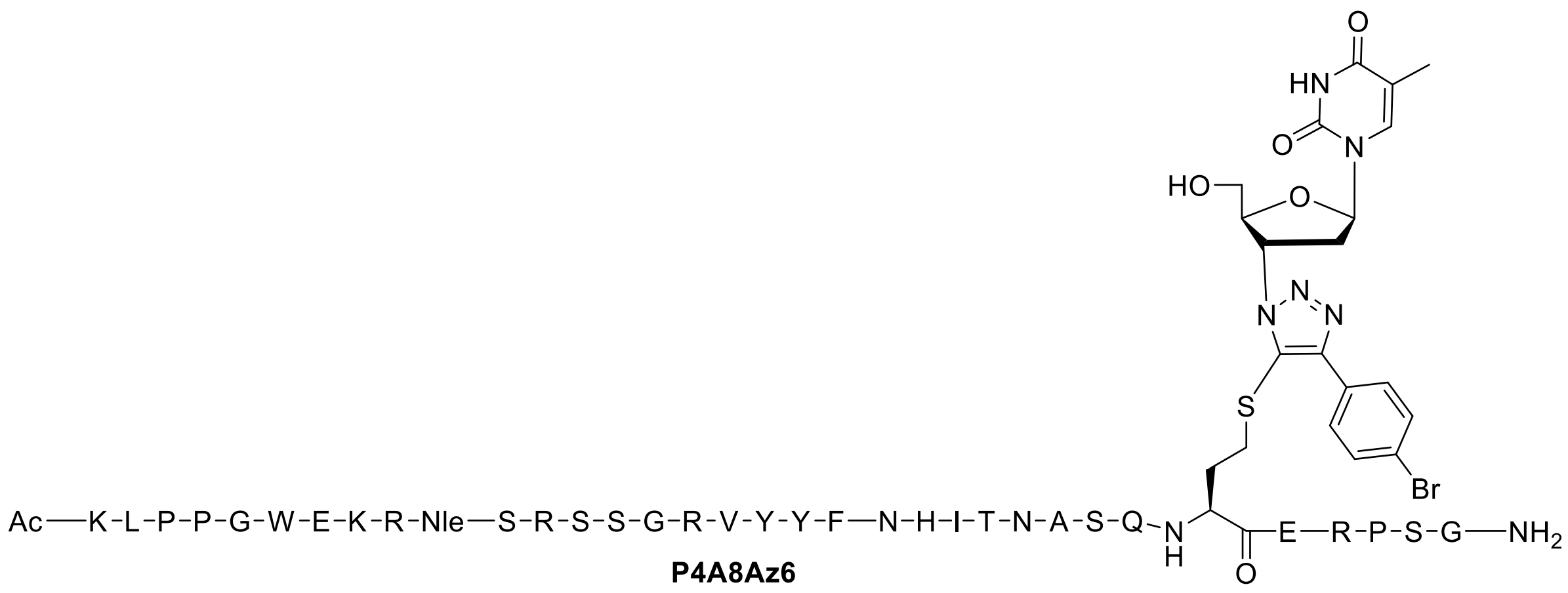

P4A8Az6

Chemical Formula: $C_{193}H_{287}BrN_{60}O_{54}S$
Exact Mass: 4420.05
Molecular Weight: 4423.75

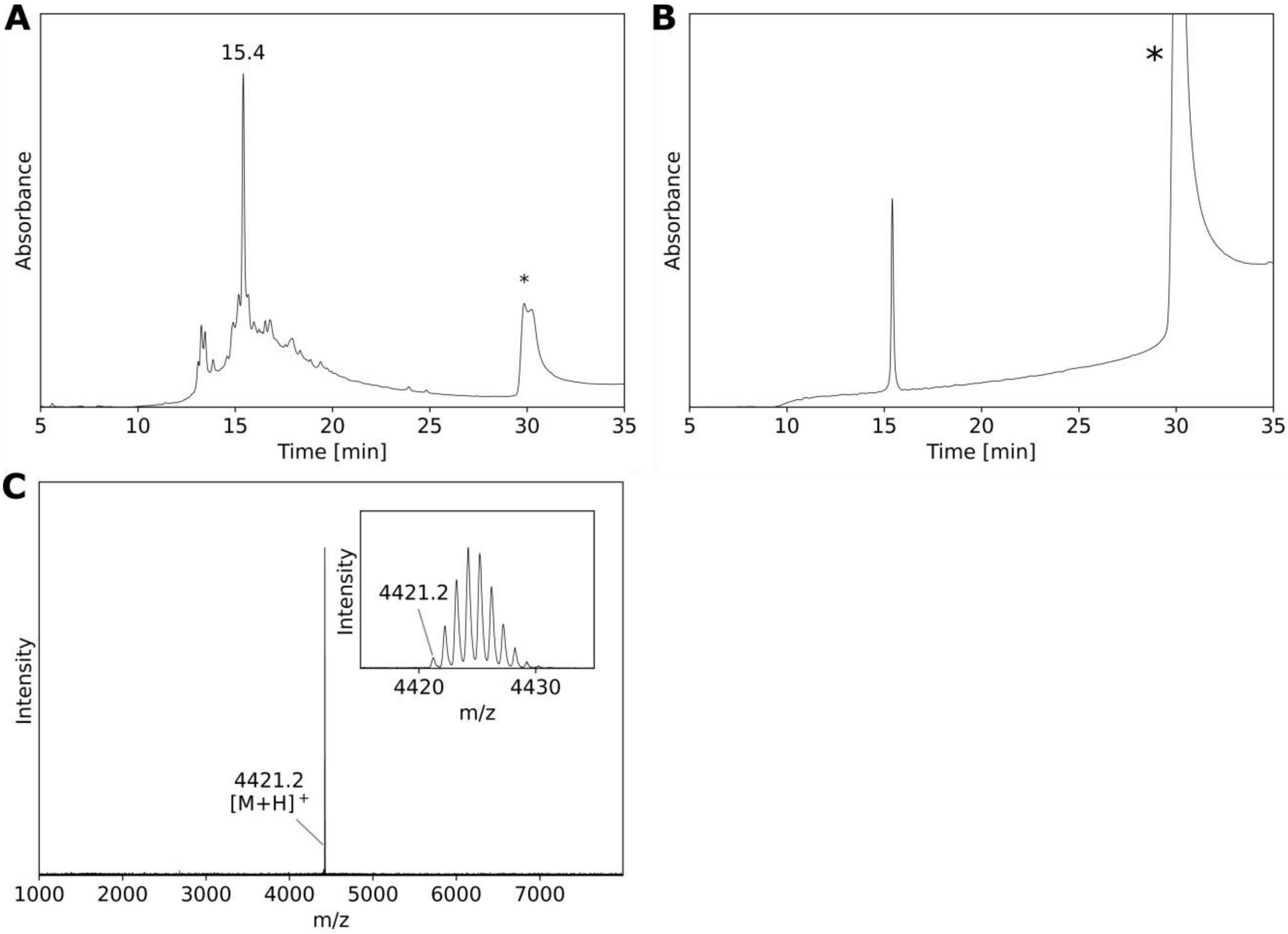

Figure S151: **P4A8Az6** A) Analytical HPLC (220 nm) of crude peptide. HPLC Gradients are listed in Section 2.3.5 Table S3. B) Analytical HPLC (220 nm) of pure peptide. * Carryover peak. C) MALDI-TOF MS of pure peptide. Calculated mass: $[M+H]^+$ 4421.1.

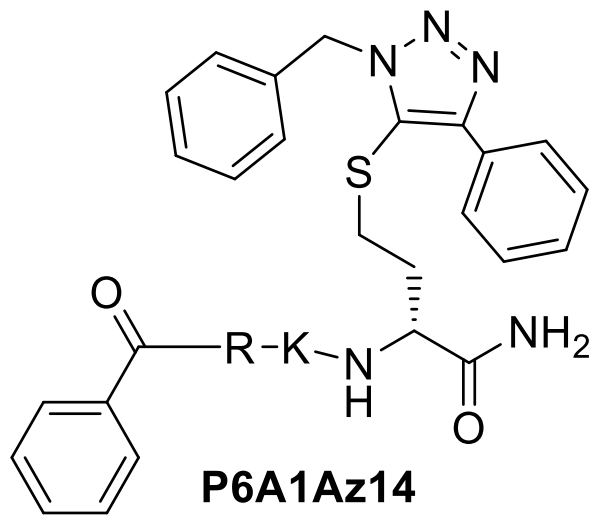

P6A1Az14

Chemical Formula: $C_{38}H_{49}N_{11}O_4S$
Exact Mass: 755.37
Molecular Weight: 755.94

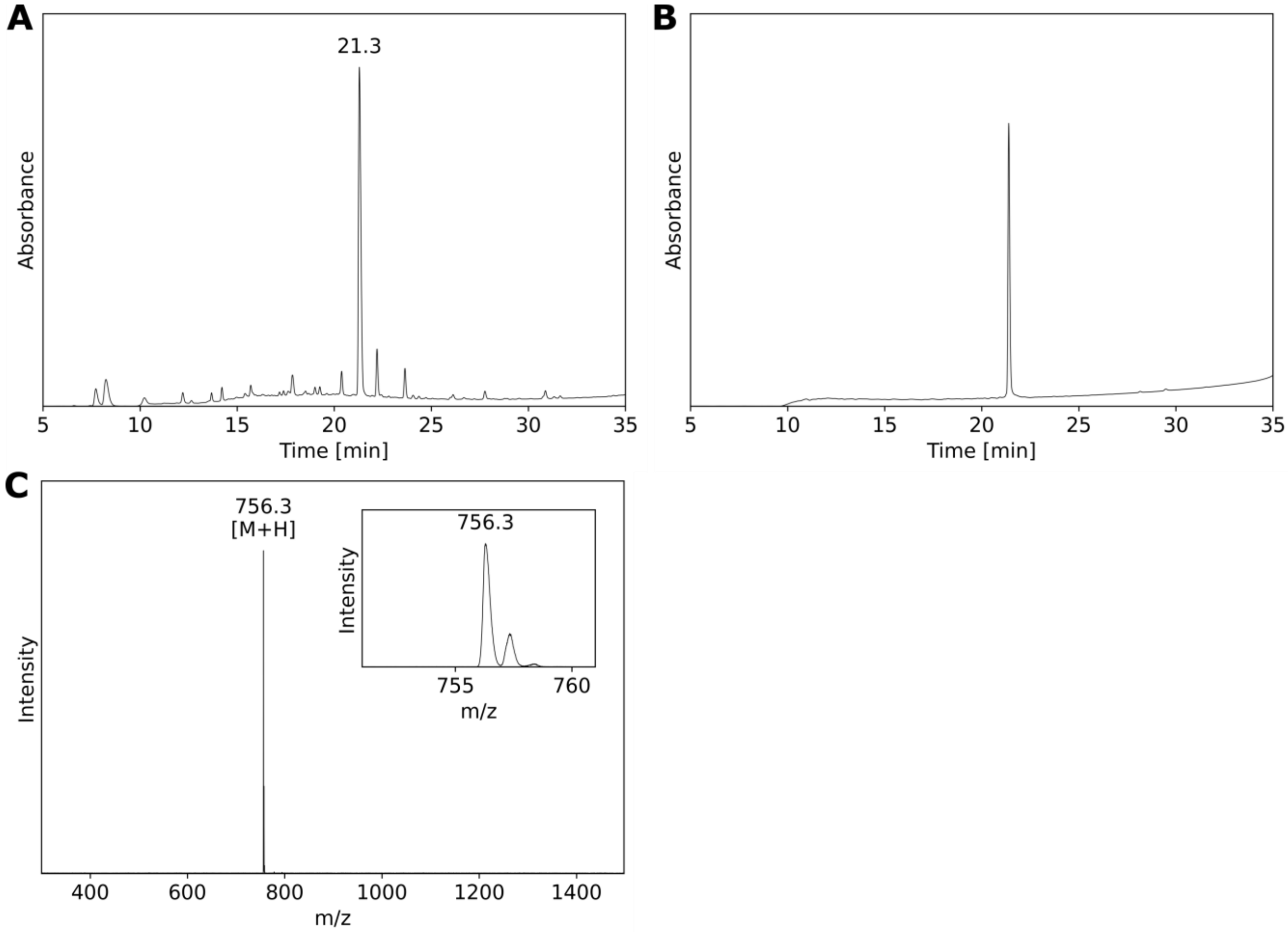

Figure S152: **P6A1Az14** A) Analytical HPLC (220 nm) of crude peptide. HPLC Gradients are listed in Section 2.3.5 Table S3. B) Analytical HPLC (220 nm) of pure peptide. C) MALDI-TOF MS of pure peptide. Calculated mass: $[M+H]^+$ 756.4.

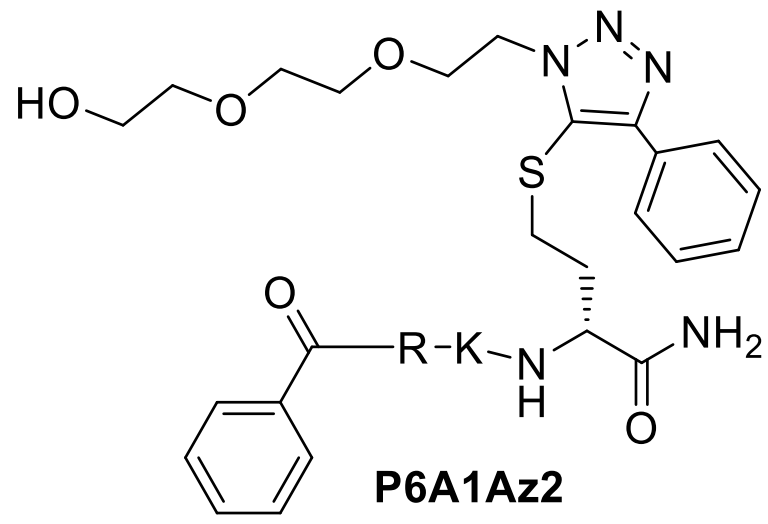

P6A1Az2

Chemical Formula: $C_{37}H_{55}N_{11}O_7S$
Exact Mass: 797.40
Molecular Weight: 797.98

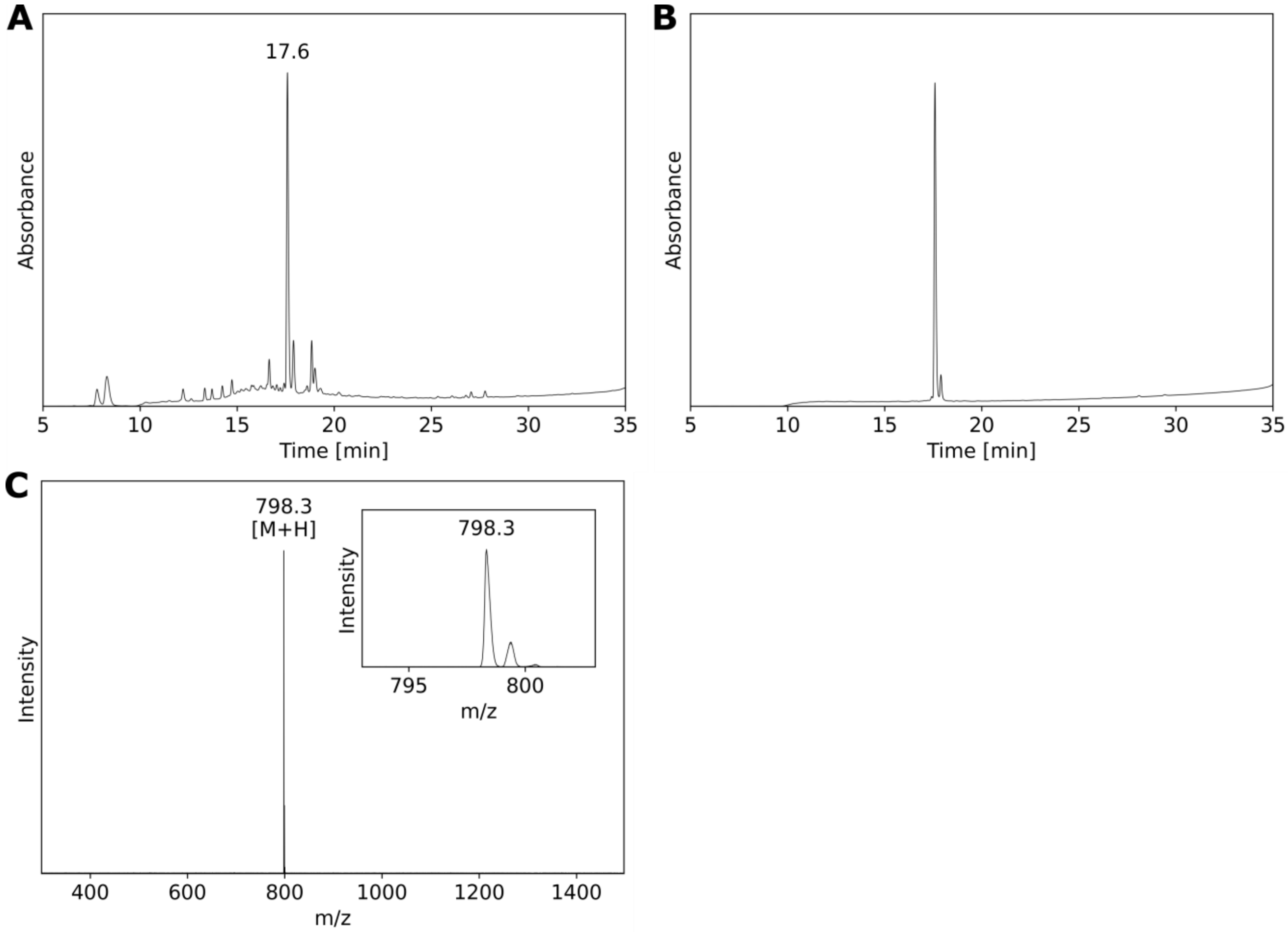

Figure S153: **P6A1Az2** A) Analytical HPLC (220 nm) of crude peptide. HPLC Gradients are listed in Section 2.3.5 Table S3. B) Analytical HPLC (220 nm) of pure peptide. C) MALDI-TOF MS of pure peptide. Calculated mass: $[M+H]^+$ 798.4.

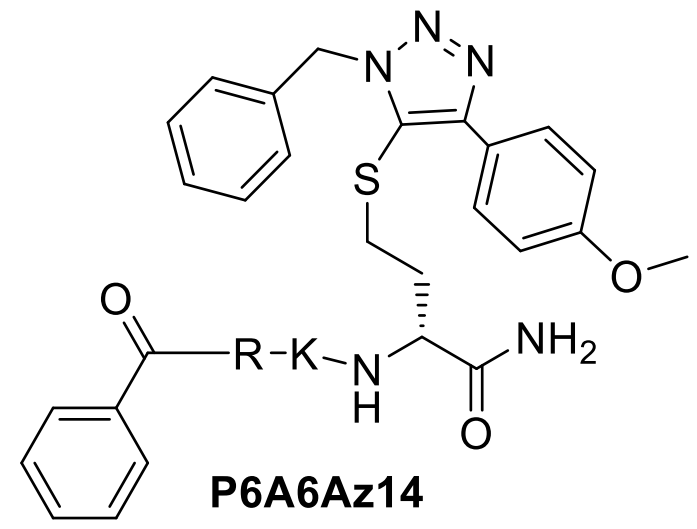

P6A6Az14

Chemical Formula: $C_{39}H_{51}N_{11}O_5S$
Exact Mass: 785.38
Molecular Weight: 785.97

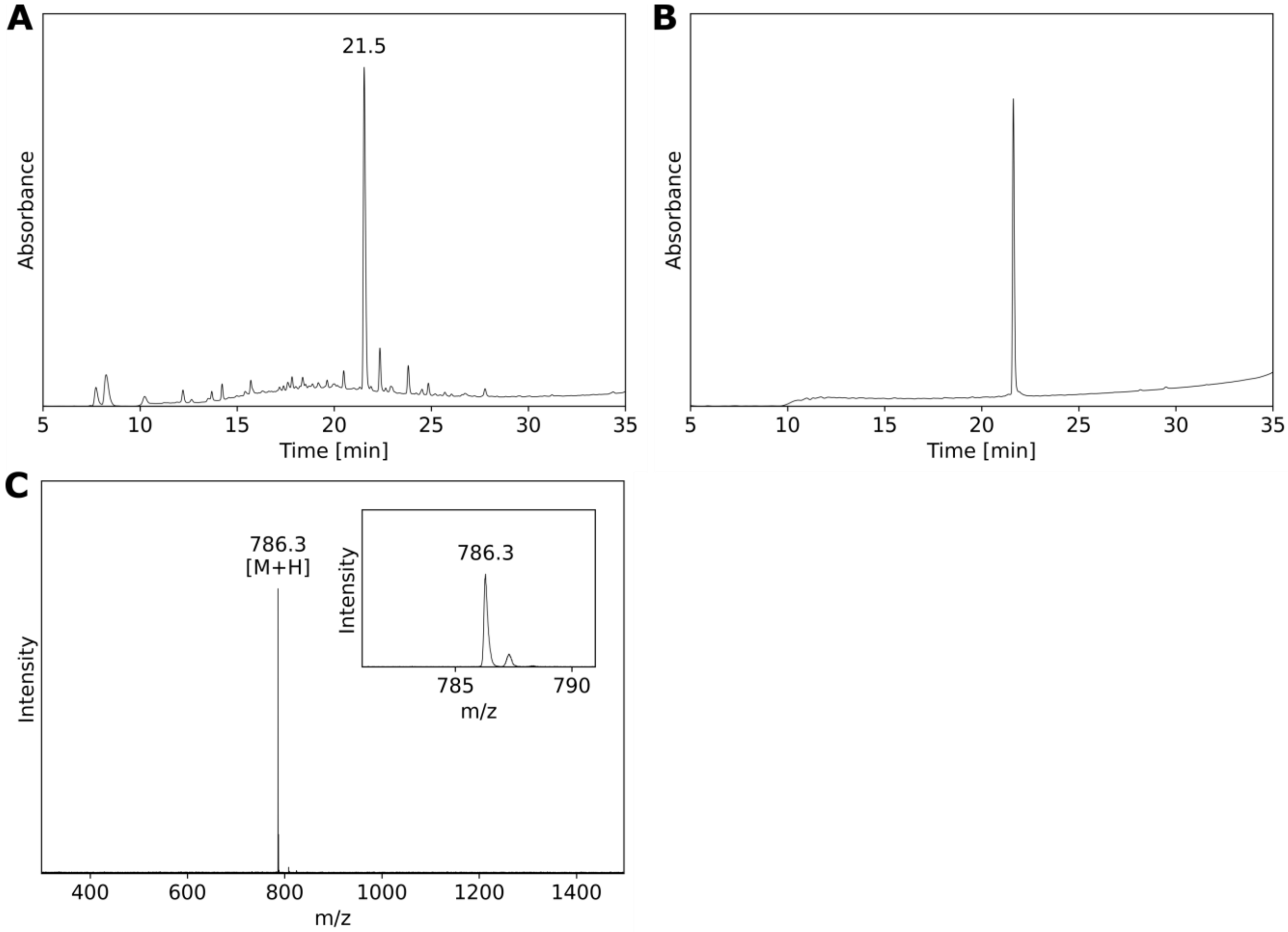

Figure S154: **P6A6Az14** A) Analytical HPLC (220 nm) of crude peptide. HPLC Gradients are listed in Section 2.3.5 Table S3. B) Analytical HPLC (220 nm) of pure peptide. C) MALDI-TOF MS of pure peptide. Calculated mass: $[M+H]^+$ 786.4.

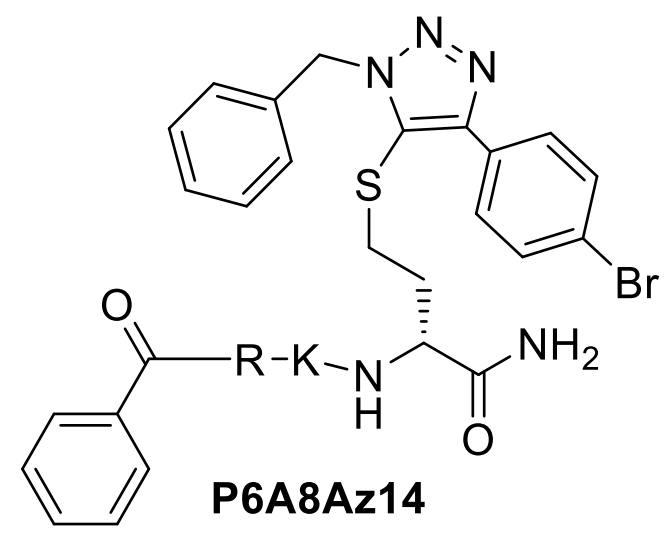

P6A8Az14

Chemical Formula: $C_{38}H_{48}BrN_{11}O_4S$
Exact Mass: 833.28
Molecular Weight: 834.84

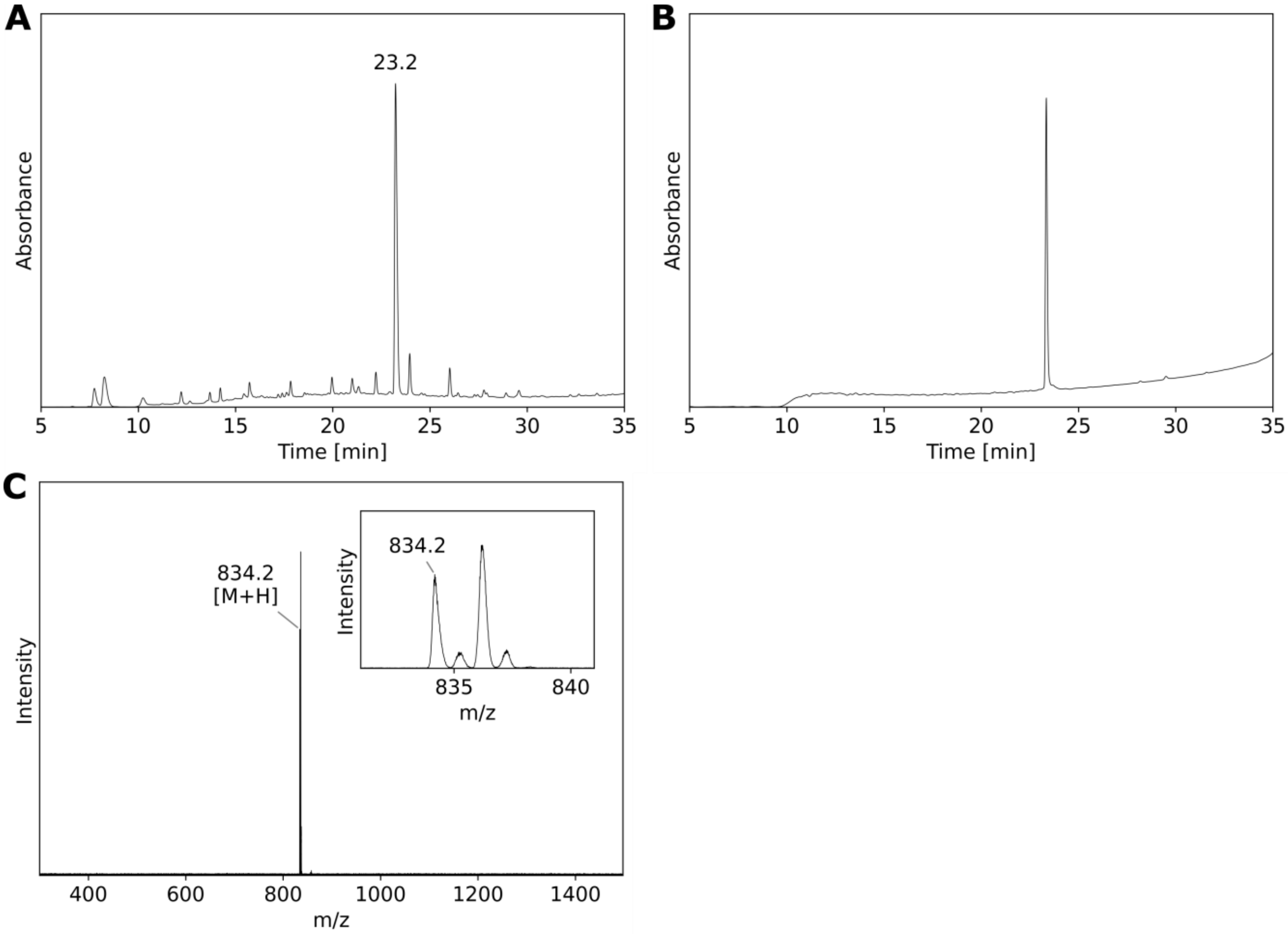

Figure S155: **P6A8Az14** A) Analytical HPLC (220 nm) of crude peptide. HPLC Gradients are listed in Section 2.3.5 Table S3. B) Analytical HPLC (220 nm) of pure peptide. C) MALDI-TOF MS of pure peptide. Calculated mass: $[M+H]^+$ 834.3.

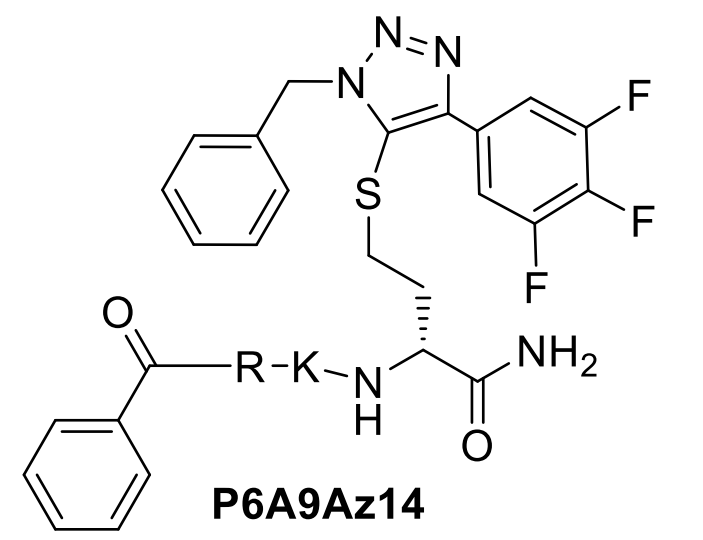

P6A9Az14

Chemical Formula: $C_{38}H_{46}F_3N_{11}O_4S$
Exact Mass: 809.34
Molecular Weight: 809.91

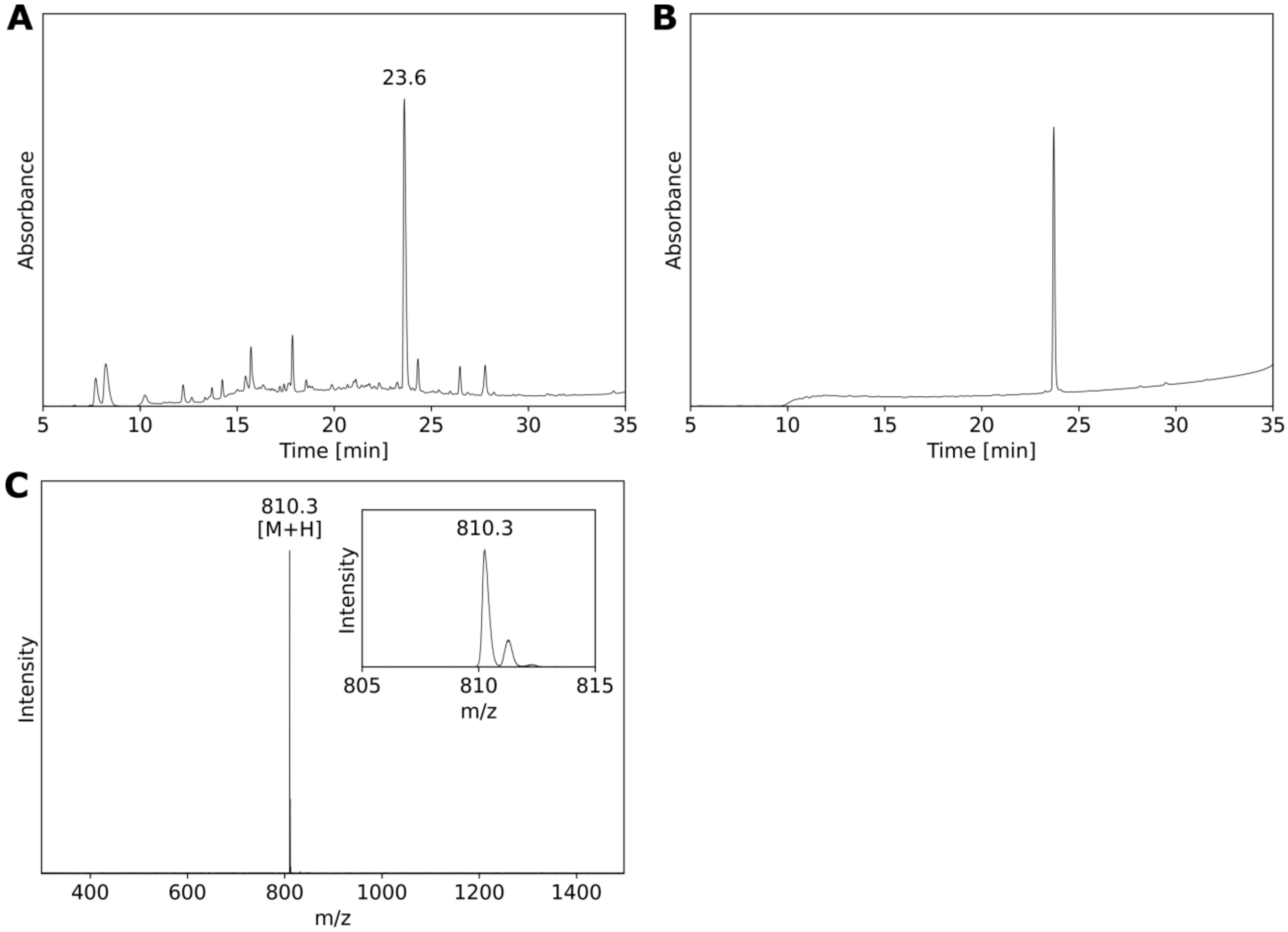

Figure S156: **P6A9Az14** A) Analytical HPLC (220 nm) of crude peptide. HPLC Gradients are listed in Section 2.3.5 Table S3. B) Analytical HPLC (220 nm) of pure peptide. C) MALDI-TOF MS of pure peptide. Calculated mass: $[M+H]^+$ 810.3.

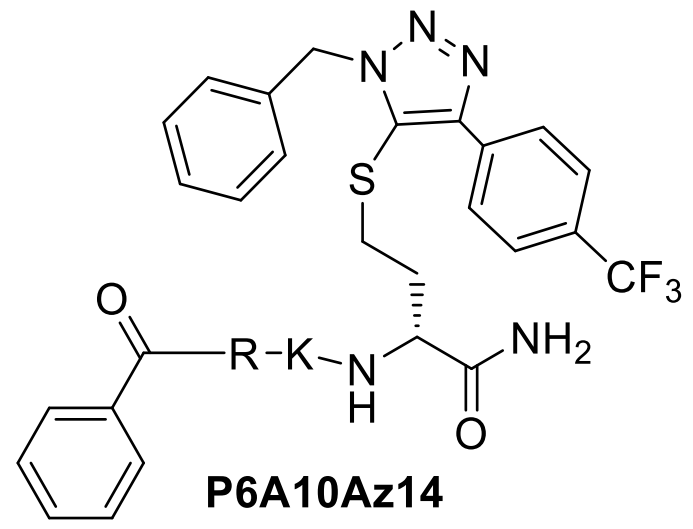

P6A10Az14

Chemical Formula: $C_{39}H_{48}F_3N_{11}O_4S$
Exact Mass: 823.36
Molecular Weight: 823.94

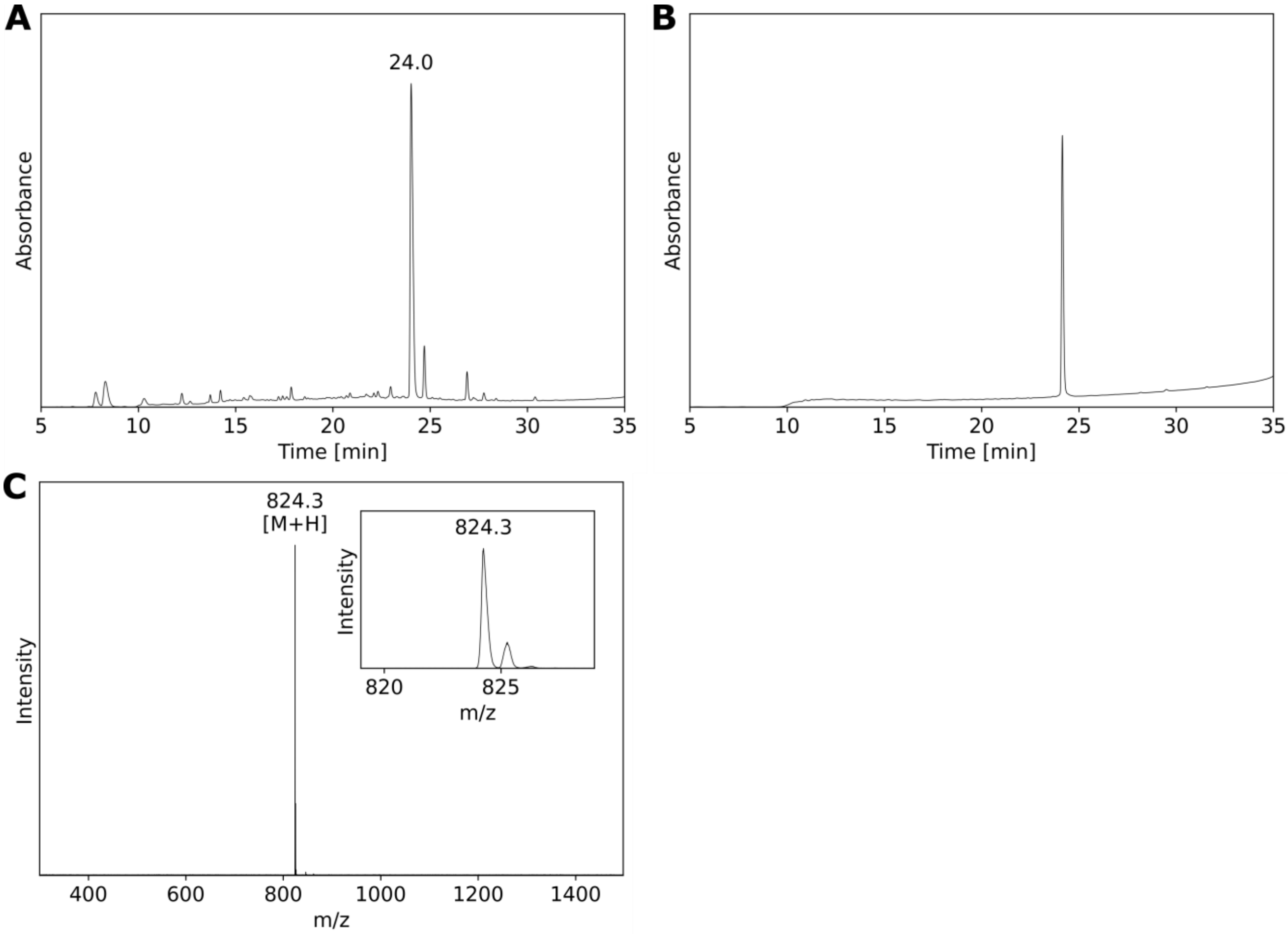

Figure S157: **P6A10Az14** A) Analytical HPLC (220 nm) of crude peptide. HPLC Gradients are listed in Section 2.3.5 Table S3. B) Analytical HPLC (220 nm) of pure peptide. C) MALDI-TOF MS of pure peptide. Calculated mass: $[M+H]^+$ 824.4.

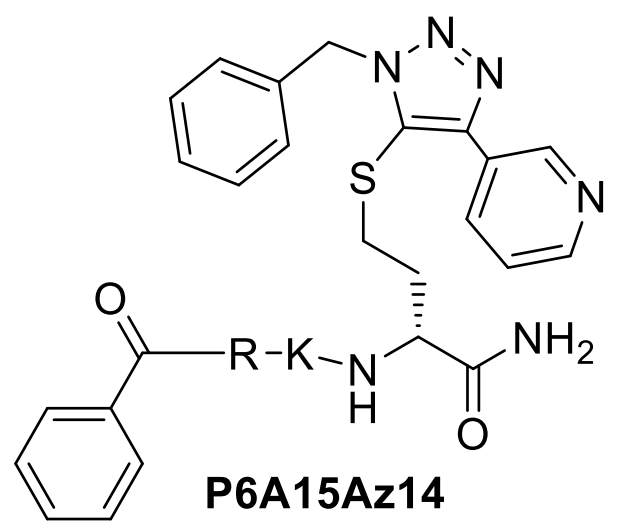

P6A15Az14

Chemical Formula: $C_{37}H_{48}N_{12}O_4S$
Exact Mass: 756.36
Molecular Weight: 756.93

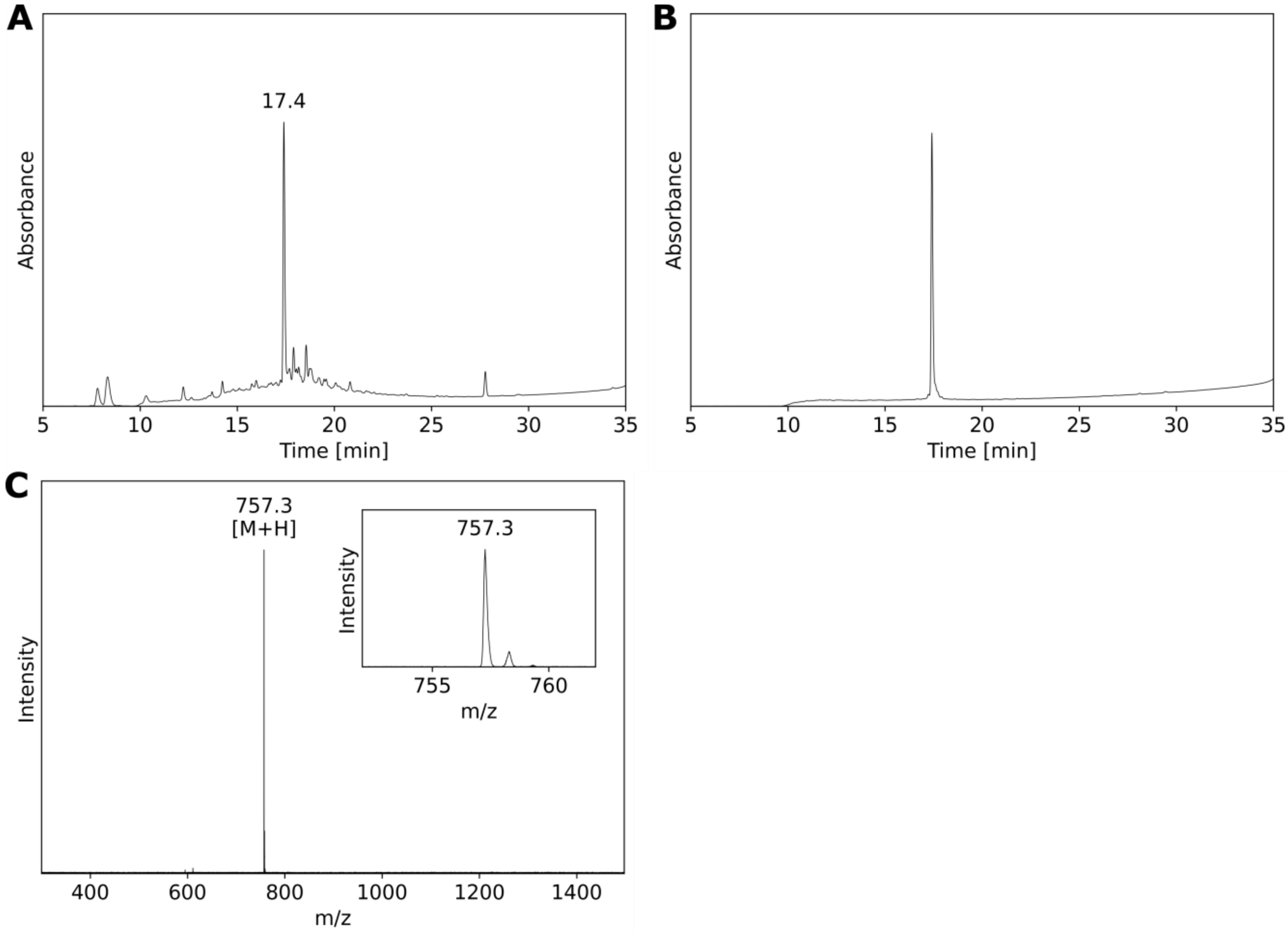

Figure S158: **P6A15Az14** A) Analytical HPLC (220 nm) of crude peptide. HPLC Gradients are listed in Section 2.3.5 Table S3. B) Analytical HPLC (220 nm) of pure peptide. C) MALDI-TOF MS of pure peptide. Calculated mass: $[M+H]^+$ 757.4.

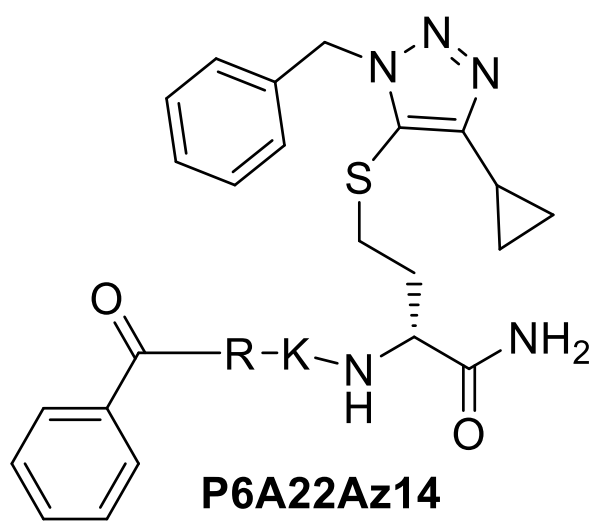

P6A22Az14

Chemical Formula: $C_{35}H_{49}N_{11}O_4S$
Exact Mass: 719.37
Molecular Weight: 719.91

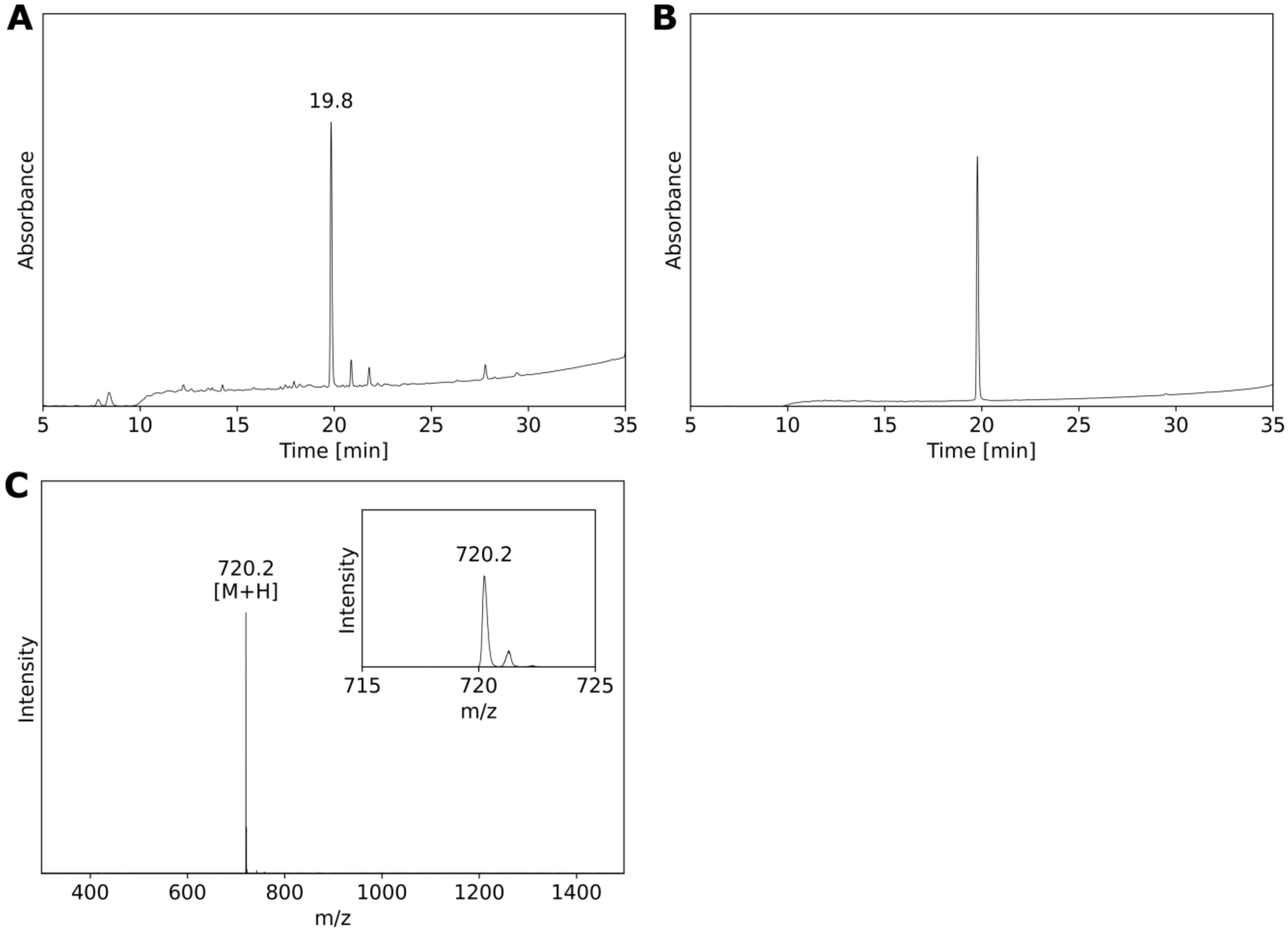

Figure S159: **P6A22Az14** A) Analytical HPLC (220 nm) of crude peptide. HPLC Gradients are listed in Section 2.3.5 Table S3. B) Analytical HPLC (220 nm) of pure peptide. C) MALDI-TOF MS of pure peptide. Calculated mass: $[M+H]^+$ 720.4.

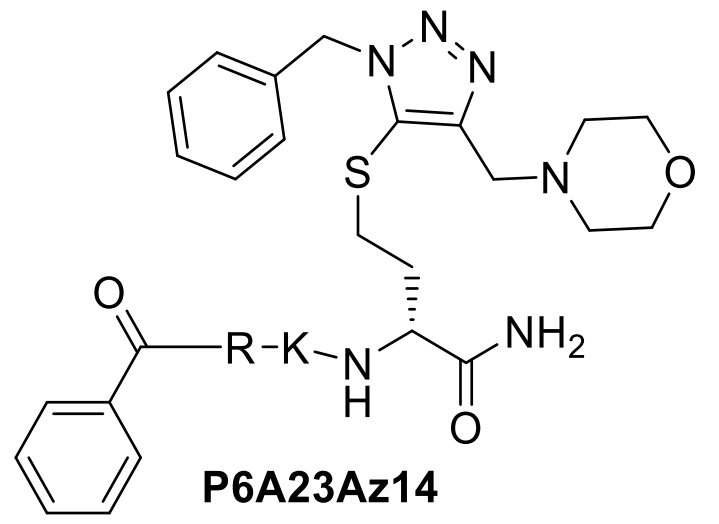

P6A23Az14

Chemical Formula: $C_{37}H_{54}N_{12}O_5S$
Exact Mass: 778.41
Molecular Weight: 778.98

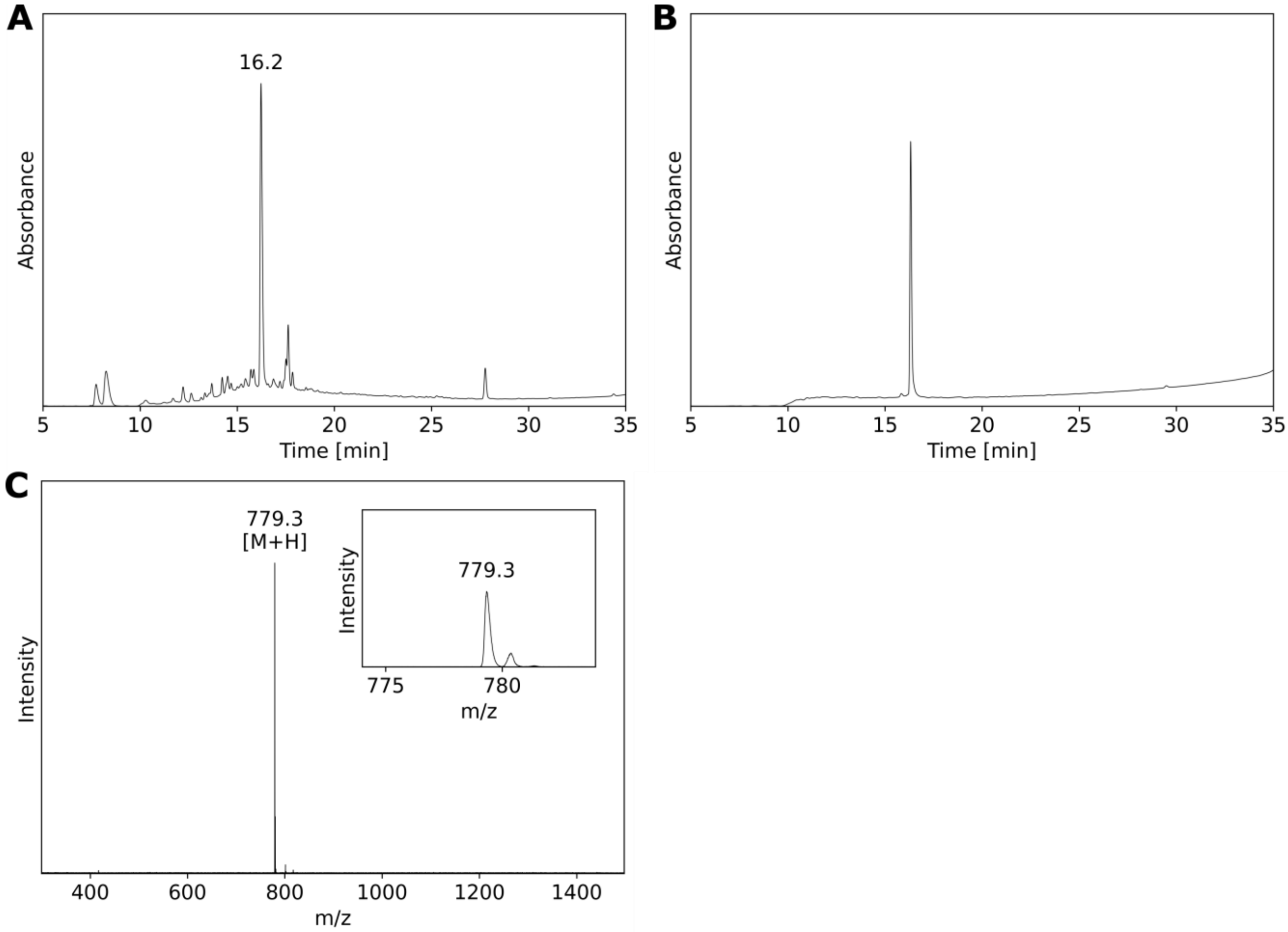

Figure S160: **P6A23Az14** A) Analytical HPLC (220 nm) of crude peptide. HPLC Gradients are listed in Section 2.3.5 Table S3. B) Analytical HPLC (220 nm) of pure peptide. C) MALDI-TOF MS of pure peptide. Calculated mass: $[M+H]^+$ 779.4.

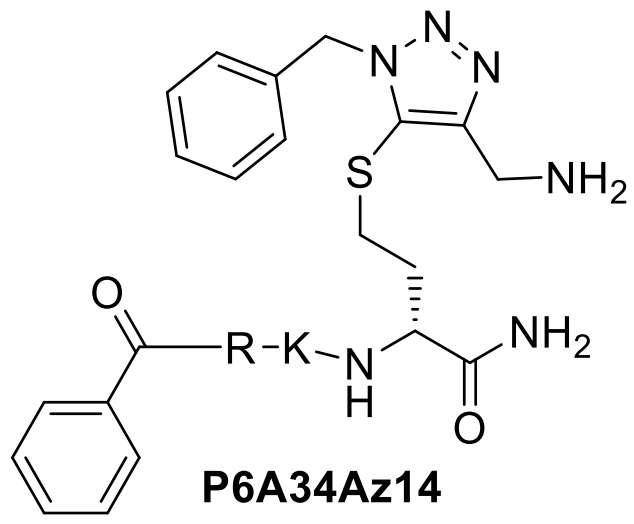

P6A34Az14

Chemical Formula: $C_{33}H_{48}N_{12}O_4S$
Exact Mass: 708.36
Molecular Weight: 708.89

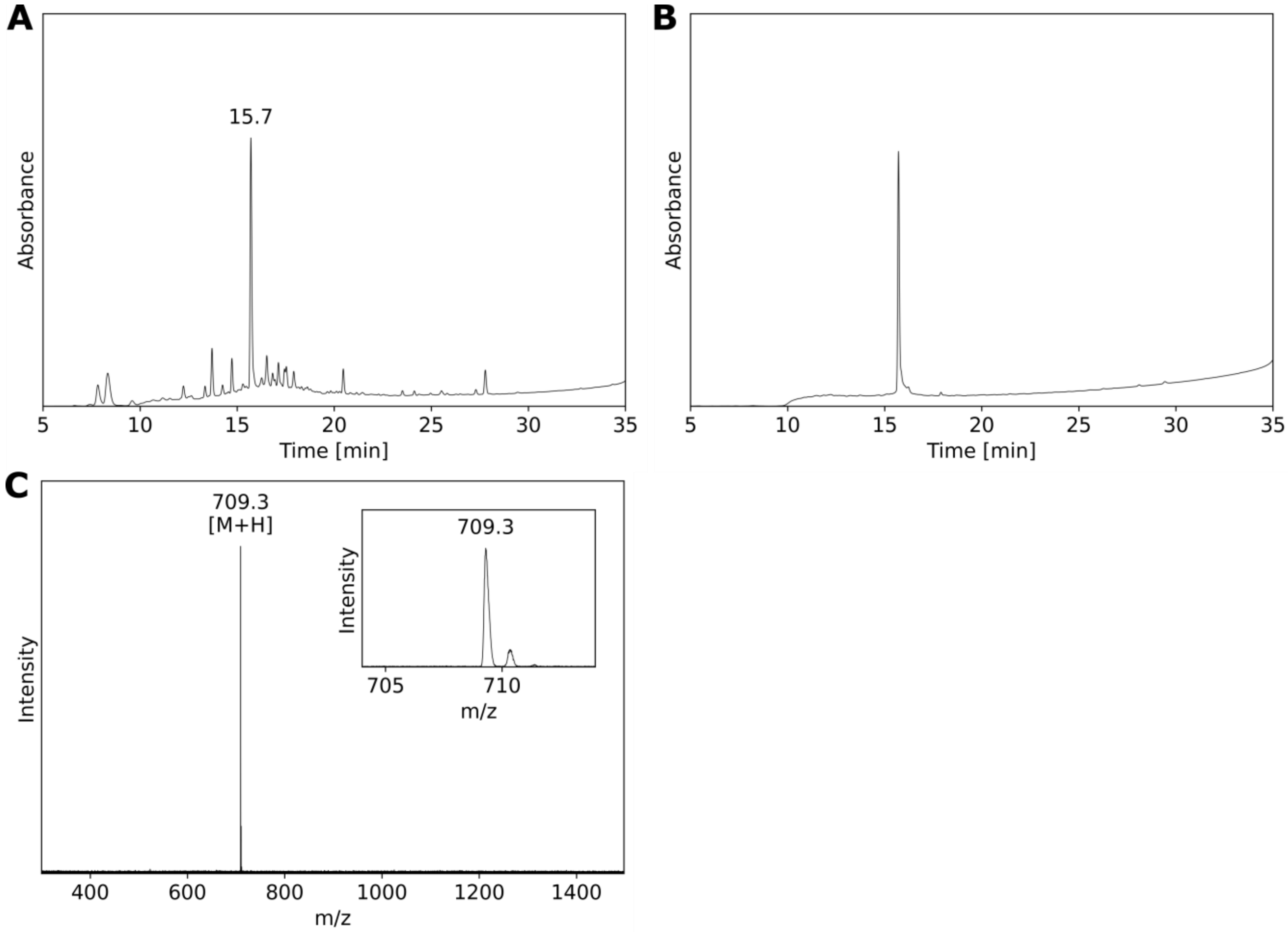

Figure S161: **P6A34Az14** A) Analytical HPLC (220 nm) of crude peptide. HPLC Gradients are listed in Section 2.3.5 Table S3. B) Analytical HPLC (220 nm) of pure peptide. C) MALDI-TOF MS of pure peptide. Calculated mass: $[M+H]^+$ 709.4.

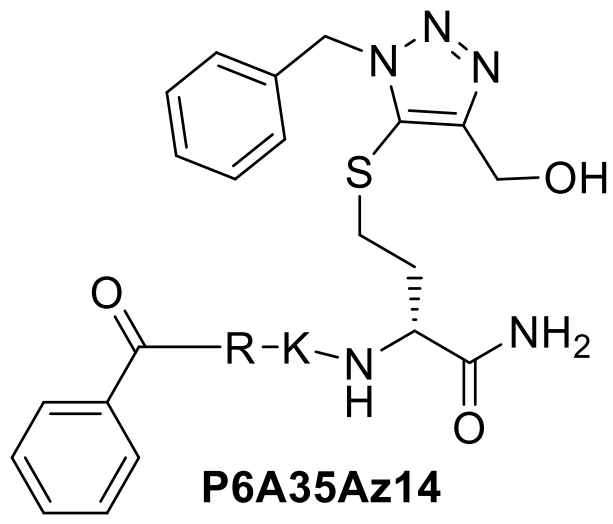

P6A35Az14

Chemical Formula: $C_{33}H_{47}N_{11}O_5S$
Exact Mass: 709.35
Molecular Weight: 709.87

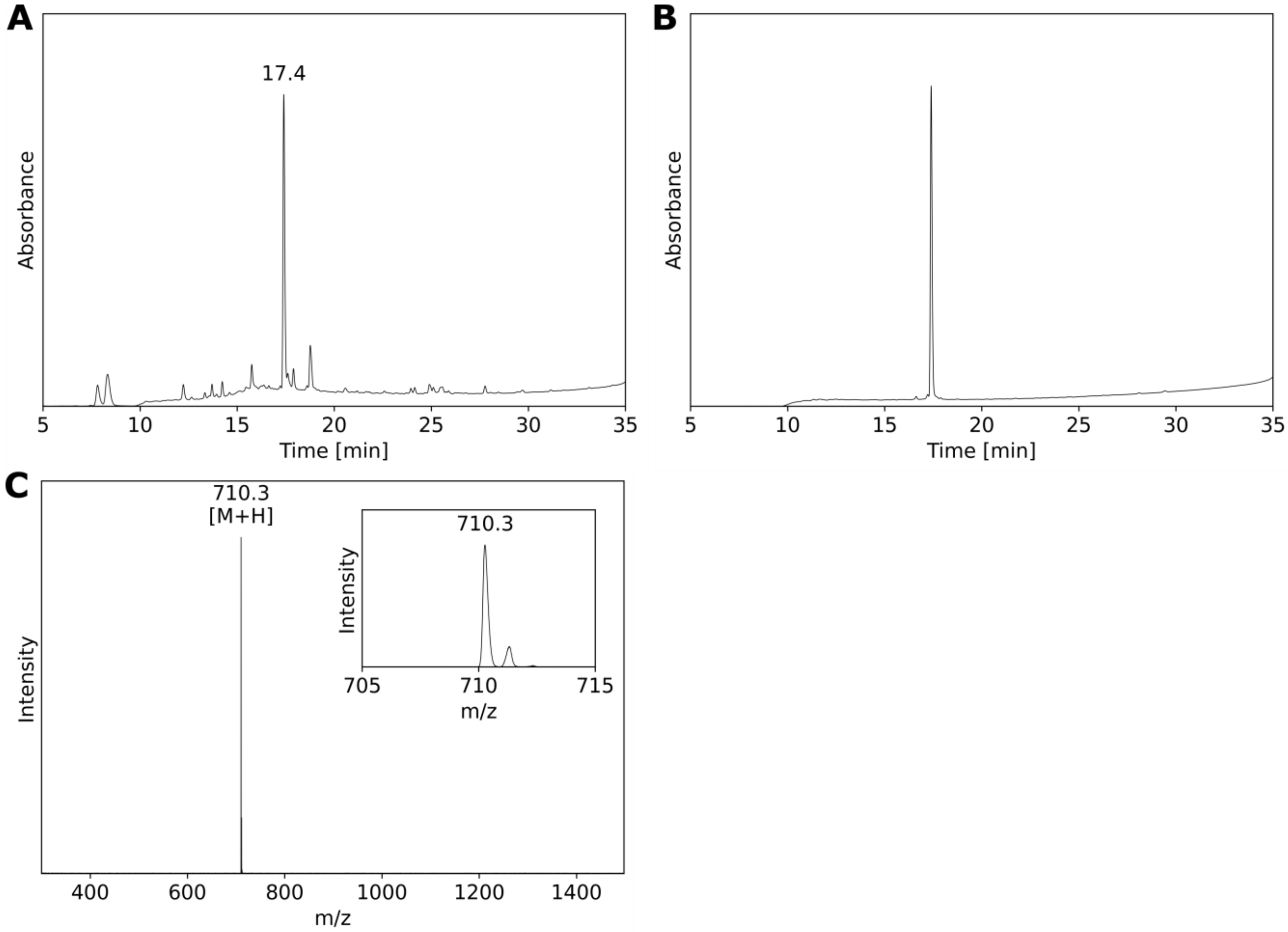

Figure S162: **P6A35Az14** A) Analytical HPLC (220 nm) of crude peptide. HPLC Gradients are listed in Section 2.3.5 Table S3. B) Analytical HPLC (220 nm) of pure peptide. C) MALDI-TOF MS of pure peptide. Calculated mass: $[M+H]^+$ 710.4.

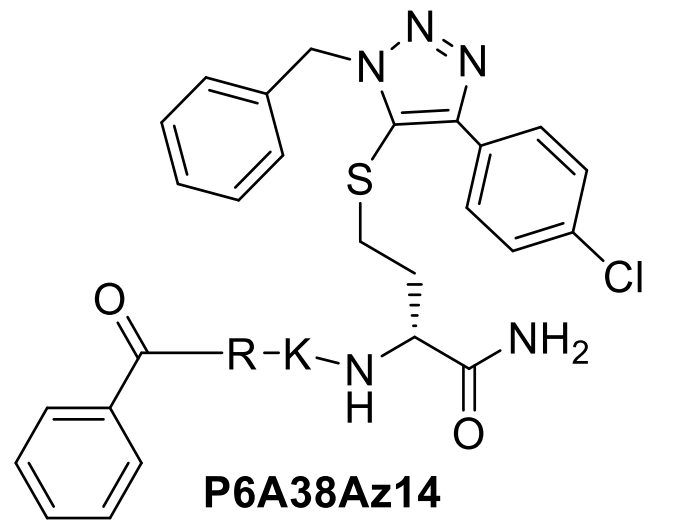

P6A38Az14

Chemical Formula: $C_{38}H_{48}ClN_{11}O_4S$
Exact Mass: 789.33
Molecular Weight: 790.39

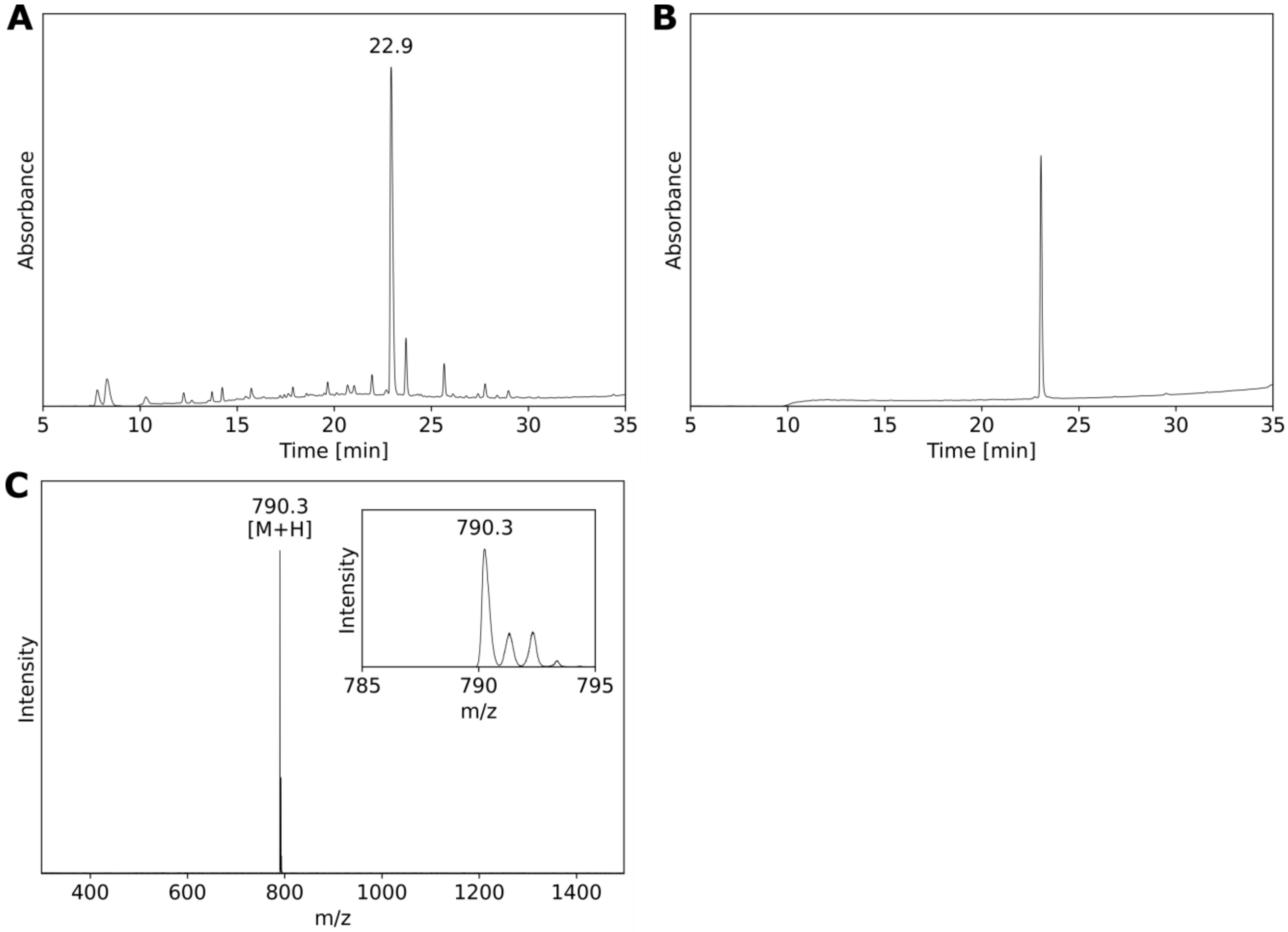

Figure S163: **P6A38Az14** A) Analytical HPLC (220 nm) of crude peptide. HPLC Gradients are listed in Section 2.3.5 Table S3. B) Analytical HPLC (220 nm) of pure peptide. C) MALDI-TOF MS of pure peptide. Calculated mass: $[M+H]^+$ 790.4.

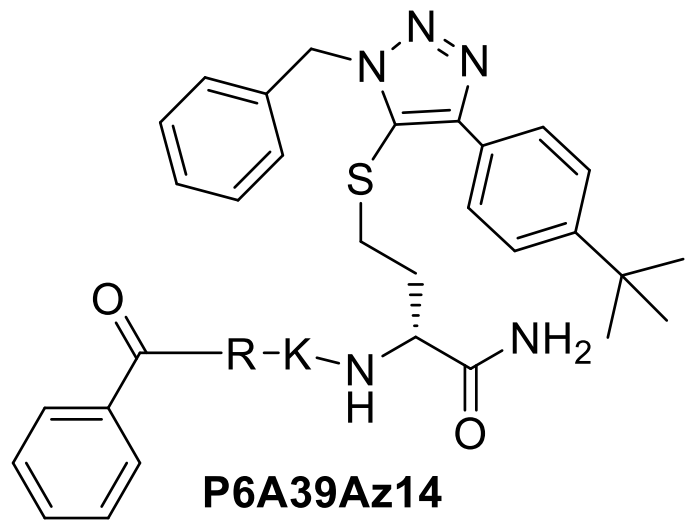

P6A39Az14

Chemical Formula: $C_{42}H_{57}N_{11}O_4S$
Exact Mass: 811.43
Molecular Weight: 812.05

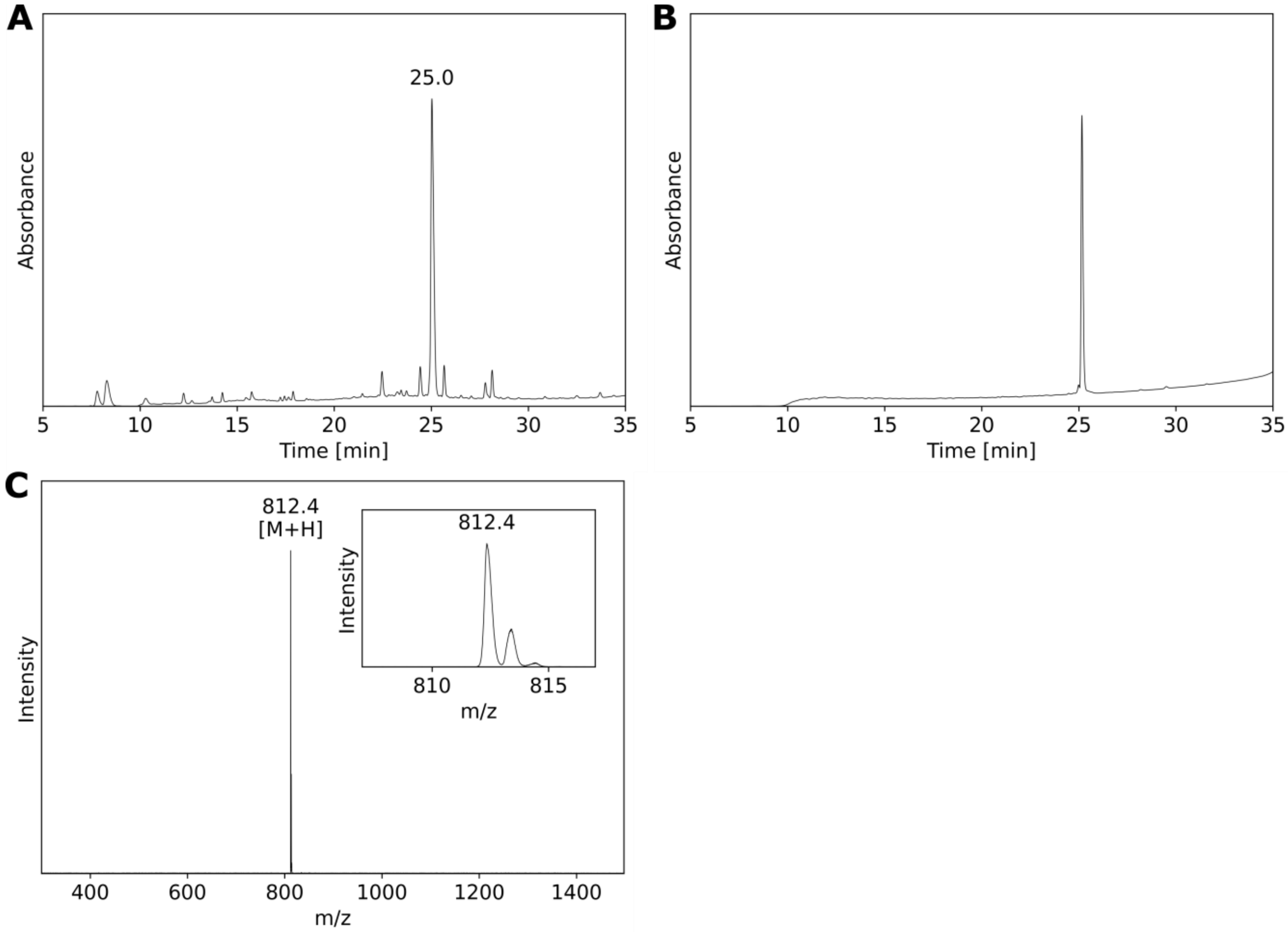

Figure S164: **P6A39Az14** A) Analytical HPLC (220 nm) of crude peptide. HPLC Gradients are listed in Section 2.3.5 Table S3. B) Analytical HPLC (220 nm) of pure peptide. C) MALDI-TOF MS of pure peptide. Calculated mass: $[M+H]^+$ 812.4.

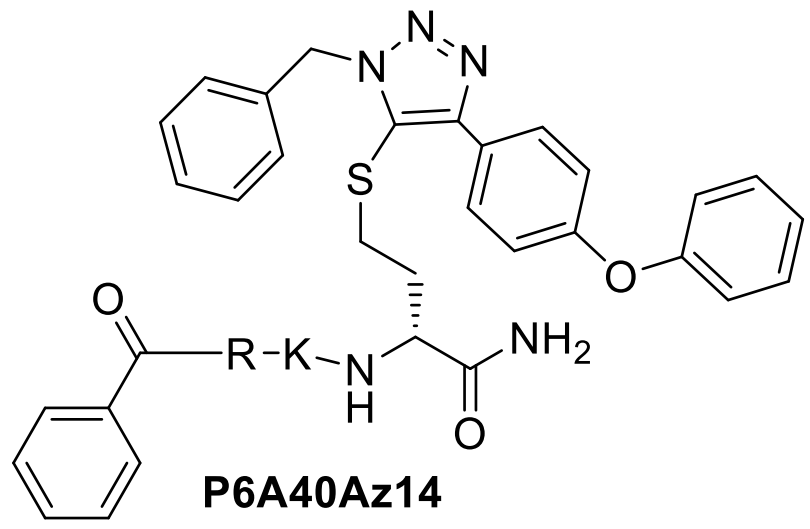

P6A40Az14

Chemical Formula: $C_{44}H_{53}N_{11}O_5S$
Exact Mass: 847.40
Molecular Weight: 848.04

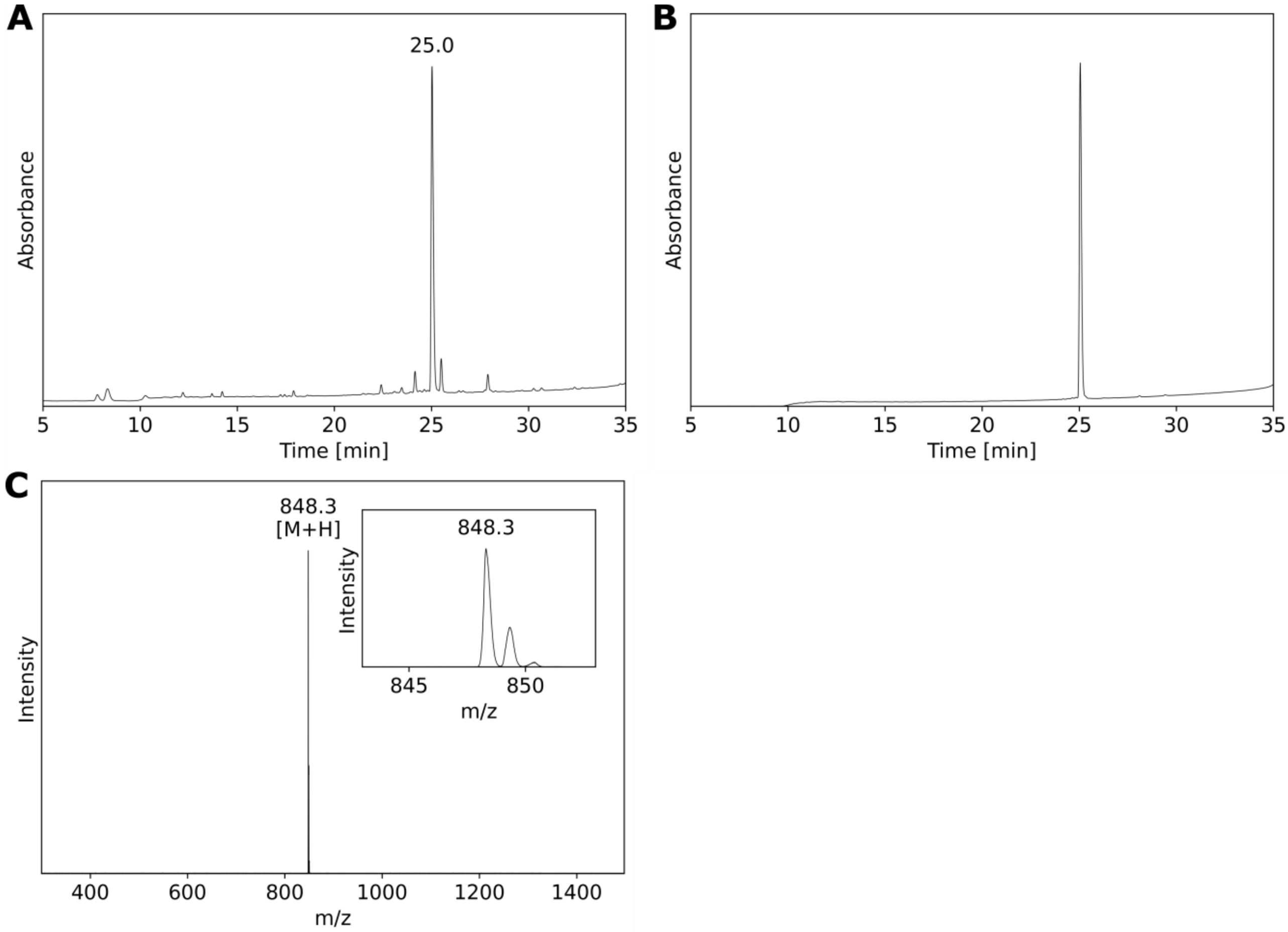

Figure S165: **P6A40Az14** A) Analytical HPLC (220 nm) of crude peptide. HPLC Gradients are listed in Section 2.3.5 Table S3. B) Analytical HPLC (220 nm) of pure peptide. C) MALDI-TOF MS of pure peptide. Calculated mass: $[M+H]^+$ 848.4.

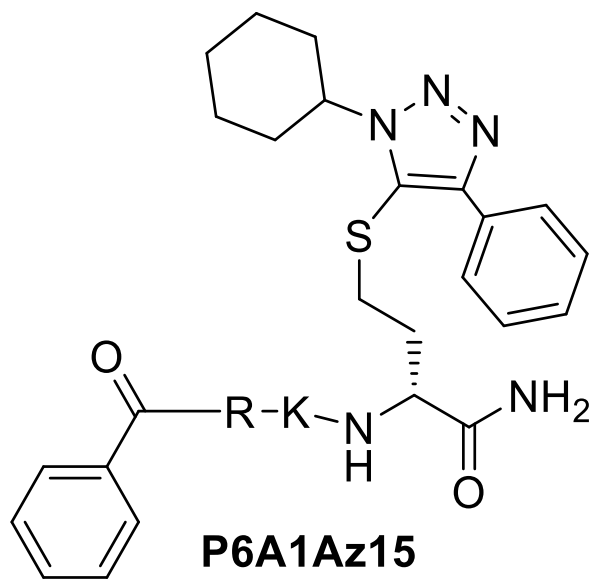

P6A1Az15

Chemical Formula: $C_{37}H_{53}N_{11}O_{4}S$
Exact Mass: 747.40
Molecular Weight: 747.96

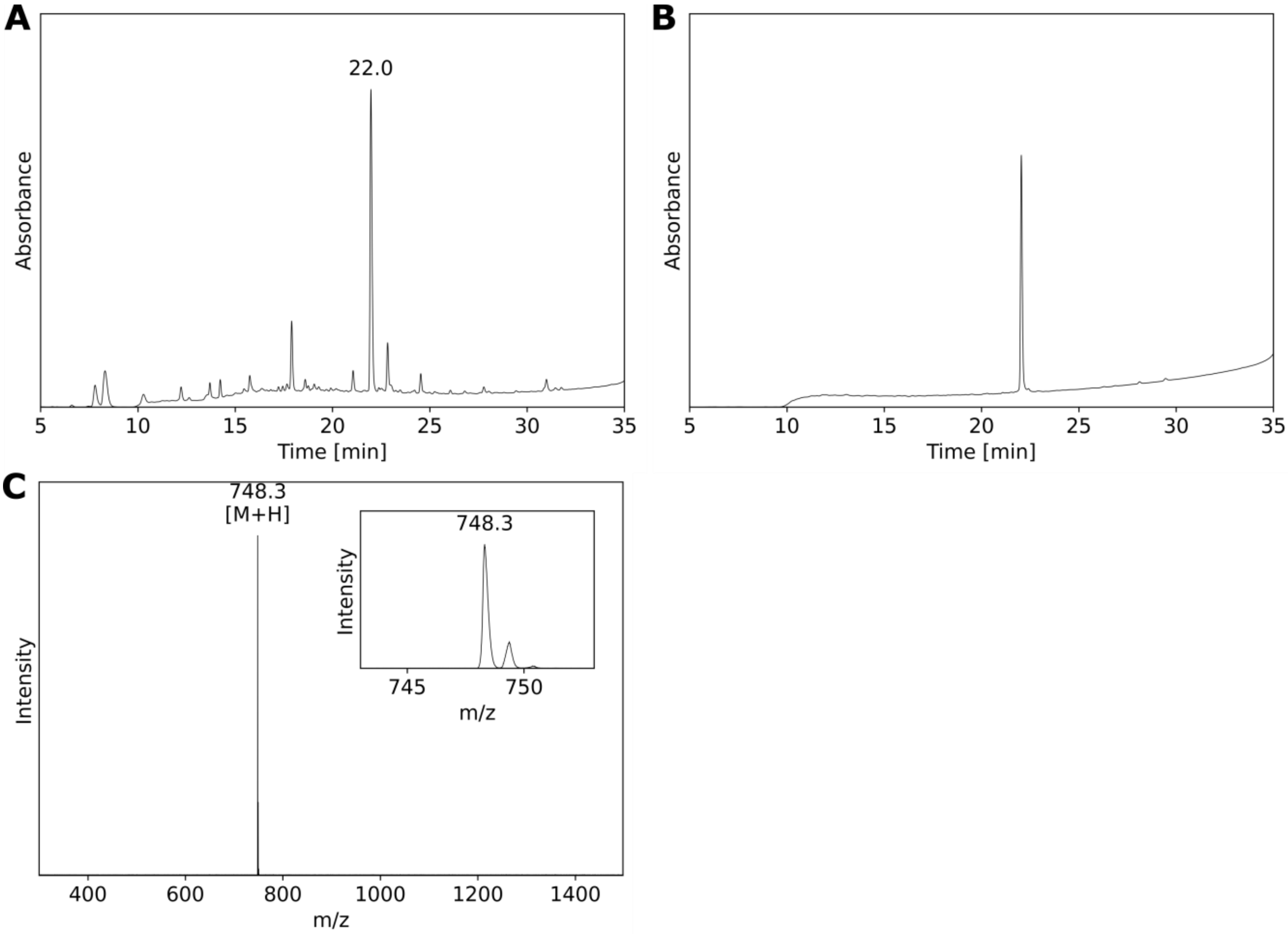

Figure S166: **P6A1Az15** A) Analytical HPLC (220 nm) of crude peptide. HPLC Gradients are listed in Section 2.3.5 Table S3. B) Analytical HPLC (220 nm) of pure peptide. C) MALDI-TOF MS of pure peptide. Calculated mass: $[M+H]^+$ 748.4.

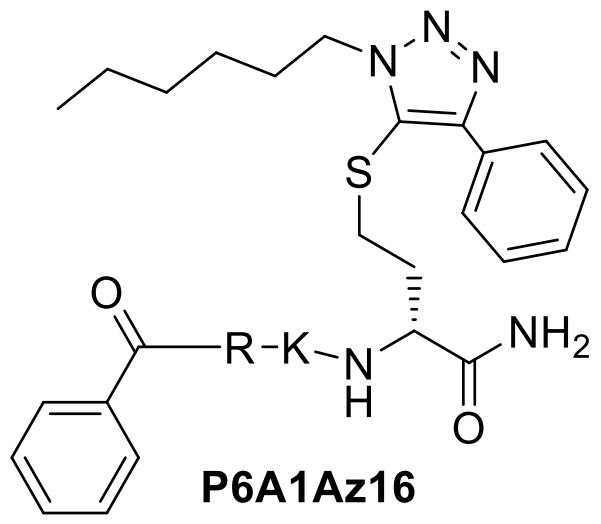

P6A1Az16

Chemical Formula: $C_{37}H_{55}N_{11}O_4S$
Exact Mass: 749.42
Molecular Weight: 749.98

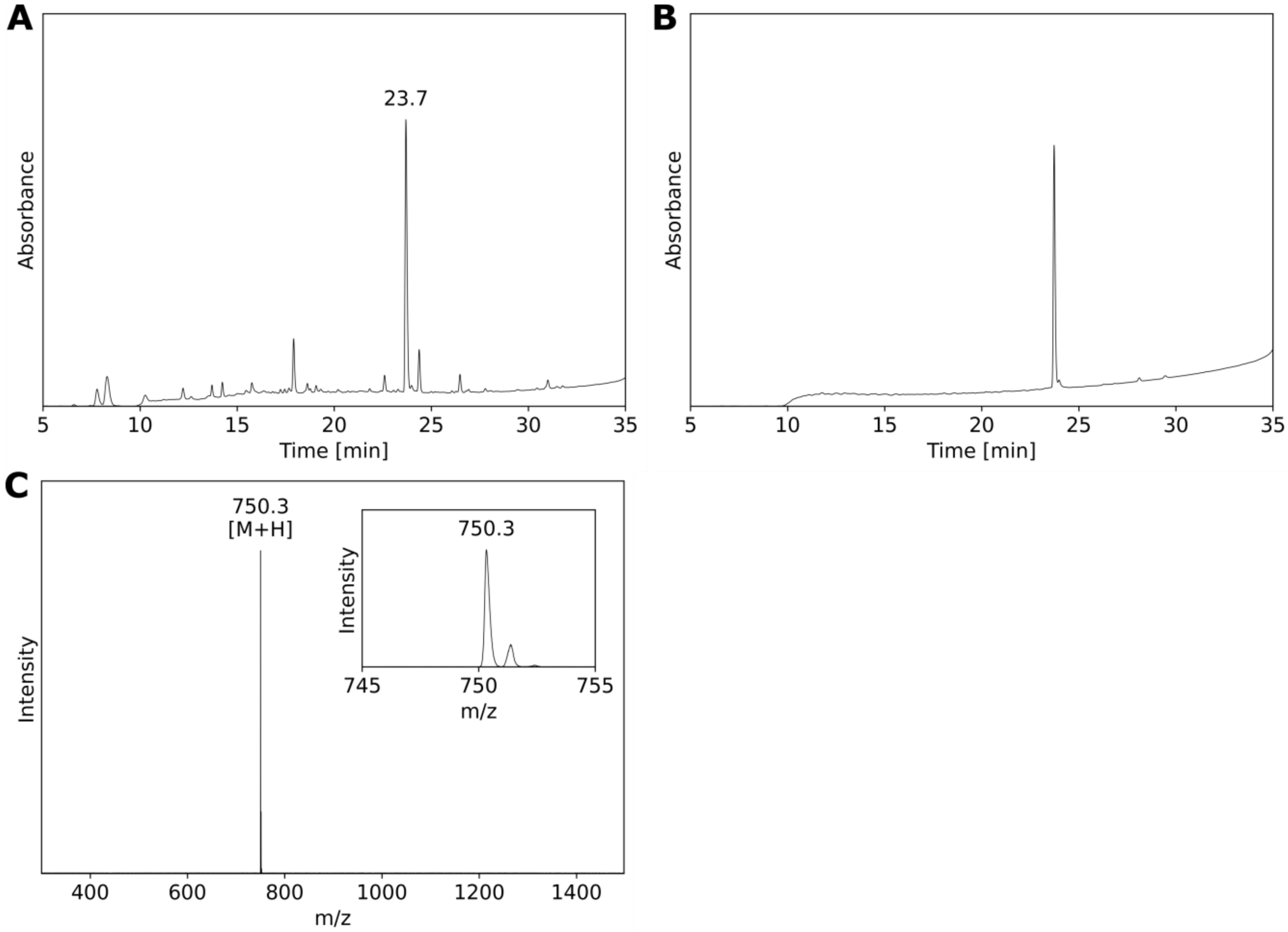

Figure S167: **P6A1Az16** A) Analytical HPLC (220 nm) of crude peptide. HPLC Gradients are listed in Section 2.3.5 Table S3. B) Analytical HPLC (220 nm) of pure peptide. C) MALDI-TOF MS of pure peptide. Calculated mass: $[M+H]^+$ 750.4.

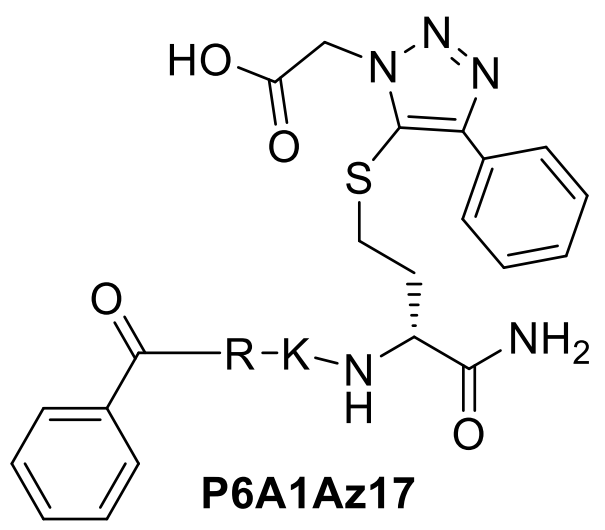

P6A1Az17

Chemical Formula: $C_{33}H_{45}N_{11}O_6S$
Exact Mass: 723.33
Molecular Weight: 723.85

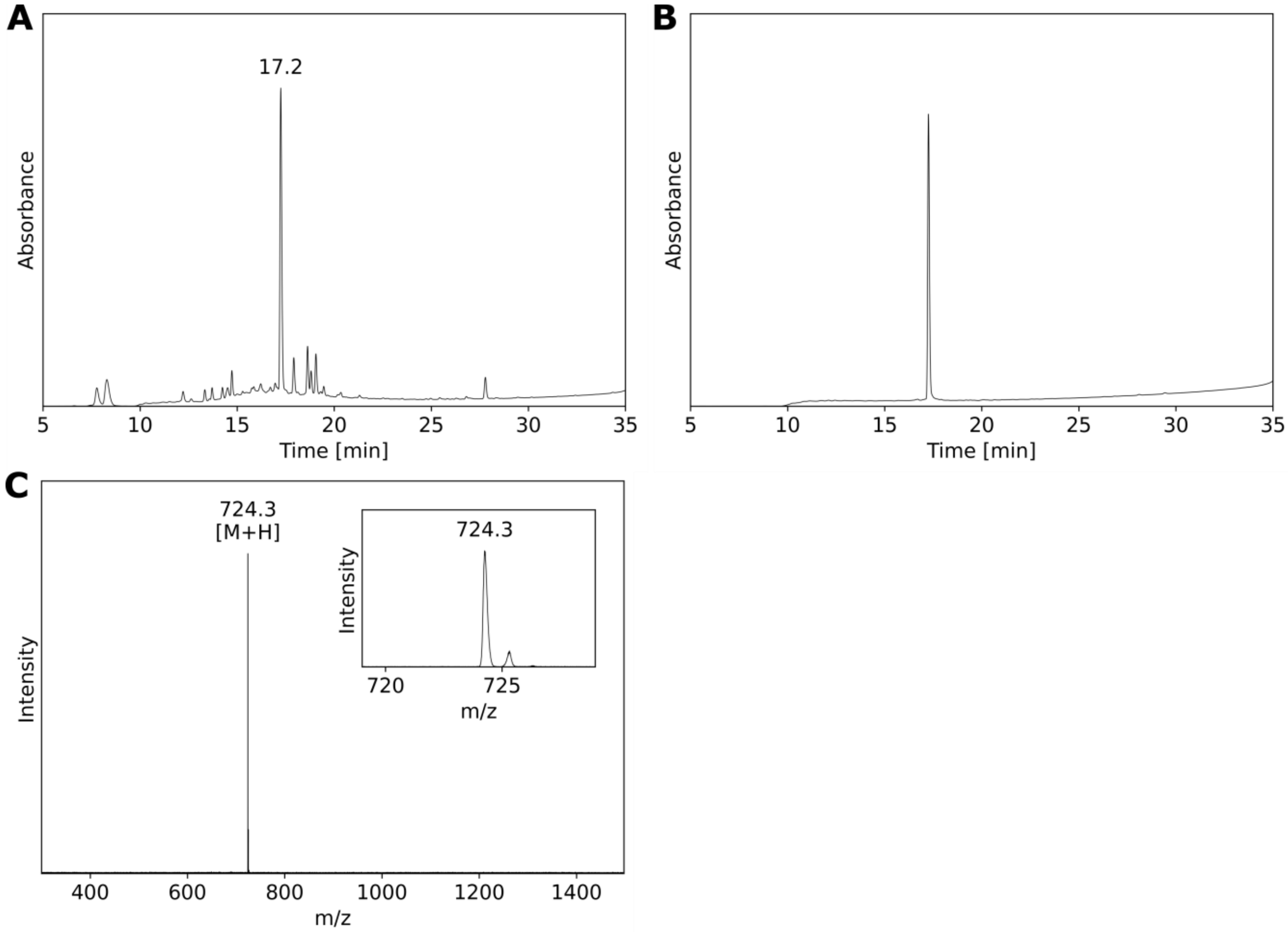

Figure S168: **P6A1Az17** A) Analytical HPLC (220 nm) of crude peptide. HPLC Gradients are listed in Section 2.3.5 Table S3. B) Analytical HPLC (220 nm) of pure peptide. C) MALDI-TOF MS of pure peptide. Calculated mass: $[M+H]^+$ 724.3.

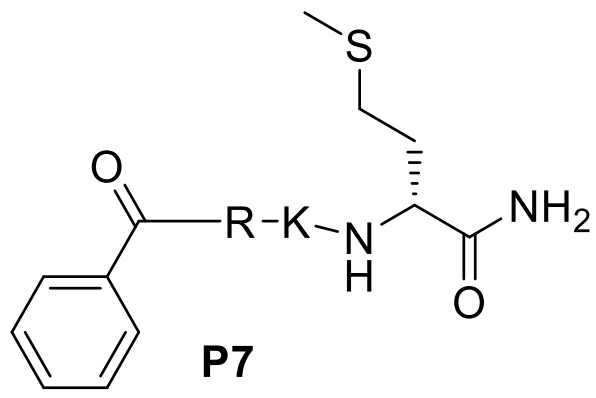

P7

Chemical Formula: $C_{24}H_{40}N_8O_4S$
Exact Mass: 536.29
Molecular Weight: 536.70

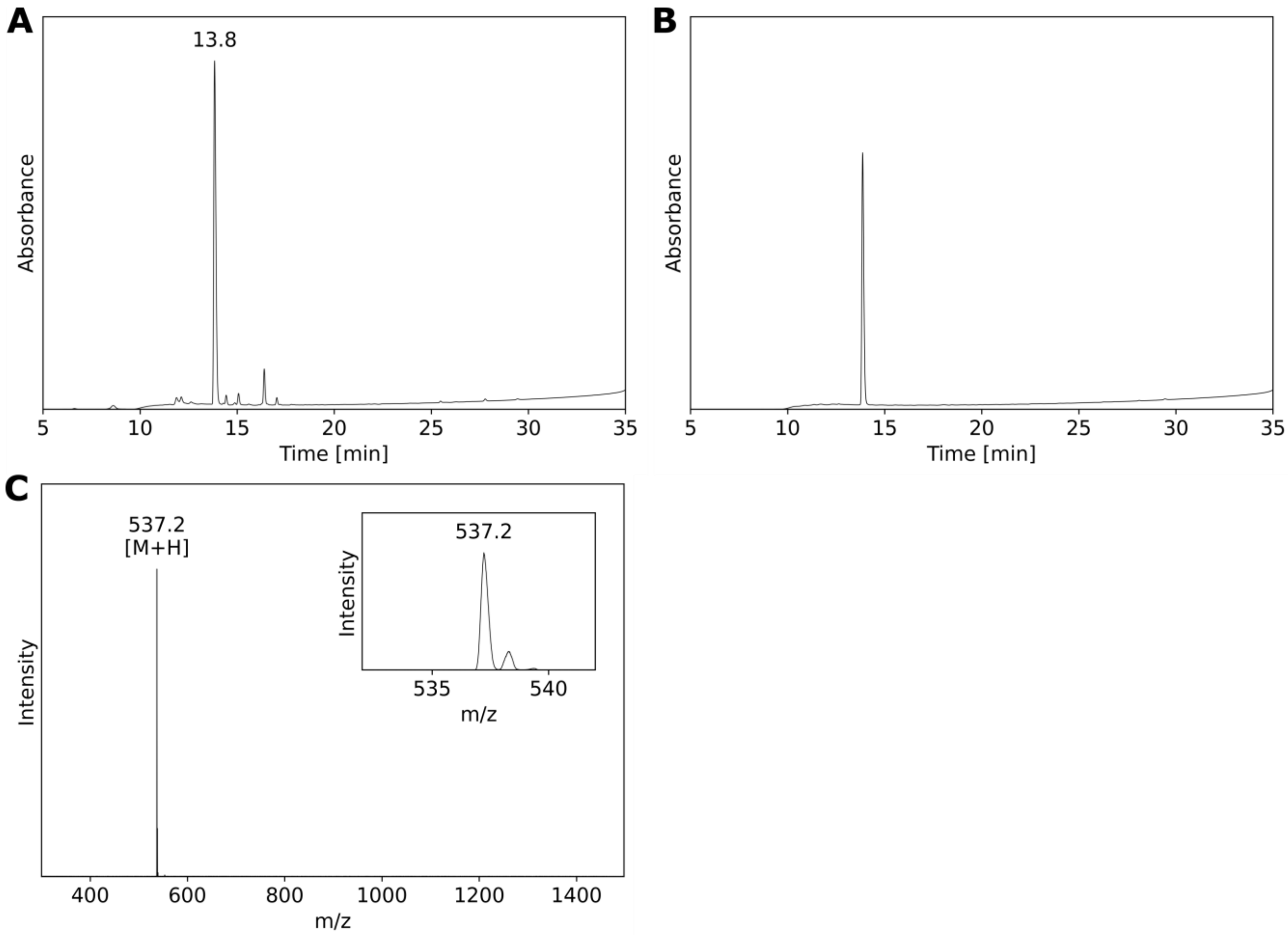

Figure S169: **P7** A) Analytical HPLC (220 nm) of crude peptide. HPLC Gradients are listed in Section 2.3.5 Table S3. B) Analytical HPLC (220 nm) of pure peptide. C) MALDI-TOF MS of pure peptide. Calculated mass: $[M+H]^+$ 537.3.

2 Inhibition of dengue virus protease

For the determining of the inhibitory activity against dengue virus serotype 2 (DENV2) NS2B−NS3 protease, a construct possessing a glycine-serine G_4SG_4 linker, which covalently connects the NS3 protease and NS2B hydrophilic core domain, was used.[2] Expression and purification of the His_6-tagged protein were performed as previously described.[3]

DENV2 protease inhibition assay and IC_{50} determination were performed on a BMG Labtech FLUOstar OMEGA microtiter plate reader, in black 96-well V-bottom plates (Eppendorf, Germany).[4] Proteolytic activity was measured as a continuous assay at room temperature in a 50 mM Hepes, pH 7.5 buffer with 10% ethylene glycol and 1 mM CHAPS as additives. The substrate Bz-nKRR-AMC (50 µM) was used at excitation and emission wavelengths of 355 nm and 460 nm, respectively. The final protease concentration in the assay was 100 nM, and the final assay volume was 100 µL per well. The enzymatic activity was determined as slope per second (relative fluorescence units per second, RFU/s) and monitored for 17 min. Percentage inhibition was calculated relative to a positive control (without the inhibitor). Experiments were performed in triplicate and averaged.

An initial screening of the compounds in Table S1 was performed at a concentration of 25 µM. The compounds in Table S2 were selected for IC_{50} determination. For the IC_{50} plots, the percentage inhibitions at nine concentrations of the compounds **P6A15Az14, P6A40Az14, P6A1Az16,** and **P6A1Az14** were assessed covering the range 0–200 µM, and for compound **P7**, ten concentrations were tested covering the range 0–500 µM. The results were analyzed using GraphPad Prism 8.0.

Table S1: Screening results against DENV2 protease.

Compound	% inhibition at 25 µM
**P6A22Az14**	27.0 ± 1.8
**P6A23Az14**	42.3 ± 0.5
**P6A10Az14**	55.2 ± 1.0
**P6A9Az14**	66.4 ± 3.7
**P6A8Az14**	64.4 ± 2.0
**P6A6Az14**	53.2 ± 4.7
**P6A1Az14**	41.1 ± 1.6
**P6A38Az14**	57.1 ± 2.4
**P6A39Az14**	66.7 ± 0.9
**P7**	7.6 ± 3.5
**P6A40Az14**	68.5 ± 0.9
**P6A34Az14**	47.2 ± 2.4
**P6A35Az14**	46.7 ± 3.6
**P6A15Az14**	74.6 ± 0.5
**P6A1Az15**	51.5 ± 6.0
**P6A1Az16**	58.1 ± 4.3
**P6A1Az17**	26.0 ± 0.8
**P6A1Az2**	37.9 ± 1.4

Table S2: IC_{50} values of selected compounds against DENV2 protease.

Compound	IC_{50} (µM)	Plot
**P6A15Az14**	10.96 ± 1.02	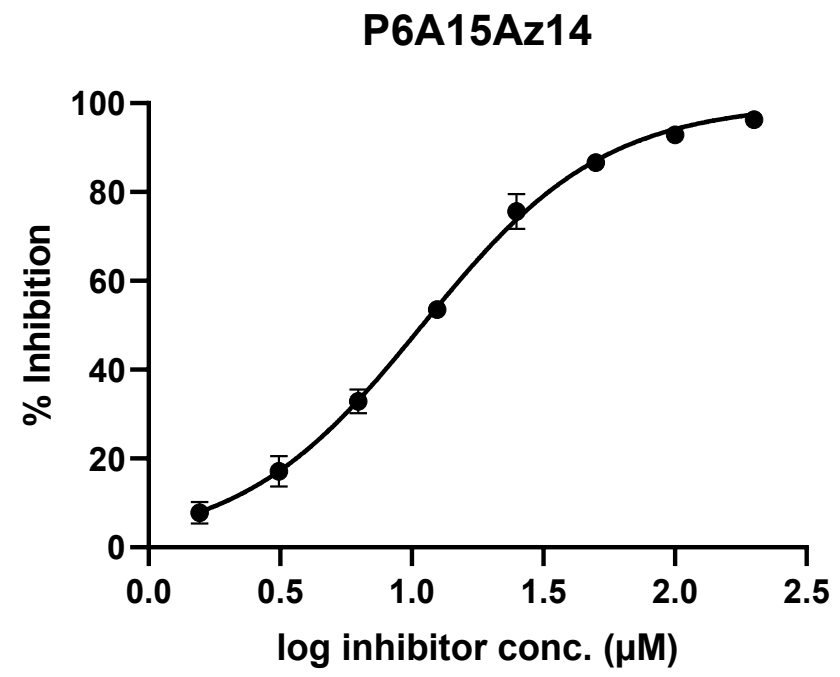
**P6A40Az14**	16.83 ± 1.06	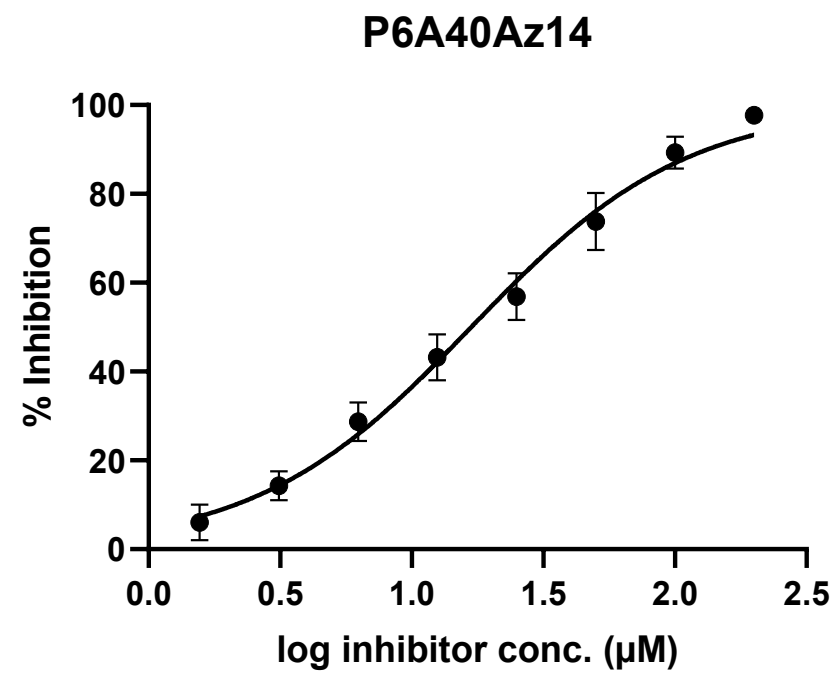
**P6A1Az16**	21.83 ± 1.05	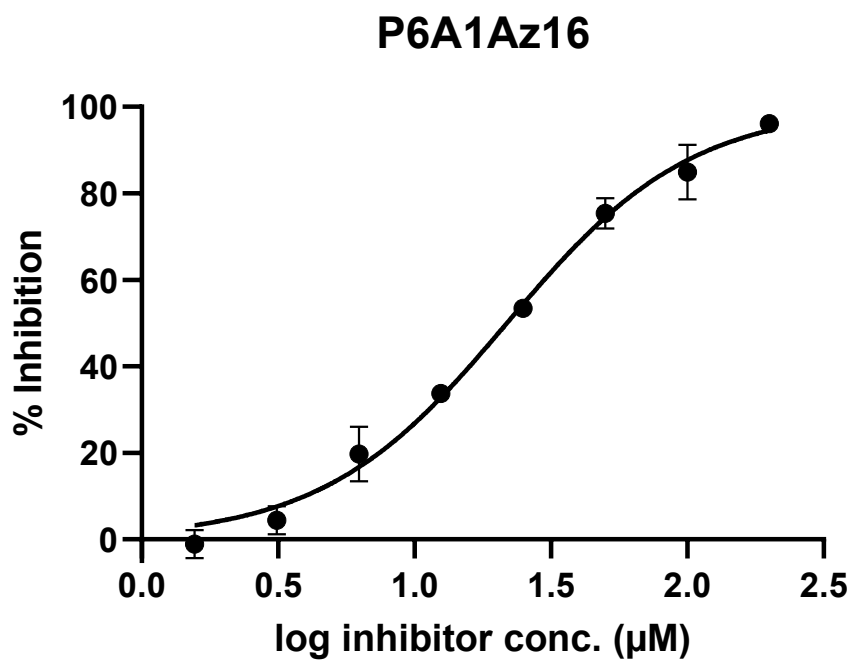
**P6A1Az14**	25.01 ± 1.04	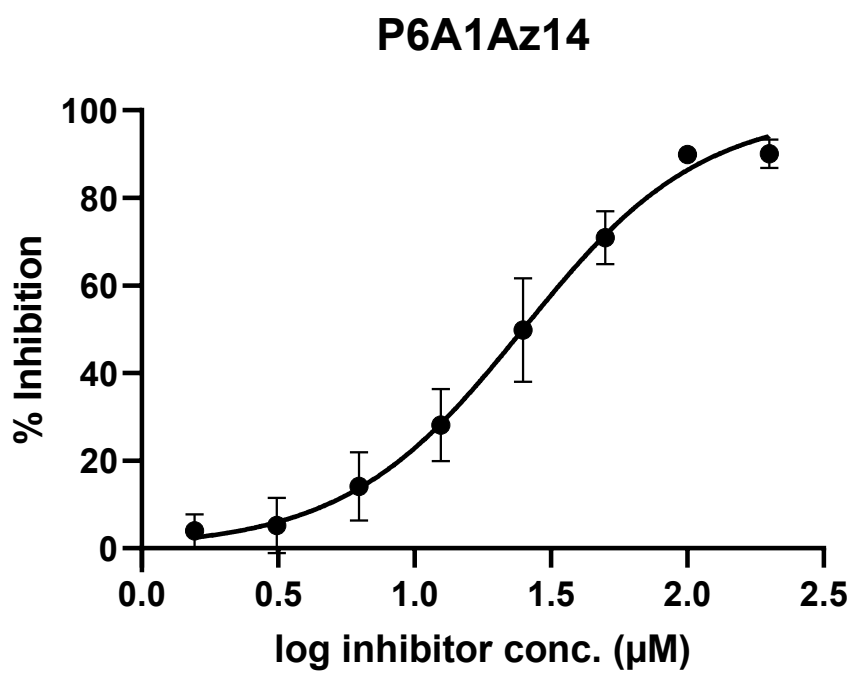
**P7**	210.9 ± 1.05	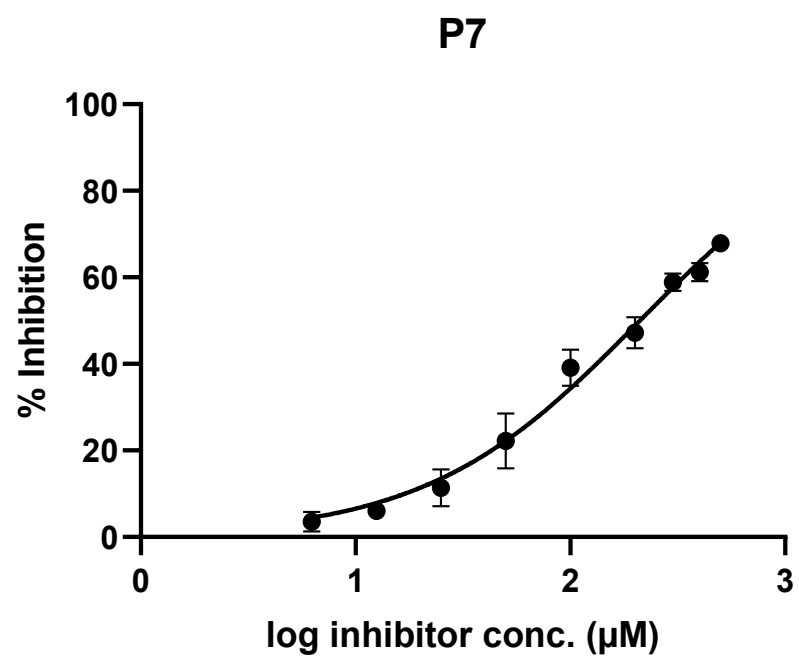

3 Experimental procedures

3.1 Reagents and solvents

Standard Fmoc-protected amino acids, Fmoc-Rink-Amide MBHA polystyrene resin, Oxyma® and *N,N'*-diisopropylcarbodiimide (DIC) were acquired from *Iris Biotech GmbH* (Marktredwitz, Germany). Preloaded Wang resins, Dmt-Cl and Fmoc-Nle-OH were purchased from *Carbolution Chemicals GmbH* (St. Ingebert, Germany). Sodium methanethiosulfonate, Boc-Gly-OH, D-homoserine and Fmoc-Hse-OH were purchased from *BLD Pharmatech GmbH.* (Reinbek, Germany). *N,N'*-diisopropylethylamine (DIPEA), piperidine, MeCN (HPLC grade), trifluoroacetic acid (TFA, HPLC grade) and triisopropyl silane (TIPS) were acquired from *Merck KGaA* (Darmstadt, Germany). DMF (peptide synthesis grade) and formic acid (98+%) was purchased from *Fisher Scientific* (Loughborough, United Kingdom). All other solvents and reagents were at least *reagent* grade quality and were acquired from *TCI Deutschland GmbH* (Eschborn, Germany), *Carbolution Chemicals GmbH* (St. Ingebert, Germany), *BLD Pharmatech GmbH.* (Reinbek, Germany), *Carl Roth GmbH + Co. KG* (Karlsruhe, Germany), *Fisher Scientific* (Loughborough, United Kingdom), *Grüssing GmbH* (Filsum, Germany), *Honeywell* (Seelze, Germany), *Merck KGaA* (Darmstadt, Germany), *neoFroxx GmbH* (Einhausen, Germany), *Th. Geyer GmbH & Co. KG* (Renningen, Germany) and *VWR International* (Fontenay-sous-Bois, France). Reagents and solvents were used as received. Water was purified with a *Sartorius arium® mini* lab water system.

3.2 Software for data analysis and visualization

Unless stated otherwise, data were analyzed and visualized using *Python 3.9* with a *Tkinter 8.*6-based graphical user interface. Data analysis was conducted with the *NumPy 1.24.3*, and *SciPy 1.11.3* modules and data visualization was performed with the *matplotlib 3.8.0* module. Chemical structures were drawn with *ChemDraw 22.2.0*.

3.3 Peptide synthesis, functionalization and purification

3.3.1 Automated solid-phase peptide synthesis

Peptide **P1** was synthesized at 0.25 mmol scale and **P2**, **P3** and **P4** were synthesized at 0.1 mmol scale on a microwave-assisted peptide synthesizer (*CEM Liberty Blue*, see section 2.3.7 for detailed settings). Fmoc-Rink-Amide MBHA polystyrene resin with a loading density of 0.67 mmol/g was used as solid support for **P1** and **P4**. **P2** and **P3** were synthesized with preloaded Fmoc-Lys(Boc)-Wang polystyrene resin (loading density: 0.345 mmol/g) and Fmoc-Arg(Pbf)-Wang polystyrene resin (loading density: 0.322 mmol/g). Prior to synthesis, the resin was swollen in DMF for at least 10 min. Fmoc-protected amino acids were used as 0.2 M solution in DMF. *N,N'*-Diisopropylcarbodiimide (DIC) dissolved in DMF (0.5 M) was used as activator, and Oxyma® solution in DMF (1.0 M) plus 0.1 M *N,N*-diisopropylethylamine (DIPEA) was used as the activator base. A solution of piperidine/DMF 1:4 *v*/*v* was used to remove the Fmoc protecting group. Post synthesis, the peptide was transferred to a 10 mL syringe reactor with a PE frit, washed with DMF (5 x 5 mL) and DCM (10 x 5 mL) and dried under reduced pressure in a desiccator for at least 10 minutes. For *N*-terminal acetylation of peptides **P4** and **P5**, the peptide resin was allowed to swell in DMF (5 mL) for at least 5 minutes and subsequently treated with acetic anhydride (0.5 mL) in pyridine (4.5 mL) for 10 min. The peptide resin was washed with DMF (3 x 5 mL) and DCM (10 x 5 mL) and dried under reduced pressure in a desiccator. The mass of the resin was determined, and it was assumed that this corresponds to 0.25 mmol peptide for **P1** or 0.1 mmol peptide for **P2**, **P3** and **P4**.

3.3.2 Manual solid-phase peptide synthesis

Peptides **P5** and **P6** were synthesized at 0.1 mmol scale, peptide **P7** was at 0.05 mmol scale and peptide **Az10** was synthesized at 0.4 mmol scale by manual solid-phase peptide synthesis in a 20 mL syringe reactor equipped with a PE frit. Fmoc-Rink-Amide MBHA polystyrene resin with a loading density of 0.67 mmol/g was used as solid support. Prior to synthesis, the resin was swollen in DMF for at least 10 min. The standard cycle for peptide synthesis consists of 1) deprotection, 2) wash, 3) coupling and 4) wash (see Step 2).

1) **Deprotection**: To remove the Fmoc protecting group, the resin was treated with a solution of 20% piperidine in DMF (**P5**, **P6**: 5 mL, **P7**: 2.5 mL and **Az10**: 10 mL) and the syringe reactor was shaken for 20 min.
2) **Wash:** The solution was discarded and the resin was washed with DMF (**P5**, **P6**: 3 x 5 mL, **P7**: 3 x 2.5 mL, **Az10**: 3 x 10 mL), DCM (**P5**, **P6**: 3 x 5 mL, **P7**: 3 x 2.5 mL, **Az10**: 3 x 10 mL) and DMF (**P5**, **P6**: 3 x 5 mL, **P7**: 3 x 2.5 mL, **Az10**: 3 x 10 mL).
3) **Coupling:** The protected amino acid or 6-azidohexanoic acid in the last step for **Az10** and benzoic acid in the last step for **P6** and **P7** (**P5**, **P6**: 300 µmol, 3.00 eq., **P7**: 150 µmol, 3.00 eq. and **Az10**: 1.2 mmol, 3.00 eq.) and PyBOP (**P5, P6**: 156 mg, 300 µmol, 3.00 eq., **P7**: 78 mg, 150 µmol, 3.00 eq. and **Az10**: 624 mg, 1.2 mmol, 3.00 eq.) were dissolved in DMF (**P5**, **P6**: 5 mL, **P7**: 2.5 mL and **Az10**: 10 mL), before DIPEA (**P5**, **P6**: 68 µL, 400 µmol, 4.00 eq., **P7**: 34 µL, 200 µmol, 4.00 eq. and **Az10**: 272 µL, 1.6 mmol, 4.00 eq.) was added. After one minute of incubation, the resin was treated with the mixture and the syringe reactor was shaken for one hour.

Acetylation of **P5** was performed as described in the previous chapter. The peptide resin was washed with DMF (**P5**, **P6**: 3 x 5 mL, **P7**: 3 x 2.5 mL and **Az10**: 3 x 10 mL), DCM (**P5**, **P6**: 3 x 5 mL, **P7**: 3 x 2.5 mL and **Az10**: **Az10**: 6 x 10 mL) and dried under reduced pressure in a desiccator. The mass of the resin was determined and it was assumed that this corresponds to 0.1 mmol **P5** and **P6** peptide, 0.05 mmol **P7** peptide and 0.4 mmol **Az10** peptide, respectively.

3.3.3 Cleavage and isolation of peptides

Cleavage of the peptides from the resin and final deprotection were carried out in trifluoroacetic acid (TFA) containing triisopropyl silane (TIPS) and water. Dry resin with 5 µmol peptide was placed into a 2 mL syringe reactor with a PE frit and incubated with a cleavage cocktail consisting of 0.48 mL TFA, 25 µL TIPS and 25 µL water for 3 h, except for **P3**, which was incubated for 4 h. The solution was collected and the resin was washed with TFA (1 x 0.5 mL). The TFA was removed in a stream of nitrogen and the peptide was precipitated from ice-cold Et_2O (1 mL). The peptide was isolated by centrifugation. The pellet was washed with Et_2O (2 x 1 mL) and dissolved in 0.5 mL 20% Buffer B (MeCN + 0.1%) in Buffer A (water + 0.1%) and lyophilized for storage. The volume was increased linearly for larger amounts of peptide.

3.3.4 Post-cleavage sulfoxide reduction

For **P1A21Az12** and **P3A21Az6**, a sulfoxide reduction protocol was applied. The lyophilized peptide was dissolved in 0.5 mL TFA and incubated at 4 °C. Ethane-1.2-dithiol (13 µL, 150 µmol) and trimethylsilyl bromide (10 µL, 75 µmol) were added and the solution was shaken at 4 °C for 100 min. The TFA was removed in a stream of nitrogen and the peptide was precipitated and washed as described above.

3.3.5 Functionalization on solid phase

The standard cycle for peptide functionalization consists of 1) homoserine deprotection, 2) iodination, 3) nucleophilic substitution, 4) S-alkynylation and 5) azide-thioalkyne cycloaddition. In all steps except of Step 1), the reagent solutions were added to the resin inside a nitrogen-flushed desiccator. **P5A35**, **P5A37** and **P5A1Az1** were synthesized at 50 µmol scale and **P5A22Az1** was synthesized at 100 µmol scale with linearly upscaled volumes.

1) **Homoserine Deprotection:** Resin loaded with 50 µmol peptide was placed in a 20 mL syringe reactor with a PE frit. To remove the Dmt protecting group, the resin was washed with 1% TFA in DCM (5 x 10 mL) and DCM (10 x 10 mL). The peptide was directly iodinated afterwards to prevent depsipeptide formation.[5] The resin was not dried and used directly in the next step.
2) **Iodination:** Methyltriphenoxyphosphonium iodide (1.15 g, 2.5 mmol, 50 eq.) was dissolved in dry DMF (5 mL) in a nitrogen-flushed desiccator. The resin was incubated with the iodination reagent for 20 min under gentle shaking. The solution was removed, and the resin was washed with DMF (5 x 10 mL) and DCM (10 x 10 mL) and dried under reduced pressure.
3) **Nucleophilic substitution:** For nucleophilic substitution, sodium methanethiosulfonate (335 mg, 2.5 mmol, 50 eq.) was dissolved in dry DMF (5 mL) in a nitrogen-flushed desiccator. Resin loaded with 50 µmol iodinated peptide was placed in a 10 mL syringe reactor with a PE frit. The nucleophile was added to the resin and the syringe was shaken for 16 h at room temperature. After reaction, the solution

was removed and the resin was washed with DMF (5 x 5 mL) and DCM (5 x 5 mL) and dried under reduced pressure. The volume was decreased linearly for smaller batch sizes. In case of nucleophilic substitution with potassium p-toluenethiosulfonate, resin loaded with 5 µmol peptide in a 2 mL syringe reactor with a PE frit was used.

4) **S-alkynylation:** Resin loaded with 5 µmol methanethiosulfonate-containing peptide was placed in a 2 mL syringe reactor with a PE frit. $Cu(OTf)_2$ (9.0 mg, 25 µmol, 5 eq.), xantphos (14.5 mg, 25 µmol, 5 eq.), Cs_2CO_3 (32.6 mg, 100 µmol, 20 eq.) and, if solid, alkyne (250 µmol, 50 eq.) were added to the resin and dry DMSO (0.5 mL) was taken up in the syringe. Liquid alkynes were added to DMSO instead. The syringe was shaken for 16 h at room temperature. After reaction, the solution was removed and the resin was washed with DMSO (3 x 1 mL), water (3 x 1 mL), MeOH (3 x 1 mL), DMF (3 x 1 mL) and DCM (3 x 1 mL) and dried under reduced pressure. Cleavage and final deprotection for peptide thioester synthesis was performed as described above. In case of **A13**, 4-ethinylbenzaldehyde (250 µmol, 50 eq.) was dissolved in dry DMSO (0.5 mL) and propylamine (300 µmol, 60 eq.) was added. The mixture was heated at 40 °C for 3 hours in a thermoshaker. The yellow solution was added to the resin loaded with 5 µmol methanethiosulfonate-containing peptide placed in a 2 mL syringe reactor with a PE frit. $Cu(OTf)_2$ (9.0 mg, 25 µmol, 5 eq.), xantphos (14.5 mg, 25 µmol, 5 eq.) and Cs_2CO_3 (32.6 mg, 100 µmol, 20 eq.). S-alkynylation and work-up was performed as described above.

5) **Thioalkyne-azide cycloaddition:** Resin loaded with 5 µmol S-alkynylated peptide was placed in a 4 mL borosilicate reaction vial equipped with a polypropylene cap and a TFE septum (Chemglass, CG-4904-5). In case of **P5**, a 5 mL centrifuge tube was used instead. $[Ir(cod)Cl]_2$ (3.4 mg, 5 µmol, 2 eq.) was added followed by dry DMF (0.5 mL) and azide (50 µmol, 10 eq.). The reaction vial was equilibrated for 2 h at room temperature. After reaction, the resin was transferred to a 2 mL syringe reactor with a PE frit and was washed with DMF (5 x 1 mL) and DCM (5 x 1 mL) and dried under reduced pressure. Reactions from Table 3 Entry 1,2 and 3 were carried out similarly with adjusted amounts of catalyst and azide and with the respective solvents and reaction times. Cleavage and final deprotection was performed as described above. For **P2** and **P3**, the conditions were adjusted by adding $[Ir(cod)Cl]_2$ (13.6 mg, 20 µmol, 8 eq.), followed by dry DMF (0.5 mL) and azide (125 µmol, 25 eq.) and letting the mixture shake for 16 h instead.

3.3.6 High-performance liquid chromatography (HPLC)

The peptides were purified by reversed-phase HPLC on a *VDS optilab VDSpher® PUR 100 C18-SE* (250 mm x 10 mm, 100 Å, 5 µm) column at a flow rate of 3 mL/min at 50 °C. A *Jasco* chromatography system with a *PU-4180* pump, a *CO-4060* column thermostat and a *UV-4070* detector was used. Analytical HPLC was performed using a *Hitachi Primaide* chromatography system containing a *1110 Pump*, a *1210 auto sampler*, *1310 column oven* and a *1430 diode array detector*. A *VDS optilab VDSpher® PUR 100 C18-SE* (250 mm x 4.6 mm, 100 Å, 5 µm) column and a flow rate of 1 mL/min at 50 °C were used. Preparative chromatograms were monitored at 220 nm and 280 nm. Analytical chromatograms were monitored from 190 to 400 nm. Fractions containing pure peptide were identified by analytical HPLC and MALDI-TOF MS, pooled and lyophilized. As solvent system, water + 0.1% TFA (Buffer A) and MeCN + 0.1% TFA (Buffer B) was used. The gradients are displayed in **Fehler! Verweisquelle konnte nicht gefunden werden.**. Prior to injection, peptide solutions were filtered with H-PTFE syringe filters (0.22 µm pore size) from *VWR*.

Table S3: Gradients used for the purification and characterization of peptides **P1** to **P5** and **Az10**. The percentages of Buffer B in Buffer A (*v*/*v*) are given.

**preparative**	**Isocratic 1**	**linear gradient**	**Isocratic 2**	**Isocratic 3**
**time / min**	0 → 8	8 → 38	39 → 44	45 → 50
**P1 (except of P1A14, P1A36, P1A21Az5, P1A21Az9, P1A21A11) P3, P5 (except of P5A37) and Az10**	10%	10 – 40%	100%	10%
**P1A14, P1A36, P1A21Az5, P1A21Az9, P1A21A11, P2, P5A37, P6 and P7**	10%	10 – 80%	100%	10%
**P4**	20%	20 – 45%	100%	20%
**analytical**				
**time / min**	0 → 5	5 → 35	36 → 41	42 → 50

**P1 (except of P1A14, P1A36, P1A21Az5, P1A21Az9, P1A21A11) P3, P5 (except of P5A37) and Az10**	10%	10 – 40%	100%	10%
**P1A14, P1A36, P1A21Az5, P1A21Az9, P1A21A11, P2, P5A37, P6 and P7**	10%	10 – 80%	100%	10%
**P4**	20%	20 – 45%	100%	20%

3.3.7 Mass spectrometry

Electrospray ionization (ESI) was performed on an ion cyclotron resonance *Bruker microTOF-Q II*. Matrix-assisted laser desorption ionization time-of-flight mass spectrometry (MALDI-TOF MS) was performed on a *Bruker Autoflex Speed*. 1 µl of the matrix 2,5-dihydroxybenzoic acid (20 mg/mL in 3:7 MeCN/water + 0.1% TFA) was mixed with 1 µl of the sample, pipetted onto the target plate and air dried.

3.3.8 Settings of the peptide synthesizer

The following Tables (S4-S14) contain the detailed settings for the microwave-assisted peptide syntheses at 0.1 mmol scale performed in this study using the *CEM LibertyBlue* peptide synthesizer.

Table S4: Settings for **Microwave Methods.** Mixing of the reaction mixture was performed by bubbling nitrogen through the reaction vessel frit (bubble for 2 s, off for 3 s).

Microwave Method	Temperature / °C	Power / W	Hold Time / s	DeltaT / °C
Standard deprotection	75	155	15	2
	90	30	50	1
Coupling	75	170	15	2
	90	30	110	1
50 °C 10 min Coupling	25	0	120	2
	50	35	480	1

Table S5: Settings for **Resin Swelling**.

	Cycle Steps	Parameter values
1	Swell Resin	Main solvent volume: 15 mL, Time: 300 s

Table S6: Settings for **Single Coupling**.

	Cycle Steps	Parameter values
1	Deprotection	Reaction Method: Standard Deprotection, Deprotection Volume: 4 mL (10 mL for 0.25 mmol scale)
2	Wash	Volume: 4 mL (7 mL for 0.25 mmol scale), Drain Time: 10 s
3	Wash	Volume: 4 mL (7 mL for 0.25 mmol scale), Drain Time: 5 s
4	Wash	Volume: 4 mL (7 mL for 0.25 mmol scale), Drain Time: 5 s
5	Wash	Volume: 4 mL (7 mL for 0.25 mmol scale), Drain Time: 5 s
6	Coupling	Reaction Method: Standard coupling, Amino Acid: from method, Amino Acid Volume: 2.5 mL (5 mL for 0.25 mmol scale), Activator Bottle Position: PositionACT, Activator Volume: 2 mL, Activator Base Position: PositionACTB, Activator Base Volume: 1 mL, Delayed Reagent Time: 0 s, Delayed Reagent Bottle Position: PositionACTB, Delayed Reagent Volume: 0 mL, Manifold Wash Volume: 2 mL
7	Wash	Volume: 4 mL (7 mL for 0.25 mmol scale), Drain Time: 10 s
8	Wash	Volume: 4 mL (7 mL for 0.25 mmol scale), Drain Time: 5 s

Table S7. Settings for **Single 50 °C Coupling**.

	Cycle Steps	Parameter values
1	Deprotection	Reaction Method: Standard Deprotection, Deprotection Volume: 4 mL
2	Wash	Volume: 4 mL, Drain Time: 10 s
3	Wash	Volume: 4 mL, Drain Time: 5 s
4	Wash	Volume: 4 mL, Drain Time: 5 s
5	Wash	Volume: 4 mL, Drain Time: 5 s
6	Coupling	Reaction Method: 50 °C 10 min coupling, Amino Acid: from method, Amino Acid Volume: 2.5 mL, Activator Bottle Position: PositionACT, Activator Volume: 2 mL, Activator Base Position: PositionACTB, Activator Base Volume: 1 mL, Delayed Reagent Time: 0 s, Delayed Reagent Bottle Position: PositionACTB, Delayed Reagent Volume: 0 mL, Manifold Wash Volume: 2 mL
7	Wash	Volume: 4 mL, Drain Time: 10 s
8	Wash	Volume: 4 mL, Drain Time: 5 s

Table S8. Settings for **First Single 50 °C Coupling**.

	Cycle Steps	Parameter values
1	Deprotection	Reaction Method: Standard Deprotection, Deprotection Volume: 4 mL
2	Deprotection	Reaction Method: Standard Deprotection, Deprotection Volume: 4 mL
3	Wash	Volume: 4 mL, Drain Time: 10 s
4	Wash	Volume: 4 mL, Drain Time: 5 s
5	Wash	Volume: 4 mL, Drain Time: 5 s
6	Wash	Volume: 4 mL, Drain Time: 5 s
7	Coupling	Reaction Method: 50 °C 10 min coupling, Amino Acid: from method, Amino Acid Volume: 2.5 mL, Activator Bottle Position: PositionACT, Activator Volume: 2 mL, Activator Base Position: PositionACTB, Activator Base Volume: 1 mL, Delayed Reagent Time: 0 s, Delayed Reagent Bottle Position: PositionACTB, Delayed Reagent Volume: 0 mL, Manifold Wash Volume: 2 mL
8	Wash	Volume: 4 mL, Drain Time: 10 s
9	Wash	Volume: 4 mL, Drain Time: 5 s

Table S9: Settings for **Double Coupling**.

	Cycle Steps	Parameter values
1	Deprotection	Reaction Method: Standard Deprotection, Deprotection Volume: 4 mL
2	Wash	Volume: 4 mL, Drain Time: 10 s
3	Wash	Volume: 4 mL, Drain Time: 5 s
4	Wash	Volume: 4 mL, Drain Time: 5 s
5	Wash	Volume: 4 mL, Drain Time: 5 s
6	Coupling	Reaction Method: Standard coupling, Amino Acid: from method, Amino Acid Volume: 2.5 mL (5 mL for 0.25 mmol scale), Activator Bottle Position: PositionACT, Activator Volume: 2 mL, Activator Base Position: PositionACTB, Activator Base Volume: 1 mL, Delayed Reagent Time: 0 s, Delayed Reagent Bottle Position: PositionACTB, Delayed Reagent Volume: 0 mL, Manifold Wash Volume: 2 mL
7	Wash	Volume: 4 mL, Drain Time: 5 s
8	Coupling	see step 6
9	Wash	Volume: 4 mL, Drain Time: 10 s
10	Wash	Volume: 4 mL, Drain Time: 5 s

Table S10: Settings for **Double Coupling wash**.

	Cycle Steps	Parameter values
1	Deprotection	Reaction Method: Standard Deprotection, Deprotection Volume: 4 mL
2	Wash	Volume: 4 mL, Drain Time: 10 s
3	Wash	Volume: 4 mL, Drain Time: 5 s
4	Wash	Volume: 4 mL, Drain Time: 5 s
5	Wash	Volume: 4 mL, Drain Time: 5 s
6	Wash	Volume: 4 mL, Drain Time: 5 s
7	Wash	Volume: 4 mL, Drain Time: 10 s
8	Coupling	Reaction Method: Standard coupling, Amino Acid: from method, Amino Acid Volume: 2.5 mL, Activator Bottle Position: Position ACT, Activator Volume: 2 mL, Activator Base Position: Position ACTB, Activator Base Volume: 1 mL, Delayed Reagent Time: 0 s, Delayed Reagent Bottle Position: PositionACTB, Delayed Reagent Volume: 0 mL, Manifold Wash Volume: 2 mL
9	Wash	Volume: 4 mL, Drain Time: 10 s
10	Wash	Volume: 4 mL, Drain Time: 10 s
11	Coupling	see step 8
12	Wash	Volume: 4 mL, Drain Time: 10 s
13	Wash	Volume: 4 mL, Drain Time: 5 s
14	Wash	Volume: 4 mL, Drain Time: 5 s
15	Wash	Volume: 4 mL, Drain Time: 5 s

Table S11: Settings for **Final Deprotection**.

	Cycle Steps	Parameter values
1	Deprotection	Reaction Method: Standard Deprotection, Deprotection Volume: 4 mL
2	Wash	Volume: 4 mL, Drain Time: 5 s
3	Wash	Volume: 4 mL, Drain Time: 5 s
4	Wash	Volume: 4 mL, Drain Time: 5 s
5	Wash	Volume: 4 mL, Drain Time: 5 s

Table S12: Settings for the synthesis of **P1**.

	Step	Used Cycles (optimized conditions)
**1**	Resin swelling	Resin swelling
**2**	E	Single Coupling
**3**	L	Single Coupling
**4**	R	Double Coupling
**5**	Q	Single Coupling
**6**	A	Single Coupling
**7**	I	Single Coupling
**8**	E	Single Coupling
**9**	A	Single Coupling
**10**	K	Single Coupling
**11**	Hse	Single Coupling
**12**	P	Single Coupling
**13**	Boc-Gly	Double Coupling

Table S13: Settings for the synthesis of **P2**.

	Step	Used Cycles (optimized conditions)
**1**	Resin swelling	Resin swelling
**2**	E	Single Coupling
**3**	N	Single Coupling
**4**	S	Single Coupling
**5**	I	Single Coupling
**6**	I	Single Coupling
**7**	S	Single Coupling
**8**	P	Single Coupling
**9**	L	Single Coupling
**10**	I	Single Coupling
**11**	Hse	Single Coupling
**12**	F	Single Coupling
**13**	A	Single Coupling
**14**	S	Single Coupling
**15**	Boc-Lys(Boc)	Single Coupling

Table S14: Settings for the synthesis of **P3**.

	Step	Used Cycles (optimized conditions)
**1**	Resin swelling	Resin swelling
**2**	H	Single 50 °C Coupling
**3**	P	Single Coupling
**4**	K	Single Coupling
**5**	K	Single Coupling
**6**	V	Single Coupling
**7**	G	Double Coupling wash
**8**	G	Double Coupling wash
**9**	T	Single Coupling
**10**	S	Single Coupling
**11**	P	Single Coupling
**12**	A	Single Coupling
**13**	S	Single Coupling
**14**	M	Single Coupling
**15**	R	Double Coupling
**16**	A	Single Coupling
**17**	A	Single Coupling
**18**	K	Single Coupling
**19**	T	Single Coupling
**20**	A	Single Coupling
**21**	Hse	Single Coupling
**22**	Q	Single Coupling
**23**	K	Single Coupling
**24**	R	Double Coupling
**25**	P	Single Coupling
**26**	A	Single Coupling
**27**	Boc-Lys(Boc)	Single Coupling

Table S15: Settings for the synthesis of **P4**.

	Step	Used Cycles (optimized conditions)
**1**	Resin swelling	Resin swelling
**2**	G	Double Coupling wash
**3**	S	Single Coupling
**4**	P	Single Coupling
**5**	R	Double Coupling
**6**	E	Single Coupling
**7**	Hse	Single Coupling
**8**	Q	Single Coupling
**9**	S	Single Coupling
**10**	A	Single Coupling
**11**	N	Single Coupling
**12**	T	Single Coupling
**13**	I	Single Coupling
**14**	H	Single 50 °C Coupling
**15**	N	Single Coupling
**16**	F	Single Coupling
**17**	Y	Single Coupling
**18**	Y	Single Coupling
**19**	V	Single Coupling
**20**	R	Double Coupling
**21**	G	Double Coupling wash
**22**	S	Single Coupling
**23**	S	Single Coupling
**24**	R	Double Coupling
**25**	S	Single Coupling
**26**	Nle	Single Coupling
**27**	R	Double Coupling
**28**	K	Single Coupling
**29**	E	Single Coupling
**30**	W	Single Coupling
**31**	G	Double Coupling wash
**32**	P	Single Coupling
**33**	P	Single Coupling
**34**	L	Single Coupling
**35**	K	Single Coupling
**36**	Final deprotection	Final Deprotection

3.4 NMR spectroscopy

Nuclear magnetic resonance (NMR) spectroscopy was performed on a *Bruker Avance III 400* spectrometer at 400 MHz for Fmoc-Hse-OH, Fmoc-Hse(Dmt)-OH and **P5A1Az1** and on a *Bruker Avance Neo 700* spectrometer at 700 MHz for **P1** peptides, **P5A35**, **P5A37**, **P5A20Az1_F1** and **P5A20Az1_F2**. **P1** peptides were measured at a concentration around 0.2 – 2.5 mM, except of **P1A1** that was measured at a concentration of 10 mM. Due to the low peptide concentration, peaks from minor impurities that may have been introduced during sample preparation through the use of plastic syringes and disposable cannulas with a silicone coating, may be visible in the spectra, appearing at approximately 5.3 ppm and 0.1 ppm. Prior to measurement, **P1** peptides were incubated in MeOH-d_4 to exchange the amide backbone protons, except for **P1A1** and **P1A13**. The measured ^{1}H-NMR spectra were referenced to the residual protons of the deuterated solvent (δ = 2.50 ppm for DMSO-d_6 for **P5** peptides and δ = 3.31 ppm for MeOH-d_4 for **P1** peptides).
Spectra processing and analysis was performed using automatic phase correction and automatic baseline correction with *MestReNova 15.0.0*. Signal multiplicities were abbreviated with s (singlet), d (doublet), t (triplet), q (quartet) and m (multiplet).

3.5 Thin layer and column chromatography

Thin layer chromatography (TLC) was performed to monitor the reaction. *Merck TLC Silica gel 60 F_{254}* on aluminium foil was used. The spots were visualized with UV-light (254 nm) and by staining with diluted sulfuric acid.
Column chromatography was performed with *Merck Silica gel 60* (0.040 to 0.063 mm). The collected fractions were analysed with TLC.

4 Organic synthesis

4.1 Fmoc-Hse(Dmt)-OH

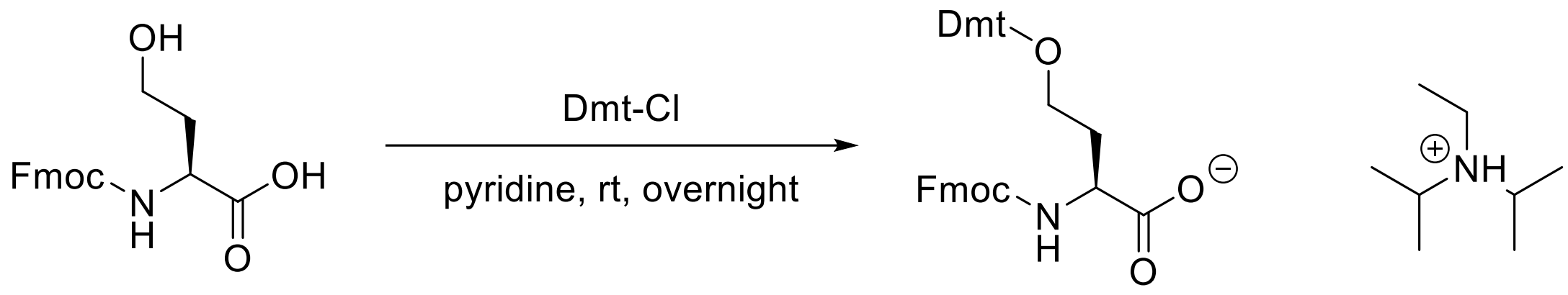

Fmoc-Hse-OH **Fmoc-Hse(Dmt)-OH**

Fmoc-Hse-OH (2.00 g, 5.96 mmol, 1.00 eq.) was dissolved in anhydrous pyridine (30 mL) in an N_2 atmosphere. Dmt-Cl (4.04 g, 11.9 mmol, 2.00 eq.) was added in four portions in 30 min intervals and the mixture was stirred overnight at rt. The solvent was evaporated under reduced pressure. The residue was dissolved in EtOAc (250 mL), washed with water (3 x 150 mL) and brine (150 mL) and dried over Na_2SO_4. After filtration, the solvent was evaporated under reduced pressure. The viscous yellow oil was kept at −21 °C. The crude product was purified by column chromatography (silica gel, DCM:MeOH:DIPEA (95:4:1)). Product fractions were identified by TLC and the solvent was evaporated under reduced pressure. The remaining oil was dried in vacuum to yield a colourless foam.

Yield: 4.12 g, 5.33 mmol, 86% (as DIPEA salt).

TLC: R_f (DCM/MeOH/DIPEA 95:4:1) = 0.21.

^{1}H NMR (400 MHz, DMSO-d$_6$) δ = 7.89 (d, 2H, J = 7.5 Hz, H$_{ar}$), 7.67 (t, 2H, J = 6.5 Hz, H$_{ar}$), 7.41-7.17 (m, 14H, H$_{ar}$), 6.86-6.82 (m, 4H, H$_{ar}$), 4.25 (d, 2H, J = 5.9 Hz, CH-C**H**$_2$ Fmoc), 4.20-4.15 (m, 1H, C**H**-CH$_2$ Fmoc), 4.12-4.05 (m, 1H, α-CH), 3.68 (s, 1H, *O*-CH$_3$), 3.67 (s, 1H, *O*-CH$_3$), 3.16-3.03 (m, 5H, C**H**-(CH$_3$)$_2$ DIPEA, γ-CH$_2$), 2.99-2.97 (m, 1H, γ-CH$_2$), 2.56 (q, 4H, J = 7.6 Hz, C**H**$_2$-CH$_3$ DIPEA), 2.08-2.04 (m, 1H, β-CH$_2$), 1.86-1.81 (m, 1H, β-CH$_2$), 1.02-0.98 (m, 24H, CH$_3$ DIPEA) ppm.

The analytical data are identical to the literature.[5]

4.2 Fmoc-D-Hse-OH

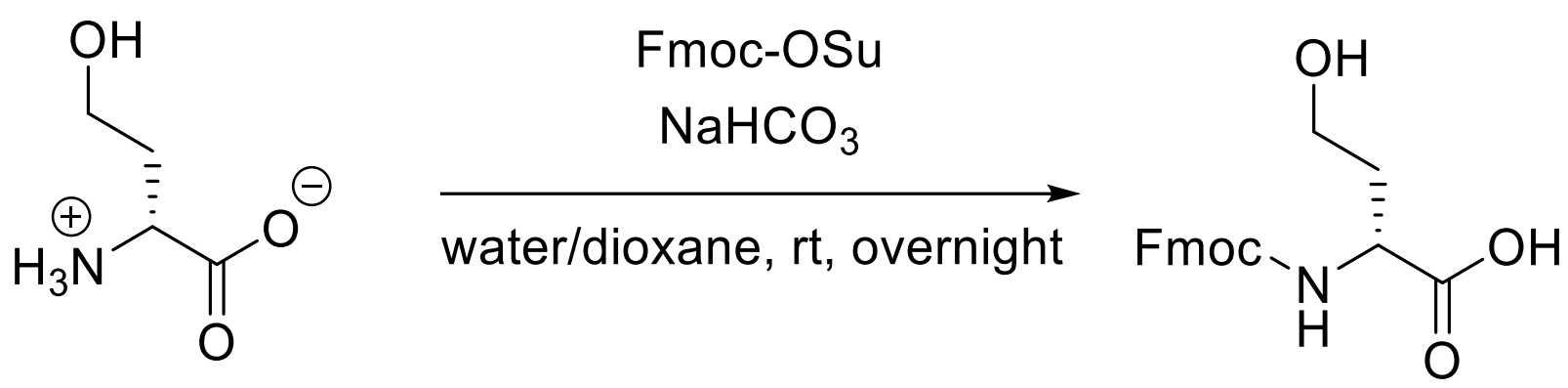

H-D-Hse-OH **Fmoc-D-Hse-OH**

D-Homoserine (2.00 g, 16.8 mmol, 1.00 eq.), Fmoc-OSu (5.66 g, 16.8 mmol, 1.00 eq.) and $NaHCO_3$ (2.75 g, 32.7 mmol, 1.95 eq.) were dissolved in water (55 mL) and dioxane (65 mL). The solution was stirred overnight at rt. Dioxane was removed under reduced pressure. The mixture was transferred into a separating funnel with water (200 mL), 5% $NaHCO_3$ in water (150 mL) and Et_2O (150 mL). After removing of the organic phase, the aqueous phase was washed with diethyl ether (3 x 200 mL). The organic phases were discarded. Citric acid (50 g) was added to the aqueous phase. The aqueous phase was extracted with EtOAc (3 x 150 mL). The combined EtOAc phases were washed with brine (100 mL), dried over Na_2SO_4 and filtered. The solvent was removed under reduced pressure to give an oil, which was dissolved in EtOAc (25 mL). Crystallisation occurred overnight at −20 °C. The colourless solid was filtered and washed with ice-cold EtOAc. The product was dried in a desiccator under reduced pressure.

Yield: 4.56 g, 13.4 mmol, 80%.

ESI-MS: $[M+Na]^+$ 364.1162 (calculated: 264.1155)

^{1}H NMR (400 MHz, DMSO-d_6) δ = 12.54 (s, 1H, COOH), 7.89 (d, 2H, J = 7.5 Hz, H_{ar}), 7.73 (dd, 2H, J = 7.4, 1.1 Hz, H_{ar}), 7.60 (d, 1H, J = 8.0 Hz, NH), 7.42 (td, J = 7.5, 1.2 Hz, 2H, H_{ar}), 7.33 (td, 2H, J = 7.4, 1.2 Hz, H_{ar}), 4.59 (s, 1H, OH), 4.29 - 4.18 (m, 3H, CH-CH_2 Fmoc), 4.12 - 4.07 (m, 1H, α-CH), 3.50 - 3.43 (m, 2H, γ-CH_2), 1.92 - 1.69 (m, 2H, β-CH_2) ppm.

^{13}C NMR (101 MHz, DMSO-d6) δ = 174.36 (COOH), 156.19 (NH-COO), 143.86 (C_{ar}), 140.74 (C_{ar}), 127.68 (HC_{ar}), 127.12 (HC_{ar}), 125.32 (HC_{ar}), 120.16 (HC_{ar}), 65.64 (CH-**C**H_2 Fmoc), 57.24 (γ-CH_2), 50.83 (α-CH), 46.65 (**C**H-CH_2 Fmoc), 33.83 (β-CH_2) ppm.

4.3 Fmoc-D-Hse(Dmt)-OH

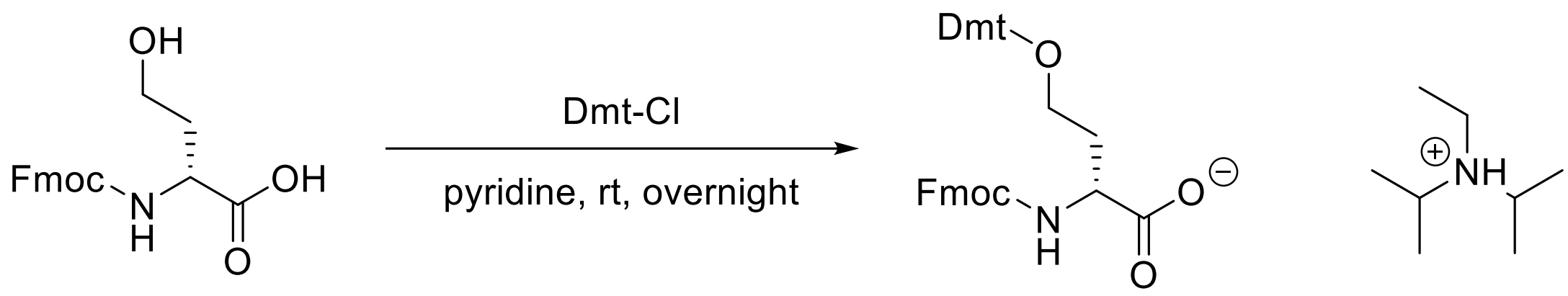

Fmoc-D-Hse-OH **Fmoc-D-Hse(Dmt)-OH**

Fmoc-D-Hse-OH (2.00 g, 5.96 mmol, 1.00 eq.) was dissolved in anhydrous pyridine (30 mL) in an N_2 atmosphere. Dmt-Cl (4.04 g, 11.9 mmol, 2.00 eq.) was added in four portions in 30 min intervals and the mixture was stirred overnight at rt. The solvent was evaporated under reduced pressure. The residue was dissolved in EtOAc (250 mL), washed with water (3 x 150 mL) and brine (150 mL) and dried over Na_2SO_4. After filtration, the solvent was evaporated under reduced pressure. The crude product was purified by column chromatography (silica gel, DCM:MeOH:DIPEA (95:4:1)). Product fractions were identified by TLC and the solvent was evaporated under reduced pressure. The remaining oil was dried in vacuum to yield a colourless foam.

Yield: 4.23 g, 5.47 mmol, 92% (as DIPEA salt).

ESI-MS: $[M+Na]^+$ 666.2453 (calculated: 666.2462), $[M+2Na-H]^+$ 688.2276 (calculated: 688.2293)

^{1}H NMR (400 MHz, DMSO-d_6) δ = 7.89 (d, 2H, J = 7.5 Hz, H_{ar}), 7.67 (t, 2H, J = 7.8 Hz, H_{ar}), 7.43-7.15 (m, 14H, H_{ar}), 6.86-6.82 (m, 4H, H_{ar}), 4.25-4.13 (m, 3H, C**H**-C**H**$_2$ Fmoc), 4.05-4.00 (m, 1H, α-CH), 3.68 (s, 4H, O-CH_3), 3.67 (s, 3H, O-CH_3), 3.16-3.05 (m, 5H, C**H**-$(CH_3)_2$ DIPEA, γ-CH_2), 2.99-2.94 (m, 1H, γ-CH_2), 2.56 (q, 4H, J = 7.2 Hz, C**H**$_2$-CH_3 DIPEA), 2.11-2.03 (m, 1H, β-CH_2), 1.89-1.80 (m, 1H, β-CH_2), 1.02-0.99 (m, 24H, CH_3 DIPEA) ppm.

^{13}C NMR (101 MHz, DMSO-d6) δ = 173.91 (COO^-), 157.93 (C_{ar}OMe), 155.76 (O-C(O)-NH), 145.22 (C_{ar}), 143.93 (C_{ar}), 143.81 (C_{ar}), 140.69 (C_{ar}), 139.44 (C_{ar}), 137.44 (C_{ar}), 136.06 (C_{ar}), 135.99 (C_{ar}), 135.95 (C_{ar}), 129.63 (C_{ar}), 129.60 (C_{ar}), 128.94 (C_{ar}), 127.79 (C_{ar}), 127.73 (C_{ar}), 127.66 (C_{ar}), 127.30 (C_{ar}), 126.56 (Car), 127.51 (C_{ar}), 125.29 (C_{ar}), 125.23 (C_{ar}), 121.40 (C_{ar}), 120.11 (C_{ar}), 120.09 (C_{ar}), 120.05 (C_{ar}), 113.12 (C_{ar}), 113.05 (C_{ar}), 109.78 (C_{ar}), 85.25 ($C(Ph)_3$), 65.56 (CH-**C**H_2 Fmoc), 59.83 (γ-CH_2), 52.13 (O-CH3), 51.65 (α-CH), 48.96 (**C**H-CH_3 DIPEA), 46.69 (**C**H-CH_2 Fmoc), 31.95 (β-CH_2), 19.78 (CH-**C**H_3 DIPEA), 15.61 (CH_2-**C**H_3 DIPEA) ppm.

Abbreviation

Ac	Acetyl
Boc	*tert*-Butoxycarbonyl
Bz	Benzyl
Bz-nKRR-AMC	Benzoyl-norleucine-lysine-arginine-arginine-(7-amino-4-methylcoumarin)
CHAPS	3-[(3-cholamidopropyl)dimethylammonio]-1-propanesulfonate
COSY	Correlation spectroscopy
DCM	Dichloromethane
DENV2	Dengue virus serotype 2
DHB	2,5-Dihydroxybenzoic acid
DIC	*N*,*N′*-Diisopropylcarbodiimide
DIPEA	*N*,*N*-Diisopropylethylamine
DMF	*N*,*N*-Dimethylformamide
DMSO	Dimethyl sulfoxide
Dmt	4,4'-Dimethoxytrityl
Et_2O	Diethyl ether
EtOAc	Ethyl acetate
Fmoc	9*H*-Fluoren-9-ylmethoxycarbonyl
Hepes	2-[4-(2-Hydroxyethyl)piperazin-1-yl]ethane-1-sulfonic acid
HPLC	High performance liquid chromatography
Hse	Homoserine
HMBC	Heteronuclear multiple bond coherence
HSQC	Heteronuclear single quantum coherence
IC_{50}	Half-maximal inhibitory concentration
IrAAC	Iridium-catalyzed azide-alkyne cycloaddition
LSF	Late-stage-functionalization
MALDI	Matrix assisted laser desorption ionisation
MBHA	4-Methylbenzhydrylamine
MeCN	Acetonitrile
MeOH	Methanol
MS	Mass spectrometry
MTPI	Methyltriphenoxyphosphonium iodide
Nle	Norleucine
NS2B	Non-structural protein 2B
NS3	Non-structural protein 3
Oxyma	Ethyl cyano(hydroxyimino)acetate
Phg	Phenylglycine
PyBOP	Benzotriazol-1-yloxytripyrrolidinophosphonium hexafluorophosphate
RP	Reversed phase
rt	Room temperature
SPPS	Solid-phase peptide synthesis
tBu	Tertbutyl
TFA	Trifluoroacetic acid
TIPS	Triisopropyl silane
TLC	Thin-layer chromatography
TMSBr	Trimethylsilyl bromide
TOF	Time-of-flight

5 References

[1] J. B. Crumpton, W. Zhang, W. L. Santos, *Anal. Chem.* **2011**, *83*, 3548-3554.
[2] C. Steuer, K. H. Heinonen, L. Kattner, C. D. Klein, *J. Biomol. Screen.* **2009**, *14*, 1102-1108.
[3] M. A. M. Behnam, C. D. Klein, *Biochimie* **2024**, *227*, 15-27.
[4] J. Lang, S. K. Dutta, M. M. Leuthold, L. Reichert, N. Kühl, B. Martina, C. D. Klein, *Antiviral Res.* **2025**, *234*, 106053.
[5] M. Werner, J. Pampel, T. L. Pham, F. Thomas, *Chem. Eur. J.* **2022**, *28*, e202201339.
